# Supplementary material for: Electrochemical and Structural Characterization of Lanthanum-Doped Hydroxyapatite: A Promising Material for Sensing Applications
Source: Materials (Basel). 2023 Jun 22;16(13):4522. doi: 10.3390/ma16134522 (PMC10342981; doi:10.3390/ma16134522)
Supplement: Supplementary file 1 [file materials-16-04522-s001.zip › materials-2407697-supplementary.pdf]

SUPPORTING INFORMATIONS

Electrochemical and structural characterization of lanthanum-doped hydroxyapatite: a promising material for sensing applications

Rocco Cancelliere [1,*], Giuseppina Rea [2], Laura Micheli [1], Pietro Mantegazza [1], Elvira Maria Bauer [3], Asmaa El Khouri [4], Emanuela Tempesta [5], Angela Altomare [6], Davide Capelli [2] and Francesco Capitelli [2,*]

[1] Dipartimento di Scienze e Tecnologie Chimiche, Università degli Studi di Roma Tor Vergata, Via della Ricerca Scientifica, 00133 Rome, Italy; laura.micheli@uniroma2.it (L.M.); mantegazza.pietro@gmail.com (P.M.)

[2] Institute of Crystallography (IC), National Research Council (CNR), Via Salaria Km 29.300, 00016 Rome, Italy; giuseppina.rea@ic.cnr.it (G.R.); davide.capelli@ic.cnr.it (D.C.)

[3] Institute of Structure of Matter (ISM), National Research Council (CNR), Via Salaria Km 29.300, 00016 Rome, Italy; elvira.bauer@ism.cnr.it

[4] Faculté des Sciences Semlalia, BP 2390, Université Cadi Ayyad, Marrakech 40000, Morocco; elkhouriasma@gmail.com

[5] Institute of Environmental Geology and Geoengineering (IGAG), National Research Council (CNR), Via Salaria Km 29.300, 00016 Rome, Italy; emanuela.tempesta@igag.cnr.it

[6] Institute of Crystallography (IC), National Research Council (CNR), Via Amendola 122/o, 70100 Bari, Italy; angela.altomare@ic.cnr.it

* Correspondence: rocco.cancelliere@uniroma2.it (R.C.); francesco.capitelli@ic.cnr.it (F.C.); Tel.: +39-06-0672594737 (R.C.); +39-06-90672616 (F.C.)

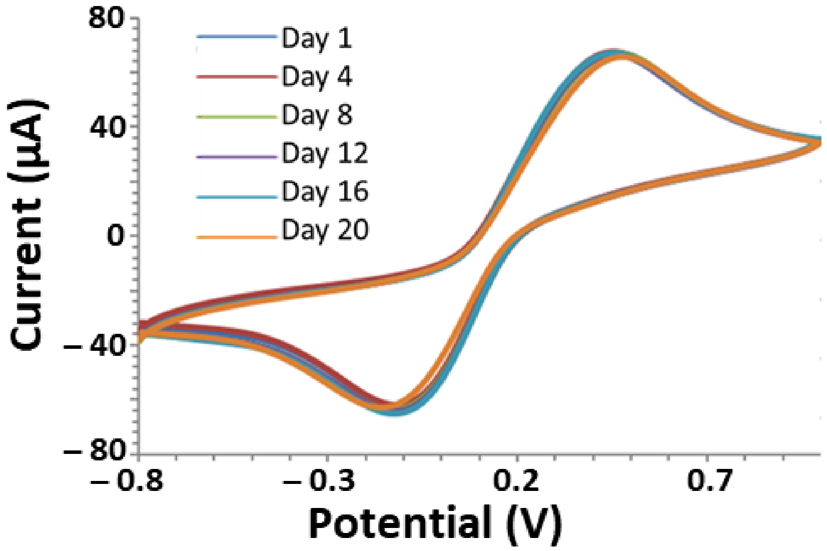

Figure S1. Electrochemical characterization of the stability using cyclic voltammetry as an analytical tool and $[Fe(CN)_6]^{3-/4-}$ as a redox couple.

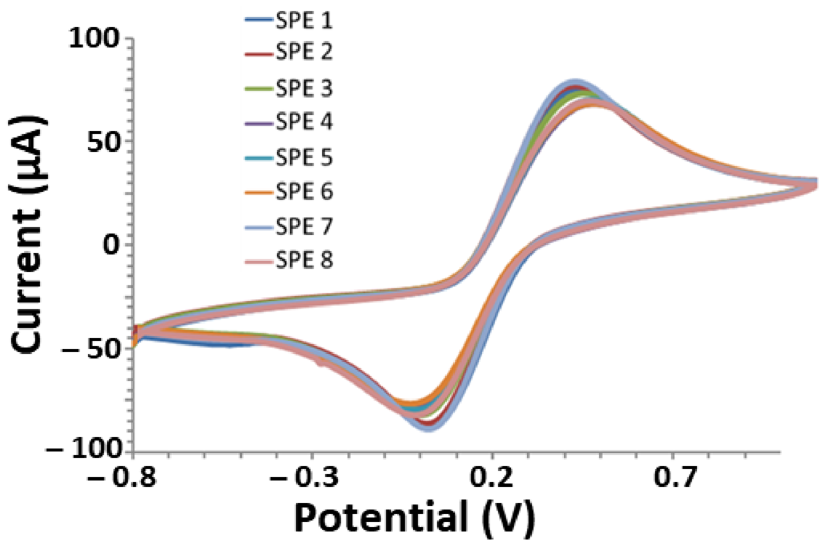

Figure S2. Electrochemical characterization of the reproducibility using cyclic voltammetry as an analytical tool and $[Fe(CN)_6]^{3-/4-}$ as a redox couple.

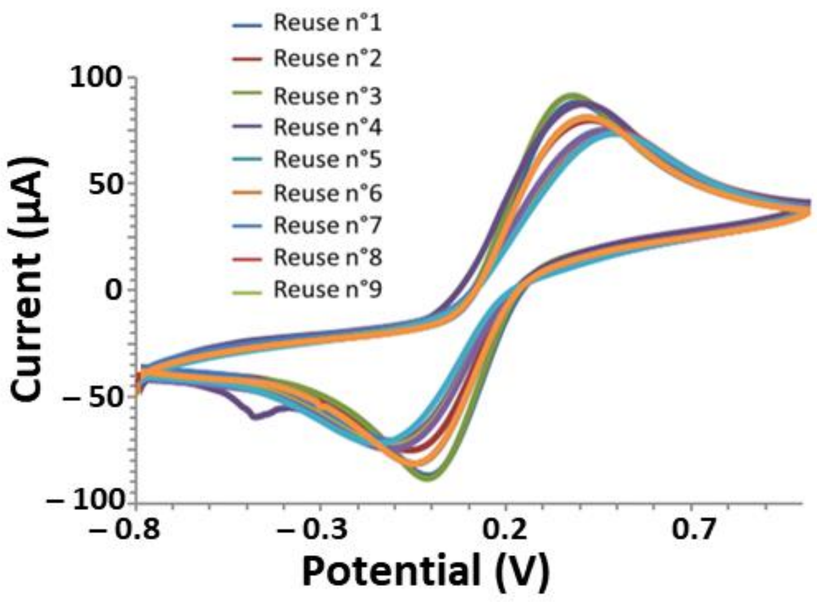

Figure S3. Electrochemical characterization of the reusability using cyclic voltammetry as an analytical tool and $[Fe(CN)_6]^{3-/4-}$ as a redox couple.

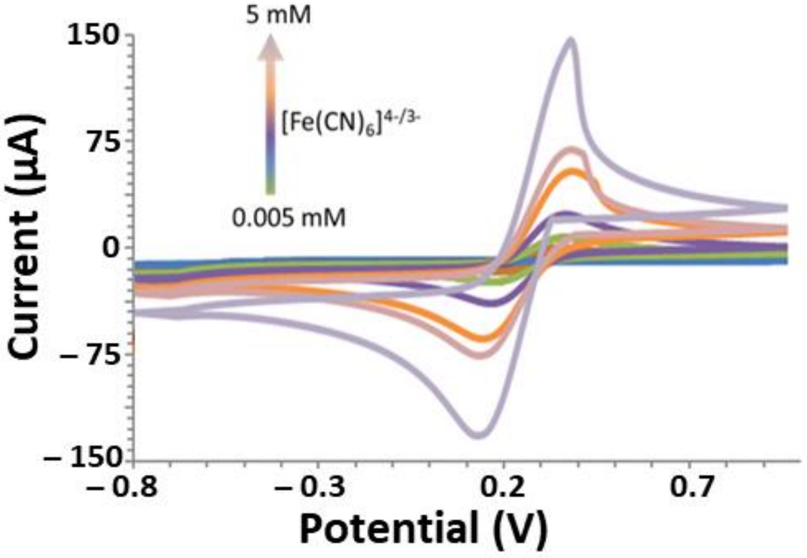

Figure S4. Electrochemical characterization of the sensitivity using cyclic voltammetry as an analytical tool, La3-doped_HAp-modified-SPEs, and several concentrations of $[Fe(CN)_6]^{3-/4-}$. The limit of detection (LOD 1.7μM and 19.2 μM for La3-doped_HAp-modified-SPEs and bare SPE, respectively) a is calculated (3·σ/δ) using the slope of the relative linear regression whose equation is y = 13.03x + 0.51, R^2 = 0.9997.

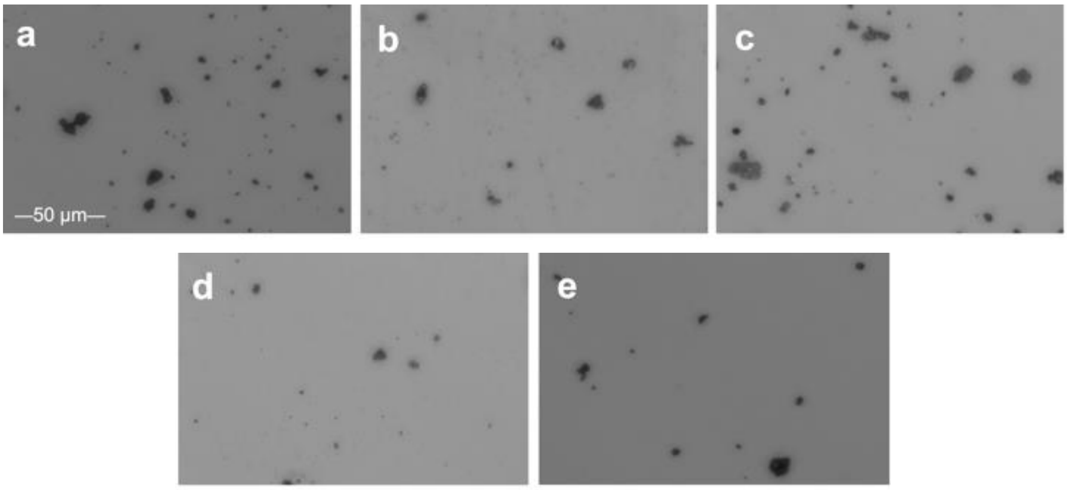

Figure S5. Optical microscopy images of HAp samples: pure HAp (a), La1-HAp (b), La2-HAp (c), La3-HAp (d), La4-HAp (e).

Energy Dispersive Spectroscopy - EDS Bruker Quantax 200, LN2 free

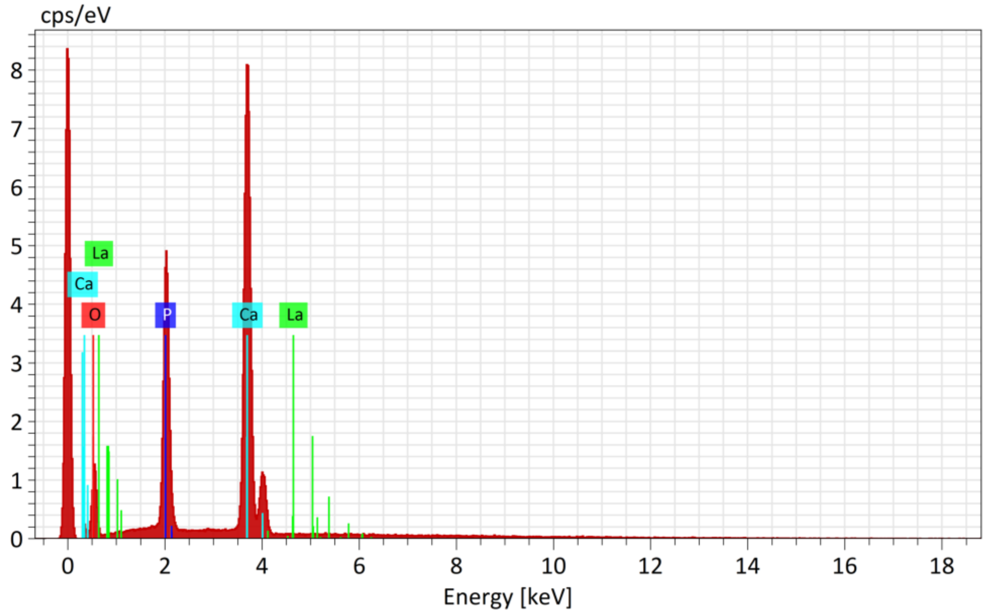

Figure S6. EDS qualitative spectrum of La1-HAp.

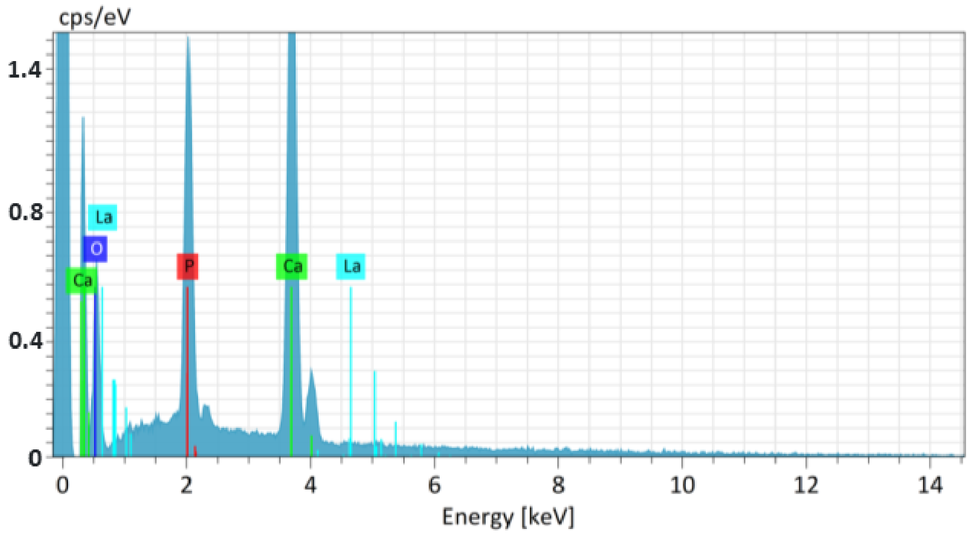

Figure S7. EDS qualitative spectrum of La2-HAp.

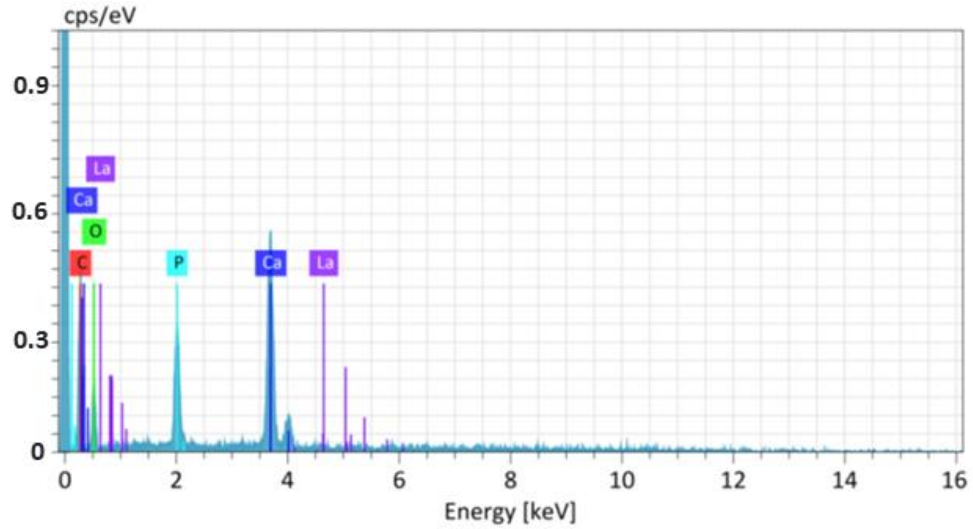

Figure S8. EDS qualitative spectrum of La3-HAp.

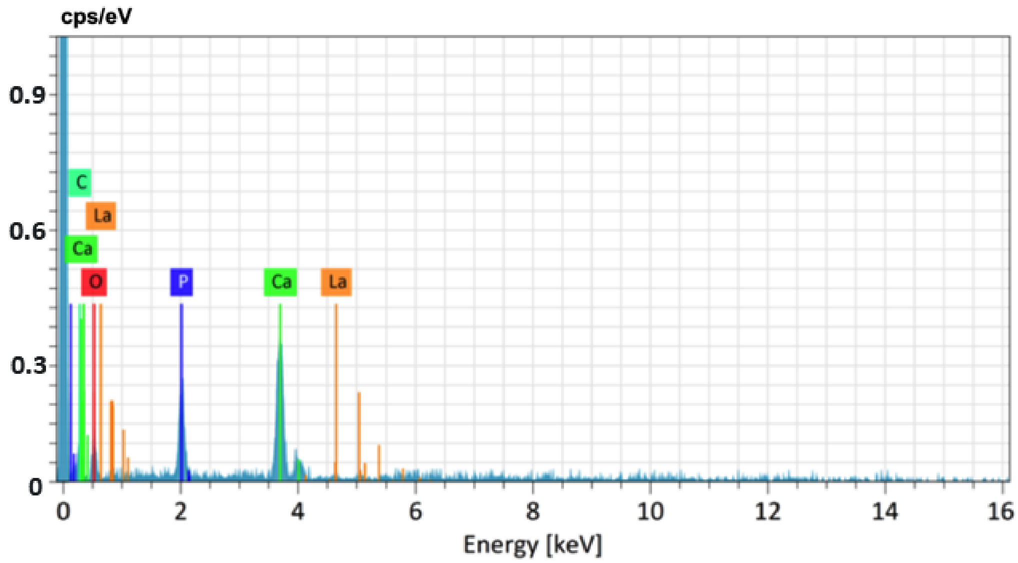

Figure S9. EDS qualitative spectrum of La4-HAp.

La1-HAP CIF FILE

```
#==============================================================================
data_global
#==============================================================================

_publ_contact_author_name  'Dr. Francesco Capitelli'
_publ_contact_author_address
;
  Institute of Crystallography - CNR
  V. Salaria Km 29.300, 00015 Monterotondo (Rome), Italy
;
_publ_contact_author_email    francesco.capitelli@ic.cnr.it
_publ_contact_author_fax     '+39 06 90672616'
_publ_contact_author_phone   '+39 06 90672630'
_journale_name_full          ?
_publ_requested_category     FI
_audit_creation_method              Expo2014

_chemical_name_systematic     ?
_chemical_formula_moiety      'H Ca5 La0.01 O13 P3'
_chemical_formula_sum         'H Ca5 La0.01 O13 P3'
_chemical_formula_weight      502.805

loop_
     _atom_type_symbol
     _atom_type_description
     _atom_type_scat_source
    'H'   'Hydrogen'   'International Tables Vol C Tables 4.2.6.8 and 6.1.1.4'
    'O'   'Oxygen'   'International Tables Vol C Tables 4.2.6.8 and 6.1.1.4'
    'P'   'Phosphorus'   'International Tables Vol C Tables 4.2.6.8 and 6.1.1.4'
    'Ca'   'Calcium'   'International Tables Vol C Tables 4.2.6.8 and 6.1.1.4'
    'La'   'Lanthanum'   'International Tables Vol C Tables 4.2.6.8 and 6.1.1.4'

_cell_length_a                   9.4146(3)
_cell_length_b                   9.4146(3)
_cell_length_c                   6.8834(4)
```

```
_cell_angle_alpha                90.000
_cell_angle_beta                 90.000
_cell_angle_gamma                120.000
_cell_volume                     528.36(4)
_cell_formula_units_Z            2
_exptl_crystal_description    powder
_exptl_crystal_colour           ?
_cell_measurement_temperature    ?

_exptl_crystal_density_diffrn         3.160
_exptl_crystal_density_meas      ?
_exptl_crystal_density_method 'not measured'
_exptl_absorpt_coefficient_mu        27.393
_symmetry_Int_Tables_number         176
_symmetry_cell_setting              hexagonal
_symmetry_space_group_name_H-M      'P 63/m'
_symmetry_space_group_name_hall     '-P 6c'

loop_
    _symmetry_equiv_pos_site_id
    _symmetry_equiv_pos_as_xyz
    1   'x, y, z'
    2   'x-y, x, z+1/2'
    3   '-y, x-y, z'
    4   '-x, -y, z+1/2'
    5   '-x+y, -x, z'
    6   'y, -x+y, z+1/2'
    7   '-x, -y, -z'
    8   '-x+y, -x, -z+1/2'
    9   'y, -x+y, -z'
   10   'x, y, -z+1/2'
   11   'x-y, x, -z'
   12   '-y, x-y, -z+1/2'

loop_
    _atom_site_type_symbol
    _atom_site_label
    _atom_site_fract_x
```

```
    _atom_site_fract_y
    _atom_site_fract_z
    _atom_site_U_iso_or_equiv
    _atom_site_occupancy
    _atom_site_adp_type
Ca  Ca1 0.6667 0.3333 1.0007(11) 0.0040(7) 1.0000        Uiso
Ca  Ca2 0.2536(4) 0.2464(5) 0.7500 0.0038 0.998(3) Uiso
La  La1 0.2536 0.2464 0.7500 0.0038(5) 0.002(3) Uiso
P   P   0.3983(7) 0.3676(7) 1.2500 0.0010(7) 1.0000        Uiso
O   O1  0.3257(14) 0.4844(14) 1.2500 0.010(2) 1.0000        Uiso
O   O2  0.5873(15) 0.4653(15) 1.2500 0.008(2) 1.0000        Uiso
O   O3  0.3414(9) 0.2560(9) 1.0685(10) 0.0125(15) 1.0000        Uiso
O   O-H 0.0000 0.0000 0.700(4) 0.017(5) 0.50000       Uiso
H   H   0.0000 0.0000 0.5611 0.0206 0.50000       Uiso

loop_
    _geom_bond_atom_site_label_1
    _geom_bond_atom_site_label_2
    _geom_bond_distance
    _geom_bond_site_symmetry_2
Ca1 O1  2.409(8) 6_554
Ca1 O1  2.409(12) 4_664
Ca1 O1  2.409(12) 2_654
Ca1 O2  2.445(13) .
Ca1 O2  2.445(11) 5_665
Ca1 O2  2.445(10) 3_655
Ca1 O3  2.810(9) .
Ca1 O3  2.810(7) 5_665
Ca1 O3  2.810(10) 3_655
Ca2 O1  2.683(16) 6_554
Ca2 O2  2.354(13) 4_664
Ca2 O3  2.329(8) 10_556
Ca2 O3  2.507(11) 2_554
Ca2 O3  2.507(11) 11_557
Ca2 O3  2.329(8) .
Ca2 O-H  2.379(5) .
P O1  1.558(18) .
P O2  1.541(13) .
```

```
P O3  1.546(8) .
P O3  1.546(8) 10_557
O1 Ca1  2.409(12) 7_667
O1 Ca1  2.409(12) 2_555
O1 Ca2  2.683(10) 2_555
O2 Ca1  2.445(13) 8_667
O2 Ca2  2.354(13) 4_665
O3 Ca2  2.507(9) 6_555
O-H Ca2  2.379(6) 3_555
O-H Ca2  2.379(6) 5_555
O-H H  0.957477 .

loop_
_geom_angle_atom_site_label_1
_geom_angle_atom_site_label_2
_geom_angle_atom_site_label_3
_geom_angle
_geom_angle_site_symmetry_1
_geom_angle_site_symmetry_3
O1 Ca1 O1 74.4(4) 6_554 4_664
O1 Ca1 O1 74.4(4) 6_554 2_654
O1 Ca1 O2 123.7(4) 6_554 .
O1 Ca1 O2 154.8(4) 6_554 5_665
O1 Ca1 O2 92.8(4) 6_554 3_655
O1 Ca1 O3 68.3(3) 6_554 .
O1 Ca1 O3 141.5(4) 6_554 5_665
O1 Ca1 O3 86.8(3) 6_554 3_655
O1 Ca1 O1 74.4(4) 4_664 2_654
O1 Ca1 O2 92.8(4) 4_664 .
O1 Ca1 O2 123.7(4) 4_664 5_665
O1 Ca1 O2 154.8(4) 4_664 3_655
O1 Ca1 O3 86.8(3) 4_664 .
O1 Ca1 O3 68.3(3) 4_664 5_665
O1 Ca1 O3 141.5(4) 4_664 3_655
O1 Ca1 O2 154.8(4) 2_654 .
O1 Ca1 O2 92.8(4) 2_654 5_665
O1 Ca1 O2 123.7(4) 2_654 3_655
O1 Ca1 O3 141.5(4) 2_654 .
```

O1 Ca1 O3 86.8(3) 2_654 5_665

O1 Ca1 O3 68.3(3) 2_654 3_655

O2 Ca1 O2 76.2(4) . 5_665

O2 Ca1 O2 76.2(4) . 3_655

O2 Ca1 O3 56.3(3) . .

O2 Ca1 O3 68.2(3) . 5_665

O2 Ca1 O3 125.3(4) . 3_655

Ca1 O2 P 104.6(6) . .

Ca1 O2 Ca1 89.2(4) . 8_667

Ca1 O2 Ca2 113.9(5) . 4_665

O2 Ca1 O2 76.2(4) 5_665 3_655

O2 Ca1 O3 125.3(4) 5_665 .

O2 Ca1 O3 56.3(3) 5_665 5_665

O2 Ca1 O3 68.2(3) 5_665 3_655

O2 Ca1 O3 68.2(3) 3_655 .

O2 Ca1 O3 125.3(4) 3_655 5_665

O2 Ca1 O3 56.3(3) 3_655 3_655

O3 Ca1 O3 117.3(3) . 5_665

O3 Ca1 O3 117.3(3) . 3_655

Ca1 O3 Ca2 100.0(3) . .

Ca1 O3 P 89.8(4) . .

Ca1 O3 Ca2 98.2(3) . 6_555

O3 Ca1 O3 117.3(3) 5_665 3_655

O1 Ca2 O2 102.0(4) 6_554 4_664

O1 Ca2 O3 71.7(3) 6_554 10_556

O1 Ca2 O3 149.7(3) 6_554 2_554

O1 Ca2 O3 149.7(3) 6_554 11_557

O1 Ca2 O3 71.7(3) 6_554 .

O1 Ca2 O-H 105.8(4) 6_554 .

O2 Ca2 O3 86.9(4) 4_664 10_556

O2 Ca2 O3 74.9(4) 4_664 2_554

O2 Ca2 O3 74.9(4) 4_664 11_557

O2 Ca2 O3 86.9(4) 4_664 .

O2 Ca2 O-H 150.9(5) 4_664 .

O3 Ca2 O3 78.0(3) 10_556 2_554

O3 Ca2 O3 136.9(3) 10_556 11_557

O3 Ca2 O3 140.6(3) 10_556 .

O3 Ca2 O-H 93.8(4) 10_556 .

```
O3 Ca2 O3 59.8(3) 2_554 11_557
O3 Ca2 O3 136.9(3) 2_554 .
O3 Ca2 O-H 76.8(4) 2_554 .
O3 Ca2 O3 78.0(3) 11_557 .
O3 Ca2 O-H 85.1(4) 11_557 .
O3 Ca2 O-H 109.7(4) . .
Ca2 O3 P 141.5(5) . .
Ca2 O3 Ca2 119.1(4) . 6_555
Ca2 O-H Ca2 118.0(6) . 3_555
Ca2 O-H Ca2 118.0(6) . 5_555
Ca2 O-H H      98.29       .       .
O1 P O2 111.2(7) . .
O1 P O3 110.8(6) . .
O1 P O3 110.8(6) . 10_557
P O1 Ca1 129.4(6) . 7_667
P O1 Ca1 129.4(6) . 2_555
P O1 Ca2 97.8(5) . 2_555
O2 P O3 108.1(6) . .
O2 P O3 108.1(6) . 10_557
P O2 Ca1 104.6(6) . 8_667
P O2 Ca2 124.6(7) . 4_665
O3 P O3 107.9(5) . 10_557
P O3 Ca2 95.8(4) . 6_555
Ca1 O1 Ca1 91.5(4) 7_667 2_555
Ca1 O1 Ca2 101.5(4) 7_667 2_555
Ca1 O1 Ca2 101.5(4) 2_555 2_555
Ca1 O2 Ca2 113.9(5) 8_667 4_665
Ca2 O-H Ca2 118.0(6) 3_555 5_555
Ca2 O-H H      98.29   3_555       .
Ca2 O-H H      98.29   5_555       .

loop_
   _geom_torsion_atom_site_label_1
   _geom_torsion_atom_site_label_2
   _geom_torsion_atom_site_label_3
   _geom_torsion_atom_site_label_4
   _geom_torsion
   _geom_torsion_site_symmetry_1
```

_geom_torsion_site_symmetry_2
_geom_torsion_site_symmetry_3
_geom_torsion_site_symmetry_4
O1 Ca1 O1 Ca2 -63.2(4) 4_664 . 6_554 .
O1 Ca1 O1 Ca2 -141.0(4) 2_654 . 6_554 .
O2 Ca1 O1 Ca2 19.5(5) . . 6_554 .
O2 Ca1 O1 Ca2 157.4(8) 5_665 . 6_554 .
O2 Ca1 O1 Ca2 94.7(4) 3_655 . 6_554 .
O3 Ca1 O1 Ca2 29.6(3) . . 6_554 .
O3 Ca1 O1 Ca2 -77.4(6) 5_665 . 6_554 .
O3 Ca1 O1 Ca2 150.7(3) 3_655 . 6_554 .
O1 Ca1 O2 P 18.6(6) 6_554 . . .
O1 Ca1 O2 Ca1 123.4(4) 6_554 . . 8_667
O1 Ca1 O2 Ca2 -120.6(5) 6_554 . . 4_665
O1 Ca1 O2 P 91.6(5) 4_664 . . .
O1 Ca1 O2 Ca1 -163.5(4) 4_664 . . 8_667
O1 Ca1 O2 Ca2 -47.6(4) 4_664 . . 4_665
O1 Ca1 O2 P 149.6(8) 2_654 . . .
O1 Ca1 O2 Ca1 -105.5(9) 2_654 . . 8_667
O1 Ca1 O2 Ca2 10.5(11) 2_654 . . 4_665
O2 Ca1 O2 P -144.3(5) 5_665 . . .
O2 Ca1 O2 Ca1 -39.4(4) 5_665 . . 8_667
O2 Ca1 O2 Ca2 76.5(4) 5_665 . . 4_665
O2 Ca1 O2 P -65.4(5) 3_655 . . .
O2 Ca1 O2 Ca1 39.4(4) 3_655 . . 8_667
O2 Ca1 O2 Ca2 155.4(5) 3_655 . . 4_665
O3 Ca1 O2 P 7.3(4) . . . .
O3 Ca1 O2 Ca1 112.2(4) . . . 8_667
O3 Ca1 O2 Ca2 -131.8(5) . . . 4_665
O3 Ca1 O2 P 156.9(5) 5_665 . . .
O3 Ca1 O2 Ca1 -98.3(3) 5_665 . . 8_667
O3 Ca1 O2 Ca2 17.7(4) 5_665 . . 4_665
O3 Ca1 O2 P -94.4(5) 3_655 . . .
O3 Ca1 O2 Ca1 10.5(5) 3_655 . . 8_667
O3 Ca1 O2 Ca2 126.5(4) 3_655 . . 4_665
O1 Ca1 O3 Ca2 -34.5(4) 6_554 . . .
O1 Ca1 O3 P -177.0(5) 6_554 . . .
O1 Ca1 O3 Ca2 87.2(4) 6_554 . . 6_555

O1 Ca1 O3 Ca2 40.0(4) 4_664 . . .

O1 Ca1 O3 P -102.6(4) 4_664 . . .

O1 Ca1 O3 Ca2 161.6(3) 4_664 . . 6_555

O1 Ca1 O3 Ca2 -19.7(6) 2_654 . . .

O1 Ca1 O3 P -162.3(6) 2_654 . . .

O1 Ca1 O3 Ca2 101.9(6) 2_654 . . 6_555

O2 Ca1 O3 Ca2 135.5(4) . . . .

O2 Ca1 O3 P -7.1(4) . . . .

O2 Ca1 O3 Ca2 -102.9(4) . . . 6_555

O2 Ca1 O3 Ca2 169.9(4) 5_665 . . .

O2 Ca1 O3 P 27.3(5) 5_665 . . .

O2 Ca1 O3 Ca2 -68.5(5) 5_665 . . 6_555

O2 Ca1 O3 Ca2 -136.9(4) 3_655 . . .

O2 Ca1 O3 P 80.5(4) 3_655 . . .

O2 Ca1 O3 Ca2 -15.3(4) 3_655 . . 6_555

O3 Ca1 O3 Ca2 103.5(3) 5_665 . . .

O3 Ca1 O3 P -39.1(4) 5_665 . . .

O3 Ca1 O3 Ca2 -134.8(3) 5_665 . . 6_555

O3 Ca1 O3 Ca2 -108.6(3) 3_655 . . .

O3 Ca1 O3 P 108.8(4) 3_655 . . .

O3 Ca1 O3 Ca2 13.0(4) 3_655 . . 6_555

O2 Ca2 O1 Ca1 47.0(4) 4_664 . 6_554 .

O3 Ca2 O1 Ca1 129.6(4) 10_556 . 6_554 .

O3 Ca2 O1 Ca1 127.7(6) 2_554 . 6_554 .

O3 Ca2 O1 Ca1 -33.7(7) 11_557 . 6_554 .

O3 Ca2 O1 Ca1 -35.7(3) . . 6_554 .

O-H Ca2 O1 Ca1 -141.7(6) . . 6_554 .

O1 Ca2 O3 Ca1 29.8(3) 6_554 . . .

O1 Ca2 O3 P 132.2(7) 6_554 . . .

O1 Ca2 O3 Ca2 -75.5(4) 6_554 . . 6_555

O2 Ca2 O3 Ca1 -73.9(4) 4_664 . . .

O2 Ca2 O3 P 28.5(7) 4_664 . . .

O2 Ca2 O3 Ca2 -179.2(4) 4_664 . . 6_555

O3 Ca2 O3 Ca1 7.5(6) 10_556 . . .

O3 Ca2 O3 P 109.9(7) 10_556 . . .

O3 Ca2 O3 Ca2 -97.9(5) 10_556 . . 6_555

O3 Ca2 O3 Ca1 -137.9(4) 2_554 . . .

O3 Ca2 O3 P -35.5(9) 2_554 . . .

O3 Ca2 O3 Ca2 116.8(4) 2_554 . . 6_555

O3 Ca2 O3 Ca1 -149.2(3) 11_557 . . .

O3 Ca2 O3 P -46.8(7) 11_557 . . .

O3 Ca2 O3 Ca2 105.5(4) 11_557 . . 6_555

O-H Ca2 O3 Ca1 130.5(6) . . . .

O-H Ca2 O3 P -127.1(8) . . . .

O-H Ca2 O3 Ca2 25.2(7) . . . 6_555

O1 Ca2 O-H Ca2 168.3(6) 6_554 . . 3_555

O1 Ca2 O-H Ca2 16.4(8) 6_554 . . 5_555

O1 Ca2 O-H H -87.64 6_554 . . .

O2 Ca2 O-H Ca2 -29.2(14) 4_664 . . 3_555

O2 Ca2 O-H Ca2 178.8(7) 4_664 . . 5_555

O2 Ca2 O-H H 74.81 4_664 . . .

O3 Ca2 O-H Ca2 -119.7(6) 10_556 . . 3_555

O3 Ca2 O-H Ca2 88.4(7) 10_556 . . 5_555

O3 Ca2 O-H H -15.64 10_556 . . .

O3 Ca2 O-H Ca2 -42.9(6) 2_554 . . 3_555

O3 Ca2 O-H Ca2 165.1(7) 2_554 . . 5_555

O3 Ca2 O-H H 61.11 2_554 . . .

O3 Ca2 O-H Ca2 17.2(6) 11_557 . . 3_555

O3 Ca2 O-H Ca2 -134.8(7) 11_557 . . 5_555

O3 Ca2 O-H H 121.19 11_557 . . .

O3 Ca2 O-H Ca2 92.6(7) . . . 3_555

O3 Ca2 O-H Ca2 -59.4(7) . . . 5_555

O3 Ca2 O-H H -163.40 . . . .

O2 P O1 Ca1 68.0(7) . . . 7_667

O2 P O1 Ca1 -68.0(7) . . . 2_555

O2 P O1 Ca2 180.0(5) . . . 2_555

O3 P O1 Ca1 -52.2(7) . . . 7_667

O3 P O1 Ca1 171.8(5) . . . 2_555

O3 P O1 Ca2 59.8(5) . . . 2_555

O3 P O1 Ca1 -171.8(5) 10_557 . . 7_667

O3 P O1 Ca1 52.2(7) 10_557 . . 2_555

O3 P O1 Ca2 -59.8(5) 10_557 . . 2_555

O1 P O2 Ca1 -133.5(5) . . . .

O1 P O2 Ca1 133.5(5) . . . 8_667

O1 P O2 Ca2 0.0(7) . . . 4_665

O3 P O2 Ca1 -11.7(6) . . . .

```
O3 P O2 Ca1 -104.7(5) . . . 8_667
O3 P O2 Ca2 121.8(5) . . . 4_665
O3 P O2 Ca1 104.7(5) 10_557 . . .
O3 P O2 Ca1 11.7(6) 10_557 . . 8_667
O3 P O2 Ca2 -121.8(5) 10_557 . . 4_665
O1 P O3 Ca1 131.9(4) . . . .
O1 P O3 Ca2 26.0(8) . . . .
O1 P O3 Ca2 -129.9(4) . . . 6_555
O2 P O3 Ca1 9.9(5) . . . .
O2 P O3 Ca2 -96.0(7) . . . .
O2 P O3 Ca2 108.1(5) . . . 6_555
O3 P O3 Ca1 -106.7(4) 10_557 . . .
O3 P O3 Ca2 147.4(6) 10_557 . . .
O3 P O3 Ca2 -8.5(5) 10_557 . . 6_555

_diffrn_ambient_temperature     ?
_diffrn_radiation_wavelength     1.540560
_diffrn_radiation_type          'Cu K\a~1~'
_diffrn_measurement_device_type ?

# POWDER PROFILE

_pd_meas_2theta_range_min      8.000
_pd_meas_2theta_range_max    120.000
_pd_proc_number_of_points       5601

_pd_proc_ls_prof_R_factor           0.04205
_pd_proc_ls_prof_wR_factor          0.06814
_pd_proc_ls_prof_wR_expected        0.06070

_refine_ls_number_reflns      288
_refine_ls_number_parameters  55
_refine_ls_number_restraints  0
_refine_ls_hydrogen_treatment  constr
_refine_ls_R_I_factor               0.16815
_refine_ls_goodness_of_fit_all      1.12257
_refine_ls_restrained_S_all         1.12257
```

```
loop_
     _pd_meas_2theta_scan
     _pd_meas_counts_total
     _pd_proc_2theta_corrected
     _pd_calc_intensity_total
     _pd_proc_intensity_bkg_calc
```

8.000000	1799.669922	7.999248	0.000000	1805.242920
8.020000	1788.500000	8.019247	0.000000	1799.663574
8.040000	1785.500000	8.039247	0.000000	1794.123535
8.060000	1777.669922	8.059247	0.000000	1788.619629
8.080000	1736.169922	8.079247	0.000000	1783.153320
8.100000	1741.669922	8.099247	0.000000	1777.723145
8.120001	1760.330078	8.119247	0.000000	1772.329834
8.140000	1747.000000	8.139247	0.000000	1766.972168
8.160001	1729.500000	8.159247	0.000000	1761.650146
8.180000	1744.169922	8.179247	0.000000	1756.364502
8.200000	1763.669922	8.199246	0.000000	1751.112793
8.220000	1759.169922	8.219248	0.000000	1745.895996
8.240000	1723.669922	8.239247	0.000000	1740.712646
8.260000	1711.330078	8.259248	0.000000	1735.564453
8.280000	1728.830078	8.279247	0.000000	1730.448486
8.300000	1713.830078	8.299248	0.000000	1725.366455
8.320001	1681.169922	8.319248	0.000000	1720.317139
8.340000	1692.330078	8.339248	0.000000	1715.301514
8.360001	1719.669922	8.359248	0.000000	1710.316650
8.380000	1715.330078	8.379247	0.000000	1705.364258
8.400000	1702.500000	8.399246	0.000000	1700.443848
8.420000	1674.169922	8.419247	0.000000	1695.554199
8.440000	1687.330078	8.439246	0.000000	1690.695557
8.460000	1700.000000	8.459247	0.000000	1685.867432
8.480000	1684.169922	8.479246	0.000000	1681.070312
8.500000	1697.330078	8.499247	0.000000	1676.302490
8.520000	1676.330078	8.519247	0.000000	1671.564453
8.540000	1661.830078	8.539247	0.000000	1666.856201
8.560000	1678.330078	8.559247	0.000000	1662.177246
8.580000	1665.169922	8.579247	0.000000	1657.526855
8.600000	1671.330078	8.599247	0.000000	1652.904785

8.620001	1614.000000	8.619247	0.000000	1648.311279
8.640000	1646.000000	8.639247	0.000000	1643.746338
8.660001	1657.330078	8.659247	0.000000	1639.208740
8.680000	1617.500000	8.679247	0.000000	1634.697998
8.700000	1635.000000	8.699246	0.000000	1630.214600
8.720000	1629.830078	8.719247	0.000000	1625.758789
8.740000	1637.830078	8.739246	0.000000	1621.328857
8.760000	1618.669922	8.759247	0.000000	1616.926514
8.780000	1625.330078	8.779245	0.000000	1612.549072
8.800000	1597.669922	8.799246	0.000000	1608.197510
8.820001	1605.330078	8.819246	0.000000	1603.871826
8.840000	1613.669922	8.839246	0.000000	1599.572266
8.860001	1604.169922	8.859246	0.000000	1595.296875
8.880000	1582.000000	8.879246	0.000000	1591.046387
8.900000	1581.669922	8.899245	0.000000	1586.820068
8.920000	1601.669922	8.919246	0.000000	1582.618652
8.940000	1579.500000	8.939245	0.000000	1578.441406
8.960000	1598.500000	8.959246	0.000000	1574.288086
8.980000	1565.669922	8.979245	0.000000	1570.157959
9.000000	1568.500000	8.999246	0.000000	1566.051270
9.020000	1586.330078	9.019246	0.000000	1561.968018
9.040000	1572.669922	9.039246	0.000000	1557.907227
9.060000	1546.000000	9.059246	0.000000	1553.869629
9.080000	1563.669922	9.079246	0.000000	1549.854980
9.100000	1545.500000	9.099246	0.000000	1545.861816
9.120001	1546.669922	9.119246	0.000000	1541.890625
9.140000	1524.669922	9.139246	0.000000	1537.941406
9.160001	1533.500000	9.159245	0.000000	1534.013672
9.180000	1537.000000	9.179245	0.000000	1530.108154
9.200000	1538.330078	9.199244	0.000000	1526.223145
9.220000	1526.669922	9.219245	0.000000	1522.358887
9.240000	1523.500000	9.239244	0.000000	1518.515625
9.260000	1527.669922	9.259245	0.000000	1514.692871
9.280000	1515.669922	9.279244	0.000000	1510.890869
9.300000	1528.669922	9.299245	0.000000	1507.108643
9.320001	1493.169922	9.319246	0.000000	1503.345703
9.340000	1514.830078	9.339246	0.000000	1499.603760
9.360001	1533.169922	9.359246	0.000000	1495.880859

9.380000	1518.669922	9.379246	0.000000	1492.177246
9.400000	1477.830078	9.399245	0.000000	1488.492920
9.420000	1492.169922	9.419246	0.000000	1484.827393
9.440000	1502.669922	9.439245	0.000000	1481.181641
9.460000	1496.500000	9.459246	0.000000	1477.553711
9.480000	1489.169922	9.479245	1.095409	1473.944092
9.500000	1479.830078	9.499246	1.119062	1470.352783
9.520000	1464.830078	9.519246	1.143593	1466.779541
9.540000	1450.330078	9.539246	1.169047	1463.224609
9.560000	1482.500000	9.559245	1.195473	1459.687744
9.580000	1472.830078	9.579245	1.222926	1456.167969
9.600000	1454.330078	9.599245	1.251464	1452.665527
9.620001	1474.500000	9.619246	1.281147	1449.180176
9.640000	1470.330078	9.639245	1.312040	1445.712402
9.660001	1445.500000	9.659245	1.344215	1442.260986
9.680000	1433.000000	9.679245	1.377746	1438.827393
9.700000	1425.669922	9.699244	1.412717	1435.409424
9.720000	1432.669922	9.719245	1.449217	1432.007812
9.740000	1461.000000	9.739244	1.487341	1428.622803
9.760000	1425.169922	9.759245	1.527193	1425.253906
9.780000	1433.000000	9.779244	1.568884	1421.901367
9.800000	1437.669922	9.799245	1.612540	1418.564209
9.820001	1424.669922	9.819245	1.658289	1415.242432
9.840000	1431.169922	9.839245	1.706277	1411.936768
9.860001	1407.000000	9.859245	1.756663	1408.646240
9.880000	1416.000000	9.879245	1.809615	1405.370605
9.900000	1417.500000	9.899244	1.865325	1402.110840
9.920000	1426.169922	9.919245	1.924001	1398.865479
9.940000	1381.830078	9.939244	1.985866	1395.635010
9.960000	1401.669922	9.959244	2.051171	1392.418945
9.980000	1391.330078	9.979243	2.120192	1389.218018
10.000000	1388.000000	9.999244	2.193239	1386.031006
10.020000	1382.830078	10.019244	2.270646	1382.858398
10.040000	1378.169922	10.039244	2.352785	1379.700195
10.060000	1378.330078	10.059244	2.440081	1376.554932
10.080000	1386.000000	10.079244	2.532995	1373.424561
10.100000	1381.669922	10.099244	2.632058	1370.307373
10.120001	1395.330078	10.119245	2.737854	1367.204346

10.140000	1367.330078	10.139244	2.851042	1364.114746
10.160001	1376.000000	10.159245	2.972381	1361.038330
10.180000	1380.500000	10.179244	3.102710	1357.975098
10.200000	1373.000000	10.199244	3.243003	1354.925537
10.220000	1359.330078	10.219244	3.394376	1351.888672
10.240000	1346.169922	10.239244	3.558084	1348.864258
10.260000	1342.330078	10.259244	3.735610	1345.852539
10.280000	1337.330078	10.279243	3.928635	1342.853760
10.300000	1340.500000	10.299245	4.139166	1339.867920
10.320001	1336.830078	10.319245	4.369489	1336.893799
10.340000	1330.669922	10.339244	4.622316	1333.932861
10.360001	1307.169922	10.359244	4.900927	1330.983398
10.380000	1312.669922	10.379244	5.209135	1328.046387
10.400000	1347.330078	10.399243	5.551583	1325.121094
10.420000	1353.000000	10.419244	5.933899	1322.208008
10.440000	1309.830078	10.439243	6.362895	1319.306641
10.460000	1316.000000	10.459244	6.847057	1316.416992
10.480000	1314.830078	10.479243	7.396860	1313.538574
10.500000	1302.330078	10.499244	8.025618	1310.671631
10.520000	1326.500000	10.519244	8.750224	1307.816162
10.540000	1326.669922	10.539244	9.592531	1304.972168
10.560000	1325.330078	10.559244	10.581411	1302.139404
10.580000	1310.169922	10.579244	11.755296	1299.317627
10.600000	1317.330078	10.599244	13.166969	1296.506836
10.620001	1311.169922	10.619245	14.890549	1293.706787
10.640000	1303.330078	10.639244	17.037554	1290.917480
10.660001	1323.830078	10.659245	19.809929	1288.138916
10.680000	1290.000000	10.679244	23.704338	1285.371338
10.700000	1330.000000	10.699244	30.177055	1282.614502
10.720000	1325.000000	10.719244	43.043522	1279.867432
10.740000	1370.169922	10.739243	69.534599	1277.130859
10.760000	1385.000000	10.759243	117.424042	1274.404785
10.780000	1420.669922	10.779243	187.175110	1271.688965
10.800000	1504.000000	10.799243	271.660431	1268.982910
10.820001	1591.000000	10.819243	375.043274	1266.287109
10.840000	1630.500000	10.839243	422.071198	1263.600830
10.860001	1507.000000	10.859243	175.874496	1260.924805
10.880000	1410.830078	10.879243	17.883516	1258.258545

10.900000	1306.330078	10.899242	-17.866188	1255.601807
10.920000	1276.830078	10.919243	-5.083505	1252.954834
10.940000	1250.669922	10.939242	14.694562	1250.317383
10.960000	1242.500000	10.959243	25.026684	1247.689209
10.980000	1236.330078	10.979242	26.380169	1245.070312
11.000000	1232.169922	10.999243	23.813322	1242.460938
11.020000	1243.330078	11.019243	20.577412	1239.860596
11.040000	1264.500000	11.039243	17.756056	1237.270020
11.060000	1252.500000	11.059243	15.478331	1234.687988
11.080000	1263.000000	11.079243	13.645289	1232.114990
11.100000	1239.000000	11.099243	12.149617	1229.551270
11.120001	1232.669922	11.119244	10.910923	1226.995850
11.140000	1246.669922	11.139242	9.871384	1224.450195
11.160001	1230.000000	11.159243	8.988608	1221.912354
11.180000	1221.000000	11.179242	8.231392	1219.383301
11.200000	1206.330078	11.199243	7.575939	1216.863037
11.220000	1212.330078	11.219243	7.004041	1214.351318
11.240000	1190.830078	11.239243	6.501479	1211.848145
11.260000	1195.330078	11.259243	6.056940	1209.353027
11.280000	1213.669922	11.279243	5.661460	1206.867432
11.300000	1184.669922	11.299243	5.307716	1204.389160
11.320001	1172.330078	11.319243	4.989779	1201.918945
11.340000	1231.000000	11.339243	4.702765	1199.457275
11.360001	1217.169922	11.359243	4.442576	1197.003418
11.380000	1218.000000	11.379243	4.205835	1194.558350
11.400000	1186.830078	11.399242	3.989664	1192.120361
11.420000	1203.000000	11.419243	3.791622	1189.690674
11.440000	1183.500000	11.439242	3.609656	1187.268799
11.460000	1179.669922	11.459243	3.441973	1184.854980
11.480000	1198.830078	11.479242	3.287059	1182.448486
11.500000	1153.669922	11.499243	3.143571	1180.050049
11.520000	1168.000000	11.519243	3.010363	1177.659180
11.540000	1175.830078	11.539242	2.886440	1175.276123
11.560000	1150.500000	11.559242	2.770894	1172.900635
11.580000	1140.830078	11.579242	2.662964	1170.532471
11.600000	1170.500000	11.599242	2.561951	1168.171631
11.620001	1159.330078	11.619243	2.467253	1165.818359
11.640000	1154.830078	11.639242	2.378332	1163.472168

11.660001	1157.830078	11.659243	2.294694	1161.133545
11.680000	1164.830078	11.679242	2.215916	1158.802002
11.700000	1136.500000	11.699242	2.141606	1156.477539
11.720000	1162.330078	11.719242	2.071411	1154.160645
11.740000	1166.669922	11.739242	2.005024	1151.850830
11.760000	1150.500000	11.759242	1.942155	1149.547852
11.780000	1132.830078	11.779242	1.882553	1147.251709
11.800000	1142.669922	11.799242	1.825976	1144.962891
11.820001	1146.169922	11.819242	1.772217	1142.681152
11.840000	1129.830078	11.839242	1.721084	1140.405762
11.860001	1157.500000	11.859242	1.672395	1138.137451
11.880000	1126.000000	11.879242	1.625994	1135.876221
11.900000	1146.330078	11.899241	1.581729	1133.621338
11.920000	1159.330078	11.919242	1.539462	1131.373291
11.940000	1136.000000	11.939240	1.499075	1129.132080
11.960000	1144.330078	11.959241	1.460445	1126.897461
11.980000	1133.500000	11.979240	1.423470	1124.668945
12.000000	1120.169922	11.999241	1.388050	1122.447266
12.020000	1133.330078	12.019241	1.354096	1120.232422
12.040000	1131.000000	12.039242	1.321523	1118.023438
12.060000	1135.330078	12.059242	1.290254	1115.821533
12.080000	1125.500000	12.079242	1.260217	1113.625977
12.100000	1106.169922	12.099242	1.231343	1111.436279
12.120001	1120.000000	12.119243	1.203571	1109.253174
12.140000	1097.500000	12.139242	1.176844	1107.076416
12.160001	1125.000000	12.159243	1.151103	1104.905762
12.180000	1083.669922	12.179242	1.126303	1102.741455
12.200000	1068.169922	12.199242	1.102392	1100.583496
12.220000	1087.330078	12.219242	0.000000	1098.431641
12.240000	1081.500000	12.239242	0.000000	1096.285400
12.260000	1065.169922	12.259242	0.000000	1094.145508
12.280000	1081.330078	12.279242	0.000000	1092.011719
12.300000	1083.169922	12.299242	0.000000	1089.883789
12.320001	1102.669922	12.319242	0.000000	1087.762207
12.340000	1062.000000	12.339241	0.000000	1085.646484
12.360001	1076.169922	12.359241	0.000000	1083.536377
12.380000	1075.169922	12.379241	0.000000	1081.432373
12.400000	1062.330078	12.399240	0.000000	1079.334473

12.420000	1075.830078	12.419241	0.000000	1077.241943
12.440000	1072.830078	12.439240	0.000000	1075.155273
12.460000	1053.669922	12.459241	0.000000	1073.074463
12.480000	1075.000000	12.479240	0.000000	1070.999268
12.500000	1075.500000	12.499241	0.000000	1068.929932
12.520000	1065.669922	12.519241	0.000000	1066.866211
12.540000	1051.000000	12.539241	0.000000	1064.808350
12.560000	1035.669922	12.559241	0.000000	1062.755859
12.580000	1051.330078	12.579241	0.000000	1060.708984
12.600000	1047.169922	12.599241	0.000000	1058.667725
12.620001	1063.169922	12.619242	0.000000	1056.632080
12.640000	1054.000000	12.639241	0.000000	1054.601807
12.660001	1032.830078	12.659242	0.000000	1052.577026
12.680000	1051.500000	12.679241	0.000000	1050.557861
12.700000	1047.330078	12.699241	0.000000	1048.544312
12.720000	1051.500000	12.719240	0.000000	1046.535645
12.740000	1073.169922	12.739240	0.000000	1044.533203
12.760000	1031.500000	12.759240	0.000000	1042.535645
12.780000	1021.330017	12.779240	0.000000	1040.542969
12.800000	1022.169983	12.799240	0.000000	1038.556152
12.820001	1017.830017	12.819242	0.000000	1036.574219
12.840000	1023.830017	12.839241	0.000000	1034.598022
12.860001	1037.330078	12.859241	0.000000	1032.626709
12.880000	1033.169922	12.879241	0.000000	1030.660889
12.900000	1014.669983	12.899240	0.000000	1028.700439
12.920000	1036.830078	12.919241	0.000000	1026.744751
12.940000	1021.000000	12.939240	0.000000	1024.794312
12.960000	1016.330017	12.959241	0.000000	1022.849121
12.980000	997.333008	12.979240	0.000000	1020.909546
13.000000	1016.169983	12.999241	0.000000	1018.974487
13.020000	1018.330017	13.019241	0.000000	1017.044434
13.040000	1020.500000	13.039241	0.000000	1015.119263
13.060000	1014.330017	13.059241	0.000000	1013.199829
13.080000	1014.500000	13.079241	0.000000	1011.284546
13.100000	1008.330017	13.099241	0.000000	1009.374878
13.120001	1004.669983	13.119241	0.000000	1007.469849
13.140000	1013.830017	13.139240	0.000000	1005.570190
13.160001	1007.330017	13.159241	0.000000	1003.675415

13.180000	1010.000000	13.179240	0.000000	1001.785400
13.200000	1002.830017	13.199240	0.000000	999.900513
13.220000	1006.669983	13.219240	0.000000	998.020142
13.240000	984.000000	13.239240	0.000000	996.144897
13.260000	998.333008	13.259240	0.000000	994.274414
13.280000	1000.830017	13.279240	0.000000	992.408569
13.300000	997.333008	13.299240	0.000000	990.547852
13.320001	984.000000	13.319241	0.000000	988.691650
13.340000	992.000000	13.339240	0.000000	986.840576
13.360001	982.833008	13.359241	0.000000	984.994141
13.380000	996.666992	13.379240	0.000000	983.152222
13.400000	984.000000	13.399240	0.000000	981.315186
13.420000	990.833008	13.419240	0.000000	979.482788
13.440000	990.000000	13.439240	0.000000	977.655396
13.460000	963.833008	13.459240	0.000000	975.832275
13.480000	952.000000	13.479239	0.000000	974.014160
13.500000	983.000000	13.499240	0.000000	972.200562
13.520000	966.666992	13.519239	0.000000	970.391357
13.540000	943.500000	13.539239	0.000000	968.587402
13.560000	960.000000	13.559240	0.000000	966.787109
13.580000	969.500000	13.579240	0.000000	964.992310
13.600000	985.000000	13.599240	0.000000	963.201782
13.620001	991.666992	13.619241	0.000000	961.415771
13.640000	955.500000	13.639240	0.000000	959.634155
13.660001	955.000000	13.659241	0.000000	957.857178
13.680000	966.666992	13.679240	0.000000	956.084595
13.700000	944.000000	13.699240	0.000000	954.317017
13.720000	944.500000	13.719240	0.000000	952.553589
13.740000	956.000000	13.739240	0.000000	950.794556
13.760000	952.833008	13.759240	0.000000	949.040039
13.780000	931.166992	13.779240	0.000000	947.290161
13.800000	935.833008	13.799240	0.000000	945.544434
13.820001	928.333008	13.819241	0.000000	943.803101
13.840000	933.000000	13.839240	0.000000	942.066772
13.860001	950.666992	13.859241	0.000000	940.334473
13.880000	938.500000	13.879240	0.000000	938.606445
13.900000	908.833008	13.899240	0.000000	936.882690
13.920000	930.666992	13.919239	0.000000	935.163696

13.940000	932.500000	13.939239	0.000000	933.448975
13.960000	934.833008	13.959239	0.000000	931.738525
13.980000	925.666992	13.979239	0.000000	930.031982
14.000000	936.500000	13.999239	0.000000	928.330444
14.020000	918.500000	14.019239	0.000000	926.632568
14.040000	937.500000	14.039239	0.000000	924.939575
14.060000	904.166992	14.059239	0.000000	923.250732
14.080000	933.333008	14.079239	0.000000	921.565918
14.100000	921.833008	14.099239	0.000000	919.885376
14.120001	935.333008	14.119240	0.000000	918.209229
14.140000	913.500000	14.139239	0.000000	916.537476
14.160001	896.666992	14.159240	0.000000	914.869629
14.180000	914.833008	14.179239	0.000000	913.205933
14.200000	905.000000	14.199239	0.000000	911.546753
14.220000	882.166992	14.219239	0.000000	909.891479
14.240000	911.500000	14.239239	0.000000	908.240356
14.260000	905.166992	14.259240	0.000000	906.593628
14.280000	892.666992	14.279240	0.000000	904.950928
14.300000	919.500000	14.299240	0.000000	903.312378
14.320001	895.666992	14.319240	0.000000	901.678345
14.340000	892.833008	14.339239	0.000000	900.048096
14.360001	893.666992	14.359240	0.000000	898.421875
14.380000	891.666992	14.379239	0.000000	896.800171
14.400000	901.666992	14.399239	0.000000	895.182251
14.420000	895.500000	14.419239	0.000000	893.568481
14.440000	892.833008	14.439239	0.000000	891.958740
14.460000	903.500000	14.459239	0.000000	890.352905
14.480000	864.000000	14.479239	0.000000	888.751343
14.500000	887.833008	14.499239	0.000000	887.153931
14.520000	874.166992	14.519239	0.000000	885.560425
14.540000	882.166992	14.539239	0.000000	883.970825
14.560000	889.833008	14.559239	0.000000	882.385498
14.580000	868.500000	14.579239	0.000000	880.804199
14.600000	887.666992	14.599239	0.000000	879.226440
14.620001	869.500000	14.619240	0.000000	877.653076
14.640000	894.000000	14.639239	0.000000	876.083740
14.660001	880.666992	14.659240	0.000000	874.518311
14.680000	888.833008	14.679239	0.000000	872.956787

14.700000	868.166992	14.699239	0.000000	871.399170
14.720000	864.000000	14.719238	0.000000	869.845825
14.740000	862.166992	14.739238	0.000000	868.295898
14.760000	860.166992	14.759238	0.000000	866.750366
14.780000	875.333008	14.779238	0.000000	865.208374
14.800000	871.833008	14.799238	0.000000	863.670654
14.820001	849.000000	14.819239	0.000000	862.136353
14.840000	860.000000	14.839238	0.000000	860.606567
14.860001	844.500000	14.859239	0.000000	859.080322
14.880000	866.000000	14.879238	0.000000	857.557861
14.900000	854.000000	14.899238	0.000000	856.039429
14.920000	857.166992	14.919239	0.000000	854.524658
14.940000	875.166992	14.939239	0.000000	853.014160
14.960000	847.666992	14.959239	0.000000	851.506958
14.980000	858.166992	14.979239	0.000000	850.004150
15.000000	849.333008	14.999239	0.000000	848.505005
15.020000	845.833008	15.019239	0.000000	847.009399
15.040000	847.000000	15.039239	0.000000	845.517700
15.060000	827.833008	15.059239	0.000000	844.029907
15.080000	840.666992	15.079239	0.000000	842.546143
15.100000	840.833008	15.099239	0.000000	841.065796
15.120001	836.333008	15.119239	0.000000	839.589355
15.140000	837.166992	15.139238	0.000000	838.116577
15.160001	837.833008	15.159239	0.000000	836.647583
15.180000	850.000000	15.179238	0.000000	835.182373
15.200000	853.666992	15.199238	0.000000	833.721191
15.220000	831.833008	15.219238	0.000000	832.263428
15.240000	803.666992	15.239238	0.000000	830.809448
15.260000	822.333008	15.259238	0.000000	829.359131
15.280000	821.166992	15.279238	0.000000	827.912598
15.300000	817.000000	15.299238	0.000000	826.469849
15.320001	840.000000	15.319239	0.000000	825.030762
15.340000	840.666992	15.339238	0.000000	823.595093
15.360001	826.666992	15.359239	0.000000	822.163330
15.380000	821.666992	15.379238	0.000000	820.735107
15.400000	824.833008	15.399238	0.000000	819.310791
15.420000	805.833008	15.419238	0.000000	817.889893
15.440000	811.833008	15.439238	0.000000	816.473022

15.460000	809.500000	15.459238	0.000000	815.059448
15.480000	812.666992	15.479238	0.000000	813.649536
15.500000	814.000000	15.499238	0.000000	812.243408
15.520000	805.333008	15.519238	0.316906	810.840820
15.540000	802.666992	15.539237	0.322053	809.441772
15.560000	803.000000	15.559238	0.327365	808.046387
15.580000	813.833008	15.579238	0.332850	806.654541
15.600000	809.000000	15.599238	0.338516	805.266113
15.620001	803.000000	15.619239	0.344373	803.881470
15.640000	789.500000	15.639238	0.350430	802.500366
15.660001	786.166992	15.659239	0.356699	801.122925
15.680000	802.000000	15.679238	0.363188	799.748779
15.700000	797.333008	15.699238	0.369912	798.378662
15.720000	791.833008	15.719238	0.376882	797.011597
15.740000	799.666992	15.739238	0.384112	795.648438
15.760000	799.333008	15.759238	0.391616	794.288452
15.780000	781.000000	15.779238	0.399411	792.932373
15.800000	783.500000	15.799238	0.407514	791.579590
15.820001	793.833008	15.819239	0.415943	790.230225
15.840000	792.833008	15.839238	0.424717	788.884399
15.860001	776.500000	15.859239	0.433859	787.541992
15.880000	780.166992	15.879238	0.443391	786.203125
15.900000	784.500000	15.899238	0.453339	784.867798
15.920000	787.500000	15.919238	0.463730	783.535767
15.940000	789.833008	15.939237	0.474594	782.207397
15.960000	786.500000	15.959237	0.485966	780.882568
15.980000	763.833008	15.979237	0.497879	779.561035
16.000000	778.833008	15.999238	0.510374	778.242676
16.020000	793.333008	16.019239	0.523494	776.927856
16.040001	778.666992	16.039240	0.537285	775.616577
16.059999	775.166992	16.059238	0.551800	774.308838
16.080000	766.666992	16.079239	0.567098	773.004272
16.100000	755.166992	16.099239	0.583244	771.703247
16.120001	780.500000	16.119240	0.600308	770.405518
16.140001	766.166992	16.139238	0.618368	769.111084
16.160000	761.166992	16.159237	0.637515	767.820312
16.180000	762.666992	16.179237	0.657853	766.532593
16.200001	758.500000	16.199238	0.679490	765.248291

16.220001	773.500000	16.219238	0.702556	763.967529
16.240002	757.000000	16.239239	0.727194	762.689819
16.260000	774.500000	16.259237	0.753567	761.415894
16.280001	763.166992	16.279238	0.781867	760.144897
16.299999	764.000000	16.299236	0.812307	758.877197
16.320000	758.333008	16.319237	0.845142	757.612915
16.340000	774.000000	16.339237	0.880659	756.351929
16.359999	733.666992	16.359236	0.919192	755.094360
16.379999	755.000000	16.379236	0.961148	753.839966
16.400000	748.000000	16.399237	1.006993	752.588745
16.420000	721.333008	16.419237	1.057288	751.340942
16.440001	752.333008	16.439238	1.112705	750.096313
16.459999	742.833008	16.459238	1.174059	748.854858
16.480000	735.500000	16.479239	1.242345	747.616699
16.500000	758.500000	16.499239	1.318793	746.381836
16.520000	729.333008	16.519239	1.404940	745.150024
16.540001	748.833008	16.539240	1.502729	743.921631
16.559999	751.500000	16.559238	1.614647	742.696655
16.580000	763.333008	16.579239	1.743975	741.474609
16.600000	750.166992	16.599239	1.895062	740.255737
16.620001	744.833008	16.619240	2.074003	739.040161
16.640001	738.500000	16.639240	2.290462	737.827759
16.660000	733.166992	16.659239	2.564098	736.618408
16.680000	747.833008	16.679239	2.945213	735.412354
16.700001	740.500000	16.699240	3.561187	734.209351
16.720001	734.000000	16.719240	4.669902	733.009888
16.740002	740.500000	16.739241	6.614280	731.813110
16.760000	747.666992	16.759239	9.561362	730.619629
16.780001	759.500000	16.779240	13.247187	729.429199
16.799999	789.666992	16.799238	17.423792	728.242065
16.820000	856.666992	16.819239	23.266756	727.057983
16.840000	836.166992	16.839239	29.299152	725.876953
16.859999	800.833008	16.859238	16.066441	724.698975
16.879999	756.500000	16.879238	5.830048	723.524048
16.900000	738.166992	16.899239	2.227859	722.352295
16.920000	737.166992	16.919239	1.754806	721.183716
16.940001	739.000000	16.939238	2.389457	720.018188
16.959999	742.000000	16.959236	2.942656	718.855591

16.980000	742.333008	16.979237	3.068961	717.696167
17.000000	740.666992	16.999237	2.899726	716.539673
17.020000	723.500000	17.019238	2.631519	715.386230
17.040001	715.166992	17.039238	2.369456	714.235718
17.059999	708.000000	17.059237	2.143641	713.088257
17.080000	710.333008	17.079237	1.954044	711.943848
17.100000	706.500000	17.099237	1.794143	710.802490
17.120001	712.000000	17.119238	1.657771	709.664185
17.140001	718.166992	17.139238	1.540168	708.528687
17.160000	700.500000	17.159237	1.437754	707.396240
17.180000	695.000000	17.179237	1.347775	706.266724
17.200001	698.500000	17.199238	1.268123	705.140137
17.220001	699.333008	17.219238	1.197132	704.016479
17.240002	718.666992	17.239239	1.133474	702.895996
17.260000	682.833008	17.259237	1.076083	701.778320
17.280001	693.000000	17.279238	1.024077	700.663574
17.299999	684.000000	17.299236	0.976746	699.551636
17.320000	709.166992	17.319237	0.933485	698.442993
17.340000	689.833008	17.339237	0.893798	697.336792
17.359999	679.500000	17.359236	0.857268	696.233643
17.379999	685.833008	17.379236	0.823529	695.133423
17.400000	712.166992	17.399237	0.792278	694.035889
17.420000	691.333008	17.419237	0.763253	692.941406
17.440001	705.000000	17.439238	0.736226	691.849976
17.459999	696.833008	17.459236	0.711001	690.761108
17.480000	687.166992	17.479237	0.687402	689.675049
17.500000	698.833008	17.499237	0.665279	688.591919
17.520000	688.333008	17.519238	0.920993	687.511719
17.540001	676.500000	17.539238	0.907108	686.434082
17.559999	674.000000	17.559237	0.894554	685.359619
17.580000	694.666992	17.579237	0.883244	684.287720
17.600000	687.833008	17.599239	0.873109	683.218506
17.620001	669.166992	17.619240	0.864088	682.152222
17.640001	669.666992	17.639240	0.856127	681.088745
17.660000	686.833008	17.659239	0.849182	680.028076
17.680000	698.000000	17.679239	0.843211	678.970093
17.700001	683.500000	17.699240	0.838184	677.914795
17.720001	672.166992	17.719240	0.834074	676.862427

17.740002	653.833008	17.739241	0.830860	675.812622
17.760000	676.833008	17.759237	0.828525	674.765625
17.780001	682.666992	17.779238	0.827059	673.721436
17.799999	663.000000	17.799236	0.826455	672.680054
17.820000	684.333008	17.819237	0.826713	671.641113
17.840000	675.000000	17.839237	0.827834	670.604980
17.859999	665.666992	17.859236	0.829827	669.571533
17.879999	672.166992	17.879236	0.832704	668.540771
17.900000	655.000000	17.899237	0.836483	667.512573
17.920000	652.833008	17.919237	0.841186	666.487061
17.940001	657.500000	17.939238	0.846842	665.464233
17.959999	672.166992	17.959236	0.853482	664.443970
17.980000	656.166992	17.979237	0.861148	663.426392
18.000000	669.500000	17.999237	0.869887	662.411499
18.020000	652.833008	18.019238	0.879752	661.399048
18.040001	661.666992	18.039238	0.890805	660.389282
18.059999	669.166992	18.059237	0.903117	659.382568
18.080000	673.000000	18.079237	0.916772	658.377930
18.100000	648.333008	18.099237	0.931860	657.375977
18.120001	646.500000	18.119238	0.948489	656.376587
18.140001	668.000000	18.139238	0.966780	655.379883
18.160000	666.666992	18.159237	0.986868	654.385864
18.180000	639.500000	18.179237	0.695269	653.394043
18.200001	655.000000	18.199238	0.724347	652.404907
18.220001	642.333008	18.219238	0.755625	651.418457
18.240002	654.500000	18.239239	0.789350	650.434326
18.260000	640.500000	18.259237	0.825802	649.452759
18.280001	654.666992	18.279238	0.865314	648.473511
18.299999	658.333008	18.299236	0.908258	647.497314
18.320000	635.166992	18.319237	0.955087	646.523193
18.340000	638.500000	18.339237	1.006317	645.551636
18.359999	648.500000	18.359236	1.062558	644.582764
18.379999	635.333008	18.379236	1.124554	643.616089
18.400000	638.000000	18.399237	1.193177	642.651855
18.420000	648.833008	18.419237	1.269488	641.690063
18.440001	645.166992	18.439238	1.354777	640.730835
18.459999	646.166992	18.459236	1.450624	639.774048
18.480000	649.666992	18.479237	1.559021	638.819580

18.500000	640.166992	18.499237	1.682438	637.867554
18.520000	651.500000	18.519238	1.824032	636.917969
18.540001	643.833008	18.539238	1.987879	635.970703
18.559999	645.833008	18.559237	2.179304	635.026001
18.580000	644.333008	18.579235	2.405467	634.083740
18.600000	627.500000	18.599236	2.676222	633.143555
18.620001	627.500000	18.619236	3.005887	632.206055
18.640001	620.666992	18.639236	3.418525	631.270630
18.660000	635.666992	18.659235	3.964564	630.337646
18.680000	642.333008	18.679237	4.766850	629.406860
18.700001	636.000000	18.699238	6.106635	628.478638
18.720001	613.833008	18.719238	8.494609	627.552612
18.740002	656.833008	18.739239	12.550455	626.628906
18.760000	644.166992	18.759237	18.615902	625.707520
18.780001	647.000000	18.779238	26.648094	624.788452
18.799999	663.166992	18.799236	37.451691	623.871704
18.820000	686.666992	18.819237	53.537949	622.957153
18.840000	691.166992	18.839237	55.061466	622.044800
18.859999	678.666992	18.859236	25.846552	621.135010
18.879999	661.000000	18.879236	9.863112	620.227295
18.900000	631.500000	18.899237	4.684821	619.321655
18.920000	625.166992	18.919237	4.065309	618.418457
18.940001	631.333008	18.939238	4.648470	617.517456
18.959999	629.833008	18.959236	4.944180	616.618530
18.980000	625.333008	18.979237	4.723165	615.721924
19.000000	618.833008	18.999237	4.237518	614.827515
19.020000	628.333008	19.019238	3.719178	613.935303
19.040001	617.166992	19.039238	3.265189	613.045166
19.059999	619.666992	19.059237	2.890364	612.157593
19.080000	620.000000	19.079237	2.582797	611.271729
19.100000	610.666992	19.099237	2.327888	610.388306
19.120001	608.333008	19.119238	2.113888	609.506958
19.140001	605.000000	19.139238	1.932067	608.627686
19.160000	598.666992	19.159237	1.775958	607.750732
19.180000	594.333008	19.179237	1.640646	606.875610
19.200001	597.666992	19.199238	1.522403	606.002686
19.220001	614.333008	19.219238	1.418312	605.132080
19.240002	609.000000	19.239239	1.326074	604.263306

19.260000	609.000000	19.259237	1.243858	603.396851
19.280001	616.166992	19.279238	1.170167	602.532471
19.299999	591.500000	19.299236	1.103802	601.670166
19.320000	597.500000	19.319237	1.043756	600.809692
19.340000	606.166992	19.339237	0.989208	599.951538
19.359999	587.833008	19.359236	0.939470	599.095337
19.379999	595.666992	19.379236	0.893951	598.241211
19.400000	593.166992	19.399235	0.852165	597.389038
19.420000	599.666992	19.419235	0.813681	596.538818
19.440001	600.333008	19.439236	0.778144	595.690674
19.459999	608.166992	19.459234	0.745243	594.844727
19.480000	583.333008	19.479235	0.714702	594.000610
19.500000	605.333008	19.499235	0.686290	593.158569
19.520000	616.333008	19.519236	0.659799	592.318237
19.540001	597.166992	19.539236	0.635051	591.480225
19.559999	599.000000	19.559235	0.611887	590.644165
19.580000	605.833008	19.579235	0.590163	589.809814
19.600000	590.000000	19.599236	0.569756	588.977539
19.620001	601.500000	19.619236	0.550554	588.147339
19.640001	580.500000	19.639236	0.532459	587.318848
19.660000	584.833008	19.659235	0.515383	586.492188
19.680000	591.833008	19.679237	0.499241	585.667725
19.700001	591.500000	19.699238	0.483967	584.845093
19.720001	570.666992	19.719238	0.469494	584.024292
19.740002	583.833008	19.739239	0.455763	583.205322
19.760000	570.666992	19.759237	0.442721	582.388550
19.780001	589.500000	19.779238	0.430320	581.573486
19.799999	579.333008	19.799236	0.418516	580.760376
19.820000	571.833008	19.819237	0.407267	579.948853
19.840000	559.500000	19.839237	0.396537	579.139282
19.859999	571.000000	19.859236	0.386295	578.331665
19.879999	573.833008	19.879236	0.376507	577.525757
19.900000	545.166992	19.899237	0.367145	576.721680
19.920000	580.500000	19.919237	0.358184	575.919556
19.940001	568.166992	19.939238	0.349600	575.119141
19.959999	568.000000	19.959236	0.341371	574.320557
19.980000	568.333008	19.979237	0.333475	573.523804
20.000000	575.000000	19.999237	0.325893	572.728760

20.020000	562.333008	20.019238	0.318609	571.935425
20.040001	584.000000	20.039238	0.311606	571.143921
20.059999	575.166992	20.059237	0.304868	570.354370
20.080000	572.000000	20.079237	0.298382	569.566406
20.100000	577.500000	20.099237	0.292134	568.780151
20.120001	566.333008	20.119238	0.286111	567.995605
20.140001	557.500000	20.139238	0.280303	567.212891
20.160000	568.166992	20.159237	0.000000	566.431885
20.180000	569.833008	20.179237	0.000000	565.652588
20.200001	561.666992	20.199238	0.000000	564.875000
20.220001	580.833008	20.219236	0.000000	564.099243
20.240002	564.000000	20.239237	0.000000	563.325073
20.260000	575.833008	20.259235	0.000000	562.552490
20.280001	579.000000	20.279236	0.000000	561.781616
20.299999	559.833008	20.299234	0.000000	561.012451
20.320000	581.333008	20.319235	0.000000	560.244873
20.340000	566.500000	20.339235	0.000000	559.479004
20.359999	573.500000	20.359234	0.276431	558.714844
20.379999	549.166992	20.379234	0.283429	557.952148
20.400000	557.333008	20.399235	0.290707	557.191162
20.420000	554.666992	20.419235	0.298283	556.431885
20.440001	558.833008	20.439236	0.306172	555.674194
20.459999	565.833008	20.459234	0.314393	554.918091
20.480000	560.500000	20.479235	0.322964	554.163574
20.500000	555.000000	20.499235	0.331908	553.410522
20.520000	552.500000	20.519236	0.341245	552.659180
20.540001	556.833008	20.539236	0.351000	551.909424
20.559999	548.333008	20.559235	0.361197	551.161011
20.580000	551.500000	20.579235	0.371867	550.414307
20.600000	552.000000	20.599236	0.383039	549.669189
20.620001	557.833008	20.619236	0.394745	548.925537
20.640001	554.500000	20.639238	0.407022	548.183594
20.660000	529.500000	20.659237	0.419906	547.443115
20.680000	552.000000	20.679237	0.433440	546.704102
20.700001	567.500000	20.699238	0.447670	545.966431
20.720001	540.500000	20.719238	0.462644	545.230591
20.740002	541.166992	20.739239	0.478418	544.496094
20.760000	552.333008	20.759237	0.495049	543.763184

20.780001	538.333008	20.779238	0.512604	543.031616
20.799999	546.833008	20.799236	0.531151	542.301758
20.820000	540.500000	20.819237	0.550773	541.573120
20.840000	559.166992	20.839237	0.571554	540.846069
20.859999	537.500000	20.859236	0.593586	540.120361
20.879999	535.000000	20.879236	0.616979	539.396240
20.900000	561.500000	20.899237	0.641847	538.673462
20.920000	525.666992	20.919237	0.668321	537.952148
20.940001	520.833008	20.939238	0.696544	537.232178
20.959999	539.500000	20.959236	0.726676	536.513672
20.980000	533.333008	20.979237	0.758901	535.796631
21.000000	547.166992	20.999237	0.793419	535.081055
21.020000	548.166992	21.019238	0.830459	534.366821
21.040001	544.000000	21.039236	0.870274	533.653931
21.059999	556.333008	21.059235	0.913159	532.942383
21.080000	529.833008	21.079235	0.959449	532.232300
21.100000	538.833008	21.099236	1.009519	531.523438
21.120001	535.500000	21.119236	1.063801	530.816040
21.140001	536.333008	21.139236	1.122792	530.110107
21.160000	537.333008	21.159235	1.187059	529.405396
21.180000	540.166992	21.179235	1.257279	528.702026
21.200001	524.333008	21.199236	1.334220	528.000000
21.220001	539.333008	21.219236	1.418789	527.299316
21.240002	525.833008	21.239237	1.512052	526.599976
21.260000	549.833008	21.259235	1.615260	525.901978
21.280001	510.000000	21.279236	1.729936	525.205078
21.299999	513.166992	21.299234	1.857858	524.509766
21.320000	518.000000	21.319235	2.001225	523.815552
21.340000	517.166992	21.339235	2.162671	523.122559
21.359999	522.333008	21.359234	2.345422	522.431152
21.379999	524.000000	21.379234	2.553529	521.740723
21.400000	523.000000	21.399235	2.791998	521.051758
21.420000	513.333008	21.419235	3.067177	520.363892
21.440001	516.333008	21.439236	3.535454	519.677246
21.459999	512.666992	21.459234	3.914871	518.992065
21.480000	533.000000	21.479235	4.363702	518.307983
21.500000	522.666992	21.499235	4.900452	517.625244
21.520000	521.666992	21.519236	5.550390	516.943604

21.540001	517.500000	21.539236	6.348851	516.263184
21.559999	523.166992	21.559237	7.347363	515.583984
21.580000	509.000000	21.579237	8.627996	514.905884
21.600000	515.833008	21.599237	10.343554	514.229126
21.620001	526.833008	21.619238	12.826897	513.553589
21.640001	511.000000	21.639238	16.827999	512.879150
21.660000	517.333008	21.659237	23.846142	512.205933
21.680000	535.500000	21.679237	36.262459	511.533844
21.700001	545.666992	21.699238	56.877060	510.863007
21.720001	573.833008	21.719238	88.490524	510.193329
21.740002	650.500000	21.739239	136.023987	509.524689
21.760000	727.000000	21.759237	208.421890	508.857269
21.780001	745.666992	21.779238	261.855255	508.191132
21.799999	690.666992	21.799236	168.102142	507.525909
21.820000	596.833008	21.819237	70.248901	506.861908
21.840000	555.333008	21.839237	30.639357	506.199005
21.859999	521.000000	21.859236	19.237932	505.537323
21.879999	508.666992	21.879234	17.226526	504.876617
21.900000	505.332977	21.899235	16.576195	504.217133
21.920000	511.332977	21.919235	15.114690	503.558563
21.940001	496.332977	21.939236	13.077237	502.901276
21.959999	513.166992	21.959234	11.059369	502.245148
21.980000	496.500000	21.979235	9.354974	501.589813
22.000000	489.666992	21.999235	8.000731	500.935699
22.020000	512.500000	22.019236	6.936757	500.282684
22.040001	503.500000	22.039236	6.093678	499.630646
22.059999	500.332977	22.059235	5.416803	498.979767
22.080000	504.666992	22.079235	4.866430	498.329865
22.100000	499.332977	22.099236	4.414247	497.680878
22.120001	501.166992	22.119236	4.039622	497.033234
22.140001	512.166992	22.139236	3.727299	496.386566
22.160000	509.000000	22.159235	3.465857	495.740875
22.180000	490.832977	22.179235	3.246551	495.096222
22.200001	503.832977	22.199236	3.062747	494.452484
22.220001	501.666992	22.219236	2.909306	493.809784
22.240002	503.166992	22.239237	2.782264	493.168060
22.260000	503.332977	22.259235	2.678584	492.527435
22.280001	511.000000	22.279236	2.595943	491.887726

22.299999	502.666992	22.299234	2.532665	491.248932
22.320000	504.666992	22.319235	2.487572	490.611298
22.340000	506.332977	22.339235	2.459976	489.974701
22.359999	490.166992	22.359234	2.449619	489.339020
22.379999	476.500000	22.379234	2.456679	488.704254
22.400000	480.166992	22.399235	2.481787	488.070343
22.420000	479.500000	22.419235	2.526070	487.437531
22.440001	485.332977	22.439236	2.591223	486.805573
22.459999	492.832977	22.459236	2.679626	486.174591
22.480000	484.332977	22.479237	2.794503	485.544525
22.500000	489.000000	22.499237	2.940161	484.915436
22.520000	491.832977	22.519238	3.122323	484.287262
22.540001	488.332977	22.539238	3.348612	483.659943
22.559999	485.832977	22.559237	3.629234	483.033661
22.580000	489.666992	22.579237	3.978133	482.408234
22.600000	492.666992	22.599237	4.414447	481.783661
22.620001	490.500000	22.619238	4.965168	481.160126
22.640001	493.000000	22.639238	5.669655	480.537445
22.660000	483.332977	22.659237	6.589325	479.915680
22.680000	500.166992	22.679237	7.833744	479.294830
22.700001	496.000000	22.699238	9.629953	478.674713
22.720001	505.332977	22.719236	12.483744	478.055695
22.740002	504.500000	22.739237	17.447920	477.437347
22.760000	493.332977	22.759235	26.347872	476.819916
22.780001	520.166992	22.779236	41.682980	476.203400
22.799999	527.666992	22.799234	66.350349	475.587799
22.820000	579.000000	22.819235	104.726646	474.973053
22.840000	633.500000	22.839235	164.678543	474.358978
22.859999	704.666992	22.859234	227.646744	473.745941
22.879999	663.833008	22.879234	178.647415	473.133698
22.900000	574.166992	22.899235	81.076813	472.522186
22.920000	519.000000	22.919235	34.297119	471.911530
22.940001	493.666992	22.939236	18.913692	471.301727
22.959999	490.332977	22.959234	15.019707	470.692780
22.980000	489.000000	22.979235	13.769509	470.084503
23.000000	468.500000	22.999235	12.354808	469.477264
23.020000	470.832977	23.019236	10.568759	468.870697
23.040001	467.500000	23.039236	8.807613	468.264984

23.059999	462.500000	23.059235	7.318023	467.660126
23.080000	474.332977	23.079235	6.139285	467.055878
23.100000	472.166992	23.099236	5.220695	466.452606
23.120001	466.166992	23.119236	4.498849	465.850006
23.140001	466.832977	23.139236	3.922790	465.248138
23.160000	472.666992	23.159235	3.455822	464.647186
23.180000	472.166992	23.179235	3.071775	464.046967
23.200001	457.332977	23.199236	2.751952	463.447540
23.220001	457.832977	23.219236	2.482617	462.848724
23.240002	447.666992	23.239237	1.987846	462.250885
23.260000	463.000000	23.259235	1.797592	461.653656
23.280001	456.832977	23.279236	1.633603	461.057159
23.299999	448.332977	23.299236	1.491258	460.461456
23.320000	456.500000	23.319237	1.366895	459.866547
23.340000	452.832977	23.339237	1.257600	459.272308
23.359999	478.166992	23.359236	1.161033	458.678925
23.379999	460.832977	23.379236	1.075269	458.086151
23.400000	453.000000	23.399237	0.998755	457.494110
23.420000	465.332977	23.419237	0.930203	456.902802
23.440001	453.500000	23.439238	0.868541	456.312164
23.459999	460.332977	23.459236	0.812877	455.722382
23.480000	465.500000	23.479237	0.762444	455.133270
23.500000	443.000000	23.499237	0.716610	454.544830
23.520000	470.832977	23.519238	0.674828	453.957123
23.540001	464.000000	23.539238	0.636633	453.370087
23.559999	460.000000	23.559235	0.601630	452.783844
23.580000	444.166992	23.579235	0.569460	452.198090
23.600000	452.500000	23.599236	0.539829	451.613251
23.620001	452.332977	23.619236	0.512476	451.028900
23.640001	451.332977	23.639236	0.487172	450.445282
23.660000	452.500000	23.659235	0.463718	449.862518
23.680000	463.332977	23.679235	0.441933	449.280182
23.700001	446.332977	23.699236	0.421665	448.698700
23.720001	460.666992	23.719236	0.402774	448.117645
23.740002	437.666992	23.739237	0.385138	447.537445
23.760000	451.000000	23.759235	0.368650	446.957916
23.780001	437.666992	23.779236	0.353207	446.378937
23.799999	447.666992	23.799234	0.338727	445.800690

23.820000	450.000000	23.819235	0.325128	445.223175
23.840000	438.000000	23.839235	0.447400	444.646149
23.859999	458.166992	23.859234	0.438557	444.069916
23.879999	446.166992	23.879234	0.430513	443.494110
23.900000	442.166992	23.899235	0.423220	442.919098
23.920000	456.166992	23.919235	0.416638	442.344635
23.940001	444.832977	23.939236	0.410727	441.770844
23.959999	440.832977	23.959234	0.405457	441.197601
23.980000	445.166992	23.979235	0.421193	440.625092
24.000000	455.166992	23.999235	0.417801	440.053131
24.020000	453.666992	24.019236	0.415003	439.481842
24.040001	433.832977	24.039236	0.412781	438.911102
24.059999	444.166992	24.059235	0.411120	438.341217
24.080000	456.166992	24.079235	0.410006	437.771698
24.100000	441.832977	24.099236	0.409432	437.202850
24.120001	452.000000	24.119238	0.409388	436.634308
24.140001	433.166992	24.139238	0.409869	436.066681
24.160000	439.166992	24.159237	0.410874	435.499786
24.180000	440.832977	24.179237	0.412400	434.933258
24.200001	441.332977	24.199238	0.414450	434.367401
24.220001	439.166992	24.219238	0.417028	433.801971
24.240002	439.332977	24.239239	0.420139	433.237274
24.260000	449.500000	24.259237	0.423791	432.673065
24.280001	435.166992	24.279238	0.427995	432.109528
24.299999	438.500000	24.299236	0.432763	431.546661
24.320000	433.000000	24.319237	0.294172	430.984222
24.340000	439.000000	24.339237	0.303871	430.422394
24.359999	426.000000	24.359236	0.314040	429.861053
24.379999	424.666992	24.379236	0.324714	429.300323
24.400000	436.500000	24.399237	0.335925	428.740143
24.420000	425.500000	24.419235	0.347709	428.180634
24.440001	416.332977	24.439236	0.360107	427.621552
24.459999	427.832977	24.459234	0.373162	427.063019
24.480000	425.166992	24.479235	0.386923	426.505096
24.500000	439.332977	24.499235	0.401441	425.947662
24.520000	427.332977	24.519236	0.416774	425.390900
24.540001	426.666992	24.539236	0.432985	424.834564
24.559999	410.500000	24.559235	0.450141	424.278961

24.580000	419.666992	24.579235	0.468322	423.723846
24.600000	437.832977	24.599236	0.487612	423.169098
24.620001	435.500000	24.619236	0.508104	422.614899
24.640001	432.000000	24.639236	0.529904	422.061310
24.660000	421.332977	24.659235	0.553127	421.508148
24.680000	430.500000	24.679235	0.577908	420.955658
24.700001	428.500000	24.699236	0.604392	420.403595
24.720001	416.500000	24.719236	0.632747	419.852142
24.740002	425.666992	24.739237	0.663162	419.301117
24.760000	420.500000	24.759235	0.695848	418.750702
24.780001	411.832977	24.779236	0.731057	418.200836
24.799999	419.500000	24.799234	0.769065	417.651337
24.820000	427.000000	24.819235	0.810207	417.102325
24.840000	432.500000	24.839235	0.854858	416.554047
24.859999	417.166992	24.859234	0.903458	416.006195
24.879999	430.832977	24.879234	0.956542	415.458710
24.900000	422.166992	24.899235	1.014727	414.911835
24.920000	422.166992	24.919237	1.078765	414.365387
24.940001	415.332977	24.939238	1.149547	413.819550
24.959999	412.166992	24.959236	1.228173	413.274017
24.980000	407.332977	24.979237	1.316020	412.729156
25.000000	395.166992	24.999237	1.414786	412.184784
25.020000	411.500000	25.019238	1.526633	411.640900
25.040001	421.832977	25.039238	1.654348	411.097382
25.059999	428.666992	25.059237	1.801563	410.554474
25.080000	402.666992	25.079237	1.973182	410.012054
25.100000	419.500000	25.099237	2.175840	409.470184
25.120001	409.166992	25.119238	2.418845	408.928680
25.140001	406.000000	25.139238	2.715705	408.387665
25.160000	418.000000	25.159237	3.087211	407.847260
25.180000	403.500000	25.179237	3.569160	407.307159
25.200001	406.666992	25.199238	4.231608	406.767548
25.220001	406.332977	25.219238	5.224264	406.228546
25.240002	415.166992	25.239239	6.859406	405.689850
25.260000	418.166992	25.259235	9.715014	405.151947
25.280001	415.332977	25.279236	14.707218	404.614227
25.299999	423.666992	25.299234	23.142096	404.077057
25.320000	434.332977	25.319235	37.052601	403.540253

25.340000	473.666992	25.339235	59.417316	403.003998
25.359999	503.666992	25.359234	84.885277	402.468231
25.379999	466.832977	25.379234	76.248405	401.932892
25.400000	442.166992	25.399235	40.802570	401.397919
25.420000	422.666992	25.419235	20.346035	400.863495
25.440001	407.000000	25.439236	13.133080	400.329559
25.459999	400.666992	25.459234	11.267286	399.796051
25.480000	414.166992	25.479235	11.035348	399.263092
25.500000	407.500000	25.499235	11.206629	398.730499
25.520000	413.666992	25.519236	11.582508	398.198395
25.540001	400.832977	25.539236	12.254354	397.666656
25.559999	392.832977	25.559235	13.329057	397.135468
25.580000	396.666992	25.579235	14.893609	396.604645
25.600000	399.166992	25.599236	17.049925	396.074371
25.620001	412.666992	25.619236	19.954620	395.544464
25.640001	412.332977	25.639236	23.861019	395.015045
25.660000	412.666992	25.659235	29.194389	394.485931
25.680000	414.666992	25.679237	36.741806	393.957428
25.700001	430.000000	25.699238	48.119415	393.429352
25.720001	426.832977	25.719238	66.821770	392.901642
25.740002	471.332977	25.739239	99.988541	392.374420
25.760000	521.000000	25.759237	160.420258	391.847565
25.780001	605.666992	25.779238	268.008179	391.321198
25.799999	782.166992	25.799236	451.986115	390.795441
25.820000	1081.669922	25.819237	759.713135	390.269928
25.840000	1464.000000	25.839237	1239.862305	389.744904
25.859999	1658.000000	25.859236	1631.085205	389.220245
25.879999	1411.830078	25.879236	1217.193481	388.696075
25.900000	923.666992	25.899237	567.355408	388.172394
25.920000	632.833008	25.919237	247.865494	387.649017
25.940001	496.000000	25.939238	134.442947	387.126129
25.959999	442.000000	25.959236	96.660126	386.603729
25.980000	420.666992	25.979237	79.198906	386.081696
26.000000	423.500000	25.999237	65.210472	385.560089
26.020000	398.666992	26.019238	52.329029	385.038849
26.040001	401.000000	26.039238	41.393116	384.518158
26.059999	393.666992	26.059237	32.824203	383.997833
26.080000	403.500000	26.079237	26.357685	383.477997

26.100000	391.166992	26.099237	21.500725	382.958466
26.120001	394.666992	26.119236	17.808449	382.439484
26.140001	382.500000	26.139236	14.952047	381.920807
26.160000	380.500000	26.159235	12.704412	381.402679
26.180000	388.500000	26.179235	10.907731	380.884918
26.200001	385.666992	26.199236	9.451740	380.367523
26.220001	391.332977	26.219236	8.257308	379.850555
26.240002	390.000000	26.239237	7.266690	379.334076
26.260000	366.166992	26.259235	6.437096	378.818146
26.280001	378.666992	26.279236	5.736052	378.302460
26.299999	385.832977	26.299234	5.138991	377.787201
26.320000	374.832977	26.319235	4.626673	377.272308
26.340000	378.832977	26.339235	4.184176	376.757843
26.359999	386.832977	26.359234	3.799674	376.243927
26.379999	375.166992	26.379234	3.463619	375.730377
26.400000	380.332977	26.399235	3.168405	375.217133
26.420000	398.166992	26.419235	2.907815	374.704376
26.440001	384.000000	26.439238	2.676739	374.191864
26.459999	380.000000	26.459236	2.471035	373.680084
26.480000	384.332977	26.479237	2.287151	373.168549
26.500000	371.832977	26.499237	2.122189	372.657440
26.520000	376.332977	26.519238	1.973697	372.146698
26.540001	381.666992	26.539238	1.839601	371.636444
26.559999	377.000000	26.559237	1.744244	371.126617
26.580000	379.500000	26.579237	1.634822	370.617157
26.600000	367.000000	26.599237	1.535286	370.108063
26.620001	380.000000	26.619238	1.444528	369.599518
26.640001	380.832977	26.639238	1.361590	369.091339
26.660000	366.332977	26.659237	1.285649	368.583466
26.680000	377.000000	26.679237	1.215967	368.076080
26.700001	370.166992	26.699238	1.151922	367.569122
26.720001	355.000000	26.719238	1.092959	367.062469
26.740002	371.000000	26.739239	1.038593	366.556305
26.760000	351.500000	26.759237	0.988402	366.050568
26.780001	347.666992	26.779238	0.922123	365.545258
26.799999	353.332977	26.799236	0.879811	365.040436
26.820000	365.166992	26.819237	0.840628	364.535919
26.840000	366.500000	26.839237	0.804318	364.031769

26.859999	368.666992	26.859236	0.770649	363.528107
26.879999	366.000000	26.879236	0.739411	363.024811
26.900000	358.166992	26.899237	0.710421	362.521942
26.920000	362.500000	26.919237	0.683516	362.019440
26.940001	376.832977	26.939238	0.658550	361.517365
26.959999	364.832977	26.959236	0.635395	361.015778
26.980000	353.332977	26.979237	0.613932	360.514557
27.000000	364.166992	26.999235	0.594062	360.013702
27.020000	352.832977	27.019236	0.575692	359.513397
27.040001	364.666992	27.039236	0.558745	359.013397
27.059999	350.832977	27.059235	0.543154	358.513763
27.080000	368.832977	27.079235	0.528856	358.014557
27.100000	354.000000	27.099236	0.515805	357.515717
27.120001	368.500000	27.119236	0.503958	357.017365
27.140001	358.832977	27.139236	0.493283	356.519379
27.160000	352.832977	27.159237	0.483757	356.021820
27.180000	362.000000	27.179237	0.475364	355.524628
27.200001	339.500000	27.199238	0.468098	355.027924
27.220001	352.832977	27.219238	0.461961	354.531647
27.240002	349.332977	27.239239	0.485191	354.035675
27.260000	348.000000	27.259237	0.482287	353.540253
27.280001	356.166992	27.279238	0.480618	353.045197
27.299999	354.166992	27.299236	0.480232	352.550629
27.320000	344.166992	27.319237	0.481190	352.056305
27.340000	360.166992	27.339237	0.483567	351.562469
27.359999	335.666992	27.359236	0.487456	351.069000
27.379999	351.666992	27.379236	0.492969	350.575897
27.400000	355.500000	27.399237	0.500243	350.083282
27.420000	346.000000	27.419237	0.509441	349.591095
27.440001	354.000000	27.439238	0.520757	349.099274
27.459999	354.166992	27.459236	0.534424	348.608124
27.480000	340.832977	27.479237	0.550725	348.117157
27.500000	345.332977	27.499237	0.569992	347.626617
27.520000	344.000000	27.519238	0.592630	347.136383
27.540001	351.832977	27.539238	0.621889	346.646698
27.559999	359.666992	27.559237	0.652936	346.157379
27.580000	350.000000	27.579237	0.689156	345.668549
27.600000	343.500000	27.599237	0.731425	345.180023

27.620001	347.000000	27.619238	0.780828	344.691986
27.640001	345.000000	27.639238	0.838715	344.204315
27.660000	346.166992	27.659237	0.906765	343.717133
27.680000	347.332977	27.679237	0.987123	343.230377
27.700001	360.500000	27.699238	1.082503	342.743927
27.720001	358.166992	27.719238	1.196409	342.258026
27.740002	379.666992	27.739239	1.333411	341.772491
27.760000	392.500000	27.759237	1.499528	341.287384
27.780001	393.832977	27.779238	1.702874	340.802643
27.799999	392.832977	27.799236	1.954406	340.318451
27.820000	395.666992	27.819237	2.269392	339.834503
27.840000	357.332977	27.839237	2.669298	339.351105
27.859999	357.000000	27.859234	3.185042	338.868195
27.879999	355.666992	27.879236	3.863082	338.385468
27.900000	351.500000	27.899237	4.775294	337.903229
27.920000	343.666992	27.919237	5.912590	337.421539
27.940001	339.500000	27.939238	7.769184	336.940155
27.959999	340.332977	27.959236	10.685955	336.459381
27.980000	355.832977	27.979237	15.668616	335.978973
28.000000	343.166992	27.999237	24.718140	335.498932
28.020000	354.166992	28.019238	41.417686	335.019257
28.040001	386.666992	28.039238	71.629410	334.540070
28.059999	443.000000	28.059237	124.828522	334.061249
28.080000	531.166992	28.079237	215.436340	333.582916
28.100000	642.000000	28.099237	341.770966	333.105011
28.120001	642.833008	28.119238	385.756287	332.627533
28.140001	543.833008	28.139238	246.478165	332.150421
28.160000	424.332977	28.159237	112.182594	331.673920
28.180000	384.666992	28.179237	51.657185	331.197723
28.200001	359.332977	28.199238	29.596598	330.722015
28.220001	334.166992	28.219238	21.095699	330.246613
28.240002	344.332977	28.239239	16.468180	329.771759
28.260000	343.000000	28.259237	12.950241	329.297211
28.280001	327.000000	28.279238	10.086180	328.823212
28.299999	323.500000	28.299236	7.865149	328.349762
28.320000	325.832977	28.319237	6.223691	327.876556
28.340000	333.166992	28.339237	5.037117	327.403839
28.359999	338.000000	28.359236	4.181098	326.931549

28.379999	325.666992	28.379236	3.559911	326.459686
28.400000	328.500000	28.399237	3.107645	325.988312
28.420000	332.332977	28.419237	2.780241	325.517426
28.440001	332.000000	28.439238	2.548445	325.046844
28.459999	337.500000	28.459236	2.392909	324.576935
28.480000	319.000000	28.479237	2.301055	324.107208
28.500000	317.332977	28.499237	2.265297	323.638092
28.520000	315.166992	28.519238	2.281880	323.169281
28.540001	324.832977	28.539238	2.350352	322.701019
28.559999	324.000000	28.559238	2.473411	322.233124
28.580000	324.832977	28.579239	2.657131	321.765717
28.600000	322.000000	28.599239	2.911595	321.298737
28.620001	331.666992	28.619240	3.252025	320.832245
28.640001	331.500000	28.639240	3.700663	320.366119
28.660000	331.832977	28.659239	4.289706	319.900543
28.680000	336.666992	28.679239	5.066390	319.435333
28.700001	321.666992	28.699240	6.100771	318.970673
28.720001	318.500000	28.719240	7.500558	318.506378
28.740002	326.832977	28.739241	9.440990	318.042572
28.760000	334.500000	28.759237	12.233802	317.579254
28.780001	314.832977	28.779238	16.498125	317.116425
28.799999	324.332977	28.799236	23.534744	316.653961
28.820000	340.166992	28.819237	36.044224	316.191925
28.840000	372.166992	28.839237	59.195766	315.730438
28.859999	386.000000	28.859236	101.976936	315.269318
28.879999	449.500000	28.879236	179.230408	314.808746
28.900000	572.500000	28.899237	315.193634	314.348602
28.920000	806.000000	28.919237	535.599426	313.888824
28.940001	976.833008	28.939238	756.069092	313.429596
28.959999	887.166992	28.959236	659.649902	312.970978
28.980000	634.000000	28.979237	347.780212	312.512665
29.000000	451.000000	28.999237	154.573593	312.054779
29.020000	367.666992	29.019238	77.035362	311.597382
29.040001	340.500000	29.039238	48.292454	311.140472
29.059999	331.500000	29.059237	35.467129	310.683990
29.080000	317.332977	29.079237	27.265873	310.227997
29.100000	324.332977	29.099237	20.875271	309.772491
29.120001	321.166992	29.119238	15.869328	309.317413

29.140001	308.832977	29.139238	12.114840	308.862885
29.160000	311.832977	29.159237	9.377020	308.408844
29.180000	323.332977	29.179237	7.387646	307.955292
29.200001	323.666992	29.199238	5.924695	307.502106
29.220001	318.500000	29.219238	4.828969	307.049347
29.240002	318.166992	29.239241	3.992517	306.596954
29.260000	319.832977	29.259239	3.342912	306.145416
29.280001	325.166992	29.279240	2.830335	305.694122
29.299999	317.166992	29.299238	2.420314	305.243378
29.320000	313.500000	29.319239	2.088182	304.793060
29.340000	317.000000	29.339239	1.816171	304.343231
29.359999	309.832977	29.359238	1.591179	303.893890
29.379999	312.666992	29.379238	1.403350	303.444916
29.400000	313.666992	29.399239	1.245268	302.996674
29.420000	307.332977	29.419239	1.111219	302.548798
29.440001	299.000000	29.439240	0.996767	302.101349
29.459999	312.666992	29.459238	0.898439	301.654388
29.480000	323.666992	29.479239	0.813457	301.207977
29.500000	305.166992	29.499239	0.739625	300.761993
29.520000	291.166992	29.519239	0.675163	300.316559
29.540001	320.832977	29.539240	0.618628	299.871552
29.559999	310.666992	29.559238	0.568839	299.427155
29.580000	301.166992	29.579239	0.524814	298.983124
29.600000	321.166992	29.599239	0.485749	298.539581
29.620001	326.500000	29.619240	0.450971	298.096588
29.640001	325.332977	29.639238	0.419919	297.654083
29.660000	330.000000	29.659237	0.392112	297.212189
29.680000	317.500000	29.679237	0.367146	296.770660
29.700001	313.000000	29.699238	0.319446	296.329620
29.720001	313.166992	29.719238	0.323764	295.889130
29.740002	305.332977	29.739239	0.306928	295.449005
29.760000	309.000000	29.759237	0.291825	295.009552
29.780001	311.166992	29.779238	0.278267	294.570526
29.799999	312.666992	29.799236	0.266100	294.131927
29.820000	308.500000	29.819237	0.255183	293.694000
29.840000	302.666992	29.839237	0.245397	293.256439
29.859999	304.500000	29.859236	0.236641	292.819366
29.879999	303.332977	29.879236	0.228820	292.382904

29.900000	291.166992	29.899239	0.221857	291.946869
29.920000	289.666992	29.919239	0.215684	291.511383
29.940001	308.166992	29.939240	0.210239	291.076385
29.959999	302.666992	29.959238	0.205471	290.641876
29.980000	290.166992	29.979239	0.201331	290.207855
30.000000	296.166992	29.999239	0.197782	289.774445
30.020000	286.000000	30.019239	0.194787	289.341461
30.040001	299.666992	30.039240	0.192317	288.909027
30.059999	296.500000	30.059238	0.190347	288.477203
30.080000	301.832977	30.079239	0.188854	288.045746
30.100000	291.000000	30.099239	0.187820	287.614899
30.120001	284.166992	30.119240	0.187229	287.184479
30.140001	298.500000	30.139240	0.187071	286.754669
30.160000	299.000000	30.159239	0.187335	286.325287
30.180000	288.666992	30.179239	0.195538	285.896454
30.200001	297.666992	30.199240	0.196808	285.468109
30.220001	290.166992	30.219240	0.198494	285.040375
30.240002	287.000000	30.239241	0.200596	284.613129
30.260000	300.166992	30.259239	0.203118	284.186554
30.280001	296.000000	30.279240	0.206065	283.760284
30.299999	289.832977	30.299238	0.209445	283.334686
30.320000	290.166992	30.319239	0.213269	282.909515
30.340000	290.500000	30.339239	0.217548	282.484894
30.359999	279.832977	30.359238	0.222298	282.060883
30.379999	290.000000	30.379238	0.227537	281.637299
30.400000	286.332977	30.399239	0.230718	281.214264
30.420000	292.832977	30.419239	0.237095	280.791779
30.440001	294.500000	30.439240	0.244027	280.369781
30.459999	285.166992	30.459238	0.251544	279.948395
30.480000	289.332977	30.479239	0.259680	279.527496
30.500000	287.500000	30.499239	0.268474	279.107086
30.520000	277.332977	30.519239	0.277967	278.687286
30.540001	288.666992	30.539238	0.288206	278.268036
30.559999	297.166992	30.559238	0.299245	277.849274
30.580000	281.666992	30.579239	0.311142	277.431061
30.600000	297.832977	30.599239	0.323960	277.013397
30.620001	303.166992	30.619240	0.337772	276.596283
30.640001	288.832977	30.639240	0.352658	276.179657

30.660000	302.166992	30.659239	0.368705	275.763641
30.680000	290.500000	30.679239	0.358608	275.348114
30.700001	302.666992	30.699240	0.378146	274.933197
30.720001	296.666992	30.719240	0.399144	274.518829
30.740002	313.666992	30.739241	0.421741	274.104950
30.760000	307.166992	30.759239	0.446093	273.691742
30.780001	287.166992	30.779240	0.472378	273.278961
30.799999	294.832977	30.799238	0.500788	272.866730
30.820000	291.166992	30.819239	0.531550	272.455048
30.840000	294.666992	30.839239	0.564912	272.043976
30.859999	289.500000	30.859238	0.601152	271.633453
30.879999	286.000000	30.879238	0.640598	271.223480
30.900000	295.500000	30.899239	0.683611	270.814056
30.920000	302.500000	30.919239	0.730606	270.405121
30.940001	301.832977	30.939240	0.782058	269.996796
30.959999	311.666992	30.959238	0.838507	269.589081
30.980000	330.000000	30.979239	0.900591	269.181854
31.000000	364.000000	30.999239	0.969030	268.775177
31.020000	392.166992	31.019239	1.044664	268.369049
31.040001	389.832977	31.039240	1.128471	267.963531
31.059999	380.666992	31.059238	1.221583	267.558502
31.080000	335.332977	31.079239	1.325358	267.154083
31.100000	324.166992	31.099239	1.441366	266.750275
31.120001	295.666992	31.119240	1.571476	266.346954
31.140001	304.666992	31.139240	1.717911	265.944183
31.160000	287.832977	31.159239	1.883312	265.542023
31.180000	290.666992	31.179241	2.070925	265.140411
31.200001	276.332977	31.199242	2.284568	264.739410
31.220001	271.332977	31.219242	2.528962	264.339020
31.240002	282.332977	31.239243	2.809870	263.939117
31.260000	277.000000	31.259241	3.134374	263.539764
31.280001	281.666992	31.279242	3.511377	263.141022
31.299999	274.000000	31.299240	3.951891	262.742950
31.320000	281.332977	31.319241	4.470015	262.345306
31.340000	281.166992	31.339241	5.083602	261.948273
31.359999	276.332977	31.359240	5.815655	261.551788
31.379999	278.166992	31.379240	6.696399	261.155945
31.400000	277.332977	31.399240	7.765484	260.760590

31.420000	279.832977	31.419241	9.076021	260.365845
31.440001	268.332977	31.439240	10.699894	259.971832
31.459999	284.166992	31.459238	12.736501	259.578247
31.480000	270.666992	31.479239	15.325402	259.185242
31.500000	272.332977	31.499239	18.665730	258.792816
31.520000	290.332977	31.519239	23.049057	258.401031
31.540001	273.666992	31.539240	28.914021	258.009735
31.559999	286.000000	31.559238	36.945827	257.619049
31.580000	294.500000	31.579239	48.285690	257.228943
31.600000	296.832977	31.599239	64.995789	256.839508
31.620001	306.166992	31.619240	91.161369	256.450592
31.640001	339.500000	31.639240	135.300568	256.062256
31.660000	384.666992	31.659239	214.981369	255.674530
31.680000	498.832977	31.679239	364.478455	255.287354
31.700001	712.000000	31.699240	646.277222	254.900726
31.720001	1154.169922	31.719240	1169.532959	254.514709
31.740002	2113.830078	31.739241	2110.676758	254.129395
31.760000	3558.830078	31.759239	3601.902588	253.744629
31.780001	4616.669922	31.779240	4920.293457	253.360413
31.799999	3913.500000	31.799240	4191.351562	252.976776
31.820000	2233.500000	31.819241	2258.723389	252.593719
31.840000	1068.830078	31.839241	1034.781616	252.211243
31.859999	602.500000	31.859240	519.055847	251.829407
31.879999	437.166992	31.879240	315.126984	251.448181
31.900000	364.000000	31.899240	221.036011	251.067505
31.920000	326.332977	31.919241	165.011703	250.687439
31.940001	323.500000	31.939241	126.416481	250.307953
31.959999	310.332977	31.959240	99.916016	249.929230
31.980000	300.332977	31.979240	83.430840	249.550964
32.000000	305.332977	31.999241	75.675560	249.173248
32.020000	301.666992	32.019238	76.538597	248.796265
32.040001	320.500000	32.039238	88.278549	248.419739
32.060001	325.166992	32.059238	117.543182	248.043854
32.080002	366.166992	32.079239	178.747452	247.668610
32.099998	485.332977	32.099236	299.342560	247.293976
32.119999	688.666992	32.119240	528.494568	246.919861
32.139999	1107.330078	32.139240	947.856995	246.546356
32.160000	1797.830078	32.159241	1639.534180	246.173553

32.180000	2387.170166	32.179241	2365.649170	245.801178
32.200001	2207.830078	32.199242	2215.934814	245.429565
32.220001	1391.169922	32.219242	1279.218018	245.058502
32.240002	746.666992	32.239243	593.793091	244.688080
32.260002	468.666992	32.259243	290.136719	244.318207
32.280003	354.166992	32.279243	169.263000	243.949005
32.299999	325.000000	32.299240	114.880806	243.580414
32.320000	306.832977	32.319241	83.420364	243.212402
32.340000	290.000000	32.339241	61.637402	242.845001
32.360001	281.500000	32.359241	45.852146	242.478210
32.380001	277.166992	32.379242	34.571186	242.111969
32.400002	292.000000	32.399242	26.645885	241.746429
32.420002	285.500000	32.419243	21.101765	241.381439
32.440002	296.500000	32.439243	17.203142	241.017059
32.460003	284.666992	32.459244	14.445404	240.653351
32.480003	271.332977	32.479244	12.498978	240.290161
32.500000	264.166992	32.499241	11.153083	239.927826
32.520000	264.832977	32.519241	10.274397	239.565918
32.540001	250.833008	32.539242	9.783466	239.204590
32.560001	249.667007	32.559242	9.639265	238.843933
32.580002	258.166992	32.579243	9.832449	238.483856
32.599998	254.667007	32.599239	10.383565	238.124481
32.619999	261.166992	32.619240	11.346884	237.765686
32.639999	246.667007	32.639240	12.818938	237.407440
32.660000	265.166992	32.659241	14.956491	237.049835
32.680000	259.832977	32.679241	18.008385	236.692871
32.700001	261.332977	32.699242	22.375715	236.336517
32.720001	259.832977	32.719242	28.735888	235.980743
32.740002	269.500000	32.739243	38.322880	235.625702
32.760002	280.500000	32.759243	53.577705	235.271179
32.780003	293.832977	32.779243	79.575218	234.917267
32.799999	324.666992	32.799240	126.795578	234.564026
32.820000	383.332977	32.819241	215.921417	234.211456
32.840000	508.832977	32.839241	385.337250	233.859406
32.860001	802.333008	32.859241	703.270325	233.507935
32.880001	1353.330078	32.879242	1280.276245	233.157196
32.900002	2225.670166	32.899242	2199.527588	232.807007
32.920002	2872.330078	32.919243	3037.218018	232.457489

32.940002	2571.000000	32.939243	2659.803467	232.108612
32.960003	1540.000000	32.959244	1476.639404	231.760376
32.980003	813.000000	32.979244	683.480774	231.412720
33.000000	476.332977	32.999241	337.847198	231.065765
33.020000	360.166992	33.019241	198.039749	230.719360
33.040001	311.500000	33.039242	133.135391	230.373566
33.060001	275.000000	33.059242	94.949425	230.028412
33.080002	269.000000	33.079243	68.686325	229.683899
33.099998	273.166992	33.099239	49.926170	229.340027
33.119999	258.500000	33.119240	36.659306	228.996857
33.139999	243.833008	33.139240	27.377657	228.654205
33.160000	248.500000	33.159241	20.863194	228.312164
33.180000	236.333008	33.179241	16.223949	227.970764
33.200001	245.833008	33.199242	12.854598	227.629974
33.220001	234.833008	33.219242	10.357638	227.289856
33.240002	240.667007	33.239243	8.472291	226.950348
33.260002	241.667007	33.259243	7.024856	226.611481
33.280003	233.833008	33.279243	5.897223	226.273224
33.299999	239.333008	33.299240	5.007557	225.935608
33.320000	229.833008	33.319244	4.297404	225.598572
33.340000	232.333008	33.339245	3.725480	225.262238
33.360001	242.667007	33.359245	3.261146	224.926514
33.380001	238.500000	33.379246	2.881844	224.591400
33.400002	235.500000	33.399246	2.570700	224.256927
33.420002	229.167007	33.419247	2.314991	223.923065
33.440002	239.500000	33.439247	2.105074	223.589935
33.460003	235.000000	33.459248	1.933643	223.257355
33.480003	243.667007	33.479248	1.795197	222.925354
33.500000	240.500000	33.499245	1.685698	222.594116
33.520000	241.167007	33.519245	1.602233	222.263458
33.540001	241.000000	33.539246	1.542982	221.933380
33.560001	229.167007	33.559246	1.507042	221.603973
33.580002	236.000000	33.579247	1.494432	221.275208
33.599998	222.667007	33.599243	1.506136	220.947113
33.619999	221.333008	33.619244	1.544249	220.619598
33.639999	235.667007	33.639244	1.612183	220.292694
33.660000	223.333008	33.659245	1.715036	219.966461
33.680000	236.667007	33.679245	1.860135	219.640808

33.700001	222.833008	33.699245	2.057859	219.315857
33.720001	230.333008	33.719246	2.322902	218.991486
33.740002	236.500000	33.739243	2.676108	218.667847
33.760002	235.500000	33.759243	3.147778	218.344849
33.780003	230.500000	33.779243	3.782094	218.022369
33.799999	239.000000	33.799240	4.645165	217.700623
33.820000	236.167007	33.819241	5.839608	217.379456
33.840000	241.500000	33.839241	7.530039	217.058868
33.860001	238.667007	33.859241	9.999275	216.739014
33.880001	235.000000	33.879242	13.761736	216.419708
33.900002	236.167007	33.899242	19.903896	216.101105
33.920002	243.833008	33.919243	30.613010	215.783112
33.940002	246.000000	33.939243	50.402458	215.465759
33.960003	290.000000	33.959244	88.082207	215.148956
33.980003	349.000000	33.979244	160.015701	214.832947
34.000000	491.832977	33.999241	295.111237	214.517487
34.020000	715.333008	34.019241	535.937256	214.202667
34.040001	1070.669922	34.039242	883.558899	213.888458
34.060001	1227.830078	34.059242	1089.325806	213.574982
34.080002	1027.000000	34.079243	829.614990	213.262024
34.099998	592.500000	34.099239	430.286987	212.949860
34.119999	389.500000	34.119240	200.660904	212.638245
34.139999	302.666992	34.139240	103.181435	212.327240
34.160000	290.332977	34.159241	62.265755	212.016815
34.180000	264.166992	34.179241	41.999348	211.707092
34.200001	261.832977	34.199242	29.633598	211.397949
34.220001	256.000000	34.219242	21.172220	211.089478
34.240002	241.000000	34.239243	15.245179	210.781647
34.260002	238.833008	34.259243	11.124730	210.474518
34.280003	248.000000	34.279243	8.271244	210.167969
34.299999	281.332977	34.299240	6.279041	209.862030
34.320000	291.832977	34.319241	4.863564	209.556763
34.340000	290.332977	34.339241	3.837159	209.252167
34.360001	290.500000	34.359241	3.077509	208.948059
34.380001	262.666992	34.379242	2.504602	208.644714
34.400002	248.000000	34.399242	2.065163	208.341980
34.420002	233.500000	34.419243	1.722962	208.039795
34.440002	222.833008	34.439243	1.452844	207.738403

34.460003	219.500000	34.459248	1.236968	207.437439
34.480003	221.333008	34.479248	1.062596	207.137268
34.500000	205.667007	34.499245	0.920328	206.837799
34.520000	220.833008	34.519245	0.803153	206.538910
34.540001	218.333008	34.539246	0.705869	206.240570
34.560001	220.667007	34.559246	0.624494	205.942871
34.580002	225.000000	34.579247	0.555969	205.645813
34.599998	222.833008	34.599243	0.497925	205.349426
34.619999	218.500000	34.619244	0.448476	205.053650
34.639999	216.333008	34.639244	0.406163	204.758514
34.660000	216.167007	34.659245	0.369814	204.463989
34.680000	214.833008	34.679245	0.328015	204.170197
34.700001	210.333008	34.699245	0.290807	203.876953
34.720001	208.333008	34.719246	0.267890	203.584259
34.740002	215.000000	34.739246	0.248132	203.292358
34.760002	208.500000	34.759247	0.231137	203.001038
34.780003	206.667007	34.779247	0.216588	202.710327
34.799999	206.167007	34.799244	0.204238	202.420319
34.820000	216.167007	34.819244	0.193887	202.130859
34.840000	210.333008	34.839245	0.185397	201.842072
34.860001	201.667007	34.859245	0.178669	201.553894
34.880001	212.333008	34.879246	0.173654	201.266357
34.900002	222.500000	34.899246	0.170344	200.979431
34.920002	205.500000	34.919247	0.168782	200.693146
34.940002	205.333008	34.939247	0.169062	200.407532
34.960003	205.833008	34.959248	0.171339	200.122498
34.980003	201.667007	34.979248	0.175844	199.838104
35.000000	214.167007	34.999245	0.182898	199.554413
35.020000	208.500000	35.019245	0.192947	199.271271
35.040001	208.833008	35.039246	0.206588	198.988800
35.060001	204.333008	35.059246	0.224629	198.706909
35.080002	210.833008	35.079247	0.248167	198.425629
35.099998	216.333008	35.099243	0.278688	198.144989
35.119999	207.333008	35.119244	0.318266	197.865021
35.139999	207.000000	35.139244	0.369770	197.585754
35.160000	196.833008	35.159245	0.437280	197.306976
35.180000	206.500000	35.179245	0.526678	197.028870
35.200001	208.000000	35.199245	0.646630	196.751434

35.220001	197.500000	35.219246	0.810213	196.474579
35.240002	203.833008	35.239246	1.037798	196.198303
35.260002	197.000000	35.259247	1.362564	195.922760
35.280003	201.667007	35.279247	1.842156	195.647766
35.299999	197.833008	35.299244	2.584747	195.373474
35.320000	197.167007	35.319244	3.808728	195.099731
35.340000	205.833008	35.339245	5.966704	194.826599
35.360001	200.833008	35.359245	9.984941	194.554108
35.380001	216.667007	35.379246	17.682846	194.282288
35.400002	223.000000	35.399246	32.478142	194.011017
35.420002	264.000000	35.419247	60.420624	193.740479
35.440002	313.166992	35.439247	109.870430	193.470459
35.460003	382.500000	35.459248	178.176773	193.201111
35.480003	409.000000	35.479248	212.194412	192.932373
35.500000	351.166992	35.499245	157.745148	192.664337
35.520000	277.666992	35.519245	81.925400	192.396851
35.540001	239.167007	35.539246	38.662449	192.129974
35.560001	224.833008	35.559246	19.983801	191.863678
35.580002	216.333008	35.579250	11.945817	191.597992
35.599998	216.000000	35.599247	7.914484	191.333069
35.619999	207.833008	35.619247	5.483693	191.068634
35.639999	210.167007	35.639244	3.859520	190.804932
35.660000	209.500000	35.659245	2.745172	190.541718
35.680000	195.500000	35.679245	1.982137	190.279144
35.700001	194.000000	35.699245	1.452153	190.017273
35.720001	203.500000	35.719246	1.090620	189.755951
35.740002	187.000000	35.739246	0.835907	189.495239
35.760002	188.667007	35.759247	0.652656	189.235199
35.780003	186.667007	35.779247	0.518062	188.975708
35.799999	186.333008	35.799244	0.417321	188.716919
35.820000	194.167007	35.819244	0.340584	188.458679
35.840000	194.167007	35.839245	0.281238	188.201019
35.860001	194.833008	35.859245	0.234703	187.944000
35.880001	192.667007	35.879246	0.197754	187.687592
35.900002	188.833008	35.899246	0.168081	187.431793
35.920002	195.000000	35.919247	0.144003	187.176605
35.940002	193.167007	35.939247	0.124281	186.922058
35.960003	185.667007	35.959248	0.107984	186.668091

35.980003	184.333008	35.979248	0.094409	186.414703
36.000000	189.500000	35.999245	0.083020	186.162079
36.020000	169.000000	36.019245	0.067634	185.909973
36.040001	183.167007	36.039246	0.059640	185.658417
36.060001	190.667007	36.059246	0.052821	185.407501
36.080002	185.833008	36.079247	0.046972	185.157257
36.099998	190.667007	36.099243	0.041931	184.907593
36.119999	181.000000	36.119244	0.037564	184.658478
36.139999	199.000000	36.139244	0.033765	184.410004
36.160000	182.667007	36.159245	0.030447	184.162079
36.180000	191.000000	36.179245	0.027536	183.914825
36.200001	183.833008	36.199245	0.024973	183.668152
36.220001	182.000000	36.219246	0.022710	183.422089
36.240002	181.167007	36.239246	0.020703	183.176544
36.260002	185.000000	36.259247	0.018919	182.931702
36.280003	187.833008	36.279247	0.017328	182.687439
36.299999	189.500000	36.299244	0.015906	182.443817
36.320000	182.667007	36.319244	0.014629	182.200775
36.340000	179.667007	36.339245	0.013482	181.958282
36.360001	180.167007	36.359245	0.012448	181.716431
36.380001	176.667007	36.379246	0.011514	181.475159
36.400002	172.167007	36.399246	0.010668	181.234497
36.420002	169.833008	36.419247	0.009900	180.994415
36.440002	174.333008	36.439247	0.009203	180.754944
36.460003	179.500000	36.459248	0.008567	180.516022
36.480003	182.833008	36.479248	0.007986	180.277740
36.500000	170.167007	36.499245	0.007456	180.040070
36.520000	174.833008	36.519245	0.006969	179.802948
36.540001	186.500000	36.539246	0.006523	179.566467
36.560001	181.500000	36.559246	0.006113	179.330505
36.580002	184.167007	36.579247	0.005736	179.095154
36.599998	179.333008	36.599243	0.005388	178.860474
36.619999	176.667007	36.619244	0.005067	178.626343
36.639999	184.167007	36.639244	0.004770	178.392731
36.660000	181.833008	36.659245	0.004557	178.159760
36.680000	182.667007	36.679249	0.004305	177.927338
36.700001	183.000000	36.699249	0.004072	177.695496
36.720001	171.000000	36.719250	0.003855	177.464325

36.740002	180.667007	36.739250	0.003654	177.233704
36.760002	177.667007	36.759251	0.003468	177.003632
36.780003	169.833008	36.779251	0.003294	176.774200
36.799999	175.667007	36.799248	0.003133	176.545349
36.820000	180.000000	36.819248	0.002983	176.317047
36.840000	169.500000	36.839249	0.002843	176.089355
36.860001	182.500000	36.859249	0.002713	175.862213
36.880001	181.000000	36.879250	0.002592	175.635681
36.900002	175.333008	36.899250	0.002479	175.409668
36.920002	174.000000	36.919250	0.002374	175.184296
36.940002	176.500000	36.939251	0.002276	174.959473
36.960003	172.500000	36.959251	0.002185	174.735260
36.980003	173.667007	36.979252	0.002100	174.511566
37.000000	180.333008	36.999249	0.002021	174.288483
37.020000	173.500000	37.019249	0.001949	174.066010
37.040001	169.333008	37.039249	0.001881	173.844055
37.060001	172.667007	37.059250	0.001819	173.622650
37.080002	177.000000	37.079250	0.000202	173.401886
37.099998	178.167007	37.099247	0.000216	173.181702
37.119999	175.000000	37.119247	0.000231	172.962006
37.139999	179.000000	37.139248	0.000247	172.742950
37.160000	172.667007	37.159248	0.000265	172.524445
37.180000	175.000000	37.179249	0.000285	172.306488
37.200001	182.000000	37.199249	0.000307	172.089142
37.220001	162.500000	37.219250	0.000330	171.872314
37.240002	172.833008	37.239250	0.000356	171.656067
37.260002	179.500000	37.259251	0.000385	171.440369
37.280003	177.833008	37.279251	0.000417	171.225281
37.299999	185.167007	37.299248	0.000452	171.010773
37.320000	177.333008	37.319248	0.000491	170.796783
37.340000	172.833008	37.339249	0.000534	170.583374
37.360001	184.833008	37.359249	0.000582	170.370483
37.380001	171.333008	37.379250	0.000636	170.158203
37.400002	175.667007	37.399250	0.000696	169.946442
37.420002	174.667007	37.419250	0.000764	169.735229
37.440002	163.167007	37.439251	0.000840	169.524628
37.460003	166.167007	37.459251	0.000926	169.314514
37.480003	164.667007	37.479252	0.001024	169.105011

37.500000	160.667007	37.499249	0.001135	168.896088
37.520000	153.500000	37.519249	0.001262	168.687683
37.540001	161.833008	37.539249	0.001408	168.479828
37.560001	168.000000	37.559246	0.001575	168.272552
37.580002	169.333008	37.579247	0.002341	168.065796
37.599998	167.167007	37.599243	0.002593	167.859619
37.619999	160.000000	37.619244	0.002884	167.653961
37.639999	166.667007	37.639244	0.003429	167.448914
37.660000	167.667007	37.659245	0.003834	167.244324
37.680000	169.667007	37.679245	0.004309	167.040283
37.700001	160.833008	37.699245	0.004870	166.836884
37.720001	164.333008	37.719246	0.005538	166.633911
37.740002	163.000000	37.739250	0.006338	166.431488
37.760002	167.000000	37.759251	0.007306	166.229675
37.780003	166.000000	37.779251	0.008487	166.028381
37.799999	171.333008	37.799248	0.009942	165.827667
37.820000	171.667007	37.819248	0.011755	165.627441
37.840000	174.000000	37.839249	0.014040	165.427826
37.860001	161.667007	37.859249	0.016959	165.228607
37.880001	158.333008	37.879250	0.021902	165.030029
37.900002	164.000000	37.899250	0.026921	164.832001
37.920002	168.333008	37.919250	0.033626	164.634460
37.940002	160.667007	37.939251	0.042763	164.437469
37.960003	165.333008	37.959251	0.055501	164.241058
37.980003	169.500000	37.979252	0.073750	164.045166
38.000000	167.833008	37.999249	0.100811	163.849731
38.020000	166.167007	38.019249	0.142845	163.654907
38.040001	160.500000	38.039249	0.212053	163.460541
38.060001	157.333008	38.059250	0.333576	163.266785
38.080002	162.667007	38.079250	0.559197	163.073517
38.099998	158.333008	38.099247	0.992726	162.880829
38.119999	153.167007	38.119247	1.835176	162.688629
38.139999	164.000000	38.139248	3.451448	162.496918
38.160000	161.000000	38.159248	6.372727	162.305786
38.180000	169.333008	38.179249	10.651409	162.115143
38.200001	164.000000	38.199249	13.650867	161.925049
38.220001	165.833008	38.219250	11.360122	161.735413
38.240002	160.500000	38.239250	6.439176	161.546356

38.260002	162.500000	38.259251	3.121355	161.357819
38.280003	157.167007	38.279251	1.573304	161.169769
38.299999	157.167007	38.299248	0.894866	160.982239
38.320000	152.333008	38.319248	0.566374	160.795288
38.340000	157.667007	38.339249	0.381207	160.608795
38.360001	158.500000	38.359249	0.264847	160.422821
38.380001	154.667007	38.379250	0.188508	160.237366
38.400002	154.333008	38.399250	0.138063	160.052429
38.420002	152.500000	38.419250	0.104770	159.867950
38.440002	149.333008	38.439251	0.082787	159.684052
38.460003	157.000000	38.459251	0.068261	159.500671
38.480003	157.000000	38.479252	0.058728	159.317749
38.500000	148.500000	38.499249	0.052634	159.135376
38.520000	155.833008	38.519249	0.049003	158.953461
38.540001	156.167007	38.539249	0.047224	158.772095
38.560001	150.167007	38.559250	0.046915	158.591248
38.580002	148.333008	38.579250	0.047849	158.410858
38.599998	160.000000	38.599247	0.049906	158.231079
38.619999	151.167007	38.619247	0.053048	158.051666
38.639999	162.500000	38.639248	0.057303	157.872742
38.660000	156.167007	38.659248	0.062762	157.694427
38.680000	153.500000	38.679249	0.069579	157.516541
38.700001	157.000000	38.699249	0.077980	157.339203
38.720001	151.833008	38.719250	0.088276	157.162292
38.740002	158.000000	38.739250	0.100890	156.985962
38.760002	161.667007	38.759254	0.116391	156.810028
38.780003	154.167007	38.779255	0.135534	156.634644
38.799999	158.333008	38.799252	0.159346	156.459778
38.820000	156.167007	38.819252	0.189244	156.285309
38.840000	144.000000	38.839252	0.227171	156.111450
38.860001	152.000000	38.859253	0.276093	155.937988
38.880001	152.833008	38.879253	0.339472	155.765076
38.900002	152.833008	38.899254	0.423213	155.592590
38.920002	154.333008	38.919254	0.535768	155.420593
38.940002	154.833008	38.939255	0.690017	155.249176
38.960003	154.833008	38.959255	0.906195	155.078156
38.980003	147.500000	38.979256	1.217443	154.907654
39.000000	161.333008	38.999252	1.681083	154.737671

39.020000	154.833008	39.019253	2.403824	154.568115
39.040001	157.833008	39.039253	3.596313	154.399017
39.060001	146.333008	39.059254	5.691349	154.230438
39.080002	157.500000	39.079254	9.581011	154.062347
39.099998	166.500000	39.099251	17.062378	153.894775
39.119999	172.167007	39.119251	31.640362	153.727600
39.139999	206.500000	39.139252	59.714699	153.560944
39.160000	267.500000	39.159252	110.659271	153.394745
39.180000	368.666992	39.179253	185.970779	153.228973
39.200001	435.000000	39.199253	241.835342	153.063751
39.220001	374.332977	39.219254	206.776062	152.898926
39.240002	290.832977	39.239254	120.286385	152.734558
39.260002	211.833008	39.259254	59.012108	152.570770
39.280003	182.000000	39.279255	29.658459	152.407349
39.299999	161.000000	39.299252	16.666704	152.244476
39.320000	153.167007	39.319252	10.415619	152.082001
39.340000	146.500000	39.339252	6.960488	151.920074
39.360001	147.667007	39.359253	4.843162	151.758484
39.380001	155.500000	39.379253	3.493090	151.597473
39.400002	151.500000	39.399254	2.635853	151.436859
39.420002	148.500000	39.419254	2.109022	151.276703
39.440002	154.167007	39.439255	1.809283	151.117035
39.460003	150.667007	39.459255	1.673390	150.957794
39.480003	150.667007	39.479256	1.667011	150.798981
39.500000	150.833008	39.499252	1.777279	150.640747
39.520000	149.667007	39.519253	2.009129	150.482941
39.540001	148.333008	39.539253	2.385192	150.325500
39.560001	141.667007	39.559250	2.950004	150.168579
39.580002	151.667007	39.579250	3.780750	150.012115
39.599998	150.000000	39.599247	5.007468	149.856079
39.619999	153.833008	39.619247	6.858374	149.700470
39.639999	162.167007	39.639248	9.754161	149.545349
39.660000	160.167007	39.659248	14.521070	149.390656
39.680000	166.500000	39.679249	22.849110	149.236420
39.700001	167.833008	39.699249	38.227875	149.082611
39.720001	201.167007	39.719250	67.742592	148.929230
39.740002	241.833008	39.739250	125.327721	148.776306
39.760002	373.832977	39.759254	236.923264	148.623810

39.780003	603.166992	39.779255	442.806274	148.471771
39.799999	945.666992	39.799252	763.394714	148.320190
39.820000	1219.169922	39.819252	1052.064941	148.169037
39.840000	1173.330078	39.839252	976.320618	148.018311
39.860001	782.000000	39.859253	604.284546	147.868042
39.880001	452.666992	39.879253	302.124451	147.718231
39.900002	282.832977	39.899254	149.603394	147.568787
39.920002	219.833008	39.919254	81.522751	147.419800
39.940002	180.000000	39.939255	49.302441	147.271271
39.960003	177.000000	39.959255	31.885584	147.123199
39.980003	159.667007	39.979256	21.328627	146.975433
40.000000	156.833008	39.999252	14.543917	146.828278
40.020000	158.167007	40.019253	10.092762	146.681427
40.040001	150.833008	40.039253	7.149143	146.535065
40.060001	143.833008	40.059254	5.182076	146.389099
40.080002	149.833008	40.079254	3.846959	146.243561
40.099998	151.333008	40.099251	2.924344	146.098419
40.119999	147.167007	40.119251	2.275753	145.953766
40.139999	142.833008	40.139252	1.814553	145.809479
40.160000	146.333008	40.159252	1.485930	145.665619
40.180000	150.667007	40.179253	1.255488	145.522247
40.200001	145.167007	40.199253	1.102615	145.379181
40.220001	138.333008	40.219254	1.017113	145.236572
40.240002	135.500000	40.239254	0.998390	145.094452
40.260002	144.833008	40.259254	1.057590	144.952637
40.280003	139.333008	40.279255	1.224776	144.811310
40.299999	144.500000	40.299252	1.566360	144.670380
40.320000	141.167007	40.319252	2.224512	144.529846
40.340000	134.667007	40.339252	3.495156	144.389740
40.360001	140.667007	40.359253	5.984345	144.250031
40.380001	147.167007	40.379253	10.886646	144.110748
40.400002	155.833008	40.399254	20.418226	143.971893
40.420002	175.667007	40.419254	37.953552	143.833405
40.440002	189.167007	40.439255	64.843491	143.695343
40.460003	217.500000	40.459255	87.977486	143.557678
40.480003	207.167007	40.479256	80.407631	143.420395
40.500000	181.333008	40.499252	49.532482	143.283600
40.520000	156.333008	40.519253	24.891481	143.147125

40.540001	147.833008	40.539253	12.439965	143.011063
40.560001	141.167007	40.559254	6.843645	142.875443
40.580002	135.833008	40.579254	4.184268	142.740173
40.599998	133.667007	40.599251	2.758526	142.605301
40.619999	139.500000	40.619251	1.919535	142.470856
40.639999	146.333008	40.639252	1.419628	142.336792
40.660000	141.333008	40.659252	1.157355	142.203140
40.680000	141.667007	40.679253	1.104033	142.069839
40.700001	133.000000	40.699253	1.299673	141.936981
40.720001	140.667007	40.719254	1.885325	141.804474
40.740002	142.667007	40.739258	3.170783	141.672363
40.760002	140.333008	40.759258	5.721078	141.540619
40.780003	146.000000	40.779259	10.226049	141.409286
40.799999	162.333008	40.799255	16.113052	141.278351
40.820000	149.000000	40.819256	18.808744	141.147827
40.840000	145.333008	40.839256	14.445871	141.017670
40.860001	147.833008	40.859257	8.007598	140.887909
40.880001	140.167007	40.879257	3.965354	140.758499
40.900002	138.833008	40.899258	2.059982	140.629486
40.920002	148.833008	40.919258	1.188972	140.500839
40.940002	144.667007	40.939259	0.750237	140.372589
40.960003	143.333008	40.959259	0.499614	140.244690
40.980003	142.667007	40.979259	0.343409	140.117203
41.000000	141.333008	40.999256	0.242068	139.990097
41.020000	144.167007	41.019257	0.175217	139.863342
41.040001	144.833008	41.039257	0.130596	139.737000
41.060001	149.667007	41.059258	0.100336	139.610977
41.080002	151.167007	41.079258	0.079407	139.485367
41.099998	147.333008	41.099255	0.064634	139.360138
41.119999	143.667007	41.119255	0.054006	139.235229
41.139999	132.333008	41.139256	0.046251	139.110733
41.160000	143.667007	41.159256	0.040542	138.986557
41.180000	141.333008	41.179256	0.036332	138.862823
41.200001	140.833008	41.199257	0.033254	138.739410
41.220001	141.333008	41.219257	0.031060	138.616333
41.240002	141.000000	41.239258	0.029578	138.493652
41.260002	136.667007	41.259258	0.028696	138.371353
41.280003	133.667007	41.279259	0.028337	138.249390

41.299999	136.167007	41.299255	0.028459	138.127838
41.320000	139.000000	41.319256	0.029040	138.006607
41.340000	138.833008	41.339256	0.030081	137.885696
41.360001	140.333008	41.359257	0.031602	137.765182
41.380001	133.000000	41.379257	0.033639	137.645035
41.400002	136.500000	41.399258	0.036253	137.525253
41.420002	140.000000	41.419258	0.039523	137.405792
41.440002	132.667007	41.439259	0.043560	137.286682
41.460003	134.333008	41.459259	0.048506	137.167953
41.480003	138.333008	41.479259	0.054546	137.049561
41.500000	136.500000	41.499256	0.061921	136.931564
41.520000	130.667007	41.519257	0.070406	136.813843
41.540001	134.667007	41.539257	0.081519	136.696503
41.560001	136.833008	41.559258	0.095236	136.579514
41.580002	127.833000	41.579258	0.112282	136.462875
41.599998	134.000000	41.599251	0.133622	136.346619
41.619999	137.833008	41.619251	0.160603	136.230652
41.639999	150.167007	41.639252	0.195053	136.115051
41.660000	146.500000	41.659252	0.239545	135.999771
41.680000	148.667007	41.679253	0.297748	135.884888
41.700001	158.333008	41.699257	0.374997	135.770233
41.720001	143.667007	41.719257	0.479136	135.656036
41.740002	148.500000	41.739258	0.622093	135.542114
41.760002	134.500000	41.759258	0.822387	135.428558
41.780003	138.333008	41.779259	1.109802	135.315338
41.799999	141.167007	41.799255	1.533196	135.202469
41.820000	145.500000	41.819256	2.184219	135.089920
41.840000	148.500000	41.839256	3.230846	134.977692
41.860001	135.167007	41.859257	5.011364	134.865829
41.880001	137.500000	41.879257	8.220894	134.754257
41.900002	142.333008	41.899258	14.288175	134.643051
41.920002	157.000000	41.919258	26.092638	134.532150
41.940002	170.333008	41.939259	49.210361	134.421585
41.960003	211.000000	41.959259	93.361443	134.311325
41.980003	291.166992	41.979259	169.709473	134.201431
42.000000	376.332977	41.999256	266.881134	134.091873
42.020000	411.000000	42.019257	308.928101	133.982651
42.040001	365.000000	42.039257	236.938385	133.873688

42.060001	261.166992	42.059258	132.118561	133.765091
42.080002	194.167007	42.079258	65.660545	133.656799
42.099998	161.167007	42.099255	33.949673	133.548828
42.119999	146.500000	42.119255	19.372776	133.441208
42.139999	145.000000	42.139256	12.121496	133.333878
42.160000	138.500000	42.159256	8.155719	133.226883
42.180000	140.333008	42.179256	5.939230	133.120209
42.200001	135.000000	42.199257	4.914870	133.013885
42.220001	139.500000	42.219257	4.999184	132.907776
42.240002	139.833008	42.239258	6.524948	132.802078
42.260002	133.333008	42.259258	10.405395	132.696671
42.280003	155.000000	42.279259	18.346647	132.591492
42.299999	167.833008	42.299255	32.174416	132.486755
42.320000	187.167007	42.319256	49.096039	132.382278
42.340000	193.500000	42.339256	54.856434	132.278061
42.360001	166.333008	42.359257	40.844318	132.174225
42.380001	144.167007	42.379257	22.533695	132.070633
42.400002	133.667007	42.399258	11.271672	131.967422
42.420002	137.333008	42.419258	5.906124	131.864456
42.440002	128.833008	42.439259	3.403102	131.761841
42.460003	136.333008	42.459259	2.123924	131.659500
42.480003	137.833008	42.479259	1.392529	131.557449
42.500000	135.333008	42.499256	0.940487	131.455734
42.520000	140.667007	42.519257	0.649948	131.354324
42.540001	127.167000	42.539257	0.459731	131.253235
42.560001	124.500000	42.559258	0.333292	131.152359
42.580002	130.833008	42.579258	0.247675	131.051819
42.599998	125.833000	42.599255	0.188408	130.951675
42.619999	141.000000	42.619255	0.146386	130.851761
42.639999	132.500000	42.639259	0.115905	130.752075
42.660000	126.667000	42.659260	0.093333	130.652756
42.680000	134.000000	42.679260	0.076296	130.553726
42.700001	131.167007	42.699261	0.063221	130.454926
42.720001	130.667007	42.719261	0.053036	130.356506
42.740002	122.833000	42.739262	0.045001	130.258347
42.760002	136.000000	42.759262	0.038659	130.160461
42.780003	118.667000	42.779263	0.033496	130.062866
42.799999	125.667000	42.799259	0.029304	129.965607

42.820000	128.333008	42.819260	0.025876	129.868591
42.840000	130.167007	42.839260	0.023060	129.771896
42.860001	130.333008	42.859261	0.020741	129.675461
42.880001	124.500000	42.879261	0.018829	129.579315
42.900002	126.667000	42.899261	0.017256	129.483429
42.920002	130.167007	42.919262	0.015970	129.387894
42.940002	128.333008	42.939262	0.014929	129.292587
42.960003	133.333008	42.959263	0.014103	129.197586
42.980003	129.333008	42.979263	0.013468	129.102844
43.000000	128.167007	42.999260	0.013006	129.008453
43.020000	125.667000	43.019260	0.012705	128.914261
43.040001	129.667007	43.039261	0.012558	128.820374
43.060001	127.333000	43.059261	0.012561	128.726746
43.080002	122.500000	43.079262	0.012715	128.633438
43.099998	130.500000	43.099258	0.013024	128.540405
43.119999	130.167007	43.119259	0.013498	128.447601
43.139999	126.500000	43.139259	0.014149	128.355133
43.160000	122.333000	43.159260	0.014997	128.262894
43.180000	124.167000	43.179260	0.016066	128.170914
43.200001	124.333000	43.199261	0.017388	128.079224
43.220001	119.333000	43.219261	0.019003	127.987808
43.240002	125.667000	43.239262	0.020963	127.896667
43.260002	134.167007	43.259262	0.023334	127.805801
43.280003	132.500000	43.279263	0.026199	127.715195
43.299999	124.333000	43.299259	0.029665	127.624878
43.320000	134.167007	43.319260	0.033868	127.534760
43.340000	129.667007	43.339260	0.038983	127.444992
43.360001	125.833000	43.359261	0.045241	127.355423
43.380001	133.000000	43.379261	0.052940	127.266174
43.400002	127.333000	43.399261	0.062477	127.177139
43.420002	125.333000	43.419262	0.074385	127.088379
43.440002	134.667007	43.439262	0.089384	126.999893
43.460003	134.500000	43.459263	0.108462	126.911652
43.480003	127.667000	43.479263	0.132992	126.823685
43.500000	126.500000	43.499260	0.164908	126.735992
43.520000	121.000000	43.519260	0.207010	126.648575
43.540001	133.000000	43.539261	0.263368	126.561356
43.560001	133.833008	43.559265	0.340082	126.474396

43.580002	133.500000	43.579266	0.446421	126.387756
43.599998	131.667007	43.599262	0.597115	126.301346
43.619999	133.000000	43.619263	0.815676	126.215210
43.639999	126.333000	43.639263	1.142651	126.129272
43.660000	132.500000	43.659264	1.650696	126.043625
43.680000	132.667007	43.679264	2.479185	125.958191
43.700001	135.167007	43.699261	3.906182	125.873062
43.720001	134.500000	43.719261	6.503981	125.788177
43.740002	136.333008	43.739262	11.446670	125.703491
43.760002	140.333008	43.759262	21.102861	125.619110
43.780003	162.500000	43.779263	40.029510	125.534943
43.799999	206.500000	43.799259	75.881088	125.451065
43.820000	264.332977	43.819260	136.029968	125.367401
43.840000	335.500000	43.839260	206.756882	125.283997
43.860001	339.166992	43.859261	228.468842	125.200790
43.880001	303.000000	43.879261	169.787186	125.117905
43.900002	228.000000	43.899261	94.318848	125.035202
43.920002	181.500000	43.919262	47.389122	124.952759
43.940002	155.333008	43.939262	24.683964	124.870544
43.960003	140.000000	43.959263	13.986228	124.788605
43.980003	128.167007	43.979263	8.530735	124.706879
44.000000	132.667007	43.999260	5.456762	124.625458
44.020000	124.000000	44.019260	3.595877	124.544220
44.040001	125.667000	44.039261	2.427939	124.463242
44.060001	131.333008	44.059261	1.683145	124.382477
44.080002	120.333000	44.079262	1.204310	124.301926
44.099998	116.833000	44.099258	0.895511	124.221664
44.119999	126.333000	44.119259	0.698216	124.141647
44.139999	120.333000	44.139259	0.578113	124.061783
44.160000	121.333000	44.159260	0.516778	123.982239
44.180000	118.667000	44.179260	0.507649	123.902893
44.200001	116.000000	44.199261	0.555578	123.823776
44.220001	117.667000	44.219261	0.681040	123.744873
44.240002	118.833000	44.239262	0.932383	123.666229
44.260002	124.000000	44.259262	1.413732	123.587830
44.280003	131.333008	44.279263	2.343519	123.509659
44.299999	125.500000	44.299259	4.167718	123.431702
44.320000	134.833008	44.319260	7.769548	123.353973

44.340000	134.333008	44.339260	14.740817	123.276443
44.360001	141.833008	44.359261	27.189245	123.199173
44.380001	168.667007	44.379261	44.693645	123.122131
44.400002	178.833008	44.399261	56.775650	123.045273
44.420002	175.167007	44.419262	49.223915	122.968704
44.440002	160.500000	44.439262	30.132683	122.892334
44.460003	147.500000	44.459267	15.502382	122.816177
44.480003	137.667007	44.479267	7.905210	122.740234
44.500000	123.833000	44.499264	4.331089	122.664551
44.520000	132.667007	44.519264	2.569823	122.589081
44.540001	124.500000	44.539265	1.614313	122.513824
44.560001	127.333000	44.559265	1.050766	122.438751
44.580002	127.167000	44.579266	0.701524	122.363937
44.599998	129.833008	44.599262	0.479695	122.289352
44.619999	124.000000	44.619263	0.336561	122.214951
44.639999	128.833008	44.639263	0.242823	122.140793
44.660000	121.333000	44.659264	0.180376	122.066833
44.680000	120.167000	44.679264	0.138045	121.993103
44.700001	119.500000	44.699265	0.108927	121.919571
44.720001	120.500000	44.719265	0.088730	121.846252
44.740002	126.833000	44.739265	0.074749	121.773148
44.760002	119.667000	44.759266	0.065276	121.700272
44.780003	118.167000	44.779266	0.059194	121.627609
44.799999	124.000000	44.799263	0.055831	121.555161
44.820000	117.833000	44.819263	0.054790	121.482925
44.840000	122.000000	44.839264	0.055893	121.410919
44.860001	120.667000	44.859264	0.059145	121.339035
44.880001	118.833000	44.879265	0.064724	121.267456
44.900002	121.667000	44.899265	0.073003	121.196045
44.920002	122.500000	44.919266	0.084588	121.124832
44.940002	119.667000	44.939266	0.100403	121.053864
44.960003	122.333000	44.959267	0.121814	120.983093
44.980003	115.500000	44.979267	0.150839	120.912506
45.000000	118.000000	44.999264	0.190481	120.842133
45.020000	123.000000	45.019264	0.245308	120.771957
45.040001	117.167000	45.039265	0.322357	120.701981
45.060001	109.333000	45.059265	0.432815	120.632248
45.080002	119.667000	45.079266	0.595098	120.562698

45.099998	118.167000	45.099262	0.840888	120.493378
45.119999	117.500000	45.119263	1.228076	120.424225
45.139999	123.500000	45.139263	1.867903	120.355255
45.160000	120.333000	45.159264	2.983986	120.286514
45.180000	126.167000	45.179264	5.035370	120.217972
45.200001	126.333000	45.199265	8.963989	120.149612
45.220001	142.500000	45.219265	16.662628	120.081467
45.240002	160.000000	45.239265	31.722616	120.013504
45.260002	200.500000	45.259266	59.854752	119.945755
45.280003	256.166992	45.279266	105.045258	119.878204
45.299999	303.832977	45.299263	152.575806	119.810837
45.320000	308.832977	45.319263	158.781693	119.743698
45.340000	261.000000	45.339268	112.876854	119.676697
45.360001	203.833008	45.359268	61.941738	119.609940
45.380001	159.000000	45.379269	31.400606	119.543335
45.400002	136.500000	45.399269	16.560732	119.476959
45.420002	124.000000	45.419270	9.435273	119.410767
45.440002	128.667007	45.439270	5.741468	119.344788
45.460003	125.500000	45.459270	3.647627	119.278961
45.480003	117.833000	45.479271	2.383236	119.213348
45.500000	116.833000	45.499268	1.592819	119.147919
45.520000	117.167000	45.519268	1.088703	119.082672
45.540001	121.333000	45.539268	0.761987	119.017670
45.560001	115.333000	45.559269	0.546344	118.952789
45.580002	119.833000	45.579269	0.401040	118.888092
45.599998	116.667000	45.599266	0.300777	118.823608
45.619999	111.667000	45.619267	0.230491	118.759308
45.639999	116.333000	45.639267	0.180177	118.695221
45.660000	119.000000	45.659267	0.143577	118.631256
45.680000	114.833000	45.679268	0.116596	118.567535
45.700001	112.167000	45.699268	0.096498	118.503998
45.720001	115.500000	45.719269	0.081422	118.440643
45.740002	109.333000	45.739269	0.070081	118.377441
45.760002	115.000000	45.759270	0.061573	118.314423
45.780003	121.667000	45.779270	0.055257	118.251617
45.799999	121.833000	45.799267	0.050678	118.188995
45.820000	124.833000	45.819267	0.047509	118.126526
45.840000	115.000000	45.839268	0.045519	118.064240

45.860001	113.000000	45.859268	0.044548	118.002197
45.880001	110.833000	45.879269	0.044492	117.940277
45.900002	111.500000	45.899265	0.045290	117.878540
45.920002	114.500000	45.919266	0.046919	117.816986
45.940002	119.000000	45.939266	0.049390	117.755585
45.960003	113.500000	45.959267	0.052749	117.694427
45.980003	119.667000	45.979267	0.057074	117.633392
46.000000	113.333000	45.999264	0.062485	117.572540
46.020000	109.833000	46.019264	0.069149	117.511902
46.040001	110.000000	46.039265	0.077287	117.451385
46.060001	119.667000	46.059265	0.087199	117.391083
46.080002	111.333000	46.079266	0.099215	117.330902
46.099998	113.833000	46.099262	0.114002	117.270935
46.119999	115.333000	46.119263	0.132225	117.211182
46.139999	114.000000	46.139263	0.154895	117.151550
46.160000	116.833000	46.159264	0.183451	117.092102
46.180000	119.500000	46.179264	0.220020	117.032837
46.200001	120.667000	46.199265	0.267870	116.973724
46.220001	116.167000	46.219269	0.332337	116.914825
46.240002	118.167000	46.239269	0.422804	116.856049
46.260002	107.333000	46.259270	0.556098	116.797455
46.280003	115.333000	46.279270	0.765529	116.739014
46.299999	107.833000	46.299267	1.116914	116.680756
46.320000	114.333000	46.319267	1.740409	116.622711
46.340000	115.667000	46.339268	2.885124	116.564789
46.360001	120.000000	46.359268	4.971125	116.507019
46.380001	122.167000	46.379269	8.433512	116.449432
46.400002	128.167007	46.399269	12.730075	116.392059
46.420002	132.167007	46.419270	14.964038	116.334808
46.440002	135.333008	46.439270	13.018461	116.277740
46.460003	125.833000	46.459270	9.838681	116.220795
46.480003	128.000000	46.479271	8.459129	116.164062
46.500000	128.333008	46.499268	9.280173	116.107513
46.520000	126.667000	46.519268	12.203032	116.051086
46.540001	134.000000	46.539268	17.867275	115.994812
46.560001	137.000000	46.559269	28.201685	115.938721
46.580002	164.500000	46.579269	47.465683	115.882782
46.599998	187.167007	46.599266	84.519035	115.827057

46.619999	233.000000	46.619267	157.259659	115.771423
46.639999	392.332977	46.639267	299.532288	115.715973
46.660000	653.000000	46.659267	564.337158	115.660675
46.680000	1105.330078	46.679268	985.862610	115.605560
46.700001	1532.669922	46.699268	1424.134888	115.550568
46.720001	1577.830078	46.719269	1483.559326	115.495819
46.740002	1193.500000	46.739269	1066.821777	115.441193
46.760002	718.833008	46.759270	594.190125	115.386688
46.780003	414.500000	46.779270	304.225555	115.332367
46.799999	256.332977	46.799267	160.720718	115.278198
46.820000	186.667007	46.819267	91.056076	115.224182
46.840000	162.167007	46.839268	54.902420	115.170319
46.860001	156.167007	46.859268	34.542923	115.116608
46.880001	152.667007	46.879269	22.365870	115.063110
46.900002	142.167007	46.899269	14.820704	115.009705
46.920002	153.000000	46.919270	10.044437	114.956451
46.940002	146.167007	46.939270	6.966656	114.903412
46.960003	149.167007	46.959270	4.944821	114.850433
46.980003	147.167007	46.979271	3.587922	114.797699
47.000000	140.333008	46.999268	2.656911	114.745087
47.020000	132.667007	47.019268	2.003865	114.692627
47.040001	127.667000	47.039268	1.536550	114.640350
47.060001	116.333000	47.059273	1.195734	114.588135
47.080002	123.667000	47.079273	0.943318	114.536102
47.099998	113.667000	47.099270	0.753454	114.484283
47.119999	114.167000	47.119270	0.608639	114.432587
47.139999	112.000000	47.139271	0.497011	114.381042
47.160000	112.167000	47.159271	0.409771	114.329620
47.180000	114.667000	47.179272	0.341010	114.278412
47.200001	111.167000	47.199272	0.286333	114.227295
47.220001	110.167000	47.219273	0.242521	114.176361
47.240002	107.500000	47.239273	0.207182	114.125549
47.260002	124.667000	47.259274	0.178524	114.074890
47.280003	117.667000	47.279274	0.155192	114.024384
47.299999	115.833000	47.299271	0.136157	113.973969
47.320000	109.167000	47.319271	0.120620	113.923798
47.340000	120.833000	47.339272	0.107978	113.873749
47.360001	115.667000	47.359272	0.097765	113.823822

47.380001	110.833000	47.379272	0.089624	113.774017
47.400002	114.500000	47.399273	0.083290	113.724396
47.420002	115.667000	47.419273	0.078568	113.674896
47.440002	115.667000	47.439274	0.075326	113.625580
47.460003	118.333000	47.459274	0.073491	113.576385
47.480003	111.333000	47.479275	0.073040	113.527313
47.500000	104.500000	47.499271	0.074003	113.478424
47.520000	109.833000	47.519272	0.076470	113.429657
47.540001	116.667000	47.539272	0.080733	113.381042
47.560001	112.000000	47.559273	0.086753	113.332520
47.580002	112.167000	47.579273	0.094989	113.284210
47.599998	109.833000	47.599270	0.105895	113.235992
47.619999	112.667000	47.619270	0.120091	113.187958
47.639999	110.000000	47.639271	0.138418	113.140045
47.660000	119.833000	47.659271	0.162026	113.092255
47.680000	115.500000	47.679272	0.192494	113.044647
47.700001	116.667000	47.699272	0.232012	112.997101
47.720001	109.500000	47.719273	0.283659	112.949799
47.740002	117.167000	47.739273	0.351817	112.902557
47.760002	118.667000	47.759274	0.442825	112.855469
47.780003	108.333000	47.779274	0.566015	112.808563
47.799999	114.667000	47.799271	0.735367	112.761749
47.820000	111.500000	47.819271	0.972465	112.715118
47.840000	116.333000	47.839272	1.311221	112.668549
47.860001	119.000000	47.859272	1.806906	112.622192
47.880001	122.000000	47.879272	2.553256	112.575928
47.900002	126.000000	47.899277	3.717492	112.529816
47.920002	130.333008	47.919277	5.612650	112.483795
47.940002	130.667007	47.939278	8.855572	112.437958
47.960003	145.667007	47.959278	14.698181	112.392242
47.980003	147.167007	47.979279	25.712429	112.346649
48.000000	167.167007	47.999275	47.129658	112.301239
48.020000	209.167007	48.019276	89.203049	112.255951
48.040001	295.666992	48.039276	169.735703	112.210754
48.060001	452.166992	48.059277	309.175018	112.165741
48.080002	664.500000	48.079277	492.683197	112.120819
48.099998	771.000000	48.099274	602.593872	112.076050
48.119999	681.833008	48.119274	513.771545	112.031403

48.139999	460.666992	48.139275	319.154114	111.986908
48.160000	285.666992	48.159271	168.782578	111.942535
48.180000	200.000000	48.179272	87.671082	111.898285
48.200001	150.667007	48.199272	48.030071	111.854156
48.220001	125.333000	48.219273	28.071175	111.810150
48.240002	127.000000	48.239273	17.261332	111.766327
48.260002	130.500000	48.259274	11.006990	111.722565
48.280003	118.833000	48.279274	7.231117	111.678986
48.299999	121.000000	48.299271	4.899219	111.635529
48.320000	121.167000	48.319271	3.443544	111.592194
48.340000	116.333000	48.339272	2.536728	111.548981
48.360001	126.333000	48.359272	1.987503	111.505920
48.380001	121.000000	48.379272	1.688889	111.462952
48.400002	126.167000	48.399273	1.590606	111.420135
48.420002	120.333000	48.419273	1.688178	111.377441
48.440002	119.833000	48.439274	2.028303	111.334869
48.460003	112.333000	48.459274	2.737578	111.292450
48.480003	119.167000	48.479275	4.094039	111.250092
48.500000	120.500000	48.499271	6.682424	111.207916
48.520000	116.833000	48.519272	11.715562	111.165863
48.540001	135.667007	48.539272	21.635836	111.123901
48.560001	157.000000	48.559273	41.037235	111.082123
48.580002	198.500000	48.579273	76.941849	111.040405
48.599998	248.167007	48.599270	133.330154	110.998871
48.619999	311.666992	48.619270	190.994720	110.957428
48.639999	313.666992	48.639271	199.086517	110.916107
48.660000	272.000000	48.659271	145.246933	110.874908
48.680000	201.167007	48.679272	82.527626	110.833862
48.700001	153.333008	48.699272	42.887119	110.792908
48.720001	135.000000	48.719276	22.772680	110.752075
48.740002	120.667000	48.739277	12.865720	110.711365
48.760002	111.167000	48.759277	7.708926	110.670807
48.780003	109.167000	48.779278	4.825821	110.630341
48.799999	114.167000	48.799274	3.123078	110.590027
48.820000	108.667000	48.819275	2.082503	110.549805
48.840000	108.667000	48.839275	1.433753	110.509705
48.860001	107.667000	48.859276	1.023547	110.469757
48.880001	111.167000	48.879276	0.761433	110.429901

48.900002	113.833000	48.899277	0.593238	110.390167
48.920002	111.667000	48.919277	0.486396	110.350555
48.940002	107.833000	48.939278	0.421331	110.311035
48.960003	111.167000	48.959278	0.386371	110.271667
48.980003	102.333000	48.979279	0.374779	110.232422
49.000000	112.000000	48.999275	0.383069	110.193268
49.020000	110.833000	49.019276	0.410119	110.154297
49.040001	109.333000	49.039276	0.456746	110.115387
49.060001	112.500000	49.059277	0.525661	110.076599
49.080002	106.333000	49.079277	0.621728	110.037933
49.099998	110.000000	49.099274	0.752530	109.999420
49.119999	111.000000	49.119274	0.929540	109.960999
49.139999	109.333000	49.139275	1.169716	109.922668
49.160000	110.833000	49.159275	1.498445	109.884430
49.180000	117.000000	49.179276	1.953969	109.846405
49.200001	115.333000	49.199276	2.596273	109.808441
49.220001	108.167000	49.219276	3.519421	109.770569
49.240002	118.333000	49.239277	4.877692	109.732819
49.260002	123.833000	49.259277	6.933662	109.695221
49.280003	120.167000	49.279278	10.155923	109.657684
49.299999	127.333000	49.299274	15.422958	109.620331
49.320000	138.667007	49.319275	24.464640	109.583038
49.340000	148.000000	49.339275	40.782246	109.545898
49.360001	174.000000	49.359276	71.563454	109.508850
49.380001	234.333008	49.379276	131.432800	109.471924
49.400002	369.166992	49.399277	248.902191	109.435089
49.420002	616.666992	49.419277	472.864899	109.398346
49.440002	1040.000000	49.439278	856.955627	109.361755
49.460003	1568.330078	49.459278	1354.065796	109.325287
49.480003	1771.330078	49.479279	1644.697388	109.288940
49.500000	1515.830078	49.499275	1404.796143	109.252655
49.520000	1093.000000	49.519276	881.710999	109.216492
49.540001	642.166992	49.539280	471.582520	109.180481
49.560001	388.000000	49.559280	246.602325	109.144562
49.580002	258.000000	49.579281	135.083618	109.108734
49.599998	185.333008	49.599277	78.510849	109.073029
49.619999	156.333008	49.619278	47.844143	109.037415
49.639999	140.667007	49.639278	30.164358	109.001923

49.660000	128.500000	49.659279	19.526012	108.966553
49.680000	126.500000	49.679279	12.945817	108.931274
49.700001	127.667000	49.699280	8.790346	108.896149
49.720001	118.167000	49.719280	6.113389	108.861084
49.740002	116.500000	49.739281	4.351964	108.826141
49.760002	118.167000	49.759281	3.167004	108.791290
49.780003	113.667000	49.779282	2.352222	108.756561
49.799999	119.167000	49.799278	1.780454	108.721954
49.820000	117.000000	49.819279	1.371562	108.687469
49.840000	120.333000	49.839279	1.074434	108.653015
49.860001	121.000000	49.859280	0.855538	108.618744
49.880001	116.000000	49.879280	0.692507	108.584564
49.900002	112.333000	49.899281	0.570166	108.550476
49.920002	108.000000	49.919281	0.478077	108.516510
49.940002	107.667000	49.939281	0.408872	108.482605
49.960003	106.333000	49.959282	0.357761	108.448853
49.980003	111.667000	49.979282	0.320995	108.415192
50.000000	108.667000	49.999279	0.296570	108.381622
50.020000	111.333000	50.019279	0.282916	108.348175
50.040001	104.333000	50.039280	0.279503	108.314850
50.060001	100.333000	50.059280	0.286202	108.281586
50.080002	108.167000	50.079281	0.303927	108.248444
50.099998	105.000000	50.099277	0.334346	108.215424
50.119999	105.000000	50.119278	0.380243	108.182465
50.139999	109.000000	50.139278	0.445879	108.149658
50.160000	105.667000	50.159279	0.537649	108.116913
50.180000	110.500000	50.179279	0.665113	108.084320
50.200001	108.833000	50.199280	0.842663	108.051788
50.220001	116.833000	50.219280	1.092287	108.019348
50.240002	111.500000	50.239281	1.448260	107.987030
50.260002	121.500000	50.259281	1.965402	107.954834
50.280003	116.833000	50.279282	2.734293	107.922729
50.299999	119.500000	50.299278	3.910514	107.890717
50.320000	118.500000	50.319279	5.775635	107.858795
50.340000	119.667000	50.339283	8.863025	107.826996
50.360001	128.167007	50.359283	14.224803	107.795258
50.380001	140.000000	50.379284	24.001905	107.763641
50.400002	147.333008	50.399284	42.584705	107.732147

50.420002	189.500000	50.419285	78.851532	107.700745
50.440002	261.666992	50.439285	149.770416	107.669403
50.460003	410.832977	50.459286	282.721252	107.638214
50.480003	615.500000	50.479286	500.645874	107.607086
50.500000	864.000000	50.499283	753.937866	107.576080
50.520000	957.500000	50.519283	854.214722	107.545166
50.540001	792.333008	50.539280	683.048767	107.514343
50.560001	547.166992	50.559280	413.521240	107.483643
50.580002	348.666992	50.579281	220.081863	107.453003
50.599998	227.000000	50.599277	116.316994	107.422485
50.619999	182.833008	50.619278	64.485710	107.392059
50.639999	140.000000	50.639278	37.760990	107.361725
50.660000	120.167000	50.659279	23.091696	107.331512
50.680000	129.833008	50.679279	14.583235	107.301361
50.700001	115.333000	50.699280	9.457115	107.271332
50.720001	117.000000	50.719280	6.288868	107.241394
50.740002	117.667000	50.739281	4.290564	107.211517
50.760002	117.833000	50.759281	3.005395	107.181763
50.780003	107.667000	50.779282	2.162429	107.152130
50.799999	111.500000	50.799278	1.599175	107.122559
50.820000	113.333000	50.819279	1.216886	107.093109
50.840000	106.333000	50.839279	0.955253	107.063690
50.860001	107.833000	50.859280	0.776795	107.034424
50.880001	102.667000	50.879280	0.658188	107.005219
50.900002	107.667000	50.899281	0.585172	106.976166
50.920002	106.833000	50.919281	0.549670	106.947174
50.940002	101.833000	50.939281	0.548265	106.918243
50.960003	108.000000	50.959282	0.581633	106.889435
50.980003	107.500000	50.979282	0.654760	106.860687
51.000000	109.333000	50.999279	0.778121	106.832092
51.020000	105.167000	51.019279	0.969781	106.803558
51.040001	101.667000	51.039280	1.260230	106.775116
51.060001	103.167000	51.059280	1.700408	106.746765
51.080002	106.333000	51.079281	2.377747	106.718536
51.099998	107.667000	51.099277	3.448015	106.690338
51.119999	115.500000	51.119282	5.203463	106.662262
51.139999	116.833000	51.139282	8.212111	106.634308
51.160000	126.667000	51.159283	13.620903	106.606384

51.180000	127.833000	51.179283	23.778444	106.578583
51.200001	156.500000	51.199284	43.468628	106.550873
51.220001	190.167007	51.219284	82.053123	106.523224
51.240002	290.332977	51.239285	155.752838	106.495697
51.260002	430.500000	51.259285	283.377808	106.468262
51.280003	613.000000	51.279285	454.502472	106.440887
51.299999	705.666992	51.299282	571.160095	106.413635
51.320000	685.500000	51.319283	512.986755	106.386475
51.340000	506.000000	51.339283	337.535004	106.359375
51.360001	331.332977	51.359283	185.723358	106.332367
51.380001	229.000000	51.379284	97.922485	106.305450
51.400002	176.833008	51.399284	53.370125	106.278656
51.420002	134.833008	51.419285	30.673641	106.251862
51.440002	129.333008	51.439285	18.464853	106.225220
51.460003	122.333000	51.459286	11.512901	106.198700
51.480003	117.667000	51.479286	7.383815	106.172211
51.500000	111.333000	51.499283	4.860519	106.145782
51.520000	109.667000	51.519283	3.284380	106.119507
51.540001	112.667000	51.539284	2.280780	106.093292
51.560001	104.333000	51.559284	1.629396	106.067169
51.580002	110.667000	51.579285	1.198964	106.041107
51.599998	104.000000	51.599281	0.910511	106.015137
51.619999	109.167000	51.619282	0.715859	105.989258
51.639999	102.000000	51.639282	0.585533	105.963470
51.660000	105.500000	51.659283	0.501347	105.937775
51.680000	104.000000	51.679283	0.452329	105.912140
51.700001	107.833000	51.699284	0.432379	105.886627
51.720001	102.000000	51.719284	0.439043	105.861145
51.740002	109.667000	51.739285	0.473020	105.835754
51.760002	106.667000	51.759285	0.538282	105.810486
51.780003	109.667000	51.779285	0.642840	105.785309
51.799999	107.333000	51.799282	0.800323	105.760162
51.820000	105.667000	51.819283	1.033094	105.735138
51.840000	113.000000	51.839283	1.377449	105.710175
51.860001	110.333000	51.859283	1.893486	105.685333
51.880001	106.000000	51.879284	2.684723	105.660492
51.900002	108.833000	51.899288	3.935677	105.635773
51.920002	111.500000	51.919289	5.990945	105.611176

51.940002	112.500000	51.939289	9.524731	105.586609
51.960003	120.333000	51.959290	15.896053	105.562164
51.980003	122.500000	51.979290	27.885874	105.537750
52.000000	152.333008	51.999287	51.134640	105.513458
52.020000	188.333008	52.019287	96.594933	105.489227
52.040001	295.000000	52.039288	182.731247	105.465088
52.060001	469.666992	52.059288	329.036743	105.441010
52.080002	676.833008	52.079288	517.713684	105.417023
52.099998	763.833008	52.099285	636.201294	105.393127
52.119999	718.333008	52.119286	566.020081	105.369293
52.139999	497.166992	52.139286	376.692444	105.345551
52.160000	331.332977	52.159286	211.039810	105.321899
52.180000	230.333008	52.179287	112.441216	105.298279
52.200001	168.667007	52.199287	61.385933	105.274780
52.220001	132.000000	52.219288	35.169407	105.251343
52.240002	125.000000	52.239288	21.068695	105.227997
52.260002	120.000000	52.259289	13.068961	105.204712
52.280003	113.333000	52.279289	8.337747	105.181488
52.299999	115.833000	52.299286	5.456367	105.158386
52.320000	104.333000	52.319286	3.660089	105.135345
52.340000	115.667000	52.339287	2.516449	105.112335
52.360001	106.500000	52.359287	1.772293	105.089447
52.380001	107.667000	52.379288	1.277156	105.066650
52.400002	108.000000	52.399288	0.940410	105.043884
52.420002	105.167000	52.419289	0.706639	105.021210
52.440002	101.833000	52.439289	0.541339	104.998627
52.460003	107.667000	52.459290	0.422590	104.976105
52.480003	108.000000	52.479290	0.336073	104.953644
52.500000	110.333000	52.499287	0.272521	104.931305
52.520000	101.833000	52.519287	0.225722	104.908966
52.540001	105.167000	52.539288	0.191286	104.886749
52.560001	110.667000	52.559288	0.166276	104.864594
52.580002	106.667000	52.579288	0.148689	104.842560
52.599998	101.833000	52.599285	0.137183	104.820557
52.619999	99.333298	52.619286	0.130907	104.798615
52.639999	106.333000	52.639286	0.129420	104.776733
52.660000	106.000000	52.659290	0.132579	104.754974
52.680000	111.833000	52.679291	0.140598	104.733246

52.700001	103.333000	52.699291	0.154026	104.711609
52.720001	106.167000	52.719292	0.173815	104.690033
52.740002	102.000000	52.739292	0.201440	104.668549
52.760002	102.167000	52.759293	0.239073	104.647125
52.780003	110.000000	52.779293	0.289877	104.625763
52.799999	103.667000	52.799290	0.358434	104.604492
52.820000	104.167000	52.819290	0.451516	104.583282
52.840000	102.833000	52.839291	0.579140	104.562103
52.860001	106.667000	52.859291	0.756487	104.541046
52.880001	106.333000	52.879292	1.007040	104.520050
52.900002	110.500000	52.899292	1.368080	104.499115
52.920002	119.000000	52.919292	1.900697	104.478241
52.940002	124.667000	52.939293	2.708786	104.457428
52.960003	121.833000	52.959293	3.976722	104.436737
52.980003	144.833008	52.979294	6.046420	104.416046
53.000000	143.000000	52.999290	9.576758	104.395477
53.020000	151.333008	53.019291	15.877147	104.374969
53.040001	153.500000	53.039291	27.555952	104.354523
53.060001	186.833008	53.059288	49.654327	104.334106
53.080002	235.000000	53.079288	91.103386	104.313812
53.099998	331.832977	53.099285	165.224442	104.293549
53.119999	442.832977	53.119286	288.364166	104.273346
53.139999	607.833008	53.139286	472.056244	104.253235
53.160000	696.500000	53.159286	675.088013	104.233215
53.180000	706.833008	53.179287	748.652771	104.213196
53.200001	648.833008	53.199287	601.929688	104.193268
53.220001	524.666992	53.219288	372.300323	104.173431
53.240002	364.500000	53.239288	202.594620	104.153656
53.260002	248.833008	53.259289	108.520844	104.133911
53.280003	191.333008	53.279289	60.326721	104.114258
53.299999	144.000000	53.299286	35.138268	104.094666
53.320000	125.333000	53.319286	21.285145	104.075134
53.340000	114.000000	53.339287	13.300238	104.055664
53.360001	112.500000	53.359287	8.533683	104.036255
53.380001	110.667000	53.379288	5.613445	104.016907
53.400002	110.333000	53.399288	3.784246	103.997650
53.420002	112.000000	53.419292	2.613018	103.978424
53.440002	110.667000	53.439293	1.846290	103.959259

53.460003	103.667000	53.459293	1.332641	103.940186
53.480003	106.500000	53.479294	0.980783	103.921143
53.500000	107.833000	53.499290	0.734678	103.902191
53.520000	106.833000	53.519291	0.559110	103.883270
53.540001	112.667000	53.539291	0.431660	103.864441
53.560001	102.667000	53.559292	0.337642	103.845673
53.580002	109.333000	53.579292	0.267266	103.826935
53.599998	108.667000	53.599289	0.213892	103.808289
53.619999	118.500000	53.619289	0.172902	103.789703
53.639999	112.500000	53.639290	0.141080	103.771179
53.660000	111.333000	53.659290	0.116124	103.752716
53.680000	102.667000	53.679291	0.096372	103.734283
53.700001	102.000000	53.699291	0.080609	103.715912
53.720001	101.000000	53.719292	0.067935	103.697632
53.740002	106.833000	53.739292	0.057678	103.679382
53.760002	103.833000	53.759293	0.049332	103.661194
53.780003	103.667000	53.779293	0.042511	103.643066
53.799999	97.500000	53.799290	0.036923	103.625031
53.820000	96.666695	53.819290	0.032339	103.607025
53.840000	97.500000	53.839291	0.028587	103.589050
53.860001	99.833298	53.859291	0.025535	103.571167
53.880001	100.833000	53.879292	0.023085	103.553314
53.900002	98.333298	53.899292	0.021211	103.535553
53.920002	102.167000	53.919292	0.019757	103.517853
53.940002	94.333298	53.939293	0.018782	103.500183
53.960003	104.000000	53.959293	0.018264	103.482574
53.980003	95.833298	53.979294	0.018224	103.465027
54.000000	94.500000	53.999290	0.018715	103.447510
54.020000	96.333298	54.019291	0.019827	103.430084
54.040001	90.333298	54.039291	0.021700	103.412689
54.060001	100.667000	54.059292	0.024544	103.395355
54.080002	95.500000	54.079292	0.028675	103.378082
54.099998	95.000000	54.099289	0.034561	103.360870
54.119999	101.000000	54.119289	0.042910	103.343719
54.139999	99.500000	54.139290	0.054799	103.326569
54.160000	98.833298	54.159294	0.071914	103.309509
54.180000	98.000000	54.179295	0.096931	103.292511
54.200001	98.166695	54.199295	0.134163	103.275543

54.220001	99.500000	54.219296	0.191315	103.258636
54.240002	97.166695	54.239296	0.281617	103.241791
54.260002	106.333000	54.259296	0.429843	103.225006
54.280003	99.666695	54.279297	0.684051	103.208252
54.299999	99.833298	54.299294	1.140492	103.191559
54.320000	99.666695	54.319294	1.995929	103.174896
54.340000	103.500000	54.339294	3.649120	103.158356
54.360001	107.167000	54.359295	6.872915	103.141815
54.380001	120.667000	54.379295	12.973438	103.125336
54.400002	133.500000	54.399296	23.367241	103.108856
54.420002	148.000000	54.419296	37.027119	103.092499
54.440002	146.667007	54.439297	46.336075	103.076141
54.460003	145.167007	54.459297	42.226875	103.059845
54.480003	136.667007	54.479298	28.630293	103.043610
54.500000	120.833000	54.499294	16.240332	103.027435
54.520000	116.000000	54.519295	8.731065	103.011292
54.540001	112.000000	54.539295	4.786731	102.995209
54.560001	109.333000	54.559296	2.737796	102.979187
54.580002	102.000000	54.579296	1.630234	102.963165
54.599998	98.666695	54.599293	1.003225	102.947205
54.619999	95.666695	54.619293	0.634669	102.931335
54.639999	96.166695	54.639294	0.411953	102.915466
54.660000	99.166695	54.659294	0.274221	102.899689
54.680000	90.833298	54.679295	0.187152	102.883881
54.700001	98.333298	54.699295	0.130860	102.868164
54.720001	99.166695	54.719296	0.093626	102.852539
54.740002	103.000000	54.739296	0.068438	102.836914
54.760002	97.000000	54.759296	0.051032	102.821320
54.780003	98.333298	54.779297	0.038768	102.805786
54.799999	98.666695	54.799294	0.029975	102.790314
54.820000	93.833298	54.819294	0.023569	102.774872
54.840000	92.666695	54.839294	0.018840	102.759491
54.860001	96.833298	54.859295	0.015309	102.744141
54.880001	97.333298	54.879295	0.012649	102.728821
54.900002	92.500000	54.899300	0.010629	102.713562
54.920002	94.500000	54.919300	0.009089	102.698334
54.940002	100.167000	54.939301	0.007927	102.683197
54.960003	100.333000	54.959301	0.007053	102.668030

54.980003	94.166695	54.979301	0.006408	102.652954
55.000000	101.500000	54.999298	0.005950	102.637909
55.020000	97.833298	55.019299	0.005648	102.622925
55.040001	93.666695	55.039299	0.005480	102.607941
55.060001	99.333298	55.059299	0.005433	102.593018
55.080002	99.000000	55.079300	0.005498	102.578156
55.099998	98.666695	55.099297	0.005673	102.563293
55.119999	98.333298	55.119297	0.005957	102.548523
55.139999	103.167000	55.139297	0.006355	102.533783
55.160000	101.000000	55.159298	0.006879	102.519073
55.180000	94.500000	55.179298	0.007541	102.504364
55.200001	99.000000	55.199299	0.008361	102.489746
55.220001	98.000000	55.219299	0.009366	102.475128
55.240002	101.667000	55.239300	0.010589	102.460571
55.260002	91.500000	55.259300	0.012093	102.446075
55.280003	98.166695	55.279301	0.013911	102.431610
55.299999	99.833298	55.299297	0.016105	102.417175
55.320000	97.500000	55.319298	0.018782	102.402771
55.340000	91.500000	55.339298	0.022062	102.388397
55.360001	95.833298	55.359299	0.026103	102.374084
55.380001	95.666695	55.379299	0.031025	102.359772
55.400002	101.500000	55.399300	0.037280	102.345551
55.420002	94.666695	55.419300	0.045147	102.331329
55.440002	95.166695	55.439301	0.055127	102.317169
55.460003	99.833298	55.459301	0.067906	102.303009
55.480003	101.167000	55.479301	0.084439	102.288910
55.500000	93.833298	55.499298	0.106064	102.274872
55.520000	95.666695	55.519299	0.134707	102.260834
55.540001	95.166695	55.539299	0.173151	102.246857
55.560001	90.166695	55.559299	0.225507	102.232849
55.580002	91.500000	55.579300	0.297970	102.218933
55.599998	99.666695	55.599297	0.400036	102.205078
55.619999	101.667000	55.619297	0.546732	102.191223
55.639999	90.666695	55.639301	0.762346	102.177368
55.660000	96.500000	55.659302	1.087358	102.163605
55.680000	93.000000	55.679302	1.592267	102.149841
55.700001	100.333000	55.699303	2.404898	102.136108
55.720001	98.000000	55.719299	3.767248	102.122437

55.740002	105.000000	55.739300	6.157303	102.108795
55.760002	104.667000	55.759300	10.541080	102.095154
55.780003	110.167000	55.779301	18.887131	102.081573
55.799999	131.667007	55.799297	35.105804	102.068024
55.820000	163.000000	55.819298	66.347878	102.054474
55.840000	231.167007	55.839298	122.794899	102.041016
55.860001	308.000000	55.859299	208.784012	102.027527
55.880001	410.500000	55.879299	296.610931	102.014099
55.900002	419.166992	55.899300	318.785675	102.000732
55.920002	361.000000	55.919300	250.497299	101.987335
55.940002	255.333008	55.939301	154.835373	101.973999
55.960003	194.500000	55.959301	85.419395	101.960693
55.980003	151.333008	55.979301	46.434834	101.947418
56.000000	123.667000	55.999298	26.026026	101.934174
56.020000	119.500000	56.019299	15.167738	101.920929
56.040001	109.500000	56.039299	9.150241	101.907745
56.060001	106.333000	56.059299	5.683844	101.894562
56.080002	97.833298	56.079300	3.625935	101.881439
56.099998	104.667000	56.099297	2.374802	101.868347
56.119999	107.167000	56.119297	1.597733	101.855286
56.139999	109.500000	56.139297	1.105968	101.842194
56.160000	101.500000	56.159298	0.790247	101.829193
56.180000	103.500000	56.179298	0.587380	101.816193
56.200001	101.000000	56.199299	0.462112	101.803223
56.220001	99.833298	56.219299	0.398214	101.790283
56.240002	98.166695	56.239300	0.396475	101.777374
56.260002	100.167000	56.259300	0.479381	101.764465
56.280003	101.500000	56.279301	0.703961	101.751617
56.299999	97.833298	56.299297	1.179243	101.738770
56.320000	96.833298	56.319298	2.055000	101.725983
56.340000	97.000000	56.339298	3.361808	101.713165
56.360001	104.667000	56.359303	4.605788	101.700409
56.380001	102.500000	56.379303	4.772328	101.687653
56.400002	103.833000	56.399303	3.648650	101.674957
56.420002	102.333000	56.419304	2.233230	101.662262
56.440002	99.333298	56.439304	1.241022	101.649597
56.460003	97.000000	56.459305	0.687845	101.636963
56.480003	96.000000	56.479305	0.396660	101.624359

56.500000	90.333298	56.499302	0.240485	101.611725
56.520000	97.833298	56.519302	0.153440	101.599182
56.540001	97.166695	56.539303	0.103415	101.586609
56.560001	97.000000	56.559303	0.074241	101.574097
56.580002	96.000000	56.579304	0.057391	101.561584
56.599998	98.833298	56.599300	0.048221	101.549103
56.619999	104.333000	56.619301	0.044207	101.536621
56.639999	93.833298	56.639301	0.044085	101.524170
56.660000	92.666695	56.659302	0.047407	101.511749
56.680000	92.833298	56.679302	0.054385	101.499359
56.700001	94.833298	56.699303	0.065923	101.487000
56.720001	100.500000	56.719303	0.083908	101.474640
56.740002	95.666695	56.739304	0.111907	101.462311
56.760002	87.666695	56.759304	0.156672	101.449982
56.780003	93.833298	56.779305	0.231314	101.437653
56.799999	92.666695	56.799301	0.361741	101.425415
56.820000	91.833298	56.819302	0.599026	101.413147
56.840000	94.333298	56.839302	1.038207	101.400909
56.860001	94.166695	56.859303	1.828758	101.388672
56.880001	106.000000	56.879303	3.100661	101.376465
56.900002	105.833000	56.899303	4.658350	101.364288
56.920002	109.167000	56.919304	5.668978	101.352081
56.940002	102.667000	56.939304	5.464123	101.339935
56.960003	97.500000	56.959305	4.765300	101.327820
56.980003	102.333000	56.979305	4.796698	101.315674
57.000000	104.333000	56.999302	6.307483	101.303619
57.020000	107.667000	57.019302	10.135237	101.291504
57.040001	111.667000	57.039303	18.082489	101.279449
57.060001	133.000000	57.059307	33.712662	101.267365
57.080002	168.000000	57.079308	62.533859	101.255371
57.099998	217.000000	57.099304	108.451683	101.243317
57.119999	274.332977	57.119305	161.020279	101.231323
57.139999	294.666992	57.139305	185.225616	101.219330
57.160000	281.166992	57.159306	156.652985	101.207367
57.180000	209.000000	57.179306	102.222412	101.195404
57.200001	164.333008	57.199306	57.869339	101.183441
57.220001	140.167007	57.219307	31.610407	101.171509
57.240002	120.167000	57.239307	17.618727	101.159607

57.260002	108.333000	57.259308	10.176266	101.147675
57.280003	106.000000	57.279308	6.079460	101.135834
57.299999	103.667000	57.299305	3.739686	101.123932
57.320000	91.833298	57.319305	2.361802	101.112061
57.340000	105.000000	57.339306	1.530258	101.100220
57.360001	96.333298	57.359306	1.016989	101.088379
57.380001	101.500000	57.379307	0.692976	101.076569
57.400002	99.500000	57.399307	0.483686	101.064728
57.420002	99.166695	57.419308	0.345372	101.052948
57.440002	94.333298	57.439308	0.251936	101.041138
57.460003	95.000000	57.459309	0.187530	101.029327
57.480003	94.500000	57.479309	0.142335	101.017609
57.500000	97.000000	57.499306	0.110148	101.005829
57.520000	99.666695	57.519306	0.086956	100.994080
57.540001	103.167000	57.539307	0.070139	100.982361
57.560001	95.833298	57.559307	0.057955	100.970642
57.580002	86.166695	57.579308	0.049238	100.958893
57.599998	93.000000	57.599304	0.043219	100.947174
57.619999	93.333298	57.619305	0.039402	100.935516
57.639999	97.500000	57.639305	0.037508	100.923828
57.660000	90.666695	57.659306	0.037432	100.912109
57.680000	99.000000	57.679306	0.039236	100.900452
57.700001	91.500000	57.699306	0.043166	100.888794
57.720001	92.166695	57.719307	0.049696	100.877136
57.740002	97.333298	57.739307	0.059614	100.865509
57.760002	94.000000	57.759312	0.074177	100.853821
57.780003	89.833298	57.779312	0.095351	100.842224
57.799999	93.833298	57.799309	0.126260	100.830597
57.820000	93.666695	57.819309	0.171982	100.819000
57.840000	95.500000	57.839310	0.240924	100.807373
57.860001	94.833298	57.859310	0.347550	100.795746
57.880001	98.333298	57.879311	0.517725	100.784180
57.900002	98.166695	57.899311	0.799734	100.772583
57.920002	99.833298	57.919312	1.287367	100.761017
57.940002	95.500000	57.939312	2.168685	100.749420
57.960003	99.333298	57.959312	3.824863	100.737854
57.980003	112.500000	57.979313	7.015486	100.726288
58.000000	114.333000	57.999310	13.146402	100.714752

58.020000	134.000000	58.019310	24.343760	100.703186
58.040001	145.333008	58.039310	42.092026	100.691620
58.060001	179.333008	58.059311	62.541309	100.680084
58.080002	187.500000	58.079311	73.085968	100.668549
58.099998	176.333008	58.099308	65.134743	100.657013
58.119999	155.333008	58.119308	47.651726	100.645447
58.139999	136.167007	58.139309	31.769682	100.633942
58.160000	124.667000	58.159309	20.019049	100.622406
58.180000	122.667000	58.179310	12.267793	100.610870
58.200001	110.500000	58.199310	8.050044	100.599365
58.220001	99.833298	58.219311	6.583160	100.587860
58.240002	97.833298	58.239311	7.518418	100.576324
58.260002	108.000000	58.259312	11.303523	100.564819
58.280003	121.167000	58.279312	18.874294	100.553314
58.299999	133.500000	58.299309	29.943914	100.541809
58.320000	144.833008	58.319309	39.650154	100.530273
58.340000	147.500000	58.339310	39.811649	100.518768
58.360001	132.167007	58.359310	29.907986	100.507294
58.380001	121.500000	58.379311	18.267405	100.495789
58.400002	106.667000	58.399311	10.195652	100.484283
58.420002	100.333000	58.419312	5.653771	100.472809
58.440002	95.666695	58.439312	3.229990	100.461304
58.460003	95.166695	58.459316	1.917273	100.449799
58.480003	89.500000	58.479317	1.184859	100.438293
58.500000	92.666695	58.499313	0.767290	100.426788
58.520000	95.000000	58.519314	0.529241	100.415314
58.540001	94.666695	58.539314	0.400168	100.403778
58.560001	93.333298	58.559315	0.344210	100.392334
58.580002	90.833298	58.579311	0.347048	100.380798
58.599998	99.166695	58.599308	0.411636	100.369324
58.619999	92.000000	58.619308	0.561318	100.357819
58.639999	98.000000	58.639309	0.852768	100.346344
58.660000	92.666695	58.659309	1.406946	100.334808
58.680000	98.333298	58.679310	2.473261	100.323303
58.700001	95.833298	58.699310	4.546194	100.311798
58.720001	105.667000	58.719311	8.515801	100.300293
58.740002	106.000000	58.739311	15.613312	100.288757
58.760002	131.333008	58.759312	26.297466	100.277252

58.780003	132.333008	58.779312	37.217934	100.265717
58.799999	136.667007	58.799309	40.424068	100.254211
58.820000	136.333008	58.819309	32.615925	100.242676
58.840000	116.833000	58.839310	20.816013	100.231201
58.860001	110.667000	58.859310	11.785285	100.219666
58.880001	106.167000	58.879311	6.503819	100.208130
58.900002	95.666695	58.899311	3.663177	100.196594
58.920002	96.500000	58.919312	2.130432	100.185028
58.940002	99.333298	58.939312	1.277413	100.173492
58.960003	92.333298	58.959312	0.787290	100.161957
58.980003	87.166695	58.979313	0.498075	100.150391
59.000000	91.833298	58.999310	0.323458	100.138824
59.020000	91.833298	59.019310	0.215622	100.127289
59.040001	98.500000	59.039310	0.147505	100.115692
59.060001	93.666695	59.059311	0.103473	100.104126
59.080002	94.666695	59.079311	0.074373	100.092590
59.099998	94.666695	59.099308	0.054722	100.080994
59.119999	100.833000	59.119308	0.041189	100.069427
59.139999	98.000000	59.139313	0.031715	100.057831
59.160000	99.833298	59.159313	0.024999	100.046204
59.180000	97.833298	59.179314	0.020196	100.034607
59.200001	94.833298	59.199314	0.016751	100.022980
59.220001	104.000000	59.219315	0.014296	100.011383
59.240002	104.167000	59.239315	0.012582	99.999756
59.260002	101.500000	59.259315	0.011441	99.988129
59.280003	100.833000	59.279316	0.010761	99.976501
59.299999	100.333000	59.299313	0.010470	99.964844
59.320000	95.166695	59.319313	0.010527	99.953186
59.340000	103.833000	59.339314	0.010912	99.941559
59.360001	96.333298	59.359314	0.011629	99.929871
59.380001	98.833298	59.379314	0.012699	99.918152
59.400002	100.833000	59.399315	0.014166	99.906525
59.420002	97.833298	59.419315	0.016094	99.894836
59.440002	102.333000	59.439316	0.018578	99.883118
59.460003	106.667000	59.459316	0.021749	99.871399
59.480003	104.667000	59.479317	0.025785	99.859680
59.500000	107.667000	59.499313	0.030927	99.847961
59.520000	103.000000	59.519314	0.037512	99.836273

59.540001	101.833000	59.539314	0.045998	99.824493
59.560001	99.000000	59.559315	0.057023	99.812744
59.580002	111.000000	59.579315	0.071488	99.800995
59.599998	108.500000	59.599312	0.090678	99.789246
59.619999	98.333298	59.619312	0.116464	99.777435
59.639999	101.167000	59.639313	0.151607	99.765656
59.660000	99.666695	59.659313	0.200212	99.753876
59.680000	99.666695	59.679314	0.268610	99.742035
59.700001	106.500000	59.699314	0.366707	99.730255
59.720001	103.333000	59.719315	0.510347	99.718414
59.740002	102.500000	59.739315	0.725715	99.706573
59.760002	101.500000	59.759315	1.057515	99.694733
59.780003	102.833000	59.779316	1.585027	99.682892
59.799999	98.166695	59.799313	2.454706	99.671021
59.820000	105.333000	59.819317	3.949909	99.659119
59.840000	104.333000	59.839317	6.633172	99.647247
59.860001	114.000000	59.859318	11.644580	99.635345
59.880001	123.333000	59.879318	21.262365	99.623444
59.900002	149.833008	59.899319	39.760151	99.611511
59.920002	182.333008	59.919319	73.856689	99.599548
59.940002	241.833008	59.939320	129.431259	99.587616
59.960003	297.832977	59.959320	198.035507	99.575653
59.980003	344.666992	59.979321	241.267166	99.563690
60.000000	319.166992	59.999317	220.208679	99.551727
60.020000	281.666992	60.019318	154.157272	99.539734
60.040001	204.000000	60.039318	91.361847	99.527710
60.060001	160.500000	60.059319	50.959564	99.515686
60.080002	140.000000	60.079319	28.541630	99.503662
60.099998	118.667000	60.099316	16.444317	99.491638
60.119999	110.667000	60.119316	9.787992	99.479584
60.139999	97.833298	60.139317	6.028336	99.467499
60.160000	110.167000	60.159317	3.865638	99.455444
60.180000	99.833298	60.179317	2.618889	99.443359
60.200001	100.333000	60.199318	1.923527	99.431213
60.220001	94.833298	60.219318	1.588461	99.419098
60.240002	96.666695	60.239319	1.528506	99.406952
60.260002	96.333298	60.259319	1.738077	99.394836
60.280003	98.333298	60.279320	2.297060	99.382660

60.299999	103.667000	60.299316	3.416588	99.370483
60.320000	114.667000	60.319317	5.555301	99.358307
60.340000	106.833000	60.339317	9.661222	99.346100
60.360001	123.000000	60.359318	17.619799	99.333923
60.380001	138.833008	60.379318	32.856770	99.321686
60.400002	160.667007	60.399319	60.285942	99.309418
60.420002	202.333008	60.419319	102.567101	99.297180
60.440002	251.000000	60.439320	148.865433	99.284912
60.460003	261.332977	60.459320	168.689804	99.272614
60.480003	257.000000	60.479321	143.116287	99.260315
60.500000	202.167007	60.499321	95.250603	99.247986
60.520000	173.500000	60.519321	55.290710	99.235687
60.540001	141.500000	60.539322	30.829857	99.223358
60.560001	115.000000	60.559322	17.402729	99.210968
60.580002	118.667000	60.579323	10.123026	99.198608
60.599998	110.500000	60.599319	6.104616	99.186218
60.619999	99.166695	60.619320	3.866290	99.173828
60.639999	90.833298	60.639320	2.672377	99.161407
60.660000	101.333000	60.659321	2.167084	99.148987
60.680000	95.666695	60.679321	2.164167	99.136536
60.700001	93.500000	60.699322	2.434919	99.124054
60.720001	105.167000	60.719322	2.553275	99.111603
60.740002	98.833298	60.739323	2.181888	99.099091
60.760002	95.500000	60.759323	1.512148	99.086609
60.780003	96.833298	60.779324	0.926985	99.074036
60.799999	92.666695	60.799320	0.552398	99.061523
60.820000	93.166695	60.819321	0.337657	99.048981
60.840000	94.500000	60.839321	0.215461	99.036407
60.860001	96.000000	60.859322	0.143776	99.023834
60.880001	105.167000	60.879322	0.100184	99.011200
60.900002	99.666695	60.899323	0.072830	98.998566
60.920002	95.000000	60.919323	0.055220	98.985962
60.940002	104.833000	60.939323	0.043663	98.973328
60.960003	93.833298	60.959324	0.036006	98.960663
60.980003	99.666695	60.979324	0.030971	98.947968
61.000000	100.333000	60.999321	0.027792	98.935272
61.020000	95.833298	61.019321	0.026013	98.922546
61.040001	89.500000	61.039322	0.025370	98.909821

61.060001	87.833298	61.059322	0.025727	98.897064
61.080002	99.833298	61.079323	0.027046	98.884308
61.099998	91.833298	61.099319	0.029364	98.871552
61.119999	96.333298	61.119320	0.032794	98.858765
61.139999	86.333298	61.139320	0.037532	98.845917
61.160000	92.666695	61.159325	0.043875	98.833130
61.180000	93.000000	61.179325	0.052245	98.820251
61.200001	91.166695	61.199326	0.063247	98.807373
61.220001	89.333298	61.219326	0.077733	98.794525
61.240002	89.166695	61.239326	0.096917	98.781616
61.260002	99.500000	61.259327	0.122541	98.768738
61.280003	99.833298	61.279327	0.157145	98.755798
61.299999	96.666695	61.299324	0.204477	98.742828
61.320000	91.000000	61.319324	0.270216	98.729889
61.340000	85.833298	61.339325	0.363057	98.716858
61.360001	98.000000	61.359325	0.496687	98.703888
61.380001	92.833298	61.379326	0.693185	98.690887
61.400002	99.666695	61.399326	0.989251	98.677856
61.420002	99.833298	61.419327	1.448059	98.664795
61.440002	95.166695	61.439327	2.182739	98.651703
61.460003	101.833000	61.459328	3.404469	98.638641
61.480003	100.500000	61.479328	5.522496	98.625580
61.500000	109.667000	61.499325	9.348803	98.612457
61.520000	125.667000	61.519325	16.500660	98.599304
61.540001	130.333008	61.539326	30.061861	98.586151
61.560001	162.333008	61.559326	55.243969	98.572968
61.580002	212.500000	61.579327	98.373985	98.559784
61.599998	283.332977	61.599323	160.299088	98.546600
61.619999	353.332977	61.619324	225.752884	98.533386
61.639999	395.500000	61.639324	270.913757	98.520172
61.660000	412.832977	61.659325	288.377777	98.506866
61.680000	388.332977	61.679325	272.795105	98.493622
61.700001	330.666992	61.699322	216.137360	98.480316
61.720001	284.666992	61.719322	142.342041	98.467010
61.740002	224.500000	61.739323	83.511208	98.453705
61.760002	172.333008	61.759323	47.250053	98.440399
61.780003	143.333008	61.779324	26.990360	98.427002
61.799999	129.333008	61.799320	15.804433	98.413635

61.820000	116.667000	61.819324	9.499419	98.400238
61.840000	109.667000	61.839325	5.857169	98.386841
61.860001	103.667000	61.859325	3.703443	98.373413
61.880001	103.333000	61.879326	2.402013	98.359955
61.900002	98.833298	61.899326	1.598078	98.346497
61.920002	101.833000	61.919327	1.089855	98.333038
61.940002	96.500000	61.939327	0.760842	98.319519
61.960003	105.500000	61.959328	0.542771	98.306030
61.980003	106.000000	61.979328	0.394947	98.292511
62.000000	98.500000	61.999325	0.292638	98.278961
62.020000	95.000000	62.019325	0.220434	98.265381
62.040001	98.833298	62.039326	0.168575	98.251801
62.060001	93.500000	62.059326	0.130789	98.238220
62.080002	97.166695	62.079327	0.102834	98.224579
62.099998	93.500000	62.099323	0.081905	98.210968
62.119999	100.000000	62.119324	0.066059	98.197296
62.139999	94.000000	62.139324	0.053955	98.183655
62.160000	98.333298	62.159325	0.044641	98.169952
62.180000	92.333298	62.179325	0.037439	98.156250
62.200001	98.000000	62.199326	0.031858	98.142548
62.220001	103.333000	62.219326	0.027545	98.128815
62.240002	100.667000	62.239326	0.024234	98.115051
62.260002	95.500000	62.259327	0.021738	98.101288
62.280003	97.500000	62.279327	0.019920	98.087494
62.299999	94.500000	62.299324	0.018683	98.073700
62.320000	97.500000	62.319324	0.017963	98.059906
62.340000	90.666695	62.339325	0.017721	98.046051
62.360001	96.666695	62.359325	0.017943	98.032196
62.380001	91.833298	62.379326	0.018639	98.018341
62.400002	93.666695	62.399326	0.019839	98.004456
62.420002	96.833298	62.419327	0.021602	97.990540
62.440002	96.333298	62.439327	0.024013	97.976624
62.460003	96.500000	62.459328	0.027196	97.962708
62.480003	91.500000	62.479332	0.031306	97.948730
62.500000	89.833298	62.499329	0.036609	97.934753
62.520000	93.166695	62.519329	0.043404	97.920807
62.540001	97.166695	62.539330	0.052122	97.906799
62.560001	91.666695	62.559330	0.063354	97.892792

62.580002	94.500000	62.579330	0.077916	97.878723
62.599998	98.166695	62.599327	0.096945	97.864685
62.619999	96.833298	62.619328	0.122063	97.850616
62.639999	100.667000	62.639328	0.155580	97.836548
62.660000	99.500000	62.659328	0.200866	97.822449
62.680000	101.000000	62.679329	0.262908	97.808350
62.700001	97.500000	62.699329	0.349215	97.794220
62.720001	100.667000	62.719330	0.471328	97.780090
62.740002	97.833298	62.739330	0.647353	97.765930
62.760002	103.833000	62.759331	0.906394	97.751740
62.780003	96.833298	62.779331	1.296521	97.737549
62.799999	100.833000	62.799328	1.899560	97.723358
62.820000	99.000000	62.819328	2.860772	97.709137
62.840000	100.000000	62.839329	4.447586	97.694885
62.860001	106.500000	62.859329	7.173962	97.680634
62.880001	111.833000	62.879330	12.059871	97.666351
62.900002	118.333000	62.899330	21.157547	97.652100
62.920002	137.500000	62.919331	38.522667	97.637787
62.940002	187.500000	62.939331	71.595871	97.623474
62.960003	256.832977	62.959332	131.563797	97.609161
62.980003	373.000000	62.979332	226.936707	97.594818
63.000000	495.332977	62.999329	341.098724	97.580444
63.020000	569.833008	63.019329	410.849030	97.566101
63.040001	531.833008	63.039330	376.250092	97.551727
63.060001	427.832977	63.059330	268.164490	97.537323
63.080002	297.832977	63.079330	162.719910	97.522888
63.099998	218.667007	63.099327	92.625389	97.508484
63.119999	193.333008	63.119331	52.528954	97.494049
63.139999	153.500000	63.139332	30.392056	97.479553
63.160000	140.333008	63.159332	18.028500	97.465088
63.180000	116.167000	63.179333	10.978271	97.450623
63.200001	112.500000	63.199333	6.887492	97.436127
63.220001	104.333000	63.219334	4.489672	97.421631
63.240002	106.500000	63.239334	3.090506	97.407074
63.260002	104.000000	63.259335	2.312338	97.392548
63.280003	101.500000	63.279335	1.966307	97.377991
63.299999	99.333298	63.299332	1.995569	97.363434
63.320000	96.333298	63.319332	2.471337	97.348846

63.340000	103.333000	63.339333	3.646330	97.334259
63.360001	111.833000	63.359333	6.086942	97.319672
63.380001	113.833000	63.379333	10.888825	97.305023
63.400002	122.833000	63.399334	19.810516	97.290436
63.420002	145.667007	63.419334	34.557056	97.275757
63.440002	167.167007	63.439335	53.647259	97.261108
63.460003	188.833008	63.459335	68.052948	97.246460
63.480003	182.000000	63.479336	66.207909	97.231750
63.500000	165.833008	63.499332	49.665871	97.217102
63.520000	144.333008	63.519333	31.077219	97.202393
63.540001	125.167000	63.539333	17.925859	97.187653
63.560001	115.167000	63.559334	10.228800	97.172943
63.580002	106.333000	63.579334	5.967687	97.158173
63.599998	108.833000	63.599331	3.609236	97.143463
63.619999	103.167000	63.619331	2.291771	97.128662
63.639999	102.167000	63.639332	1.562026	97.113922
63.660000	105.167000	63.659332	1.180460	97.099121
63.680000	101.667000	63.679333	1.022242	97.084320
63.700001	98.000000	63.699333	1.029093	97.069519
63.720001	90.166695	63.719334	1.187508	97.054688
63.740002	101.333000	63.739334	1.522724	97.039856
63.760002	100.833000	63.759338	2.106920	97.025024
63.780003	98.166695	63.779339	3.085878	97.010193
63.799999	103.667000	63.799335	4.741493	96.995331
63.820000	103.000000	63.819336	7.626828	96.980469
63.840000	123.333000	63.839336	12.845583	96.965607
63.860001	125.000000	63.859337	22.613085	96.950684
63.880001	158.333008	63.879337	41.240032	96.935791
63.900002	216.500000	63.899338	76.332382	96.920868
63.920002	275.666992	63.919338	138.234497	96.905945
63.940002	381.832977	63.939339	231.342941	96.891052
63.960003	455.166992	63.959339	331.231018	96.876099
63.980003	481.666992	63.979340	375.232605	96.861145
64.000000	454.000000	63.999332	325.089630	96.846222
64.019997	366.500000	64.019325	225.814590	96.831238
64.040001	294.166992	64.039330	140.834335	96.816254
64.059998	253.000000	64.059326	91.046623	96.801300
64.080002	212.000000	64.079330	71.926468	96.786316

64.099998	219.000000	64.099327	78.806473	96.771301
64.119995	261.166992	64.119324	113.148201	96.756317
64.139999	320.000000	64.139328	176.649597	96.741272
64.159996	408.000000	64.159325	253.478104	96.726288
64.180000	442.166992	64.179329	296.906433	96.711243
64.199997	423.500000	64.199326	267.501709	96.696198
64.219994	345.500000	64.219322	189.768402	96.681183
64.239998	267.666992	64.239326	115.631607	96.666138
64.259995	191.000000	64.259323	66.376938	96.651062
64.279999	159.667007	64.279327	37.966255	96.635986
64.300003	144.667007	64.299332	22.113110	96.620941
64.320000	131.667007	64.319328	13.175469	96.605835
64.340004	118.500000	64.339333	8.030197	96.590729
64.360001	104.333000	64.359329	5.012733	96.575684
64.380005	104.000000	64.379333	3.208672	96.560577
64.400002	107.167000	64.399330	2.109514	96.545502
64.419998	100.667000	64.419327	1.424873	96.530365
64.440002	100.000000	64.439331	0.988388	96.515259
64.459999	95.833298	64.459328	0.703927	96.500153
64.480003	100.000000	64.479332	0.514484	96.485016
64.500000	96.500000	64.499329	0.386217	96.469879
64.519997	102.000000	64.519325	0.298308	96.454773
64.540001	106.000000	64.539330	0.237859	96.439636
64.559998	104.333000	64.559326	0.196822	96.424500
64.580002	95.166695	64.579330	0.170074	96.409332
64.599998	93.333298	64.599327	0.154531	96.394226
64.619995	104.500000	64.619324	0.148467	96.379059
64.639999	96.000000	64.639328	0.151254	96.363892
64.659996	98.000000	64.659325	0.163232	96.348724
64.680000	99.500000	64.679329	0.185783	96.333557
64.699997	95.500000	64.699326	0.221529	96.318420
64.719994	94.666695	64.719330	0.274891	96.303253
64.739998	95.833298	64.739334	0.352861	96.288086
64.759995	105.667000	64.759331	0.466448	96.272888
64.779999	100.167000	64.779335	0.633365	96.257721
64.800003	106.500000	64.799339	0.882172	96.242523
64.820000	102.500000	64.819336	1.260197	96.227356
64.840004	99.500000	64.839340	1.849055	96.212189

64.860001	100.667000	64.859337	2.792692	96.196960
64.880005	108.500000	64.879341	4.359270	96.181793
64.900002	106.333000	64.899338	7.062641	96.166626
64.919998	109.667000	64.919334	11.925351	96.151428
64.940002	122.333000	64.939339	20.997332	96.136230
64.959999	139.667007	64.959335	38.251526	96.121063
64.980003	192.333008	64.979340	70.752983	96.105865
65.000000	276.666992	64.999336	128.233688	96.090668
65.019997	360.832977	65.019333	215.655121	96.075500
65.040001	464.832977	65.039337	312.501862	96.060303
65.059998	517.666992	65.059334	361.123505	96.045105
65.080002	479.500000	65.079338	319.966309	96.029938
65.099998	387.666992	65.099335	224.881409	96.014709
65.119995	299.332977	65.119331	137.241486	95.999542
65.139999	213.833008	65.139336	79.841606	95.984375
65.159996	177.333008	65.159332	47.406231	95.969147
65.180000	157.833008	65.179337	30.558702	95.953979
65.199997	137.667007	65.199333	23.585558	95.938782
65.219994	134.167007	65.219330	24.261259	95.923615
65.239998	135.833008	65.239334	32.391376	95.908447
65.259995	156.333008	65.259331	47.127029	95.893280
65.279999	166.500000	65.279335	62.514183	95.878113
65.300003	169.667007	65.299339	67.089989	95.862915
65.320000	159.667007	65.319336	55.888004	95.847748
65.340004	141.833008	65.339340	37.741810	95.832611
65.360001	127.667000	65.359337	22.639051	95.817413
65.380005	118.833000	65.379341	13.070143	95.802277
65.400002	106.333000	65.399338	7.586951	95.787109
65.419998	101.000000	65.419334	4.491832	95.772003
65.440002	101.167000	65.439339	2.719582	95.756836
65.459999	99.000000	65.459335	1.686121	95.741699
65.480003	106.833000	65.479340	1.071798	95.726562
65.500000	99.166695	65.499336	0.699916	95.711426
65.519997	96.500000	65.519333	0.469841	95.696320
65.540001	94.666695	65.539337	0.324028	95.681213
65.559998	100.500000	65.559334	0.229325	95.666077
65.580002	95.333298	65.579338	0.166192	95.650970
65.599998	97.000000	65.599335	0.123113	95.635864

65.619995	94.833298	65.619331	0.093047	95.620758
65.639999	88.833298	65.639336	0.071638	95.605682
65.659996	91.166695	65.659332	0.056140	95.590607
65.680000	88.833298	65.679337	0.044745	95.575531
65.699997	90.500000	65.699333	0.036275	95.560455
65.719994	89.333298	65.719330	0.029924	95.545380
65.739998	90.166695	65.739334	0.025143	95.530334
65.759995	105.667000	65.759331	0.021555	95.515289
65.779999	95.500000	65.779335	0.018896	95.500244
65.800003	94.000000	65.799339	0.016984	95.485199
65.820000	99.666695	65.819336	0.015700	95.470215
65.840004	99.000000	65.839340	0.014965	95.455200
65.860001	97.666695	65.859337	0.014744	95.440186
65.880005	94.500000	65.879341	0.015031	95.425201
65.900002	93.166695	65.899338	0.015855	95.410187
65.919998	90.833298	65.919334	0.017284	95.395233
65.940002	96.833298	65.939339	0.019429	95.380249
65.959999	88.666695	65.959343	0.022457	95.365295
65.980003	88.166695	65.979347	0.026614	95.350372
66.000000	96.333298	65.999344	0.032251	95.335419
66.019997	96.166695	66.019341	0.039879	95.320496
66.040001	93.333298	66.039345	0.050241	95.305573
66.059998	96.000000	66.059341	0.064413	95.290680
66.080002	98.833298	66.079346	0.084020	95.275787
66.099998	96.666695	66.099342	0.111484	95.260925
66.119995	102.833000	66.119339	0.150566	95.246033
66.139999	98.000000	66.139343	0.207186	95.231201
66.159996	100.667000	66.159340	0.290775	95.216339
66.180000	98.500000	66.179344	0.417021	95.201508
66.199997	105.667000	66.199341	0.612359	95.186676
66.219994	100.833000	66.219337	0.923631	95.171906
66.239998	107.167000	66.239342	1.436926	95.157104
66.259995	100.833000	66.259338	2.316056	95.142303
66.279999	103.333000	66.279343	3.885604	95.127563
66.300003	107.833000	66.299347	6.790223	95.112793
66.320000	112.000000	66.319344	12.277151	95.098053
66.340004	128.333008	66.339348	22.565575	95.083344
66.360001	163.833008	66.359344	40.774956	95.068634

66.380005	198.667007	66.379349	68.917557	95.053955
66.400002	228.667007	66.399345	102.034843	95.039276
66.419998	243.500000	66.419342	123.854843	95.024628
66.440002	232.000000	66.439346	118.361763	95.009979
66.459999	216.667007	66.459343	89.961594	94.995331
66.480003	169.667007	66.479347	57.836281	94.980713
66.500000	145.167007	66.499344	34.150562	94.966125
66.519997	126.000000	66.519341	19.715309	94.951538
66.540001	115.167000	66.539345	11.467206	94.936981
66.559998	113.500000	66.559341	6.784230	94.922455
66.580002	103.167000	66.579346	4.088876	94.907898
66.599998	103.167000	66.599342	2.515076	94.893372
66.619995	90.166695	66.619339	1.581742	94.878906
66.639999	95.333298	66.639343	1.018772	94.864410
66.659996	91.000000	66.659340	0.672780	94.849976
66.680000	96.833298	66.679344	0.455211	94.835510
66.699997	90.333298	66.699341	0.315245	94.821136
66.719994	93.833298	66.719337	0.223013	94.806732
66.739998	89.666695	66.739342	0.160834	94.792328
66.759995	90.166695	66.759338	0.118065	94.777985
66.779999	90.500000	66.779343	0.088055	94.763611
66.800003	89.166695	66.799347	0.066638	94.749298
66.820000	90.666695	66.819344	0.051113	94.735016
66.840004	86.500000	66.839348	0.039687	94.720703
66.860001	91.333298	66.859344	0.031173	94.706451
66.880005	92.166695	66.879349	0.024747	94.692200
66.900002	91.833298	66.899345	0.019849	94.677979
66.919998	93.333298	66.919342	0.016078	94.663788
66.940002	90.166695	66.939346	0.013153	94.649628
66.959999	92.333298	66.959343	0.010873	94.635498
66.980003	84.500000	66.979347	0.009092	94.621368
67.000000	86.000000	66.999344	0.007707	94.607239
67.019997	87.500000	67.019341	0.006648	94.593170
67.040001	90.000000	67.039345	0.005868	94.579071
67.059998	87.833298	67.059341	0.005349	94.565063
67.080002	92.000000	67.079346	0.005096	94.551025
67.099998	89.666695	67.099342	0.005161	94.537048
67.119995	85.500000	67.119339	0.005644	94.523071

67.139999	82.333298	67.139343	0.006746	94.509155
67.159996	95.000000	67.159340	0.008850	94.495209
67.180000	98.000000	67.179344	0.012705	94.481323
67.199997	84.833298	67.199348	0.019790	94.467407
67.219994	88.333298	67.219345	0.033057	94.453583
67.239998	90.333298	67.239349	0.058297	94.439758
67.259995	89.666695	67.259346	0.105931	94.425995
67.279999	89.500000	67.279350	0.191449	94.412201
67.300003	85.500000	67.299355	0.326918	94.398468
67.320000	89.166695	67.319351	0.492540	94.384735
67.340004	87.500000	67.339355	0.607572	94.371033
67.360001	95.166695	67.359352	0.583291	94.357391
67.380005	85.166695	67.379356	0.440618	94.343781
67.400002	89.500000	67.399353	0.281597	94.330170
67.419998	99.666695	67.419350	0.166277	94.316589
67.440002	90.666695	67.439354	0.096427	94.303040
67.459999	92.833298	67.459351	0.056456	94.289490
67.480003	88.666695	67.479355	0.033623	94.275970
67.500000	97.166695	67.499352	0.020427	94.262543
67.519997	98.500000	67.519348	0.012694	94.249115
67.540001	90.166695	67.539352	0.008099	94.235687
67.559998	86.500000	67.559349	0.005328	94.222321
67.580002	97.000000	67.579353	0.003626	94.208954
67.599998	89.333298	67.599350	0.002561	94.195618
67.619995	88.666695	67.619347	0.001880	94.182373
67.639999	91.500000	67.639351	0.001445	94.169098
67.659996	93.333298	67.659348	0.001158	94.155853
67.680000	94.166695	67.679352	0.000972	94.142639
67.699997	92.666695	67.699348	0.000855	94.129486
67.719994	91.500000	67.719345	0.000789	94.116364
67.739998	92.500000	67.739349	0.000760	94.103241
67.759995	85.000000	67.759346	0.000763	94.090179
67.779999	95.000000	67.779350	0.000794	94.077118
67.800003	96.500000	67.799355	0.000851	94.064117
67.820000	92.666695	67.819351	0.000936	94.051117
67.840004	91.666695	67.839355	0.001051	94.038208
67.860001	91.166695	67.859352	0.001201	94.025269
67.880005	90.500000	67.879356	0.001393	94.012390

67.900002	80.500000	67.899353	0.001637	93.999542
67.919998	90.333298	67.919350	0.001947	93.986725
67.940002	89.000000	67.939354	0.002341	93.973938
67.959999	93.833298	67.959351	0.002843	93.961212
67.980003	91.833298	67.979355	0.003489	93.948486
68.000000	92.833298	67.999352	0.004325	93.935822
68.019997	93.000000	68.019348	0.005417	93.923187
68.040001	90.000000	68.039352	0.006860	93.910583
68.059998	89.666695	68.059349	0.008787	93.897980
68.080002	88.666695	68.079353	0.011394	93.885468
68.099998	94.000000	68.099350	0.014968	93.872986
68.119995	91.166695	68.119347	0.019942	93.860504
68.139999	88.833298	68.139351	0.026984	93.848083
68.159996	85.833298	68.159348	0.037122	93.835724
68.180000	82.500000	68.179352	0.052015	93.823334
68.199997	84.833298	68.199348	0.074344	93.811035
68.219994	86.000000	68.219345	0.108628	93.798767
68.239998	91.666695	68.239349	0.162734	93.786499
68.259995	86.666695	68.259346	0.250792	93.774292
68.279999	93.000000	68.279350	0.399598	93.762115
68.300003	91.666695	68.299355	0.661337	93.750000
68.320000	82.500000	68.319351	1.139474	93.737885
68.340004	83.000000	68.339355	2.037252	93.725861
68.360001	96.333298	68.359352	3.724849	93.713837
68.380005	102.667000	68.379356	6.774437	93.701874
68.400002	100.667000	68.399361	11.714653	93.689941
68.419998	111.333000	68.419357	18.089258	93.678009
68.440002	117.000000	68.439362	23.207653	93.666138
68.459999	110.500000	68.459358	23.398146	93.654358
68.480003	111.167000	68.479362	18.509768	93.642578
68.500000	108.167000	68.499359	12.216411	93.630829
68.519997	98.666695	68.519356	7.333078	93.619141
68.540001	93.333298	68.539360	4.276423	93.607483
68.559998	87.333298	68.559357	2.503016	93.595856
68.580002	91.333298	68.579361	1.484807	93.584259
68.599998	90.500000	68.599358	0.895592	93.572784
68.619995	88.000000	68.619354	0.550660	93.561279
68.639999	84.833298	68.639359	0.346521	93.549805

68.659996	87.000000	68.659355	0.224442	93.538391
68.680000	92.833298	68.679359	0.150535	93.527008
68.699997	86.833298	68.699356	0.105479	93.515717
68.719994	84.666695	68.719353	0.078176	93.504456
68.739998	85.666695	68.739357	0.062394	93.493195
68.759995	83.666695	68.759354	0.054800	93.482025
68.779999	88.000000	68.779358	0.053903	93.470856
68.800003	93.000000	68.799362	0.059652	93.459747
68.820000	84.000000	68.819359	0.073418	93.448700
68.840004	86.500000	68.839363	0.098532	93.437653
68.860001	92.500000	68.859360	0.141515	93.426697
68.880005	95.833298	68.879364	0.214959	93.415741
68.900002	89.500000	68.899361	0.343270	93.404877
68.919998	82.333298	68.919357	0.574868	93.394012
68.940002	88.833298	68.939362	1.005109	93.383240
68.959999	94.666695	68.959358	1.811674	93.372467
68.980003	91.833298	68.979362	3.287101	93.361786
69.000000	91.666695	68.999359	5.768651	93.351105
69.019997	97.166695	69.019356	9.262698	93.340485
69.040001	96.833298	69.039360	12.743431	93.329926
69.059998	98.166695	69.059357	14.287907	93.319397
69.080002	105.000000	69.079361	13.232318	93.308929
69.099998	100.500000	69.099358	11.204449	93.298462
69.119995	96.166695	69.119354	9.651954	93.288116
69.139999	91.666695	69.139359	8.274344	93.277771
69.159996	92.666695	69.159355	6.407117	93.267487
69.180000	95.833298	69.179359	4.335060	93.257233
69.199997	97.500000	69.199356	2.673859	93.247070
69.219994	90.333298	69.219353	1.593219	93.236908
69.239998	86.333298	69.239357	0.952226	93.226807
69.259995	85.833298	69.259354	0.581469	93.216736
69.279999	95.833298	69.279358	0.366882	93.206757
69.300003	85.166695	69.299362	0.242891	93.196777
69.320000	87.500000	69.319359	0.172579	93.186859
69.340004	84.166695	69.339363	0.135215	93.177032
69.360001	85.166695	69.359360	0.119532	93.167206
69.380005	86.166695	69.379364	0.119916	93.157440
69.400002	91.166695	69.399361	0.134568	93.147736

69.419998	89.000000	69.419357	0.164711	93.138092
69.440002	86.833298	69.439362	0.214707	93.128479
69.459999	85.666695	69.459358	0.292929	93.118927
69.480003	90.333298	69.479362	0.414206	93.109406
69.500000	88.000000	69.499359	0.603990	93.099945
69.519997	93.333298	69.519356	0.907479	93.090546
69.540001	90.000000	69.539360	1.407706	93.081177
69.559998	91.166695	69.559357	2.262168	93.071869
69.580002	88.833298	69.579361	3.781758	93.062622
69.599998	95.166695	69.599365	6.580216	93.053406
69.619995	101.500000	69.619362	11.833329	93.044250
69.639999	114.167000	69.639366	21.601809	93.035156
69.659996	137.333008	69.659363	38.711582	93.026123
69.680000	157.833008	69.679367	64.825447	93.017090
69.699997	188.500000	69.699364	94.989258	93.008148
69.719994	207.500000	69.719360	113.771530	92.999268
69.739998	210.167007	69.739365	106.905838	92.990417
69.759995	189.333008	69.759361	80.203033	92.981628
69.779999	145.167007	69.779366	51.555141	92.972900
69.800003	121.500000	69.799370	30.805616	92.964203
69.820000	113.167000	69.819366	18.081116	92.955597
69.840004	114.667000	69.839371	10.674240	92.946991
69.860001	101.167000	69.859367	6.389107	92.938477
69.880005	93.500000	69.879372	3.891627	92.929993
69.900002	95.000000	69.899368	2.429345	92.921600
69.919998	90.833298	69.919365	1.573413	92.913239
69.940002	87.833298	69.939369	1.081190	92.904907
69.959999	86.833298	69.959366	0.820000	92.896637
69.980003	79.833298	69.979370	0.726790	92.888458
70.000000	80.333298	69.999367	0.793369	92.880310
70.019997	92.666695	70.019363	1.070625	92.872223
70.040001	84.166695	70.039368	1.694741	92.864166
70.059998	94.500000	70.059364	2.924976	92.856171
70.080002	88.000000	70.079369	5.141487	92.848267
70.099998	100.500000	70.099365	8.610329	92.840393
70.119995	98.666695	70.119362	12.794602	92.832581
70.139999	98.500000	70.139366	15.705029	92.824799
70.159996	97.833298	70.159363	15.196964	92.817108

70.180000	107.667000	70.179367	11.701843	92.809448
70.199997	103.667000	70.199364	7.655399	92.801849
70.219994	98.166695	70.219360	4.616179	92.794312
70.239998	92.333298	70.239365	2.719358	92.786835
70.259995	87.833298	70.259361	1.608401	92.779419
70.279999	80.500000	70.279366	0.962966	92.772034
70.300003	95.166695	70.299370	0.585709	92.764740
70.320000	87.000000	70.319366	0.363548	92.757446
70.340004	84.833298	70.339371	0.231765	92.750275
70.360001	78.833298	70.359367	0.153365	92.743134
70.380005	85.333298	70.379372	0.107044	92.736023
70.400002	87.500000	70.399368	0.081067	92.728973
70.419998	85.333298	70.419365	0.069620	92.722015
70.440002	85.666695	70.439369	0.071385	92.715088
70.459999	83.833298	70.459366	0.089615	92.708252
70.480003	86.500000	70.479370	0.133819	92.701447
70.500000	82.333298	70.499367	0.222069	92.694702
70.519997	88.666695	70.519363	0.379666	92.688019
70.540001	83.833298	70.539368	0.620793	92.681396
70.559998	92.333298	70.559364	0.899473	92.674805
70.580002	88.833298	70.579369	1.076945	92.668304
70.599998	87.666695	70.599365	1.027376	92.661835
70.619995	89.666695	70.619362	0.805799	92.655457
70.639999	88.166695	70.639366	0.578105	92.649109
70.659996	97.333298	70.659363	0.444673	92.642822
70.680000	86.166695	70.679367	0.428332	92.636627
70.699997	87.666695	70.699364	0.545608	92.630463
70.719994	94.666695	70.719360	0.854177	92.624359
70.739998	89.166695	70.739365	1.478928	92.618317
70.759995	91.666695	70.759361	2.606764	92.612335
70.779999	101.000000	70.779373	4.371533	92.606415
70.800003	98.666695	70.799377	6.500558	92.600525
70.820000	93.166695	70.819374	7.996511	92.594727
70.840004	91.000000	70.839378	7.776453	92.588959
70.860001	88.500000	70.859375	6.030539	92.583282
70.880005	86.833298	70.879379	3.972952	92.577637
70.900002	87.666695	70.899376	2.410682	92.572083
70.919998	94.500000	70.919373	1.428582	92.566589

70.940002	82.833298	70.939377	0.850669	92.561127
70.959999	82.666695	70.959373	0.515278	92.555756
70.980003	86.166695	70.979378	0.320540	92.550415
71.000000	87.166695	70.999374	0.208295	92.545135
71.019997	89.833298	71.019371	0.145349	92.539917
71.040001	85.833298	71.039375	0.113129	92.534760
71.059998	86.000000	71.059372	0.101904	92.529694
71.080002	90.833298	71.079376	0.107764	92.524658
71.099998	83.500000	71.099373	0.131542	92.519684
71.119995	84.500000	71.119370	0.179375	92.514801
71.139999	88.666695	71.139374	0.265654	92.509918
71.159996	84.333298	71.159370	0.419885	92.505157
71.180000	88.500000	71.179375	0.701500	92.500397
71.199997	101.500000	71.199371	1.225580	92.495758
71.219994	85.500000	71.219368	2.200028	92.491150
71.239998	89.666695	71.239372	3.938981	92.486603
71.259995	99.833298	71.259369	6.723614	92.482117
71.279999	103.000000	71.279373	10.312016	92.477692
71.300003	106.333000	71.299377	13.284997	92.473328
71.320000	105.333000	71.319374	13.660458	92.469025
71.340004	98.000000	71.339378	11.187140	92.464783
71.360001	102.667000	71.359375	7.738702	92.460602
71.380005	97.666695	71.379379	4.962864	92.456482
71.400002	90.500000	71.399376	3.242039	92.452423
71.419998	92.000000	71.419373	2.350505	92.448456
71.440002	93.666695	71.439377	2.042318	92.444519
71.459999	95.333298	71.459373	2.204703	92.440643
71.480003	91.833298	71.479370	2.870210	92.436829
71.500000	94.000000	71.499367	4.249671	92.433075
71.519997	94.166695	71.519363	6.843981	92.429382
71.540001	99.666695	71.539368	11.692079	92.425751
71.559998	116.667000	71.559364	20.791838	92.422180
71.580002	155.833008	71.579369	37.622044	92.418671
71.599998	188.167007	71.599365	66.899292	92.415253
71.619995	226.667007	71.619362	111.387253	92.411865
71.639999	301.332977	71.639366	163.322083	92.408539
71.659996	324.500000	71.659363	198.149643	92.405273
71.680000	314.832977	71.679367	192.286697	92.402100

71.699997	270.666992	71.699364	152.375305	92.398956
71.719994	219.167007	71.719360	105.041176	92.395874
71.739998	183.167007	71.739365	66.890541	92.392853
71.759995	152.500000	71.759361	40.845692	92.389923
71.779999	131.000000	71.779366	24.499693	92.387024
71.800003	123.667000	71.799370	14.684614	92.384186
71.820000	117.167000	71.819366	8.882202	92.381439
71.840004	110.833000	71.839371	5.450084	92.378723
71.860001	100.667000	71.859367	3.410110	92.376068
71.880005	105.667000	71.879372	2.186452	92.373505
71.900002	92.166695	71.899368	1.445580	92.370972
71.919998	88.000000	71.919373	0.992415	92.368530
71.940002	90.166695	71.939377	0.714744	92.366119
71.959999	92.166695	71.959373	0.548062	92.363800
71.980003	91.333298	71.979378	0.456113	92.361511
72.000000	93.166695	71.999374	0.420330	92.359314
72.019997	91.666695	72.019371	0.433960	92.357147
72.040001	89.333298	72.039375	0.500272	92.355072
72.059998	94.000000	72.059372	0.633379	92.353058
72.080002	99.166695	72.079376	0.863003	92.351074
72.099998	101.500000	72.099373	1.244394	92.349182
72.119995	89.333298	72.119370	1.880784	92.347321
72.139999	89.500000	72.139374	2.969531	92.345551
72.159996	98.833298	72.159370	4.894829	92.343842
72.180000	105.833000	72.179375	8.415504	92.342194
72.199997	111.500000	72.199371	14.971798	92.340576
72.219994	124.167000	72.219368	27.071005	92.339050
72.239998	155.333008	72.239372	48.203342	92.337555
72.259995	190.667007	72.259369	80.631142	92.336151
72.279999	220.333008	72.279373	119.329475	92.334808
72.300003	249.500000	72.299377	146.658066	92.333527
72.320000	250.833008	72.319374	143.949768	92.332275
72.340004	216.000000	72.339378	114.660324	92.331116
72.360001	193.667007	72.359375	79.753357	92.330017
72.380005	161.500000	72.379379	52.711643	92.328949
72.400002	144.500000	72.399376	34.387959	92.327972
72.419998	123.833000	72.419373	21.926970	92.327057
72.440002	117.500000	72.439377	13.568051	92.326202

72.459999	113.833000	72.459373	8.262488	92.325409
72.480003	112.000000	72.479378	5.038322	92.324677
72.500000	98.000000	72.499374	3.112039	92.323975
72.519997	90.166695	72.519371	1.957650	92.323364
72.540001	88.166695	72.539375	1.257870	92.322784
72.559998	90.166695	72.559372	0.827505	92.322296
72.580002	89.500000	72.579376	0.557887	92.321869
72.599998	86.500000	72.599373	0.385850	92.321472
72.619995	90.000000	72.619370	0.273952	92.321167
72.639999	84.500000	72.639374	0.200020	92.320892
72.659996	84.333298	72.659370	0.150861	92.320709
72.680000	89.000000	72.679375	0.118509	92.320557
72.699997	92.500000	72.699371	0.098372	92.320496
72.719994	83.833298	72.719368	0.088053	92.320465
72.739998	90.500000	72.739372	0.086934	92.320526
72.759995	83.166695	72.759369	0.096227	92.320648
72.779999	88.833298	72.779373	0.119659	92.320770
72.800003	82.833298	72.799377	0.165237	92.321014
72.820000	81.666695	72.819374	0.249098	92.321289
72.840004	90.500000	72.839378	0.403609	92.321625
72.860001	94.166695	72.859375	0.691878	92.322021
72.880005	84.666695	72.879379	1.231051	92.322479
72.900002	86.333298	72.899376	2.208941	92.322998
72.919998	88.666695	72.919373	3.839740	92.323608
72.940002	103.167000	72.939377	6.128150	92.324219
72.959999	102.667000	72.959373	8.422560	92.324921
72.980003	95.333298	72.979378	9.411287	92.325684
73.000000	103.667000	72.999374	8.354195	92.326477
73.019997	99.166695	73.019371	6.081956	92.327362
73.040001	95.333298	73.039375	3.898387	92.328278
73.059998	89.500000	73.059372	2.361455	92.329285
73.080002	92.333298	73.079384	1.410724	92.330322
73.099998	86.666695	73.099380	0.846557	92.331421
73.119995	92.833298	73.119377	0.513047	92.332611
73.139999	86.666695	73.139381	0.315171	92.333832
73.159996	86.500000	73.159378	0.197400	92.335114
73.180000	85.166695	73.179382	0.126912	92.336426
73.199997	81.666695	73.199379	0.084471	92.337830

73.219994	84.500000	73.219376	0.058710	92.339264
73.239998	83.333298	73.239380	0.043016	92.340790
73.259995	86.166695	73.259377	0.033568	92.342346
73.279999	88.500000	73.279381	0.028147	92.343964
73.300003	94.000000	73.299385	0.025502	92.345673
73.320000	88.000000	73.319382	0.024936	92.347382
73.340004	90.666695	73.339386	0.026124	92.349182
73.360001	88.333298	73.359383	0.028998	92.351013
73.380005	85.500000	73.379387	0.033699	92.352936
73.400002	85.333298	73.399384	0.040585	92.354889
73.419998	80.833298	73.419380	0.050277	92.356903
73.440002	85.500000	73.439384	0.063752	92.358978
73.459999	87.333298	73.459381	0.082489	92.361115
73.480003	88.833298	73.479385	0.108770	92.363312
73.500000	83.833298	73.499382	0.146062	92.365540
73.519997	88.000000	73.519379	0.199816	92.367828
73.540001	89.333298	73.539383	0.278706	92.370209
73.559998	85.666695	73.559380	0.396705	92.372589
73.580002	84.666695	73.579384	0.577344	92.375061
73.599998	74.666695	73.599380	0.861061	92.377563
73.619995	91.000000	73.619377	1.321195	92.380127
73.639999	90.333298	73.639381	2.096203	92.382782
73.659996	88.333298	73.659378	3.453709	92.385468
73.680000	95.833298	73.679382	5.918572	92.388184
73.699997	108.167000	73.699379	10.476693	92.390961
73.719994	116.667000	73.719376	18.816957	92.393799
73.739998	140.167007	73.739380	33.224148	92.396698
73.759995	171.500000	73.759377	55.030800	92.399658
73.779999	183.667007	73.779381	80.645340	92.402649
73.800003	197.833008	73.799385	98.374168	92.405701
73.820000	196.000000	73.819382	96.183609	92.408813
73.840004	176.667007	73.839386	76.374695	92.411957
73.860001	164.333008	73.859383	52.773621	92.415192
73.880005	141.167007	73.879387	35.148140	92.418457
73.900002	129.667007	73.899384	25.461748	92.421753
73.919998	116.333000	73.919380	22.910336	92.425110
73.940002	124.000000	73.939384	27.601967	92.428558
73.959999	151.833008	73.959381	41.641918	92.432007

73.980003	196.167007	73.979385	68.661751	92.435547
74.000000	231.000000	73.999382	110.304695	92.439117
74.019997	274.500000	74.019379	158.211746	92.442749
74.040001	307.500000	74.039383	188.941666	92.446411
74.059998	283.500000	74.059380	180.657806	92.450134
74.080002	256.166992	74.079384	140.034180	92.453918
74.099998	214.167007	74.099380	93.446396	92.457733
74.119995	154.667007	74.119377	57.719654	92.461609
74.139999	130.000000	74.139381	34.744568	92.465546
74.159996	121.500000	74.159378	20.884237	92.469482
74.180000	114.667000	74.179382	12.627034	92.473541
74.199997	108.000000	74.199387	7.704587	92.477600
74.219994	103.833000	74.219383	4.761238	92.481720
74.239998	91.666695	74.239388	2.991524	92.485901
74.259995	93.666695	74.259384	1.919336	92.490112
74.279999	89.833298	74.279388	1.260434	92.494354
74.300003	84.166695	74.299393	0.848256	92.498688
74.320000	86.666695	74.319389	0.584914	92.503052
74.340004	87.833298	74.339394	0.412715	92.507446
74.360001	84.500000	74.359390	0.297759	92.511902
74.380005	84.500000	74.379395	0.219488	92.516418
74.400002	81.000000	74.399391	0.165411	92.520966
74.419998	87.166695	74.419388	0.127647	92.525574
74.440002	82.000000	74.439392	0.101193	92.530212
74.459999	82.333298	74.459389	0.082858	92.534912
74.480003	82.000000	74.479393	0.070579	92.539642
74.500000	85.166695	74.499390	0.063097	92.544403
74.519997	79.500000	74.519386	0.059721	92.549255
74.540001	79.500000	74.539391	0.060238	92.554108
74.559998	86.666695	74.559387	0.064902	92.559021
74.580002	79.166695	74.579391	0.074482	92.563995
74.599998	84.666695	74.599388	0.090433	92.569000
74.619995	89.000000	74.619385	0.115230	92.574036
74.639999	88.000000	74.639389	0.152928	92.579132
74.659996	84.166695	74.659386	0.210091	92.584259
74.680000	88.833298	74.679390	0.297689	92.589447
74.699997	83.166695	74.699387	0.434253	92.594666
74.719994	91.666695	74.719383	0.652692	92.599945

74.739998	87.833298	74.739388	1.013890	92.605255
74.759995	89.833298	74.759384	1.633831	92.610596
74.779999	86.833298	74.779388	2.739568	92.615997
74.800003	100.000000	74.799393	4.765854	92.621429
74.820000	104.833000	74.819389	8.492537	92.626892
74.840004	110.000000	74.839394	15.121748	92.632416
74.860001	122.000000	74.859390	25.862366	92.637970
74.880005	144.167007	74.879395	40.334030	92.643555
74.900002	152.333008	74.899391	54.011662	92.649200
74.919998	152.333008	74.919388	59.117264	92.654907
74.940002	152.167007	74.939392	52.378330	92.660614
74.959999	143.500000	74.959389	39.650120	92.666382
74.980003	128.500000	74.979393	28.887156	92.672180
75.000000	124.667000	74.999390	23.902687	92.678009
75.019997	126.167000	75.019386	25.461277	92.683899
75.040001	133.000000	75.039391	33.269474	92.689789
75.059998	140.000000	75.059387	45.018555	92.695770
75.080002	154.000000	75.079391	54.508278	92.701721
75.099998	146.167007	75.099388	54.452805	92.707764
75.119995	143.667007	75.119385	44.284073	92.713837
75.139999	135.500000	75.139389	30.652067	92.719940
75.159996	113.167000	75.159386	19.369450	92.726074
75.180000	104.000000	75.179390	11.833109	92.732239
75.199997	94.000000	75.199387	7.216158	92.738464
75.219994	91.666695	75.219383	4.459154	92.744720
75.239998	96.833298	75.239388	2.830076	92.750977
75.259995	97.166695	75.259384	1.887975	92.757324
75.279999	92.500000	75.279388	1.373300	92.763672
75.300003	100.167000	75.299400	1.140197	92.770050
75.320000	103.833000	75.319397	1.115854	92.776489
75.340004	98.666695	75.339401	1.281180	92.782959
75.360001	98.666695	75.359398	1.666998	92.789429
75.380005	96.833298	75.379402	2.367601	92.795959
75.400002	102.500000	75.399399	3.579860	92.802521
75.419998	105.167000	75.419395	5.696101	92.809113
75.440002	123.500000	75.439400	9.491184	92.815735
75.459999	127.833000	75.459396	16.456707	92.822388
75.480003	164.667007	75.479401	29.316109	92.829071

75.500000	202.500000	75.499397	52.338001	92.835785
75.519997	245.833008	75.519394	90.226868	92.842529
75.540001	278.666992	75.539398	142.850235	92.849335
75.559998	326.000000	75.559395	195.733902	92.856140
75.580002	348.666992	75.579399	220.126251	92.862976
75.599998	319.666992	75.599396	198.735474	92.869843
75.619995	287.832977	75.619392	148.048599	92.876770
75.639999	240.667007	75.639397	97.079002	92.883698
75.659996	203.333008	75.659393	59.914589	92.890656
75.680000	162.667007	75.679398	36.300674	92.897644
75.699997	144.333008	75.699394	21.989576	92.904663
75.719994	130.667007	75.719391	13.393785	92.911682
75.739998	121.000000	75.739395	8.231356	92.918762
75.759995	113.833000	75.759392	5.132487	92.925873
75.779999	111.333000	75.779396	3.269305	92.933014
75.800003	101.167000	75.799400	2.147651	92.940155
75.820000	92.000000	75.819397	1.473118	92.947327
75.840004	95.333298	75.839401	1.073338	92.954529
75.860001	93.000000	75.859398	0.851544	92.961761
75.880005	97.333298	75.879402	0.756823	92.969025
75.900002	101.833000	75.899399	0.769683	92.976318
75.919998	94.666695	75.919395	0.896082	92.983612
75.940002	97.333298	75.939400	1.171089	92.990936
75.959999	99.500000	75.959396	1.673795	92.998322
75.980003	100.167000	75.979401	2.564985	93.005707
76.000000	103.333000	75.999397	4.161759	93.013092
76.019997	100.333000	76.019394	7.083907	93.020538
76.040001	115.500000	76.039398	12.485525	93.027985
76.059998	135.667007	76.059395	22.272610	93.035431
76.080002	153.333008	76.079399	38.879353	93.042938
76.099998	180.000000	76.099396	63.360703	93.050446
76.119995	217.000000	76.119392	91.101181	93.057983
76.139999	226.167007	76.139397	109.233109	93.065552
76.159996	218.833008	76.159393	105.586067	93.073120
76.180000	198.333008	76.179398	83.241074	93.080719
76.199997	178.167007	76.199394	56.599236	93.088348
76.219994	146.333008	76.219391	35.569248	93.095978
76.239998	130.333008	76.239395	21.745153	93.103638

76.259995	118.833000	76.259392	13.280357	93.111298
76.279999	112.667000	76.279396	8.204060	93.118988
76.300003	101.833000	76.299400	5.200599	93.126709
76.320000	102.833000	76.319397	3.475701	93.134430
76.340004	97.000000	76.339401	2.568020	93.142181
76.360001	95.833298	76.359398	2.235937	93.149933
76.380005	100.333000	76.379402	2.401345	93.157715
76.400002	95.333298	76.399406	3.151375	93.165527
76.419998	96.666695	76.419403	4.797700	93.173340
76.440002	112.333000	76.439407	8.015573	93.181152
76.459999	122.833000	76.459404	14.023721	93.188995
76.480003	140.167007	76.479408	24.620260	93.196869
76.500000	152.167007	76.499405	41.346237	93.204742
76.519997	182.667007	76.519402	62.862103	93.212616
76.540001	190.833008	76.539406	81.586273	93.220520
76.559998	185.500000	76.559402	86.313179	93.228424
76.580002	193.333008	76.579407	73.794060	93.236328
76.599998	171.667007	76.599403	53.041653	93.244293
76.619995	145.000000	76.619400	34.291214	93.252228
76.639999	127.500000	76.639404	21.188169	93.260193
76.659996	116.000000	76.659401	12.977268	93.268158
76.680000	104.167000	76.679405	8.012218	93.276154
76.699997	110.667000	76.699402	5.054271	93.284149
76.719994	110.167000	76.719398	3.326554	93.292145
76.739998	100.167000	76.739403	2.368271	93.300171
76.759995	107.167000	76.759399	1.915525	93.308197
76.779999	98.666695	76.779404	1.832197	93.316223
76.800003	97.333298	76.799408	2.078887	93.324249
76.820000	96.666695	76.819405	2.706180	93.332306
76.840004	97.833298	76.839409	3.882841	93.340363
76.860001	98.333298	76.859406	5.972884	93.348419
76.880005	105.667000	76.879410	9.712699	93.356476
76.900002	120.667000	76.899406	16.529757	93.364594
76.919998	152.000000	76.919403	29.057222	93.372681
76.940002	185.167007	76.939407	51.590874	93.380768
76.959999	243.667007	76.959404	89.346909	93.388855
76.980003	293.000000	76.979408	144.123154	93.396973
77.000000	360.832977	76.999405	204.737000	93.405060

77.019997	390.832977	77.019402	242.846634	93.413177
77.040001	381.332977	77.039406	233.779877	93.421295
77.059998	347.666992	77.059402	186.479385	93.429413
77.080002	288.000000	77.079407	132.852783	93.437531
77.099998	238.333008	77.099403	95.133095	93.445679
77.119995	216.167007	77.119400	79.168800	93.453796
77.139999	215.500000	77.139404	84.898689	93.461945
77.159996	264.666992	77.159401	111.176186	93.470062
77.180000	311.000000	77.179405	151.291809	93.478210
77.199997	333.832977	77.199402	185.971970	93.486328
77.219994	321.666992	77.219398	190.629410	93.494507
77.239998	307.832977	77.239403	159.930893	93.502594
77.259995	258.000000	77.259399	113.939484	93.510742
77.279999	203.167007	77.279404	73.514595	93.518890
77.300003	163.000000	77.299408	45.466618	93.527008
77.320000	140.167007	77.319405	27.822565	93.535156
77.340004	125.333000	77.339409	17.040051	93.543274
77.360001	119.667000	77.359406	10.492594	93.551392
77.380005	117.000000	77.379410	6.517066	93.559540
77.400002	113.833000	77.399406	4.105212	93.567657
77.419998	104.167000	77.419403	2.638343	93.575806
77.440002	96.000000	77.439407	1.741650	93.583923
77.459999	98.166695	77.459404	1.191376	93.592010
77.480003	91.500000	77.479416	0.855423	93.600128
77.500000	85.333298	77.499413	0.660605	93.608246
77.519997	81.000000	77.519409	0.571074	93.616333
77.540001	92.666695	77.539413	0.581468	93.624420
77.559998	92.833298	77.559410	0.718555	93.632507
77.580002	90.333298	77.579414	1.051300	93.640594
77.599998	92.500000	77.599411	1.697653	93.648651
77.619995	93.000000	77.619408	2.793116	93.656708
77.639999	96.000000	77.639412	4.345944	93.664795
77.659996	93.500000	77.659409	5.968205	93.672821
77.680000	91.666695	77.679413	6.837098	93.680878
77.699997	95.166695	77.699409	6.365945	93.688904
77.719994	90.666695	77.719406	4.923921	93.696930
77.739998	89.333298	77.739410	3.368582	93.704956
77.759995	93.166695	77.759407	2.198520	93.712952

77.779999	88.833298	77.779411	1.462408	93.720947
77.800003	93.500000	77.799416	1.054262	93.728943
77.820000	86.666695	77.819412	0.879928	93.736908
77.840004	85.500000	77.839417	0.894914	93.744873
77.860001	93.833298	77.859413	1.108546	93.752838
77.880005	86.500000	77.879417	1.598009	93.760742
77.900002	98.333298	77.899414	2.545535	93.768677
77.919998	100.000000	77.919411	4.303121	93.776581
77.940002	98.500000	77.939415	7.448483	93.784485
77.959999	112.167000	77.959412	12.677991	93.792358
77.980003	110.333000	77.979416	20.227629	93.800232
78.000000	119.667000	77.999413	28.633398	93.808075
78.019997	123.333000	78.019409	34.236546	93.815918
78.040001	126.000000	78.039413	33.920025	93.823730
78.059998	135.667007	78.059410	29.015068	93.831543
78.080002	125.667000	78.079414	24.069820	93.839325
78.099998	118.833000	78.099411	22.805090	93.847107
78.119995	137.833008	78.119408	27.524479	93.854858
78.139999	156.833008	78.139412	41.066082	93.862610
78.159996	198.500000	78.159409	68.147636	93.870331
78.180000	263.000000	78.179413	114.463188	93.878021
78.199997	327.832977	78.199409	180.730927	93.885712
78.219994	392.832977	78.219406	251.544952	93.893372
78.239998	428.832977	78.239410	292.254059	93.901031
78.259995	408.832977	78.259407	275.739777	93.908661
78.279999	367.332977	78.279411	214.834793	93.916229
78.300003	306.332977	78.299416	146.104584	93.923859
78.320000	230.333008	78.319412	92.505356	93.931396
78.340004	174.000000	78.339417	57.027817	93.938934
78.360001	167.833008	78.359413	34.964561	93.946442
78.380005	140.833008	78.379417	21.458342	93.953949
78.400002	134.167007	78.399414	13.227861	93.961426
78.419998	131.667007	78.419411	8.219557	93.968872
78.440002	117.333000	78.439415	5.172111	93.976318
78.459999	99.166695	78.459412	3.312457	93.983734
78.480003	96.666695	78.479416	2.166070	93.991089
78.500000	97.666695	78.499413	1.449158	93.998444
78.519997	95.500000	78.519409	0.991739	94.005768

78.540001	93.833298	78.539413	0.693311	94.013092
78.559998	83.666695	78.559418	0.494301	94.020386
78.580002	90.666695	78.579422	0.358730	94.027618
78.599998	90.000000	78.599419	0.264578	94.034851
78.619995	83.500000	78.619415	0.197991	94.042023
78.639999	88.500000	78.639420	0.150121	94.049225
78.659996	87.166695	78.659416	0.115230	94.056366
78.680000	83.500000	78.679420	0.089435	94.063477
78.699997	87.333298	78.699417	0.070143	94.070557
78.719994	84.666695	78.719414	0.055543	94.077637
78.739998	85.666695	78.739418	0.044372	94.084656
78.759995	92.666695	78.759415	0.035747	94.091644
78.779999	82.333298	78.779419	0.029019	94.098633
78.800003	81.833298	78.799423	0.023728	94.105560
78.820000	83.666695	78.819420	0.019534	94.112488
78.840004	89.833298	78.839424	0.016184	94.119354
78.860001	90.666695	78.859421	0.013490	94.126190
78.880005	86.833298	78.879425	0.011308	94.133026
78.900002	87.333298	78.899422	0.009529	94.139801
78.919998	84.500000	78.919418	0.008071	94.146545
78.940002	87.166695	78.939423	0.006867	94.153259
78.959999	85.000000	78.959419	0.005870	94.159943
78.980003	86.500000	78.979424	0.005039	94.166595
79.000000	85.333298	78.999420	0.004343	94.173218
79.019997	85.833298	79.019417	0.003758	94.179810
79.040001	87.500000	79.039421	0.003264	94.186340
79.059998	81.833298	79.059418	0.002844	94.192841
79.080002	83.666695	79.079422	0.002487	94.199310
79.099998	89.166695	79.099419	0.002182	94.205780
79.119995	78.500000	79.119415	0.001920	94.212158
79.139999	79.333298	79.139420	0.001694	94.218536
79.159996	84.333298	79.159416	0.001500	94.224854
79.180000	86.000000	79.179420	0.001331	94.231171
79.199997	77.666695	79.199417	0.001184	94.237396
79.219994	84.166695	79.219414	0.001057	94.243622
79.239998	81.000000	79.239418	0.000945	94.249817
79.259995	84.166695	79.259415	0.000847	94.255951
79.279999	85.833298	79.279419	0.000761	94.262054

79.300003	87.833298	79.299423	0.000686	94.268127
79.320000	89.333298	79.319420	0.000617	94.274139
79.340004	85.833298	79.339424	0.000558	94.280121
79.360001	88.000000	79.359421	0.000506	94.286041
79.380005	84.000000	79.379425	0.000458	94.291931
79.400002	85.166695	79.399422	0.000417	94.297821
79.419998	88.666695	79.419418	0.000381	94.303619
79.440002	78.500000	79.439423	0.000348	94.309418
79.459999	86.333298	79.459419	0.000320	94.315125
79.480003	87.333298	79.479424	0.000294	94.320801
79.500000	83.166695	79.499420	0.000271	94.326447
79.519997	78.833298	79.519417	0.000252	94.332062
79.540001	85.666695	79.539421	0.000234	94.337616
79.559998	81.833298	79.559418	0.000218	94.343140
79.580002	74.166695	79.579422	0.000204	94.348602
79.599998	85.833298	79.599419	0.000187	94.354034
79.619995	85.333298	79.619423	0.000176	94.359406
79.639999	85.500000	79.639427	0.000167	94.364746
79.659996	80.333298	79.659424	0.000159	94.370026
79.680000	83.333298	79.679428	0.000152	94.375275
79.699997	90.666695	79.699425	0.000146	94.380463
79.719994	84.833298	79.719421	0.000142	94.385620
79.739998	90.000000	79.739426	0.000138	94.390717
79.759995	84.000000	79.759422	0.000136	94.395782
79.779999	92.166695	79.779427	0.000135	94.400787
79.800003	91.833298	79.799431	0.000135	94.405762
79.820000	80.333298	79.819427	0.000136	94.410645
79.840004	78.333298	79.839432	0.000138	94.415527
79.860001	89.166695	79.859428	0.000142	94.420319
79.880005	84.666695	79.879433	0.000147	94.425079
79.900002	80.333298	79.899429	0.000154	94.429779
79.919998	88.000000	79.919426	0.000163	94.434448
79.940002	84.666695	79.939430	0.000173	94.439087
79.959999	86.000000	79.959427	0.000187	94.443634
79.980003	81.833298	79.979431	0.000203	94.448151
80.000000	83.833298	79.999428	0.000222	94.452637
80.019997	86.500000	80.019424	0.000245	94.457001
80.040001	81.000000	80.039429	0.000272	94.461365

80.059998	82.333298	80.059425	0.000306	94.465668
80.080002	86.333298	80.079430	0.000345	94.469940
80.099998	91.333298	80.099426	0.000393	94.474152
80.119995	84.500000	80.119423	0.000450	94.478271
80.139999	86.666695	80.139427	0.000518	94.482361
80.159996	85.166695	80.159424	0.000602	94.486420
80.180000	83.333298	80.179428	0.000703	94.490387
80.199997	82.333298	80.199425	0.000827	94.494324
80.219994	88.333298	80.219421	0.000980	94.498199
80.239998	84.666695	80.239426	0.001170	94.502014
80.259995	85.666695	80.259422	0.001406	94.505768
80.279999	85.333298	80.279427	0.001703	94.509521
80.300003	78.000000	80.299431	0.002080	94.513153
80.320000	80.500000	80.319427	0.002561	94.516754
80.340004	90.333298	80.339432	0.003182	94.520294
80.360001	81.833298	80.359428	0.003990	94.523773
80.380005	84.500000	80.379433	0.005055	94.527222
80.400002	93.000000	80.399429	0.006471	94.530579
80.419998	86.666695	80.419426	0.008379	94.533905
80.440002	80.166695	80.439430	0.010985	94.537170
80.459999	80.666695	80.459427	0.014590	94.540344
80.480003	83.333298	80.479431	0.019653	94.543488
80.500000	79.166695	80.499428	0.026865	94.546570
80.519997	83.166695	80.519424	0.037307	94.549591
80.540001	87.000000	80.539429	0.052679	94.552551
80.559998	89.166695	80.559425	0.075709	94.555450
80.580002	82.166695	80.579430	0.110970	94.558319
80.599998	87.166695	80.599426	0.166330	94.561096
80.619995	81.333298	80.619423	0.256112	94.563812
80.639999	80.666695	80.639427	0.407336	94.566498
80.659996	86.333298	80.659431	0.671723	94.569122
80.680000	91.333298	80.679436	1.147209	94.571655
80.699997	84.000000	80.699432	2.008082	94.574127
80.719994	90.500000	80.719429	3.526217	94.576538
80.739998	93.000000	80.739433	6.009731	94.578888
80.759995	102.667000	80.759430	9.510157	94.581207
80.779999	104.333000	80.779434	13.284943	94.583435
80.800003	110.167000	80.799438	15.615631	94.585602

80.820000	98.666695	80.819435	15.049620	94.587738
80.840004	92.166695	80.839439	12.047359	94.589783
80.860001	97.500000	80.859436	8.420707	94.591766
80.880005	95.833298	80.879440	5.464366	94.593658
80.900002	95.500000	80.899437	3.452787	94.595551
80.919998	95.500000	80.919434	2.182839	94.597321
80.940002	95.166695	80.939438	1.407931	94.599060
80.959999	93.000000	80.959435	0.954483	94.600739
80.980003	89.333298	80.979439	0.716298	94.602325
81.000000	90.500000	80.999435	0.637765	94.603912
81.019997	91.166695	81.019432	0.704491	94.605377
81.040001	97.166695	81.039436	0.948202	94.606750
81.059998	94.166695	81.059433	1.465659	94.608124
81.080002	91.666695	81.079437	2.455301	94.609375
81.099998	96.166695	81.099434	4.251070	94.610626
81.119995	102.000000	81.119431	7.286801	94.611755
81.139999	106.167000	81.139435	11.809098	94.612823
81.159996	104.000000	81.159431	17.185236	94.613861
81.180000	108.333000	81.179436	21.356001	94.614777
81.199997	114.833000	81.199432	21.886480	94.615662
81.219994	113.500000	81.219429	18.498575	94.616486
81.239998	104.000000	81.239433	13.412477	94.617249
81.259995	95.666695	81.259430	8.860023	94.617920
81.279999	95.333298	81.279434	5.612856	94.618530
81.300003	95.833298	81.299438	3.511365	94.619080
81.320000	96.666695	81.319435	2.196201	94.619568
81.340004	91.666695	81.339439	1.382339	94.619965
81.360001	88.333298	81.359436	0.884563	94.620300
81.380005	92.000000	81.379440	0.585177	94.620575
81.400002	91.333298	81.399437	0.411110	94.620819
81.419998	86.833298	81.419434	0.317471	94.620941
81.440002	83.166695	81.439438	0.278453	94.621002
81.459999	87.166695	81.459435	0.281259	94.621033
81.480003	92.666695	81.479439	0.322668	94.620941
81.500000	96.500000	81.499435	0.407856	94.620789
81.519997	89.166695	81.519432	0.551578	94.620575
81.540001	84.000000	81.539436	0.782480	94.620331
81.559998	85.166695	81.559433	1.152479	94.619965

81.580002	92.833298	81.579437	1.758027	94.619568
81.599998	89.833298	81.599434	2.780277	94.619049
81.619995	97.166695	81.619431	4.565785	94.618500
81.639999	104.167000	81.639435	7.763352	94.617889
81.659996	114.667000	81.659431	13.500652	94.617157
81.680000	127.500000	81.679436	23.479050	94.616425
81.699997	154.000000	81.699440	39.468132	94.615570
81.719994	181.167007	81.719437	61.432404	94.614685
81.739998	192.333008	81.739441	84.585197	94.613678
81.759995	212.333008	81.759438	99.459373	94.612640
81.779999	207.167007	81.779442	99.823067	94.611511
81.800003	196.000000	81.799446	89.725349	94.610321
81.820000	183.333008	81.819443	77.142036	94.609070
81.840004	164.167007	81.839447	64.496239	94.607727
81.860001	149.167007	81.859444	50.555790	94.606323
81.880005	135.333008	81.879448	36.212910	94.604828
81.900002	125.000000	81.899445	24.067202	94.603302
81.919998	117.667000	81.919441	15.344715	94.601685
81.940002	106.000000	81.939445	9.635405	94.599976
81.959999	104.500000	81.959442	6.040061	94.598206
81.980003	98.166695	81.979446	3.800414	94.596375
82.000000	94.166695	81.999443	2.411585	94.594482
82.019997	99.000000	82.019440	1.551772	94.592468
82.040001	93.666695	82.039444	1.019977	94.590424
82.059998	85.500000	82.059441	0.691774	94.588287
82.080002	87.833298	82.079445	0.490450	94.586090
82.099998	89.166695	82.099442	0.370516	94.583832
82.119995	88.333298	82.119438	0.305874	94.581482
82.139999	87.500000	82.139442	0.283471	94.579071
82.159996	91.500000	82.159439	0.299849	94.576569
82.180000	86.000000	82.179443	0.360618	94.574005
82.199997	84.333298	82.199440	0.483214	94.571350
82.219994	79.166695	82.219437	0.705044	94.568634
82.239998	87.333298	82.239441	1.100745	94.565857
82.259995	91.500000	82.259438	1.813634	94.563019
82.279999	95.500000	82.279442	3.107662	94.560089
82.300003	91.500000	82.299446	5.417792	94.557068
82.320000	102.833000	82.319443	9.316800	94.553986

82.340004	106.500000	82.339447	15.191566	94.550842
82.360001	115.833000	82.359444	22.392946	94.547638
82.380005	117.167000	82.379448	28.433687	94.544342
82.400002	121.833000	82.399445	30.002083	94.540955
82.419998	126.667000	82.419441	26.176535	94.537506
82.440002	109.000000	82.439445	19.515196	94.533966
82.459999	97.666695	82.459442	13.155865	94.530426
82.480003	92.666695	82.479446	8.453560	94.526733
82.500000	93.666695	82.499443	5.355416	94.522980
82.519997	87.666695	82.519440	3.403250	94.519196
82.540001	90.333298	82.539444	2.202170	94.515289
82.559998	92.166695	82.559441	1.490500	94.511322
82.580002	89.833298	82.579445	1.106865	94.507294
82.599998	93.833298	82.599442	0.963503	94.503174
82.619995	88.000000	82.619438	1.032301	94.498993
82.639999	91.500000	82.639442	1.350225	94.494720
82.659996	93.666695	82.659439	2.043264	94.490417
82.680000	93.166695	82.679443	3.374298	94.486023
82.699997	92.833298	82.699440	5.789186	94.481537
82.719994	105.667000	82.719437	9.878890	94.476990
82.739998	107.500000	82.739449	16.029999	94.472351
82.759995	120.500000	82.759445	23.527744	94.467682
82.779999	122.500000	82.779449	29.757200	94.462891
82.800003	124.333000	82.799454	31.291897	94.458038
82.820000	122.333000	82.819450	27.234713	94.453156
82.840004	119.667000	82.839455	20.285322	94.448151
82.860001	115.167000	82.859451	13.688734	94.443085
82.880005	99.500000	82.879456	8.822133	94.437958
82.900002	98.500000	82.899452	5.617075	94.432709
82.919998	97.000000	82.919449	3.598227	94.427460
82.940002	92.000000	82.939453	2.359491	94.422089
82.959999	95.666695	82.959450	1.632174	94.416656
82.980003	94.666695	82.979454	1.250230	94.411133
83.000000	101.333000	82.999451	1.122304	94.405548
83.019997	93.666695	83.019447	1.215973	94.399902
83.040001	96.333298	83.039452	1.560355	94.394196
83.059998	87.166695	83.059448	2.267102	94.388367
83.080002	97.000000	83.079453	3.581891	94.382507

83.099998	98.333298	83.099449	5.964470	94.376587
83.119995	101.167000	83.119446	10.175534	94.370575
83.139999	115.167000	83.139450	17.224869	94.364471
83.159996	130.667007	83.159447	27.855396	94.358307
83.180000	147.500000	83.179451	41.386490	94.352051
83.199997	174.500000	83.199448	54.691334	94.345734
83.219994	174.000000	83.219444	63.472015	94.339355
83.239998	164.500000	83.239449	65.180435	94.332916
83.259995	171.667007	83.259445	59.980553	94.326385
83.279999	160.500000	83.279449	50.336300	94.319794
83.300003	158.667007	83.299454	40.722153	94.313141
83.320000	154.833008	83.319450	35.670090	94.306366
83.340004	157.500000	83.339455	38.313068	94.299561
83.360001	183.000000	83.359451	51.040359	94.292664
83.380005	226.000000	83.379456	75.538063	94.285736
83.400002	258.832977	83.399452	109.692314	94.278717
83.419998	296.166992	83.419449	142.918793	94.271606
83.440002	301.832977	83.439453	157.871384	94.264404
83.459999	294.000000	83.459450	144.789841	94.257172
83.480003	264.000000	83.479454	112.478409	94.249878
83.500000	219.167007	83.499451	77.844124	94.242493
83.519997	168.333008	83.519447	50.672371	94.235016
83.540001	150.833008	83.539452	32.178864	94.227509
83.559998	122.500000	83.559448	20.270641	94.219879
83.580002	122.833000	83.579453	12.729093	94.212250
83.599998	121.833000	83.599449	7.993558	94.204498
83.619995	111.667000	83.619446	5.038939	94.196686
83.639999	108.667000	83.639450	3.205088	94.188812
83.659996	100.000000	83.659447	2.069587	94.180878
83.680000	95.000000	83.679451	1.363102	94.172882
83.699997	96.166695	83.699448	0.919526	94.164764
83.719994	91.333298	83.719444	0.636894	94.156616
83.739998	89.333298	83.739456	0.453831	94.148376
83.759995	86.333298	83.759453	0.333834	94.140106
83.779999	86.000000	83.779457	0.254594	94.131714
83.800003	92.833298	83.799461	0.202780	94.123291
83.820000	91.500000	83.819458	0.170305	94.114807
83.840004	87.333298	83.839462	0.152362	94.106232

83.860001	84.833298	83.859459	0.146391	94.097595
83.880005	85.000000	83.879463	0.151451	94.088898
83.900002	90.666695	83.899460	0.168021	94.080139
83.919998	93.000000	83.919456	0.198061	94.071289
83.940002	89.833298	83.939461	0.245369	94.062378
83.959999	88.500000	83.959457	0.316184	94.053406
83.980003	89.333298	83.979462	0.420527	94.044373
84.000000	93.000000	83.999458	0.573976	94.035248
84.019997	86.000000	84.019455	0.801355	94.026093
84.040001	93.000000	84.039459	1.142908	94.016846
84.059998	99.833298	84.059456	1.665360	94.007538
84.080002	96.500000	84.079460	2.485676	93.998199
84.099998	102.167000	84.099457	3.814151	93.988739
84.119995	105.333000	84.119453	6.043445	93.979218
84.139999	112.667000	84.139458	9.908238	93.969666
84.159996	130.167007	84.159454	16.732424	93.960052
84.180000	146.000000	84.179459	28.740843	93.950348
84.199997	177.000000	84.199455	49.085651	93.940582
84.219994	227.500000	84.219452	80.897392	93.930756
84.239998	281.000000	84.239456	124.135178	93.920868
84.259995	330.332977	84.259453	170.610229	93.910919
84.279999	341.000000	84.279457	203.627426	93.900909
84.300003	356.832977	84.299461	209.707550	93.890808
84.320000	318.332977	84.319458	191.609909	93.880676
84.340004	296.000000	84.339462	161.334473	93.870483
84.360001	255.667007	84.359459	126.733368	93.860199
84.380005	214.500000	84.379463	92.263191	93.849884
84.400002	184.000000	84.399460	62.707294	93.839478
84.419998	164.500000	84.419456	40.757645	93.829010
84.440002	149.667007	84.439461	25.941639	93.818512
84.459999	145.167007	84.459457	16.401550	93.807953
84.480003	126.333000	84.479462	10.362858	93.797302
84.500000	121.000000	84.499458	6.571082	93.786621
84.519997	111.833000	84.519455	4.198411	93.775848
84.540001	110.333000	84.539459	2.715067	93.765045
84.559998	100.333000	84.559456	1.785739	93.754150
84.580002	95.000000	84.579460	1.198475	93.743225
84.599998	92.166695	84.599457	0.823095	93.732239

84.619995	95.333298	84.619453	0.579508	93.721191
84.639999	92.000000	84.639458	0.419283	93.710052
84.659996	89.666695	84.659454	0.313363	93.698883
84.680000	94.500000	84.679459	0.244258	93.687653
84.699997	91.166695	84.699455	0.202090	93.676392
84.719994	85.000000	84.719452	0.182161	93.665070
84.739998	82.666695	84.739456	0.184481	93.653656
84.759995	86.666695	84.759460	0.214802	93.642181
84.779999	87.833298	84.779465	0.287853	93.630646
84.800003	88.666695	84.799469	0.433549	93.619110
84.820000	87.666695	84.819466	0.705564	93.607483
84.840004	88.833298	84.839470	1.186880	93.595795
84.860001	89.333298	84.859467	1.969559	93.584076
84.880005	91.666695	84.879471	3.074022	93.572296
84.900002	90.333298	84.899467	4.293923	93.560455
84.919998	90.666695	84.919464	5.134889	93.548553
84.940002	95.500000	84.939468	5.119796	93.536591
84.959999	93.500000	84.959465	4.282492	93.524628
84.980003	90.833298	84.979469	3.127256	93.512543
85.000000	84.666695	84.999466	2.107756	93.500427
85.019997	85.333298	85.019463	1.373827	93.488281
85.040001	84.666695	85.039467	0.890781	93.476074
85.059998	90.333298	85.059464	0.585159	93.463837
85.080002	84.000000	85.079468	0.397687	93.451508
85.099998	84.333298	85.099464	0.289535	93.439148
85.119995	83.333298	85.119461	0.236537	93.426727
85.139999	82.666695	85.139465	0.225349	93.414276
85.159996	90.833298	85.159462	0.251386	93.401764
85.180000	85.500000	85.179466	0.318886	93.389191
85.199997	90.333298	85.199463	0.443480	93.376587
85.219994	84.500000	85.219460	0.658996	93.363922
85.239998	88.166695	85.239464	1.030316	93.351227
85.259995	94.500000	85.259460	1.672331	93.338470
85.279999	88.333298	85.279465	2.770217	93.325684
85.300003	94.333298	85.299469	4.565400	93.312836
85.320000	105.667000	85.319466	7.244811	93.299957
85.340004	99.833298	85.339470	10.701970	93.286987
85.360001	105.167000	85.359467	14.390793	93.274017

85.380005	107.667000	85.379471	17.873333	93.260986
85.400002	124.167000	85.399467	21.916906	93.247894
85.419998	132.833008	85.419464	28.625389	93.234802
85.440002	158.833008	85.439468	39.724895	93.221649
85.459999	177.500000	85.459465	54.083431	93.208435
85.480003	200.000000	85.479469	66.313774	93.195190
85.500000	189.667007	85.499466	69.309464	93.181885
85.519997	180.167007	85.519463	60.882179	93.168579
85.540001	159.833008	85.539467	46.118221	93.155212
85.559998	145.833008	85.559464	31.680920	93.141815
85.580002	124.000000	85.579468	20.713402	93.128326
85.599998	109.167000	85.599464	13.289672	93.114838
85.619995	103.333000	85.619461	8.476339	93.101318
85.639999	103.000000	85.639465	5.406901	93.087738
85.659996	99.166695	85.659462	3.479719	93.074127
85.680000	102.167000	85.679466	2.298877	93.060455
85.699997	97.833298	85.699463	1.615684	93.046783
85.719994	98.666695	85.719460	1.284953	93.033051
85.739998	94.833298	85.739471	1.248219	93.019287
85.759995	90.500000	85.759468	1.529373	93.005493
85.779999	95.833298	85.779472	2.236897	92.991669
85.800003	93.000000	85.799477	3.541776	92.977783
85.820000	97.166695	85.819473	5.563108	92.963867
85.840004	95.666695	85.839478	8.100240	92.949921
85.860001	102.833000	85.859474	10.348140	92.935944
85.880005	103.500000	85.879478	11.165262	92.921936
85.900002	98.500000	85.899475	10.070461	92.907898
85.919998	98.666695	85.919472	7.783518	92.893829
85.940002	97.333298	85.939476	5.415149	92.879730
85.959999	88.833298	85.959473	3.566290	92.865570
85.980003	86.000000	85.979477	2.295985	92.851410
86.000000	92.666695	85.999474	1.465681	92.837219
86.019997	80.833298	86.019470	0.931653	92.822968
86.040001	91.166695	86.039474	0.591136	92.808716
86.059998	85.500000	86.059471	0.376027	92.794403
86.080002	93.500000	86.079475	0.241008	92.780090
86.099998	85.333298	86.099472	0.156585	92.765778
86.119995	83.000000	86.119469	0.103614	92.751373

86.139999	82.000000	86.139473	0.070047	92.736969
86.159996	88.333298	86.159470	0.048464	92.722565
86.180000	81.500000	86.179474	0.034305	92.708099
86.199997	81.666695	86.199471	0.024826	92.693604
86.219994	86.000000	86.219467	0.018343	92.679108
86.239998	89.000000	86.239471	0.013823	92.664581
86.259995	89.166695	86.259468	0.010619	92.650024
86.279999	86.333298	86.279472	0.008313	92.635437
86.300003	82.666695	86.299477	0.006637	92.620850
86.320000	87.000000	86.319473	0.005407	92.606232
86.340004	89.166695	86.339478	0.004501	92.591583
86.360001	85.333298	86.359474	0.003835	92.576935
86.380005	89.166695	86.379478	0.003348	92.562225
86.400002	89.333298	86.399475	0.003001	92.547516
86.419998	87.833298	86.419472	0.002764	92.532776
86.440002	91.500000	86.439476	0.002617	92.518036
86.459999	96.833298	86.459473	0.002546	92.503265
86.480003	95.833298	86.479477	0.002542	92.488495
86.500000	92.000000	86.499474	0.002601	92.473663
86.519997	86.000000	86.519470	0.002719	92.458893
86.540001	92.500000	86.539474	0.002899	92.444031
86.559998	100.500000	86.559471	0.003143	92.429169
86.580002	99.000000	86.579475	0.003458	92.414337
86.599998	91.666695	86.599472	0.003852	92.399414
86.619995	86.166695	86.619469	0.004337	92.384552
86.639999	91.000000	86.639473	0.004930	92.369629
86.659996	91.333298	86.659470	0.005651	92.354706
86.680000	92.333298	86.679474	0.006527	92.339752
86.699997	94.000000	86.699471	0.007591	92.324829
86.719994	89.500000	86.719467	0.008887	92.309875
86.739998	92.000000	86.739479	0.010470	92.294891
86.759995	94.000000	86.759476	0.012414	92.279907
86.779999	87.333298	86.779480	0.014810	92.264923
86.800003	87.500000	86.799484	0.017783	92.249908
86.820000	96.666695	86.819481	0.021494	92.234894
86.840004	90.666695	86.839485	0.026159	92.219879
86.860001	91.500000	86.859482	0.032060	92.204865
86.880005	85.333298	86.879486	0.039593	92.189819

86.900002	94.666695	86.899483	0.049281	92.174805
86.919998	93.333298	86.919479	0.061862	92.159760
86.940002	90.500000	86.939484	0.078365	92.144714
86.959999	92.166695	86.959480	0.100226	92.129669
86.980003	91.666695	86.979485	0.129528	92.114594
87.000000	88.833298	86.999481	0.169238	92.099518
87.019997	91.000000	87.019478	0.223738	92.084473
87.040001	89.666695	87.039482	0.299528	92.069427
87.059998	87.500000	87.059479	0.406240	92.054352
87.080002	93.333298	87.079483	0.558686	92.039307
87.099998	99.333298	87.099480	0.779546	92.024231
87.119995	96.500000	87.119476	1.105089	92.009186
87.139999	102.000000	87.139481	1.595121	91.994110
87.159996	99.833298	87.159477	2.351275	91.979034
87.180000	101.667000	87.179482	3.555508	91.963959
87.199997	99.333298	87.199478	5.536973	91.948914
87.219994	114.833000	87.219475	8.896962	91.933868
87.239998	123.167000	87.239479	14.696943	91.918823
87.259995	143.167007	87.259476	24.657024	91.903778
87.279999	178.667007	87.279480	41.202072	91.888733
87.300003	232.500000	87.299484	66.809799	91.873688
87.320000	284.000000	87.319481	102.180199	91.858673
87.340004	332.666992	87.339485	143.863632	91.843658
87.360001	372.666992	87.359482	184.327911	91.828644
87.380005	384.166992	87.379486	216.506012	91.813629
87.400002	372.500000	87.399483	235.268005	91.798645
87.419998	357.166992	87.419479	233.624069	91.783661
87.440002	363.832977	87.439484	207.169952	91.768677
87.459999	319.832977	87.459480	164.351746	91.753723
87.480003	280.000000	87.479485	121.239731	91.738800
87.500000	250.667007	87.499481	87.758667	91.723846
87.519997	232.500000	87.519478	64.222939	91.708923
87.540001	192.167007	87.539482	46.941593	91.694000
87.559998	173.167007	87.559479	33.282509	91.679108
87.580002	157.000000	87.579483	22.619482	91.664215
87.599998	134.000000	87.599480	14.909804	91.649353
87.619995	125.833000	87.619476	9.712370	91.634491
87.639999	116.833000	87.639481	6.344715	91.619659

87.659996	112.000000	87.659477	4.203120	91.604828
87.680000	105.500000	87.679482	2.857394	91.590057
87.699997	105.000000	87.699478	2.032255	91.575256
87.719994	98.500000	87.719482	1.556071	91.560486
87.739998	92.333298	87.739487	1.329599	91.545746
87.759995	101.833000	87.759483	1.304980	91.531036
87.779999	95.166695	87.779488	1.478579	91.516327
87.800003	90.333298	87.799492	1.896122	91.501617
87.820000	91.500000	87.819489	2.675509	91.487000
87.840004	105.333000	87.839493	4.061342	91.472351
87.860001	103.333000	87.859489	6.522193	91.457733
87.880005	108.333000	87.879494	10.912522	91.443146
87.900002	130.333008	87.899490	18.631016	91.428619
87.919998	152.500000	87.919487	31.583969	91.414093
87.940002	178.500000	87.939491	51.365467	91.399567
87.959999	210.000000	87.959488	76.946968	91.385101
87.980003	223.000000	87.979492	101.820221	91.370636
88.000000	220.167007	87.999489	114.867035	91.356232
88.019997	220.833008	88.019485	108.866615	91.341827
88.040001	210.333008	88.039490	88.066139	91.327454
88.059998	182.667007	88.059486	63.463787	91.313110
88.080002	159.333008	88.079491	42.811348	91.298798
88.099998	142.667007	88.099487	28.055058	91.284515
88.119995	128.667007	88.119484	18.186911	91.270294
88.139999	116.167000	88.139488	11.745874	91.256073
88.159996	114.667000	88.159485	7.603860	91.241913
88.180000	114.000000	88.179489	4.984008	91.227753
88.199997	110.667000	88.199486	3.371949	91.213684
88.219994	100.000000	88.219482	2.426837	91.199615
88.239998	99.166695	88.239487	1.934522	91.185577
88.259995	101.833000	88.259483	1.773885	91.171600
88.279999	99.666695	88.279488	1.896003	91.157623
88.300003	100.333000	88.299492	2.318067	91.143707
88.320000	95.833298	88.319489	3.131232	91.129822
88.340004	101.000000	88.339493	4.530177	91.115997
88.360001	108.333000	88.359489	6.861468	91.102203
88.380005	119.000000	88.379494	10.703030	91.088440
88.400002	123.000000	88.399490	16.959385	91.074707

88.419998	145.667007	88.419487	27.065886	91.061035
88.440002	181.333008	88.439491	43.440552	91.047394
88.459999	212.667007	88.459488	70.057793	91.033783
88.480003	279.832977	88.479492	111.934952	91.020264
88.500000	353.500000	88.499489	170.885651	91.006775
88.519997	426.000000	88.519485	237.694016	90.993317
88.540001	451.332977	88.539490	287.987061	90.979919
88.559998	460.332977	88.559486	295.313599	90.966553
88.580002	435.000000	88.579491	256.250061	90.953217
88.599998	370.832977	88.599487	194.058777	90.939972
88.619995	291.666992	88.619484	134.594315	90.926758
88.639999	243.833008	88.639488	89.291748	90.913605
88.659996	194.500000	88.659485	58.150768	90.900482
88.680000	164.000000	88.679497	37.522835	90.887390
88.699997	148.833008	88.699493	24.074415	90.874390
88.719994	129.333008	88.719490	15.388129	90.861420
88.739998	149.000000	88.739494	9.835457	90.848511
88.759995	137.500000	88.759491	6.321198	90.835663
88.779999	127.333000	88.779495	4.106389	90.822876
88.800003	112.333000	88.799500	2.709723	90.810089
88.820000	105.167000	88.819496	1.822495	90.797424
88.840004	106.333000	88.839500	1.250785	90.784790
88.860001	96.000000	88.859497	0.876092	90.772217
88.880005	92.666695	88.879501	0.625393	90.759674
88.900002	84.000000	88.899498	0.454368	90.747253
88.919998	83.166695	88.919495	0.335385	90.734833
88.940002	85.000000	88.939499	0.251125	90.722504
88.959999	88.333298	88.959496	0.190566	90.710236
88.980003	90.000000	88.979500	0.146406	90.697998
89.000000	85.000000	88.999496	0.113839	90.685822
89.019997	91.333298	89.019493	0.089561	90.673737
89.040001	88.833298	89.039497	0.071303	90.661682
89.059998	87.833298	89.059494	0.057499	90.649719
89.080002	89.500000	89.079498	0.047028	90.637817
89.099998	78.333298	89.099495	0.039115	90.625977
89.119995	83.333298	89.119492	0.033207	90.614197
89.139999	88.833298	89.139496	0.028930	90.602478
89.159996	86.166695	89.159492	0.026051	90.590851

89.180000	91.166695	89.179497	0.024449	90.579254
89.199997	87.000000	89.199493	0.024120	90.567749
89.219994	87.666695	89.219490	0.025179	90.556305
89.239998	84.666695	89.239494	0.027896	90.544952
89.259995	91.166695	89.259491	0.032745	90.533630
89.279999	93.833298	89.279495	0.040495	90.522400
89.300003	93.500000	89.299500	0.052355	90.511230
89.320000	89.000000	89.319496	0.070219	90.500153
89.340004	84.500000	89.339500	0.097139	90.489136
89.360001	87.166695	89.359497	0.138115	90.478180
89.380005	87.666695	89.379501	0.201841	90.467316
89.400002	83.833298	89.399498	0.303856	90.456543
89.419998	83.166695	89.419495	0.473078	90.445831
89.440002	85.666695	89.439499	0.763526	90.435181
89.459999	92.333298	89.459496	1.272641	90.424591
89.480003	89.166695	89.479500	2.165274	90.414124
89.500000	89.333298	89.499496	3.678736	90.403717
89.519997	96.166695	89.519493	6.057759	90.393372
89.540001	92.833298	89.539497	9.322357	90.383118
89.559998	96.500000	89.559494	12.876268	90.372955
89.580002	96.500000	89.579498	15.387450	90.362854
89.599998	96.500000	89.599495	15.556565	90.352844
89.619995	96.500000	89.619492	13.362891	90.342896
89.639999	91.000000	89.639503	10.075110	90.333038
89.659996	95.000000	89.659500	6.995883	90.323273
89.680000	84.333298	89.679504	4.661901	90.313599
89.699997	80.666695	89.699501	3.053942	90.303986
89.719994	82.500000	89.719498	1.984297	90.294464
89.739998	79.666695	89.739502	1.282954	90.285034
89.759995	83.000000	89.759499	0.829312	90.275696
89.779999	82.666695	89.779503	0.540403	90.266418
89.800003	80.833298	89.799507	0.360508	90.257233
89.820000	81.166695	89.819504	0.252632	90.248169
89.840004	81.000000	89.839508	0.193553	90.239166
89.860001	86.666695	89.859505	0.170887	90.230255
89.880005	81.333298	89.879509	0.181483	90.221436
89.900002	79.666695	89.899506	0.232186	90.212738
89.919998	80.666695	89.919502	0.342790	90.204071

89.940002	82.833298	89.939507	0.550287	90.195557
89.959999	88.166695	89.959503	0.908399	90.187073
89.980003	86.166695	89.979507	1.469654	90.178711
90.000000	93.000000	89.999504	2.226772	90.170441
90.019997	77.833298	90.019501	3.026823	90.162262
90.040001	74.333298	90.039505	3.557099	90.154205
90.059998	83.666695	90.059502	3.540432	90.146210
90.080002	83.833298	90.079506	3.004521	90.138306
90.099998	83.833298	90.099503	2.250219	90.130524
90.119995	81.000000	90.119499	1.559243	90.122833
90.139999	92.000000	90.139503	1.039971	90.115204
90.159996	80.000000	90.159500	0.682632	90.107697
90.180000	85.333298	90.179504	0.444325	90.100311
90.199997	76.666695	90.199501	0.287637	90.092987
90.219994	76.833298	90.219498	0.185722	90.085785
90.239998	84.333298	90.239502	0.120220	90.078674
90.259995	80.000000	90.259499	0.078628	90.071686
90.279999	86.333298	90.279503	0.052414	90.064758
90.300003	84.833298	90.299507	0.035980	90.057953
90.320000	84.166695	90.319504	0.025713	90.051239
90.340004	80.000000	90.339508	0.019338	90.044647
90.360001	84.166695	90.359505	0.015471	90.038177
90.380005	86.500000	90.379509	0.013273	90.031769
90.400002	83.333298	90.399506	0.012253	90.025452
90.419998	76.666695	90.419502	0.012139	90.019287
90.440002	86.166695	90.439507	0.012796	90.013214
90.459999	75.000000	90.459503	0.014195	90.007233
90.480003	74.666695	90.479507	0.016387	90.001373
90.500000	77.333298	90.499504	0.019501	89.995605
90.519997	85.000000	90.519501	0.023757	89.989960
90.540001	81.166695	90.539505	0.029489	89.984406
90.559998	80.333298	90.559502	0.037180	89.978973
90.580002	80.333298	90.579506	0.047538	89.973663
90.599998	76.500000	90.599510	0.061577	89.968414
90.619995	91.000000	90.619507	0.080766	89.963318
90.639999	88.833298	90.639511	0.107281	89.958344
90.659996	83.000000	90.659508	0.144290	89.953461
90.680000	78.666695	90.679512	0.196570	89.948700

90.699997	85.333298	90.699509	0.271223	89.944031
90.719994	86.333298	90.719505	0.379191	89.939484
90.739998	80.166695	90.739510	0.537713	89.935028
90.759995	88.500000	90.759506	0.774653	89.930725
90.779999	84.500000	90.779510	1.137967	89.926514
90.800003	79.333298	90.799515	1.712870	89.922424
90.820000	85.500000	90.819511	2.656055	89.918457
90.840004	93.333298	90.839516	4.259934	89.914581
90.860001	101.667000	90.859512	7.051394	89.910858
90.880005	108.667000	90.879517	11.927153	89.907227
90.900002	124.500000	90.899513	20.204266	89.903717
90.919998	145.333008	90.919510	33.333790	89.900330
90.940002	164.333008	90.939514	51.752193	89.897064
90.959999	184.667007	90.959511	72.745247	89.893890
90.980003	186.667007	90.979515	89.282639	89.890869
91.000000	191.833008	90.999512	93.328201	89.887939
91.019997	188.333008	91.019508	82.987335	89.885162
91.040001	177.833008	91.039513	64.453400	89.882477
91.059998	149.167007	91.059509	45.721970	89.879913
91.080002	141.833008	91.079514	30.910669	89.877502
91.099998	127.500000	91.099510	20.462305	89.875183
91.119995	110.167000	91.119507	13.406603	89.872986
91.139999	110.833000	91.139511	8.719316	89.870911
91.159996	100.333000	91.159508	5.643774	89.868988
91.180000	100.833000	91.179512	3.650201	89.867157
91.199997	103.000000	91.199509	2.376324	89.865479
91.219994	97.333298	91.219505	1.572191	89.863922
91.239998	88.666695	91.239510	1.070990	89.862457
91.259995	88.500000	91.259506	0.765228	89.861115
91.279999	82.666695	91.279510	0.587895	89.859924
91.300003	81.000000	91.299515	0.500893	89.858856
91.320000	85.666695	91.319511	0.486674	89.857941
91.340004	86.500000	91.339516	0.545169	89.857117
91.360001	81.500000	91.359512	0.695327	89.856415
91.380005	89.000000	91.379517	0.982190	89.855865
91.400002	86.666695	91.399513	1.487890	89.855438
91.419998	89.833298	91.419510	2.342891	89.855133
91.440002	90.333298	91.439514	3.718492	89.854950

91.459999	94.000000	91.459511	5.769284	89.854919
91.480003	93.000000	91.479515	8.536192	89.854980
91.500000	102.500000	91.499512	11.925881	89.855225
91.519997	112.500000	91.519508	16.011526	89.855560
91.540001	115.000000	91.539520	21.447947	89.856049
91.559998	125.833000	91.559517	29.133745	89.856659
91.580002	143.333008	91.579521	38.801140	89.857391
91.599998	143.167007	91.599518	47.654102	89.858246
91.619995	139.833008	91.619514	51.322865	89.859253
91.639999	151.500000	91.639519	47.465794	89.860382
91.659996	132.500000	91.659515	38.184097	89.861664
91.680000	134.500000	91.679520	27.742943	89.863068
91.699997	116.167000	91.699516	19.017601	89.864594
91.719994	107.167000	91.719513	12.688979	89.866272
91.739998	103.167000	91.739517	8.360250	89.868073
91.759995	96.666695	91.759514	5.464281	89.869995
91.779999	96.333298	91.779518	3.547490	89.872101
91.800003	90.333298	91.799522	2.294155	89.874298
91.820000	91.333298	91.819519	1.484704	89.876617
91.840004	89.166695	91.839523	0.966542	89.879120
91.860001	88.500000	91.859520	0.636680	89.881714
91.880005	85.666695	91.879524	0.426242	89.884491
91.900002	83.666695	91.899521	0.291079	89.887360
91.919998	83.833298	91.919518	0.203126	89.890381
91.940002	84.666695	91.939522	0.144981	89.893555
91.959999	88.000000	91.959518	0.105963	89.896851
91.980003	83.833298	91.979523	0.079424	89.900299
92.000000	84.666695	91.999519	0.061281	89.903870
92.019997	87.333298	92.019516	0.048966	89.907562
92.040001	86.666695	92.039520	0.040889	89.911438
92.059998	79.000000	92.059517	0.036102	89.915436
92.080002	80.500000	92.079521	0.034097	89.919556
92.099998	89.333298	92.099518	0.034727	89.923828
92.119995	82.666695	92.119514	0.038175	89.928253
92.139999	89.833298	92.139519	0.045000	89.932770
92.159996	81.833298	92.159515	0.056229	89.937469
92.180000	83.333298	92.179520	0.073592	89.942322
92.199997	78.333298	92.199516	0.099875	89.947296

92.219994	81.500000	92.219513	0.139699	89.952393
92.239998	76.666695	92.239517	0.200958	89.957672
92.259995	79.833298	92.259514	0.297474	89.963043
92.279999	78.500000	92.279518	0.454402	89.968597
92.300003	79.333298	92.299522	0.717146	89.974274
92.320000	83.333298	92.319519	1.165458	89.980072
92.340004	85.000000	92.339523	1.931073	89.986053
92.360001	92.500000	92.359520	3.203577	89.992157
92.380005	99.500000	92.379524	5.201590	89.998383
92.400002	99.166695	92.399521	8.058749	90.004791
92.419998	103.167000	92.419518	11.631210	90.011322
92.440002	107.167000	92.439522	15.283998	90.017975
92.459999	111.833000	92.459518	17.860384	90.024811
92.480003	112.667000	92.479530	18.194065	90.031769
92.500000	111.500000	92.499527	16.071363	90.038879
92.519997	97.000000	92.519524	12.543014	90.046112
92.540001	98.000000	92.539528	8.977460	90.053497
92.559998	93.166695	92.559525	6.127187	90.061066
92.580002	86.666695	92.579529	4.090161	90.068726
92.599998	88.833298	92.599525	2.700524	90.076569
92.619995	83.500000	92.619522	1.768586	90.084534
92.639999	86.166695	92.639526	1.150574	90.092621
92.659996	82.166695	92.659523	0.746068	90.100891
92.680000	83.000000	92.679527	0.484285	90.109283
92.699997	77.666695	92.699524	0.316527	90.117828
92.719994	85.500000	92.719521	0.209388	90.126526
92.739998	78.500000	92.739525	0.140778	90.135345
92.759995	84.500000	92.759521	0.096499	90.144348
92.779999	80.666695	92.779526	0.067509	90.153473
92.800003	77.500000	92.799530	0.048217	90.162750
92.820000	83.666695	92.819527	0.035154	90.172150
92.840004	81.000000	92.839531	0.026151	90.181732
92.860001	78.666695	92.859528	0.019865	90.191437
92.880005	76.166695	92.879532	0.015430	90.201263
92.900002	79.333298	92.899529	0.012297	90.211273
92.919998	81.666695	92.919525	0.010100	90.221405
92.940002	78.000000	92.939529	0.008601	90.231720
92.959999	82.166695	92.959526	0.007649	90.242126

92.980003	88.833298	92.979530	0.007148	90.252716
93.000000	76.166695	92.999527	0.007052	90.263458
93.019997	77.833298	93.019524	0.007349	90.274323
93.040001	86.666695	93.039528	0.008067	90.285339
93.059998	86.833298	93.059525	0.009274	90.296478
93.080002	86.333298	93.079529	0.011088	90.307800
93.099998	87.500000	93.099525	0.013691	90.319275
93.119995	84.000000	93.119522	0.017360	90.330872
93.139999	82.666695	93.139526	0.022506	90.342621
93.159996	86.833298	93.159523	0.029732	90.354492
93.180000	87.166695	93.179527	0.039941	90.366547
93.199997	81.166695	93.199524	0.054465	90.378754
93.219994	81.666695	93.219521	0.075338	90.391052
93.239998	77.000000	93.239525	0.105727	90.403534
93.259995	88.833298	93.259521	0.150692	90.416168
93.279999	87.333298	93.279526	0.218840	90.428925
93.300003	84.000000	93.299530	0.325285	90.441803
93.320000	86.000000	93.319527	0.497521	90.454895
93.340004	94.000000	93.339531	0.786409	90.468109
93.360001	84.166695	93.359528	1.283065	90.481445
93.380005	90.166695	93.379532	2.142808	90.494965
93.400002	91.666695	93.399529	3.597714	90.508575
93.419998	91.833298	93.419533	5.922076	90.522369
93.440002	96.000000	93.439537	9.259628	90.536285
93.459999	106.667000	93.459534	13.280600	90.550354
93.480003	108.833000	93.479538	16.898645	90.564575
93.500000	107.333000	93.499535	18.617224	90.578949
93.519997	106.333000	93.519531	17.686386	90.593445
93.540001	108.167000	93.539536	14.794174	90.608093
93.559998	101.000000	93.559532	11.309937	90.622894
93.580002	100.667000	93.579536	8.174036	90.637848
93.599998	89.666695	93.599533	5.698771	90.652924
93.619995	84.333298	93.619530	3.872393	90.668152
93.639999	86.166695	93.639534	2.584212	90.683533
93.659996	92.000000	93.659531	1.705168	90.699036
93.680000	85.666695	93.679535	1.117923	90.714691
93.699997	84.666695	93.699532	0.732122	90.730499
93.719994	86.000000	93.719528	0.481845	90.746429

93.739998	86.166695	93.739532	0.321316	90.762543
93.759995	86.000000	93.759529	0.219635	90.778778
93.779999	82.333298	93.779533	0.156246	90.795135
93.800003	83.833298	93.799538	0.118129	90.811646
93.820000	76.500000	93.819534	0.097342	90.828308
93.840004	82.833298	93.839539	0.089454	90.845123
93.860001	85.666695	93.859535	0.092629	90.862061
93.880005	78.500000	93.879539	0.107155	90.879150
93.900002	80.833298	93.899536	0.135555	90.896362
93.919998	81.166695	93.919533	0.183490	90.913727
93.940002	81.166695	93.939537	0.261903	90.931244
93.959999	77.666695	93.959534	0.391228	90.948883
93.980003	85.666695	93.979538	0.609700	90.966705
94.000000	91.000000	93.999535	0.986179	90.984619
94.019997	87.000000	94.019531	1.638380	91.002686
94.040001	85.000000	94.039536	2.745551	91.020874
94.059998	84.666695	94.059532	4.522184	91.039246
94.080002	98.333298	94.079536	7.099251	91.057739
94.099998	103.000000	94.099533	10.256778	91.076355
94.119995	106.667000	94.119530	13.177114	91.095123
94.139999	103.833000	94.139534	14.648491	91.114044
94.159996	94.166695	94.159531	13.948036	91.133057
94.180000	98.500000	94.179535	11.540281	91.152252
94.199997	94.166695	94.199532	8.599923	91.171570
94.219994	94.500000	94.219528	6.021242	91.191010
94.239998	92.166695	94.239532	4.089723	91.210602
94.259995	90.166695	94.259529	2.738761	91.230316
94.279999	80.333298	94.279533	1.816283	91.250214
94.300003	89.000000	94.299538	1.194405	91.270233
94.320000	84.833298	94.319534	0.780518	91.290344
94.340004	86.166695	94.339546	0.508553	91.310638
94.360001	83.000000	94.359543	0.332336	91.331024
94.380005	84.000000	94.379547	0.218981	91.351593
94.400002	81.166695	94.399544	0.146333	91.372253
94.419998	87.333298	94.419540	0.099618	91.393066
94.440002	80.000000	94.439545	0.069347	91.414001
94.459999	82.333298	94.459541	0.049576	91.435089
94.480003	83.500000	94.479546	0.036602	91.456299

94.500000	78.000000	94.499542	0.028219	91.477631
94.519997	86.833298	94.519539	0.023177	91.499084
94.540001	78.000000	94.539543	0.020942	91.520721
94.559998	86.500000	94.559540	0.021636	91.542450
94.580002	88.666695	94.579544	0.026103	91.564301
94.599998	84.166695	94.599541	0.036021	91.586304
94.619995	80.333298	94.619537	0.053748	91.608429
94.639999	82.333298	94.639542	0.081257	91.630676
94.659996	85.833298	94.659538	0.117416	91.653076
94.680000	83.500000	94.679543	0.154647	91.675598
94.699997	85.333298	94.699539	0.179086	91.698212
94.719994	77.666695	94.719536	0.179185	91.720978
94.739998	85.500000	94.739540	0.156167	91.743866
94.759995	81.500000	94.759537	0.122827	91.766907
94.779999	85.000000	94.779541	0.091904	91.790070
94.800003	84.333298	94.799545	0.069237	91.813324
94.820000	85.166695	94.819542	0.055371	91.836731
94.840004	83.166695	94.839546	0.049294	91.860291
94.860001	92.166695	94.859543	0.050493	91.883911
94.880005	84.000000	94.879547	0.059699	91.907684
94.900002	88.666695	94.899544	0.079554	91.931580
94.919998	85.666695	94.919540	0.115882	91.955597
94.940002	87.166695	94.939545	0.179978	91.979767
94.959999	90.500000	94.959541	0.291884	92.003998
94.980003	88.166695	94.979546	0.483999	92.028381
95.000000	82.333298	94.999542	0.799685	92.052917
95.019997	88.166695	95.019539	1.278065	92.077545
95.040001	89.166695	95.039543	1.911288	92.102264
95.059998	80.500000	95.059540	2.582893	92.127136
95.080002	92.166695	95.079544	3.059951	92.152130
95.099998	83.666695	95.099541	3.121208	92.177216
95.119995	93.166695	95.119537	2.751938	92.202454
95.139999	93.166695	95.139542	2.156629	92.227814
95.159996	86.333298	95.159538	1.568549	92.253265
95.180000	89.500000	95.179543	1.104134	92.278839
95.199997	82.333298	95.199539	0.776703	92.304504
95.219994	86.333298	95.219536	0.560043	92.330292
95.239998	85.000000	95.239540	0.426762	92.356232

95.259995	81.166695	95.259544	0.357967	92.382233
95.279999	81.666695	95.279549	0.343061	92.408386
95.300003	80.833298	95.299553	0.379251	92.434662
95.320000	81.166695	95.319550	0.472683	92.461029
95.340004	85.833298	95.339554	0.642550	92.487518
95.360001	81.500000	95.359550	0.929365	92.514069
95.380005	87.666695	95.379555	1.412145	92.540802
95.400002	86.500000	95.399551	2.236101	92.567596
95.419998	87.833298	95.419548	3.655663	92.594482
95.440002	105.667000	95.439552	6.076472	92.621552
95.459999	103.500000	95.459549	10.037714	92.648651
95.480003	111.000000	95.479553	16.025501	92.675873
95.500000	113.500000	95.499550	23.934130	92.703217
95.519997	122.333000	95.519547	32.342876	92.730682
95.540001	126.167000	95.539551	38.402885	92.758240
95.559998	139.167007	95.559547	39.422268	92.785889
95.580002	126.333000	95.579552	35.258801	92.813660
95.599998	121.333000	95.599548	28.535257	92.841522
95.619995	119.833000	95.619545	22.327747	92.869476
95.639999	116.833000	95.639549	18.382170	92.897522
95.659996	111.000000	95.659546	17.154564	92.925690
95.680000	116.333000	95.679550	18.342144	92.954010
95.699997	124.500000	95.699547	20.950617	92.982330
95.719994	129.500000	95.719543	23.234951	93.010773
95.739998	120.167000	95.739548	23.368006	93.039368
95.759995	116.500000	95.759544	20.750809	93.068024
95.779999	114.500000	95.779549	16.433958	93.096771
95.800003	108.667000	95.799553	11.998191	93.125641
95.820000	102.333000	95.819550	8.379029	93.154572
95.840004	99.666695	95.839554	5.742604	93.183624
95.860001	106.833000	95.859550	3.914796	93.212738
95.880005	95.333298	95.879555	2.675375	93.241974
95.900002	95.000000	95.899551	1.855930	93.271271
95.919998	94.666695	95.919548	1.337768	93.300690
95.940002	99.333298	95.939552	1.041135	93.330200
95.959999	90.500000	95.959549	0.917214	93.359772
95.980003	93.500000	95.979553	0.945687	93.389496
96.000000	92.166695	95.999550	1.139354	93.419220

96.019997	91.833298	96.019547	1.556836	93.449066
96.040001	91.166695	96.039551	2.328617	93.479034
96.059998	96.333298	96.059547	3.696573	93.509064
96.080002	102.500000	96.079552	6.062392	93.539154
96.099998	111.667000	96.099548	9.985483	93.569336
96.119995	115.667000	96.119545	16.029825	93.599640
96.139999	126.167000	96.139549	24.276716	93.629974
96.159996	143.000000	96.159554	33.525188	93.660431
96.180000	143.000000	96.179558	40.924316	93.690979
96.199997	141.500000	96.199554	43.248398	93.721558
96.219994	140.333008	96.219551	39.504097	93.752228
96.239998	133.500000	96.239555	31.875086	93.782990
96.259995	128.833008	96.259552	23.640125	93.813812
96.279999	115.167000	96.279556	16.818417	93.844727
96.300003	108.833000	96.299561	11.887716	93.875763
96.320000	102.000000	96.319557	8.597242	93.906769
96.340004	101.500000	96.339561	6.601754	93.937958
96.360001	105.167000	96.359558	5.693614	93.969147
96.380005	107.833000	96.379562	5.847337	94.000427
96.400002	110.833000	96.399559	7.257571	94.031769
96.419998	120.667000	96.419556	10.384159	94.063202
96.440002	139.833008	96.439560	15.947947	94.094696
96.459999	159.833008	96.459557	24.706480	94.126251
96.480003	161.000000	96.479561	36.860832	94.157898
96.500000	187.333008	96.499557	51.148777	94.189606
96.519997	206.833008	96.519554	64.735657	94.221344
96.540001	223.833008	96.539558	75.146446	94.253204
96.559998	242.333008	96.559555	83.178734	94.285095
96.580002	239.167007	96.579559	92.688293	94.317047
96.599998	263.166992	96.599556	105.997612	94.349060
96.619995	267.332977	96.619553	119.974014	94.381134
96.639999	272.666992	96.639557	127.737579	94.413300
96.659996	266.832977	96.659554	125.388893	94.445465
96.680000	276.166992	96.679558	116.398598	94.477753
96.699997	270.666992	96.699554	107.608650	94.510071
96.719994	260.666992	96.719551	101.872803	94.542419
96.739998	250.333008	96.739555	96.111336	94.574860
96.759995	235.500000	96.759552	85.811218	94.607361

96.779999	217.667007	96.779556	70.280304	94.639923
96.800003	198.500000	96.799561	52.970554	94.672516
96.820000	175.667007	96.819557	37.672955	94.705139
96.840004	162.500000	96.839561	25.969988	94.737854
96.860001	141.000000	96.859558	17.658516	94.770599
96.880005	128.167007	96.879562	11.925398	94.803406
96.900002	116.833000	96.899559	8.026456	94.836273
96.919998	119.500000	96.919556	5.407859	94.869141
96.940002	113.667000	96.939560	3.681710	94.902069
96.959999	115.167000	96.959557	2.578911	94.935059
96.980003	108.833000	96.979561	1.915214	94.968109
97.000000	106.667000	96.999557	1.577793	95.001190
97.019997	102.000000	97.019554	1.514155	95.034302
97.040001	97.833298	97.039558	1.731309	95.067474
97.059998	99.666695	97.059563	2.297908	95.100677
97.080002	98.833298	97.079567	3.345336	95.133911
97.099998	115.500000	97.099564	5.056772	95.167206
97.119995	106.500000	97.119560	7.643509	95.200500
97.139999	113.000000	97.139565	11.308248	95.233856
97.159996	119.667000	97.159561	16.140141	95.267273
97.180000	120.000000	97.179565	21.810814	95.300690
97.199997	118.500000	97.199562	27.088785	95.334137
97.219994	132.833008	97.219559	29.965790	95.367645
97.239998	126.833000	97.239563	28.981184	95.401154
97.259995	125.500000	97.259560	24.624283	95.434723
97.279999	107.333000	97.279564	18.891754	95.468292
97.300003	115.000000	97.299568	13.598036	95.501923
97.320000	112.500000	97.319565	9.494533	95.535583
97.340004	111.833000	97.339569	6.561454	95.569244
97.360001	94.166695	97.359566	4.541921	95.602936
97.380005	96.000000	97.379570	3.189340	95.636658
97.400002	95.333298	97.399567	2.327100	95.670410
97.419998	93.666695	97.419563	1.835445	95.704163
97.440002	86.833298	97.439568	1.643239	95.737946
97.459999	107.000000	97.459564	1.727234	95.771759
97.480003	97.166695	97.479568	2.123323	95.805573
97.500000	108.500000	97.499565	2.953579	95.839417
97.519997	107.500000	97.519562	4.476424	95.873291

97.540001	111.167000	97.539566	7.159055	95.907166
97.559998	121.167000	97.559563	11.737964	95.941071
97.580002	141.667007	97.579567	19.183989	95.974945
97.599998	142.000000	97.599564	30.313984	96.008881
97.619995	158.333008	97.619560	44.867855	96.042816
97.639999	163.000000	97.639565	60.241356	96.076782
97.659996	163.833008	97.659561	71.315674	96.110718
97.680000	169.167007	97.679565	73.205086	96.144714
97.699997	166.833008	97.699562	65.306541	96.178650
97.719994	159.000000	97.719559	51.854832	96.212646
97.739998	139.500000	97.739563	38.040474	96.246643
97.759995	145.500000	97.759560	26.733814	96.280609
97.779999	121.500000	97.779564	18.411060	96.314636
97.800003	115.333000	97.799568	12.542789	96.348663
97.820000	111.333000	97.819565	8.475199	96.382629
97.840004	104.833000	97.839569	5.691197	96.416626
97.860001	106.000000	97.859566	3.820989	96.450623
97.880005	103.167000	97.879570	2.593244	96.484619
97.900002	102.000000	97.899567	1.814217	96.518616
97.919998	102.500000	97.919563	1.347768	96.552612
97.940002	98.166695	97.939568	1.106444	96.586609
97.959999	101.167000	97.959572	1.044049	96.620575
97.980003	95.500000	97.979576	1.153372	96.654572
98.000000	97.000000	97.999573	1.472726	96.688538
98.019997	102.833000	98.019569	2.104525	96.722504
98.040001	103.833000	98.039574	3.247557	96.756439
98.059998	97.666695	98.059570	5.232372	96.790375
98.080002	106.000000	98.079575	8.531557	96.824310
98.099998	122.667000	98.099571	13.637354	96.858215
98.119995	129.500000	98.119568	20.697308	96.892090
98.139999	136.500000	98.139572	28.874041	96.925995
98.159996	139.833008	98.159569	35.917286	96.959839
98.180000	149.833008	98.179573	38.983723	96.993683
98.199997	145.500000	98.199570	36.711063	97.027527
98.219994	129.833008	98.219566	30.475822	97.061310
98.239998	122.667000	98.239571	23.067785	97.095123
98.259995	130.000000	98.259567	16.560303	97.128876
98.279999	114.333000	98.279572	11.628681	97.162628

98.300003	103.833000	98.299576	8.142927	97.196350
98.320000	108.000000	98.319572	5.771217	97.230042
98.340004	102.000000	98.339577	4.230520	97.263733
98.360001	95.166695	98.359573	3.332433	97.297363
98.380005	97.166695	98.379578	2.969970	97.330994
98.400002	106.500000	98.399574	3.123316	97.364532
98.419998	105.333000	98.419571	3.884718	97.398102
98.440002	112.333000	98.439575	5.514723	97.431641
98.459999	124.500000	98.459572	8.521967	97.465088
98.480003	128.833008	98.479576	13.752340	97.498566
98.500000	144.167007	98.499573	22.356571	97.531982
98.519997	169.000000	98.519569	35.435131	97.565369
98.540001	183.167007	98.539574	53.021358	97.598724
98.559998	205.333008	98.559570	72.511696	97.632019
98.580002	219.167007	98.579575	88.086746	97.665283
98.599998	218.833008	98.599571	93.228661	97.698517
98.619995	202.167007	98.619568	85.806107	97.731689
98.639999	203.000000	98.639572	70.008530	97.764862
98.659996	180.167007	98.659569	52.477352	97.797943
98.680000	155.500000	98.679573	37.568398	97.830994
98.699997	147.167007	98.699570	26.507267	97.864014
98.719994	131.500000	98.719566	18.878849	97.896973
98.739998	120.833000	98.739571	13.952744	97.929901
98.759995	120.667000	98.759567	11.111341	97.962738
98.779999	111.167000	98.779572	9.789358	97.995575
98.800003	117.667000	98.799576	9.333631	98.028351
98.820000	111.000000	98.819572	8.995337	98.061066
98.840004	113.333000	98.839577	8.196424	98.093750
98.860001	109.833000	98.859581	6.840164	98.126343
98.880005	107.167000	98.879585	5.253703	98.158936
98.900002	102.833000	98.899582	3.811414	98.191406
98.919998	92.000000	98.919579	2.683812	98.223846
98.940002	102.333000	98.939583	1.865386	98.256256
98.959999	102.167000	98.959579	1.288304	98.288544
98.980003	93.000000	98.979584	0.885243	98.320831
99.000000	81.833298	98.999580	0.606423	98.353027
99.019997	85.166695	99.019577	0.415703	98.385132
99.040001	91.833298	99.039581	0.286888	98.417236

99.059998	90.833298	99.059578	0.200991	98.449219
99.080002	92.833298	99.079582	0.144261	98.481171
99.099998	88.666695	99.099579	0.107214	98.513062
99.119995	88.166695	99.119576	0.083409	98.544861
99.139999	88.000000	99.139580	0.068662	98.576630
99.159996	87.666695	99.159576	0.060367	98.608276
99.180000	91.500000	99.179581	0.056981	98.639862
99.199997	87.166695	99.199577	0.057729	98.671417
99.219994	97.500000	99.219574	0.062399	98.702850
99.239998	95.666695	99.239578	0.071272	98.734253
99.259995	96.000000	99.259575	0.085099	98.765533
99.279999	87.500000	99.279579	0.105208	98.796783
99.300003	87.500000	99.299583	0.133642	98.827972
99.320000	90.833298	99.319580	0.173418	98.859039
99.340004	95.333298	99.339584	0.229010	98.890045
99.360001	94.500000	99.359581	0.306871	98.920929
99.380005	95.833298	99.379585	0.416663	98.951782
99.400002	89.166695	99.399582	0.572810	98.982544
99.419998	87.333298	99.419579	0.798044	99.013214
99.440002	87.333298	99.439583	1.129679	99.043793
99.459999	97.000000	99.459579	1.631181	99.074310
99.480003	92.000000	99.479584	2.416048	99.104736
99.500000	96.000000	99.499580	3.687041	99.135040
99.519997	100.667000	99.519577	5.805214	99.165314
99.540001	118.167000	99.539581	9.384561	99.195465
99.559998	135.667007	99.559578	15.375594	99.225494
99.580002	149.167007	99.579582	25.051113	99.255493
99.599998	171.333008	99.599579	39.602341	99.285400
99.619995	199.833008	99.619576	59.120411	99.315155
99.639999	222.500000	99.639580	80.996353	99.344879
99.659996	218.000000	99.659576	99.103378	99.374481
99.680000	233.000000	99.679581	106.229095	99.404022
99.699997	245.500000	99.699577	99.325127	99.433380
99.719994	212.333008	99.719574	82.289452	99.462708
99.739998	193.000000	99.739586	62.356125	99.491913
99.759995	173.000000	99.759583	44.823502	99.521057
99.779999	142.000000	99.779587	31.385298	99.550079
99.800003	134.833008	99.799591	21.684736	99.579010

99.820000	118.000000	99.819588	14.833768	99.607819
99.840004	111.667000	99.839592	10.047318	99.636505
99.860001	103.500000	99.859589	6.750808	99.665131
99.880005	107.667000	99.879593	4.516388	99.693634
99.900002	99.166695	99.899590	3.029475	99.722046
99.919998	111.500000	99.919586	2.056137	99.750336
99.940002	110.333000	99.939590	1.429594	99.778503
99.959999	104.667000	99.959587	1.035676	99.806580
99.980003	98.333298	99.979591	0.798580	99.834564
100.000000	100.500000	99.999588	0.671562	99.862396
100.019997	92.666695	100.019585	0.628136	99.890167
100.040001	94.833298	100.039589	0.657321	99.917816
100.059998	94.333298	100.059586	0.761130	99.945343
100.080002	90.166695	100.079590	0.955664	99.972748
100.099998	91.000000	100.099586	1.276239	100.000031
100.119995	97.500000	100.119583	1.790688	100.027222
100.139999	102.500000	100.139587	2.624909	100.054291
100.159996	102.833000	100.159584	4.006713	100.081207
100.180000	105.000000	100.179588	6.339750	100.108063
100.199997	116.667000	100.199585	10.288202	100.134766
100.219994	133.167007	100.219582	16.838869	100.161346
100.239998	148.333008	100.239586	27.179905	100.187836
100.259995	177.167007	100.259583	42.113445	100.214203
100.279999	196.833008	100.279587	60.869217	100.240417
100.300003	213.000000	100.299591	79.715736	100.266571
100.320000	226.333008	100.319588	92.308456	100.292542
100.340004	222.167007	100.339592	93.289314	100.318390
100.360001	217.000000	100.359589	82.672424	100.344147
100.380005	198.000000	100.379593	65.790955	100.369781
100.400002	171.000000	100.399590	48.717995	100.395233
100.419998	160.833008	100.419586	34.698723	100.420593
100.440002	143.667007	100.439590	24.268219	100.445831
100.459999	123.000000	100.459587	16.813761	100.470947
100.480003	111.167000	100.479591	11.569210	100.495941
100.500000	113.333000	100.499588	7.939943	100.520782
100.519997	104.000000	100.519585	5.488225	100.545502
100.540001	110.500000	100.539589	3.897121	100.570068
100.559998	106.000000	100.559586	2.943924	100.594513

100.580002	111.000000	100.579590	2.487163	100.618866
100.599998	108.833000	100.599586	2.471477	100.643066
100.619995	112.167000	100.619591	2.938886	100.667114
100.639999	116.167000	100.639595	4.053975	100.691040
100.659996	117.833000	100.659592	6.130097	100.714813
100.680000	124.167000	100.679596	9.625837	100.738464
100.699997	131.333008	100.699593	15.010425	100.761993
100.719994	137.000000	100.719589	22.410091	100.785339
100.739998	141.500000	100.739594	31.032181	100.808594
100.759995	146.667007	100.759590	38.834045	100.831665
100.779999	147.000000	100.779594	43.329964	100.854645
100.800003	156.000000	100.799599	43.436798	100.877472
100.820000	153.667007	100.819595	40.515190	100.900146
100.840004	157.833008	100.839600	36.984657	100.922699
100.860001	151.167007	100.859596	34.098854	100.945099
100.880005	134.167007	100.879601	31.324152	100.967377
100.900002	132.167007	100.899597	27.572334	100.989471
100.919998	130.333008	100.919594	22.622484	101.011444
100.940002	125.333000	100.939598	17.293461	101.033264
100.959999	111.333000	100.959595	12.563647	101.054962
100.980003	111.500000	100.979599	8.884603	101.076508
101.000000	111.167000	100.999596	6.229238	101.097870
101.019997	108.333000	101.019592	4.381695	101.119110
101.040001	91.166695	101.039597	3.132331	101.140228
101.059998	94.166695	101.059593	2.329953	101.161163
101.080002	98.666695	101.079597	1.877611	101.181976
101.099998	95.666695	101.099594	1.730212	101.202637
101.119995	98.500000	101.119591	1.898248	101.223145
101.139999	98.666695	101.139595	2.465846	101.243500
101.159996	104.500000	101.159592	3.614406	101.263733
101.180000	105.500000	101.179596	5.641609	101.283783
101.199997	117.667000	101.199593	8.921668	101.303711
101.219994	116.000000	101.219589	13.738575	101.323456
101.239998	120.000000	101.239594	19.908318	101.343048
101.259995	126.333000	101.259590	26.314880	101.362518
101.279999	123.333000	101.279594	30.933910	101.381866
101.300003	128.333008	101.299599	31.855257	101.401031
101.320000	121.833000	101.319595	28.790167	101.420013

101.340004	120.500000	101.339600	23.311121	101.438873
101.360001	112.167000	101.359596	17.487307	101.457550
101.380005	110.833000	101.379601	12.564043	101.476105
101.400002	103.500000	101.399597	8.841547	101.494476
101.419998	97.000000	101.419594	6.148861	101.512726
101.440002	98.666695	101.439598	4.231252	101.530792
101.459999	87.500000	101.459595	2.881193	101.548706
101.480003	96.166695	101.479599	1.943049	101.566498
101.500000	95.500000	101.499603	1.302016	101.584106
101.519997	96.000000	101.519600	0.870974	101.601562
101.540001	93.500000	101.539604	0.584475	101.618866
101.559998	94.333298	101.559601	0.395569	101.636017
101.580002	96.000000	101.579605	0.271080	101.652985
101.599998	90.833298	101.599602	0.188724	101.669830
101.619995	90.333298	101.619598	0.133670	101.686493
101.639999	95.833298	101.639603	0.096340	101.703003
101.659996	96.000000	101.659599	0.070629	101.719391
101.680000	89.833298	101.679604	0.052599	101.735565
101.699997	93.166695	101.699600	0.039751	101.751617
101.719994	91.333298	101.719597	0.030447	101.767487
101.739998	96.500000	101.739601	0.023615	101.783203
101.759995	92.000000	101.759598	0.018539	101.798798
101.779999	90.000000	101.779602	0.014726	101.814178
101.800003	88.666695	101.799606	0.011837	101.829437
101.820000	84.666695	101.819603	0.009633	101.844543
101.840004	88.833298	101.839607	0.007942	101.859467
101.860001	89.000000	101.859604	0.006641	101.874207
101.880005	86.166695	101.879608	0.005640	101.888824
101.900002	83.166695	101.899605	0.004873	101.903259
101.919998	82.166695	101.919601	0.004293	101.917572
101.940002	85.166695	101.939606	0.003863	101.931671
101.959999	94.000000	101.959602	0.003558	101.945648
101.980003	88.500000	101.979607	0.003359	101.959473
102.000000	84.166695	101.999603	0.003255	101.973114
102.019997	93.833298	102.019600	0.003238	101.986603
102.040001	88.666695	102.039604	0.003304	101.999939
102.059998	85.333298	102.059601	0.003454	102.013092
102.080002	91.333298	102.079605	0.003691	102.026093

102.099998	86.000000	102.099602	0.004025	102.038910
102.119995	89.500000	102.119598	0.004467	102.051636
102.139999	85.000000	102.139603	0.005036	102.064148
102.159996	88.833298	102.159599	0.005756	102.076508
102.180000	83.833298	102.179604	0.006659	102.088715
102.199997	84.666695	102.199600	0.007788	102.100739
102.219994	87.166695	102.219597	0.009200	102.112640
102.239998	89.833298	102.239601	0.010971	102.124359
102.259995	94.000000	102.259598	0.013200	102.135925
102.279999	85.500000	102.279602	0.016024	102.147339
102.300003	90.000000	102.299606	0.019623	102.158600
102.320000	90.166695	102.319603	0.024245	102.169678
102.340004	85.000000	102.339607	0.030232	102.180634
102.360001	82.833298	102.359612	0.038051	102.191376
102.380005	89.500000	102.379616	0.048354	102.201996
102.400002	87.333298	102.399612	0.062040	102.212433
102.419998	87.333298	102.419609	0.080385	102.222687
102.440002	91.000000	102.439613	0.105199	102.232849
102.459999	93.000000	102.459610	0.139059	102.242798
102.480003	89.833298	102.479614	0.185829	102.252625
102.500000	91.166695	102.499611	0.251371	102.262299
102.519997	87.833298	102.519608	0.345181	102.271759
102.540001	86.166695	102.539612	0.483285	102.281128
102.559998	101.000000	102.559608	0.693366	102.290314
102.580002	94.000000	102.579613	1.024560	102.299347
102.599998	94.833298	102.599609	1.561637	102.308228
102.619995	96.333298	102.619606	2.445838	102.316925
102.639999	101.167000	102.639610	3.892928	102.325500
102.659996	100.167000	102.659607	6.182150	102.333893
102.680000	114.000000	102.679611	9.575686	102.342133
102.699997	113.000000	102.699608	14.092725	102.350250
102.719994	125.000000	102.719604	19.201059	102.358185
102.739998	110.500000	102.739609	23.683996	102.365967
102.759995	115.500000	102.759605	26.139614	102.373596
102.779999	120.333000	102.779610	26.066204	102.381073
102.800003	129.667007	102.799614	24.488173	102.388397
102.820000	134.333008	102.819611	23.414154	102.395569
102.840004	141.667007	102.839615	24.888470	102.402618

102.860001	156.667007	102.859612	30.696033	102.409454
102.880005	176.333008	102.879616	42.539677	102.416199
102.900002	209.333008	102.899612	61.760719	102.422760
102.919998	244.833008	102.919609	88.075691	102.429169
102.940002	259.166992	102.939613	117.568329	102.435394
102.959999	290.166992	102.959610	141.877609	102.441559
102.980003	278.332977	102.979614	151.538513	102.447510
103.000000	273.166992	102.999611	142.533752	102.453339
103.019997	258.500000	103.019608	119.685715	102.459015
103.040001	250.667007	103.039612	92.330261	102.464539
103.059998	228.000000	103.059608	67.663269	102.469910
103.080002	185.667007	103.079613	48.314270	102.475159
103.099998	160.667007	103.099609	34.047112	102.480225
103.119995	148.500000	103.119606	23.745117	102.485199
103.139999	122.500000	103.139610	16.378618	102.489960
103.159996	116.333000	103.159607	11.178664	102.494629
103.180000	111.333000	103.179611	7.563268	102.499176
103.199997	119.333000	103.199608	5.093709	102.503510
103.219994	116.000000	103.219612	3.430465	102.507751
103.239998	114.500000	103.239616	2.322674	102.511871
103.259995	118.667000	103.259613	1.588960	102.515808
103.279999	121.000000	103.279617	1.102420	102.519623
103.300003	105.333000	103.299622	0.778076	102.523285
103.320000	101.333000	103.319618	0.559758	102.526825
103.340004	100.500000	103.339622	0.410846	102.530212
103.360001	104.167000	103.359619	0.308043	102.533478
103.380005	97.666695	103.379623	0.236257	102.536591
103.400002	95.166695	103.399620	0.185892	102.539581
103.419998	88.000000	103.419617	0.150683	102.542450
103.440002	88.000000	103.439621	0.126584	102.545197
103.459999	94.666695	103.459618	0.111027	102.547760
103.480003	97.166695	103.479622	0.102413	102.550232
103.500000	92.166695	103.499619	0.099901	102.552582
103.519997	87.333298	103.519615	0.103244	102.554749
103.540001	85.000000	103.539619	0.112759	102.556824
103.559998	100.500000	103.559616	0.129341	102.558746
103.580002	102.167000	103.579620	0.154599	102.560577
103.599998	83.833298	103.599617	0.191010	102.562256

103.619995	92.166695	103.619614	0.242312	102.563843
103.639999	90.666695	103.639618	0.314073	102.565277
103.659996	91.666695	103.659615	0.414540	102.566589
103.680000	96.333298	103.679619	0.556554	102.567780
103.699997	96.666695	103.699615	0.760239	102.568878
103.719994	98.833298	103.719612	1.058715	102.569794
103.739998	96.666695	103.739616	1.507422	102.570648
103.759995	102.333000	103.759613	2.198625	102.571350
103.779999	108.500000	103.779617	3.285084	102.571960
103.800003	104.333000	103.799622	5.004641	102.572449
103.820000	120.167000	103.819618	7.696250	102.572784
103.840004	123.000000	103.839622	11.780451	102.573059
103.860001	137.000000	103.859619	17.653290	102.573212
103.880005	148.333008	103.879623	25.557343	102.573212
103.900002	166.500000	103.899620	35.566093	102.573151
103.919998	186.000000	103.919617	48.000156	102.572968
103.940002	217.833008	103.939621	63.970661	102.572662
103.959999	252.667007	103.959618	84.956734	102.572235
103.980003	277.000000	103.979622	110.701447	102.571716
104.000000	295.332977	103.999619	136.580734	102.571136
104.019997	302.666992	104.019615	153.927948	102.570404
104.040001	294.500000	104.039619	154.964600	102.569550
104.059998	292.332977	104.059616	138.991867	102.568634
104.080002	271.666992	104.079628	112.925026	102.567596
104.099998	248.000000	104.099625	85.597984	102.566467
104.119995	217.000000	104.119621	62.359821	102.565216
104.139999	190.333008	104.139626	44.549774	102.563934
104.159996	163.500000	104.159622	31.479153	102.562500
104.180000	142.333008	104.179626	22.016735	102.561005
104.199997	130.333008	104.199623	15.233467	102.559387
104.219994	130.167007	104.219620	10.430695	102.557709
104.239998	121.500000	104.239624	7.085027	102.555908
104.259995	122.333000	104.259621	4.795615	102.554047
104.279999	125.167000	104.279625	3.250312	102.552094
104.300003	124.000000	104.299629	2.218128	102.550018
104.320000	120.500000	104.319626	1.532218	102.547882
104.340004	111.500000	104.339630	1.076015	102.545685
104.360001	111.167000	104.359627	0.771633	102.543365

104.380005	110.500000	104.379631	0.567261	102.540985
104.400002	107.833000	104.399628	0.429661	102.538544
104.419998	105.500000	104.419624	0.337402	102.536011
104.440002	101.833000	104.439629	0.277003	102.533417
104.459999	98.666695	104.459625	0.240223	102.530701
104.480003	86.333298	104.479630	0.222228	102.527954
104.500000	98.333298	104.499626	0.220732	102.525116
104.519997	89.166695	104.519623	0.235416	102.522217
104.540001	110.667000	104.539627	0.267816	102.519257
104.559998	95.333298	104.559624	0.321384	102.516205
104.580002	100.667000	104.579628	0.402021	102.513092
104.599998	98.666695	104.599625	0.518738	102.509918
104.619995	96.333298	104.619621	0.685374	102.506683
104.639999	98.333298	104.639626	0.923450	102.503418
104.659996	97.666695	104.659622	1.267236	102.500061
104.680000	95.500000	104.679626	1.774927	102.496613
104.699997	93.666695	104.699623	2.547007	102.493164
104.719994	98.500000	104.719620	3.762470	102.489655
104.739998	108.333000	104.739624	5.737404	102.486084
104.759995	122.000000	104.759621	9.007712	102.482452
104.779999	130.500000	104.779625	14.434174	102.478760
104.800003	154.833008	104.799629	23.238018	102.475037
104.820000	170.500000	104.819626	36.816685	102.471283
104.840004	193.000000	104.839630	56.082211	102.467468
104.860001	230.500000	104.859627	80.071480	102.463593
104.880005	245.833008	104.879631	104.552025	102.459686
104.900002	265.500000	104.899628	122.206268	102.455750
104.919998	252.333008	104.919624	126.298386	102.451782
104.940002	252.000000	104.939636	115.587120	102.447754
104.959999	236.500000	104.959633	95.385277	102.443695
104.980003	217.667007	104.979637	73.111305	102.439606
105.000000	188.833008	104.999634	53.698452	102.435516
105.019997	159.500000	105.019630	38.619614	102.431335
105.040001	148.333008	105.039635	27.449633	102.427185
105.059998	139.833008	105.059631	19.311666	102.422974
105.080002	116.500000	105.079636	13.431386	102.418732
105.099998	114.500000	105.099632	9.239013	102.414520
105.119995	112.833000	105.119629	6.297921	102.410248

105.139999	112.000000	105.139633	4.269704	102.405945
105.159996	112.333000	105.159630	2.893316	102.401642
105.180000	120.000000	105.179634	1.968884	102.397339
105.199997	114.000000	105.199631	1.352092	102.392975
105.219994	119.167000	105.219627	0.940444	102.388641
105.239998	113.833000	105.239632	0.664194	102.384277
105.259995	105.333000	105.259628	0.477142	102.379944
105.279999	103.000000	105.279633	0.348758	102.375549
105.300003	101.833000	105.299637	0.259426	102.371185
105.320000	91.500000	105.319633	0.196445	102.366821
105.340004	95.500000	105.339638	0.151538	102.362457
105.360001	99.666695	105.359634	0.119413	102.358063
105.380005	91.166695	105.379639	0.096618	102.353699
105.400002	92.000000	105.399635	0.081067	102.349335
105.419998	96.333298	105.419632	0.071655	102.344971
105.440002	88.166695	105.439636	0.068146	102.340637
105.459999	95.000000	105.459633	0.071086	102.336304
105.480003	92.166695	105.479637	0.081561	102.332001
105.500000	89.833298	105.499634	0.100519	102.327698
105.519997	88.500000	105.519630	0.127432	102.323364
105.540001	90.000000	105.539635	0.158630	102.319122
105.559998	87.333298	105.559631	0.186958	102.314850
105.580002	88.000000	105.579636	0.205026	102.310638
105.599998	92.166695	105.599632	0.210726	102.306458
105.619995	91.333298	105.619629	0.209885	102.302277
105.639999	95.500000	105.639633	0.212696	102.298126
105.659996	87.000000	105.659630	0.228420	102.294006
105.680000	93.166695	105.679634	0.263813	102.289948
105.699997	98.500000	105.699631	0.325184	102.285919
105.719994	89.666695	105.719627	0.421736	102.281891
105.739998	98.833298	105.739632	0.569290	102.277954
105.759995	93.666695	105.759628	0.795493	102.274017
105.779999	85.000000	105.779640	1.149826	102.270142
105.800003	96.833298	105.799644	1.718144	102.266327
105.820000	106.167000	105.819641	2.646632	102.262543
105.840004	99.833298	105.839645	4.170831	102.258789
105.860001	109.500000	105.859642	6.630001	102.255096
105.880005	117.500000	105.879646	10.439948	102.251495

105.900002	130.833008	105.899643	15.937563	102.247894
105.919998	129.500000	105.919640	23.083218	102.244415
105.940002	138.167007	105.939644	31.101416	102.240936
105.959999	153.333008	105.959641	38.431503	102.237549
105.980003	155.833008	105.979645	43.378441	102.234253
106.000000	157.167007	105.999641	45.018353	102.230957
106.019997	156.167007	106.019638	43.459595	102.227783
106.040001	147.667007	106.039642	39.303314	102.224670
106.059998	144.000000	106.059639	33.328384	102.221649
106.080002	130.833008	106.079643	26.567093	102.218658
106.099998	130.000000	106.099640	20.132526	102.215759
106.119995	120.667000	106.119637	14.745842	102.212982
106.139999	112.833000	106.139641	10.590569	102.210236
106.159996	110.833000	106.159637	7.520871	102.207611
106.180000	107.667000	106.179642	5.294976	102.205017
106.199997	104.500000	106.199638	3.702189	102.202545
106.219994	101.333000	106.219635	2.578163	102.200195
106.239998	105.500000	106.239639	1.799251	102.197906
106.259995	100.000000	106.259636	1.272192	102.195709
106.279999	98.166695	106.279640	0.925920	102.193634
106.300003	97.166695	106.299644	0.709320	102.191650
106.320000	95.666695	106.319641	0.587350	102.189789
106.340004	101.167000	106.339645	0.538382	102.187958
106.360001	97.333298	106.359642	0.552251	102.186310
106.380005	98.666695	106.379646	0.629248	102.184723
106.400002	94.166695	106.399643	0.781656	102.183319
106.419998	100.500000	106.419640	1.039176	102.181976
106.440002	98.500000	106.439644	1.460046	102.180695
106.459999	105.333000	106.459641	2.149865	102.179596
106.480003	101.333000	106.479645	3.293157	102.178619
106.500000	106.333000	106.499641	5.188806	102.177765
106.519997	109.833000	106.519638	8.280060	102.177002
106.540001	114.500000	106.539642	13.122091	102.176392
106.559998	130.000000	106.559639	20.187246	102.175903
106.580002	137.667007	106.579643	29.446079	102.175537
106.599998	143.833008	106.599640	39.747318	102.175323
106.619995	152.167007	106.619644	48.587029	102.175201
106.639999	156.167007	106.639648	52.936180	102.175262

106.659996	155.333008	106.659645	51.168850	102.175446
106.680000	148.833008	106.679649	44.332737	102.175781
106.699997	133.000000	106.699646	35.272545	102.176270
106.719994	124.667000	106.719643	26.568365	102.176880
106.739998	132.667007	106.739647	19.442387	102.177643
106.759995	121.500000	106.759644	14.029213	102.178558
106.779999	108.833000	106.779648	10.021130	102.179657
106.800003	107.000000	106.799652	7.081684	102.180878
106.820000	98.166695	106.819649	4.947198	102.182281
106.840004	99.833298	106.839653	3.418752	102.183807
106.860001	104.167000	106.859650	2.344589	102.185516
106.880005	103.333000	106.879654	1.602580	102.187408
106.900002	99.333298	106.899651	1.098416	102.189453
106.919998	104.833000	106.919647	0.760023	102.191681
106.940002	106.667000	106.939651	0.535212	102.194031
106.959999	103.000000	106.959648	0.387824	102.196594
106.980003	94.666695	106.979652	0.293575	102.199341
107.000000	107.000000	106.999649	0.237612	102.202209
107.019997	100.167000	107.019646	0.212257	102.205322
107.040001	96.500000	107.039650	0.216334	102.208588
107.059998	99.500000	107.059647	0.255323	102.212067
107.080002	88.333298	107.079651	0.342231	102.215698
107.099998	91.000000	107.099648	0.497493	102.219543
107.119995	101.667000	107.119644	0.745414	102.223541
107.139999	91.500000	107.139648	1.101972	102.227783
107.159996	93.166695	107.159645	1.550768	102.232178
107.180000	101.833000	107.179649	2.018337	102.236816
107.199997	96.500000	107.199646	2.374309	102.241608
107.219994	95.000000	107.219643	2.490504	102.246613
107.239998	96.500000	107.239647	2.328217	102.251831
107.259995	87.833298	107.259644	1.969521	102.257263
107.279999	88.666695	107.279648	1.550405	102.262878
107.300003	97.000000	107.299652	1.172993	102.268707
107.320000	86.833298	107.319649	0.875174	102.274780
107.340004	87.833298	107.339653	0.652308	102.281036
107.360001	93.166695	107.359650	0.487026	102.287506
107.380005	91.333298	107.379654	0.363849	102.294281
107.400002	95.000000	107.399651	0.273511	102.301178

107.419998	85.500000	107.419647	0.211246	102.308319
107.440002	90.333298	107.439651	0.174902	102.315704
107.459999	90.666695	107.459656	0.164426	102.323303
107.480003	96.833298	107.479660	0.182836	102.331146
107.500000	88.833298	107.499657	0.237580	102.339203
107.519997	93.000000	107.519653	0.340717	102.347504
107.540001	88.333298	107.539658	0.505841	102.356049
107.559998	95.000000	107.559654	0.738729	102.364838
107.580002	88.166695	107.579659	1.022305	102.373810
107.599998	95.333298	107.599655	1.302113	102.383087
107.619995	87.666695	107.619652	1.494553	102.392578
107.639999	95.500000	107.639656	1.528526	102.402313
107.659996	89.833298	107.659653	1.396255	102.412323
107.680000	91.833298	107.679657	1.159017	102.422546
107.699997	93.500000	107.699654	0.899284	102.433044
107.719994	91.500000	107.719650	0.671556	102.443787
107.739998	97.500000	107.739655	0.492934	102.454803
107.759995	92.500000	107.759651	0.359425	102.466064
107.779999	87.333298	107.779655	0.261297	102.477570
107.800003	92.166695	107.799660	0.190189	102.489410
107.820000	92.500000	107.819656	0.139893	102.501434
107.840004	89.166695	107.839661	0.105743	102.513763
107.860001	95.833298	107.859657	0.084231	102.526337
107.880005	92.666695	107.879662	0.072694	102.539185
107.900002	87.833298	107.899658	0.069332	102.552307
107.919998	100.000000	107.919655	0.073151	102.565704
107.940002	96.500000	107.939659	0.083974	102.579376
107.959999	90.000000	107.959656	0.102447	102.593292
107.980003	98.000000	107.979660	0.130163	102.607544
108.000000	91.166695	107.999657	0.169858	102.622040
108.019997	92.166695	108.019653	0.225994	102.636871
108.040001	96.000000	108.039658	0.305818	102.651917
108.059998	96.166695	108.059654	0.421285	102.667328
108.080002	93.333298	108.079659	0.593161	102.682953
108.099998	90.333298	108.099655	0.857527	102.698883
108.119995	93.833298	108.119652	1.277730	102.715118
108.139999	100.833000	108.139656	1.961890	102.731628
108.159996	103.833000	108.159653	3.083567	102.748444

108.180000	109.833000	108.179657	4.900409	102.765594
108.199997	112.000000	108.199654	7.734337	102.782990
108.219994	118.500000	108.219650	11.877462	102.800690
108.239998	125.333000	108.239655	17.358587	102.818695
108.259995	134.167007	108.259651	23.596689	102.837036
108.279999	135.667007	108.279655	29.223080	102.855652
108.300003	132.500000	108.299667	32.445923	102.874603
108.320000	130.167007	108.319664	32.089867	102.893829
108.340004	123.333000	108.339668	28.475649	102.913361
108.360001	128.833008	108.359665	23.151052	102.933197
108.380005	126.167000	108.379669	17.747755	102.953400
108.400002	132.333008	108.399666	13.183497	102.973877
108.419998	119.333000	108.419662	9.654257	102.994690
108.440002	107.833000	108.439667	7.017700	103.015778
108.459999	101.833000	108.459663	5.074751	103.037231
108.480003	100.167000	108.479668	3.661085	103.058960
108.500000	100.167000	108.499664	2.658900	103.081024
108.519997	99.166695	108.519661	1.979284	103.103424
108.540001	102.167000	108.539673	1.556762	103.126129
108.559998	96.166695	108.559669	1.349487	103.149170
108.580002	102.333000	108.579674	1.341937	103.172577
108.599998	103.667000	108.599670	1.555074	103.196259
108.619995	107.000000	108.619667	2.059727	103.220276
108.639999	108.500000	108.639671	2.995239	103.244629
108.659996	123.000000	108.659668	4.582731	103.269318
108.680000	119.000000	108.679672	7.117470	103.294373
108.699997	124.333000	108.699669	10.882863	103.319672
108.719994	122.833000	108.719666	15.954165	103.345367
108.739998	136.000000	108.739670	21.884611	103.371429
108.759995	137.833008	108.759666	27.472223	103.397736
108.779999	143.000000	108.779671	31.031261	103.424469
108.800003	139.833008	108.799675	31.281042	103.451508
108.820000	133.667007	108.819672	28.279108	103.478882
108.840004	135.500000	108.839676	23.361835	103.506592
108.860001	126.667000	108.859673	18.147932	103.534637
108.880005	126.333000	108.879677	13.644731	103.563049
108.900002	119.167000	108.899673	10.148434	103.591827
108.919998	117.500000	108.919670	7.566682	103.620911

108.940002	118.167000	108.939674	5.724948	103.650360
108.959999	107.667000	108.959671	4.492369	103.680145
108.980003	107.500000	108.979675	3.801356	103.710297
109.000000	105.667000	108.999672	3.652762	103.740784
109.019997	110.167000	109.019669	4.121068	103.771606
109.040001	112.833000	109.039673	5.366303	103.802765
109.059998	124.000000	109.059669	7.622790	103.834320
109.080002	125.333000	109.079674	11.143682	103.866211
109.099998	137.167007	109.099670	16.053236	103.898438
109.119995	156.333008	109.119667	22.142284	103.931000
109.139999	156.167007	109.139679	28.666885	103.964020
109.159996	156.833008	109.159676	34.297241	103.997284
109.180000	167.833008	109.179680	37.472416	104.030945
109.199997	166.333008	109.199677	37.106937	104.064941
109.219994	157.833008	109.219673	33.345654	104.099274
109.239998	144.000000	109.239677	27.547245	104.134003
109.259995	140.000000	109.259674	21.404709	104.169098
109.279999	133.833008	109.279678	16.031887	104.204529
109.300003	130.333008	109.299683	11.787068	104.240356
109.320000	114.667000	109.319679	8.577847	104.276489
109.340004	111.167000	109.339684	6.184465	104.313019
109.360001	109.000000	109.359680	4.414527	104.349884
109.380005	109.667000	109.379684	3.120017	104.387085
109.400002	107.000000	109.399681	2.190356	104.424683
109.419998	103.333000	109.419678	1.536467	104.462616
109.440002	105.000000	109.439682	1.087302	104.500946
109.459999	104.667000	109.459679	0.787791	104.539581
109.480003	101.167000	109.479683	0.596345	104.578613
109.500000	104.667000	109.499680	0.484187	104.617950
109.519997	108.000000	109.519676	0.433073	104.657684
109.540001	101.167000	109.539680	0.434570	104.697845
109.559998	107.333000	109.559677	0.490212	104.738251
109.580002	106.500000	109.579681	0.613955	104.779083
109.599998	100.500000	109.599678	0.837400	104.820282
109.619995	105.500000	109.619675	1.219185	104.861786
109.639999	106.500000	109.639679	1.857254	104.903717
109.659996	103.167000	109.659676	2.898025	104.945953
109.680000	115.833000	109.679680	4.532939	104.988586

109.699997	111.167000	109.699677	6.946747	105.031555
109.719994	124.833000	109.719673	10.198772	105.074890
109.739998	127.500000	109.739677	14.029501	105.118561
109.759995	121.667000	109.759674	17.706768	105.162598
109.779999	123.833000	109.779678	20.168505	105.207031
109.800003	126.167000	109.799683	20.551203	105.251801
109.820000	122.333000	109.819679	18.797045	105.296906
109.840004	132.000000	109.839684	15.690853	105.342377
109.860001	114.833000	109.859680	12.275240	105.388214
109.880005	108.167000	109.879684	9.249874	105.434418
109.900002	107.667000	109.899681	6.850487	105.480896
109.919998	108.500000	109.919678	5.032509	105.527802
109.940002	115.667000	109.939682	3.675224	105.575043
109.959999	104.833000	109.959686	2.672004	105.622650
109.980003	104.167000	109.979691	1.943412	105.670624
110.000000	101.000000	109.999687	1.430380	105.718903
110.019997	104.000000	110.019684	1.086520	105.767548
110.040001	111.167000	110.039688	0.876085	105.816528
110.059998	106.667000	110.059685	0.773751	105.865845
110.080002	108.500000	110.079689	0.765056	105.915558
110.099998	103.500000	110.099686	0.848706	105.965607
110.119995	105.333000	110.119682	1.040476	106.015900
110.139999	112.000000	110.139687	1.381201	106.066681
110.159996	112.500000	110.159683	1.949572	106.117706
110.180000	106.500000	110.179688	2.883198	106.169098
110.199997	121.167000	110.199684	4.399873	106.220886
110.219994	122.667000	110.219681	6.812909	106.272919
110.239998	130.167007	110.239685	10.502366	106.325348
110.259995	140.667007	110.259682	15.783645	106.378113
110.279999	147.000000	110.279686	22.653011	106.431183
110.300003	159.000000	110.299690	30.434122	106.484619
110.320000	166.667007	110.319687	37.664627	106.538361
110.340004	174.000000	110.339691	42.627182	106.592438
110.360001	180.833008	110.359688	44.456955	106.646820
110.380005	188.333008	110.379692	43.951550	106.701569
110.400002	197.833008	110.399689	43.062618	106.756622
110.419998	206.500000	110.419685	43.474949	106.811981
110.440002	204.000000	110.439690	45.613300	106.867706

110.459999	215.500000	110.459686	48.826485	106.923737
110.480003	225.667007	110.479691	52.477650	106.980072
110.500000	223.333008	110.499687	56.994743	107.036713
110.519997	226.000000	110.519684	63.861580	107.093658
110.540001	227.667007	110.539688	74.245132	107.151001
110.559998	223.000000	110.559685	87.221863	107.208527
110.580002	227.333008	110.579689	99.133430	107.266449
110.599998	224.333008	110.599686	104.959251	107.324646
110.619995	219.500000	110.619682	101.424973	107.383148
110.639999	214.167007	110.639687	89.330887	107.441956
110.659996	207.667007	110.659683	72.826279	107.501068
110.680000	200.667007	110.679688	56.354225	107.560486
110.699997	194.000000	110.699684	42.490162	107.620148
110.719994	176.500000	110.719681	31.842236	107.680145
110.739998	171.667007	110.739685	24.071529	107.740417
110.759995	173.500000	110.759682	18.688526	107.800995
110.779999	157.500000	110.779694	15.314832	107.861877
110.800003	150.000000	110.799698	13.674798	107.923065
110.820000	143.500000	110.819695	13.436109	107.984406
110.840004	151.000000	110.839699	14.072313	108.046143
110.860001	138.333008	110.859695	14.828223	108.108093
110.880005	140.667007	110.879700	14.921181	108.170380
110.900002	134.167007	110.899696	13.918870	108.232849
110.919998	133.333008	110.919693	11.969376	108.295654
110.940002	126.667000	110.939697	9.615380	108.358673
110.959999	121.000000	110.959694	7.386806	108.421936
110.980003	127.667000	110.979698	5.549229	108.485504
111.000000	123.833000	110.999695	4.137545	108.549316
111.019997	116.333000	111.019691	3.082634	108.613373
111.040001	117.500000	111.039696	2.305408	108.677704
111.059998	106.167000	111.059692	1.747043	108.742218
111.080002	102.167000	111.079697	1.367658	108.807037
111.099998	103.500000	111.099693	1.144134	108.872040
111.119995	112.667000	111.119690	1.069051	108.937286
111.139999	111.500000	111.139694	1.156241	109.002777
111.159996	106.333000	111.159691	1.448673	109.068512
111.180000	110.333000	111.179695	2.027821	109.134460
111.199997	112.667000	111.199692	3.015440	109.200592

111.219994	114.167000	111.219688	4.556829	109.266998
111.239998	115.000000	111.239693	6.759546	109.333618
111.259995	118.333000	111.259689	9.569254	109.400421
111.279999	122.833000	111.279694	12.630693	109.467407
111.300003	123.667000	111.299698	15.240734	109.534637
111.320000	132.500000	111.319695	16.585566	109.602051
111.340004	123.000000	111.339699	16.213289	109.669678
111.360001	117.500000	111.359695	14.358164	109.737457
111.380005	120.333000	111.379700	11.761428	109.805481
111.400002	120.000000	111.399696	9.172794	109.873657
111.419998	112.500000	111.419693	7.011011	109.942017
111.440002	119.500000	111.439697	5.380172	110.010529
111.459999	110.000000	111.459694	4.236207	110.079193
111.480003	115.167000	111.479698	3.502692	110.148071
111.500000	111.000000	111.499695	3.106134	110.217072
111.519997	107.000000	111.519691	2.956506	110.286255
111.540001	104.333000	111.539696	2.929297	110.355591
111.559998	103.333000	111.559692	2.878957	110.425049
111.580002	106.000000	111.579697	2.693799	110.494720
111.599998	109.333000	111.599701	2.352104	110.564484
111.619995	98.500000	111.619698	1.920149	110.634369
111.639999	103.333000	111.639702	1.490111	110.704407
111.659996	104.833000	111.659698	1.123003	110.774567
111.680000	97.500000	111.679703	0.834962	110.844849
111.699997	101.500000	111.699699	0.616920	110.915222
111.719994	102.833000	111.719696	0.453185	110.985748
111.739998	106.667000	111.739700	0.330536	111.056335
111.759995	97.000000	111.759697	0.239460	111.127045
111.779999	97.833298	111.779701	0.172758	111.197876
111.800003	105.833000	111.799706	0.124843	111.268829
111.820000	99.166695	111.819702	0.091158	111.339752
111.840004	99.500000	111.839706	0.068017	111.410858
111.860001	99.666695	111.859703	0.052603	111.481964
111.880005	97.166695	111.879707	0.042806	111.553223
111.900002	94.666695	111.899704	0.037159	111.624512
111.919998	102.500000	111.919701	0.034689	111.695831
111.940002	102.167000	111.939705	0.034826	111.767242
111.959999	98.666695	111.959702	0.037324	111.838684

111.980003	98.500000	111.979706	0.042207	111.910187
112.000000	108.500000	111.999702	0.049742	111.981720
112.019997	102.833000	112.019699	0.060460	112.053253
112.040001	103.000000	112.039703	0.075189	112.124878
112.059998	99.666695	112.059700	0.095109	112.196442
112.080002	101.167000	112.079704	0.121914	112.268097
112.099998	96.000000	112.099701	0.157958	112.339722
112.119995	102.167000	112.119698	0.206670	112.411346
112.139999	102.000000	112.139702	0.273195	112.483002
112.159996	106.833000	112.159698	0.365465	112.554596
112.180000	103.333000	112.179703	0.496556	112.626282
112.199997	108.500000	112.199699	0.688188	112.697845
112.219994	110.167000	112.219696	0.977466	112.769409
112.239998	101.000000	112.239700	1.427009	112.840973
112.259995	106.833000	112.259697	2.138319	112.912476
112.279999	117.500000	112.279701	3.268828	112.983978
112.300003	113.333000	112.299706	5.038164	113.055389
112.320000	120.667000	112.319702	7.707312	113.126740
112.340004	132.500000	112.339706	11.501696	113.198029
112.360001	135.167007	112.359703	16.439451	113.269257
112.380005	149.333008	112.379707	22.143869	113.340424
112.400002	146.833008	112.399712	27.775692	113.411530
112.419998	159.500000	112.419708	32.359543	113.482513
112.440002	164.000000	112.439713	35.434513	113.553406
112.459999	172.667007	112.459709	37.438915	113.624176
112.480003	170.500000	112.479713	39.281590	113.694855
112.500000	172.667007	112.499710	41.329613	113.765411
112.519997	174.167007	112.519707	42.834354	113.835846
112.540001	172.500000	112.539711	42.410118	113.906189
112.559998	170.833008	112.559708	39.197395	113.976349
112.580002	159.667007	112.579712	33.620815	114.046387
112.599998	150.500000	112.599709	27.057972	114.116241
112.619995	154.333008	112.619705	20.858362	114.185974
112.639999	145.667007	112.639709	15.732492	114.255585
112.659996	139.167007	112.659706	11.797997	114.324982
112.680000	132.167007	112.679710	8.889050	114.394196
112.699997	126.833000	112.699707	6.806435	114.463196
112.719994	123.833000	112.719704	5.386805	114.532104

112.739998	125.167000	112.739708	4.494786	114.600739
112.759995	126.833000	112.759705	3.987914	114.669189
112.779999	114.833000	112.779709	3.695955	114.737427
112.800003	118.500000	112.799713	3.443613	114.805450
112.820000	118.500000	112.819710	3.107232	114.873230
112.840004	110.500000	112.839714	2.662266	114.940796
112.860001	108.167000	112.859711	2.170690	115.008118
112.880005	114.000000	112.879715	1.715449	115.075165
112.900002	114.000000	112.899712	1.348841	115.141998
112.919998	102.833000	112.919708	1.083795	115.208527
112.940002	106.500000	112.939713	0.914724	115.274811
112.959999	107.000000	112.959709	0.837753	115.340790
112.980003	104.667000	112.979713	0.861575	115.406494
113.000000	110.833000	112.999710	1.013627	115.471924
113.019997	102.833000	113.019707	1.344768	115.537018
113.040001	104.167000	113.039711	1.931900	115.601837
113.059998	111.000000	113.059708	2.869307	115.666290
113.080002	120.833000	113.079712	4.239517	115.730469
113.099998	117.833000	113.099709	6.043556	115.794281
113.119995	122.833000	113.119705	8.112014	115.857758
113.139999	117.333000	113.139709	10.048451	115.920929
113.159996	114.667000	113.159706	11.313653	115.983643
113.180000	110.167000	113.179710	11.494433	116.046082
113.199997	103.167000	113.199707	10.572592	116.108124
113.219994	116.333000	113.219711	8.937412	116.169830
113.239998	109.167000	113.239716	7.112570	116.231110
113.259995	109.167000	113.259712	5.469264	116.292023
113.279999	116.167000	113.279716	4.144798	116.352448
113.300003	116.333000	113.299721	3.129567	116.412567
113.320000	113.000000	113.319717	2.365963	116.472198
113.340004	110.833000	113.339722	1.799724	116.531433
113.360001	105.667000	113.359718	1.393147	116.590179
113.380005	107.167000	113.379723	1.120175	116.648560
113.400002	99.000000	113.399719	0.964378	116.706451
113.419998	103.167000	113.419716	0.918188	116.763824
113.440002	105.333000	113.439720	0.986674	116.820801
113.459999	112.167000	113.459717	1.193244	116.877319
113.480003	105.333000	113.479721	1.588584	116.933289

113.500000	105.500000	113.499718	2.260318	116.988800
113.519997	110.000000	113.519714	3.342158	117.043793
113.540001	124.167000	113.539719	5.010464	117.098328
113.559998	123.167000	113.559715	7.448403	117.152252
113.580002	122.500000	113.579720	10.771058	117.205719
113.599998	134.167007	113.599716	14.901067	117.258636
113.619995	135.833008	113.619713	19.502829	117.311005
113.639999	149.167007	113.639717	24.087996	117.362854
113.659996	149.833008	113.659714	28.346039	117.414093
113.680000	162.000000	113.679718	32.459061	117.464813
113.699997	171.500000	113.699715	36.915401	117.514923
113.719994	179.833008	113.719711	41.818344	117.564484
113.739998	187.833008	113.739716	46.265175	117.613434
113.759995	174.000000	113.759712	48.550583	117.661774
113.779999	178.000000	113.779716	47.265949	117.709534
113.800003	175.000000	113.799721	42.384029	117.756653
113.820000	165.833008	113.819717	35.310570	117.803192
113.840004	163.833008	113.839722	27.867306	117.849060
113.860001	144.333008	113.859718	21.310457	117.894226
113.880005	137.167007	113.879723	16.061941	117.938843
113.900002	130.833008	113.899719	12.038927	117.982758
113.919998	132.000000	113.919716	8.997754	118.026001
113.940002	115.167000	113.939720	6.720986	118.068573
113.959999	127.667000	113.959717	5.056142	118.110504
113.980003	121.000000	113.979721	3.898508	118.151703
114.000000	122.833000	113.999718	3.185024	118.192200
114.019997	114.333000	114.019722	2.891293	118.232025
114.040001	121.000000	114.039726	3.043149	118.271118
114.059998	122.667000	114.059723	3.725037	118.309479
114.080002	129.167007	114.079727	5.080256	118.347137
114.099998	128.333008	114.099724	7.275214	118.384003
114.119995	137.167007	114.119720	10.410478	118.420197
114.139999	137.667007	114.139725	14.359278	118.455536
114.159996	132.667007	114.159721	18.587528	118.490173
114.180000	143.333008	114.179726	22.143650	118.524048
114.199997	142.000000	114.199722	23.964823	118.557159
114.219994	135.667007	114.219719	23.472490	118.589447
114.239998	138.333008	114.239723	20.958361	118.620941

114.259995	130.667007	114.259720	17.377571	118.651642
114.279999	126.000000	114.279724	13.720639	118.681519
114.300003	125.833000	114.299728	10.575109	118.710571
114.320000	124.333000	114.319725	8.099937	118.738831
114.340004	120.000000	114.339729	6.226467	118.766205
114.360001	121.333000	114.359726	4.839694	118.792786
114.380005	124.500000	114.379730	3.845446	118.818542
114.400002	115.667000	114.399727	3.184454	118.843384
114.419998	113.167000	114.419724	2.822368	118.867401
114.440002	115.000000	114.439728	2.749289	118.890533
114.459999	120.833000	114.459724	2.987073	118.912781
114.480003	121.833000	114.479729	3.607953	118.934113
114.500000	124.333000	114.499725	4.764900	118.954590
114.519997	132.500000	114.519722	6.739558	118.974152
114.540001	138.167007	114.539726	10.003829	118.992828
114.559998	147.500000	114.559723	15.273453	119.010590
114.580002	163.000000	114.579727	23.527248	119.027374
114.599998	195.000000	114.599724	35.846970	119.043274
114.619995	222.167007	114.619720	53.000477	119.058228
114.639999	242.500000	114.639725	74.627411	119.072235
114.659996	270.166992	114.659721	98.251526	119.085297
114.680000	274.000000	114.679726	119.044174	119.097412
114.699997	284.332977	114.699722	131.170441	119.108551
114.719994	291.832977	114.719719	130.895508	119.118744
114.739998	281.832977	114.739723	119.044128	119.127930
114.759995	257.166992	114.759720	100.478523	119.136139
114.779999	249.500000	114.779724	80.888466	119.143372
114.800003	231.000000	114.799728	64.057243	119.149597
114.820000	208.000000	114.819733	51.283279	119.154816
114.840004	182.000000	114.839737	42.291359	119.159027
114.860001	171.000000	114.859734	36.164631	119.162262
114.880005	165.667007	114.879738	31.787388	119.164429
114.900002	159.000000	114.899734	28.086943	119.165588
114.919998	148.333008	114.919731	24.295685	119.165710
114.940002	141.167007	114.939735	20.194656	119.164825
114.959999	139.500000	114.959732	16.063850	119.162842
114.980003	142.833008	114.979736	12.331450	119.159821
115.000000	142.667007	114.999733	9.268789	119.155792

115.019997	143.833008	115.019730	6.903888	119.150665
115.040001	145.500000	115.039734	5.125005	119.144501
115.059998	139.000000	115.059731	3.793754	119.137238
115.080002	127.667000	115.079735	2.794522	119.128937
115.099998	129.000000	115.099731	2.046042	119.119537
115.119995	129.667007	115.119728	1.489205	119.109070
115.139999	132.167007	115.139732	1.079497	119.097504
115.159996	119.333000	115.159729	0.782061	119.084839
115.180000	118.333000	115.179733	0.568484	119.071075
115.199997	114.667000	115.199730	0.416699	119.056244
115.219994	110.333000	115.219727	0.309555	119.040253
115.239998	106.167000	115.239731	0.234327	119.023193
115.259995	113.167000	115.259727	0.181890	119.005005
115.279999	107.500000	115.279732	0.145740	118.985718
115.300003	110.000000	115.299736	0.121485	118.965302
115.320000	109.500000	115.319733	0.106220	118.943787
115.340004	106.167000	115.339737	0.098128	118.921082
115.360001	104.167000	115.359734	0.096238	118.897308
115.380005	108.167000	115.379738	0.100221	118.872375
115.400002	105.833000	115.399734	0.110311	118.846313
115.419998	104.000000	115.419731	0.127295	118.819122
115.440002	104.667000	115.439735	0.152593	118.790802
115.459999	111.667000	115.459732	0.188413	118.761322
115.480003	103.833000	115.479736	0.238231	118.730743
115.500000	111.833000	115.499733	0.307446	118.698944
115.519997	95.000000	115.519730	0.404947	118.666107
115.540001	101.500000	115.539734	0.545699	118.632019
115.559998	105.000000	115.559731	0.755002	118.596802
115.580002	108.500000	115.579735	1.076148	118.560486
115.599998	110.000000	115.599731	1.580309	118.523010
115.619995	111.167000	115.619736	2.381031	118.484344
115.639999	113.000000	115.639740	3.645127	118.444519
115.659996	126.167000	115.659737	5.591673	118.403564
115.680000	132.000000	115.679741	8.459312	118.361420
115.699997	131.333008	115.699738	12.399732	118.318207
115.719994	140.667007	115.719734	17.306719	118.273773
115.739998	149.667007	115.739738	22.617146	118.228241
115.759995	148.500000	115.759735	27.262640	118.181549

115.779999	149.333008	115.779739	30.008451	118.133698
115.800003	151.333008	115.799744	30.097816	118.084686
115.820000	150.833008	115.819740	27.782515	118.034515
115.840004	147.833008	115.839745	24.193577	117.983215
115.860001	147.000000	115.859741	20.688988	117.930817
115.880005	140.500000	115.879745	18.272999	117.877167
115.900002	141.333008	115.899742	17.488064	117.822479
115.919998	145.833008	115.919739	18.563932	117.766663
115.940002	142.000000	115.939743	21.513441	117.709656
115.959999	150.667007	115.959740	26.012089	117.651550
115.980003	147.500000	115.979744	31.200943	117.592316
116.000000	147.333008	115.999741	35.658825	117.531952
116.019997	145.833008	116.019737	37.855644	117.470490
116.040001	148.667007	116.039742	36.938301	117.407867
116.059998	141.167007	116.059738	33.275784	117.344208
116.080002	146.667007	116.079742	28.182682	117.279388
116.099998	137.667007	116.099739	23.072643	117.213470
116.119995	147.167007	116.119736	18.774723	117.146484
116.139999	135.333008	116.139740	15.447412	117.078400
116.159996	124.333000	116.159737	12.859237	117.009277
116.180000	118.833000	116.179741	10.693749	116.939026
116.199997	121.167000	116.199738	8.752026	116.867706
116.219994	117.000000	116.219734	6.996856	116.795319
116.239998	121.500000	116.239738	5.494384	116.721893
116.259995	115.000000	116.259735	4.321887	116.647430
116.279999	110.000000	116.279739	3.515783	116.571930
116.300003	113.833000	116.299744	3.085724	116.495392
116.320000	111.833000	116.319740	3.045631	116.417816
116.340004	119.500000	116.339745	3.434361	116.339233
116.360001	119.500000	116.359741	4.306911	116.259674
116.380005	120.833000	116.379745	5.693071	116.179047
116.400002	121.000000	116.399742	7.519786	116.097534
116.419998	129.000000	116.419746	9.535164	116.014984
116.440002	122.167000	116.439751	11.289178	115.931488
116.459999	126.500000	116.459747	12.267432	115.847015
116.480003	124.500000	116.479752	12.150491	115.761627
116.500000	119.333000	116.499748	11.012877	115.675323
116.519997	118.667000	116.519745	9.268761	115.588104

116.540001	117.833000	116.539749	7.403307	115.499969
116.559998	118.000000	116.559746	5.741127	115.410950
116.580002	107.667000	116.579750	4.392884	115.321014
116.599998	100.667000	116.599747	3.341227	115.230255
116.619995	117.500000	116.619743	2.527210	115.138641
116.639999	107.500000	116.639755	1.897555	115.046173
116.659996	106.500000	116.659752	1.415158	114.952881
116.680000	108.667000	116.679756	1.051871	114.858826
116.699997	115.667000	116.699753	0.786143	114.763947
116.719994	105.833000	116.719749	0.599395	114.668335
116.739998	103.500000	116.739754	0.476288	114.571930
116.759995	105.167000	116.759750	0.405243	114.474823
116.779999	107.667000	116.779755	0.378956	114.376923
116.800003	107.167000	116.799759	0.395718	114.278351
116.820000	107.000000	116.819756	0.461016	114.179138
116.840004	106.167000	116.839760	0.590582	114.079254
116.860001	117.667000	116.859756	0.814557	113.978668
116.880005	107.333000	116.879761	1.183593	113.877533
116.900002	117.000000	116.899757	1.773070	113.775757
116.919998	115.500000	116.919754	2.683228	113.673462
116.940002	117.333000	116.939758	4.023277	113.570496
116.959999	113.333000	116.959755	5.865379	113.467072
116.980003	126.833000	116.979759	8.171519	113.363098
117.000000	121.667000	116.999756	10.702057	113.258636
117.019997	128.333008	117.019753	13.000183	113.153778
117.040001	133.500000	117.039757	14.521496	113.048340
117.059998	128.667007	117.059753	14.910358	112.942596
117.080002	127.667000	117.079758	14.233484	112.836395
117.099998	121.833000	117.099754	12.933115	112.729828
117.119995	128.167007	117.119751	11.526361	112.622955
117.139999	123.167000	117.139755	10.329443	112.515747
117.159996	127.667000	117.159752	9.418415	112.408234
117.180000	126.333000	117.179756	8.774486	112.300446
117.199997	134.667007	117.199753	8.440506	112.192474
117.219994	126.833000	117.219757	8.547182	112.084320
117.239998	135.333008	117.239761	9.215486	111.975922
117.259995	126.167000	117.259758	10.414849	111.867462
117.279999	128.000000	117.279762	11.873856	111.758789

117.300003	130.333008	117.299767	13.103683	111.650116
117.320000	129.667007	117.319763	13.581653	111.541351
117.340004	126.167000	117.339767	13.024298	111.432648
117.360001	124.333000	117.359764	11.553498	111.323914
117.380005	125.667000	117.379768	9.596596	111.215240
117.400002	118.833000	117.399765	7.615078	111.106689
117.419998	118.833000	117.419762	5.891132	110.998260
117.440002	116.667000	117.439766	4.505811	110.889954
117.459999	113.333000	117.459763	3.426605	110.781921
117.480003	112.667000	117.479767	2.588665	110.674042
117.500000	114.167000	117.499763	1.937879	110.566498
117.519997	112.833000	117.519760	1.434840	110.459320
117.540001	114.333000	117.539764	1.050940	110.352386
117.559998	109.833000	117.559761	0.763492	110.245880
117.580002	111.833000	117.579765	0.552441	110.139832
117.599998	104.833000	117.599762	0.400778	110.034271
117.619995	109.000000	117.619759	0.294028	109.929260
117.639999	107.167000	117.639763	0.220655	109.824738
117.659996	107.833000	117.659760	0.171935	109.720917
117.680000	106.333000	117.679764	0.141461	109.617706
117.699997	102.500000	117.699760	0.124907	109.515228
117.719994	105.500000	117.719757	0.119555	109.413483
117.739998	104.000000	117.739761	0.124073	109.312469
117.759995	104.667000	117.759758	0.138354	109.212402
117.779999	103.833000	117.779762	0.163638	109.113159
117.800003	98.166695	117.799767	0.202900	109.014862
117.820000	107.500000	117.819763	0.261786	108.917603
117.840004	112.333000	117.839767	0.350451	108.821381
117.860001	111.500000	117.859764	0.486289	108.726288
117.880005	107.000000	117.879768	0.698700	108.632294
117.900002	114.000000	117.899765	1.034636	108.539520
117.919998	115.333000	117.919762	1.565530	108.448090
117.940002	109.000000	117.939766	2.390693	108.357880
117.959999	118.167000	117.959763	3.628303	108.269135
117.980003	115.667000	117.979767	5.386784	108.181763
118.000000	118.167000	117.999771	7.695153	108.095856
118.019997	118.833000	118.019768	10.407194	108.011566
118.040001	117.333000	118.039772	13.128832	107.928864

118.059998	126.167000	118.059769	15.250776	107.847870
118.080002	124.000000	118.079773	16.181440	107.768524
118.099998	128.167007	118.099770	15.664121	107.691101
118.119995	120.500000	118.119766	13.945459	107.615479
118.139999	123.500000	118.139771	11.610723	107.541840
118.159996	123.667000	118.159767	9.243027	107.470123
118.180000	113.167000	118.179771	7.182213	107.400513
118.199997	115.167000	118.199768	5.522990	107.333038
118.219994	110.833000	118.219765	4.222479	107.267792
118.239998	118.833000	118.239769	3.205168	107.204803
118.259995	107.500000	118.259766	2.408955	107.144135
118.279999	108.333000	118.279770	1.788925	107.085907
118.300003	110.500000	118.299774	1.313016	107.030212
118.320000	110.000000	118.319771	0.954762	106.977020
118.340004	114.167000	118.339775	0.690466	106.926514
118.360001	115.167000	118.359772	0.499678	106.878693
118.380005	112.000000	118.379776	0.364683	106.833710
118.400002	115.333000	118.399773	0.271356	106.791626
118.419998	105.667000	118.419769	0.208666	106.752472
118.440002	109.333000	118.439774	0.168540	106.716339
118.459999	108.333000	118.459770	0.145394	106.683380
118.480003	112.833000	118.479774	0.135570	106.653595
118.500000	105.000000	118.499771	0.137040	106.627136
118.519997	108.667000	118.519768	0.149111	106.604034
118.540001	107.333000	118.539772	0.172403	106.584412
118.559998	112.500000	118.559769	0.209018	106.568359
118.580002	106.000000	118.579773	0.263239	106.555939
118.599998	114.167000	118.599770	0.342695	106.547241
118.619995	111.500000	118.619766	0.460784	106.542419
118.639999	110.000000	118.639771	0.640474	106.541473
118.659996	115.167000	118.659767	0.919757	106.544586
118.680000	108.500000	118.679771	1.359896	106.551788
118.699997	115.667000	118.699768	2.052622	106.563232
118.719994	112.000000	118.719765	3.125294	106.578949
118.739998	120.333000	118.739769	4.731451	106.599121
118.759995	126.500000	118.759766	7.009467	106.623810
118.779999	131.333008	118.779778	10.005767	106.653107
118.800003	131.333008	118.799782	13.543350	106.687103

118.820000	134.500000	118.819778	17.130409	106.725983
118.840004	132.833008	118.839783	19.999697	106.769775
118.860001	131.833008	118.859779	21.371183	106.818573
118.880005	145.500000	118.879784	20.867714	106.872589
118.900002	141.333008	118.899780	18.749445	106.931854
118.919998	133.333008	118.919777	15.746440	106.996460
118.940002	133.167007	118.939781	12.626686	107.066589
118.959999	126.833000	118.959778	9.874524	107.142365
118.980003	122.333000	118.979782	7.641068	107.223846
119.000000	117.833000	118.999779	5.887756	107.311127
119.019997	110.833000	119.019775	4.518123	107.404388
119.040001	107.500000	119.039780	3.449256	107.503784
119.059998	110.833000	119.059776	2.625743	107.609283
119.080002	109.500000	119.079781	2.010123	107.721283
119.099998	114.667000	119.099777	1.578156	107.839630
119.119995	111.500000	119.119774	1.314525	107.964569
119.139999	99.666695	119.139778	1.214707	108.096283
119.159996	118.333000	119.159775	1.285666	108.234833
119.180000	112.000000	119.179779	1.542881	108.380371
119.199997	107.333000	119.199776	1.999436	108.533051
119.219994	112.167000	119.219772	2.645315	108.692932
119.239998	107.500000	119.239777	3.419107	108.860260
119.259995	112.833000	119.259773	4.187768	109.035095
119.279999	109.500000	119.279778	4.766283	109.217682
119.300003	109.167000	119.299782	4.985236	109.408051
119.320000	113.500000	119.319778	4.782208	109.606323
119.340004	114.000000	119.339783	4.239228	109.812775
119.360001	108.500000	119.359779	3.530124	110.027466
119.380005	113.833000	119.379784	2.820492	110.250549
119.400002	112.500000	119.399780	2.206219	110.482239
119.419998	106.833000	119.419777	1.711685	110.722534
119.440002	106.500000	119.439781	1.323524	110.971863
119.459999	109.500000	119.459778	1.019863	111.230072
119.480003	102.833000	119.479782	0.782264	111.497620
119.500000	108.667000	119.499779	0.598405	111.774353
119.519997	102.667000	119.519775	0.459352	112.060577
119.540001	115.667000	119.539780	0.358050	112.356720
119.559998	109.333000	119.559776	0.288568	112.662476

119.580002	107.333000	119.579788	0.245776	112.978546
119.599998	110.833000	119.599785	0.225863	113.304504
119.619995	113.667000	119.619781	0.226407	113.640930
119.639999	108.000000	119.639786	0.246948	113.987915
119.659996	113.500000	119.659782	0.289544	114.345581
119.680000	116.167000	119.679787	0.360019	114.714142
119.699997	114.167000	119.699783	0.469900	115.093781
119.719994	111.167000	119.719780	0.640039	115.484528
119.739998	111.667000	119.739784	0.905949	115.886993
119.759995	119.500000	119.759781	1.324610	116.300812
119.779999	111.167000	119.779785	1.983362	116.726776
119.800003	115.333000	119.799789	3.003952	117.164551
119.820000	118.167000	119.819786	4.535916	117.614471
119.840004	127.500000	119.839790	6.727084	118.076904
119.860001	132.333008	119.859787	9.646439	118.551849
119.880005	131.167007	119.879791	13.178023	119.039673
119.900002	142.333008	119.899788	16.902771	119.540482
119.919998	135.667007	119.919785	20.104219	120.054291
119.940002	147.667007	119.939789	21.972158	120.581818
119.959999	134.667007	119.959785	21.987089	121.122711
119.980003	137.833008	119.979790	20.231552	121.677719
120.000000	136.167007	119.999786	17.339878	122.246460

```
_reflns_number_total      288
_reflns_limit_h_min       0
_reflns_limit_h_max       10
_reflns_limit_k_min       -8
_reflns_limit_k_max       0
_reflns_limit_l_min       0
_reflns_limit_l_max       7
_reflns_d_resolution_high 8.153
_reflns_d_resolution_low  .890

loop_
      _refln_index_h
      _refln_index_k
      _refln_index_l
      _refln_F_squared_meas
```

_refln_d_spacing

_refln_intensity_meas

1	0	0	56.529	8.15325	33813.727
1	0	1	43.582	5.25961	21356.488
2	-1	0	85.816	4.70728	16730.023
2	0	0	179.290	4.07662	25925.045
2	-1	1	98.530	3.88558	25771.340
2	0	1	55.999	3.50762	11805.772
0	0	2	3251.416	3.44168	109739.977
1	0	2	164.303	3.17076	27936.047
3	-2	0	329.405	3.08164	26341.648
3	-1	0	328.495	3.08164	26268.855
3	-2	1	1263.045	2.81263	165781.109
3	-1	1	1261.544	2.81263	165583.938
2	-1	2	1381.674	2.77829	176560.641
3	0	0	3418.696	2.71775	208166.469
2	0	2	767.012	2.62980	86903.391
3	0	1	209.874	2.52785	21792.100
4	-2	0	60.825	2.35364	2693.581
3	-2	2	141.887	2.29582	11884.020
3	-1	2	141.839	2.29582	11880.032
4	-3	0	1123.453	2.26130	45469.637
4	-1	0	1132.250	2.26130	45825.680
4	-2	1	103.325	2.22705	8080.240
1	0	3	74.529	2.20866	5719.956
4	-3	1	193.956	2.14835	13978.551
4	-1	1	194.047	2.14834	13985.095
3	0	2	137.158	2.13292	9724.176
2	-1	3	368.192	2.06249	24175.209
4	0	0	295.856	2.03831	9453.528
2	0	3	343.279	1.99951	20988.691
4	0	1	343.385	1.95442	19918.443
4	-2	2	2329.105	1.94279	133249.141
4	-3	2	575.566	1.88988	30885.547
4	-1	2	574.195	1.88988	30811.959
5	-3	0	434.659	1.87048	11385.703
5	-2	0	434.749	1.87048	11388.035
3	-2	3	1537.471	1.84036	77557.094

3	-1	3	1536.847	1.84036	77525.570
5	-3	1	842.902	1.80503	40638.129
5	-2	1	843.470	1.80503	40665.512
5	-4	0	1309.912	1.77918	30529.592
5	-1	0	1310.547	1.77918	30544.369
4	0	2	1428.122	1.75381	64367.051
3	0	3	1430.005	1.75320	64399.480
5	-4	1	901.565	1.72257	38957.598
5	-1	1	901.713	1.72257	38963.965
0	0	4	10816.827	1.72084	77717.680
1	0	4	196.709	1.68375	8057.094
5	-3	2	432.688	1.64345	16742.578
5	-2	2	432.901	1.64345	16750.826
4	-2	3	876.052	1.64295	33873.980
5	0	0	215.905	1.63065	4101.148
2	-1	4	543.140	1.61623	20208.307
4	-3	3	309.909	1.61058	11436.199
4	-1	3	309.607	1.61058	11425.050
5	0	1	412.854	1.58673	14711.436
2	0	4	487.312	1.58538	17329.957
5	-4	2	179.120	1.58049	6323.974
5	-1	2	179.112	1.58049	6323.686
6	-3	0	398.660	1.56909	6919.282
6	-4	0	908.143	1.54082	15106.249
6	-2	0	909.118	1.54082	15122.460
6	-3	1	685.028	1.52985	22413.264
4	0	3	341.195	1.52385	11061.781
6	-4	1	727.952	1.50361	22878.521
6	-2	1	728.200	1.50361	22886.285
3	-2	4	735.678	1.50246	23080.273
3	-1	4	735.619	1.50246	23078.428
5	0	2	1817.872	1.47362	54531.746
6	-5	0	537.437	1.46437	7944.889
6	-1	0	538.252	1.46437	7956.923
3	0	4	2884.847	1.45390	83899.086
5	-2	3	1352.734	1.44978	39086.234
5	-3	3	1352.734	1.44978	39086.234
6	-5	1	984.002	1.43231	27656.061

6	-1	1	985.183	1.43231	27689.244
6	-3	2	1602.984	1.42772	44724.742
6	-4	2	424.411	1.40632	11443.693
6	-2	2	425.088	1.40632	11461.961
5	-4	3	420.809	1.40600	11340.870
5	-1	3	420.510	1.40600	11332.800
4	-2	4	125.396	1.38915	3289.240
4	-3	4	103.444	1.36941	2628.544
4	-1	4	103.444	1.36941	2628.544
6	0	0	348.900	1.35887	4358.108
1	0	5	167.339	1.35746	4170.914
6	-5	2	329.829	1.34747	8089.449
6	-1	2	330.047	1.34747	8094.792
7	-4	0	160.288	1.34039	1943.270
7	-3	0	160.336	1.34039	1943.858
6	0	1	117.511	1.33314	2816.218
5	0	3	140.316	1.32917	3341.253
2	-1	5	331.036	1.32133	7783.675
7	-4	1	688.854	1.31567	16050.317
7	-3	1	689.369	1.31567	16062.327
4	0	4	1374.138	1.31490	31977.729
7	-5	0	1060.475	1.30556	12155.385
7	-2	0	1060.738	1.30556	12158.392
2	0	5	1022.349	1.30431	23389.549
6	-3	3	168.424	1.29519	3797.350
7	-5	1	628.654	1.28270	13893.963
7	-2	1	628.693	1.28270	13894.820
6	-4	3	824.539	1.27915	18120.885
6	-2	3	823.915	1.27915	18107.182
5	-3	4	420.326	1.26642	9053.157
5	-2	4	420.348	1.26642	9053.636
6	0	2	871.783	1.26392	18703.107
3	-1	5	951.935	1.25695	20200.225
3	-2	5	951.935	1.25695	20200.225
7	-4	2	543.139	1.24901	11383.457
7	-3	2	544.261	1.24901	11406.976
7	-6	0	967.232	1.24336	10047.485
7	-1	0	967.891	1.24336	10054.328

5	-4	4	1670.715	1.23693	34367.859
5	-1	4	1669.683	1.23693	34346.605
6	-5	3	1642.599	1.23439	33657.988
6	-1	3	1642.472	1.23439	33655.359
3	0	5	271.955	1.22810	5519.242
7	-6	1	842.749	1.22356	16985.805
7	-1	1	842.861	1.22356	16988.043
7	-5	2	1311.152	1.22069	26312.246
7	-2	2	1314.046	1.22069	26370.318
4	-2	5	305.250	1.18832	5841.126
5	0	4	377.400	1.18364	7173.923
8	-4	0	2415.230	1.17682	22736.748
4	-3	5	609.130	1.17590	11453.947
4	-1	5	609.130	1.17590	11453.944
7	-6	2	198.170	1.16939	3693.094
7	-1	2	198.250	1.16939	3694.581
6	0	3	399.466	1.16921	7442.625
8	-5	0	334.862	1.16475	3100.684
8	-3	0	334.956	1.16475	3101.557
7	0	0	334.956	1.16475	3101.557
8	-4	1	1641.320	1.15999	30202.920
6	-3	4	2236.406	1.15946	41124.574
7	-4	3	1152.970	1.15737	21143.049
7	-3	3	1144.241	1.15737	20982.963
8	-5	1	820.942	1.14842	14880.545
8	-3	1	821.096	1.14842	14883.330
7	0	1	821.174	1.14842	14884.733
6	-4	4	1289.562	1.14791	23359.502
6	-2	4	1299.109	1.14791	23532.438
0	0	6	15249.013	1.14723	45997.441
4	0	5	193.098	1.14084	3466.826
1	0	6	1037.304	1.13604	18513.592
7	-5	3	535.354	1.13473	9539.706
7	-2	3	535.354	1.13473	9539.702
8	-6	0	385.515	1.13065	3418.048
8	-2	0	385.481	1.13065	3417.743
8	-6	1	1629.957	1.11570	28411.061
8	-2	1	1630.392	1.11570	28418.650

6	-5	4	1752.964	1.11523	30539.295
6	-1	4	1759.793	1.11523	30658.260
2	-1	6	3536.735	1.11460	61572.902
8	-4	2	3502.112	1.11352	60898.168
5	-2	5	765.422	1.10875	13241.745
5	-3	5	765.422	1.10875	13241.746
2	0	6	3551.539	1.10433	61158.879
8	-5	2	1193.991	1.10328	20538.865
8	-3	2	1195.216	1.10328	20559.922
7	0	2	1195.624	1.10328	20566.945
7	-6	3	110.184	1.09317	1876.579
7	-1	3	110.260	1.09317	1877.868
5	-4	5	48.937	1.08879	830.079
5	-1	5	48.928	1.08879	829.926
8	-7	0	1181.935	1.07992	9946.326
8	-1	0	1183.134	1.07992	9956.412
3	-2	6	382.128	1.07514	6406.280
3	-1	6	382.170	1.07514	6406.984
8	-6	2	386.849	1.07417	6480.444
8	-2	2	387.100	1.07417	6484.646
8	-7	1	160.649	1.06687	2676.364
8	-1	1	160.682	1.06687	2676.923
6	0	4	325.880	1.06646	5427.469
7	-4	4	186.820	1.05745	3092.662
7	-3	4	186.732	1.05745	3091.213
3	0	6	345.768	1.05692	5722.034
5	0	5	282.642	1.05192	4663.505
8	-4	3	142.329	1.04712	2342.300
9	-5	0	191.577	1.04392	1573.885
9	-4	0	191.577	1.04392	1573.885
7	-5	4	417.982	1.04010	6855.951
7	-2	4	415.298	1.04010	6811.917
8	-5	3	293.181	1.03859	4805.832
8	-3	3	293.155	1.03859	4805.404
7	0	3	293.102	1.03859	4804.549
6	-3	5	1027.712	1.03484	16821.744
9	-5	1	1460.598	1.03211	23884.662
9	-4	1	1460.728	1.03211	23886.791

4	-2	6	3375.066	1.03125	55175.758
8	-7	2	1643.409	1.03039	26859.354
8	-1	2	1642.228	1.03039	26840.047
9	-3	0	860.699	1.02721	7027.096
9	-6	0	860.699	1.02721	7027.097
6	-4	5	371.379	1.02660	6063.201
6	-2	5	371.303	1.02660	6061.975
4	-3	6	611.395	1.02309	9973.621
4	-1	6	611.110	1.02309	9968.976
8	0	0	1739.165	1.01916	14175.327
9	-6	1	842.708	1.01596	13731.573
9	-3	1	844.232	1.01596	13756.421
8	-6	3	593.867	1.01420	9675.251
8	-2	3	593.812	1.01420	9674.341
8	0	1	1674.580	1.00817	27276.777
7	-6	4	867.960	1.00782	14138.046
7	-1	4	870.682	1.00782	14182.389
6	-5	5	829.796	1.00302	13520.195
6	-1	5	829.642	1.00302	13517.702
4	0	6	1144.174	0.99975	18650.125
9	-5	2	557.728	0.99897	9092.136
9	-4	2	557.458	0.99897	9087.741
9	-7	0	608.245	0.99608	4960.585
9	-2	0	608.507	0.99608	4962.719
9	-7	1	716.393	0.98581	11722.336
9	-2	1	716.513	0.98581	11724.308
9	-6	2	1239.740	0.98431	20298.645
9	-3	2	1231.534	0.98431	20164.293
5	-2	6	1348.172	0.97794	22143.902
5	-3	6	1348.172	0.97794	22143.902
8	0	2	2748.603	0.97721	45164.895
8	-7	3	1385.255	0.97711	22763.811
8	-1	3	1382.060	0.97711	22711.320
1	0	7	1973.661	0.97626	32449.082
8	-4	4	2016.850	0.97140	33263.645
6	0	5	158.855	0.96710	2628.366
8	-5	4	299.143	0.96457	4959.825
8	-3	4	299.513	0.96457	4965.964

7	0	4	299.513	0.96457	4965.966
5	-4	6	455.108	0.96417	7548.354
5	-1	6	455.034	0.96417	7547.133
2	-1	7	542.821	0.96256	9015.870
7	-3	5	417.878	0.96037	6954.710
7	-4	5	417.878	0.96037	6954.710
9	-7	2	86.418	0.95681	1443.322
9	-2	2	86.404	0.95681	1443.098
2	0	7	88.278	0.95592	1475.765
9	-8	0	88.339	0.95427	739.710
9	-1	0	88.344	0.95427	739.751
9	-5	3	306.167	0.95019	5151.463
9	-4	3	306.167	0.95019	5151.464
7	-5	5	393.002	0.94732	6636.156
7	-2	5	392.926	0.94731	6634.867
9	-8	1	524.325	0.94523	8877.847
9	-1	1	524.569	0.94523	8881.982
8	-6	4	488.879	0.94494	8280.844
8	-2	4	487.993	0.94494	8265.833
10	-5	0	1106.127	0.94146	9413.504
5	0	6	2275.886	0.93828	38919.930
9	-6	3	1307.916	0.93755	22392.096
9	-3	3	1307.916	0.93754	22392.102
3	-2	7	1193.658	0.93680	20459.707
3	-1	7	1193.658	0.93680	20459.711
10	-6	0	1512.459	0.93524	12994.335
10	-4	0	1512.367	0.93524	12993.539
10	-5	1	520.006	0.93277	8971.929
8	0	3	378.293	0.93141	6542.167
10	-6	1	535.007	0.92673	9331.071
10	-4	1	535.039	0.92673	9331.641
6	-3	6	1198.803	0.92610	20933.299
3	0	7	445.083	0.92467	7793.346
7	-6	5	131.248	0.92273	2307.010
7	-1	5	131.198	0.92273	2306.134
6	-4	6	517.750	0.92018	9148.680
6	-2	6	518.119	0.92018	9155.204
9	-8	2	533.297	0.91957	9435.528

9	-1	2	529.883	0.91957	9375.113
10	-7	0	579.015	0.91731	5147.338
10	-3	0	579.838	0.91731	5154.649
8	-7	4	1111.375	0.91472	19875.430
8	-1	4	1116.671	0.91472	19970.158
9	-7	3	1011.450	0.91369	18131.576
9	-2	3	1011.233	0.91369	18127.699
10	-7	1	281.945	0.90927	5108.599
10	-3	1	282.038	0.90927	5110.282
10	-5	2	749.304	0.90809	13617.269
4	-2	7	362.361	0.90733	6598.135
9	0	0	278.657	0.90592	2546.398
6	-5	6	148.505	0.90309	2734.887
6	-1	6	148.539	0.90309	2735.499
10	-6	2	158.327	0.90251	2920.402
10	-4	2	158.364	0.90251	2921.082
4	-3	7	83.183	0.90177	1537.553
4	-1	7	83.176	0.90177	1537.418
9	0	1	47.413	0.89817	885.521
8	-4	5	159.619	0.89453	3014.426
9	-5	4	0.457	0.89253	8.688
9	-4	4	0.457	0.89253	8.688
10	-8	0	76.771	0.88959	736.626
10	-2	0	76.790	0.88959	736.814

La2-HAp CIF FILE

```
#==============================================================================
data_global
#==============================================================================
_publ_contact_author_name  'Dr. Francesco Capitelli'
_publ_contact_author_address
;
  Institute of Crystallography - CNR
  V. Salaria Km 29.300, 00015 Monterotondo (Rome), Italy
;
```

```
_publ_contact_author_email    francesco.capitelli@ic.cnr.it
_publ_contact_author_fax     '+39 06 90672616'
_publ_contact_author_phone   '+39 06 90672630'
_journale_name_full          ?
_publ_requested_category     FI

_audit_creation_method              Expo2014

_chemical_name_systematic     ?
_chemical_formula_moiety      'H Ca5 La0.02 O13 P3'
_chemical_formula_sum         'H Ca5 La0.02 O13 P3'
_chemical_formula_weight      504.053

loop_
     _atom_type_symbol
     _atom_type_description
     _atom_type_scat_source
    'H'   'Hydrogen'   'International Tables Vol C Tables 4.2.6.8 and 6.1.1.4'
    'O'   'Oxygen'   'International Tables Vol C Tables 4.2.6.8 and 6.1.1.4'
    'P'   'Phosphorus'   'International Tables Vol C Tables 4.2.6.8 and 6.1.1.4'
    'Ca'   'Calcium'   'International Tables Vol C Tables 4.2.6.8 and 6.1.1.4'
    'La'   'Lanthanum'   'International Tables Vol C Tables 4.2.6.8 and 6.1.1.4'

_cell_length_a                  9.41472(13)
_cell_length_b                  9.41472(13)
_cell_length_c                  6.88333(18)
_cell_angle_alpha               90.000
_cell_angle_beta                90.000
_cell_angle_gamma               120.000
_cell_volume                    528.377(17)
_cell_formula_units_Z           2
_exptl_crystal_description    powder
_exptl_crystal_colour           ?
_cell_measurement_temperature    ?

_exptl_crystal_density_diffrn         3.168
_exptl_crystal_density_meas      ?
_exptl_crystal_density_method 'not measured'
```

```
_exptl_absorpt_coefficient_mu        27.721
_symmetry_Int_Tables_number         176
_symmetry_cell_setting              hexagonal
_symmetry_space_group_name_H-M      'P 63/m'
_symmetry_space_group_name_hall     '-P 6c'

loop_
    _symmetry_equiv_pos_site_id
    _symmetry_equiv_pos_as_xyz
    1   'x, y, z'
    2   'x-y, x, z+1/2'
    3   '-y, x-y, z'
    4   '-x, -y, z+1/2'
    5   '-x+y, -x, z'
    6   'y, -x+y, z+1/2'
    7   '-x, -y, -z'
    8   '-x+y, -x, -z+1/2'
    9   'y, -x+y, -z'
   10   'x, y, -z+1/2'
   11   'x-y, x, -z'
   12   '-y, x-y, -z+1/2'

loop_
    _atom_site_type_symbol
    _atom_site_label
    _atom_site_fract_x
    _atom_site_fract_y
    _atom_site_fract_z
    _atom_site_U_iso_or_equiv
    _atom_site_occupancy
    _atom_site_adp_type
Ca   Ca1 0.6667 0.3333 0.9979(4) 0.0023(4) 1.0000        Uiso
Ca   Ca2 0.25390(17) 0.24621(18) 0.7500 0.0019 0.9941(12) Uiso
La   La1 0.2539 0.2462 0.7500 0.0019(4) 0.0059(12) Uiso
P    P   0.3977(2) 0.3677(2) 1.2500 0.0005(4) 1.0000        Uiso
O    O1  0.3270(4) 0.4826(5) 1.2500 0.0022(12) 1.0000        Uiso
O    O2  0.5874(5) 0.4644(5) 1.2500 0.0042(12) 1.0000        Uiso
O    O3  0.3389(3) 0.2544(4) 1.0705(4) 0.0117(9) 1.0000        Uiso
```

```
O   O-H 0.0000 0.0000 0.7020(14) 0.006(3) 0.50000       Uiso
H   H   0.0000 0.0000 0.5629 0.0071 0.50000       Uiso

loop_
    _geom_bond_atom_site_label_1
    _geom_bond_atom_site_label_2
    _geom_bond_distance
    _geom_bond_site_symmetry_2
Ca1 O1  2.412(3) 6_554
Ca1 O1  2.412(4) 4_664
Ca1 O1  2.412(4) 2_654
Ca1 O2  2.451(4) .
Ca1 O2  2.451(3) 5_665
Ca1 O2  2.451(3) 3_655
Ca1 O3  2.834(3) .
Ca1 O3  2.834(2) 5_665
Ca1 O3  2.834(4) 3_655
Ca2 O1  2.683(5) 6_554
Ca2 O2  2.364(4) 4_664
Ca2 O3  2.335(3) 10_556
Ca2 O3  2.495(4) 2_554
Ca2 O3  2.495(4) 11_557
Ca2 O3  2.335(3) .
Ca2 O-H  2.3781(17) .
P O1  1.527(6) .
P O2  1.547(4) .
P O3  1.543(3) .
P O3  1.543(3) 10_557
O1 Ca1  2.412(4) 7_667
O1 Ca1  2.412(4) 2_555
O1 Ca2  2.683(3) 2_555
O2 Ca1  2.451(4) 8_667
O2 Ca2  2.364(4) 4_665
O3 Ca2  2.495(3) 6_555
O-H Ca2  2.378(2) 3_555
O-H Ca2  2.378(2) 5_555
O-H H  0.957472 .
```

```
loop_
_geom_angle_atom_site_label_1
_geom_angle_atom_site_label_2
_geom_angle_atom_site_label_3
_geom_angle
_geom_angle_site_symmetry_1
_geom_angle_site_symmetry_3
O1 Ca1 O1 75.45(12) 6_554 4_664
O1 Ca1 O1 75.45(12) 6_554 2_654
O1 Ca1 O2 123.78(13) 6_554 .
O1 Ca1 O2 154.54(14) 6_554 5_665
O1 Ca1 O2 92.50(13) 6_554 3_655
O1 Ca1 O3 68.42(11) 6_554 .
O1 Ca1 O3 142.77(12) 6_554 5_665
O1 Ca1 O3 86.88(11) 6_554 3_655
O1 Ca1 O1 75.45(12) 4_664 2_654
O1 Ca1 O2 92.50(13) 4_664 .
O1 Ca1 O2 123.78(13) 4_664 5_665
O1 Ca1 O2 154.54(14) 4_664 3_655
O1 Ca1 O3 86.88(11) 4_664 .
O1 Ca1 O3 68.42(11) 4_664 5_665
O1 Ca1 O3 142.77(12) 4_664 3_655
O1 Ca1 O2 154.54(14) 2_654 .
O1 Ca1 O2 92.50(13) 2_654 5_665
O1 Ca1 O2 123.78(13) 2_654 3_655
O1 Ca1 O3 142.77(12) 2_654 .
O1 Ca1 O3 86.88(11) 2_654 5_665
O1 Ca1 O3 68.42(11) 2_654 3_655
O2 Ca1 O2 75.44(13) . 5_665
O2 Ca1 O2 75.44(13) . 3_655
O2 Ca1 O3 56.06(11) . .
O2 Ca1 O3 67.75(11) . 5_665
O2 Ca1 O3 124.26(12) . 3_655
Ca1 O2 P 104.9(2) . .
Ca1 O2 Ca1 90.10(13) . 8_667
Ca1 O2 Ca2 113.83(16) . 4_665
O2 Ca1 O2 75.44(13) 5_665 3_655
O2 Ca1 O3 124.26(12) 5_665 .
```

O2 Ca1 O3 56.06(11) 5_665 5_665

O2 Ca1 O3 67.75(11) 5_665 3_655

O2 Ca1 O3 67.75(11) 3_655 .

O2 Ca1 O3 124.26(12) 3_655 5_665

O2 Ca1 O3 56.06(11) 3_655 3_655

O3 Ca1 O3 116.96(10) . 5_665

O3 Ca1 O3 116.96(10) . 3_655

Ca1 O3 Ca2 98.77(11) . .

Ca1 O3 P 89.58(14) . .

Ca1 O3 Ca2 98.22(11) . 6_555

O3 Ca1 O3 116.96(10) 5_665 3_655

O1 Ca2 O2 102.78(13) 6_554 4_664

O1 Ca2 O3 72.20(11) 6_554 10_556

O1 Ca2 O3 150.01(12) 6_554 2_554

O1 Ca2 O3 150.01(12) 6_554 11_557

O1 Ca2 O3 72.20(11) 6_554 .

O1 Ca2 O-H 105.39(16) 6_554 .

O2 Ca2 O3 87.32(13) 4_664 10_556

O2 Ca2 O3 75.11(12) 4_664 2_554

O2 Ca2 O3 75.11(12) 4_664 11_557

O2 Ca2 O3 87.32(13) 4_664 .

O2 Ca2 O-H 150.66(17) 4_664 .

O3 Ca2 O3 77.81(11) 10_556 2_554

O3 Ca2 O3 136.46(12) 10_556 11_557

O3 Ca2 O3 141.78(12) 10_556 .

O3 Ca2 O-H 93.54(16) 10_556 .

O3 Ca2 O3 59.38(10) 2_554 11_557

O3 Ca2 O3 136.46(12) 2_554 .

O3 Ca2 O-H 76.42(15) 2_554 .

O3 Ca2 O3 77.81(11) 11_557 .

O3 Ca2 O-H 84.43(15) 11_557 .

O3 Ca2 O-H 108.93(16) . .

Ca2 O3 P 141.09(19) . .

Ca2 O3 Ca2 119.39(13) . 6_555

Ca2 O-H Ca2 118.1(2) . 3_555

Ca2 O-H Ca2 118.1(2) . 5_555

Ca2 O-H H 97.99 . .

O1 P O2 111.5(2) . .

```
O1 P O3 110.9(2) . .
O1 P O3 110.9(2) . 10_557
P O1 Ca1 130.2(2) . 7_667
P O1 Ca1 130.2(2) . 2_555
P O1 Ca2 98.14(17) . 2_555
O2 P O3 108.4(2) . .
O2 P O3 108.4(2) . 10_557
P O2 Ca1 104.9(2) . 8_667
P O2 Ca2 123.8(2) . 4_665
O3 P O3 106.42(19) . 10_557
P O3 Ca2 96.56(15) . 6_555
Ca1 O1 Ca1 90.10(13) 7_667 2_555
Ca1 O1 Ca2 101.07(13) 7_667 2_555
Ca1 O1 Ca2 101.07(13) 2_555 2_555
Ca1 O2 Ca2 113.83(16) 8_667 4_665
Ca2 O-H Ca2 118.1(2) 3_555 5_555
Ca2 O-H H      97.99   3_555       .
Ca2 O-H H      97.99   5_555       .

loop_
   _geom_torsion_atom_site_label_1
   _geom_torsion_atom_site_label_2
   _geom_torsion_atom_site_label_3
   _geom_torsion_atom_site_label_4
   _geom_torsion
   _geom_torsion_site_symmetry_1
   _geom_torsion_site_symmetry_2
   _geom_torsion_site_symmetry_3
   _geom_torsion_site_symmetry_4
O1 Ca1 O1 Ca2 -62.09(12) 4_664 . 6_554 .
O1 Ca1 O1 Ca2 -140.51(13) 2_654 . 6_554 .
O2 Ca1 O1 Ca2 21.05(17) . . 6_554 .
O2 Ca1 O1 Ca2 155.6(3) 5_665 . 6_554 .
O2 Ca1 O1 Ca2 95.19(12) 3_655 . 6_554 .
O3 Ca1 O1 Ca2 30.33(10) . . 6_554 .
O3 Ca1 O1 Ca2 -76.3(2) 5_665 . 6_554 .
O3 Ca1 O1 Ca2 150.98(11) 3_655 . 6_554 .
O1 Ca1 O2 P 16.9(2) 6_554 . . .
```

O1 Ca1 O2 Ca1 122.35(14) 6_554 . . 8_667

O1 Ca1 O2 Ca2 -121.39(15) 6_554 . . 4_665

O1 Ca1 O2 P 91.07(16) 4_664 . . .

O1 Ca1 O2 Ca1 -163.51(12) 4_664 . . 8_667

O1 Ca1 O2 Ca2 -47.25(15) 4_664 . . 4_665

O1 Ca1 O2 P 151.5(3) 2_654 . . .

O1 Ca1 O2 Ca1 -103.1(3) 2_654 . . 8_667

O1 Ca1 O2 Ca2 13.2(4) 2_654 . . 4_665

O2 Ca1 O2 P -144.63(17) 5_665 . . .

O2 Ca1 O2 Ca1 -39.21(12) 5_665 . . 8_667

O2 Ca1 O2 Ca2 77.06(14) 5_665 . . 4_665

O2 Ca1 O2 P -66.21(16) 3_655 . . .

O2 Ca1 O2 Ca1 39.21(12) 3_655 . . 8_667

O2 Ca1 O2 Ca2 155.47(15) 3_655 . . 4_665

O3 Ca1 O2 P 6.52(13) . . . .

O3 Ca1 O2 Ca1 111.94(14) . . . 8_667

O3 Ca1 O2 Ca2 -131.80(16) . . . 4_665

O3 Ca1 O2 P 156.51(17) 5_665 . . .

O3 Ca1 O2 Ca1 -98.07(12) 5_665 . . 8_667

O3 Ca1 O2 Ca2 18.20(12) 5_665 . . 4_665

O3 Ca1 O2 P -95.19(17) 3_655 . . .

O3 Ca1 O2 Ca1 10.23(16) 3_655 . . 8_667

O3 Ca1 O2 Ca2 126.50(14) 3_655 . . 4_665

O1 Ca1 O3 Ca2 -35.19(12) 6_554 . . .

O1 Ca1 O3 P -177.02(15) 6_554 . . .

O1 Ca1 O3 Ca2 86.40(12) 6_554 . . 6_555

O1 Ca1 O3 Ca2 40.39(12) 4_664 . . .

O1 Ca1 O3 P -101.45(14) 4_664 . . .

O1 Ca1 O3 Ca2 161.98(12) 4_664 . . 6_555

O1 Ca1 O3 Ca2 -20.4(2) 2_654 . . .

O1 Ca1 O3 P -162.26(19) 2_654 . . .

O1 Ca1 O3 Ca2 101.2(2) 2_654 . . 6_555

O2 Ca1 O3 Ca2 135.51(15) . . . .

O2 Ca1 O3 P -6.32(14) . . . .

O2 Ca1 O3 Ca2 -102.89(14) . . . 6_555

O2 Ca1 O3 Ca2 169.93(14) 5_665 . . .

O2 Ca1 O3 P 28.10(18) 5_665 . . .

O2 Ca1 O3 Ca2 -68.48(16) 5_665 . . 6_555

O2 Ca1 O3 Ca2 -137.46(14) 3_655 . . .

O2 Ca1 O3 P 80.70(15) 3_655 . . .

O2 Ca1 O3 Ca2 -15.87(12) 3_655 . . 6_555

O3 Ca1 O3 Ca2 104.23(12) 5_665 . . .

O3 Ca1 O3 P -37.61(15) 5_665 . . .

O3 Ca1 O3 Ca2 -134.18(11) 5_665 . . 6_555

O3 Ca1 O3 Ca2 -109.72(12) 3_655 . . .

O3 Ca1 O3 P 108.45(14) 3_655 . . .

O3 Ca1 O3 Ca2 11.88(14) 3_655 . . 6_555

O2 Ca2 O1 Ca1 46.15(14) 4_664 . 6_554 .

O3 Ca2 O1 Ca1 129.07(13) 10_556 . 6_554 .

O3 Ca2 O1 Ca1 128.4(2) 2_554 . 6_554 .

O3 Ca2 O1 Ca1 -36.1(2) 11_557 . 6_554 .

O3 Ca2 O1 Ca1 -36.78(12) . . 6_554 .

O-H Ca2 O1 Ca1 -142.1(2) . . 6_554 .

O1 Ca2 O3 Ca1 30.39(11) 6_554 . . .

O1 Ca2 O3 P 130.7(3) 6_554 . . .

O1 Ca2 O3 Ca2 -74.26(13) 6_554 . . 6_555

O2 Ca2 O3 Ca1 -73.95(12) 4_664 . . .

O2 Ca2 O3 P 26.4(2) 4_664 . . .

O2 Ca2 O3 Ca2 -178.60(14) 4_664 . . 6_555

O3 Ca2 O3 Ca1 8.3(2) 10_556 . . .

O3 Ca2 O3 P 108.6(3) 10_556 . . .

O3 Ca2 O3 Ca2 -96.4(2) 10_556 . . 6_555

O3 Ca2 O3 Ca1 -138.94(15) 2_554 . . .

O3 Ca2 O3 P -38.6(3) 2_554 . . .

O3 Ca2 O3 Ca2 116.41(16) 2_554 . . 6_555

O3 Ca2 O3 Ca1 -149.29(12) 11_557 . . .

O3 Ca2 O3 P -49.0(2) 11_557 . . .

O3 Ca2 O3 Ca2 106.06(14) 11_557 . . 6_555

O-H Ca2 O3 Ca1 131.0(2) . . . .

O-H Ca2 O3 P -128.6(3) . . . .

O-H Ca2 O3 Ca2 26.4(2) . . . 6_555

O1 Ca2 O-H Ca2 168.7(2) 6_554 . . 3_555

O1 Ca2 O-H Ca2 15.8(3) 6_554 . . 5_555

O1 Ca2 O-H H -87.78 6_554 . . .

O2 Ca2 O-H Ca2 -28.0(5) 4_664 . . 3_555

O2 Ca2 O-H Ca2 179.1(2) 4_664 . . 5_555

O2 Ca2 O-H H 75.54 4_664 . . .

O3 Ca2 O-H Ca2 -118.8(2) 10_556 . . 3_555

O3 Ca2 O-H Ca2 88.3(2) 10_556 . . 5_555

O3 Ca2 O-H H -15.28 10_556 . . .

O3 Ca2 O-H Ca2 -42.3(2) 2_554 . . 3_555

O3 Ca2 O-H Ca2 164.8(3) 2_554 . . 5_555

O3 Ca2 O-H H 61.28 2_554 . . .

O3 Ca2 O-H Ca2 17.5(2) 11_557 . . 3_555

O3 Ca2 O-H Ca2 -135.4(2) 11_557 . . 5_555

O3 Ca2 O-H H 121.08 11_557 . . .

O3 Ca2 O-H Ca2 92.6(2) . . . 3_555

O3 Ca2 O-H Ca2 -60.3(3) . . . 5_555

O3 Ca2 O-H H -163.85 . . . .

O2 P O1 Ca1 68.0(2) . . . 7_667

O2 P O1 Ca1 -68.0(2) . . . 2_555

O2 P O1 Ca2 180.00(15) . . . 2_555

O3 P O1 Ca1 -53.0(2) . . . 7_667

O3 P O1 Ca1 171.05(18) . . . 2_555

O3 P O1 Ca2 59.02(16) . . . 2_555

O3 P O1 Ca1 -171.05(18) 10_557 . . 7_667

O3 P O1 Ca1 53.0(2) 10_557 . . 2_555

O3 P O1 Ca2 -59.02(16) 10_557 . . 2_555

O1 P O2 Ca1 -132.92(16) . . . .

O1 P O2 Ca1 132.92(16) . . . 8_667

O1 P O2 Ca2 0.0(2) . . . 4_665

O3 P O2 Ca1 -10.51(19) . . . .

O3 P O2 Ca1 -104.66(16) . . . 8_667

O3 P O2 Ca2 122.42(18) . . . 4_665

O3 P O2 Ca1 104.66(16) 10_557 . . .

O3 P O2 Ca1 10.51(19) 10_557 . . 8_667

O3 P O2 Ca2 -122.42(18) 10_557 . . 4_665

O1 P O3 Ca1 131.57(15) . . . .

O1 P O3 Ca2 28.1(3) . . . .

O1 P O3 Ca2 -130.20(15) . . . 6_555

O2 P O3 Ca1 8.77(16) . . . .

O2 P O3 Ca2 -94.7(3) . . . .

O2 P O3 Ca2 107.00(16) . . . 6_555

O3 P O3 Ca1 -107.70(14) 10_557 . . .

```
O3 P O3 Ca2 148.8(2) 10_557 . . .
O3 P O3 Ca2 -9.47(16) 10_557 . . 6_555

_diffrn_ambient_temperature     ?
_diffrn_radiation_wavelength     1.540560
_diffrn_radiation_type          'Cu K\a~1~'
_diffrn_measurement_device_type ?

# POWDER PROFILE

_pd_meas_2theta_range_min      6.000
_pd_meas_2theta_range_max    120.000
_pd_proc_number_of_points       5701

_pd_proc_ls_prof_R_factor           0.02218
_pd_proc_ls_prof_wR_factor          0.03589
_pd_proc_ls_prof_wR_expected        0.04735

_refine_ls_number_reflns      288
_refine_ls_number_parameters  57
_refine_ls_number_restraints  0
_refine_ls_hydrogen_treatment  constr
_refine_ls_R_I_factor               0.06800
_refine_ls_goodness_of_fit_all      0.75795
_refine_ls_restrained_S_all         0.75795

loop_
     _pd_meas_2theta_scan
     _pd_meas_counts_total
     _pd_proc_2theta_corrected
     _pd_calc_intensity_total
     _pd_proc_intensity_bkg_calc

        6.000000     3385.330078       6.012100        0.000000     3343.560547
        6.020000     3338.330078       6.032100        0.000000     3333.390137
        6.040000     3363.830078       6.052100        0.000000     3323.251953
        6.060000     3279.500000       6.072100        0.000000     3313.145020
        6.080000     3325.500000       6.092100        0.000000     3303.070801
```

6.100000	3305.170166	6.112100	0.000000	3293.030762
6.120000	3286.500000	6.132100	0.000000	3283.019531
6.140000	3240.000000	6.152100	0.000000	3273.043945
6.160000	3274.500000	6.172100	0.000000	3263.101074
6.180000	3247.330078	6.192100	0.000000	3253.190918
6.200000	3245.500000	6.212100	0.000000	3243.311523
6.220000	3242.330078	6.232100	0.000000	3233.467285
6.240000	3232.500000	6.252100	0.000000	3223.655762
6.260000	3232.000000	6.272100	0.000000	3213.875488
6.280000	3202.500000	6.292100	0.000000	3204.130371
6.300000	3221.670166	6.312101	0.000000	3194.418457
6.320000	3176.500000	6.332100	0.000000	3184.739746
6.340000	3166.170166	6.352101	0.000000	3175.093262
6.360000	3180.330078	6.372101	0.000000	3165.482422
6.380000	3129.500000	6.392101	0.000000	3155.904297
6.400000	3149.500000	6.412101	0.000000	3146.358398
6.420000	3154.170166	6.432101	0.000000	3136.847656
6.440000	3116.170166	6.452101	0.000000	3127.370605
6.460000	3147.500000	6.472101	0.000000	3117.927246
6.480000	3119.170166	6.492101	0.000000	3108.517090
6.500000	3110.330078	6.512101	0.000000	3099.140625
6.520000	3095.170166	6.532101	0.000000	3089.800293
6.540000	3084.500000	6.552102	0.000000	3080.492188
6.560000	3062.000000	6.572101	0.000000	3071.217285
6.580000	3066.170166	6.592102	0.000000	3061.977051
6.600000	3054.670166	6.612101	0.000000	3052.771484
6.620000	3074.000000	6.632102	0.000000	3043.600586
6.640000	3046.500000	6.652101	0.000000	3034.462891
6.660000	3036.330078	6.672101	0.000000	3025.356445
6.680000	2978.170166	6.692101	0.000000	3016.285645
6.700000	3005.500000	6.712101	0.000000	3007.250977
6.720000	3002.000000	6.732102	0.000000	2998.247559
6.740000	2997.670166	6.752101	0.000000	2989.279297
6.760000	2942.500000	6.772102	0.000000	2980.345215
6.780000	2984.670166	6.792101	0.000000	2971.445801
6.800000	2993.330078	6.812102	0.000000	2962.579102
6.820000	2946.500000	6.832102	0.000000	2953.747559
6.840000	2922.000000	6.852102	0.000000	2944.948730

6.860000	2925.830078	6.872102	0.000000	2936.183594
6.880000	2880.330078	6.892102	0.000000	2927.454102
6.900000	2926.170166	6.912102	0.000000	2918.758789
6.920000	2882.000000	6.932102	0.000000	2910.097168
6.940000	2918.330078	6.952103	0.000000	2901.467285
6.960000	2904.330078	6.972102	0.000000	2892.872070
6.980000	2916.830078	6.992103	0.000000	2884.311523
7.000000	2884.330078	7.012102	0.000000	2875.786133
7.020000	2872.830078	7.032103	0.000000	2867.291016
7.040000	2835.500000	7.052102	0.000000	2858.832520
7.060000	2837.170166	7.072103	0.000000	2850.406250
7.080000	2814.670166	7.092103	0.000000	2842.013184
7.100000	2839.000000	7.112103	0.000000	2833.654785
7.120000	2814.000000	7.132102	0.000000	2825.330078
7.140000	2798.330078	7.152102	0.000000	2817.038574
7.160000	2812.670166	7.172102	0.000000	2808.778809
7.180000	2769.500000	7.192102	0.000000	2800.553711
7.200000	2777.670166	7.212102	0.000000	2792.363281
7.220000	2794.000000	7.232102	0.000000	2784.205078
7.240000	2745.000000	7.252102	0.000000	2776.080566
7.260000	2779.670166	7.272103	0.000000	2767.987793
7.280000	2773.000000	7.292102	0.000000	2759.929199
7.300000	2765.000000	7.312103	0.000000	2751.901855
7.320000	2683.000000	7.332103	0.000000	2743.910156
7.340000	2706.330078	7.352103	0.000000	2735.950195
7.360000	2702.830078	7.372103	0.000000	2728.023926
7.380000	2692.330078	7.392103	0.000000	2720.128418
7.400000	2744.500000	7.412103	0.000000	2712.266602
7.420000	2735.330078	7.432103	0.000000	2704.437500
7.440000	2731.500000	7.452103	0.000000	2696.642090
7.460000	2681.170166	7.472103	0.000000	2688.876465
7.480000	2687.170166	7.492103	0.000000	2681.145996
7.500000	2651.500000	7.512103	0.000000	2673.447266
7.520000	2656.000000	7.532103	0.000000	2665.780273
7.540000	2659.000000	7.552103	0.000000	2658.145020
7.560000	2645.830078	7.572103	0.000000	2650.541504
7.580000	2646.170166	7.592103	0.000000	2642.971680
7.600000	2655.000000	7.612103	0.000000	2635.432617

7.620000	2654.000000	7.632103	0.000000	2627.924316
7.640000	2625.670166	7.652103	0.000000	2620.450195
7.660000	2603.500000	7.672103	0.000000	2613.006348
7.680000	2624.500000	7.692103	0.000000	2605.594727
7.700000	2588.670166	7.712103	0.000000	2598.212891
7.720000	2583.000000	7.732103	0.000000	2590.863281
7.740000	2601.830078	7.752103	0.000000	2583.545898
7.760000	2553.830078	7.772103	0.000000	2576.259277
7.780000	2581.500000	7.792103	0.000000	2569.002441
7.800000	2526.330078	7.812104	0.000000	2561.778320
7.820000	2561.670166	7.832104	0.000000	2554.584473
7.840000	2519.670166	7.852104	0.000000	2547.421387
7.860000	2546.500000	7.872104	0.000000	2540.287598
7.880000	2510.500000	7.892104	0.000000	2533.187012
7.900000	2530.500000	7.912104	0.000000	2526.115723
7.920000	2535.330078	7.932104	0.000000	2519.074219
7.940000	2524.670166	7.952104	0.000000	2512.062988
7.960000	2508.830078	7.972104	0.000000	2505.083496
7.980000	2535.000000	7.992103	0.000000	2498.133301
8.000000	2504.500000	8.012104	0.000000	2491.211914
8.020000	2468.330078	8.032104	0.000000	2484.321777
8.040000	2455.500000	8.052103	0.000000	2477.461426
8.060000	2460.170166	8.072104	0.000000	2470.630859
8.080000	2463.330078	8.092103	0.000000	2463.829590
8.100000	2459.500000	8.112103	0.000000	2457.057129
8.120001	2434.830078	8.132104	0.000000	2450.314453
8.140000	2452.330078	8.152103	0.000000	2443.601562
8.160001	2457.670166	8.172104	0.000000	2436.916016
8.180000	2447.330078	8.192103	0.000000	2430.261230
8.200000	2431.330078	8.212103	0.000000	2423.634766
8.220000	2415.830078	8.232103	0.000000	2417.038086
8.240000	2386.500000	8.252103	0.000000	2410.469727
8.260000	2390.000000	8.272103	0.000000	2403.928223
8.280000	2411.330078	8.292103	0.000000	2397.416992
8.300000	2372.500000	8.312103	0.000000	2390.933594
8.320001	2383.500000	8.332104	0.000000	2384.477539
8.340000	2392.000000	8.352103	0.000000	2378.050781
8.360001	2353.500000	8.372104	0.000000	2371.651367

8.380000	2346.500000	8.392103	0.000000	2365.280762
8.400000	2333.670166	8.412103	0.000000	2358.937500
8.420000	2364.330078	8.432103	0.000000	2352.621094
8.440000	2343.170166	8.452103	0.000000	2346.333008
8.460000	2307.330078	8.472103	0.000000	2340.072754
8.480000	2329.330078	8.492103	0.000000	2333.839844
8.500000	2343.170166	8.512103	0.000000	2327.632812
8.520000	2327.170166	8.532104	0.000000	2321.453613
8.540000	2297.330078	8.552103	0.000000	2315.301758
8.560000	2334.830078	8.572104	0.000000	2309.175781
8.580000	2309.670166	8.592103	0.000000	2303.076660
8.600000	2313.330078	8.612103	0.000000	2297.005859
8.620001	2245.170166	8.632104	0.000000	2290.958984
8.640000	2271.000000	8.652103	0.000000	2284.940430
8.660001	2287.330078	8.672104	0.000000	2278.947754
8.680000	2246.330078	8.692103	0.000000	2272.980957
8.700000	2257.000000	8.712103	0.000000	2267.041504
8.720000	2262.500000	8.732102	0.000000	2261.126465
8.740000	2206.830078	8.752103	0.000000	2255.236816
8.760000	2241.330078	8.772103	0.000000	2249.373047
8.780000	2240.330078	8.792102	0.000000	2243.536133
8.800000	2220.670166	8.812103	0.000000	2237.722168
8.820001	2236.500000	8.832104	0.000000	2231.936523
8.840000	2260.500000	8.852103	0.000000	2226.174805
8.860001	2256.000000	8.872103	0.000000	2220.437988
8.880000	2200.170166	8.892103	0.000000	2214.725586
8.900000	2179.670166	8.912103	0.000000	2209.039062
8.920000	2195.170166	8.932102	0.000000	2203.376953
8.940000	2194.000000	8.952103	0.000000	2197.739746
8.960000	2181.670166	8.972103	0.000000	2192.125977
8.980000	2200.170166	8.992102	0.000000	2186.538574
9.000000	2179.330078	9.012102	0.000000	2180.973145
9.020000	2158.330078	9.032104	0.000000	2175.433594
9.040000	2152.000000	9.052103	0.000000	2169.916504
9.060000	2147.330078	9.072103	0.000000	2164.423340
9.080000	2158.170166	9.092102	0.000000	2158.955078
9.100000	2139.330078	9.112103	0.000000	2153.509766
9.120001	2160.330078	9.132103	0.000000	2148.088379

9.140000	2151.830078	9.152102	0.000000	2142.690918
9.160001	2123.670166	9.172104	0.086861	2137.315918
9.180000	2101.830078	9.192102	0.089482	2131.965332
9.200000	2138.500000	9.212102	0.092215	2126.636719
9.220000	2135.830078	9.232102	0.095067	2121.330566
9.240000	2090.000000	9.252103	0.098045	2116.047852
9.260000	2078.670166	9.272102	0.101155	2110.788086
9.280000	2102.500000	9.292102	0.104405	2105.551270
9.300000	2089.170166	9.312102	0.107805	2100.335938
9.320001	2092.330078	9.332103	0.111361	2095.143555
9.340000	2073.500000	9.352102	0.115085	2089.973633
9.360001	2086.170166	9.372103	0.118986	2084.824219
9.380000	2115.330078	9.392102	0.123076	2079.698730
9.400000	2100.670166	9.412102	0.127365	2074.594727
9.420000	2088.330078	9.432102	0.131868	2069.512695
9.440000	2083.830078	9.452101	0.136597	2064.452148
9.460000	2048.330078	9.472102	0.141569	2059.412109
9.480000	2040.330078	9.492102	0.146798	2054.394531
9.500000	2044.330078	9.512101	0.152303	2049.398438
9.520000	2036.169922	9.532102	0.158102	2044.423706
9.540000	2016.169922	9.552102	0.164216	2039.469116
9.560000	2008.000000	9.572102	0.170667	2034.535767
9.580000	2027.000000	9.592101	0.177480	2029.623901
9.600000	2039.169922	9.612103	0.184682	2024.732788
9.620001	2029.169922	9.632102	0.192301	2019.861694
9.640000	1999.830078	9.652102	0.200369	2015.011597
9.660001	1978.500000	9.672102	0.208920	2010.182007
9.680000	2004.500000	9.692102	0.217993	2005.372192
9.700000	1985.500000	9.712101	0.227629	2000.583862
9.720000	1980.669922	9.732101	0.237874	1995.814819
9.740000	1965.330078	9.752102	0.248779	1991.065796
9.760000	1979.000000	9.772101	0.260399	1986.336548
9.780000	2007.169922	9.792101	0.272796	1981.627563
9.800000	1950.830078	9.812101	0.286039	1976.936890
9.820001	1956.330078	9.832102	0.300202	1972.267212
9.840000	1996.000000	9.852101	0.315370	1967.616577
9.860001	1975.669922	9.872102	0.331637	1962.984497
9.880000	1980.500000	9.892100	0.349106	1958.373657

9.900000	1943.500000	9.912101	0.367896	1953.779175
9.920000	1945.169922	9.932101	0.388139	1949.205933
9.940000	1945.500000	9.952100	0.409979	1944.650757
9.960000	1940.169922	9.972101	0.433587	1940.114136
9.980000	1951.000000	9.992101	0.459148	1935.596558
10.000000	1969.500000	10.012100	0.486876	1931.098022
10.020000	1921.830078	10.032101	0.517014	1926.617554
10.040000	1922.000000	10.052101	0.549839	1922.155151
10.060000	1897.330078	10.072101	0.585665	1917.710815
10.080000	1939.830078	10.092100	0.624857	1913.285522
10.100000	1907.169922	10.112101	0.667833	1908.878296
10.120001	1923.669922	10.132101	0.715077	1904.488159
10.140000	1915.669922	10.152101	0.767147	1900.116333
10.160001	1888.830078	10.172101	0.824702	1895.762573
10.180000	1887.000000	10.192101	0.888503	1891.426636
10.200000	1896.669922	10.212100	0.959452	1887.107788
10.220000	1858.830078	10.232100	1.038614	1882.807007
10.240000	1899.500000	10.252099	1.127256	1878.522583
10.260000	1884.830078	10.272100	1.226897	1874.256226
10.280000	1894.330078	10.292099	1.339331	1870.007202
10.300000	1856.830078	10.312099	1.466782	1865.774292
10.320001	1858.330078	10.332101	1.611924	1861.558716
10.340000	1870.669922	10.352099	1.778011	1857.360229
10.360001	1841.000000	10.372100	1.969143	1853.178833
10.380000	1844.169922	10.392099	2.190357	1849.013550
10.400000	1864.830078	10.412099	2.448035	1844.864868
10.420000	1840.500000	10.432099	2.750294	1840.733032
10.440000	1839.169922	10.452099	3.107514	1836.617798
10.460000	1835.830078	10.472098	3.533242	1832.518921
10.480000	1840.500000	10.492099	4.045335	1828.434692
10.500000	1820.000000	10.512099	4.667602	1824.368042
10.520000	1864.000000	10.532099	5.432357	1820.317505
10.540000	1853.000000	10.552099	6.384451	1816.283081
10.560000	1794.169922	10.572100	7.587451	1812.262817
10.580000	1815.830078	10.592098	9.135735	1808.259888
10.600000	1822.000000	10.612099	11.183376	1804.272095
10.620001	1828.169922	10.632100	14.020049	1800.299438
10.640000	1833.169922	10.652099	18.275139	1796.342896

10.660001	1876.830078	10.672099	25.387272	1792.401489
10.680000	1876.000000	10.692098	38.399071	1788.475952
10.700000	1913.500000	10.712098	62.729671	1784.565063
10.720000	1942.169922	10.732099	105.732109	1780.668335
10.740000	1961.330078	10.752097	173.817200	1776.788452
10.760000	2066.330078	10.772099	268.909424	1772.922241
10.780000	2141.000000	10.792098	392.195862	1769.071899
10.800000	2295.170166	10.812098	559.459351	1765.235229
10.820001	2492.170166	10.832098	745.310791	1761.413696
10.840000	2484.000000	10.852098	683.544617	1757.606567
10.860001	2239.330078	10.872098	362.604675	1753.814575
10.880000	1973.169922	10.892097	125.177040	1750.036743
10.900000	1836.830078	10.912096	29.872890	1746.273071
10.920000	1803.000000	10.932097	10.565691	1742.523071
10.940000	1790.669922	10.952097	15.483319	1738.788208
10.960000	1760.330078	10.972096	21.261120	1735.067261
10.980000	1740.330078	10.992097	22.344341	1731.359985
11.000000	1732.830078	11.012097	20.105162	1727.666138
11.020000	1717.000000	11.032097	16.786777	1723.986938
11.040000	1710.500000	11.052096	13.654570	1720.321655
11.060000	1733.830078	11.072097	11.100693	1716.669800
11.080000	1702.500000	11.092096	9.114859	1713.030884
11.100000	1695.830078	11.112097	7.578988	1709.406860
11.120001	1689.000000	11.132096	6.379085	1705.795288
11.140000	1681.669922	11.152097	5.428239	1702.197388
11.160001	1700.000000	11.172097	4.664258	1698.612183
11.180000	1728.330078	11.192096	4.042654	1695.040894
11.200000	1722.669922	11.212096	3.531023	1691.482544
11.220000	1712.669922	11.232097	3.105642	1687.937622
11.240000	1693.669922	11.252095	2.748732	1684.405151
11.260000	1702.000000	11.272096	2.446707	1680.885376
11.280000	1694.830078	11.292095	2.189221	1677.379272
11.300000	1670.330078	11.312096	1.968155	1673.885376
11.320001	1665.830078	11.332096	1.777155	1670.404419
11.340000	1671.169922	11.352095	1.611178	1666.935913
11.360001	1693.000000	11.372096	1.466128	1663.479614
11.380000	1688.330078	11.392095	1.338767	1660.036255
11.400000	1705.169922	11.412094	1.226393	1656.605103

11.420000	1667.000000	11.432096	1.126808	1653.186646
11.440000	1655.500000	11.452094	1.038220	1649.780151
11.460000	1663.500000	11.472095	0.959095	1646.385864
11.480000	1645.330078	11.492094	0.888182	1643.003540
11.500000	1642.500000	11.512094	0.824411	1639.633423
11.520000	1641.000000	11.532095	0.766884	1636.274780
11.540000	1635.169922	11.552094	0.714841	1632.928833
11.560000	1621.669922	11.572095	0.667616	1629.594116
11.580000	1635.830078	11.592094	0.624661	1626.271362
11.600000	1628.000000	11.612094	0.585487	1622.960815
11.620001	1608.000000	11.632094	0.549674	1619.661255
11.640000	1613.169922	11.652094	0.516864	1616.373413
11.660001	1602.500000	11.672094	0.486738	1613.097290
11.680000	1619.669922	11.692094	0.459021	1609.832642
11.700000	1621.500000	11.712092	0.433471	1606.579468
11.720000	1597.669922	11.732094	0.409871	1603.336792
11.740000	1602.000000	11.752092	0.388040	1600.106079
11.760000	1592.500000	11.772093	0.367804	1596.886841
11.780000	1592.830078	11.792093	0.349020	1593.677856
11.800000	1570.669922	11.812093	0.331557	1590.480103
11.820001	1566.330078	11.832093	0.315295	1587.293823
11.840000	1557.000000	11.852093	0.300132	1584.118530
11.860001	1560.500000	11.872093	0.285974	1580.953491
11.880000	1603.500000	11.892093	0.272736	1577.799683
11.900000	1586.330078	11.912091	0.260343	1574.656860
11.920000	1576.500000	11.932092	0.248726	1571.524536
11.940000	1566.500000	11.952092	0.237824	1568.402954
11.960000	1564.330078	11.972092	0.227582	1565.291626
11.980000	1567.669922	11.992091	0.217949	1562.191040
12.000000	1565.169922	12.012092	0.208879	1559.100464
12.020000	1565.169922	12.032092	0.200329	1556.020630
12.040000	1595.000000	12.052092	0.192264	1552.951538
12.060000	1578.169922	12.072091	0.184647	1549.892456
12.080000	1524.000000	12.092092	0.177448	1546.843384
12.100000	1581.000000	12.112091	0.170636	1543.804810
12.120001	1554.830078	12.132092	0.164186	1540.776245
12.140000	1533.000000	12.152090	0.158074	1537.757690
12.160001	1522.669922	12.172091	0.152276	1534.748657

12.180000	1540.669922	12.192091	0.146773	1531.750366
12.200000	1562.669922	12.212090	0.141545	1528.762085
12.220000	1533.330078	12.232091	0.136575	1525.783569
12.240000	1531.669922	12.252090	0.131847	1522.814331
12.260000	1521.169922	12.272090	0.127345	1519.854858
12.280000	1485.500000	12.292089	0.123056	1516.905640
12.300000	1514.000000	12.312090	0.118968	1513.965942
12.320001	1499.500000	12.332090	0.115067	1511.035278
12.340000	1516.669922	12.352089	0.111345	1508.114868
12.360001	1502.500000	12.372089	0.107789	1505.203735
12.380000	1487.500000	12.392090	0.104390	1502.301880
12.400000	1495.330078	12.412088	0.101140	1499.409790
12.420000	1497.000000	12.432089	0.098031	1496.526978
12.440000	1510.500000	12.452088	0.095054	1493.653198
12.460000	1493.500000	12.472089	0.092202	1490.788452
12.480000	1475.830078	12.492087	0.089470	1487.933472
12.500000	1472.830078	12.512088	0.086850	1485.087280
12.520000	1499.169922	12.532089	0.000000	1482.250122
12.540000	1482.669922	12.552088	0.000000	1479.422974
12.560000	1486.169922	12.572088	0.000000	1476.603394
12.580000	1476.500000	12.592087	0.000000	1473.793823
12.600000	1456.000000	12.612088	0.000000	1470.992798
12.620001	1454.830078	12.632089	0.000000	1468.200317
12.640000	1473.500000	12.652087	0.000000	1465.417603
12.660001	1448.000000	12.672089	0.000000	1462.643433
12.680000	1466.830078	12.692087	0.000000	1459.877808
12.700000	1444.169922	12.712087	0.000000	1457.120972
12.720000	1453.169922	12.732086	0.000000	1454.372681
12.740000	1458.669922	12.752087	0.000000	1451.632935
12.760000	1447.500000	12.772087	0.000000	1448.901489
12.780000	1440.500000	12.792086	0.000000	1446.179321
12.800000	1417.000000	12.812086	0.000000	1443.465454
12.820001	1434.000000	12.832087	0.000000	1440.759155
12.840000	1423.669922	12.852086	0.000000	1438.062378
12.860001	1438.330078	12.872086	0.000000	1435.373657
12.880000	1418.830078	12.892086	0.000000	1432.693237
12.900000	1413.330078	12.912086	0.000000	1430.021362
12.920000	1428.500000	12.932085	0.000000	1427.357056

12.940000	1413.169922	12.952085	0.000000	1424.701538
12.960000	1407.000000	12.972085	0.000000	1422.054321
12.980000	1405.169922	12.992085	0.000000	1419.414917
13.000000	1433.330078	13.012084	0.000000	1416.783813
13.020000	1402.000000	13.032084	0.000000	1414.160034
13.040000	1401.330078	13.052084	0.000000	1411.545532
13.060000	1384.500000	13.072084	0.000000	1408.938110
13.080000	1395.169922	13.092084	0.000000	1406.339233
13.100000	1379.500000	13.112084	0.000000	1403.747437
13.120001	1398.169922	13.132085	0.000000	1401.164185
13.140000	1398.830078	13.152083	0.000000	1398.588501
13.160001	1369.330078	13.172084	0.000000	1396.020874
13.180000	1377.669922	13.192083	0.000000	1393.461060
13.200000	1385.830078	13.212083	0.000000	1390.909058
13.220000	1381.500000	13.232083	0.000000	1388.364136
13.240000	1375.669922	13.252082	0.000000	1385.827515
13.260000	1372.330078	13.272083	0.000000	1383.298218
13.280000	1396.169922	13.292082	0.000000	1380.776978
13.300000	1398.830078	13.312082	0.000000	1378.262817
13.320001	1375.830078	13.332083	0.000000	1375.755981
13.340000	1357.169922	13.352082	0.000000	1373.256958
13.360001	1345.169922	13.372082	0.000000	1370.765747
13.380000	1361.669922	13.392081	0.000000	1368.281616
13.400000	1379.169922	13.412081	0.000000	1365.805054
13.420000	1361.000000	13.432081	0.000000	1363.335815
13.440000	1391.500000	13.452081	0.000000	1360.873901
13.460000	1355.500000	13.472080	0.000000	1358.419312
13.480000	1354.830078	13.492081	0.000000	1355.971802
13.500000	1354.000000	13.512080	0.000000	1353.532104
13.520000	1353.000000	13.532081	0.000000	1351.098755
13.540000	1354.500000	13.552079	0.000000	1348.673218
13.560000	1340.000000	13.572081	0.000000	1346.254761
13.580000	1407.000000	13.592079	0.000000	1343.843872
13.600000	1386.669922	13.612080	0.000000	1341.439575
13.620001	1371.830078	13.632081	0.000000	1339.041870
13.640000	1342.169922	13.652080	0.000000	1336.652222
13.660001	1350.169922	13.672080	0.000000	1334.269165
13.680000	1334.330078	13.692079	0.000000	1331.892944

13.700000	1313.830078	13.712079	0.000000	1329.523560
13.720000	1322.169922	13.732079	0.000000	1327.161499
13.740000	1332.500000	13.752078	0.000000	1324.806030
13.760000	1324.169922	13.772078	0.000000	1322.458130
13.780000	1350.000000	13.792078	0.000000	1320.116577
13.800000	1316.169922	13.812078	0.000000	1317.781860
13.820001	1342.169922	13.832078	0.000000	1315.453491
13.840000	1299.830078	13.852077	0.000000	1313.132690
13.860001	1309.000000	13.872078	0.000000	1310.818481
13.880000	1297.000000	13.892077	0.000000	1308.510620
13.900000	1294.000000	13.912076	0.000000	1306.210083
13.920000	1305.330078	13.932077	0.000000	1303.915405
13.940000	1277.830078	13.952076	0.000000	1301.627808
13.960000	1290.669922	13.972076	0.000000	1299.347046
13.980000	1299.669922	13.992075	0.000000	1297.072876
14.000000	1295.000000	14.012076	0.000000	1294.804565
14.020000	1297.500000	14.032076	0.000000	1292.543335
14.040000	1313.000000	14.052075	0.000000	1290.288940
14.060000	1282.330078	14.072076	0.000000	1288.040649
14.080000	1278.169922	14.092075	0.000000	1285.799194
14.100000	1282.330078	14.112075	0.000000	1283.563599
14.120001	1261.669922	14.132075	0.000000	1281.335083
14.140000	1267.500000	14.152075	0.000000	1279.112671
14.160001	1262.169922	14.172075	0.000000	1276.896851
14.180000	1267.169922	14.192074	0.000000	1274.687134
14.200000	1289.500000	14.212073	0.000000	1272.484253
14.220000	1257.330078	14.232075	0.000000	1270.286987
14.240000	1245.330078	14.252073	0.000000	1268.096069
14.260000	1257.830078	14.272074	0.000000	1265.911987
14.280000	1262.669922	14.292073	0.000000	1263.733521
14.300000	1253.669922	14.312074	0.000000	1261.561890
14.320001	1258.669922	14.332073	0.000000	1259.396606
14.340000	1238.169922	14.352073	0.000000	1257.237183
14.360001	1253.500000	14.372073	0.000000	1255.083862
14.380000	1241.669922	14.392073	0.000000	1252.936890
14.400000	1264.669922	14.412071	0.000000	1250.796265
14.420000	1235.169922	14.432072	0.000000	1248.661255
14.440000	1246.000000	14.452071	0.000000	1246.532593

14.460000	1239.169922	14.472072	0.000000	1244.409790
14.480000	1223.669922	14.492070	0.000000	1242.293335
14.500000	1234.169922	14.512071	0.000000	1240.182739
14.520000	1229.669922	14.532071	0.000000	1238.078247
14.540000	1230.000000	14.552071	0.000000	1235.979858
14.560000	1219.830078	14.572070	0.000000	1233.887329
14.580000	1238.500000	14.592071	0.000000	1231.800659
14.600000	1222.669922	14.612070	0.000000	1229.720337
14.620001	1201.669922	14.632071	0.000000	1227.645386
14.640000	1215.169922	14.652069	0.000000	1225.577271
14.660001	1235.500000	14.672071	0.000000	1223.514282
14.680000	1213.169922	14.692069	0.000000	1221.457397
14.700000	1230.500000	14.712069	0.000000	1219.406372
14.720000	1221.830078	14.732068	0.000000	1217.360474
14.740000	1207.330078	14.752069	0.000000	1215.321167
14.760000	1218.330078	14.772068	0.000000	1213.287720
14.780000	1214.169922	14.792068	0.000000	1211.259888
14.800000	1212.500000	14.812068	0.000000	1209.237915
14.820001	1189.330078	14.832068	0.000000	1207.221313
14.840000	1198.169922	14.852067	0.000000	1205.211060
14.860001	1192.330078	14.872067	0.000000	1203.206177
14.880000	1183.000000	14.892067	0.000000	1201.206909
14.900000	1206.500000	14.912066	0.000000	1199.213745
14.920000	1206.330078	14.932066	0.000000	1197.226196
14.940000	1194.330078	14.952065	0.000000	1195.243774
14.960000	1207.669922	14.972066	0.000000	1193.267212
14.980000	1181.330078	14.992065	0.000000	1191.296265
15.000000	1177.000000	15.012065	0.000000	1189.331177
15.020000	1176.330078	15.032066	0.000000	1187.371216
15.040000	1160.000000	15.052065	0.000000	1185.417603
15.060000	1188.669922	15.072065	0.000000	1183.468872
15.080000	1179.500000	15.092064	0.000000	1181.526489
15.100000	1198.000000	15.112065	0.000000	1179.588501
15.120001	1171.169922	15.132065	0.000000	1177.656616
15.140000	1146.000000	15.152064	0.000000	1175.730103
15.160001	1169.500000	15.172064	0.000000	1173.809204
15.180000	1162.669922	15.192064	0.000000	1171.893921
15.200000	1157.830078	15.212063	0.000000	1169.984009

15.220000	1164.669922	15.232063	0.000000	1168.079468
15.240000	1163.169922	15.252062	0.000000	1166.180542
15.260000	1106.330078	15.272063	0.141909	1164.286499
15.280000	1139.830078	15.292062	0.145565	1162.398315
15.300000	1144.169922	15.312062	0.149364	1160.515259
15.320001	1135.669922	15.332063	0.153315	1158.637573
15.340000	1135.500000	15.352062	0.157424	1156.765747
15.360001	1142.000000	15.372062	0.161701	1154.898804
15.380000	1158.500000	15.392061	0.166156	1153.037720
15.400000	1146.830078	15.412061	0.170797	1151.181274
15.420000	1129.330078	15.432061	0.175636	1149.330444
15.440000	1134.500000	15.452060	0.180684	1147.484741
15.460000	1139.169922	15.472060	0.185954	1145.644165
15.480000	1154.500000	15.492060	0.191457	1143.809448
15.500000	1115.669922	15.512060	0.197210	1141.979370
15.520000	1157.830078	15.532060	0.203226	1140.155151
15.540000	1144.000000	15.552060	0.209523	1138.335815
15.560000	1128.830078	15.572060	0.216117	1136.521606
15.580000	1099.000000	15.592059	0.223029	1134.712769
15.600000	1158.169922	15.612059	0.230277	1132.909058
15.620001	1143.830078	15.632060	0.237887	1131.110229
15.640000	1134.669922	15.652059	0.245880	1129.317261
15.660001	1140.169922	15.672058	0.254284	1127.529175
15.680000	1113.830078	15.692058	0.263127	1125.745972
15.700000	1115.330078	15.712057	0.272440	1123.967896
15.720000	1139.000000	15.732058	0.282259	1122.195190
15.740000	1123.169922	15.752056	0.292618	1120.427368
15.760000	1110.169922	15.772057	0.303560	1118.664917
15.780000	1114.500000	15.792056	0.315129	1116.907104
15.800000	1108.830078	15.812057	0.327374	1115.154419
15.820001	1107.330078	15.832056	0.340347	1113.406860
15.840000	1100.830078	15.852057	0.354109	1111.664429
15.860001	1114.500000	15.872056	0.368725	1109.926880
15.880000	1096.500000	15.892056	0.384266	1108.194946
15.900000	1091.830078	15.912054	0.400811	1106.467651
15.920000	1093.330078	15.932055	0.418454	1104.744751
15.940000	1088.500000	15.952054	0.437288	1103.027466
15.960000	1096.830078	15.972054	0.457426	1101.315308

15.980000	1107.330078	15.992053	0.478989	1099.607788
16.000000	1069.000000	16.012053	0.502118	1097.905151
16.020000	1114.330078	16.032053	0.526967	1096.207642
16.040001	1100.830078	16.052055	0.553714	1094.514771
16.059999	1090.000000	16.072054	0.582548	1092.827026
16.080000	1100.669922	16.092054	0.613702	1091.144165
16.100000	1091.669922	16.112053	0.647424	1089.466187
16.120001	1105.000000	16.132053	0.684012	1087.792847
16.140001	1084.000000	16.152054	0.723797	1086.124634
16.160000	1102.500000	16.172052	0.767158	1084.461304
16.180000	1095.669922	16.192053	0.814547	1082.802612
16.200001	1088.000000	16.212053	0.866472	1081.148804
16.220001	1061.169922	16.232054	0.923532	1079.499878
16.240002	1088.330078	16.252052	0.986422	1077.855835
16.260000	1065.000000	16.272051	1.055968	1076.216675
16.280001	1043.500000	16.292051	1.133153	1074.582153
16.299999	1062.169922	16.312050	1.219121	1072.952515
16.320000	1062.000000	16.332048	1.315265	1071.327515
16.340000	1049.000000	16.352051	1.423278	1069.706909
16.359999	1082.500000	16.372049	1.545147	1068.091675
16.379999	1058.500000	16.392050	1.683385	1066.480835
16.400000	1058.169922	16.412048	1.841027	1064.874878
16.420000	1065.000000	16.432049	2.021911	1063.273560
16.440001	1074.330078	16.452049	2.230815	1061.676636
16.459999	1049.669922	16.472046	2.473774	1060.085083
16.480000	1059.330078	16.492048	2.758741	1058.497681
16.500000	1053.330078	16.512049	3.095824	1056.914917
16.520000	1047.169922	16.532049	3.498547	1055.337036
16.540001	1058.830078	16.552048	3.985039	1053.763794
16.559999	1051.000000	16.572046	4.580426	1052.195190
16.580000	1034.169922	16.592047	5.321004	1050.630981
16.600000	1062.669922	16.612047	6.263678	1049.071655
16.620001	1061.830078	16.632048	7.510551	1047.516724
16.640001	1055.169922	16.652048	9.268233	1045.966431
16.660000	1033.169922	16.672047	11.966773	1044.421021
16.680000	1055.330078	16.692045	16.428726	1042.880005
16.700001	1065.669922	16.712046	23.963440	1041.343140
16.720001	1074.000000	16.732046	36.145695	1039.811157

16.740002	1069.669922	16.752047	54.245564	1038.283569
16.760000	1105.330078	16.772043	79.134583	1036.760864
16.780001	1149.330078	16.792046	113.638145	1035.242310
16.799999	1208.330078	16.812044	166.768478	1033.728882
16.820000	1236.830078	16.832043	233.463043	1032.219116
16.840000	1266.500000	16.852043	222.020844	1030.714478
16.859999	1180.169922	16.872042	128.704681	1029.213989
16.879999	1112.000000	16.892042	59.809082	1027.717896
16.900000	1072.330078	16.912041	27.775965	1026.226685
16.920000	1061.000000	16.932043	16.426149	1024.739380
16.940001	1082.669922	16.952044	13.565630	1023.256714
16.959999	1093.330078	16.972040	12.803208	1021.778687
16.980000	1065.000000	16.992041	11.835653	1020.304810
17.000000	1034.830078	17.012041	10.411702	1018.835327
17.020000	1055.330078	17.032042	8.864380	1017.369995
17.040001	1018.830017	17.052040	7.462808	1015.909790
17.059999	1016.169983	17.072039	6.300659	1014.453735
17.080000	1006.669983	17.092041	5.367720	1013.001587
17.100000	982.333008	17.112040	4.621444	1011.554077
17.120001	1027.830078	17.132040	4.018909	1010.110718
17.140001	1009.669983	17.152040	3.526461	1008.671997
17.160000	1025.830078	17.172039	3.119107	1007.237427
17.180000	1011.169983	17.192038	2.778358	1005.807495
17.200001	999.833008	17.212038	2.490455	1004.381226
17.220001	983.000000	17.232040	2.245032	1002.959595
17.240002	995.166992	17.252039	2.034196	1001.542114
17.260000	988.500000	17.272038	1.851715	1000.128784
17.280001	986.500000	17.292038	1.769957	998.720093
17.299999	996.166992	17.312037	1.632719	997.315552
17.320000	995.333008	17.332035	1.512097	995.915649
17.340000	1019.330017	17.352036	1.405576	994.519165
17.359999	998.166992	17.372034	1.311125	993.127075
17.379999	955.833008	17.392035	1.227042	991.739624
17.400000	980.833008	17.412035	1.151939	990.355591
17.420000	966.333008	17.432035	1.084651	988.976196
17.440001	1001.500000	17.452036	1.024199	987.601196
17.459999	978.833008	17.472033	0.969768	986.230347
17.480000	1009.169983	17.492033	0.920640	984.863403

17.500000	977.500000	17.512033	0.876228	983.500610
17.520000	987.166992	17.532034	0.836024	982.141724
17.540001	990.666992	17.552034	0.799590	980.787476
17.559999	994.833008	17.572033	0.766554	979.437012
17.580000	977.500000	17.592033	0.736583	978.090698
17.600000	977.500000	17.612032	0.709402	976.748413
17.620001	949.000000	17.632032	0.684762	975.410400
17.640001	974.333008	17.652033	0.662456	974.076172
17.660000	986.833008	17.672029	0.642302	972.746460
17.680000	997.166992	17.692032	0.624133	971.420044
17.700001	994.833008	17.712032	0.607820	970.098022
17.720001	963.000000	17.732031	0.593242	968.780396
17.740002	968.833008	17.752031	0.580297	967.466309
17.760000	966.500000	17.772030	0.568900	966.156616
17.780001	960.833008	17.792030	0.558977	964.850586
17.799999	955.333008	17.812027	0.550472	963.548950
17.820000	937.166992	17.832029	0.543334	962.250610
17.840000	967.666992	17.852030	0.537529	960.956543
17.859999	967.333008	17.872026	0.533033	959.666504
17.879999	950.333008	17.892027	0.529829	958.380493
17.900000	952.000000	17.912027	0.527913	957.098267
17.920000	964.333008	17.932026	0.527293	955.819824
17.940001	949.000000	17.952026	0.527985	954.545532
17.959999	964.833008	17.972025	0.530017	953.274902
17.980000	945.000000	17.992027	0.533431	952.008301
18.000000	963.333008	18.012026	0.538278	950.745850
18.020000	953.500000	18.032026	0.544626	949.486572
18.040001	944.000000	18.052027	0.552558	948.231689
18.059999	941.333008	18.072023	0.562171	946.980347
18.080000	948.500000	18.092024	0.573587	945.733154
18.100000	930.000000	18.112024	0.586947	944.489502
18.120001	938.500000	18.132025	0.602417	943.249756
18.140001	960.166992	18.152025	0.620194	942.013550
18.160000	963.666992	18.172024	0.640506	940.781372
18.180000	947.666992	18.192024	0.663631	939.553101
18.200001	926.666992	18.212023	0.689883	938.328247
18.220001	918.166992	18.232023	0.719647	937.107422
18.240002	933.333008	18.252024	0.753370	935.890137

18.260000	916.833008	18.272020	0.791577	934.677002
18.280001	941.833008	18.292023	0.834925	933.466919
18.299999	931.166992	18.312021	0.884158	932.261108
18.320000	954.333008	18.332020	0.940208	931.058838
18.340000	920.500000	18.352020	1.004204	929.859985
18.359999	914.666992	18.372019	1.077504	928.665161
18.379999	911.666992	18.392017	1.161807	927.473877
18.400000	943.000000	18.412018	1.259225	926.286255
18.420000	920.166992	18.432018	1.233781	925.102051
18.440001	918.500000	18.452021	1.369449	923.921631
18.459999	920.666992	18.472017	1.528330	922.744995
18.480000	928.166992	18.492018	1.716066	921.571655
18.500000	933.000000	18.512018	1.939957	920.401733
18.520000	907.166992	18.532017	2.209770	919.235840
18.540001	903.833008	18.552017	2.538908	918.073486
18.559999	934.833008	18.572016	2.946043	916.914673
18.580000	928.500000	18.592016	3.458987	915.759155
18.600000	911.166992	18.612017	4.122812	914.607178
18.620001	904.500000	18.632017	5.020599	913.458740
18.640001	913.333008	18.652016	6.319603	912.313965
18.660000	912.166992	18.672014	8.355143	911.172974
18.680000	920.000000	18.692015	11.730940	910.035034
18.700001	916.166992	18.712015	17.345329	908.900757
18.720001	936.666992	18.732014	26.230913	907.769775
18.740002	953.666992	18.752016	39.328159	906.641968
18.760000	949.666992	18.772015	57.879631	905.518066
18.780001	981.333008	18.792013	85.498558	904.397461
18.799999	1009.169983	18.812012	128.128143	903.280518
18.820000	1045.000000	18.832012	163.872177	902.166626
18.840000	1011.830017	18.852011	129.681519	901.056274
18.859999	970.833008	18.872009	69.172890	899.949341
18.879999	933.333008	18.892012	32.749462	898.845459
18.900000	928.666992	18.912010	16.832077	897.745605
18.920000	922.833008	18.932011	11.149351	896.648438
18.940001	934.833008	18.952011	9.312527	895.554932
18.959999	907.833008	18.972008	8.349998	894.464844
18.980000	907.666992	18.992008	7.343259	893.377563
19.000000	899.833008	19.012009	6.254005	892.293945

19.020000	888.333008	19.032007	5.232054	891.213989
19.040001	883.000000	19.052010	4.366751	890.136597
19.059999	891.166992	19.072008	3.670350	889.062744
19.080000	886.500000	19.092009	3.117766	887.992188
19.100000	881.833008	19.112007	2.677489	886.924683
19.120001	905.166992	19.132008	2.322586	885.860718
19.140001	873.000000	19.152008	2.032846	884.799438
19.160000	874.666992	19.172005	1.793465	883.742065
19.180000	884.833008	19.192007	1.593425	882.687500
19.200001	876.333008	19.212008	1.424682	881.635864
19.220001	888.500000	19.232006	1.281079	880.588013
19.240002	889.833008	19.252007	1.157872	879.542969
19.260000	886.833008	19.272005	1.051419	878.500977
19.280001	876.666992	19.292004	0.958828	877.462158
19.299999	876.666992	19.312002	0.877807	876.426636
19.320000	883.333008	19.332003	0.806513	875.394043
19.340000	885.833008	19.352003	0.743464	874.364624
19.359999	882.833008	19.372002	0.687450	873.338257
19.379999	886.500000	19.392002	0.637459	872.314819
19.400000	890.000000	19.412001	0.592672	871.294434
19.420000	872.000000	19.432001	0.552386	870.277344
19.440001	859.000000	19.452002	0.516027	869.262939
19.459999	859.166992	19.471998	0.483108	868.251953
19.480000	867.666992	19.491999	0.453200	867.243896
19.500000	870.166992	19.512001	0.425953	866.238525
19.520000	871.333008	19.532000	0.401068	865.236450
19.540001	866.500000	19.552000	0.378275	864.237183
19.559999	858.833008	19.571999	0.357351	863.240967
19.580000	865.333008	19.591997	0.338097	862.247681
19.600000	845.333008	19.611998	0.320340	861.257080
19.620001	860.833008	19.631998	0.303930	860.269531
19.640001	866.666992	19.651999	0.288735	859.285034
19.660000	846.166992	19.671997	0.274640	858.303223
19.680000	850.500000	19.691998	0.261540	857.324341
19.700001	868.000000	19.711996	0.249347	856.348389
19.720001	848.833008	19.731997	0.237975	855.375244
19.740002	869.166992	19.751997	0.227356	854.405029
19.760000	852.000000	19.771994	0.217426	853.437866

19.780001	856.833008	19.791994	0.208124	852.473022
19.799999	852.500000	19.811995	0.199399	851.511230
19.820000	863.333008	19.831993	0.191206	850.552246
19.840000	843.333008	19.851994	0.183502	849.595825
19.859999	831.500000	19.871992	0.176250	848.642578
19.879999	841.833008	19.891991	0.169414	847.692017
19.900000	856.000000	19.911991	0.162964	846.743896
19.920000	852.166992	19.931992	0.156871	845.798340
19.940001	848.666992	19.951992	0.151109	844.855957
19.959999	836.000000	19.971991	0.145656	843.916260
19.980000	827.833008	19.991991	0.140490	842.979004
20.000000	841.166992	20.011990	0.135591	842.044556
20.020000	841.666992	20.031990	0.130940	841.112671
20.040001	850.666992	20.051991	0.126522	840.183594
20.059999	833.333008	20.071987	0.122322	839.257202
20.080000	852.500000	20.091988	0.118325	838.333130
20.100000	846.000000	20.111990	0.227428	837.411621
20.120001	851.666992	20.131989	0.226887	836.493164
20.140001	854.500000	20.151989	0.226638	835.576904
20.160000	865.333008	20.171988	0.226677	834.663696
20.180000	838.500000	20.191986	0.227001	833.752563
20.200001	852.833008	20.211987	0.227609	832.844116
20.220001	860.666992	20.231987	0.228501	831.938232
20.240002	851.500000	20.251986	0.229678	831.034912
20.260000	838.000000	20.271986	0.231142	830.134033
20.280001	839.166992	20.291986	0.232897	829.235474
20.299999	836.500000	20.311983	0.234946	828.339722
20.320000	841.333008	20.331984	0.237296	827.446411
20.340000	815.833008	20.351984	0.239953	826.555420
20.359999	842.333008	20.371981	0.242925	825.667114
20.379999	830.500000	20.391981	0.246222	824.781250
20.400000	850.833008	20.411983	0.175889	823.897339
20.420000	815.000000	20.431982	0.181775	823.016113
20.440001	846.500000	20.451982	0.187949	822.137329
20.459999	842.166992	20.471981	0.194431	821.260864
20.480000	811.666992	20.491980	0.201241	820.386841
20.500000	822.666992	20.511980	0.208402	819.515015
20.520000	827.333008	20.531981	0.215938	818.645508

20.540001	822.833008	20.551979	0.223873	817.778564
20.559999	817.666992	20.571980	0.232237	816.913696
20.580000	820.500000	20.591980	0.241061	816.051392
20.600000	842.166992	20.611979	0.250377	815.191162
20.620001	831.833008	20.631979	0.260224	814.333130
20.640001	809.166992	20.651979	0.270640	813.477661
20.660000	821.833008	20.671976	0.281668	812.624512
20.680000	806.500000	20.691977	0.293359	811.773438
20.700001	805.833008	20.711977	0.305765	810.924316
20.720001	834.166992	20.731977	0.318944	810.077881
20.740002	817.500000	20.751978	0.332961	809.233398
20.760000	818.000000	20.771976	0.347883	808.390991
20.780001	813.666992	20.791975	0.363793	807.550781
20.799999	827.833008	20.811974	0.380775	806.713013
20.820000	826.666992	20.831974	0.398930	805.877197
20.840000	825.666992	20.851973	0.418361	805.043579
20.859999	816.500000	20.871973	0.439192	804.212036
20.879999	826.666992	20.891972	0.461553	803.382690
20.900000	818.833008	20.911972	0.485601	802.555298
20.920000	805.500000	20.931973	0.511502	801.729858
20.940001	799.833008	20.951971	0.539445	800.906982
20.959999	796.500000	20.971970	0.569649	800.085693
20.980000	785.500000	20.991970	0.602362	799.266724
21.000000	815.333008	21.011969	0.637857	798.449951
21.020000	819.166992	21.031971	0.676464	797.634644
21.040001	798.333008	21.051971	0.718535	796.821777
21.059999	798.666992	21.071968	0.764484	796.010620
21.080000	793.833008	21.091969	0.814812	795.201782
21.100000	814.333008	21.111969	0.870071	794.394775
21.120001	802.666992	21.131968	0.930903	793.589478
21.140001	802.000000	21.151968	0.998083	792.786499
21.160000	792.000000	21.171967	1.072488	791.985229
21.180000	798.666992	21.191967	1.155185	791.185913
21.200001	794.333008	21.211967	1.330397	790.388428
21.220001	793.500000	21.231966	1.435995	789.593262
21.240002	803.166992	21.251966	1.554506	788.799438
21.260000	798.500000	21.271965	1.688068	788.007690
21.280001	777.833008	21.291964	1.839319	787.217773

21.299999	782.000000	21.311962	2.011477	786.429810
21.320000	791.000000	21.331964	2.208556	785.643555
21.340000	794.333008	21.351963	2.435433	784.859009
21.359999	793.666992	21.371962	2.698370	784.076660
21.379999	788.166992	21.391962	3.005313	783.295654
21.400000	800.666992	21.411961	3.366377	782.516846
21.420000	799.166992	21.431961	3.794874	781.739258
21.440001	780.666992	21.451962	4.308245	780.963989
21.459999	805.000000	21.471958	4.929778	780.190186
21.480000	777.666992	21.491961	5.691747	779.417969
21.500000	791.333008	21.511959	6.638535	778.647827
21.520000	806.833008	21.531960	7.835588	777.878906
21.540001	786.166992	21.551960	9.384808	777.112183
21.559999	794.333008	21.571957	11.462365	776.346802
21.580000	778.666992	21.591957	14.405544	775.583374
21.600000	782.833008	21.611958	18.875473	774.821289
21.620001	812.500000	21.631956	26.099689	774.061035
21.640001	808.333008	21.651958	38.083679	773.302368
21.660000	822.166992	21.671957	57.565258	772.545410
21.680000	837.666992	21.691956	87.791458	771.790039
21.700001	909.500000	21.711956	133.091873	771.036011
21.720001	978.166992	21.731956	202.316086	770.283691
21.740002	1082.000000	21.751955	311.365906	769.532959
21.760000	1193.330078	21.771954	437.408356	768.783813
21.780001	1205.500000	21.791954	418.799652	768.036255
21.799999	1052.669922	21.811953	255.072678	767.290283
21.820000	939.166992	21.831953	127.864426	766.545532
21.840000	868.833008	21.851952	65.242249	765.802490
21.859999	814.000000	21.871950	39.248135	765.061157
21.879999	806.166992	21.891951	29.009842	764.320923
21.900000	789.000000	21.911949	24.063198	763.582275
21.920000	784.000000	21.931950	20.394588	762.845093
21.940001	780.666992	21.951952	17.060553	762.109375
21.959999	765.666992	21.971949	14.105351	761.375122
21.980000	758.666992	21.991949	11.653775	760.642090
22.000000	771.666992	22.011948	9.709318	759.910767
22.020000	755.166992	22.031948	8.192953	759.180542
22.040001	767.000000	22.051949	7.009371	758.452026

22.059999	761.666992	22.071945	6.077176	757.724854
22.080000	767.666992	22.091946	5.334517	756.998779
22.100000	781.666992	22.111948	4.736926	756.274048
22.120001	764.666992	22.131947	4.252277	755.550781
22.140001	778.333008	22.151947	3.856822	754.828613
22.160000	776.500000	22.171944	3.533204	754.108276
22.180000	761.666992	22.191944	3.268231	753.388916
22.200001	770.666992	22.211945	3.052112	752.670654
22.220001	781.500000	22.231943	2.877401	751.954102
22.240002	780.166992	22.251944	2.738431	751.238403
22.260000	753.333008	22.271944	2.631012	750.524170
22.280001	759.666992	22.291943	2.552098	749.811279
22.299999	768.000000	22.311941	2.499579	749.099487
22.320000	743.333008	22.331940	2.472197	748.388916
22.340000	774.166992	22.351940	2.469457	747.679443
22.359999	745.000000	22.371939	2.491611	746.971436
22.379999	752.666992	22.391937	2.539673	746.264404
22.400000	759.000000	22.411940	2.615516	745.558472
22.420000	747.166992	22.431940	2.721947	744.854004
22.440001	734.166992	22.451939	2.862948	744.150391
22.459999	759.666992	22.471937	3.043976	743.447876
22.480000	729.000000	22.491936	3.272368	742.746826
22.500000	744.166992	22.511936	3.557990	742.046631
22.520000	742.833008	22.531937	3.914055	741.347534
22.540001	771.666992	22.551935	4.358467	740.649658
22.559999	755.000000	22.571936	4.915976	739.952637
22.580000	744.000000	22.591936	5.621128	739.256958
22.600000	734.333008	22.611935	6.523952	738.562256
22.620001	767.000000	22.631935	7.701639	737.868408
22.640001	748.333008	22.651934	9.283430	737.175903
22.660000	750.833008	22.671932	11.509046	736.484253
22.680000	759.833008	22.691933	14.844773	735.793579
22.700001	767.166992	22.711931	20.168480	735.104004
22.720001	788.000000	22.731934	28.975594	734.415283
22.740002	787.500000	22.751934	43.450611	733.727661
22.760000	828.500000	22.771931	66.378479	733.041016
22.780001	847.333008	22.791931	101.455147	732.355225
22.799999	902.333008	22.811928	155.399277	731.670654

22.820000	1008.330017	22.831928	241.108597	730.986816
22.840000	1099.330078	22.851929	355.129700	730.303711
22.859999	1151.000000	22.871925	385.401245	729.621826
22.879999	1035.830078	22.891928	261.267883	728.940552
22.900000	886.333008	22.911928	136.106705	728.260376
22.920000	822.166992	22.931927	68.843086	727.581177
22.940001	792.000000	22.951927	39.360950	726.902710
22.959999	778.333008	22.971924	27.281979	726.225098
22.980000	736.833008	22.991924	21.690886	725.548584
23.000000	744.166992	23.011925	18.027445	724.872559
23.020000	743.666992	23.031925	14.933265	724.197388
23.040001	744.500000	23.051926	12.239754	723.523071
23.059999	720.166992	23.071922	10.006179	722.849731
23.080000	753.166992	23.091923	8.230276	722.177246
23.100000	717.500000	23.111923	6.844785	721.505249
23.120001	720.166992	23.131922	5.763802	720.834106
23.140001	716.666992	23.151922	4.911656	720.163818
23.160000	725.000000	23.171921	4.230911	719.494385
23.180000	731.166992	23.191921	3.679555	718.825439
23.200001	725.666992	23.211922	3.227414	718.157349
23.220001	721.333008	23.231920	2.852457	717.490112
23.240002	730.500000	23.251921	2.538276	716.823364
23.260000	715.333008	23.271919	2.272650	716.157593
23.280001	723.833008	23.291918	2.046180	715.492310
23.299999	727.333008	23.311916	1.851631	714.827881
23.320000	733.500000	23.331915	1.683346	714.163818
23.340000	711.500000	23.351917	1.536831	713.500732
23.359999	708.666992	23.371916	1.408571	712.838135
23.379999	722.666992	23.391914	1.295665	712.176270
23.400000	715.833008	23.411915	1.195769	711.515137
23.420000	715.833008	23.431913	1.106992	710.854492
23.440001	688.666992	23.451914	1.027739	710.194458
23.459999	715.500000	23.471912	0.846558	709.535034
23.480000	718.500000	23.491911	0.920557	708.876221
23.500000	740.333008	23.511913	0.868502	708.218018
23.520000	700.000000	23.531914	0.821874	707.560425
23.540001	710.333008	23.551912	0.780019	706.903442
23.559999	721.000000	23.571911	0.742379	706.246826

23.580000	712.166992	23.591909	0.708477	705.591064
23.600000	724.666992	23.611910	0.677904	704.935425
23.620001	713.500000	23.631910	0.650310	704.280518
23.640001	693.000000	23.651909	0.625393	703.626343
23.660000	703.166992	23.671909	0.602884	702.972534
23.680000	688.666992	23.691908	0.582555	702.319214
23.700001	689.000000	23.711908	0.564202	701.666382
23.720001	694.000000	23.731909	0.547652	701.014038
23.740002	693.833008	23.751907	0.532750	700.362061
23.760000	722.833008	23.771906	0.539323	699.710815
23.780001	687.000000	23.791904	0.527951	699.059814
23.799999	703.333008	23.811905	0.517892	698.409424
23.820000	707.666992	23.831905	0.509057	697.759399
23.840000	685.333008	23.851904	0.501366	697.109985
23.859999	703.166992	23.871902	0.494747	696.460693
23.879999	720.333008	23.891901	0.489139	695.812012
23.900000	716.166992	23.911901	0.484487	695.163574
23.920000	696.333008	23.931902	0.480746	694.515747
23.940001	675.500000	23.951900	0.477874	693.868164
23.959999	695.500000	23.971901	0.475837	693.220947
23.980000	705.500000	23.991899	0.474604	692.574341
24.000000	689.500000	24.011900	0.474152	691.927734
24.020000	691.000000	24.031900	0.474459	691.281738
24.040001	700.666992	24.051899	0.475510	690.635986
24.059999	709.333008	24.071898	0.477291	689.990845
24.080000	707.000000	24.091896	0.479795	689.345703
24.100000	694.000000	24.111897	0.483016	688.701050
24.120001	709.166992	24.131899	0.486954	688.056641
24.140001	699.166992	24.151897	0.491608	687.412476
24.160000	685.833008	24.171896	0.496985	686.768799
24.180000	678.500000	24.191895	0.503093	686.125366
24.200001	673.333008	24.211895	0.509944	685.482178
24.220001	674.666992	24.231895	0.517553	684.839355
24.240002	710.333008	24.251894	0.525938	684.196655
24.260000	706.333008	24.271893	0.535121	683.554321
24.280001	673.166992	24.291893	0.545130	682.912231
24.299999	669.000000	24.311892	0.555992	682.270386
24.320000	702.333008	24.331892	0.567743	681.628540

24.340000	675.000000	24.351891	0.580419	680.987427
24.359999	673.166992	24.371889	0.594064	680.346313
24.379999	677.333008	24.391888	0.608728	679.705322
24.400000	680.166992	24.411888	0.624464	679.064697
24.420000	680.166992	24.431889	0.641332	678.424194
24.440001	653.000000	24.451889	0.659398	677.783691
24.459999	674.833008	24.471888	0.678734	677.143677
24.480000	657.666992	24.491886	0.699424	676.503662
24.500000	658.500000	24.511887	0.721561	675.864014
24.520000	652.333008	24.531887	0.745245	675.224243
24.540001	666.333008	24.551886	0.690743	674.584839
24.559999	660.000000	24.571884	0.720012	673.945557
24.580000	657.333008	24.591883	0.751124	673.306396
24.600000	664.000000	24.611885	0.784245	672.667358
24.620001	667.500000	24.631884	0.819542	672.028564
24.640001	657.666992	24.651884	0.857225	671.389771
24.660000	668.166992	24.671883	0.897510	670.751099
24.680000	657.333008	24.691881	0.940656	670.112671
24.700001	642.666992	24.711882	0.986952	669.474365
24.720001	683.666992	24.731880	1.036715	668.836060
24.740002	674.333008	24.751881	1.090326	668.197754
24.760000	672.000000	24.771881	1.148206	667.559692
24.780001	676.500000	24.791880	1.210837	666.921753
24.799999	662.166992	24.811878	1.278794	666.283691
24.820000	648.500000	24.831877	1.352737	665.645874
24.840000	633.666992	24.851877	1.433451	665.008057
24.859999	647.166992	24.871876	1.521837	664.370483
24.879999	664.666992	24.891874	1.618994	663.732788
24.900000	656.500000	24.911877	1.726254	663.094971
24.920000	653.166992	24.931875	1.845168	662.457397
24.940001	665.333008	24.951876	1.977715	661.819580
24.959999	646.000000	24.971872	2.126266	661.182251
24.980000	688.333008	24.991873	2.293901	660.544678
25.000000	656.000000	25.011873	2.484438	659.907227
25.020000	652.000000	25.031872	2.702814	659.269653
25.040001	669.166992	25.051872	2.955577	658.632202
25.059999	652.000000	25.071871	3.251381	657.994629
25.080000	653.333008	25.091871	3.602204	657.357056

25.100000	647.833008	25.111872	4.024916	656.719727
25.120001	641.333008	25.131870	4.544730	656.082031
25.140001	644.833008	25.151871	5.202643	655.444580
25.160000	642.166992	25.171867	6.070775	654.807129
25.180000	636.500000	25.191868	7.284809	654.169312
25.200001	666.833008	25.211866	9.095405	653.531616
25.220001	656.833008	25.231869	11.936110	652.893921
25.240002	663.333008	25.251869	16.475616	652.256104
25.260000	667.666992	25.271866	23.655283	651.618408
25.280001	667.500000	25.291866	34.839157	650.980469
25.299999	707.000000	25.311863	52.388439	650.342773
25.320000	765.166992	25.331863	80.401871	649.704590
25.340000	765.666992	25.351862	118.714890	649.066650
25.359999	778.166992	25.371861	136.446396	648.428467
25.379999	760.000000	25.391863	102.726860	647.790283
25.400000	696.500000	25.411861	60.314541	647.151855
25.420000	681.666992	25.431862	35.944523	646.513428
25.440001	647.166992	25.451860	25.286074	645.875000
25.459999	666.166992	25.471859	21.438343	645.236450
25.480000	650.166992	25.491859	20.539665	644.597778
25.500000	658.000000	25.511858	20.929295	643.958984
25.520000	644.500000	25.531858	22.088909	643.319946
25.540001	650.666992	25.551859	23.983078	642.680908
25.559999	644.333008	25.571857	26.744307	642.041748
25.580000	652.833008	25.591856	30.581285	641.402344
25.600000	650.166992	25.611856	35.800266	640.763062
25.620001	661.500000	25.631857	42.890759	640.123535
25.640001	657.500000	25.651855	52.703506	639.483643
25.660000	677.833008	25.671854	66.820709	638.843994
25.680000	709.333008	25.691853	88.254257	638.203979
25.700001	720.666992	25.711855	122.554237	637.563599
25.720001	804.333008	25.731853	179.067810	636.923462
25.740002	882.166992	25.751854	272.012146	636.282959
25.760000	1029.330078	25.771852	421.522766	635.642334
25.780001	1287.830078	25.791851	658.315308	635.001709
25.799999	1653.830078	25.811850	1037.721924	634.360718
25.820000	2255.330078	25.831848	1641.030151	633.719604
25.840000	2777.670166	25.851849	2364.036133	633.078247

25.859999	2768.500000	25.871847	2436.368408	632.436890
25.879999	2183.170166	25.891848	1624.842407	631.795044
25.900000	1532.500000	25.911848	862.579712	631.153198
25.920000	1098.000000	25.931847	450.395905	630.511230
25.940001	901.500000	25.951847	262.115570	629.868896
25.959999	802.333008	25.971844	177.900162	629.226685
25.980000	728.000000	25.991844	135.114212	628.583862
26.000000	720.500000	26.011843	107.440025	627.941284
26.020000	669.666992	26.031845	86.078217	627.298096
26.040001	681.833008	26.051844	68.860008	626.654785
26.059999	658.833008	26.071842	55.224030	626.011353
26.080000	647.333008	26.091843	44.664444	625.367798
26.100000	628.500000	26.111841	36.562222	624.723877
26.120001	636.666992	26.131842	30.319242	624.079590
26.140001	658.333008	26.151840	25.454483	623.435425
26.160000	629.000000	26.171839	21.609953	622.790894
26.180000	634.500000	26.191839	18.529016	622.146118
26.200001	644.333008	26.211840	16.028500	621.501099
26.220001	632.666992	26.231840	13.975503	620.855713
26.240002	615.166992	26.251839	12.272479	620.210327
26.260000	606.666992	26.271837	10.846328	619.564697
26.280001	617.500000	26.291836	9.641846	618.918823
26.299999	608.500000	26.311834	8.616688	618.272583
26.320000	613.166992	26.331833	7.782932	617.626221
26.340000	605.666992	26.351835	7.026045	616.979370
26.359999	606.333008	26.371834	6.369521	616.332642
26.379999	598.500000	26.391832	5.796825	615.685425
26.400000	598.166992	26.411833	5.294684	615.037964
26.420000	633.666992	26.431831	4.852439	614.390259
26.440001	612.500000	26.451832	4.461182	613.742432
26.459999	613.000000	26.471828	4.113737	613.094360
26.480000	640.833008	26.491829	3.803926	612.445801
26.500000	640.000000	26.511829	3.526767	611.796997
26.520000	607.833008	26.531830	3.278011	611.148071
26.540001	620.000000	26.551830	3.054070	610.498779
26.559999	598.500000	26.571827	2.851938	609.849365
26.580000	600.500000	26.591827	2.668940	609.199585
26.600000	597.500000	26.611826	2.502906	608.549438

26.620001	587.666992	26.631826	2.351892	607.899170
26.640001	602.333008	26.651825	2.214276	607.248535
26.660000	586.000000	26.671825	2.088600	606.597656
26.680000	591.333008	26.691824	1.973646	605.946655
26.700001	610.333008	26.711824	1.868305	605.295166
26.720001	582.666992	26.731825	1.771642	604.643311
26.740002	579.333008	26.751823	1.682833	603.991455
26.760000	576.500000	26.771822	1.601136	603.339233
26.780001	590.333008	26.791821	1.525908	602.686646
26.799999	585.166992	26.811819	1.456582	602.033813
26.820000	584.000000	26.831820	1.392649	601.380859
26.840000	602.833008	26.851820	1.333670	600.727417
26.859999	615.833008	26.871817	1.279264	600.073853
26.879999	602.000000	26.891817	1.229059	599.419800
26.900000	581.666992	26.911818	1.182757	598.765625
26.920000	580.333008	26.931816	1.140089	598.111084
26.940001	589.333008	26.951817	1.100804	597.456177
26.959999	590.166992	26.971813	1.064700	596.801270
26.980000	582.833008	26.991816	1.068265	596.145752
27.000000	573.000000	27.011814	1.040093	595.490234
27.020000	577.666992	27.031815	1.014635	594.834229
27.040001	566.000000	27.051813	0.991781	594.178101
27.059999	591.000000	27.071812	0.971430	593.521362
27.080000	589.500000	27.091812	0.953502	592.864624
27.100000	584.000000	27.111811	0.937937	592.207397
27.120001	581.333008	27.131811	0.924690	591.550049
27.140001	578.500000	27.151812	0.913735	590.892334
27.160000	578.500000	27.171810	0.905064	590.234375
27.180000	581.833008	27.191809	0.898683	589.576050
27.200001	593.500000	27.211809	0.894620	588.917480
27.220001	572.333008	27.231808	0.892922	588.258667
27.240002	573.333008	27.251808	0.893658	587.599365
27.260000	567.500000	27.271805	0.896918	586.940063
27.280001	565.166992	27.291805	0.902820	586.280273
27.299999	578.166992	27.311806	0.918708	585.620361
27.320000	590.333008	27.331804	0.930599	584.959961
27.340000	576.500000	27.351805	0.945679	584.299316
27.359999	563.166992	27.371801	0.964203	583.638550

27.379999	590.833008	27.391802	0.986482	582.977295
27.400000	571.666992	27.411800	1.012874	582.315674
27.420000	578.166992	27.431801	1.043817	581.653931
27.440001	580.000000	27.451799	1.079817	580.991943
27.459999	577.166992	27.471800	1.121491	580.329712
27.480000	586.833008	27.491798	1.169556	579.667114
27.500000	566.333008	27.511799	1.224891	579.004028
27.520000	556.166992	27.531799	1.288532	578.340942
27.540001	576.500000	27.551798	1.361728	577.677368
27.559999	562.833008	27.571796	1.446006	577.013794
27.580000	553.166992	27.591795	1.543217	576.349609
27.600000	569.166992	27.611795	1.655647	575.685303
27.620001	559.000000	27.631796	1.786092	575.020508
27.640001	571.000000	27.651796	1.938046	574.355652
27.660000	588.000000	27.671793	2.115856	573.690430
27.680000	601.166992	27.691793	2.325142	573.025085
27.700001	603.666992	27.711792	2.572949	572.359314
27.720001	640.000000	27.731792	2.868515	571.693176
27.740002	661.000000	27.751791	3.223811	571.027039
27.760000	685.333008	27.771790	3.654820	570.360413
27.780001	693.833008	27.791792	4.183140	569.693665
27.799999	680.500000	27.811789	4.837920	569.026489
27.820000	634.500000	27.831789	5.660576	568.359070
27.840000	601.000000	27.851788	6.709994	567.691528
27.859999	578.833008	27.871786	8.074869	567.023621
27.879999	574.333008	27.891785	9.896695	566.355347
27.900000	565.833008	27.911785	12.419294	565.686951
27.920000	576.000000	27.931784	16.086910	565.018250
27.940001	575.500000	27.951786	21.723824	564.349182
27.959999	572.833008	27.971783	30.789873	563.679932
27.980000	600.333008	27.991783	45.703808	563.010620
28.000000	616.666992	28.011782	70.174110	562.340698
28.020000	650.000000	28.031782	109.864586	561.670776
28.040001	708.666992	28.051781	174.193390	561.000366
28.059999	849.333008	28.071779	279.281494	560.330139
28.080000	986.333008	28.091780	437.460022	559.659241
28.100000	1091.669922	28.111780	576.859924	558.988098
28.120001	1018.169983	28.131781	515.185486	558.317017

28.140001	868.500000	28.151779	317.477875	557.645325
28.160000	721.833008	28.171778	168.759628	556.973633
28.180000	651.833008	28.191776	91.987495	556.301758
28.200001	595.833008	28.211777	56.256214	555.629517
28.220001	587.500000	28.231775	39.171898	554.956848
28.240002	574.833008	28.251776	29.614290	554.284180
28.260000	550.333008	28.271772	23.343777	553.611389
28.280001	572.500000	28.291775	18.652388	552.938171
28.299999	556.666992	28.311771	15.045829	552.264832
28.320000	543.833008	28.331772	12.305425	551.591370
28.340000	560.333008	28.351770	10.258777	550.917419
28.359999	552.166992	28.371769	8.746465	550.243469
28.379999	547.666992	28.391769	7.636279	549.569092
28.400000	536.000000	28.411768	6.829107	548.894714
28.420000	529.500000	28.431768	6.254572	548.219971
28.440001	533.666992	28.451769	5.906371	547.544983
28.459999	549.500000	28.471767	5.670469	546.869873
28.480000	529.833008	28.491766	5.567841	546.194641
28.500000	532.666992	28.511766	5.587826	545.519043
28.520000	534.333008	28.531765	5.727372	544.843323
28.540001	514.833008	28.551765	5.990496	544.167358
28.559999	552.166992	28.571762	6.388347	543.491272
28.580000	552.500000	28.591763	6.940624	542.815002
28.600000	536.833008	28.611763	7.677151	542.138367
28.620001	527.833008	28.631763	8.641564	541.461731
28.640001	557.333008	28.651762	9.896626	540.784729
28.660000	539.166992	28.671761	11.533689	540.107910
28.680000	549.833008	28.691759	13.688528	539.430542
28.700001	534.333008	28.711760	16.572950	538.753113
28.720001	540.833008	28.731758	20.538080	538.075500
28.740002	529.333008	28.751759	26.209362	537.397827
28.760000	543.166992	28.771757	34.735298	536.719788
28.780001	564.000000	28.791758	48.205879	536.041626
28.799999	578.500000	28.811756	70.207710	535.363525
28.820000	606.000000	28.831755	106.493507	534.685120
28.840000	668.500000	28.851755	165.933685	534.006531
28.859999	772.833008	28.871752	262.732941	533.328003
28.879999	937.666992	28.891752	421.521301	532.648987

28.900000	1219.830078	28.911751	676.514465	531.970032
28.920000	1525.000000	28.931753	999.025085	531.290833
28.940001	1622.000000	28.951752	1106.620728	530.611511
28.959999	1353.169922	28.971750	809.098328	529.932251
28.980000	1002.169983	28.991749	454.329742	529.252502
29.000000	760.333008	29.011749	241.648239	528.572937
29.020000	632.166992	29.031748	138.051254	527.892944
29.040001	593.500000	29.051748	89.130165	527.213135
29.059999	558.333008	29.071745	64.022751	526.533203
29.080000	544.166992	29.091747	48.781338	525.852844
29.100000	548.333008	29.111746	38.003929	525.172607
29.120001	528.500000	29.131746	29.826107	524.492065
29.140001	527.000000	29.151745	23.560061	523.811707
29.160000	525.500000	29.171743	18.797140	523.131104
29.180000	520.833008	29.191742	15.191938	522.450562
29.200001	513.833008	29.211742	12.449876	521.769592
29.220001	525.166992	29.231741	10.341058	521.088867
29.240002	515.333008	29.251741	8.695907	520.407715
29.260000	517.333008	29.271740	7.394186	519.726807
29.280001	519.833008	29.291740	6.350093	519.045654
29.299999	509.832977	29.311737	5.545260	518.364502
29.320000	516.333008	29.331738	4.850372	517.683289
29.340000	530.666992	29.351738	4.274531	517.001892
29.359999	512.500000	29.371735	3.793175	516.320435
29.379999	515.333008	29.391735	3.433697	515.638977
29.400000	520.166992	29.411734	3.090335	514.957520
29.420000	492.166992	29.431736	2.797029	514.275818
29.440001	517.666992	29.451735	2.545088	513.594238
29.459999	530.000000	29.471733	2.327465	512.912598
29.480000	517.666992	29.491732	2.138566	512.230774
29.500000	514.000000	29.511732	1.973861	511.549072
29.520000	503.166992	29.531731	1.829705	510.867371
29.540001	518.500000	29.551731	1.703060	510.185486
29.559999	508.666992	29.571728	1.591475	509.503723
29.580000	524.666992	29.591730	1.492840	508.821716
29.600000	532.500000	29.611729	1.405482	508.140015
29.620001	533.166992	29.631729	1.327931	507.458008
29.640001	539.833008	29.651728	1.258986	506.776184

29.660000	521.666992	29.671726	1.197609	506.094299
29.680000	514.500000	29.691725	1.142923	505.412476
29.700001	516.666992	29.711725	1.094175	504.730347
29.720001	514.666992	29.731724	1.086520	504.048523
29.740002	501.000000	29.751726	1.048468	503.366638
29.760000	511.000000	29.771723	1.014717	502.684937
29.780001	511.500000	29.791723	0.984839	502.002930
29.799999	509.000000	29.811720	0.958489	501.321350
29.820000	513.666992	29.831720	0.935350	500.639282
29.840000	500.666992	29.851719	0.915160	499.957642
29.859999	502.832977	29.871717	0.897684	499.275940
29.879999	505.666992	29.891716	0.882719	498.594299
29.900000	511.500000	29.911716	0.870087	497.912598
29.920000	508.166992	29.931717	0.816265	497.230957
29.940001	489.666992	29.951717	0.809093	496.549438
29.959999	503.000000	29.971714	0.803811	495.868103
29.980000	486.166992	29.991714	0.800320	495.186584
30.000000	484.000000	30.011713	0.798540	494.505432
30.020000	490.500000	30.031713	0.798403	493.824158
30.040001	499.666992	30.051712	0.799852	493.142944
30.059999	491.166992	30.071711	0.802841	492.461731
30.080000	482.666992	30.091711	0.807334	491.780701
30.100000	496.666992	30.111712	0.813305	491.099792
30.120001	489.166992	30.131710	0.820735	490.418945
30.140001	490.000000	30.151711	0.829615	489.738342
30.160000	492.332977	30.171707	0.839941	489.057739
30.180000	485.832977	30.191708	0.851722	488.377258
30.200001	484.000000	30.211706	0.864969	487.696777
30.220001	484.500000	30.231707	0.879703	487.016479
30.240002	486.832977	30.251707	0.895953	486.336243
30.260000	497.666992	30.271706	0.913752	485.656250
30.280001	511.500000	30.291704	0.933143	484.976257
30.299999	492.500000	30.311703	0.954178	484.296631
30.320000	484.332977	30.331701	0.976915	483.616821
30.340000	481.500000	30.351702	1.001424	482.937561
30.359999	474.500000	30.371698	1.027772	482.258240
30.379999	482.500000	30.391699	1.056057	481.579163
30.400000	495.500000	30.411699	1.086367	480.899963

30.420000	460.166992	30.431700	1.118810	480.221069
30.440001	480.666992	30.451698	1.153499	479.542419
30.459999	477.000000	30.471697	1.190567	478.864075
30.480000	476.000000	30.491695	1.230158	478.185486
30.500000	488.166992	30.511696	1.272436	477.507446
30.520000	482.832977	30.531694	1.317565	476.829529
30.540001	495.832977	30.551695	1.365749	476.151733
30.559999	492.000000	30.571692	1.417186	475.474182
30.580000	489.332977	30.591694	1.472135	474.796631
30.600000	496.666992	30.611692	1.523906	474.119629
30.620001	494.332977	30.631693	1.586864	473.442566
30.640001	484.000000	30.651691	1.654163	472.765930
30.660000	515.000000	30.671690	1.726160	472.089355
30.680000	502.500000	30.691689	1.803240	471.413147
30.700001	504.332977	30.711689	1.885843	470.736938
30.720001	512.333008	30.731688	1.974429	470.061279
30.740002	506.666992	30.751690	2.069556	469.385620
30.760000	498.832977	30.771687	2.171769	468.710388
30.780001	505.666992	30.791687	2.281777	468.035339
30.799999	498.166992	30.811684	2.400278	467.360535
30.820000	491.166992	30.831684	2.528144	466.685852
30.840000	492.000000	30.851683	2.666273	466.011658
30.859999	493.666992	30.871681	2.815726	465.337646
30.879999	485.332977	30.891680	2.977688	464.664001
30.900000	491.500000	30.911680	3.153518	463.990356
30.920000	518.000000	30.931681	3.290536	463.317200
30.940001	523.166992	30.951679	3.500259	462.644287
30.959999	583.500000	30.971678	3.729019	461.971680
30.980000	609.833008	30.991676	3.979085	461.299438
31.000000	672.166992	31.011677	4.253093	460.627502
31.020000	718.333008	31.031675	4.554000	459.955688
31.040001	714.833008	31.051676	4.885345	459.284363
31.059999	673.166992	31.071672	5.251080	458.613403
31.080000	611.833008	31.091675	5.656100	457.942505
31.100000	548.000000	31.111673	6.105755	457.272095
31.120001	525.000000	31.131674	6.606680	456.601868
31.140001	493.832977	31.151672	7.166484	455.932373
31.160000	481.332977	31.171671	7.794373	455.262939

31.180000	490.166992	31.191669	8.501296	454.593872
31.200001	478.500000	31.211670	9.300503	453.925232
31.220001	494.500000	31.231668	10.207767	453.256958
31.240002	481.832977	31.251671	11.242771	452.588867
31.260000	488.666992	31.271667	12.428793	451.921326
31.280001	476.166992	31.291668	13.795771	451.254028
31.299999	475.500000	31.311665	15.379790	450.587341
31.320000	477.500000	31.331665	17.227465	449.920776
31.340000	460.832977	31.351664	19.396652	449.254822
31.359999	460.332977	31.371662	21.962263	448.589111
31.379999	469.832977	31.391661	25.021132	447.923828
31.400000	481.000000	31.411663	28.700977	447.258850
31.420000	481.000000	31.431662	33.169064	446.594543
31.440001	487.166992	31.451662	38.653858	445.930420
31.459999	511.332977	31.471659	45.464951	445.266846
31.480000	506.000000	31.491657	54.038906	444.603577
31.500000	500.832977	31.511658	64.999039	443.940857
31.520000	499.332977	31.531656	79.267212	443.278381
31.540001	533.666992	31.551657	98.286957	442.616638
31.559999	539.833008	31.571653	124.439072	441.955139
31.580000	589.333008	31.591656	161.919693	441.294006
31.600000	638.000000	31.611654	218.243500	440.633423
31.620001	727.500000	31.631655	306.850006	439.973328
31.640001	832.500000	31.651653	450.745056	439.313721
31.660000	1027.669922	31.671652	687.790955	438.654602
31.680000	1408.669922	31.691650	1079.466675	437.995850
31.700001	2041.000000	31.711651	1728.929688	437.337585
31.720001	3237.670166	31.731649	2808.581787	436.679810
31.740002	5010.000000	31.751652	4513.303223	436.022522
31.760000	6838.669922	31.771648	6507.279297	435.365845
31.780001	7174.169922	31.791649	6979.805176	434.709351
31.799999	5452.329590	31.811646	5089.783203	434.053711
31.820000	3275.170166	31.831646	2925.186035	433.398560
31.840000	1947.000000	31.851645	1604.239990	432.743835
31.859999	1253.500000	31.871643	939.472351	432.089661
31.879999	941.000000	31.891642	613.244385	431.435791
31.900000	793.000000	31.911640	442.388702	430.782776
31.920000	718.166992	31.931643	342.268127	430.129944

31.940001	651.333008	31.951641	278.160614	429.477905
31.959999	615.333008	31.971640	237.065781	428.826355
31.980000	607.000000	31.991638	214.707947	428.175232
32.000000	611.333008	32.011639	210.882736	427.524780
32.020000	615.666992	32.031635	228.972183	426.875000
32.040001	645.666992	32.051636	277.326141	426.225464
32.060001	751.000000	32.071636	371.633911	425.576660
32.080002	915.166992	32.091633	538.822815	424.928467
32.099998	1190.330078	32.111629	824.979858	424.281006
32.119999	1695.830078	32.131630	1308.791382	423.633972
32.139999	2551.000000	32.151630	2092.239502	422.987427
32.160000	3437.830078	32.171631	3097.846924	422.341370
32.180000	3741.670166	32.191631	3557.221680	421.695984
32.200001	3043.000000	32.211632	2786.412354	421.051392
32.220001	2037.500000	32.231628	1659.443359	420.407288
32.240002	1280.830078	32.251629	913.860596	419.763794
32.260002	938.500000	32.271629	527.479004	419.120789
32.280003	747.166992	32.291630	335.803284	418.478577
32.299999	651.166992	32.311623	235.031860	417.837158
32.320000	597.333008	32.331623	175.317017	417.196045
32.340000	554.333008	32.351624	135.424316	416.555664
32.360001	534.500000	32.371624	106.756927	415.915833
32.380001	534.166992	32.391621	85.631836	415.276978
32.400002	526.833008	32.411621	70.038582	414.638245
32.420002	536.666992	32.431622	58.588974	414.000366
32.440002	528.333008	32.451622	50.229588	413.363220
32.460003	522.833008	32.471619	44.182678	412.726868
32.480003	477.666992	32.491619	39.897190	412.091064
32.500000	478.832977	32.511620	37.005463	411.455688
32.520000	459.666992	32.531616	35.273685	410.821472
32.540001	451.332977	32.551617	34.569714	410.187439
32.560001	441.332977	32.571617	34.845894	409.554382
32.580002	447.166992	32.591618	36.130314	408.921936
32.599998	453.332977	32.611610	38.530006	408.290344
32.619999	458.166992	32.631611	42.251476	407.659302
32.639999	461.832977	32.651611	47.629940	407.028931
32.660000	444.332977	32.671612	55.198952	406.399231
32.680000	471.832977	32.691608	65.817253	405.770386

32.700001	494.332977	32.711609	80.936188	405.142029
32.720001	504.832977	32.731609	103.094154	404.514709
32.740002	522.166992	32.751606	136.840775	403.888000
32.760002	566.000000	32.771606	190.291229	403.261841
32.780003	658.166992	32.791607	277.391602	402.636475
32.799999	775.833008	32.811604	421.328278	402.011963
32.820000	990.500000	32.831600	660.427490	401.388306
32.840000	1385.669922	32.851604	1059.651245	400.764954
32.860001	2125.500000	32.871605	1726.029785	400.142700
32.880001	3256.000000	32.891605	2776.458740	399.520996
32.900002	4337.169922	32.911602	4007.791260	398.900269
32.920002	4577.669922	32.931602	4344.328125	398.280273
32.940002	3619.330078	32.951603	3229.734863	397.661011
32.960003	2237.830078	32.971600	1884.358521	397.042480
32.980003	1380.000000	32.991600	1037.805908	396.424744
33.000000	919.666992	33.011597	602.222229	395.807861
33.020000	694.666992	33.031597	383.557526	395.191589
33.040001	618.000000	33.051594	266.248810	394.576416
33.060001	538.833008	33.071594	195.518921	393.961670
33.080002	498.332977	33.091595	147.910645	393.347900
33.099998	458.666992	33.111591	113.697044	392.735107
33.119999	453.832977	33.131588	88.478920	392.122803
33.139999	462.666992	33.151588	69.765167	391.511597
33.160000	437.832977	33.171589	55.822113	390.901001
33.180000	437.000000	33.191589	45.348621	390.291199
33.200001	423.332977	33.211590	37.385174	389.682251
33.220001	403.500000	33.231590	31.244146	389.074219
33.240002	393.832977	33.251591	26.440849	388.466797
33.260002	393.666992	33.271587	22.634718	387.860535
33.280003	400.832977	33.291588	19.581966	387.254822
33.299999	403.500000	33.311584	17.109295	386.650269
33.320000	418.500000	33.331585	15.088243	386.046387
33.340000	393.332977	33.351582	13.425129	385.443359
33.360001	397.666992	33.371582	12.048464	384.841187
33.380001	405.666992	33.391582	10.904904	384.239929
33.400002	391.166992	33.411579	9.953629	383.639587
33.420002	396.666992	33.431580	9.162826	383.039917
33.440002	401.666992	33.451580	8.508558	382.441162

33.460003	405.166992	33.471581	7.972400	381.843384
33.480003	406.500000	33.491577	7.540583	381.246399
33.500000	395.666992	33.511574	7.202947	380.650513
33.520000	395.666992	33.531574	6.952668	380.055298
33.540001	392.666992	33.551575	6.785985	379.460938
33.560001	399.000000	33.571575	6.701895	378.867554
33.580002	391.332977	33.591576	6.702294	378.275085
33.599998	393.166992	33.611572	6.792218	377.683594
33.619999	389.666992	33.631569	6.989199	377.092957
33.639999	379.832977	33.651569	7.289189	376.503174
33.660000	387.666992	33.671570	7.719647	375.914185
33.680000	397.666992	33.691570	8.307098	375.326172
33.700001	395.832977	33.711567	9.088208	374.739258
33.720001	377.000000	33.731567	10.114517	374.153137
33.740002	397.666992	33.751568	11.457842	373.568054
33.760002	386.332977	33.771564	13.220377	372.983887
33.780003	387.666992	33.791565	15.552574	372.400574
33.799999	392.332977	33.811562	18.680109	371.818420
33.820000	386.666992	33.831562	22.963894	371.236877
33.840000	391.666992	33.851559	29.004425	370.656555
33.860001	397.500000	33.871559	37.856567	370.076843
33.880001	428.332977	33.891563	51.393845	369.498291
33.900002	443.832977	33.911560	72.895287	368.920715
33.920002	462.332977	33.931561	107.994415	368.344055
33.940002	515.000000	33.951561	166.102493	367.768433
33.960003	611.000000	33.971561	263.038696	367.193726
33.980003	783.166992	33.991558	425.692383	366.620117
34.000000	1071.169922	34.011555	695.687378	366.047424
34.020000	1493.500000	34.031555	1099.357300	365.475586
34.040001	1818.169922	34.051556	1488.956787	364.904724
34.060001	1796.000000	34.071552	1458.571167	364.335022
34.080002	1372.330078	34.091553	1006.773865	363.766235
34.099998	948.666992	34.111549	576.363525	363.198425
34.119999	694.333008	34.131546	322.201019	362.631653
34.139999	573.833008	34.151546	191.727219	362.065918
34.160000	513.833008	34.171547	124.603844	361.501038
34.180000	483.166992	34.191547	87.288269	360.937195
34.200001	457.166992	34.211544	64.111328	360.374573

34.220001	453.666992	34.231548	48.368107	359.812622
34.240002	457.332977	34.251549	37.078529	359.251831
34.260002	458.166992	34.271545	28.807987	358.692078
34.280003	486.666992	34.291546	22.698240	358.133240
34.299999	496.166992	34.311543	18.155067	357.575684
34.320000	536.833008	34.331543	14.740488	357.018860
34.340000	535.166992	34.351540	12.140481	356.463318
34.360001	499.500000	34.371540	10.130454	355.908752
34.380001	460.500000	34.391541	8.553960	355.355164
34.400002	411.832977	34.411537	7.300650	354.802612
34.420002	399.500000	34.431538	6.291358	354.251099
34.440002	399.832977	34.451538	5.469514	353.700562
34.460003	384.000000	34.471539	4.793438	353.151123
34.480003	369.832977	34.491535	4.232216	352.602844
34.500000	375.666992	34.511532	3.762329	352.055725
34.520000	358.500000	34.531532	3.365860	351.509399
34.540001	359.332977	34.551529	3.029173	350.964294
34.560001	356.832977	34.571533	2.741331	350.419922
34.580002	346.000000	34.591534	2.494038	349.876953
34.599998	363.166992	34.611530	2.280544	349.335022
34.619999	350.500000	34.631527	2.095415	348.794128
34.639999	362.666992	34.651527	1.934287	348.254211
34.660000	342.500000	34.671528	1.793684	347.715515
34.680000	346.166992	34.691525	1.670766	347.177795
34.700001	342.000000	34.711525	1.563155	346.641174
34.720001	348.166992	34.731525	1.468967	346.105713
34.740002	342.332977	34.751526	1.386645	345.571167
34.760002	342.166992	34.771523	1.314938	345.037964
34.780003	356.332977	34.791523	1.252802	344.505798
34.799999	330.500000	34.811520	1.199456	343.974670
34.820000	357.000000	34.831516	1.154277	343.444519
34.840000	355.000000	34.851517	1.116817	342.915710
34.860001	344.332977	34.871517	1.086811	342.387756
34.880001	333.332977	34.891518	1.064144	341.860962
34.900002	343.000000	34.911514	1.048872	341.335388
34.920002	338.332977	34.931519	1.041219	340.810669
34.940002	343.166992	34.951519	1.041628	340.287292
34.960003	341.832977	34.971516	1.050759	339.765015

34.980003	346.500000	34.991516	1.069567	339.243774
35.000000	347.666992	35.011513	1.099346	338.723816
35.020000	344.666992	35.031513	1.141854	338.204773
35.040001	344.166992	35.051510	1.199398	337.687012
35.060001	353.666992	35.071510	1.275094	337.170349
35.080002	351.666992	35.091511	1.331194	336.654724
35.099998	352.166992	35.111504	1.457710	336.140381
35.119999	348.332977	35.131504	1.578272	335.627075
35.139999	353.332977	35.151505	1.786420	335.114807
35.160000	339.000000	35.171501	2.054505	334.603882
35.180000	338.666992	35.191502	2.403129	334.093994
35.200001	332.666992	35.211502	2.862248	333.585144
35.220001	330.666992	35.231503	3.477431	333.077515
35.240002	319.000000	35.251499	4.321055	332.571167
35.260002	345.666992	35.271503	5.515105	332.065674
35.280003	330.500000	35.291504	7.270018	331.561523
35.299999	340.666992	35.311497	9.956802	331.058594
35.320000	344.500000	35.331497	14.227881	330.556702
35.340000	347.332977	35.351498	21.198429	330.055969
35.360001	359.000000	35.371498	32.760345	329.556396
35.380001	377.332977	35.391495	52.133495	329.057983
35.400002	407.500000	35.411495	84.786934	328.560791
35.420002	492.000000	35.431496	138.644257	328.064636
35.440002	558.666992	35.451492	216.332809	327.569763
35.460003	632.833008	35.471493	284.027374	327.075928
35.480003	631.833008	35.491493	268.998932	326.583252
35.500000	539.000000	35.511490	183.355515	326.091919
35.520000	487.332977	35.531487	105.687378	325.601807
35.540001	414.166992	35.551487	59.881958	325.112732
35.560001	370.500000	35.571487	36.014332	324.624817
35.580002	361.500000	35.591484	23.473320	324.138062
35.599998	362.666992	35.611481	16.377167	323.652649
35.619999	357.332977	35.631485	11.951782	323.168091
35.639999	343.832977	35.651482	8.959825	322.684998
35.660000	338.332977	35.671482	6.834847	322.202881
35.680000	333.666992	35.691483	5.291563	321.721985
35.700001	329.000000	35.711483	4.158556	321.242493
35.720001	325.166992	35.731480	3.319203	320.763977

35.740002	318.166992	35.751480	2.689925	320.286682
35.760002	319.000000	35.771481	2.211622	319.810608
35.780003	319.000000	35.791477	1.842645	319.335754
35.799999	309.666992	35.811474	1.553713	318.862061
35.820000	333.332977	35.831474	1.324271	318.389587
35.840000	325.832977	35.851475	1.139784	317.918213
35.860001	326.832977	35.871471	0.989746	317.448181
35.880001	328.832977	35.891472	0.866389	316.979187
35.900002	332.332977	35.911472	0.764009	316.511414
35.920002	324.000000	35.931469	0.678303	316.044983
35.940002	313.000000	35.951469	0.605942	315.579590
35.960003	310.166992	35.971470	0.544403	315.115417
35.980003	307.332977	35.991470	0.491703	314.652527
36.000000	303.166992	36.011467	0.446287	314.190857
36.020000	303.332977	36.031467	0.406899	313.730286
36.040001	310.166992	36.051468	0.372553	313.270996
36.060001	297.500000	36.071465	0.342451	312.812927
36.080002	298.000000	36.091465	0.315928	312.355957
36.099998	310.666992	36.111462	0.292459	311.900330
36.119999	302.500000	36.131458	0.271596	311.445862
36.139999	291.666992	36.151459	0.217981	310.992554
36.160000	301.500000	36.171459	0.201907	310.540466
36.180000	295.666992	36.191456	0.187501	310.089722
36.200001	302.000000	36.211456	0.174541	309.640076
36.220001	305.832977	36.231457	0.162847	309.191528
36.240002	311.666992	36.251457	0.152264	308.744263
36.260002	300.500000	36.271454	0.142661	308.298401
36.280003	303.000000	36.291454	0.133919	307.853577
36.299999	310.000000	36.311451	0.125944	307.410156
36.320000	303.000000	36.331451	0.089091	306.967712
36.340000	283.000000	36.351452	0.083149	306.526550
36.360001	298.332977	36.371452	0.077723	306.086609
36.380001	299.500000	36.391449	0.073258	305.647949
36.400002	291.832977	36.411449	0.068726	305.210449
36.420002	295.500000	36.431450	0.064567	304.774170
36.440002	292.166992	36.451450	0.060743	304.339111
36.460003	290.166992	36.471447	0.057222	303.905396
36.480003	293.000000	36.491447	0.053974	303.472778

36.500000	292.832977	36.511444	0.050975	303.041443
36.520000	290.832977	36.531441	0.048201	302.611389
36.540001	299.832977	36.551441	0.045631	302.182373
36.560001	283.832977	36.571442	0.043247	301.754700
36.580002	291.000000	36.591438	0.041035	301.328247
36.599998	296.332977	36.611435	0.038978	300.903015
36.619999	295.166992	36.631435	0.037064	300.479004
36.639999	279.332977	36.651436	0.035281	300.056091
36.660000	287.832977	36.671432	0.033619	299.634583
36.680000	276.166992	36.691437	0.032068	299.214172
36.700001	282.332977	36.711437	0.030619	298.795044
36.720001	288.500000	36.731434	0.029266	298.377197
36.740002	283.166992	36.751434	0.028000	297.960510
36.760002	281.832977	36.771435	0.026816	297.545044
36.780003	290.166992	36.791431	0.025708	297.130859
36.799999	293.166992	36.811428	0.024670	296.717896
36.820000	287.166992	36.831429	0.023698	296.306152
36.840000	283.500000	36.851429	0.022788	295.895508
36.860001	277.332977	36.871426	0.021935	295.486267
36.880001	294.332977	36.891426	0.021137	295.078064
36.900002	277.332977	36.911427	0.020389	294.671265
36.920002	282.332977	36.931423	0.019690	294.265686
36.940002	282.000000	36.951424	0.019036	293.861206
36.960003	285.166992	36.971424	0.018426	293.458008
36.980003	275.500000	36.991421	0.017857	293.056152
37.000000	281.832977	37.011417	0.017327	292.655457
37.020000	270.166992	37.031418	0.016835	292.255859
37.040001	277.166992	37.051418	0.016379	291.857544
37.060001	281.666992	37.071419	0.015958	291.460510
37.080002	286.500000	37.091419	0.015572	291.064636
37.099998	283.500000	37.111416	0.015219	290.670166
37.119999	278.666992	37.131413	0.014899	290.276733
37.139999	288.166992	37.151413	0.014611	289.884583
37.160000	284.666992	37.171413	0.014356	289.493713
37.180000	269.332977	37.191410	0.014132	289.103882
37.200001	279.332977	37.211411	0.013942	288.715454
37.220001	296.332977	37.231411	0.013785	288.328247
37.240002	289.332977	37.251408	0.013661	287.942139

37.260002	293.832977	37.271408	0.018923	287.557373
37.280003	281.666992	37.291409	0.019048	287.173767
37.299999	291.000000	37.311401	0.019219	286.791504
37.320000	290.500000	37.331402	0.019438	286.410339
37.340000	292.332977	37.351402	0.012781	286.030457
37.360001	293.332977	37.371403	0.013429	285.651672
37.380001	284.332977	37.391399	0.014129	285.274231
37.400002	287.332977	37.411404	0.014887	284.897827
37.420002	269.332977	37.431404	0.015708	284.522766
37.440002	267.666992	37.451401	0.016601	284.149048
37.460003	265.166992	37.471401	0.017574	283.776367
37.480003	266.500000	37.491402	0.031854	283.404968
37.500000	278.166992	37.511395	0.033383	283.034790
37.520000	261.166992	37.531395	0.035044	282.665894
37.540001	283.332977	37.551395	0.036853	282.298096
37.560001	288.666992	37.571392	0.038830	281.931641
37.580002	269.666992	37.591393	0.041000	281.566223
37.599998	281.666992	37.611389	0.043393	281.202148
37.619999	274.500000	37.631390	0.046045	280.839294
37.639999	272.500000	37.651386	0.048997	280.477722
37.660000	270.166992	37.671387	0.052303	280.117126
37.680000	266.166992	37.691387	0.056029	279.757874
37.700001	267.166992	37.711384	0.060252	279.399719
37.720001	276.500000	37.731384	0.065077	279.042847
37.740002	281.832977	37.751385	0.070630	278.687256
37.760002	274.500000	37.771385	0.077076	278.332703
37.780003	271.666992	37.791386	0.084625	277.979431
37.799999	288.500000	37.811382	0.093553	277.627502
37.820000	276.832977	37.831379	0.104229	277.276672
37.840000	270.000000	37.851379	0.117148	276.927063
37.860001	276.500000	37.871380	0.138535	276.578613
37.880001	274.832977	37.891376	0.158396	276.231323
37.900002	259.166992	37.911377	0.183447	275.885254
37.920002	263.000000	37.931377	0.215616	275.540466
37.940002	269.500000	37.951378	0.257819	275.196777
37.960003	262.666992	37.971375	0.314627	274.854370
37.980003	267.332977	37.991375	0.393584	274.513092
38.000000	262.000000	38.011372	0.507468	274.173035

38.020000	260.500000	38.031368	0.678541	273.834259
38.040001	264.000000	38.051369	0.945694	273.496521
38.060001	266.000000	38.071369	1.376360	273.159943
38.080002	280.832977	38.091366	2.087097	272.824707
38.099998	258.166992	38.111362	3.279787	272.490570
38.119999	253.167007	38.131367	5.296337	272.157532
38.139999	268.832977	38.151363	8.627184	271.825867
38.160000	273.332977	38.171364	13.512912	271.495270
38.180000	266.000000	38.191364	18.285057	271.165771
38.200001	267.832977	38.211361	18.575424	270.837616
38.220001	262.500000	38.231361	13.697509	270.510498
38.240002	256.832977	38.251362	8.328460	270.184723
38.260002	253.667007	38.271362	4.855557	269.859955
38.280003	254.000000	38.291359	2.960840	269.536499
38.299999	263.500000	38.311356	1.945251	269.214142
38.320000	248.833008	38.331356	1.373420	268.892975
38.340000	256.832977	38.351353	1.027489	268.572968
38.360001	250.833008	38.371353	0.804073	268.254120
38.380001	255.500000	38.391354	0.653717	267.936462
38.400002	256.166992	38.411350	0.550887	267.619904
38.420002	261.832977	38.431351	0.480806	267.304535
38.440002	260.000000	38.451351	0.434082	266.990356
38.460003	251.667007	38.471348	0.404373	266.677307
38.480003	249.500000	38.491348	0.387339	266.365387
38.500000	250.500000	38.511349	0.380066	266.054749
38.520000	252.167007	38.531345	0.380628	265.745148
38.540001	249.667007	38.551346	0.387802	265.436737
38.560001	248.500000	38.571346	0.403283	265.129486
38.580002	247.667007	38.591343	0.421979	264.823364
38.599998	252.833008	38.611340	0.446180	264.518372
38.619999	252.833008	38.631340	0.476099	264.214600
38.639999	261.166992	38.651337	0.512172	263.911896
38.660000	244.333008	38.671337	0.555106	263.610382
38.680000	264.000000	38.691338	0.605859	263.309967
38.700001	268.666992	38.711338	0.665719	263.010620
38.720001	253.500000	38.731335	0.736356	262.712585
38.740002	258.500000	38.751335	0.819982	262.415588
38.760002	255.167007	38.771336	0.919425	262.119690

38.780003	258.000000	38.791332	1.038370	261.824951
38.799999	262.000000	38.811329	1.181720	261.531433
38.820000	257.000000	38.831329	1.356009	261.238922
38.840000	253.833008	38.851326	1.569924	260.947571
38.860001	260.832977	38.871330	1.835576	260.657318
38.880001	257.500000	38.891331	2.169471	260.368164
38.900002	253.500000	38.911327	2.595181	260.080200
38.920002	252.500000	38.931328	3.147241	259.793274
38.940002	260.332977	38.951328	3.877601	259.507568
38.960003	257.500000	38.971325	4.866396	259.222961
38.980003	259.166992	38.991325	6.246336	258.939392
39.000000	269.500000	39.011322	8.240109	258.656921
39.020000	260.666992	39.031319	11.233837	258.375702
39.040001	267.500000	39.051319	15.901293	258.095428
39.060001	275.500000	39.071320	23.414684	257.816284
39.080002	276.832977	39.091316	35.811634	257.538391
39.099998	316.000000	39.111313	56.634586	257.261444
39.119999	349.500000	39.131313	91.864662	256.985565
39.139999	410.832977	39.151310	150.077179	256.710815
39.160000	513.833008	39.171310	235.805679	256.437286
39.180000	623.666992	39.191311	322.149017	256.164581
39.200001	640.333008	39.211311	334.451233	255.893188
39.220001	553.000000	39.231312	253.045227	255.622742
39.240002	414.666992	39.251312	156.427734	255.353424
39.260002	355.332977	39.271313	91.625015	255.085205
39.280003	302.332977	39.291309	55.618771	254.818085
39.299999	296.666992	39.311306	36.166836	254.552063
39.320000	282.500000	39.331306	25.231226	254.287048
39.340000	272.166992	39.351303	18.701214	254.023102
39.360001	255.500000	39.371304	14.595810	253.760254
39.380001	255.500000	39.391304	11.957786	253.498474
39.400002	256.832977	39.411301	10.296765	253.237671
39.420002	264.000000	39.431301	9.341125	252.978058
39.440002	259.332977	39.451302	8.934365	252.719513
39.460003	256.666992	39.471298	8.990282	252.461884
39.480003	262.166992	39.491299	9.475684	252.205383
39.500000	260.500000	39.511295	10.403913	251.949982
39.520000	252.000000	39.531292	11.836909	251.695557

39.540001	264.332977	39.551292	13.895884	251.442200
39.560001	259.332977	39.571293	16.784222	251.189911
39.580002	268.500000	39.591290	20.834591	250.938568
39.599998	279.666992	39.611290	26.601540	250.688385
39.619999	279.332977	39.631290	35.022980	250.439148
39.639999	287.666992	39.651287	47.719337	250.191071
39.660000	311.332977	39.671288	67.528015	249.943848
39.680000	333.832977	39.691288	99.397942	249.697784
39.700001	377.166992	39.711285	151.963150	249.452698
39.720001	475.000000	39.731285	240.304413	249.208618
39.740002	633.166992	39.751286	389.976288	248.965546
39.760002	889.333008	39.771282	638.827942	248.723541
39.780003	1322.830078	39.791283	1014.117676	248.482605
39.799999	1742.330078	39.811279	1424.782104	248.242584
39.820000	1849.500000	39.831276	1553.270508	248.003571
39.840000	1558.169922	39.851276	1233.260376	247.765656
39.860001	1099.330078	39.871277	781.338501	247.528717
39.880001	728.333008	39.891273	457.636963	247.292694
39.900002	507.332977	39.911274	272.852936	247.057709
39.920002	396.166992	39.931274	171.970322	246.823761
39.940002	350.166992	39.951271	114.896889	246.590820
39.960003	314.000000	39.971272	80.420319	246.358826
39.980003	295.500000	39.991276	58.195316	246.127686
40.000000	278.666992	40.011269	43.152477	245.897766
40.020000	278.000000	40.031269	32.621464	245.668732
40.040001	274.666992	40.051270	25.107365	245.440643
40.060001	262.666992	40.071266	19.671600	245.213654
40.080002	278.666992	40.091267	15.692616	244.987579
40.099998	275.332977	40.111263	12.747114	244.762482
40.119999	248.333008	40.131264	10.547426	244.538208
40.139999	259.500000	40.151260	8.898356	244.315125
40.160000	256.332977	40.171261	7.667185	244.092865
40.180000	253.833008	40.191261	6.767715	243.871552
40.200001	246.333008	40.211258	6.147973	243.651245
40.220001	256.500000	40.231258	5.785303	243.431915
40.240002	254.333008	40.251259	5.688546	243.213501
40.260002	249.000000	40.271255	5.906998	242.996094
40.280003	249.500000	40.291256	6.552363	242.779510

40.299999	249.500000	40.311253	7.839161	242.564056
40.320000	246.667007	40.331249	10.158624	242.349457
40.340000	245.500000	40.351254	14.209496	242.135651
40.360001	267.666992	40.371254	21.214903	241.922882
40.380001	263.332977	40.391251	33.245491	241.711182
40.400002	284.332977	40.411251	53.311989	241.500244
40.420002	315.166992	40.431252	83.286690	241.290222
40.440002	333.166992	40.451248	115.199898	241.081238
40.460003	355.332977	40.471249	123.903404	240.873108
40.480003	338.832977	40.491249	97.924011	240.665955
40.500000	297.832977	40.511242	62.442631	240.459686
40.520000	272.500000	40.531242	37.083862	240.254272
40.540001	249.833008	40.551243	22.529623	240.049774
40.560001	254.833008	40.571239	14.522868	239.846313
40.580002	256.832977	40.591240	9.967915	239.643555
40.599998	242.167007	40.611237	7.228402	239.441833
40.619999	240.000000	40.631233	5.503623	239.241028
40.639999	246.333008	40.651234	4.409499	239.040985
40.660000	237.000000	40.671234	3.767838	238.841949
40.680000	234.000000	40.691231	3.516543	238.643768
40.700001	230.667007	40.711231	3.684777	238.446472
40.720001	242.500000	40.731236	4.405442	238.249969
40.740002	239.333008	40.751232	5.941168	238.054443
40.760002	245.167007	40.771233	8.636196	237.859741
40.780003	264.000000	40.791233	12.422472	237.665863
40.799999	268.166992	40.811226	15.505616	237.473053
40.820000	264.832977	40.831226	14.787299	237.280914
40.840000	258.666992	40.851227	10.735369	237.089722
40.860001	245.667007	40.871223	6.735527	236.899475
40.880001	232.000000	40.891224	4.155907	236.709900
40.900002	238.833008	40.911224	2.696795	236.521210
40.920002	247.333008	40.931221	1.871698	236.333557
40.940002	235.500000	40.951221	1.379297	236.146545
40.960003	238.667007	40.971222	1.065371	235.960510
40.980003	237.667007	40.991219	0.853394	235.775177
41.000000	248.000000	41.011215	0.704134	235.590759
41.020000	252.833008	41.031212	0.596065	235.407227
41.040001	256.500000	41.051212	0.516288	235.224487

41.060001	251.333008	41.071213	0.454946	235.042511
41.080002	260.666992	41.091209	0.409719	234.861481
41.099998	255.667007	41.111210	0.375138	234.681152
41.119999	245.333008	41.131210	0.348590	234.501740
41.139999	244.333008	41.151207	0.322982	234.323090
41.160000	234.833008	41.171207	0.307770	234.145264
41.180000	242.833008	41.191208	0.296624	233.968231
41.200001	235.667007	41.211205	0.288933	233.792023
41.220001	229.667007	41.231205	0.284278	233.616577
41.240002	230.167007	41.251205	0.282387	233.441925
41.260002	231.000000	41.271202	0.283097	233.268158
41.280003	223.667007	41.291203	0.286340	233.095184
41.299999	232.833008	41.311199	0.292125	232.922943
41.320000	232.667007	41.331196	0.300538	232.751495
41.340000	226.000000	41.351196	0.311738	232.580841
41.360001	228.833008	41.371197	0.325958	232.410919
41.380001	239.167007	41.391193	0.343512	232.241882
41.400002	224.333008	41.411194	0.364822	232.073456
41.420002	226.833008	41.431194	0.390415	231.905914
41.440002	227.333008	41.451191	0.420953	231.739136
41.460003	232.833008	41.471191	0.457288	231.573181
41.480003	239.000000	41.491196	0.500478	231.407898
41.500000	225.000000	41.511189	0.551818	231.243439
41.520000	226.833008	41.531189	0.613039	231.079620
41.540001	228.500000	41.551189	0.686272	230.916595
41.560001	237.000000	41.571186	0.774257	230.754395
41.580002	238.500000	41.591187	0.880604	230.592896
41.599998	237.167007	41.611183	1.009951	230.432159
41.619999	237.667007	41.631180	1.168507	230.272186
41.639999	251.500000	41.651180	1.364610	230.112823
41.660000	261.166992	41.671181	1.609513	229.954285
41.680000	265.500000	41.691177	1.918711	229.796478
41.700001	243.500000	41.711178	2.314174	229.639313
41.720001	255.667007	41.731178	2.827218	229.482971
41.740002	256.166992	41.751175	3.503891	229.327393
41.760002	248.167007	41.771175	4.414691	229.172363
41.780003	237.667007	41.791176	5.674126	229.018127
41.799999	242.833008	41.811169	7.449393	228.864685

41.820000	246.000000	41.831169	10.061907	228.711853
41.840000	250.833008	41.851170	14.032819	228.559753
41.860001	260.500000	41.871170	20.284328	228.408356
41.880001	262.166992	41.891171	30.439247	228.257568
41.900002	280.000000	41.911171	47.350216	228.107544
41.920002	302.000000	41.931168	75.927383	227.958282
41.940002	362.666992	41.951168	123.975716	227.809601
41.960003	461.332977	41.971165	200.308151	227.661682
41.980003	559.333008	41.991165	300.843811	227.514404
42.000000	642.666992	42.011162	376.681305	227.367828
42.020000	615.666992	42.031158	354.466064	227.221893
42.040001	515.000000	42.051159	253.537933	227.076691
42.060001	407.166992	42.071159	156.078613	226.932007
42.080002	331.832977	42.091156	93.451576	226.788147
42.099998	283.666992	42.111153	58.103157	226.644836
42.119999	269.000000	42.131153	38.340858	226.502258
42.139999	248.667007	42.151150	26.941597	226.360291
42.160000	240.500000	42.171150	20.219517	226.218994
42.180000	240.000000	42.191151	16.429335	226.078369
42.200001	240.333008	42.211147	14.898108	225.938354
42.220001	245.833008	42.231148	15.571438	225.799042
42.240002	231.833008	42.251148	18.995119	225.660248
42.260002	253.500000	42.271149	26.325254	225.522186
42.280003	277.166992	42.291149	38.875870	225.384705
42.299999	299.000000	42.311146	55.457699	225.247833
42.320000	308.000000	42.331142	67.032692	225.111694
42.340000	309.666992	42.351143	61.591110	224.976166
42.360001	273.832977	42.371143	43.731506	224.841125
42.380001	251.167007	42.391140	27.165707	224.706787
42.400002	240.000000	42.411140	16.574467	224.573059
42.420002	232.833008	42.431141	10.521641	224.439880
42.440002	223.000000	42.451138	7.049118	224.307434
42.460003	223.333008	42.471138	4.955072	224.175446
42.480003	226.167007	42.491135	3.617350	224.044189
42.500000	219.167007	42.511131	2.719151	223.913422
42.520000	220.167007	42.531132	2.093348	223.783325
42.540001	220.000000	42.551128	1.645748	223.653809
42.560001	218.833008	42.571129	1.318777	223.524811

42.580002	216.500000	42.591129	1.075597	223.396423
42.599998	220.833008	42.611122	0.891648	223.268677
42.619999	220.167007	42.631123	0.750058	223.141479
42.639999	217.667007	42.651127	0.639370	223.014832
42.660000	220.667007	42.671124	0.551628	222.888733
42.680000	224.000000	42.691124	0.481129	222.763275
42.700001	213.000000	42.711124	0.423567	222.638275
42.720001	225.833008	42.731121	0.376608	222.513916
42.740002	223.500000	42.751122	0.337772	222.390045
42.760002	225.833008	42.771122	0.305453	222.266785
42.780003	216.167007	42.791119	0.278429	222.144073
42.799999	224.167007	42.811115	0.255756	222.021973
42.820000	221.500000	42.831116	0.236702	221.900269
42.840000	221.333008	42.851112	0.220708	221.779266
42.860001	219.000000	42.871113	0.207321	221.658722
42.880001	218.167007	42.891109	0.196196	221.538696
42.900002	216.333008	42.911110	0.187052	221.419220
42.920002	214.833008	42.931110	0.179675	221.300293
42.940002	228.000000	42.951107	0.173896	221.181854
42.960003	221.833008	42.971107	0.169587	221.063995
42.980003	221.000000	42.991108	0.166656	220.946594
43.000000	223.833008	43.011101	0.165038	220.829803
43.020000	228.667007	43.031101	0.164699	220.713409
43.040001	228.333008	43.051105	0.165629	220.597534
43.060001	214.500000	43.071102	0.167841	220.482208
43.080002	222.167007	43.091103	0.171378	220.367401
43.099998	222.000000	43.111099	0.176304	220.253113
43.119999	212.000000	43.131096	0.182714	220.139282
43.139999	225.833008	43.151096	0.190736	220.025940
43.160000	218.667007	43.171097	0.200533	219.913055
43.180000	214.500000	43.191093	0.212307	219.800751
43.200001	220.500000	43.211094	0.229273	219.688812
43.220001	209.333008	43.231091	0.245927	219.577484
43.240002	219.333008	43.251091	0.265529	219.466583
43.260002	216.000000	43.271091	0.288558	219.356140
43.280003	228.833008	43.291088	0.315606	219.246216
43.299999	219.167007	43.311085	0.347417	219.136719
43.320000	220.000000	43.331085	0.384923	219.027649

43.340000	212.500000	43.351082	0.429272	218.919159
43.360001	219.000000	43.371082	0.481950	218.811035
43.380001	215.167007	43.391083	0.544822	218.703369
43.400002	221.833008	43.411079	0.620281	218.596252
43.420002	227.667007	43.431084	0.711500	218.489471
43.440002	222.000000	43.451084	0.822535	218.383179
43.460003	227.333008	43.471081	0.958816	218.277405
43.480003	225.333008	43.491081	1.127712	218.171967
43.500000	217.167007	43.511074	1.339079	218.067047
43.520000	233.333008	43.531075	1.606876	217.962524
43.540001	231.833008	43.551075	1.950479	217.858429
43.560001	227.000000	43.571072	2.397776	217.754730
43.580002	241.667007	43.591072	2.990222	217.651550
43.599998	227.500000	43.611069	3.790347	217.548737
43.619999	239.667007	43.631065	4.896947	217.446381
43.639999	232.000000	43.651066	6.471225	217.344391
43.660000	243.333008	43.671066	8.783934	217.242828
43.680000	228.333008	43.691063	12.302201	217.141693
43.700001	243.833008	43.711063	17.849129	217.040985
43.720001	239.833008	43.731064	26.884871	216.940674
43.740002	255.333008	43.751060	41.951054	216.840759
43.760002	282.000000	43.771061	67.412720	216.741241
43.780003	336.832977	43.791058	109.863426	216.642181
43.799999	404.666992	43.811054	175.741867	216.543427
43.820000	481.832977	43.831059	258.308563	216.445099
43.840000	539.833008	43.851055	314.197723	216.347137
43.860001	500.166992	43.871056	289.674133	216.249603
43.880001	430.166992	43.891056	206.894409	216.152466
43.900002	372.000000	43.911053	128.793747	216.055664
43.920002	307.332977	43.931053	78.050011	215.959259
43.940002	268.832977	43.951054	48.758057	215.863190
43.960003	244.167007	43.971050	31.900555	215.767548
43.980003	241.500000	43.991051	21.788895	215.672241
44.000000	230.167007	44.011047	15.421170	215.577362
44.020000	233.833008	44.031044	11.243686	215.482788
44.040001	226.333008	44.051044	8.419799	215.388550
44.060001	216.333008	44.071041	6.484847	215.294678
44.080002	216.500000	44.091042	5.147865	215.201263

44.099998	221.833008	44.111038	4.230798	215.108124
44.119999	220.667007	44.131035	3.621499	215.015320
44.139999	220.000000	44.151035	3.253361	214.922882
44.160000	211.167007	44.171036	3.094401	214.830750
44.180000	215.333008	44.191032	3.143753	214.739014
44.200001	220.500000	44.211033	3.436609	214.647614
44.220001	214.333008	44.231037	4.059791	214.556549
44.240002	214.167007	44.251034	5.183458	214.465790
44.260002	232.500000	44.271034	7.123696	214.375305
44.280003	219.500000	44.291031	10.445994	214.285278
44.299999	233.833008	44.311028	16.138849	214.195557
44.320000	235.333008	44.331028	25.778734	214.106110
44.340000	248.333008	44.351025	41.269024	214.016937
44.360001	270.000000	44.371025	62.603790	213.928101
44.380001	291.500000	44.391026	81.928123	213.839600
44.400002	292.000000	44.411022	83.464111	213.751495
44.420002	279.500000	44.431023	64.746315	213.663544
44.440002	252.333008	44.451023	41.965191	213.575989
44.460003	240.667007	44.471020	25.642523	213.488739
44.480003	232.500000	44.491020	15.923725	213.401764
44.500000	240.500000	44.511013	10.330118	213.315125
44.520000	223.000000	44.531013	7.008822	213.228699
44.540001	218.167007	44.551014	4.941096	213.142670
44.560001	217.000000	44.571011	3.596341	213.056885
44.580002	202.833008	44.591011	2.691791	212.971344
44.599998	214.500000	44.611008	2.068919	212.886139
44.619999	217.833008	44.631008	1.632337	212.801239
44.639999	218.500000	44.651009	1.322352	212.716583
44.660000	212.833008	44.671009	1.100147	212.632172
44.680000	208.833008	44.691006	0.940119	212.548126
44.700001	217.000000	44.711006	0.825123	212.464264
44.720001	220.667007	44.731003	0.743767	212.380676
44.740002	211.667007	44.751003	0.688366	212.297424
44.760002	211.500000	44.771004	0.653867	212.214355
44.780003	208.833008	44.791000	0.637033	212.131622
44.799999	204.667007	44.810997	0.635963	212.049133
44.820000	213.833008	44.830997	0.649843	211.966888
44.840000	215.000000	44.850994	0.678773	211.884888

44.860001	203.333008	44.870995	0.723765	211.803101
44.880001	212.833008	44.890995	0.786769	211.721649
44.900002	201.667007	44.910992	0.870842	211.640411
44.920002	212.667007	44.930992	0.980505	211.559357
44.940002	203.500000	44.950989	1.122077	211.478668
44.960003	208.333008	44.970989	1.304566	211.398132
44.980003	204.833008	44.990990	1.540590	211.317810
45.000000	204.167007	45.010983	1.848065	211.237762
45.020000	208.000000	45.030987	2.253499	211.157959
45.040001	205.667007	45.050987	2.795510	211.078369
45.060001	217.667007	45.070984	3.533317	210.998993
45.080002	210.167007	45.090984	4.560701	210.919891
45.099998	212.500000	45.110977	6.029590	210.841003
45.119999	210.667007	45.130978	8.198873	210.762390
45.139999	224.333008	45.150978	11.515410	210.683868
45.160000	218.333008	45.170975	16.767532	210.605591
45.180000	237.000000	45.190975	25.357382	210.527557
45.200001	242.833008	45.210976	39.735004	210.449738
45.220001	274.666992	45.230972	63.971073	210.372131
45.240002	317.666992	45.250973	103.861794	210.294769
45.260002	375.000000	45.270969	163.621536	210.217560
45.280003	467.332977	45.290970	232.891159	210.140503
45.299999	484.832977	45.310966	270.682861	210.063751
45.320000	459.832977	45.330963	239.778564	209.987183
45.340000	385.332977	45.350964	168.399460	209.910767
45.360001	328.832977	45.370964	105.251991	209.834503
45.380001	278.666992	45.390961	64.633896	209.758514
45.400002	250.667007	45.410961	40.902023	209.682678
45.420002	240.000000	45.430965	26.981339	209.607025
45.440002	231.667007	45.450962	18.483273	209.531586
45.460003	220.833008	45.470963	13.055628	209.456299
45.480003	221.000000	45.490959	9.461411	209.381226
45.500000	215.667007	45.510956	7.014721	209.306305
45.520000	210.333008	45.530956	5.313413	209.231567
45.540001	220.000000	45.550953	4.109504	209.157043
45.560001	219.833008	45.570953	3.243038	209.082581
45.580002	218.000000	45.590954	2.609689	209.008331
45.599998	194.833008	45.610947	2.140031	208.934296

45.619999	208.000000	45.630947	1.786864	208.860474
45.639999	203.500000	45.650944	1.518483	208.786743
45.660000	203.333008	45.670944	1.312691	208.713196
45.680000	203.333008	45.690945	1.154045	208.639709
45.700001	201.500000	45.710941	1.031537	208.566498
45.720001	186.833008	45.730942	0.937174	208.493469
45.740002	202.000000	45.750942	0.865195	208.420532
45.760002	205.333008	45.770939	0.811361	208.347717
45.780003	198.833008	45.790939	0.776182	208.275085
45.799999	198.167007	45.810932	0.750161	208.202606
45.820000	199.000000	45.830933	0.735155	208.130280
45.840000	208.833008	45.850937	0.729979	208.058044
45.860001	212.333008	45.870934	0.736577	207.986023
45.880001	208.167007	45.890934	0.749027	207.914093
45.900002	211.500000	45.910934	0.769778	207.842377
45.920002	199.667007	45.930931	0.798840	207.770721
45.940002	200.833008	45.950932	0.832713	207.699188
45.960003	209.500000	45.970928	0.879494	207.627777
45.980003	200.000000	45.990929	0.936035	207.556580
46.000000	211.833008	46.010925	1.003292	207.485474
46.020000	196.000000	46.030922	1.082550	207.414490
46.040001	202.000000	46.050922	1.175458	207.343658
46.060001	195.667007	46.070923	1.284056	207.272888
46.080002	204.667007	46.090919	1.410901	207.202271
46.099998	209.667007	46.110916	1.559260	207.131805
46.119999	194.333008	46.130913	1.733244	207.061371
46.139999	197.500000	46.150913	1.938144	206.991180
46.160000	199.333008	46.170914	2.180723	206.921021
46.180000	211.167007	46.190910	2.469877	206.850983
46.200001	203.333008	46.210911	2.817806	206.781067
46.220001	199.833008	46.230911	3.241303	206.711182
46.240002	203.667007	46.250908	3.764570	206.641479
46.260002	204.500000	46.270912	4.424378	206.571869
46.280003	207.333008	46.290909	5.276611	206.502319
46.299999	217.833008	46.310905	6.410441	206.432983
46.320000	213.500000	46.330906	7.965120	206.363678
46.340000	218.167007	46.350903	10.139730	206.294495
46.360001	215.667007	46.370903	13.143271	206.225403

46.380001	228.167007	46.390903	16.929388	206.156372
46.400002	235.000000	46.410900	20.751289	206.087494
46.420002	241.333008	46.430901	23.661482	206.018616
46.440002	246.000000	46.450897	26.262447	205.949921
46.460003	243.333008	46.470898	30.364885	205.881226
46.480003	251.167007	46.490898	37.335426	205.812653
46.500000	261.166992	46.510891	48.316505	205.744263
46.520000	294.000000	46.530891	65.169312	205.675842
46.540001	309.166992	46.550888	91.308762	205.607574
46.560001	344.500000	46.570889	132.968567	205.539307
46.580002	402.666992	46.590889	201.287460	205.471130
46.599998	510.832977	46.610882	315.633636	205.403137
46.619999	678.166992	46.630882	507.901001	205.335144
46.639999	1012.830017	46.650883	821.453796	205.267273
46.660000	1491.330078	46.670883	1282.985718	205.199371
46.680000	2016.169922	46.690884	1802.912109	205.131592
46.700001	2226.000000	46.710880	2072.701172	205.063904
46.720001	2024.000000	46.730881	1833.717529	204.996277
46.740002	1509.669922	46.750881	1298.947021	204.928711
46.760002	1007.169983	46.770878	821.733032	204.861206
46.780003	703.166992	46.790878	509.793121	204.793762
46.799999	523.833008	46.810875	324.726807	204.726379
46.820000	408.666992	46.830872	214.693710	204.659119
46.840000	355.332977	46.850872	146.790756	204.591888
46.860001	339.500000	46.870869	103.170830	204.524719
46.880001	303.832977	46.890869	74.204796	204.457550
46.900002	300.666992	46.910870	54.492279	204.390472
46.920002	286.666992	46.930866	40.808872	204.323486
46.940002	288.332977	46.950867	31.133375	204.256470
46.960003	296.332977	46.970863	24.172125	204.189545
46.980003	278.500000	46.990864	19.072697	204.122711
47.000000	260.000000	47.010860	15.273971	204.055908
47.020000	239.833008	47.030857	12.401120	203.989166
47.040001	239.333008	47.050858	10.189594	203.922394
47.060001	235.833008	47.070858	8.466424	203.855743
47.080002	220.333008	47.090858	7.107176	203.789124
47.099998	212.667007	47.110855	6.023283	203.722534
47.119999	227.000000	47.130852	5.150191	203.656006

47.139999	205.333008	47.150852	4.440566	203.589539
47.160000	207.333008	47.170853	3.859411	203.523071
47.180000	206.000000	47.190849	3.380306	203.456604
47.200001	205.500000	47.210850	2.982888	203.390198
47.220001	211.333008	47.230846	2.651788	203.323883
47.240002	207.833008	47.250847	2.374841	203.257507
47.260002	210.500000	47.270847	2.142709	203.191284
47.280003	197.000000	47.290844	1.948033	203.125000
47.299999	196.000000	47.310841	1.784938	203.058838
47.320000	196.667007	47.330841	1.648795	202.992615
47.340000	199.333008	47.350838	1.536025	202.926453
47.360001	193.333008	47.370838	1.443738	202.860352
47.380001	192.500000	47.390835	1.369773	202.794189
47.400002	203.000000	47.410835	1.312449	202.728119
47.420002	202.000000	47.430836	1.267695	202.662048
47.440002	201.667007	47.450832	1.240688	202.596008
47.460003	199.833008	47.470833	1.228066	202.530029
47.480003	193.333008	47.490829	1.229888	202.464081
47.500000	205.667007	47.510830	1.246619	202.398071
47.520000	199.333008	47.530830	1.279173	202.332092
47.540001	200.167007	47.550827	1.328957	202.266235
47.560001	193.167007	47.570827	1.398007	202.200287
47.580002	199.833008	47.590828	1.489065	202.134338
47.599998	195.333008	47.610821	1.605741	202.068542
47.619999	190.000000	47.630821	1.752957	202.002686
47.639999	194.000000	47.650818	1.936993	201.936798
47.660000	198.833008	47.670818	2.166304	201.870972
47.680000	186.667007	47.690819	2.451972	201.805084
47.700001	194.833008	47.710815	2.808773	201.739319
47.720001	202.167007	47.730816	3.256875	201.673492
47.740002	199.333008	47.750813	3.823449	201.607727
47.760002	203.667007	47.770813	4.546538	201.541931
47.780003	202.167007	47.790813	5.479162	201.476135
47.799999	197.333008	47.810806	6.696643	201.410400
47.820000	219.500000	47.830807	8.310387	201.344574
47.840000	212.000000	47.850803	10.484542	201.278809
47.860001	222.000000	47.870804	13.474092	201.213074
47.880001	231.500000	47.890804	17.684813	201.147278

47.900002	251.333008	47.910801	23.790112	201.081543
47.920002	265.332977	47.930801	32.953323	201.015808
47.940002	273.500000	47.950806	47.223915	200.950043
47.960003	297.832977	47.970802	70.230217	200.884216
47.980003	337.832977	47.990803	108.369507	200.818481
48.000000	389.166992	48.010796	172.316483	200.752747
48.020000	487.666992	48.030796	277.983368	200.686951
48.040001	666.500000	48.050797	441.063263	200.621216
48.060001	882.666992	48.070793	651.260193	200.555420
48.080002	1037.830078	48.090794	823.382751	200.489685
48.099998	1062.830078	48.110786	822.414673	200.423889
48.119999	898.166992	48.130787	643.732483	200.358093
48.139999	649.000000	48.150787	428.790771	200.292267
48.160000	468.166992	48.170784	269.648224	200.226501
48.180000	377.500000	48.190784	170.972443	200.160645
48.200001	304.500000	48.210781	112.013039	200.094879
48.220001	271.332977	48.230782	75.967674	200.029022
48.240002	245.333008	48.250782	53.114574	199.963135
48.260002	241.000000	48.270779	38.170952	199.897339
48.280003	244.167007	48.290779	28.189430	199.831482
48.299999	224.500000	48.310772	21.447721	199.765564
48.320000	230.667007	48.330772	16.879105	199.699738
48.340000	229.167007	48.350773	13.820693	199.633820
48.360001	246.500000	48.370773	11.860172	199.567902
48.380001	234.833008	48.390774	10.759739	199.501953
48.400002	227.833008	48.410770	10.415064	199.435974
48.420002	222.167007	48.430771	10.844942	199.369995
48.440002	221.833008	48.450771	12.215590	199.304047
48.460003	226.000000	48.470768	14.911842	199.238068
48.480003	226.833008	48.490768	19.685591	199.172089
48.500000	231.000000	48.510761	27.909082	199.106079
48.520000	239.833008	48.530762	41.989479	199.039978
48.540001	261.666992	48.550762	65.772156	198.973907
48.560001	314.000000	48.570759	104.207954	198.907837
48.580002	385.832977	48.590759	159.572083	198.841675
48.599998	448.832977	48.610756	219.869522	198.775574
48.619999	482.332977	48.630753	249.551605	198.709473
48.639999	458.666992	48.650753	221.264999	198.643250

48.660000	383.500000	48.670750	159.277573	198.577087
48.680000	310.166992	48.690750	102.971611	198.510895
48.700001	243.000000	48.710751	65.318924	198.444580
48.720001	215.833008	48.730747	42.509800	198.378357
48.740002	212.500000	48.750748	28.720448	198.312073
48.760002	193.500000	48.770744	20.121853	198.245789
48.780003	201.000000	48.790745	14.576703	198.179443
48.799999	207.500000	48.810745	10.914865	198.113098
48.820000	193.000000	48.830742	8.463152	198.046692
48.840000	197.167007	48.850742	6.811809	197.980286
48.860001	185.833008	48.870739	5.703506	197.913818
48.880001	197.667007	48.890739	4.972025	197.847382
48.900002	198.000000	48.910740	4.513522	197.780914
48.920002	192.167007	48.930737	4.254885	197.714417
48.940002	192.333008	48.950737	4.156425	197.647827
48.960003	188.833008	48.970734	4.195868	197.581238
48.980003	206.000000	48.990734	4.357188	197.514709
49.000000	189.833008	49.010731	4.640547	197.448029
49.020000	192.000000	49.030727	5.052751	197.381378
49.040001	200.667007	49.050728	5.609299	197.314758
49.060001	200.667007	49.070724	6.334944	197.247986
49.080002	205.667007	49.090725	7.266668	197.181274
49.099998	205.667007	49.110722	8.456109	197.114502
49.119999	199.667007	49.130718	9.976232	197.047729
49.139999	201.167007	49.150719	11.929539	196.980896
49.160000	217.833008	49.170715	14.459548	196.914062
49.180000	214.000000	49.190716	17.773670	196.847137
49.200001	217.667007	49.210716	22.172007	196.780212
49.220001	230.000000	49.230713	28.100868	196.713318
49.240002	248.500000	49.250717	36.251862	196.646301
49.260002	251.000000	49.270714	47.715733	196.579285
49.280003	261.832977	49.290714	64.322639	196.512207
49.299999	295.000000	49.310711	89.212418	196.445129
49.320000	334.832977	49.330708	127.944290	196.378021
49.340000	380.666992	49.350708	190.403732	196.310913
49.360001	468.332977	49.370705	293.802734	196.243713
49.380001	653.166992	49.390705	466.720184	196.176575
49.400002	913.000000	49.410706	750.374084	196.109314

49.420002	1378.500000	49.430702	1182.924316	196.042053
49.440002	1913.669922	49.450703	1732.147217	195.974792
49.460003	2328.830078	49.470699	2176.285645	195.907379
49.480003	2310.170166	49.490700	2177.972168	195.840027
49.500000	1916.669922	49.510696	1723.354858	195.772614
49.520000	1372.830078	49.530693	1163.947998	195.705261
49.540001	951.833008	49.550694	740.314575	195.637756
49.560001	664.166992	49.570690	472.869720	195.570251
49.580002	502.166992	49.590691	310.670166	195.502747
49.599998	395.000000	49.610687	210.373718	195.435181
49.619999	349.666992	49.630684	146.144852	195.367584
49.639999	328.166992	49.650684	103.742355	195.299927
49.660000	286.666992	49.670681	75.131844	195.232300
49.680000	265.000000	49.690685	55.484398	195.164581
49.700001	254.000000	49.710686	41.788616	195.096832
49.720001	247.333008	49.730682	32.078621	195.029083
49.740002	226.167007	49.750683	25.082001	194.961243
49.760002	227.167007	49.770679	19.950647	194.893463
49.780003	227.667007	49.790680	16.122890	194.825562
49.799999	218.500000	49.810673	13.226059	194.757690
49.820000	218.667007	49.830673	11.003620	194.689789
49.840000	212.667007	49.850674	9.281254	194.621765
49.860001	208.833008	49.870670	7.936016	194.553772
49.880001	211.500000	49.890671	6.880233	194.485779
49.900002	200.500000	49.910667	6.050095	194.417725
49.920002	200.500000	49.930668	5.399943	194.349609
49.940002	202.500000	49.950668	4.896612	194.281494
49.960003	209.333008	49.970665	4.516109	194.213318
49.980003	194.000000	49.990665	4.241368	194.145081
50.000000	203.000000	50.010658	4.061295	194.076904
50.020000	198.500000	50.030659	3.969344	194.008667
50.040001	187.167007	50.050659	3.963562	193.940338
50.060001	194.667007	50.070656	4.046203	193.872009
50.080002	180.333008	50.090656	4.224202	193.803650
50.099998	190.000000	50.110649	4.509735	193.735229
50.119999	186.667007	50.130653	4.922138	193.666870
50.139999	193.167007	50.150654	5.489013	193.598389
50.160000	206.500000	50.170650	6.250107	193.529907

50.180000	200.333008	50.190651	7.262386	193.461365
50.200001	202.167007	50.210648	8.606304	193.392822
50.220001	205.333008	50.230648	10.398858	193.324249
50.240002	208.000000	50.250648	12.810306	193.255615
50.260002	228.333008	50.270645	16.095388	193.186951
50.280003	209.000000	50.290646	20.650959	193.118286
50.299999	230.167007	50.310638	27.111738	193.049622
50.320000	240.333008	50.330639	36.556053	192.980896
50.340000	248.167007	50.350636	50.845261	192.912109
50.360001	270.832977	50.370636	73.305359	192.843323
50.380001	299.500000	50.390636	109.797958	192.774475
50.400002	361.000000	50.410633	170.401001	192.705627
50.420002	459.000000	50.430634	271.253143	192.636719
50.440002	635.000000	50.450630	433.702148	192.567810
50.460003	832.666992	50.470631	671.470642	192.498901
50.480003	1135.169922	50.490631	948.223572	192.429871
50.500000	1301.330078	50.510624	1128.599731	192.360931
50.520000	1265.669922	50.530624	1066.369873	192.291870
50.540001	1014.669983	50.550621	811.488892	192.222839
50.560001	753.166992	50.570625	541.223938	192.153717
50.580002	543.166992	50.590626	346.277985	192.084595
50.599998	394.832977	50.610619	224.041290	192.015442
50.619999	311.500000	50.630619	149.068253	191.946289
50.639999	269.832977	50.650616	101.993156	191.877075
50.660000	254.167007	50.670616	71.447441	191.807861
50.680000	238.500000	50.690617	51.111298	191.738586
50.700001	229.667007	50.710613	37.327435	191.669342
50.720001	220.000000	50.730614	27.851980	191.600006
50.740002	217.500000	50.750610	21.258074	191.530701
50.760002	214.833008	50.770611	16.608171	191.461334
50.780003	203.333008	50.790607	13.289379	191.391968
50.799999	192.500000	50.810604	10.896432	191.322571
50.820000	192.667007	50.830605	9.163134	191.253113
50.840000	203.667007	50.850601	7.912970	191.183716
50.860001	192.333008	50.870602	7.034376	191.114197
50.880001	189.000000	50.890598	6.451491	191.044678
50.900002	194.000000	50.910599	6.117867	190.975159
50.920002	197.667007	50.930599	6.010530	190.905640

50.940002	187.833008	50.950596	6.124933	190.835999
50.960003	191.500000	50.970596	6.475104	190.766479
50.980003	189.500000	50.990593	7.095556	190.696899
51.000000	189.500000	51.010590	8.046958	190.627197
51.020000	190.500000	51.030590	9.426023	190.557617
51.040001	201.333008	51.050591	11.382219	190.487885
51.060001	203.000000	51.070591	14.149643	190.418152
51.080002	209.500000	51.090588	18.104393	190.348480
51.099998	216.500000	51.110584	23.877184	190.278748
51.119999	218.333008	51.130581	32.559910	190.209015
51.139999	233.667007	51.150581	46.088791	190.139221
51.160000	269.166992	51.170582	67.893250	190.069397
51.180000	302.332977	51.190578	103.915718	189.999634
51.200001	360.332977	51.210579	163.896240	189.929779
51.220001	462.666992	51.230576	261.589325	189.859924
51.240002	625.500000	51.250576	409.488220	189.790100
51.260002	829.666992	51.270576	597.131165	189.720154
51.280003	978.666992	51.290573	754.177673	189.650299
51.299999	1014.169983	51.310570	769.674255	189.580383
51.320000	882.166992	51.330566	626.834351	189.510468
51.340000	662.833008	51.350567	434.788849	189.440521
51.360001	501.666992	51.370564	281.799408	189.370605
51.380001	385.166992	51.390564	182.111267	189.300598
51.400002	307.666992	51.410564	120.539101	189.230621
51.420002	276.332977	51.430561	82.027046	189.160645
51.440002	244.167007	51.450562	57.174076	189.090576
51.460003	241.833008	51.470558	40.700581	189.020630
51.480003	225.000000	51.490562	29.568398	188.950562
51.500000	213.667007	51.510559	21.952589	188.880493
51.520000	202.000000	51.530556	16.682037	188.810455
51.540001	205.000000	51.550556	12.993445	188.740417
51.560001	195.667007	51.570553	10.385044	188.670288
51.580002	200.167007	51.590553	8.524258	188.600220
51.599998	203.000000	51.610546	7.194317	188.530151
51.619999	194.667007	51.630547	6.251582	188.460022
51.639999	193.333008	51.650547	5.603667	188.389954
51.660000	195.167007	51.670544	5.191139	188.319794
51.680000	194.500000	51.690544	4.977929	188.249603

51.700001	190.667007	51.710541	4.946055	188.179504
51.720001	188.500000	51.730541	5.092473	188.109375
51.740002	193.500000	51.750538	5.428550	188.039246
51.760002	193.167007	51.770538	5.981710	187.969116
51.780003	193.667007	51.790539	6.798733	187.898926
51.799999	200.167007	51.810532	7.952083	187.828796
51.820000	191.833008	51.830532	9.552992	187.758606
51.840000	203.667007	51.850529	11.767778	187.688477
51.860001	210.167007	51.870529	14.856103	187.618256
51.880001	200.167007	51.890530	19.233282	187.548065
51.900002	215.000000	51.910526	25.593996	187.477905
51.920002	215.333008	51.930527	35.148689	187.407715
51.940002	234.333008	51.950527	50.031536	187.337524
51.960003	250.000000	51.970528	74.031387	187.267334
51.980003	287.500000	51.990524	113.639290	187.197174
52.000000	345.000000	52.010521	179.312164	187.126984
52.020000	470.500000	52.030521	285.384125	187.056824
52.040001	640.833008	52.050518	443.336182	186.986633
52.060001	843.833008	52.070518	638.432556	186.916443
52.080002	996.333008	52.090515	794.686768	186.846313
52.099998	1014.330017	52.110512	804.156189	186.776123
52.119999	869.833008	52.130508	657.609192	186.705933
52.139999	692.166992	52.150509	461.721954	186.635803
52.160000	502.666992	52.170509	302.572266	186.565613
52.180000	392.166992	52.190506	196.833099	186.495483
52.200001	305.166992	52.210506	130.652786	186.425293
52.220001	266.000000	52.230503	88.944244	186.355164
52.240002	247.000000	52.250504	61.897419	186.285004
52.260002	232.667007	52.270504	43.898762	186.214844
52.280003	213.667007	52.290501	31.700377	186.144714
52.299999	192.833008	52.310497	23.320923	186.074646
52.320000	207.833008	52.330494	17.495213	186.004578
52.340000	191.167007	52.350494	13.389720	185.934448
52.360001	196.833008	52.370491	10.452773	185.864380
52.380001	192.333008	52.390491	8.315851	185.794312
52.400002	182.500000	52.410496	6.730737	185.724182
52.420002	183.667007	52.430492	5.545564	185.654114
52.440002	184.333008	52.450493	4.645294	185.584106

52.460003	191.000000	52.470490	3.955633	185.514038
52.480003	183.333008	52.490490	3.424489	185.444000
52.500000	189.333008	52.510483	3.015624	185.374023
52.520000	184.333008	52.530483	2.702722	185.304016
52.540001	173.833008	52.550484	2.467350	185.234009
52.560001	183.167007	52.570480	2.296289	185.164062
52.580002	177.500000	52.590481	2.180237	185.094116
52.599998	180.333008	52.610474	2.113137	185.024231
52.619999	182.333008	52.630474	2.091382	184.954285
52.639999	185.333008	52.650471	2.113674	184.884399
52.660000	178.333008	52.670471	2.180801	184.814514
52.680000	180.333008	52.690472	2.295698	184.744629
52.700001	181.167007	52.710468	2.463650	184.674805
52.720001	182.333008	52.730469	2.692868	184.604919
52.740002	187.833008	52.750465	2.994947	184.535126
52.760002	191.333008	52.770466	3.386300	184.465393
52.780003	185.667007	52.790462	3.889257	184.395599
52.799999	183.667007	52.810459	4.534868	184.325867
52.820000	192.833008	52.830460	5.366199	184.256104
52.840000	196.167007	52.850456	6.442789	184.186401
52.860001	196.500000	52.870457	7.849539	184.116699
52.880001	208.333008	52.890457	9.707433	184.047058
52.900002	202.833008	52.910458	12.194173	183.977386
52.920002	228.167007	52.930454	15.577925	183.907806
52.940002	245.000000	52.950455	20.283281	183.838196
52.960003	252.833008	52.970455	26.998810	183.768616
52.980003	266.000000	52.990452	36.919235	183.699158
53.000000	294.166992	53.010448	52.095432	183.629639
53.020000	321.332977	53.030445	76.086990	183.560181
53.040001	354.332977	53.050446	114.854103	183.490692
53.060001	409.666992	53.070442	177.566315	183.421265
53.080002	516.666992	53.090443	276.422852	183.351868
53.099998	663.833008	53.110439	423.510712	183.282532
53.119999	825.166992	53.130436	622.115479	183.213196
53.139999	966.666992	53.150436	841.192078	183.143860
53.160000	1051.169922	53.170433	981.117249	183.074585
53.180000	984.166992	53.190434	932.978088	183.005341
53.200001	877.500000	53.210430	727.517883	182.936157

53.220001	718.666992	53.230431	499.000061	182.866943
53.240002	558.833008	53.250431	326.611237	182.797791
53.260002	416.332977	53.270428	214.700241	182.728699
53.280003	334.332977	53.290428	144.304443	182.659607
53.299999	283.332977	53.310421	99.242912	182.590576
53.320000	266.666992	53.330421	69.547203	182.521545
53.340000	244.667007	53.350418	49.547958	182.452576
53.360001	230.333008	53.370422	35.868134	182.383636
53.380001	214.000000	53.390423	26.414145	182.314728
53.400002	212.000000	53.410419	19.806984	182.245850
53.420002	214.167007	53.430420	15.124836	182.177063
53.440002	203.833008	53.450417	11.754059	182.108276
53.460003	205.000000	53.470417	9.282784	182.039459
53.480003	198.167007	53.490414	7.438704	181.970825
53.500000	208.833008	53.510410	6.038616	181.902100
53.520000	201.333008	53.530411	4.958809	181.833496
53.540001	214.833008	53.550407	4.114742	181.764893
53.560001	203.167007	53.570408	3.446579	181.696320
53.580002	201.667007	53.590405	2.911972	181.627808
53.599998	206.667007	53.610401	2.479916	181.559326
53.619999	202.833008	53.630398	2.127607	181.490967
53.639999	207.333008	53.650398	1.837937	181.422546
53.660000	210.333008	53.670395	1.596024	181.354248
53.680000	184.833008	53.690395	1.396114	181.285950
53.700001	183.333008	53.710396	1.228481	181.217651
53.720001	182.833008	53.730392	1.084673	181.149506
53.740002	180.667007	53.750393	0.965093	181.081299
53.760002	174.667007	53.770390	0.863496	181.013184
53.780003	176.167007	53.790390	0.776911	180.945129
53.799999	171.667007	53.810383	0.703045	180.877136
53.820000	187.000000	53.830383	0.639977	180.809113
53.840000	169.667007	53.850388	0.586238	180.741211
53.860001	166.667007	53.870384	0.540681	180.673309
53.880001	169.667007	53.890385	0.502360	180.605530
53.900002	171.500000	53.910381	0.470599	180.537720
53.920002	170.667007	53.930382	0.444896	180.470032
53.940002	170.333008	53.950378	0.424963	180.402344
53.960003	164.667007	53.970379	0.410684	180.334717

53.980003	169.167007	53.990379	0.402158	180.267120
54.000000	159.333008	54.010372	0.399710	180.199585
54.020000	175.000000	54.030373	0.403942	180.132111
54.040001	168.333008	54.050369	0.415844	180.064758
54.060001	180.667007	54.070370	0.436756	179.997345
54.080002	175.000000	54.090366	0.468739	179.930054
54.099998	172.667007	54.110363	0.514683	179.862823
54.119999	175.667007	54.130360	0.578679	179.795624
54.139999	169.167007	54.150360	0.666563	179.728455
54.160000	182.000000	54.170361	0.786702	179.661377
54.180000	180.667007	54.190357	0.951383	179.594391
54.200001	170.667007	54.210358	1.179443	179.527344
54.220001	178.167007	54.230354	1.500735	179.460449
54.240002	175.500000	54.250355	1.965479	179.393555
54.260002	182.333008	54.270351	2.660479	179.326782
54.280003	176.000000	54.290352	3.739789	179.260040
54.299999	183.333008	54.310349	5.473194	179.193359
54.320000	188.833008	54.330349	8.321159	179.126678
54.340000	192.833008	54.350349	13.011230	179.060120
54.360001	213.833008	54.370346	20.519316	178.993652
54.380001	215.167007	54.390347	31.634117	178.927185
54.400002	239.833008	54.410343	45.447205	178.860809
54.420002	239.667007	54.430344	57.095951	178.794434
54.440002	244.000000	54.450340	59.085041	178.728210
54.460003	223.333008	54.470341	49.674839	178.661987
54.480003	223.333008	54.490341	35.735989	178.595856
54.500000	211.500000	54.510334	23.871056	178.529755
54.520000	186.833008	54.530334	15.760044	178.463745
54.540001	186.000000	54.550331	10.584688	178.397797
54.560001	181.500000	54.570332	7.269619	178.331879
54.580002	170.000000	54.590328	5.091246	178.266083
54.599998	169.667007	54.610325	3.624944	178.200348
54.619999	176.167007	54.630322	2.622439	178.134644
54.639999	180.333008	54.650322	1.930115	178.069031
54.660000	176.167007	54.670322	1.447994	178.003479
54.680000	170.333008	54.690319	1.108781	177.938019
54.700001	167.000000	54.710320	0.867984	177.872528
54.720001	160.833008	54.730316	0.689485	177.807190

54.740002	168.167007	54.750317	0.560311	177.741882
54.760002	167.667007	54.770313	0.463567	177.676697
54.780003	164.500000	54.790314	0.390029	177.611481
54.799999	169.333008	54.810307	0.333490	177.546417
54.820000	173.000000	54.830311	0.289577	177.481415
54.840000	165.333008	54.850311	0.255282	177.416443
54.860001	167.500000	54.870308	0.228416	177.351562
54.880001	163.833008	54.890308	0.207379	177.286774
54.900002	165.833008	54.910305	0.191007	177.221985
54.920002	173.000000	54.930305	0.178426	177.157349
54.940002	172.000000	54.950302	0.168992	177.092773
54.960003	172.500000	54.970303	0.162221	177.028259
54.980003	165.167007	54.990299	0.157757	176.963806
55.000000	168.500000	55.010296	0.155341	176.899414
55.020000	164.833008	55.030296	0.154793	176.835114
55.040001	175.000000	55.050293	0.155995	176.770874
55.060001	178.500000	55.070293	0.158888	176.706726
55.080002	165.167007	55.090290	0.163455	176.642578
55.099998	166.000000	55.110287	0.169731	176.578644
55.119999	165.833008	55.130283	0.177791	176.514648
55.139999	164.833008	55.150284	0.187758	176.450836
55.160000	166.500000	55.170280	0.199799	176.387024
55.180000	163.500000	55.190281	0.214143	176.323364
55.200001	172.500000	55.210281	0.231071	176.259644
55.220001	160.000000	55.230278	0.250939	176.196136
55.240002	164.000000	55.250278	0.274198	176.132599
55.260002	168.833008	55.270275	0.301391	176.069214
55.280003	165.167007	55.290276	0.333208	176.005890
55.299999	160.333008	55.310268	0.370474	175.942627
55.320000	168.000000	55.330273	0.414275	175.879456
55.340000	166.167007	55.350269	0.465881	175.816345
55.360001	161.000000	55.370270	0.526877	175.753296
55.380001	164.167007	55.390270	0.599291	175.690338
55.400002	168.833008	55.410267	0.686125	175.627502
55.420002	167.667007	55.430267	0.790587	175.564697
55.440002	170.167007	55.450264	0.917049	175.502014
55.460003	162.167007	55.470264	1.071333	175.439362
55.480003	171.667007	55.490261	1.261008	175.376770

55.500000	169.833008	55.510258	1.496269	175.314331
55.520000	171.167007	55.530254	1.790822	175.251923
55.540001	161.333008	55.550255	2.163386	175.189636
55.560001	172.833008	55.570255	2.639521	175.127319
55.580002	168.000000	55.590252	3.254624	175.065186
55.599998	163.333008	55.610249	4.058757	175.003143
55.619999	169.833008	55.630245	5.124384	174.941193
55.639999	174.167007	55.650246	6.558053	174.879303
55.660000	173.000000	55.670242	8.527204	174.817444
55.680000	170.167007	55.690243	11.308013	174.755707
55.700001	188.333008	55.710239	15.372070	174.694031
55.720001	180.167007	55.730240	21.552315	174.632446
55.740002	186.000000	55.750240	31.310690	174.570892
55.760002	215.167007	55.770237	47.153152	174.509521
55.780003	244.500000	55.790237	73.150200	174.448151
55.799999	275.166992	55.810230	115.113968	174.386932
55.820000	342.500000	55.830231	179.206406	174.325745
55.840000	423.332977	55.850231	265.441772	174.264648
55.860001	521.000000	55.870232	354.255280	174.203644
55.880001	567.666992	55.890228	400.750305	174.142700
55.900002	544.000000	55.910229	370.645996	174.081909
55.920002	462.166992	55.930225	286.479004	174.021118
55.940002	367.166992	55.950226	198.421768	173.960480
55.960003	294.666992	55.970226	132.339188	173.899902
55.980003	261.500000	55.990223	88.720154	173.839355
56.000000	225.667007	56.010220	60.626751	173.778931
56.020000	207.000000	56.030216	42.209572	173.718628
56.040001	192.667007	56.050217	29.832443	173.658386
56.060001	180.500000	56.070213	21.370005	173.598206
56.080002	200.333008	56.090214	15.525300	173.538177
56.099998	182.000000	56.110207	11.467695	173.478210
56.119999	185.333008	56.130207	8.633493	173.418274
56.139999	191.833008	56.150204	6.642386	173.358459
56.160000	177.500000	56.170204	5.235225	173.298737
56.180000	179.167007	56.190205	4.242701	173.239075
56.200001	173.500000	56.210201	3.560076	173.179504
56.220001	178.000000	56.230202	3.132687	173.120026
56.240002	165.667007	56.250198	2.950286	173.060638

56.260002	169.333008	56.270199	3.045295	173.001312
56.280003	168.667007	56.290195	3.488325	172.942108
56.299999	178.333008	56.310192	4.353060	172.882996
56.320000	174.333008	56.330189	5.596050	172.824005
56.340000	177.667007	56.350193	6.836810	172.764954
56.360001	179.000000	56.370190	7.326561	172.706116
56.380001	170.500000	56.390190	6.607533	172.647278
56.400002	173.667007	56.410191	5.136925	172.588623
56.420002	171.000000	56.430187	3.692489	172.530060
56.440002	174.333008	56.450188	2.624990	172.471497
56.460003	171.167007	56.470184	1.916799	172.413025
56.480003	170.000000	56.490185	1.455430	172.354736
56.500000	166.000000	56.510178	1.151142	172.296509
56.520000	169.500000	56.530178	0.948369	172.238251
56.540001	164.167007	56.550175	0.814846	172.180206
56.560001	170.500000	56.570175	0.730951	172.122162
56.580002	172.167007	56.590172	0.684454	172.064270
56.599998	175.500000	56.610168	0.667687	172.006439
56.619999	174.000000	56.630169	0.676317	171.948700
56.639999	169.167007	56.650166	0.708665	171.891052
56.660000	165.333008	56.670166	0.765475	171.833496
56.680000	165.500000	56.690163	0.849986	171.776031
56.700001	162.000000	56.710163	0.968686	171.718658
56.720001	169.167007	56.730160	1.132704	171.661346
56.740002	164.000000	56.750160	1.360901	171.604126
56.760002	169.833008	56.770157	1.684236	171.546997
56.780003	164.333008	56.790157	2.155485	171.489929
56.799999	171.833008	56.810150	2.856670	171.433014
56.820000	167.833008	56.830151	3.910375	171.376160
56.840000	180.667007	56.850151	5.465888	171.319366
56.860001	174.000000	56.870152	7.619818	171.262665
56.880001	179.833008	56.890152	10.207323	171.206055
56.900002	180.500000	56.910149	12.597834	171.149536
56.920002	183.667007	56.930149	14.086945	171.093079
56.940002	181.833008	56.950146	14.933439	171.036804
56.960003	179.000000	56.970146	16.472549	170.980530
56.980003	179.167007	56.990143	20.192093	170.924377
57.000000	192.667007	57.010139	27.560825	170.868317

57.020000	196.333008	57.030136	40.602314	170.812286
57.040001	217.333008	57.050137	62.303150	170.756409
57.060001	267.332977	57.070137	96.084900	170.700592
57.080002	321.000000	57.090134	142.937622	170.644836
57.099998	395.500000	57.110130	194.761887	170.589233
57.119999	421.000000	57.130127	229.097473	170.533691
57.139999	405.332977	57.150127	222.844467	170.478180
57.160000	367.666992	57.170124	180.378128	170.422821
57.180000	315.666992	57.190125	128.762344	170.367554
57.200001	270.666992	57.210121	87.124374	170.312378
57.220001	229.667007	57.230122	58.706600	170.257202
57.240002	200.333008	57.250118	40.194408	170.202179
57.260002	197.333008	57.270119	28.015339	170.147247
57.280003	196.000000	57.290115	19.813257	170.092377
57.299999	177.000000	57.310112	14.183860	170.037567
57.320000	183.333008	57.330112	10.277102	169.982941
57.340000	188.667007	57.350109	7.552406	169.928284
57.360001	177.667007	57.370110	5.642011	169.873779
57.380001	179.167007	57.390106	4.293158	169.819336
57.400002	176.167007	57.410110	3.329768	169.765015
57.420002	177.500000	57.430107	2.632550	169.710785
57.440002	181.500000	57.450108	2.119876	169.656555
57.460003	177.667007	57.470104	1.737484	169.602417
57.480003	175.333008	57.490105	1.448640	169.548462
57.500000	173.667007	57.510098	1.228786	169.494568
57.520000	178.667007	57.530098	1.060656	169.440735
57.540001	169.500000	57.550095	0.932652	169.386993
57.560001	173.833008	57.570095	0.836506	169.333282
57.580002	167.333008	57.590096	0.766543	169.279724
57.599998	161.333008	57.610088	0.718936	169.226196
57.619999	169.667007	57.630089	0.691271	169.172760
57.639999	167.500000	57.650085	0.682421	169.119476
57.660000	165.667007	57.670086	0.692371	169.066223
57.680000	168.000000	57.690083	0.722269	169.013123
57.700001	172.000000	57.710083	0.774576	168.959961
57.720001	166.167007	57.730080	0.853297	168.906982
57.740002	166.333008	57.750080	0.964502	168.854095
57.760002	155.000000	57.770077	1.116889	168.801300

57.780003	169.833008	57.790077	1.323045	168.748474
57.799999	163.500000	57.810070	1.600956	168.695862
57.820000	168.000000	57.830070	1.978659	168.643250
57.840000	165.333008	57.850067	2.496474	168.590729
57.860001	160.000000	57.870068	3.224422	168.538300
57.880001	165.333008	57.890068	4.280475	168.485962
57.900002	174.167007	57.910065	5.870671	168.433746
57.920002	179.000000	57.930069	8.356469	168.381531
57.940002	174.667007	57.950066	12.348534	168.329468
57.960003	188.500000	57.970066	18.832575	168.277466
57.980003	200.000000	57.990063	29.214903	168.225586
58.000000	219.667007	58.010059	45.028252	168.173706
58.020000	245.667007	58.030056	66.605057	168.121887
58.040001	253.500000	58.050056	90.212593	168.070221
58.060001	285.666992	58.070053	106.159035	168.018616
58.080002	276.166992	58.090054	105.046196	167.967102
58.099998	267.332977	58.110046	88.841766	167.915619
58.119999	243.000000	58.130047	68.000397	167.864227
58.139999	217.833008	58.150043	49.771004	167.812988
58.160000	204.167007	58.170044	36.152187	167.761749
58.180000	192.167007	58.190044	27.114042	167.710602
58.200001	184.000000	58.210041	22.096796	167.659576
58.220001	186.000000	58.230042	20.613245	167.608582
58.240002	196.667007	58.250038	22.534863	167.557678
58.260002	198.333008	58.270039	27.854630	167.506805
58.280003	202.500000	58.290035	35.770050	167.456055
58.299999	221.000000	58.310032	43.328773	167.405396
58.320000	228.000000	58.330029	45.724720	167.354767
58.340000	216.667007	58.350029	40.586166	167.304260
58.360001	197.833008	58.370026	31.075533	167.253784
58.380001	195.333008	58.390026	21.884605	167.203430
58.400002	176.500000	58.410023	15.074641	167.153168
58.420002	176.833008	58.430023	10.518661	167.102905
58.440002	173.833008	58.450020	7.528565	167.052795
58.460003	166.833008	58.470020	5.541337	167.002716
58.480003	166.167007	58.490025	4.212423	166.952698
58.500000	173.000000	58.510017	3.335714	166.902740
58.520000	159.500000	58.530018	2.787553	166.852936

58.540001	164.667007	58.550014	2.495068	166.803101
58.560001	161.167007	58.570015	2.420616	166.753387
58.580002	166.333008	58.590012	2.560653	166.703796
58.599998	164.333008	58.610008	2.953264	166.654175
58.619999	164.167007	58.630005	3.697275	166.604736
58.639999	163.667007	58.650005	4.986440	166.555298
58.660000	171.833008	58.670002	7.158662	166.505920
58.680000	170.833008	58.690002	10.752364	166.456696
58.700001	171.333008	58.709999	16.500393	166.407471
58.720001	175.000000	58.730000	25.093605	166.358276
58.740002	204.833008	58.749996	36.328651	166.309235
58.760002	206.667007	58.769997	47.587048	166.260254
58.780003	217.000000	58.789993	53.453053	166.211334
58.799999	219.833008	58.809990	49.930714	166.162445
58.820000	204.167007	58.829987	39.500896	166.113647
58.840000	186.667007	58.849987	28.112415	166.064880
58.860001	180.167007	58.869987	19.214951	166.016266
58.880001	164.333008	58.889984	13.147676	165.967651
58.900002	159.000000	58.909985	9.140808	165.919128
58.920002	164.667007	58.929981	6.460958	165.870667
58.940002	162.500000	58.949982	4.627573	165.822235
58.960003	168.000000	58.969978	3.353740	165.773895
58.980003	165.500000	58.989979	2.462206	165.725677
59.000000	162.833008	59.009972	1.835922	165.677490
59.020000	157.833008	59.029976	1.395318	165.629303
59.040001	156.167007	59.049973	1.083212	165.581238
59.060001	159.500000	59.069973	0.860586	165.533203
59.080002	152.000000	59.089970	0.700225	165.485321
59.099998	154.000000	59.109966	0.582854	165.437439
59.119999	158.167007	59.129963	0.496264	165.389618
59.139999	155.500000	59.149963	0.431974	165.341797
59.160000	170.667007	59.169960	0.384275	165.294128
59.180000	165.000000	59.189960	0.349221	165.246490
59.200001	152.667007	59.209957	0.324113	165.198914
59.220001	167.167007	59.229958	0.307050	165.151398
59.240002	154.833008	59.249958	0.296725	165.103973
59.260002	162.167007	59.269955	0.292248	165.056580
59.280003	162.667007	59.289955	0.293046	165.009216

59.299999	155.500000	59.309948	0.298758	164.961975
59.320000	158.833008	59.329948	0.309379	164.914764
59.340000	162.167007	59.349945	0.324954	164.867615
59.360001	165.500000	59.369946	0.345774	164.820496
59.380001	163.333008	59.389942	0.372314	164.773438
59.400002	177.667007	59.409943	0.405284	164.726440
59.420002	178.833008	59.429939	0.445626	164.679504
59.440002	178.000000	59.449940	0.494615	164.632629
59.460003	176.000000	59.469936	0.553873	164.585815
59.480003	179.000000	59.489937	0.625550	164.539062
59.500000	183.000000	59.509930	0.712359	164.492340
59.520000	181.000000	59.529930	0.817953	164.445679
59.540001	185.500000	59.549927	0.946969	164.399078
59.560001	185.833008	59.569927	1.105633	164.352539
59.580002	178.000000	59.589928	1.301564	164.306000
59.599998	183.333008	59.609924	1.546149	164.259583
59.619999	186.333008	59.629921	1.854749	164.213226
59.639999	171.167007	59.649921	2.246603	164.166870
59.660000	170.500000	59.669922	2.748579	164.120575
59.680000	185.167007	59.689919	3.397655	164.074341
59.700001	176.333008	59.709919	4.246531	164.028137
59.720001	183.667007	59.729916	5.372016	163.981995
59.740002	170.667007	59.749916	6.894201	163.935913
59.760002	178.667007	59.769913	9.008616	163.889862
59.780003	186.500000	59.789913	12.051704	163.843872
59.799999	185.333008	59.809906	16.607203	163.797974
59.820000	192.833008	59.829906	23.697386	163.752045
59.840000	199.667007	59.849903	35.039646	163.706146
59.860001	219.833008	59.869904	53.382179	163.660400
59.880001	233.667007	59.889900	82.632278	163.614624
59.900002	292.666992	59.909901	127.133492	163.568909
59.920002	343.666992	59.929897	188.299164	163.523224
59.940002	410.832977	59.949898	257.213776	163.477600
59.960003	464.332977	59.969894	308.028656	163.432007
59.980003	458.000000	59.989895	310.646240	163.386505
60.000000	427.666992	60.009888	263.194244	163.341003
60.020000	353.166992	60.029888	195.806229	163.295563
60.040001	307.832977	60.049885	136.556808	163.250153

60.060001	264.166992	60.069885	94.029144	163.204773
60.080002	219.833008	60.089882	65.594048	163.159454
60.099998	200.167007	60.109879	46.665848	163.114136
60.119999	180.833008	60.129875	33.865513	163.068909
60.139999	188.500000	60.149876	25.117886	163.023712
60.160000	195.333008	60.169880	19.177197	162.978516
60.180000	183.333008	60.189877	15.265303	162.933411
60.200001	191.833008	60.209877	12.876971	162.888306
60.220001	168.167007	60.229874	11.707491	162.843262
60.240002	169.167007	60.249874	11.624454	162.798218
60.260002	176.667007	60.269871	12.687875	162.753296
60.280003	176.500000	60.289871	15.211952	162.708313
60.299999	186.167007	60.309864	19.878719	162.663422
60.320000	190.833008	60.329865	27.921228	162.618561
60.340000	204.333008	60.349861	41.296204	162.573730
60.360001	232.333008	60.369862	62.687130	162.528931
60.380001	257.166992	60.389858	94.650558	162.484161
60.400002	308.666992	60.409859	136.809708	162.439423
60.420002	346.000000	60.429855	180.530624	162.394745
60.440002	380.000000	60.449856	206.830215	162.350037
60.460003	361.666992	60.469852	199.108246	162.305420
60.480003	327.500000	60.489853	162.622665	162.260803
60.500000	282.000000	60.509846	118.691406	162.216217
60.520000	249.667007	60.529846	82.433945	162.171661
60.540001	219.500000	60.549843	56.978245	162.127167
60.560001	195.000000	60.569843	39.955608	162.082672
60.580002	185.000000	60.589840	28.547861	162.038208
60.599998	169.667007	60.609837	20.806671	161.993805
60.619999	175.500000	60.629833	15.569086	161.949432
60.639999	169.667007	60.649834	12.136215	161.905029
60.660000	170.500000	60.669830	10.029369	161.860718
60.680000	168.500000	60.689831	8.796778	161.816345
60.700001	165.500000	60.709827	7.907577	161.772064
60.720001	155.500000	60.729828	6.881423	161.727814
60.740002	154.833008	60.749828	5.609851	161.683533
60.760002	167.667007	60.769829	4.340957	161.639343
60.780003	151.500000	60.789825	3.304887	161.595123
60.799999	155.167007	60.809822	2.545847	161.550964

60.820000	161.000000	60.829823	2.006110	161.506836
60.840000	173.500000	60.849819	1.619582	161.462677
60.860001	163.000000	60.869820	1.337875	161.418579
60.880001	161.833008	60.889816	1.130137	161.374481
60.900002	168.667007	60.909817	0.976397	161.330414
60.920002	163.667007	60.929813	0.863343	161.286377
60.940002	173.000000	60.949814	0.781558	161.242340
60.960003	167.667007	60.969810	0.724390	161.198334
60.980003	166.833008	60.989811	0.687087	161.154358
61.000000	159.833008	61.009804	0.667582	161.110382
61.020000	169.333008	61.029804	0.661519	161.066437
61.040001	161.833008	61.049801	0.668892	161.022522
61.060001	162.000000	61.069801	0.689341	160.978607
61.080002	161.667007	61.089798	0.723168	160.934692
61.099998	166.167007	61.109795	0.771335	160.890778
61.119999	162.667007	61.129791	0.835475	160.846924
61.139999	167.667007	61.149792	0.918003	160.803101
61.160000	171.667007	61.169788	1.022208	160.759216
61.180000	167.333008	61.189789	1.152607	160.715393
61.200001	167.667007	61.209785	1.315124	160.671600
61.220001	170.833008	61.229786	1.517768	160.627747
61.240002	156.833008	61.249783	1.771031	160.583954
61.260002	158.000000	61.269783	2.089822	160.540222
61.280003	161.833008	61.289780	2.491470	160.496429
61.299999	160.833008	61.309776	3.002045	160.452667
61.320000	173.167007	61.329777	3.656075	160.408936
61.340000	172.000000	61.349777	4.501031	160.365204
61.360001	172.333008	61.369774	5.604485	160.321442
61.380001	180.833008	61.389774	7.067904	160.277740
61.400002	173.333008	61.409771	9.049314	160.234009
61.420002	181.000000	61.429771	11.810556	160.190277
61.440002	185.000000	61.449768	15.793847	160.146606
61.460003	197.500000	61.469769	21.760517	160.102905
61.480003	198.000000	61.489765	30.991243	160.059204
61.500000	216.833008	61.509762	45.567608	160.015533
61.520000	241.333008	61.529758	68.610397	159.971893
61.540001	266.166992	61.549759	104.130257	159.928223
61.560001	328.000000	61.569756	155.554459	159.884521

61.580002	390.666992	61.589756	221.940125	159.840881
61.599998	470.332977	61.609749	293.059021	159.797211
61.619999	520.333008	61.629749	351.266876	159.753540
61.639999	560.666992	61.649746	382.377747	159.709900
61.660000	555.500000	61.669746	379.254211	159.666260
61.680000	531.000000	61.689743	338.357574	159.622589
61.700001	446.000000	61.709743	269.918518	159.578918
61.720001	386.166992	61.729740	197.697708	159.535309
61.740002	318.666992	61.749741	138.983353	159.491608
61.760002	274.000000	61.769737	97.140839	159.447937
61.780003	240.333008	61.789738	68.556763	159.404327
61.799999	209.833008	61.809731	48.976627	159.360657
61.820000	205.333008	61.829731	35.331039	159.317017
61.840000	186.833008	61.849728	25.700003	159.273315
61.860001	187.833008	61.869728	18.854855	159.229675
61.880001	171.333008	61.889729	13.979489	159.185974
61.900002	183.167007	61.909725	10.501559	159.142334
61.920002	172.833008	61.929726	8.009554	159.098633
61.940002	165.000000	61.949726	6.210483	159.054932
61.960003	169.500000	61.969727	4.896549	159.011230
61.980003	169.333008	61.989723	3.923173	158.967529
62.000000	166.333008	62.009720	3.190530	158.923859
62.020000	156.167007	62.029716	2.630495	158.880127
62.040001	156.333008	62.049717	2.196250	158.836395
62.060001	160.167007	62.069714	1.855555	158.792694
62.080002	158.500000	62.089714	1.586476	158.748962
62.099998	161.167007	62.109707	1.370822	158.705170
62.119999	157.667007	62.129707	1.197495	158.661438
62.139999	157.833008	62.149704	1.057786	158.617676
62.160000	158.667007	62.169704	0.945024	158.573883
62.180000	159.500000	62.189701	0.854248	158.530121
62.200001	155.833008	62.209702	0.781611	158.486298
62.220001	165.667007	62.229698	0.724241	158.442505
62.240002	159.333008	62.249699	0.679912	158.398682
62.260002	157.167007	62.269695	0.646988	158.354858
62.280003	153.000000	62.289696	0.624252	158.311005
62.299999	153.833008	62.309689	0.610876	158.267151
62.320000	158.167007	62.329689	0.606334	158.223236

62.340000	151.667007	62.349686	0.610606	158.179382
62.360001	157.167007	62.369686	0.623336	158.135468
62.380001	155.167007	62.389683	0.645035	158.091553
62.400002	154.333008	62.409683	0.676318	158.047638
62.420002	157.667007	62.429680	0.718100	158.003632
62.440002	158.833008	62.449680	0.771694	157.959686
62.460003	155.000000	62.469677	0.838820	157.915710
62.480003	167.500000	62.489677	0.921733	157.871704
62.500000	152.500000	62.509670	1.023474	157.827637
62.520000	166.000000	62.529671	1.147917	157.783630
62.540001	164.000000	62.549671	1.300028	157.739563
62.560001	172.167007	62.569672	1.486599	157.695465
62.580002	156.667007	62.589668	1.715531	157.651367
62.599998	161.500000	62.609665	1.998291	157.607239
62.619999	169.333008	62.629662	2.349601	157.563141
62.639999	164.500000	62.649662	2.789034	157.518951
62.660000	159.500000	62.669659	3.341190	157.474792
62.680000	160.333008	62.689659	4.043011	157.430542
62.700001	161.000000	62.709656	4.939481	157.386322
62.720001	170.833008	62.729656	6.094518	157.342072
62.740002	167.667007	62.749653	7.597783	157.297821
62.760002	172.000000	62.769653	9.584183	157.253479
62.780003	176.667007	62.789650	12.265329	157.209198
62.799999	173.833008	62.809647	15.995367	157.164856
62.820000	186.167007	62.829643	21.383297	157.120483
62.840000	196.500000	62.849644	29.487192	157.076080
62.860001	211.333008	62.869640	42.106476	157.031677
62.880001	222.167007	62.889641	62.203930	156.987213
62.900002	256.500000	62.909637	94.295929	156.942749
62.920002	294.332977	62.929638	144.469971	156.898224
62.940002	395.832977	62.949635	218.680511	156.853729
62.960003	476.332977	62.969635	317.263306	156.809143
62.980003	604.500000	62.989632	424.166840	156.764557
63.000000	678.500000	63.009628	500.264557	156.719971
63.020000	692.000000	63.029625	503.502106	156.675323
63.040001	624.166992	63.049625	432.038788	156.630646
63.060001	520.333008	63.069622	328.410034	156.585968
63.080002	423.500000	63.089622	234.165344	156.541229

63.099998	335.832977	63.109615	164.272354	156.496490
63.119999	300.166992	63.129616	116.168373	156.451691
63.139999	241.667007	63.149612	83.349258	156.406891
63.160000	212.667007	63.169613	60.589069	156.362030
63.180000	214.500000	63.189613	44.578854	156.317139
63.200001	190.833008	63.209614	33.283855	156.272217
63.220001	184.667007	63.229610	25.404369	156.227295
63.240002	188.833008	63.249611	20.066994	156.182312
63.260002	188.500000	63.269608	16.698711	156.137268
63.280003	187.500000	63.289608	14.972833	156.092255
63.299999	182.333008	63.309601	14.818081	156.047180
63.320000	189.667007	63.329601	16.450771	156.002075
63.340000	187.667007	63.349598	20.421177	155.956909
63.360001	195.667007	63.369598	27.611778	155.911743
63.380001	210.667007	63.389595	39.001610	155.866516
63.400002	218.667007	63.409592	54.864624	155.821259
63.420002	249.667007	63.429592	73.083481	155.776001
63.440002	260.332977	63.449589	87.620438	155.730652
63.460003	255.667007	63.469589	90.842171	155.685272
63.480003	252.500000	63.489586	80.650589	155.639893
63.500000	236.667007	63.509583	63.244232	155.594482
63.520000	211.500000	63.529579	46.348087	155.549042
63.540001	196.333008	63.549580	33.469776	155.503510
63.560001	193.000000	63.569576	24.612000	155.457977
63.580002	176.000000	63.589577	18.698896	155.412354
63.599998	171.333008	63.609570	14.786385	155.366760
63.619999	187.333008	63.629570	12.262177	155.321106
63.639999	175.167007	63.649567	10.770524	155.275421
63.660000	170.833008	63.669567	10.106307	155.229675
63.680000	175.667007	63.689564	10.160157	155.183899
63.700001	162.667007	63.709564	10.899637	155.138092
63.720001	178.167007	63.729561	12.371426	155.092255
63.740002	178.667007	63.749561	14.731122	155.046356
63.760002	180.333008	63.769558	18.302000	155.000397
63.780003	188.333008	63.789558	23.691252	154.954407
63.799999	198.167007	63.809551	31.969707	154.908417
63.820000	211.500000	63.829556	44.974358	154.862335
63.840000	235.500000	63.849552	65.638077	154.816254

63.860001	260.832977	63.869553	98.331627	154.770081
63.880001	320.000000	63.889549	148.556992	154.723907
63.900002	405.832977	63.909550	220.942535	154.677673
63.920002	502.000000	63.929546	313.331696	154.631409
63.940002	584.000000	63.949547	407.521027	154.585114
63.960003	612.666992	63.969543	466.903870	154.538757
63.980003	613.666992	63.989544	460.125122	154.492371
64.000000	562.500000	64.009537	395.491364	154.445923
64.019997	503.500000	64.029533	314.033417	154.399414
64.040001	421.332977	64.049538	250.510864	154.352905
64.059998	390.000000	64.069527	218.183640	154.306335
64.080002	376.832977	64.089531	218.480438	154.259735
64.099998	411.666992	64.109528	250.095642	154.213043
64.119995	458.166992	64.129524	308.073486	154.166321
64.139999	519.333008	64.149521	376.096649	154.119598
64.159996	562.833008	64.169518	422.067932	154.072815
64.180000	567.500000	64.189522	414.361084	154.025940
64.199997	515.500000	64.209518	352.745758	153.979065
64.219994	430.166992	64.229507	269.046753	153.932190
64.239998	357.332977	64.249512	193.583527	153.885162
64.259995	318.000000	64.269508	137.276794	153.838165
64.279999	268.832977	64.289513	98.076164	153.791046
64.300003	248.000000	64.309509	70.977180	153.743958
64.320000	223.500000	64.329506	51.907722	153.696808
64.340004	197.500000	64.349510	38.250053	153.649567
64.360001	192.667007	64.369507	28.388731	153.602295
64.380005	188.167007	64.389503	21.253525	153.555023
64.400002	177.000000	64.409500	16.100721	153.507660
64.419998	165.333008	64.429497	12.386587	153.460266
64.440002	168.833008	64.449501	9.707674	153.412811
64.459999	167.833008	64.469490	7.771394	153.365295
64.480003	171.667007	64.489494	6.362576	153.317749
64.500000	176.333008	64.509491	5.334058	153.270172
64.519997	165.167007	64.529480	4.582791	153.222534
64.540001	167.667007	64.549484	4.038521	153.174835
64.559998	164.833008	64.569481	3.655691	153.127136
64.580002	158.333008	64.589485	3.404001	153.079285
64.599998	158.333008	64.609474	3.265386	153.031525

64.619995	165.333008	64.629471	3.230249	152.983643
64.639999	157.333008	64.649475	3.296709	152.935699
64.659996	163.167007	64.669472	3.469523	152.887726
64.680000	159.667007	64.689468	3.760475	152.839691
64.699997	172.167007	64.709465	4.189173	152.791626
64.719994	172.833008	64.729462	4.784692	152.743500
64.739998	167.667007	64.749466	5.588836	152.695312
64.759995	171.667007	64.769455	6.659646	152.647095
64.779999	173.167007	64.789459	8.085618	152.598816
64.800003	173.333008	64.809471	10.001005	152.550476
64.820000	176.000000	64.829468	12.621584	152.502075
64.840004	173.000000	64.849464	16.318323	152.453644
64.860001	184.167007	64.869461	21.729137	152.405182
64.880005	195.833008	64.889465	29.952347	152.356598
64.900002	202.000000	64.909462	42.809750	152.308044
64.919998	227.833008	64.929451	63.197285	152.259460
64.940002	244.000000	64.949455	95.380173	152.210785
64.959999	302.832977	64.969452	144.579147	152.162048
64.980003	376.832977	64.989456	215.031860	152.113251
65.000000	477.000000	65.009445	304.209564	152.064453
65.019997	578.333008	65.029442	394.464783	152.015594
65.040001	600.000000	65.049446	450.844421	151.966614
65.059998	606.500000	65.069443	442.743408	151.917664
65.080002	549.333008	65.089439	375.953186	151.868652
65.099998	483.666992	65.109436	287.527649	151.819580
65.119995	378.666992	65.129433	209.542023	151.770447
65.139999	342.500000	65.149429	152.843857	151.721283
65.159996	288.500000	65.169426	115.519829	151.672058
65.180000	266.832977	65.189430	93.045105	151.622742
65.199997	238.000000	65.209427	82.152946	151.573425
65.219994	244.500000	65.229416	80.709488	151.524078
65.239998	240.833008	65.249420	86.136780	151.474609
65.259995	250.167007	65.269417	93.489594	151.425171
65.279999	238.667007	65.289421	95.591713	151.375580
65.300003	243.833008	65.309418	87.549950	151.326019
65.320000	227.000000	65.329414	71.469681	151.276367
65.340004	198.667007	65.349419	53.769909	151.226685
65.360001	195.500000	65.369415	39.025856	151.176941

65.380005	177.000000	65.389412	28.246325	151.127167
65.400002	177.500000	65.409409	20.680820	151.077332
65.419998	158.500000	65.429405	15.341858	151.027435
65.440002	165.667007	65.449409	11.500198	150.977509
65.459999	150.667007	65.469398	8.695646	150.927551
65.480003	160.833008	65.489403	6.629052	150.877472
65.500000	154.333008	65.509399	5.104649	150.827362
65.519997	148.333008	65.529396	3.979091	150.777252
65.540001	157.667007	65.549393	3.146432	150.727051
65.559998	152.500000	65.569389	2.527282	150.676849
65.580002	156.833008	65.589394	2.062670	150.626526
65.599998	144.833008	65.609390	1.710147	150.576202
65.619995	152.667007	65.629379	1.439108	150.525879
65.639999	152.833008	65.649384	1.227515	150.475403
65.659996	156.167007	65.669380	1.060578	150.424927
65.680000	140.167007	65.689377	0.927547	150.374390
65.699997	147.833008	65.709373	0.820861	150.323792
65.719994	144.667007	65.729370	0.735092	150.273193
65.739998	153.000000	65.749374	0.666278	150.222473
65.759995	145.667007	65.769363	0.611610	150.171783
65.779999	147.333008	65.789368	0.568902	150.120941
65.800003	155.667007	65.809372	0.536695	150.070129
65.820000	155.167007	65.829369	0.513962	150.019257
65.840004	151.667007	65.849365	0.500058	149.968323
65.860001	151.167007	65.869362	0.494709	149.917358
65.880005	156.167007	65.889366	0.497985	149.866302
65.900002	151.000000	65.909363	0.510304	149.815247
65.919998	148.333008	65.929352	0.532460	149.764160
65.940002	153.167007	65.949356	0.565741	149.712952
65.959999	146.833008	65.969353	0.611917	149.661743
65.980003	152.500000	65.989357	0.673513	149.610474
66.000000	154.000000	66.009346	0.752536	149.559174
66.019997	150.667007	66.029343	0.856112	149.507812
66.040001	148.667007	66.049347	0.988899	149.456390
66.059998	145.167007	66.069344	1.158619	149.404938
66.080002	152.167007	66.089340	1.375645	149.353455
66.099998	155.500000	66.109337	1.653801	149.301910
66.119995	160.500000	66.129333	2.012050	149.250336

66.139999	153.833008	66.149330	2.477632	149.198700
66.159996	163.500000	66.169327	3.091703	149.146973
66.180000	164.500000	66.189339	3.922564	149.095215
66.199997	160.000000	66.209335	5.081411	149.043427
66.219994	159.333008	66.229324	6.761063	148.991638
66.239998	159.667007	66.249329	9.293199	148.939789
66.259995	175.500000	66.269325	13.221958	148.887878
66.279999	170.167007	66.289330	19.414789	148.835876
66.300003	186.000000	66.309326	29.125753	148.783844
66.320000	202.167007	66.329323	43.932446	148.731812
66.340004	215.500000	66.349327	65.199066	148.679749
66.360001	257.000000	66.369324	92.586723	148.627594
66.380005	279.666992	66.389320	121.759628	148.575378
66.400002	327.832977	66.409317	143.305237	148.523193
66.419998	322.166992	66.429314	147.159119	148.470886
66.440002	309.166992	66.449318	131.352005	148.418610
66.459999	267.666992	66.469307	104.433357	148.366241
66.480003	239.167007	66.489311	77.238243	148.313843
66.500000	209.000000	66.509308	55.521812	148.261414
66.519997	196.667007	66.529297	39.884594	148.208893
66.540001	175.000000	66.549301	28.909954	148.156403
66.559998	174.167007	66.569298	21.142147	148.103851
66.580002	164.833008	66.589302	15.543748	148.051208
66.599998	155.167007	66.609291	11.467916	147.998566
66.619995	152.333008	66.629288	8.488379	147.945892
66.639999	154.333008	66.649292	6.315794	147.893127
66.659996	149.000000	66.669289	4.738810	147.840363
66.680000	137.667007	66.689285	3.595900	147.787567
66.699997	139.167007	66.709282	2.766067	147.734711
66.719994	139.667007	66.729279	2.159678	147.681824
66.739998	143.833008	66.749283	1.711659	147.628876
66.759995	136.500000	66.769272	1.376405	147.575897
66.779999	148.667007	66.789276	1.121631	147.522888
66.800003	146.667007	66.809280	0.924558	147.469849
66.820000	140.167007	66.829269	0.770219	147.416779
66.840004	138.167007	66.849274	0.647582	147.363617
66.860001	144.000000	66.869270	0.549000	147.310486
66.880005	152.667007	66.889275	0.469189	147.257263

66.900002	147.500000	66.909264	0.404024	147.204041
66.919998	139.500000	66.929260	0.350401	147.150757
66.940002	142.667007	66.949265	0.306052	147.097443
66.959999	143.500000	66.969261	0.268137	147.044128
66.980003	141.500000	66.989258	0.237612	146.990753
67.000000	136.167007	67.009254	0.212297	146.937317
67.019997	135.500000	67.029251	0.191432	146.883881
67.040001	140.167007	67.049255	0.174461	146.830383
67.059998	138.500000	67.069244	0.161048	146.776855
67.080002	140.000000	67.089249	0.151027	146.723297
67.099998	129.333008	67.109245	0.143570	146.669739
67.119995	137.000000	67.129242	0.141170	146.616119
67.139999	138.833008	67.149239	0.144005	146.562439
67.159996	147.167007	67.169235	0.154293	146.508789
67.180000	139.333008	67.189240	0.175883	146.455048
67.199997	141.833008	67.209229	0.214926	146.401306
67.219994	140.833008	67.229225	0.280566	146.347534
67.239998	144.833008	67.249229	0.384448	146.293701
67.259995	148.167007	67.269226	0.536749	146.239868
67.279999	136.500000	67.289223	0.736347	146.185974
67.300003	144.500000	67.309227	0.952919	146.132080
67.320000	140.833008	67.329224	1.116154	146.078125
67.340004	152.333008	67.349228	1.146912	146.024170
67.360001	148.000000	67.369217	1.027260	145.970184
67.380005	151.333008	67.389221	0.822751	145.916168
67.400002	148.167007	67.409218	0.617343	145.862122
67.419998	147.833008	67.429214	0.453652	145.808044
67.440002	148.000000	67.449211	0.335463	145.753937
67.459999	150.500000	67.469208	0.252041	145.699799
67.480003	148.333008	67.489212	0.192435	145.645660
67.500000	145.167007	67.509201	0.149127	145.591522
67.519997	142.833008	67.529198	0.117286	145.537292
67.540001	145.333008	67.549202	0.093846	145.483063
67.559998	151.167007	67.569199	0.076691	145.428802
67.580002	131.333008	67.589195	0.064234	145.374512
67.599998	145.333008	67.609192	0.055277	145.320251
67.619995	146.333008	67.629189	0.048911	145.265930
67.639999	144.500000	67.649200	0.044456	145.211548

67.659996	138.000000	67.669189	0.041431	145.157166
67.680000	138.833008	67.689194	0.039492	145.102753
67.699997	135.833008	67.709190	0.038398	145.048340
67.719994	135.333008	67.729179	0.038003	144.993927
67.739998	139.167007	67.749184	0.038214	144.939453
67.759995	137.500000	67.769180	0.038982	144.884979
67.779999	138.167007	67.789185	0.040091	144.830505
67.800003	145.833008	67.809181	0.041960	144.775970
67.820000	136.833008	67.829178	0.044416	144.721405
67.840004	148.833008	67.849182	0.047520	144.666870
67.860001	140.000000	67.869179	0.051354	144.612305
67.880005	136.167007	67.889175	0.056034	144.557709
67.900002	144.667007	67.909172	0.061740	144.503113
67.919998	138.000000	67.929169	0.068602	144.448456
67.940002	136.833008	67.949173	0.076903	144.393860
67.959999	143.500000	67.969162	0.086950	144.339233
67.980003	138.833008	67.989166	0.099171	144.284515
68.000000	142.333008	68.009163	0.114082	144.229858
68.019997	142.000000	68.029152	0.132363	144.175171
68.040001	137.333008	68.049156	0.154922	144.120453
68.059998	137.833008	68.069153	0.181636	144.065735
68.080002	137.167007	68.089157	0.216512	144.010956
68.099998	140.667007	68.109146	0.260148	143.956268
68.119995	139.167007	68.129143	0.315109	143.901489
68.139999	143.333008	68.149147	0.384837	143.846741
68.159996	141.667007	68.169144	0.474176	143.791962
68.180000	137.000000	68.189140	0.590587	143.737183
68.199997	135.833008	68.209137	0.746063	143.682373
68.219994	147.000000	68.229134	0.960761	143.627563
68.239998	127.667000	68.249138	1.269250	143.572693
68.259995	132.667007	68.269127	1.729487	143.517914
68.279999	131.333008	68.289131	2.438572	143.463074
68.300003	138.000000	68.309135	3.548109	143.408203
68.320000	134.500000	68.329124	5.278293	143.353394
68.340004	135.333008	68.349129	7.914963	143.298523
68.360001	147.167007	68.369125	11.721942	143.243652
68.380005	149.833008	68.389130	16.725281	143.188782
68.400002	157.167007	68.409119	22.292692	143.133942

68.419998	165.000000	68.429115	26.844990	143.079071
68.440002	155.000000	68.449120	28.378246	143.024200
68.459999	157.333008	68.469116	26.088024	142.969330
68.480003	152.000000	68.489113	21.270344	142.914459
68.500000	150.667007	68.509109	16.040699	142.859589
68.519997	142.833008	68.529106	11.700616	142.804688
68.540001	145.000000	68.549103	8.510097	142.749847
68.559998	146.000000	68.569099	6.249070	142.694946
68.580002	142.167007	68.589104	4.637094	142.640045
68.599998	139.667007	68.609100	3.469450	142.585175
68.619995	140.667007	68.629089	2.614470	142.530304
68.639999	136.000000	68.649094	1.987768	142.475433
68.659996	136.000000	68.669090	1.533431	142.420593
68.680000	139.333008	68.689095	1.209795	142.365692
68.699997	136.167007	68.709084	0.986086	142.310852
68.719994	138.500000	68.729080	0.838607	142.255981
68.739998	134.000000	68.749084	0.750732	142.201141
68.759995	133.667007	68.769073	0.711429	142.146301
68.779999	142.500000	68.789078	0.715098	142.091461
68.800003	130.500000	68.809082	0.761899	142.036591
68.820000	138.000000	68.829079	0.858961	141.981781
68.840004	129.333008	68.849075	1.023541	141.926941
68.860001	133.333008	68.869072	1.288330	141.872131
68.880005	140.167007	68.889076	1.709887	141.817322
68.900002	130.667007	68.909073	2.378727	141.762573
68.919998	143.667007	68.929062	3.429886	141.707794
68.940002	140.833008	68.949066	5.043911	141.653015
68.959999	138.667007	68.969063	7.404324	141.598236
68.980003	139.833008	68.989059	10.582464	141.543518
69.000000	141.333008	69.009056	14.299465	141.488770
69.019997	144.000000	69.029053	17.693232	141.434052
69.040001	147.833008	69.049057	19.507629	141.379333
69.059998	148.333008	69.069046	18.994062	141.324646
69.080002	148.667007	69.089050	16.670298	141.270020
69.099998	148.500000	69.109047	13.761006	141.215363
69.119995	150.667007	69.129044	11.080952	141.160675
69.139999	142.500000	69.149040	8.793454	141.106049
69.159996	151.500000	69.169037	6.845350	141.051453

69.180000	138.667007	69.189041	5.238717	140.996857
69.199997	142.167007	69.209038	3.994118	140.942291
69.219994	141.000000	69.229034	3.079612	140.887726
69.239998	144.667007	69.249039	2.429765	140.833160
69.259995	137.500000	69.269035	1.981177	140.778687
69.279999	147.667007	69.289032	1.684973	140.724213
69.300003	135.667007	69.309036	1.507447	140.669739
69.320000	133.500000	69.329033	1.426883	140.615234
69.340004	135.833008	69.349037	1.429852	140.560822
69.360001	137.833008	69.369026	1.509805	140.506439
69.380005	135.000000	69.389030	1.666518	140.452026
69.400002	136.167007	69.409027	1.905677	140.397705
69.419998	140.500000	69.429016	2.240599	140.343384
69.440002	142.500000	69.449020	2.695803	140.289093
69.459999	142.833008	69.469017	3.312188	140.234833
69.480003	139.333008	69.489021	4.161322	140.180542
69.500000	133.333008	69.509010	5.364579	140.126373
69.519997	137.500000	69.529007	7.132726	140.072205
69.540001	140.333008	69.549011	9.817167	140.018036
69.559998	139.833008	69.569008	13.980021	139.963898
69.580002	156.167007	69.589005	20.477560	139.909790
69.599998	161.500000	69.609001	30.489462	139.855713
69.619995	170.833008	69.628998	45.372501	139.801697
69.639999	189.167007	69.648994	66.061127	139.747681
69.659996	209.333008	69.668991	91.686859	139.693695
69.680000	249.833008	69.688995	117.666786	139.639771
69.699997	260.832977	69.708992	135.311111	139.585876
69.719994	269.832977	69.728981	136.415710	139.532013
69.739998	256.332977	69.748985	120.581039	139.478149
69.759995	229.333008	69.768982	96.010155	139.424377
69.779999	202.167007	69.788986	71.869171	139.370605
69.800003	183.000000	69.808983	52.639011	139.316895
69.820000	176.833008	69.828979	38.632526	139.263184
69.840004	159.667007	69.848984	28.636812	139.209564
69.860001	155.667007	69.868973	21.435307	139.155945
69.880005	149.667007	69.888977	16.162178	139.102356
69.900002	149.833008	69.908974	12.287397	139.048828
69.919998	137.667007	69.928970	9.468864	138.995331

69.940002	136.167007	69.948967	7.483397	138.941925
69.959999	142.500000	69.968964	6.186489	138.888519
69.980003	143.667007	69.988968	5.500867	138.835114
70.000000	128.500000	70.008957	5.417359	138.781830
70.019997	143.167007	70.028954	5.992506	138.728577
70.040001	145.167007	70.048958	7.330209	138.675323
70.059998	142.667007	70.068954	9.506001	138.622131
70.080002	146.333008	70.088951	12.404908	138.569031
70.099998	152.167007	70.108948	15.481356	138.515930
70.119995	155.167007	70.128944	17.680746	138.462891
70.139999	152.333008	70.148949	17.928535	138.409851
70.159996	159.500000	70.168938	16.043299	138.356934
70.180000	147.000000	70.188942	12.950229	138.304047
70.199997	145.167007	70.208939	9.819190	138.251190
70.219994	141.000000	70.228928	7.275686	138.198425
70.239998	137.833008	70.248932	5.400080	138.145660
70.259995	137.833008	70.268929	4.053545	138.092957
70.279999	136.000000	70.288933	3.077339	138.040314
70.300003	140.500000	70.308929	2.358977	137.987732
70.320000	132.167007	70.328926	1.826010	137.935211
70.340004	143.167007	70.348930	1.432377	137.882721
70.360001	132.667007	70.368927	1.147505	137.830292
70.380005	129.000000	70.388924	0.949855	137.777924
70.400002	131.333008	70.408920	0.825489	137.725586
70.419998	133.333008	70.428917	0.767593	137.673340
70.440002	136.667007	70.448914	0.776766	137.621155
70.459999	130.833008	70.468910	0.860751	137.569031
70.480003	136.167007	70.488914	1.031738	137.516937
70.500000	135.833008	70.508911	1.297207	137.464905
70.519997	137.500000	70.528900	1.641358	137.412933
70.540001	138.500000	70.548904	2.001096	137.361053
70.559998	136.500000	70.568901	2.262802	137.309204
70.580002	122.333000	70.588898	2.325144	137.257385
70.599998	130.500000	70.608894	2.193684	137.205688
70.619995	128.000000	70.628891	1.991030	137.154053
70.639999	143.167007	70.648895	1.862994	137.102478
70.659996	142.667007	70.668884	1.910577	137.050964
70.680000	136.333008	70.688889	2.201128	136.999481

70.699997	131.833008	70.708885	2.805319	136.948059
70.719994	134.833008	70.728882	3.806784	136.896729
70.739998	141.167007	70.748878	5.267396	136.845490
70.759995	140.333008	70.768875	7.132303	136.794281
70.779999	139.000000	70.788879	9.090631	136.743134
70.800003	145.833008	70.808876	10.526578	136.692108
70.820000	142.833008	70.828873	10.799900	136.641052
70.840004	144.833008	70.848877	9.775457	136.590179
70.860001	131.667007	70.868874	7.983080	136.539307
70.880005	132.167007	70.888878	6.125560	136.488525
70.900002	134.000000	70.908875	4.604153	136.437805
70.919998	135.500000	70.928871	3.486931	136.387177
70.940002	128.833008	70.948868	2.696465	136.336609
70.959999	129.833008	70.968864	2.139202	136.286133
70.980003	141.000000	70.988869	1.747046	136.235687
71.000000	131.667007	71.008865	1.477742	136.185364
71.019997	133.167007	71.028854	1.305487	136.135132
71.040001	134.167007	71.048859	1.214931	136.084869
71.059998	133.167007	71.068855	1.198790	136.034790
71.080002	131.333008	71.088852	1.257542	135.984741
71.099998	135.333008	71.108849	1.402088	135.934784
71.119995	135.500000	71.128845	1.658146	135.884888
71.139999	138.667007	71.148849	2.072765	135.835083
71.159996	140.333008	71.168839	2.720633	135.785370
71.180000	128.833008	71.188843	3.711156	135.735718
71.199997	153.833008	71.208839	5.177054	135.686157
71.219994	142.500000	71.228836	7.236275	135.636658
71.239998	144.333008	71.248833	9.885369	135.587280
71.259995	152.500000	71.268829	12.826832	135.537964
71.279999	148.667007	71.288834	15.352403	135.488678
71.300003	152.333008	71.308830	16.574535	135.439545
71.320000	152.333008	71.328827	16.101177	135.390472
71.340004	151.667007	71.348831	14.455142	135.341461
71.360001	150.333008	71.368828	12.624969	135.292572
71.380005	154.167007	71.388824	11.336167	135.243774
71.400002	150.667007	71.408821	10.869570	135.195038
71.419998	146.667007	71.428818	11.295392	135.146362
71.440002	151.167007	71.448814	12.721630	135.097839

71.459999	154.667007	71.468811	15.442242	135.049347
71.480003	158.667007	71.488815	20.043547	135.000977
71.500000	167.667007	71.508812	27.507034	134.952698
71.519997	171.333008	71.528801	39.322906	134.904510
71.540001	196.167007	71.548805	57.529728	134.856384
71.559998	223.000000	71.568802	84.354591	134.808350
71.580002	270.832977	71.588799	121.208046	134.760376
71.599998	301.500000	71.608795	166.392868	134.712585
71.619995	350.166992	71.628792	212.185089	134.664825
71.639999	363.000000	71.648796	244.556641	134.617157
71.659996	392.166992	71.668785	250.132599	134.569641
71.680000	357.332977	71.688789	227.279419	134.522125
71.699997	310.666992	71.708786	187.664154	134.474762
71.719994	288.332977	71.728783	145.657715	134.427490
71.739998	251.000000	71.748779	109.646080	134.380310
71.759995	205.333008	71.768776	81.799255	134.333221
71.779999	196.833008	71.788780	61.167557	134.286194
71.800003	182.500000	71.808777	46.021488	134.239319
71.820000	179.667007	71.828773	34.818729	134.192535
71.840004	162.667007	71.848778	26.463100	134.145813
71.860001	154.500000	71.868774	20.226068	134.099213
71.880005	149.667007	71.888771	15.598285	134.052734
71.900002	145.333008	71.908768	12.211371	134.006317
71.919998	147.333008	71.928764	9.784081	133.960022
71.940002	144.000000	71.948761	8.099821	133.913788
71.959999	143.833008	71.968758	6.994321	133.867706
71.980003	140.833008	71.988762	6.349178	133.821686
72.000000	140.333008	72.008759	6.088362	133.775818
72.019997	139.500000	72.028748	6.175441	133.729980
72.040001	145.167007	72.048752	6.619037	133.684326
72.059998	148.000000	72.068748	7.484097	133.638733
72.080002	145.333008	72.088745	8.918488	133.593262
72.099998	152.667007	72.108742	11.196221	133.547852
72.119995	148.000000	72.128738	14.783251	133.502563
72.139999	154.667007	72.148743	20.425652	133.457397
72.159996	155.500000	72.168732	29.220730	133.412354
72.180000	176.500000	72.188736	42.666031	133.367371
72.199997	191.500000	72.208733	62.394920	133.322510

72.219994	212.333008	72.228722	89.458099	133.277771
72.239998	253.167007	72.248726	122.754509	133.233154
72.259995	287.332977	72.268723	156.803619	133.188599
72.279999	292.000000	72.288727	181.501221	133.144165
72.300003	311.666992	72.308723	186.833038	133.099884
72.320000	295.332977	72.328720	171.140320	133.055664
72.340004	270.332977	72.348724	142.894547	133.011566
72.360001	239.500000	72.368713	112.933266	132.967590
72.380005	214.833008	72.388718	87.144196	132.923706
72.400002	197.000000	72.408714	66.621796	132.879944
72.419998	181.000000	72.428711	50.614048	132.836304
72.440002	170.333008	72.448708	38.274128	132.792755
72.459999	155.500000	72.468704	28.882093	132.749329
72.480003	163.500000	72.488708	21.790493	132.705994
72.500000	151.500000	72.508698	16.460178	132.662811
72.519997	143.500000	72.528694	12.457549	132.619751
72.540001	137.167007	72.548698	9.461889	132.576721
72.559998	136.667007	72.568695	7.231225	132.533875
72.580002	137.000000	72.588692	5.576536	132.491150
72.599998	135.833008	72.608688	4.352829	132.448517
72.619995	147.167007	72.628685	3.448956	132.406006
72.639999	129.667007	72.648682	2.781119	132.363647
72.659996	138.333008	72.668678	2.287497	132.321320
72.680000	133.500000	72.688683	1.923157	132.279175
72.699997	131.000000	72.708679	1.658380	132.237122
72.719994	133.667007	72.728676	1.472801	132.195221
72.739998	135.333008	72.748680	1.356033	132.153320
72.759995	133.667007	72.768677	1.307104	132.111694
72.779999	129.333008	72.788673	1.335518	132.070129
72.800003	133.667007	72.808678	1.464280	132.028625
72.820000	129.500000	72.828674	1.733544	131.987305
72.840004	133.833008	72.848679	2.202672	131.946045
72.860001	134.000000	72.868668	2.947097	131.904968
72.880005	140.333008	72.888672	4.039763	131.864014
72.900002	142.333008	72.908669	5.493625	131.823181
72.919998	145.167007	72.928658	7.169969	131.782410
72.940002	141.333008	72.948662	8.693091	131.741821
72.959999	148.000000	72.968658	9.514750	131.701355

72.980003	146.667007	72.988663	9.258370	131.660950
73.000000	137.333008	73.008652	8.060018	131.620728
73.019997	142.000000	73.028648	6.456532	131.580597
73.040001	140.667007	73.048653	4.947008	131.540588
73.059998	130.167007	73.068642	3.750997	131.500702
73.080002	129.000000	73.088646	2.869193	131.460968
73.099998	130.167007	73.108643	2.229694	131.421326
73.119995	125.167000	73.128639	1.761063	131.381836
73.139999	134.333008	73.148636	1.413740	131.342438
73.159996	134.500000	73.168633	1.156203	131.303192
73.180000	131.833008	73.188637	0.967916	131.264069
73.199997	135.667007	73.208626	0.834714	131.225098
73.219994	136.333008	73.228622	0.745645	131.186188
73.239998	138.000000	73.248627	0.692433	131.147430
73.259995	132.667007	73.268623	0.668806	131.108795
73.279999	136.333008	73.288620	0.670370	131.070312
73.300003	138.000000	73.308624	0.694147	131.031921
73.320000	131.500000	73.328621	0.738791	130.993652
73.340004	136.000000	73.348618	0.804559	130.955566
73.360001	130.667007	73.368614	0.892981	130.917572
73.380005	135.333008	73.388618	1.006986	130.879700
73.400002	135.000000	73.408615	1.150775	130.841949
73.419998	135.833008	73.428604	1.330161	130.804382
73.440002	130.333008	73.448608	1.553093	130.766876
73.459999	140.500000	73.468605	1.829538	130.729523
73.480003	139.167007	73.488602	2.173400	130.692322
73.500000	130.667007	73.508598	2.604396	130.655212
73.519997	137.500000	73.528595	3.152362	130.618256
73.540001	134.333008	73.548599	3.865217	130.581421
73.559998	140.167007	73.568588	4.820509	130.544739
73.580002	138.500000	73.588593	6.150793	130.508148
73.599998	145.167007	73.608589	8.069432	130.471710
73.619995	144.333008	73.628578	10.921165	130.435425
73.639999	153.833008	73.648582	15.240413	130.399200
73.659996	151.500000	73.668579	21.786982	130.363159
73.680000	153.000000	73.688583	31.556747	130.327240
73.699997	176.500000	73.708572	45.578773	130.291473
73.719994	194.667007	73.728569	64.435524	130.255829

73.739998	225.000000	73.748573	87.178452	130.220306
73.759995	247.000000	73.768562	110.036072	130.184937
73.779999	258.332977	73.788567	126.485001	130.149628
73.800003	254.500000	73.808571	130.329834	130.114502
73.820000	247.167007	73.828568	120.887344	130.079498
73.840004	231.500000	73.848564	104.118553	130.044617
73.860001	222.167007	73.868561	87.930763	130.009888
73.880005	205.667007	73.888565	77.609291	129.975311
73.900002	206.167007	73.908554	75.439514	129.940826
73.919998	209.667007	73.928551	82.398819	129.906494
73.940002	222.500000	73.948555	99.243568	129.872284
73.959999	250.167007	73.968552	126.013077	129.838226
73.980003	288.832977	73.988548	160.255020	129.804321
74.000000	329.166992	74.008545	194.782166	129.770508
74.019997	362.000000	74.028542	217.806076	129.736847
74.040001	338.500000	74.048538	218.653519	129.703308
74.059998	321.832977	74.068535	196.276703	129.669891
74.080002	286.500000	74.088539	160.519485	129.636627
74.099998	248.667007	74.108536	123.743317	129.603516
74.119995	223.667007	74.128525	93.067001	129.570496
74.139999	202.667007	74.148529	69.854874	129.537628
74.159996	186.000000	74.168526	52.819649	129.504913
74.180000	185.000000	74.188522	40.220253	129.472290
74.199997	159.000000	74.208519	30.735315	129.439850
74.219994	159.333008	74.228516	23.502020	129.407532
74.239998	149.833008	74.248512	17.964415	129.375336
74.259995	140.167007	74.268509	13.739701	129.343262
74.279999	142.000000	74.288513	10.539341	129.311340
74.300003	137.667007	74.308517	8.136098	129.279541
74.320000	139.167007	74.328506	6.345136	129.247925
74.340004	136.000000	74.348511	5.014270	129.216370
74.360001	132.500000	74.368507	4.027904	129.184998
74.380005	123.667000	74.388504	3.295769	129.153748
74.400002	126.833000	74.408501	2.751209	129.122620
74.419998	133.167007	74.428497	2.345925	129.091644
74.440002	137.000000	74.448502	2.045532	129.060791
74.459999	133.833008	74.468491	1.826577	129.030060
74.480003	129.333008	74.488495	1.672628	128.999481

74.500000	130.833008	74.508492	1.573411	128.969055
74.519997	130.167007	74.528481	1.522550	128.938721
74.540001	135.333008	74.548485	1.516952	128.908569
74.559998	135.333008	74.568481	1.556372	128.878510
74.580002	131.000000	74.588486	1.643337	128.848602
74.599998	138.000000	74.608475	1.783564	128.818848
74.619995	128.500000	74.628471	1.988293	128.789185
74.639999	126.333000	74.648476	2.275202	128.759705
74.659996	124.000000	74.668465	2.675807	128.730316
74.680000	123.833000	74.688469	3.244128	128.701080
74.699997	123.667000	74.708466	4.068339	128.671997
74.719994	129.500000	74.728455	5.292197	128.643005
74.739998	129.000000	74.748459	7.141743	128.614197
74.759995	146.167007	74.768463	9.948362	128.585480
74.779999	143.167007	74.788467	14.163569	128.556915
74.800003	147.167007	74.808464	20.315466	128.528473
74.820000	160.167007	74.828461	28.849909	128.500183
74.840004	176.167007	74.848465	39.745960	128.471985
74.860001	192.833008	74.868454	51.895767	128.444000
74.880005	193.167007	74.888458	62.744827	128.416107
74.900002	204.500000	74.908455	68.940269	128.388367
74.919998	212.500000	74.928452	68.626244	128.360687
74.940002	192.167007	74.948448	63.323788	128.333191
74.959999	186.333008	74.968445	56.987392	128.305817
74.980003	182.333008	74.988449	53.230911	128.278595
75.000000	181.667007	75.008438	53.708511	128.251526
75.019997	183.500000	75.028435	58.080044	128.224548
75.040001	198.000000	75.048439	64.310730	128.197723
75.059998	207.333008	75.068436	69.024673	128.170990
75.080002	197.167007	75.088432	68.854111	128.144409
75.099998	195.833008	75.108429	62.659607	128.117981
75.119995	174.333008	75.128426	52.427513	128.091675
75.139999	177.667007	75.148422	41.466019	128.065521
75.159996	164.333008	75.168419	32.072968	128.039459
75.180000	149.833008	75.188423	24.930084	128.013550
75.199997	149.000000	75.208412	19.791508	127.987793
75.219994	158.500000	75.228409	16.168102	127.962128
75.239998	162.500000	75.248413	13.665781	127.936584

75.259995	143.000000	75.268410	12.033736	127.911224
75.279999	149.333008	75.288406	11.133492	127.885956
75.300003	153.833008	75.308411	10.914597	127.860840
75.320000	153.333008	75.328407	11.408104	127.835846
75.340004	169.167007	75.348404	12.746648	127.810974
75.360001	162.500000	75.368401	15.212370	127.786224
75.380005	168.000000	75.388405	19.311972	127.761597
75.400002	164.333008	75.408401	25.867943	127.737152
75.419998	184.500000	75.428391	36.126560	127.712799
75.440002	195.500000	75.448395	51.829464	127.688538
75.459999	224.167007	75.468391	75.030685	127.664490
75.480003	264.332977	75.488388	107.550224	127.640503
75.500000	301.000000	75.508385	149.519058	127.616638
75.519997	353.000000	75.528381	197.041565	127.592957
75.540001	388.000000	75.548378	240.215195	127.569366
75.559998	405.666992	75.568375	264.959686	127.545929
75.580002	383.832977	75.588379	261.076477	127.522583
75.599998	364.166992	75.608376	230.847977	127.499329
75.619995	335.832977	75.628365	187.515854	127.476288
75.639999	303.332977	75.648369	144.756897	127.453339
75.659996	253.500000	75.668365	109.674606	127.430542
75.680000	227.667007	75.688362	83.199379	127.407837
75.699997	201.667007	75.708359	63.655319	127.385254
75.719994	190.333008	75.728355	49.093887	127.362793
75.739998	172.667007	75.748360	38.066349	127.340454
75.759995	169.667007	75.768349	29.649721	127.318268
75.779999	161.667007	75.788353	23.230940	127.296173
75.800003	157.667007	75.808357	18.404007	127.274231
75.820000	152.167007	75.828346	14.862453	127.252380
75.840004	145.500000	75.848351	12.360845	127.230682
75.860001	145.667007	75.868347	10.721194	127.209106
75.880005	140.333008	75.888344	9.822792	127.187622
75.900002	162.000000	75.908340	9.619840	127.166321
75.919998	151.667007	75.928337	10.160771	127.145081
75.940002	150.333008	75.948341	11.619978	127.123962
75.959999	155.667007	75.968330	14.336091	127.102997
75.980003	165.333008	75.988335	18.869148	127.082123
76.000000	166.500000	76.008331	26.015162	127.061371

76.019997	170.667007	76.028320	36.768852	127.040771
76.040001	189.333008	76.048325	52.101219	127.020264
76.059998	218.833008	76.068321	72.300079	126.999878
76.080002	238.333008	76.088318	95.942024	126.979614
76.099998	263.166992	76.108315	118.758919	126.959473
76.119995	275.000000	76.128311	133.976074	126.939453
76.139999	266.500000	76.148315	135.639862	126.919525
76.159996	263.166992	76.168304	123.255013	126.899719
76.180000	242.167007	76.188309	102.448448	126.880035
76.199997	212.167007	76.208305	80.488144	126.860474
76.219994	204.833008	76.228294	61.841385	126.841034
76.239998	175.500000	76.248299	47.613598	126.821716
76.259995	170.000000	76.268295	37.209488	126.802490
76.279999	171.000000	76.288300	29.656717	126.783386
76.300003	166.167007	76.308296	24.218386	126.764374
76.320000	152.167007	76.328293	20.456911	126.745514
76.340004	149.000000	76.348297	18.189590	126.726746
76.360001	147.000000	76.368286	17.437918	126.708099
76.380005	152.833008	76.388290	18.409218	126.689545
76.400002	153.333008	76.408287	21.507618	126.671082
76.419998	165.333008	76.428276	27.315866	126.652802
76.440002	167.667007	76.448280	36.497887	126.634583
76.459999	186.000000	76.468277	49.444012	126.616455
76.480003	210.333008	76.488281	65.651573	126.598480
76.500000	227.500000	76.508270	82.814125	126.580627
76.519997	254.167007	76.528267	96.572289	126.562836
76.540001	235.500000	76.548271	101.959488	126.545166
76.559998	237.833008	76.568260	96.748688	126.527618
76.580002	228.000000	76.588264	83.456856	126.510162
76.599998	209.500000	76.608261	67.347588	126.492798
76.619995	194.833008	76.628250	52.684307	126.475616
76.639999	183.167007	76.648254	41.170353	126.458466
76.659996	171.667007	76.668251	32.754879	126.441437
76.680000	162.667007	76.688255	26.763725	126.424500
76.699997	155.000000	76.708244	22.577869	126.407715
76.719994	162.833008	76.728241	19.781080	126.390991
76.739998	160.167007	76.748245	18.160936	126.374359
76.759995	155.667007	76.768234	17.659286	126.357849

76.779999	158.667007	76.788239	18.360107	126.341431
76.800003	152.000000	76.808243	20.523579	126.325134
76.820000	161.333008	76.828232	24.647718	126.308929
76.840004	170.500000	76.848236	31.580828	126.292847
76.860001	168.333008	76.868233	42.604950	126.276825
76.880005	192.167007	76.888237	59.524033	126.260864
76.900002	212.833008	76.908226	84.495750	126.245087
76.919998	247.667007	76.928223	119.560905	126.229370
76.940002	304.166992	76.948227	165.244308	126.213745
76.959999	357.666992	76.968216	218.232452	126.198242
76.980003	411.666992	76.988220	269.408142	126.182800
77.000000	436.666992	77.008217	304.616119	126.167511
77.019997	431.500000	77.028206	312.104431	126.152283
77.040001	421.500000	77.048210	291.956879	126.137115
77.059998	378.500000	77.068207	257.389069	126.122070
77.080002	353.832977	77.088211	225.235672	126.107117
77.099998	345.500000	77.108200	206.649506	126.092255
77.119995	337.332977	77.128204	204.970825	126.077454
77.139999	368.832977	77.148209	217.678970	126.062805
77.159996	371.832977	77.168198	237.453293	126.048218
77.180000	397.000000	77.188202	253.066696	126.033691
77.199997	388.166992	77.208199	253.002197	126.019287
77.219994	377.500000	77.228188	232.390594	126.004974
77.239998	334.666992	77.248192	196.538391	125.990753
77.259995	288.000000	77.268188	156.331619	125.976624
77.279999	255.000000	77.288193	120.340317	125.962524
77.300003	225.333008	77.308189	91.851944	125.948547
77.320000	200.833008	77.328186	70.427177	125.934692
77.340004	184.667007	77.348190	54.416882	125.920868
77.360001	174.167007	77.368179	42.303551	125.907166
77.380005	176.167007	77.388184	32.982368	125.893524
77.400002	159.667007	77.408180	25.759596	125.879974
77.419998	151.000000	77.428169	20.165945	125.866516
77.440002	154.833008	77.448174	15.864328	125.853088
77.459999	140.000000	77.468170	12.608501	125.839813
77.480003	138.667007	77.488174	10.199141	125.826599
77.500000	129.333008	77.508163	8.488725	125.813416
77.519997	140.167007	77.528160	7.369718	125.800354

77.540001	135.500000	77.548164	6.779781	125.787354
77.559998	138.833008	77.568153	6.690230	125.774475
77.580002	145.500000	77.588158	7.084064	125.761597
77.599998	136.833008	77.608154	7.912377	125.748840
77.619995	137.000000	77.628143	9.027516	125.736206
77.639999	138.500000	77.648148	10.130341	125.723541
77.659996	138.833008	77.668144	10.809999	125.710999
77.680000	130.667007	77.688148	10.754775	125.698547
77.699997	131.833008	77.708138	9.987320	125.686157
77.719994	132.500000	77.728134	8.856816	125.673828
77.739998	127.667000	77.748138	7.776683	125.661560
77.759995	131.833008	77.768127	7.005774	125.649384
77.779999	130.000000	77.788132	6.633097	125.637268
77.800003	128.333008	77.808136	6.679521	125.625214
77.820000	129.333008	77.828125	7.179714	125.613220
77.840004	137.167007	77.848129	8.228374	125.601318
77.860001	136.833008	77.868126	9.994739	125.589508
77.880005	141.167007	77.888130	12.728424	125.577698
77.900002	143.333008	77.908119	16.727804	125.565979
77.919998	156.000000	77.928116	22.272411	125.554352
77.940002	162.333008	77.948120	29.433678	125.542755
77.959999	164.667007	77.968109	37.783455	125.531250
77.980003	175.833008	77.988113	46.195778	125.519775
78.000000	174.333008	78.008110	53.034611	125.508392
78.019997	187.333008	78.028099	57.212749	125.497040
78.040001	188.833008	78.048103	59.441223	125.485779
78.059998	199.667007	78.068100	62.403427	125.474548
78.080002	194.333008	78.088104	69.651176	125.463379
78.099998	212.833008	78.108093	84.471916	125.452271
78.119995	229.333008	78.128090	109.566399	125.441223
78.139999	268.000000	78.148094	146.704620	125.430237
78.159996	324.500000	78.168083	195.420639	125.419281
78.180000	393.832977	78.188087	250.878845	125.408417
78.199997	441.332977	78.208084	301.701935	125.397583
78.219994	449.332977	78.228073	332.144867	125.386810
78.239998	466.166992	78.248077	330.235352	125.376068
78.259995	454.332977	78.268074	296.951569	125.365387
78.279999	391.832977	78.288071	245.968826	125.354797

78.300003	341.166992	78.308075	193.274261	125.344177
78.320000	277.166992	78.328072	148.447037	125.333649
78.340004	241.833008	78.348076	113.738930	125.323151
78.360001	220.167007	78.368065	87.706367	125.312775
78.380005	205.667007	78.388069	68.036041	125.302368
78.400002	193.500000	78.408066	52.920010	125.292023
78.419998	170.833008	78.428055	41.131298	125.281708
78.440002	165.000000	78.448059	31.875380	125.271484
78.459999	152.667007	78.468056	24.634787	125.261292
78.480003	142.333008	78.488052	19.010351	125.251099
78.500000	147.167007	78.508049	14.681771	125.240967
78.519997	140.167007	78.528046	11.379526	125.230865
78.540001	131.833008	78.548050	8.875860	125.220825
78.559998	133.500000	78.568039	6.985926	125.210815
78.580002	138.333008	78.588043	5.556297	125.200836
78.599998	135.333008	78.608040	4.471369	125.190918
78.619995	130.500000	78.628029	3.641425	125.181030
78.639999	132.000000	78.648033	2.999098	125.171143
78.659996	126.333000	78.668030	2.496482	125.161316
78.680000	121.667000	78.688026	2.097966	125.151550
78.699997	126.667000	78.708023	1.778028	125.141785
78.719994	120.667000	78.728020	1.518190	125.132080
78.739998	114.500000	78.748016	1.304963	125.122375
78.759995	122.167000	78.768013	1.128384	125.112732
78.779999	128.000000	78.788017	0.980932	125.103058
78.800003	125.000000	78.808022	0.856988	125.093475
78.820000	123.833000	78.828011	0.752225	125.083862
78.840004	114.167000	78.848015	0.662924	125.074341
78.860001	121.167000	78.868011	0.586700	125.064819
78.880005	125.667000	78.888008	0.521215	125.055298
78.900002	123.333000	78.908005	0.464701	125.045837
78.919998	126.500000	78.928001	0.415726	125.036377
78.940002	123.500000	78.947998	0.373115	125.026947
78.959999	124.000000	78.967995	0.335549	125.017548
78.980003	116.667000	78.987999	0.302933	125.008148
79.000000	132.000000	79.007996	0.274264	124.998779
79.019997	118.833000	79.027985	0.248982	124.989441
79.040001	123.167000	79.047989	0.226595	124.980103

79.059998	123.667000	79.067986	0.206735	124.970795
79.080002	119.833000	79.087982	0.189063	124.961487
79.099998	127.000000	79.107979	0.173296	124.952209
79.119995	127.833000	79.127975	0.159193	124.942993
79.139999	116.833000	79.147972	0.146549	124.933716
79.159996	127.667000	79.167969	0.135187	124.924500
79.180000	121.000000	79.187973	0.124952	124.915283
79.199997	114.333000	79.207962	0.115723	124.906067
79.219994	126.333000	79.227959	0.107432	124.896912
79.239998	119.500000	79.247963	0.099885	124.887695
79.259995	114.000000	79.267960	0.093030	124.878510
79.279999	110.167000	79.287956	0.086800	124.869324
79.300003	117.000000	79.307961	0.081128	124.860168
79.320000	126.167000	79.327957	0.075962	124.851013
79.340004	120.500000	79.347954	0.071239	124.841858
79.360001	123.500000	79.367950	0.066934	124.832703
79.380005	124.667000	79.387955	0.062996	124.823608
79.400002	121.833000	79.407944	0.059396	124.814453
79.419998	118.333000	79.427940	0.056098	124.805328
79.440002	118.667000	79.447945	0.053076	124.796143
79.459999	124.000000	79.467934	0.050308	124.787048
79.480003	118.000000	79.487938	0.047505	124.777893
79.500000	118.833000	79.507935	0.045186	124.768799
79.519997	113.833000	79.527931	0.043062	124.759644
79.540001	117.333000	79.547928	0.041118	124.750488
79.559998	116.500000	79.567924	0.039342	124.741333
79.580002	122.000000	79.587929	0.037800	124.732178
79.599998	116.500000	79.607918	0.036593	124.723053
79.619995	119.000000	79.627914	0.035262	124.713867
79.639999	116.500000	79.647919	0.034133	124.704712
79.659996	117.500000	79.667908	0.033054	124.695557
79.680000	121.667000	79.687912	0.032091	124.686340
79.699997	113.333000	79.707909	0.031240	124.677185
79.719994	106.833000	79.727898	0.030497	124.667938
79.739998	117.667000	79.747902	0.029858	124.658691
79.759995	123.667000	79.767899	0.029322	124.649475
79.779999	123.500000	79.787903	0.028888	124.640259
79.800003	118.667000	79.807899	0.028555	124.630981

79.820000	122.833000	79.827896	0.028234	124.621735
79.840004	119.833000	79.847900	0.027982	124.612427
79.860001	116.667000	79.867889	0.027972	124.603149
79.880005	122.333000	79.887894	0.028066	124.593781
79.900002	114.667000	79.907890	0.028276	124.584473
79.919998	119.000000	79.927879	0.028607	124.575104
79.940002	112.500000	79.947884	0.029068	124.565735
79.959999	120.000000	79.967880	0.029669	124.556305
79.980003	123.333000	79.987877	0.030422	124.546906
80.000000	124.000000	80.007874	0.031342	124.537445
80.019997	123.000000	80.027878	0.032446	124.527954
80.040001	117.667000	80.047882	0.033756	124.518433
80.059998	123.000000	80.067871	0.035294	124.508911
80.080002	119.500000	80.087875	0.037094	124.499390
80.099998	121.000000	80.107872	0.039190	124.489807
80.119995	124.000000	80.127861	0.041624	124.480225
80.139999	118.000000	80.147865	0.044452	124.470581
80.159996	118.500000	80.167862	0.047734	124.460938
80.180000	129.667007	80.187859	0.051548	124.451233
80.199997	119.333000	80.207855	0.055986	124.441528
80.219994	111.667000	80.227852	0.061164	124.431763
80.239998	122.667000	80.247849	0.067220	124.421967
80.259995	110.833000	80.267845	0.074324	124.412140
80.279999	110.167000	80.287849	0.082691	124.402283
80.300003	116.333000	80.307846	0.092569	124.392395
80.320000	117.333000	80.327843	0.104271	124.382446
80.340004	120.833000	80.347847	0.118180	124.372498
80.360001	114.000000	80.367844	0.134739	124.362488
80.380005	114.333000	80.387840	0.154281	124.352417
80.400002	120.500000	80.407837	0.177902	124.342377
80.419998	116.833000	80.427834	0.206195	124.332245
80.440002	117.667000	80.447830	0.240205	124.322083
80.459999	117.833000	80.467827	0.281346	124.311890
80.480003	108.667000	80.487831	0.331595	124.301605
80.500000	114.500000	80.507820	0.394432	124.291321
80.519997	119.167000	80.527817	0.475000	124.281006
80.540001	124.333000	80.547821	0.581932	124.270569
80.559998	112.500000	80.567810	0.729257	124.260162

80.580002	112.167000	80.587814	0.939442	124.249695
80.599998	119.667000	80.607811	1.247257	124.239197
80.619995	117.000000	80.627808	1.704675	124.228577
80.639999	123.333000	80.647804	2.383959	124.217957
80.659996	117.833000	80.667801	3.378363	124.207275
80.680000	126.500000	80.687805	4.791154	124.196533
80.699997	117.500000	80.707794	6.698812	124.185791
80.719994	125.833000	80.727791	9.090477	124.174957
80.739998	133.667007	80.747795	11.760584	124.164062
80.759995	131.167007	80.767784	14.232611	124.153137
80.779999	138.000000	80.787788	15.843460	124.142151
80.800003	135.167007	80.807793	16.047714	124.131073
80.820000	139.667007	80.827782	14.812127	124.119995
80.840004	145.333008	80.847786	12.661009	124.108826
80.860001	136.500000	80.867783	10.307811	124.097595
80.880005	129.000000	80.887779	8.246282	124.086304
80.900002	133.667007	80.907776	6.652444	124.074982
80.919998	134.333008	80.927773	5.505745	124.063599
80.940002	127.333000	80.947777	4.730261	124.052124
80.959999	123.000000	80.967766	4.271985	124.040588
80.980003	128.833008	80.987770	4.120507	124.028992
81.000000	130.000000	81.007767	4.314087	124.017334
81.019997	131.667007	81.027756	4.936031	124.005615
81.040001	127.833000	81.047760	6.112445	123.993835
81.059998	128.833008	81.067757	7.992918	123.981995
81.080002	138.167007	81.087753	10.709209	123.970093
81.099998	136.500000	81.107750	14.278418	123.958069
81.119995	133.500000	81.127747	18.452160	123.946045
81.139999	150.833008	81.147743	22.570599	123.933899
81.159996	146.000000	81.167740	25.598022	123.921722
81.180000	137.833008	81.187744	26.530954	123.909485
81.199997	145.000000	81.207733	25.042238	123.897125
81.219994	139.167007	81.227730	21.762053	123.884735
81.239998	136.667007	81.247734	17.827791	123.872253
81.259995	132.000000	81.267731	14.178076	123.859711
81.279999	136.333008	81.287727	11.226736	123.847107
81.300003	133.833008	81.307732	8.991949	123.834412
81.320000	130.167007	81.327728	7.335828	123.821594

81.340004	127.333000	81.347725	6.108895	123.808746
81.360001	133.000000	81.367722	5.203772	123.795837
81.380005	128.333008	81.387726	4.553410	123.782837
81.400002	129.167007	81.407715	4.119627	123.769775
81.419998	122.667000	81.427711	3.880512	123.756592
81.440002	124.500000	81.447716	3.827821	123.743347
81.459999	124.500000	81.467705	3.966625	123.730011
81.480003	128.500000	81.487709	4.321374	123.716614
81.500000	118.500000	81.507706	4.945430	123.703156
81.519997	127.333000	81.527695	5.937271	123.689575
81.540001	123.167000	81.547699	7.464440	123.675903
81.559998	129.167007	81.567696	9.787067	123.662170
81.580002	135.000000	81.587700	13.294642	123.648346
81.599998	139.167007	81.607689	18.516949	123.634460
81.619995	144.833008	81.627686	26.127064	123.620453
81.639999	159.833008	81.647690	36.828163	123.606384
81.659996	165.833008	81.667679	51.072952	123.592224
81.680000	191.333008	81.687683	68.623329	123.577972
81.699997	211.167007	81.707680	87.840820	123.563629
81.719994	231.833008	81.727669	105.430367	123.549194
81.739998	236.000000	81.747673	117.235626	123.534668
81.759995	251.167007	81.767670	120.311775	123.520050
81.779999	238.167007	81.787666	114.884186	123.505371
81.800003	234.167007	81.807671	103.839310	123.490601
81.820000	211.500000	81.827667	90.370537	123.475708
81.840004	190.500000	81.847664	76.407906	123.460693
81.860001	191.167007	81.867661	62.932373	123.445618
81.880005	171.333008	81.887665	50.675220	123.430481
81.900002	165.667007	81.907654	40.209629	123.415222
81.919998	152.833008	81.927650	31.702553	123.399841
81.940002	148.667007	81.947655	24.982969	123.384430
81.959999	146.167007	81.967651	19.727791	123.368896
81.980003	142.500000	81.987648	15.612808	123.353210
82.000000	132.500000	82.007645	12.384859	123.337463
82.019997	128.500000	82.027641	9.858388	123.321655
82.040001	121.000000	82.047638	7.896498	123.305725
82.059998	128.667007	82.067635	6.394362	123.289673
82.080002	127.500000	82.087639	5.269244	123.273560

82.099998	130.500000	82.107628	4.458487	123.257324
82.119995	120.667000	82.127625	3.915668	123.240967
82.139999	127.500000	82.147629	3.616376	123.224518
82.159996	123.167000	82.167618	3.560660	123.207977
82.180000	129.500000	82.187622	3.778565	123.191345
82.199997	119.167000	82.207619	4.337052	123.174622
82.219994	117.667000	82.227608	5.345493	123.157745
82.239998	115.833000	82.247612	6.957981	123.140808
82.259995	129.333008	82.267609	9.352116	123.123749
82.279999	132.167007	82.287605	12.683169	123.106567
82.300003	131.333008	82.307610	16.976425	123.089294
82.320000	145.500000	82.327606	21.953232	123.071960
82.340004	146.833008	82.347610	26.881599	123.054443
82.360001	141.667007	82.367599	30.593393	123.036865
82.380005	148.333008	82.387604	31.935169	123.019165
82.400002	146.500000	82.407600	30.458494	123.001343
82.419998	144.167007	82.427589	26.798384	122.983459
82.440002	144.667007	82.447594	22.229368	122.965393
82.459999	136.167007	82.467590	17.886917	122.947266
82.480003	132.500000	82.487587	14.329844	122.929077
82.500000	128.667007	82.507584	11.639126	122.910706
82.519997	136.167007	82.527580	9.685011	122.892242
82.540001	119.500000	82.547577	8.319312	122.873657
82.559998	125.333000	82.567574	7.453320	122.854980
82.580002	127.833000	82.587578	7.072089	122.836182
82.599998	127.167000	82.607567	7.229814	122.817261
82.619995	124.500000	82.627563	8.043157	122.798218
82.639999	118.833000	82.647568	9.684004	122.779114
82.659996	127.167000	82.667557	12.350818	122.759888
82.680000	128.500000	82.687561	16.215067	122.740479
82.699997	144.833008	82.707558	21.277506	122.721008
82.719994	144.000000	82.727547	27.180603	122.701416
82.739998	151.667007	82.747551	33.029301	122.681702
82.759995	158.667007	82.767548	37.419563	122.661896
82.779999	149.500000	82.787552	38.982803	122.641937
82.800003	153.667007	82.807549	37.205547	122.621887
82.820000	153.000000	82.827545	32.850918	122.601746
82.840004	146.500000	82.847549	27.436960	122.581421

82.860001	138.667007	82.867538	22.312164	122.561035
82.880005	130.000000	82.887543	18.137655	122.540497
82.900002	138.333008	82.907539	15.018268	122.519836
82.919998	131.667007	82.927528	12.799420	122.499084
82.940002	129.167007	82.947533	11.301064	122.478210
82.959999	134.333008	82.967529	10.416935	122.457214
82.980003	134.167007	82.987526	10.126708	122.436096
83.000000	130.000000	83.007523	10.497382	122.414856
83.019997	137.333008	83.027519	11.682931	122.393494
83.040001	139.500000	83.047516	13.929959	122.372009
83.059998	143.333008	83.067513	17.578541	122.350403
83.080002	153.833008	83.087517	23.039257	122.328674
83.099998	161.500000	83.107506	30.694918	122.306854
83.119995	163.167007	83.127502	40.740326	122.284882
83.139999	183.333008	83.147507	52.872898	122.262756
83.159996	189.667007	83.167496	65.994644	122.240601
83.180000	204.833008	83.187500	78.258842	122.218262
83.199997	201.500000	83.207497	87.557922	122.195801
83.219994	215.167007	83.227493	92.551910	122.173218
83.239998	211.333008	83.247490	93.410126	122.150513
83.259995	218.000000	83.267487	91.932205	122.127716
83.279999	223.500000	83.287491	91.077507	122.104736
83.300003	202.833008	83.307487	94.085785	122.081696
83.320000	220.500000	83.327484	103.464569	122.058502
83.340004	242.333008	83.347488	120.209023	122.035156
83.360001	272.832977	83.367477	143.143295	122.011719
83.380005	305.666992	83.387482	168.430588	121.988159
83.400002	314.666992	83.407478	189.505280	121.964478
83.419998	332.332977	83.427467	199.048294	121.940674
83.440002	319.500000	83.447472	192.737732	121.916748
83.459999	299.832977	83.467468	172.249420	121.892670
83.480003	271.832977	83.487465	144.174225	121.868469
83.500000	229.167007	83.507462	115.676399	121.844147
83.519997	208.833008	83.527458	91.106224	121.819702
83.540001	191.333008	83.547455	71.649704	121.795135
83.559998	181.333008	83.567451	56.701790	121.770447
83.580002	181.500000	83.587456	45.168980	121.745667
83.599998	164.167007	83.607445	36.122795	121.720703

83.619995	156.500000	83.627441	28.903107	121.695618
83.639999	150.833008	83.647446	23.097122	121.670410
83.659996	136.000000	83.667435	18.439075	121.645111
83.680000	138.500000	83.687439	14.726074	121.619659
83.699997	141.667007	83.707436	11.805860	121.594116
83.719994	128.667007	83.727432	9.542131	121.568420
83.739998	126.667000	83.747429	7.816985	121.542542
83.759995	117.333000	83.767426	6.528535	121.516602
83.779999	126.167000	83.787430	5.590975	121.490509
83.800003	123.500000	83.807426	4.935791	121.464294
83.820000	126.333000	83.827423	4.508816	121.437958
83.840004	127.833000	83.847427	4.270222	121.411499
83.860001	124.167000	83.867416	4.192643	121.384888
83.880005	125.833000	83.887421	4.259031	121.358154
83.900002	126.833000	83.907417	4.462044	121.331360
83.919998	126.333000	83.927406	4.803770	121.304382
83.940002	126.667000	83.947411	5.298266	121.277283
83.959999	137.000000	83.967407	5.974344	121.250061
83.980003	136.833008	83.987404	6.885456	121.222717
84.000000	136.667007	84.007401	8.122819	121.195251
84.019997	140.500000	84.027397	9.837319	121.167603
84.040001	131.500000	84.047394	12.270366	121.139893
84.059998	144.667007	84.067390	15.796730	121.112000
84.080002	139.000000	84.087395	20.977573	121.084045
84.099998	146.167007	84.107384	28.599014	121.055908
84.119995	164.833008	84.127380	39.722889	121.027710
84.139999	175.000000	84.147385	55.622074	120.999268
84.159996	198.667007	84.167374	77.549103	120.970825
84.180000	233.167007	84.187378	106.336082	120.942200
84.199997	262.832977	84.207382	141.466156	120.913452
84.219994	314.000000	84.227371	180.102158	120.884644
84.239998	342.832977	84.247375	216.681274	120.855591
84.259995	375.166992	84.267372	243.789825	120.826416
84.279999	382.332977	84.287376	255.365112	120.797180
84.300003	380.000000	84.307373	249.786316	120.767822
84.320000	342.332977	84.327370	230.252945	120.738342
84.340004	311.500000	84.347374	202.156158	120.708679
84.360001	277.332977	84.367363	170.666885	120.678955

84.380005	246.667007	84.387367	139.779984	120.649048
84.400002	233.167007	84.407364	112.267212	120.619080
84.419998	203.667007	84.427353	89.368103	120.588989
84.440002	205.000000	84.447357	71.023460	120.558655
84.459999	183.167007	84.467354	56.541531	120.528320
84.480003	174.000000	84.487350	45.069477	120.497803
84.500000	165.167007	84.507347	35.906624	120.467163
84.519997	153.500000	84.527344	28.546186	120.436462
84.540001	152.500000	84.547340	22.631464	120.405579
84.559998	136.667007	84.567337	17.899881	120.374573
84.580002	133.333008	84.587341	14.141846	120.343445
84.599998	132.667007	84.607330	11.186562	120.312195
84.619995	129.667007	84.627327	8.879880	120.280884
84.639999	130.500000	84.647331	7.093537	120.249329
84.659996	124.667000	84.667320	5.720870	120.217773
84.680000	125.167000	84.687325	4.671150	120.186035
84.699997	124.833000	84.707321	3.875872	120.154175
84.719994	127.667000	84.727310	3.282241	120.122192
84.739998	117.667000	84.747314	2.852303	120.090088
84.759995	123.500000	84.767311	2.562483	120.057861
84.779999	115.500000	84.787308	2.398976	120.025513
84.800003	120.000000	84.807312	2.354910	119.993042
84.820000	126.333000	84.827309	2.424083	119.960510
84.840004	121.667000	84.847305	2.591503	119.927795
84.860001	120.500000	84.867302	2.821966	119.894958
84.880005	125.333000	84.887306	3.052666	119.862000
84.900002	127.333000	84.907295	3.201591	119.828979
84.919998	126.333000	84.927292	3.201056	119.795837
84.940002	122.333000	84.947296	3.037415	119.762512
84.959999	122.833000	84.967285	2.762833	119.729126
84.980003	118.000000	84.987289	2.460238	119.695557
85.000000	119.167000	85.007286	2.198331	119.661926
85.019997	122.167000	85.027283	2.009930	119.628174
85.040001	129.167007	85.047279	1.900361	119.594299
85.059998	115.833000	85.067276	1.863850	119.560303
85.080002	121.667000	85.087280	1.895357	119.526184
85.099998	125.167000	85.107269	1.995925	119.491943
85.119995	123.000000	85.127266	2.175211	119.457642

85.139999	113.833000	85.147270	2.453308	119.423157
85.159996	121.500000	85.167259	2.863329	119.388672
85.180000	114.833000	85.187263	3.457581	119.353943
85.199997	121.667000	85.207260	4.311074	119.319153
85.219994	116.000000	85.227249	5.528275	119.284241
85.239998	129.000000	85.247253	7.246556	119.249268
85.259995	126.167000	85.267250	9.623349	119.214111
85.279999	133.667007	85.287247	12.818827	119.178833
85.300003	135.833008	85.307251	16.956913	119.143555
85.320000	135.167007	85.327248	22.085526	119.108032
85.340004	148.167007	85.347244	28.201971	119.072510
85.360001	146.000000	85.367241	35.341953	119.036804
85.380005	162.333008	85.387245	43.672592	119.000977
85.400002	174.333008	85.407234	53.336510	118.965149
85.419998	188.000000	85.427231	64.003014	118.929138
85.440002	208.333008	85.447235	74.290688	118.893005
85.459999	201.667007	85.467224	81.764603	118.856812
85.480003	215.500000	85.487228	83.931381	118.820496
85.500000	212.833008	85.507225	79.739693	118.784058
85.519997	201.333008	85.527214	70.439064	118.747559
85.540001	188.000000	85.547218	58.770668	118.710876
85.559998	172.833008	85.567215	47.367344	118.674194
85.580002	165.167007	85.587212	37.677254	118.637329
85.599998	145.667007	85.607208	30.020510	118.600342
85.619995	139.000000	85.627205	24.122427	118.563354
85.639999	134.333008	85.647202	19.571203	118.526184
85.659996	130.333008	85.667198	16.031534	118.488892
85.680000	133.667007	85.687202	13.288149	118.451538
85.699997	131.333008	85.707191	11.227144	118.414124
85.719994	133.000000	85.727188	9.791309	118.376587
85.739998	128.833008	85.747192	8.957859	118.338928
85.759995	119.333000	85.767181	8.705631	118.301208
85.779999	123.667000	85.787186	8.979025	118.263306
85.800003	124.833000	85.807190	9.646317	118.225403
85.820000	127.333000	85.827179	10.461736	118.187378
85.840004	129.167007	85.847183	11.081343	118.149231
85.860001	128.333008	85.867180	11.163878	118.111023
85.880005	128.500000	85.887177	10.543623	118.072693

85.900002	122.000000	85.907173	9.330993	118.034302
85.919998	120.500000	85.927170	7.833761	117.995789
85.940002	125.000000	85.947174	6.363781	117.957214
85.959999	124.167000	85.967163	5.104860	117.918518
85.980003	117.000000	85.987167	4.099882	117.879761
86.000000	125.000000	86.007164	3.317669	117.840881
86.019997	114.333000	86.027153	2.704999	117.801941
86.040001	117.833000	86.047157	2.215651	117.762878
86.059998	112.833000	86.067154	1.818624	117.723755
86.080002	109.500000	86.087151	1.493446	117.684570
86.099998	112.833000	86.107147	1.226952	117.645264
86.119995	118.833000	86.127144	1.009737	117.605835
86.139999	117.000000	86.147141	0.834255	117.566406
86.159996	111.833000	86.167137	0.693929	117.526855
86.180000	109.000000	86.187141	0.582815	117.487183
86.199997	112.500000	86.207130	0.495748	117.447510
86.219994	118.833000	86.227127	0.428009	117.407715
86.239998	115.167000	86.247131	0.375710	117.367798
86.259995	113.333000	86.267120	0.335681	117.327881
86.279999	112.500000	86.287125	0.305277	117.287842
86.300003	116.667000	86.307129	0.282513	117.247681
86.320000	118.500000	86.327118	0.265854	117.207520
86.340004	122.000000	86.347122	0.254118	117.167236
86.360001	125.167000	86.367119	0.246454	117.126892
86.380005	121.833000	86.387115	0.242226	117.086487
86.400002	115.333000	86.407112	0.241100	117.045959
86.419998	125.000000	86.427109	0.242737	117.005432
86.440002	117.167000	86.447105	0.246963	116.964783
86.459999	124.167000	86.467102	0.253693	116.924072
86.480003	131.167007	86.487106	0.262923	116.883301
86.500000	122.500000	86.507095	0.274697	116.842468
86.519997	129.500000	86.527092	0.289146	116.801514
86.540001	116.167000	86.547096	0.306453	116.760498
86.559998	118.667000	86.567085	0.326841	116.719482
86.580002	118.667000	86.587090	0.350653	116.678345
86.599998	124.833000	86.607086	0.378254	116.637207
86.619995	112.667000	86.627075	0.410120	116.595947
86.639999	122.167000	86.647079	0.446873	116.554626

86.659996	120.667000	86.667076	0.489177	116.513245
86.680000	121.167000	86.687073	0.537899	116.471802
86.699997	116.167000	86.707069	0.594063	116.430298
86.719994	119.333000	86.727066	0.658908	116.388794
86.739998	120.333000	86.747063	0.733926	116.347107
86.759995	119.667000	86.767059	0.820913	116.305481
86.779999	114.333000	86.787064	0.922200	116.263733
86.800003	118.000000	86.807060	1.040055	116.221924
86.820000	119.333000	86.827057	1.177741	116.180115
86.840004	119.000000	86.847061	1.338932	116.138184
86.860001	120.167000	86.867050	1.527953	116.096252
86.880005	118.167000	86.887054	1.750140	116.054199
86.900002	120.667000	86.907051	2.011191	116.012207
86.919998	118.000000	86.927040	2.318247	115.970093
86.940002	112.833000	86.947044	2.679972	115.927856
86.959999	116.167000	86.967041	3.106145	115.885742
86.980003	120.333000	86.987038	3.609860	115.843506
87.000000	122.000000	87.007034	4.208637	115.801147
87.019997	128.167007	87.027031	4.927696	115.758850
87.040001	125.833000	87.047028	5.805249	115.716431
87.059998	133.833008	87.067024	6.901110	115.674011
87.080002	126.667000	87.087029	8.310545	115.631531
87.099998	131.000000	87.107018	10.180790	115.589050
87.119995	140.333008	87.127014	12.744516	115.546509
87.139999	142.833008	87.147018	16.350876	115.503906
87.159996	148.833008	87.167007	21.502142	115.461304
87.180000	149.500000	87.187012	28.912750	115.418579
87.199997	165.000000	87.207008	39.500774	115.375916
87.219994	183.000000	87.226997	54.369968	115.333191
87.239998	202.833008	87.247002	74.683937	115.290405
87.259995	225.167007	87.266998	101.278236	115.247620
87.279999	275.832977	87.286995	134.199509	115.204773
87.300003	317.832977	87.306999	172.064728	115.161865
87.320000	335.000000	87.326996	211.623856	115.119019
87.340004	368.332977	87.346992	248.141617	115.076050
87.360001	409.500000	87.366989	276.404938	115.033142
87.380005	412.166992	87.386993	291.991547	114.990112
87.400002	400.500000	87.406982	292.181091	114.947144

87.419998	386.166992	87.426979	276.879669	114.904114
87.440002	364.000000	87.446983	249.353149	114.861084
87.459999	338.000000	87.466972	215.589203	114.817993
87.480003	312.832977	87.486977	181.585648	114.774902
87.500000	287.000000	87.506973	151.117310	114.731750
87.519997	259.166992	87.526962	125.220802	114.688660
87.540001	246.833008	87.546967	103.413139	114.645508
87.559998	215.333008	87.566963	85.017044	114.602295
87.580002	214.000000	87.586960	69.558762	114.559143
87.599998	187.500000	87.606956	56.741261	114.515991
87.619995	175.500000	87.626953	46.278023	114.472717
87.639999	164.667007	87.646950	37.853664	114.429504
87.659996	155.333008	87.666946	31.159906	114.386292
87.680000	149.500000	87.686951	25.931532	114.343079
87.699997	153.833008	87.706940	21.972088	114.299805
87.719994	145.667007	87.726936	19.141817	114.256470
87.739998	147.167007	87.746941	17.380116	114.213257
87.759995	146.000000	87.766930	16.710417	114.169983
87.779999	128.167007	87.786934	17.255011	114.126709
87.800003	147.667007	87.806938	19.258957	114.083435
87.820000	153.333008	87.826927	23.096416	114.040161
87.840004	151.833008	87.846931	29.273878	113.996826
87.860001	162.333008	87.866928	38.344196	113.953613
87.880005	184.000000	87.886925	50.761074	113.910278
87.900002	197.833008	87.906921	66.557541	113.867065
87.919998	202.833008	87.926918	84.890022	113.823792
87.940002	225.500000	87.946915	103.594841	113.780518
87.959999	240.500000	87.966911	119.174026	113.737305
87.980003	250.167007	87.986916	127.724159	113.693970
88.000000	246.333008	88.006905	126.763283	113.650757
88.019997	240.667007	88.026901	116.817848	113.607544
88.040001	229.667007	88.046906	101.202202	113.564331
88.059998	211.167007	88.066895	84.111771	113.521118
88.080002	193.667007	88.086899	68.577530	113.477905
88.099998	172.167007	88.106895	55.905621	113.434753
88.119995	163.833008	88.126884	46.097580	113.391602
88.139999	161.833008	88.146889	38.613575	113.348450
88.159996	158.833008	88.166885	32.894444	113.305298

88.180000	146.667007	88.186882	28.519819	113.262207
88.199997	151.667007	88.206879	25.243845	113.219116
88.219994	156.667007	88.226875	22.956524	113.176086
88.239998	148.333008	88.246872	21.652143	113.133057
88.259995	143.500000	88.266869	21.420162	113.089966
88.279999	146.667007	88.286873	22.460243	113.046997
88.300003	142.833008	88.306870	25.113449	113.004028
88.320000	154.333008	88.326866	29.908991	112.961121
88.340004	162.667007	88.346870	37.612514	112.918213
88.360001	173.167007	88.366859	49.236801	112.875366
88.380005	188.667007	88.386864	66.068054	112.832520
88.400002	212.167007	88.406860	89.490448	112.789673
88.419998	237.333008	88.426849	120.741798	112.746948
88.440002	283.500000	88.446854	160.371002	112.704224
88.459999	332.332977	88.466850	207.124847	112.661499
88.480003	389.000000	88.486847	256.859436	112.618835
88.500000	438.166992	88.506844	301.810425	112.576233
88.519997	463.000000	88.526840	331.900208	112.533630
88.540001	463.000000	88.546837	338.724304	112.491089
88.559998	448.000000	88.566833	320.368591	112.448608
88.580002	413.832977	88.586838	282.985870	112.406128
88.599998	356.000000	88.606827	237.315384	112.363770
88.619995	320.000000	88.626823	192.878296	112.321411
88.639999	276.832977	88.646828	154.902298	112.279053
88.659996	227.333008	88.666817	124.579247	112.236816
88.680000	202.167007	88.686821	100.827454	112.194580
88.699997	195.833008	88.706818	82.082237	112.152405
88.719994	185.833008	88.726807	66.980576	112.110291
88.739998	183.333008	88.746811	54.574089	112.068237
88.759995	172.667007	88.766808	44.293037	112.026245
88.779999	155.167007	88.786804	35.762745	111.984375
88.800003	151.833008	88.806808	28.723475	111.942444
88.820000	139.500000	88.826805	22.974237	111.900635
88.840004	133.667007	88.846802	18.328087	111.858887
88.860001	131.167007	88.866798	14.613852	111.817139
88.880005	120.667000	88.886803	11.671897	111.775513
88.900002	124.167000	88.906792	9.361755	111.733948
88.919998	121.833000	88.926788	7.554789	111.692444

88.940002	118.500000	88.946793	6.144866	111.651001
88.959999	118.167000	88.966782	5.045496	111.609680
88.980003	121.667000	88.986786	4.184381	111.568359
89.000000	119.833000	89.006783	3.507194	111.527100
89.019997	121.000000	89.026772	2.971012	111.485962
89.040001	117.000000	89.046776	2.542834	111.444885
89.059998	115.333000	89.066772	2.198812	111.403870
89.080002	111.167000	89.086769	1.920565	111.362915
89.099998	114.500000	89.106766	1.694536	111.322021
89.119995	115.667000	89.126762	1.510707	111.281250
89.139999	114.500000	89.146759	1.361580	111.240601
89.159996	118.167000	89.166756	1.241614	111.199951
89.180000	120.167000	89.186760	1.146739	111.159424
89.199997	125.667000	89.206749	1.074258	111.119019
89.219994	113.000000	89.226746	1.022461	111.078613
89.239998	122.000000	89.246750	0.991043	111.038330
89.259995	111.667000	89.266739	0.981416	110.998169
89.279999	118.333000	89.286743	0.997244	110.958008
89.300003	110.833000	89.306747	1.045533	110.917969
89.320000	110.833000	89.326736	1.138368	110.878052
89.340004	104.833000	89.346741	1.294699	110.838257
89.360001	117.167000	89.366737	1.542632	110.798462
89.380005	118.667000	89.386734	1.922346	110.758789
89.400002	112.167000	89.406731	2.487628	110.719238
89.419998	116.167000	89.426727	3.305159	110.679810
89.440002	118.500000	89.446724	4.447996	110.640503
89.459999	116.000000	89.466721	5.978484	110.601257
89.480003	112.500000	89.486725	7.916018	110.562073
89.500000	120.500000	89.506714	10.182993	110.523071
89.519997	120.333000	89.526711	12.559653	110.484070
89.540001	116.667000	89.546715	14.660247	110.445190
89.559998	116.500000	89.566704	16.011679	110.406494
89.580002	121.000000	89.586708	16.250277	110.367859
89.599998	111.000000	89.606697	15.323021	110.329346
89.619995	120.333000	89.626694	13.538487	110.290894
89.639999	111.833000	89.646698	11.392452	110.252563
89.659996	106.333000	89.666687	9.320604	110.214417
89.680000	119.000000	89.686691	7.553308	110.176331

89.699997	110.833000	89.706688	6.143040	110.138367
89.719994	111.667000	89.726677	5.041771	110.100525
89.739998	111.500000	89.746681	4.176044	110.062744
89.759995	110.333000	89.766678	3.486357	110.025146
89.779999	114.167000	89.786674	2.932653	109.987610
89.800003	107.500000	89.806679	2.493131	109.950195
89.820000	107.667000	89.826675	2.159096	109.913025
89.840004	106.833000	89.846672	1.929766	109.875854
89.860001	109.500000	89.866669	1.810346	109.838867
89.880005	107.167000	89.886673	1.810316	109.801941
89.900002	99.333298	89.906662	1.940838	109.765198
89.919998	110.000000	89.926659	2.210292	109.728577
89.940002	104.333000	89.946663	2.615490	109.692017
89.959999	113.167000	89.966652	3.128026	109.655701
89.980003	111.833000	89.986656	3.683028	109.619507
90.000000	113.333000	90.006653	4.173615	109.583313
90.019997	103.333000	90.026642	4.474905	109.547363
90.040001	103.667000	90.046646	4.493141	109.511536
90.059998	107.333000	90.066643	4.217001	109.475830
90.080002	108.167000	90.086639	3.727942	109.440247
90.099998	99.666695	90.106636	3.153525	109.404785
90.119995	104.667000	90.126633	2.603714	109.369507
90.139999	107.333000	90.146629	2.137021	109.334351
90.159996	107.500000	90.166626	1.765294	109.299377
90.180000	106.667000	90.186630	1.475418	109.264465
90.199997	101.333000	90.206619	1.248393	109.229736
90.219994	101.000000	90.226616	1.067578	109.195129
90.239998	107.667000	90.246620	0.921850	109.160767
90.259995	105.167000	90.266609	0.804490	109.126465
90.279999	103.167000	90.286613	0.711182	109.092285
90.300003	98.500000	90.306618	0.639356	109.058289
90.320000	98.500000	90.326607	0.587088	109.024475
90.340004	102.167000	90.346611	0.552688	108.990723
90.360001	102.500000	90.366608	0.534833	108.957214
90.380005	105.333000	90.386604	0.532375	108.923828
90.400002	100.667000	90.406601	0.544478	108.890625
90.419998	101.000000	90.426598	0.570653	108.857544
90.440002	96.833298	90.446594	0.610806	108.824646

90.459999	103.500000	90.466591	0.665246	108.791870
90.480003	104.333000	90.486595	0.734727	108.759277
90.500000	103.833000	90.506584	0.820291	108.726807
90.519997	108.167000	90.526581	0.923578	108.694580
90.540001	111.333000	90.546577	1.046584	108.662415
90.559998	104.333000	90.566574	1.191969	108.630493
90.580002	101.167000	90.586578	1.363435	108.598694
90.599998	104.167000	90.606567	1.566035	108.567078
90.619995	103.500000	90.626564	1.808057	108.535645
90.639999	115.000000	90.646568	2.102402	108.504333
90.659996	108.833000	90.666557	2.469447	108.473267
90.680000	111.500000	90.686562	2.943145	108.442261
90.699997	115.333000	90.706558	3.575864	108.411499
90.719994	114.833000	90.726547	4.449056	108.380920
90.739998	113.667000	90.746552	5.686551	108.350464
90.759995	112.667000	90.766548	7.465081	108.320190
90.779999	116.833000	90.786545	10.032630	108.290161
90.800003	116.667000	90.806549	13.717609	108.260193
90.820000	128.667007	90.826546	18.917595	108.230530
90.840004	132.000000	90.846542	26.068684	108.200928
90.860001	138.833008	90.866539	35.541954	108.171570
90.880005	158.667007	90.886543	47.462440	108.142334
90.900002	167.000000	90.906532	61.402969	108.113342
90.919998	185.667007	90.926529	76.129913	108.084534
90.940002	210.833008	90.946533	89.441849	108.055847
90.959999	207.000000	90.966522	98.557053	108.027405
90.980003	205.833008	90.986526	101.177635	107.999084
91.000000	220.500000	91.006523	96.662651	107.971008
91.019997	198.500000	91.026512	86.527138	107.943115
91.040001	179.000000	91.046516	73.602173	107.915405
91.059998	172.000000	91.066513	60.642052	107.887817
91.080002	164.000000	91.086510	49.309002	107.860535
91.099998	140.333008	91.106506	40.119991	107.833374
91.119995	139.667007	91.126503	32.885887	107.806396
91.139999	131.667007	91.146500	27.176392	107.779602
91.159996	129.833008	91.166496	22.587156	107.752991
91.180000	143.167007	91.186501	18.826323	107.726624
91.199997	115.833000	91.206490	15.715322	107.700439

91.219994	124.333000	91.226486	13.141964	107.674438
91.239998	117.167000	91.246490	11.041490	107.648621
91.259995	114.167000	91.266479	9.373887	107.623047
91.279999	109.167000	91.286484	8.108706	107.597595
91.300003	112.167000	91.306480	7.232625	107.572449
91.320000	111.333000	91.326477	6.746084	107.547363
91.340004	108.167000	91.346481	6.671163	107.522583
91.360001	117.667000	91.366470	7.056003	107.497986
91.380005	116.167000	91.386475	7.978499	107.473572
91.400002	115.500000	91.406471	9.542817	107.449341
91.419998	121.500000	91.426460	11.870356	107.425415
91.440002	122.167000	91.446465	15.086330	107.401611
91.459999	118.000000	91.466461	19.285734	107.377991
91.480003	125.500000	91.486458	24.518410	107.354614
91.500000	133.333008	91.506454	30.750429	107.331421
91.519997	150.667007	91.526451	37.795151	107.308472
91.540001	158.167007	91.546448	45.180191	107.285706
91.559998	160.833008	91.566444	52.007378	107.263184
91.580002	157.333008	91.586449	56.992321	107.240845
91.599998	167.333008	91.606438	58.849716	107.218689
91.619995	161.833008	91.626434	56.955685	107.196716
91.639999	150.667007	91.646439	51.768578	107.175049
91.659996	152.833008	91.666428	44.649796	107.153564
91.680000	147.167007	91.686432	37.132427	107.132263
91.699997	136.000000	91.706429	30.320345	107.111206
91.719994	137.500000	91.726418	24.668945	107.090332
91.739998	120.667000	91.746422	20.162436	107.069702
91.759995	121.500000	91.766418	16.592285	107.049316
91.779999	113.333000	91.786415	13.719149	107.029114
91.800003	112.500000	91.806419	11.357372	107.009094
91.820000	115.333000	91.826416	9.386265	106.989380
91.840004	110.667000	91.846413	7.729033	106.969788
91.860001	111.000000	91.866409	6.337663	106.950439
91.880005	108.167000	91.886406	5.178074	106.931335
91.900002	106.833000	91.906403	4.222124	106.912476
91.919998	111.500000	91.926399	3.443946	106.893738
91.940002	109.833000	91.946396	2.818863	106.875366
91.959999	107.667000	91.966393	2.323565	106.857056

91.980003	111.000000	91.986397	1.936329	106.839111
92.000000	104.833000	92.006386	1.638363	106.821289
92.019997	105.167000	92.026382	1.413150	106.803711
92.040001	101.500000	92.046387	1.247076	106.786377
92.059998	110.333000	92.066376	1.129602	106.769348
92.080002	106.167000	92.086380	1.052753	106.752441
92.099998	107.833000	92.106377	1.011691	106.735718
92.119995	103.833000	92.126366	1.004540	106.719299
92.139999	104.667000	92.146370	1.033130	106.703125
92.159996	107.000000	92.166367	1.103880	106.687134
92.180000	111.667000	92.186363	1.229367	106.671387
92.199997	105.667000	92.206360	1.430077	106.655884
92.219994	106.500000	92.226357	1.737291	106.640564
92.239998	102.333000	92.246353	2.195496	106.625488
92.259995	114.000000	92.266350	2.864517	106.610657
92.279999	108.333000	92.286354	3.819762	106.596069
92.300003	108.167000	92.306351	5.145839	106.581665
92.320000	112.833000	92.326347	6.924967	106.567566
92.340004	106.167000	92.346352	9.209858	106.553650
92.360001	106.500000	92.366341	11.977131	106.539978
92.380005	117.500000	92.386345	15.086585	106.526489
92.400002	120.167000	92.406334	18.224428	106.513306
92.419998	122.833000	92.426331	20.927084	106.500305
92.440002	118.833000	92.446335	22.662813	106.487549
92.459999	128.500000	92.466324	23.021959	106.475098
92.480003	130.167007	92.486328	21.920242	106.462769
92.500000	123.167000	92.506325	19.668381	106.450806
92.519997	129.000000	92.526314	16.830961	106.438965
92.540001	120.000000	92.546318	13.960768	106.427368
92.559998	114.833000	92.566315	11.417888	106.416016
92.580002	113.500000	92.586311	9.327690	106.404907
92.599998	107.167000	92.606308	7.663198	106.394043
92.619995	109.333000	92.626305	6.337420	106.383362
92.639999	105.000000	92.646301	5.262536	106.373047
92.659996	108.667000	92.666298	4.372396	106.362854
92.680000	102.500000	92.686302	3.624209	106.352905
92.699997	96.500000	92.706291	2.992028	106.343262
92.719994	106.667000	92.726288	2.458790	106.333801

92.739998	108.667000	92.746292	2.012386	106.324585
92.759995	104.333000	92.766281	1.643142	106.315613
92.779999	106.333000	92.786285	1.340984	106.306885
92.800003	100.500000	92.806290	1.097018	106.298401
92.820000	100.000000	92.826279	0.902576	106.290161
92.840004	95.833298	92.846283	0.749213	106.282104
92.860001	108.833000	92.866272	0.629872	106.274292
92.880005	106.167000	92.886276	0.538063	106.266785
92.900002	109.667000	92.906273	0.468623	106.259460
92.919998	108.667000	92.926262	0.417243	106.252380
92.940002	112.167000	92.946266	0.380492	106.245544
92.959999	101.000000	92.966263	0.355929	106.238953
92.980003	109.000000	92.986259	0.341426	106.232666
93.000000	104.000000	93.006256	0.335713	106.226501
93.019997	106.000000	93.026253	0.337874	106.220642
93.040001	102.500000	93.046249	0.347377	106.215027
93.059998	109.500000	93.066246	0.364039	106.209595
93.080002	107.000000	93.086250	0.388063	106.204468
93.099998	109.167000	93.106239	0.420087	106.199585
93.119995	110.500000	93.126236	0.461513	106.194885
93.139999	106.500000	93.146240	0.514802	106.190430
93.159996	106.167000	93.166229	0.584022	106.186279
93.180000	108.500000	93.186234	0.676016	106.182312
93.199997	112.500000	93.206230	0.801309	106.178589
93.219994	109.833000	93.226219	0.976071	106.175110
93.239998	109.500000	93.246223	1.224545	106.171875
93.259995	114.667000	93.266212	1.580619	106.168884
93.279999	114.500000	93.286217	2.091862	106.166138
93.300003	109.000000	93.306221	2.819299	106.163574
93.320000	110.167000	93.326210	3.836270	106.161316
93.340004	112.833000	93.346214	5.224694	106.159241
93.360001	114.500000	93.366211	7.053138	106.157471
93.380005	116.167000	93.386208	9.350783	106.155884
93.400002	112.667000	93.406204	12.059072	106.154602
93.419998	124.500000	93.426201	14.980700	106.153503
93.440002	124.500000	93.446198	17.752430	106.152649
93.459999	129.667007	93.466194	19.889496	106.152039
93.480003	126.833000	93.486198	20.932405	106.151672

93.500000	135.000000	93.506187	20.650084	106.151489
93.519997	128.500000	93.526184	19.169468	106.151611
93.540001	123.667000	93.546188	16.911362	106.151978
93.559998	124.500000	93.566177	14.385385	106.152527
93.580002	120.500000	93.586182	11.980311	106.153320
93.599998	115.000000	93.606178	9.901855	106.154419
93.619995	108.500000	93.626167	8.196497	106.155701
93.639999	108.667000	93.646172	6.823551	106.157227
93.659996	117.167000	93.666161	5.718215	106.158997
93.680000	113.833000	93.686165	4.815790	106.160950
93.699997	112.833000	93.706161	4.070037	106.163147
93.719994	112.500000	93.726151	3.449267	106.165649
93.739998	109.500000	93.746155	2.933140	106.168335
93.759995	106.333000	93.766151	2.510095	106.171265
93.779999	111.500000	93.786148	2.172166	106.174438
93.800003	111.667000	93.806152	1.914557	106.177856
93.820000	106.167000	93.826149	1.735817	106.181458
93.840004	106.667000	93.846146	1.637842	106.185303
93.860001	102.333000	93.866142	1.628504	106.189453
93.880005	110.833000	93.886147	1.723438	106.193787
93.900002	109.500000	93.906136	1.948295	106.198303
93.919998	108.167000	93.926132	2.341654	106.203125
93.940002	101.333000	93.946136	2.956106	106.208130
93.959999	103.000000	93.966125	3.855850	106.213379
93.980003	107.333000	93.986130	5.113957	106.218872
94.000000	117.333000	94.006126	6.792227	106.224609
94.019997	108.500000	94.026115	8.916499	106.230469
94.040001	113.500000	94.046120	11.436865	106.236694
94.059998	108.167000	94.066109	14.169786	106.243042
94.080002	117.167000	94.086113	16.783838	106.249695
94.099998	124.167000	94.106110	18.819067	106.256531
94.119995	123.500000	94.126099	19.828173	106.263611
94.139999	121.500000	94.146103	19.565699	106.270874
94.159996	115.000000	94.166100	18.126875	106.278442
94.180000	117.167000	94.186096	15.913315	106.286194
94.199997	111.667000	94.206093	13.434546	106.294128
94.219994	114.167000	94.226089	11.096292	106.302368
94.239998	109.667000	94.246086	9.105508	106.310791

94.259995	110.333000	94.266083	7.498763	106.319458
94.279999	106.833000	94.286087	6.220961	106.328247
94.300003	105.667000	94.306084	5.194286	106.337341
94.320000	110.333000	94.326080	4.350307	106.346680
94.340004	110.500000	94.346085	3.641490	106.356201
94.360001	107.500000	94.366074	3.039251	106.365967
94.380005	107.833000	94.386078	2.525175	106.375854
94.400002	105.000000	94.406067	2.089022	106.386047
94.419998	108.000000	94.426064	1.722133	106.396484
94.440002	103.667000	94.446068	1.417495	106.407104
94.459999	102.500000	94.466057	1.168625	106.417908
94.480003	106.833000	94.486061	0.968516	106.428955
94.500000	104.167000	94.506058	0.811137	106.440186
94.519997	104.167000	94.526047	0.690718	106.451660
94.540001	98.500000	94.546051	0.602104	106.463318
94.559998	97.666695	94.566048	0.541045	106.475220
94.580002	103.833000	94.586044	0.503553	106.487305
94.599998	96.333298	94.606041	0.485636	106.499634
94.619995	106.167000	94.626038	0.482648	106.512146
94.639999	101.333000	94.646034	0.488812	106.524841
94.659996	101.833000	94.666031	0.497335	106.537781
94.680000	98.333298	94.686035	0.501581	106.550964
94.699997	100.000000	94.706024	0.497154	106.564331
94.719994	106.333000	94.726021	0.483777	106.577881
94.739998	103.500000	94.746017	0.465470	106.591675
94.759995	98.166695	94.766014	0.448737	106.605591
94.779999	105.000000	94.786018	0.440151	106.619751
94.800003	96.333298	94.806015	0.445079	106.634094
94.820000	97.833298	94.826012	0.467961	106.648682
94.840004	101.667000	94.846016	0.513625	106.663452
94.860001	105.500000	94.866005	0.588079	106.678345
94.880005	102.667000	94.886009	0.700013	106.693604
94.900002	105.833000	94.906006	0.860233	106.708923
94.919998	103.500000	94.925995	1.081012	106.724487
94.940002	102.667000	94.945999	1.374530	106.740234
94.959999	112.333000	94.965996	1.747640	106.756165
94.980003	112.000000	94.985992	2.196494	106.772339
95.000000	115.167000	95.005989	2.698145	106.788574

95.019997	108.500000	95.025978	3.204319	106.805115
95.040001	111.000000	95.045982	3.644228	106.821899
95.059998	111.833000	95.065979	3.939095	106.838745
95.080002	114.333000	95.085976	4.033895	106.855835
95.099998	110.667000	95.105972	3.924957	106.873108
95.119995	111.000000	95.125969	3.665622	106.890503
95.139999	114.500000	95.145966	3.341771	106.908142
95.159996	112.833000	95.165962	3.035135	106.925964
95.180000	101.167000	95.185966	2.799562	106.943970
95.199997	107.833000	95.205956	2.660059	106.962158
95.219994	108.167000	95.225952	2.624851	106.980530
95.239998	106.667000	95.245956	2.701035	106.999084
95.259995	111.667000	95.265945	2.905604	107.017822
95.279999	105.167000	95.285950	3.272495	107.036743
95.300003	111.667000	95.305954	3.856886	107.055786
95.320000	105.333000	95.325943	4.738299	107.075073
95.340004	109.833000	95.345947	6.025521	107.094482
95.360001	114.167000	95.365936	7.849631	107.114014
95.380005	106.167000	95.385941	10.363218	107.133789
95.400002	112.167000	95.405937	13.706379	107.153748
95.419998	123.833000	95.425926	17.968954	107.173828
95.440002	127.333000	95.445930	23.124146	107.194092
95.459999	132.333008	95.465927	28.923479	107.214600
95.480003	143.167007	95.485924	34.846512	107.235107
95.500000	143.333008	95.505920	40.113014	107.255920
95.519997	151.000000	95.525917	43.868591	107.276855
95.540001	150.167007	95.545914	45.522839	107.297913
95.559998	150.667007	95.565910	45.076424	107.319153
95.580002	140.333008	95.585915	43.186588	107.340576
95.599998	147.333008	95.605904	40.877460	107.362122
95.619995	144.333008	95.625900	39.060654	107.383850
95.639999	149.333008	95.645897	38.189873	107.405762
95.659996	146.500000	95.665894	38.160854	107.427734
95.680000	146.833008	95.685898	38.432228	107.449951
95.699997	145.667007	95.705887	38.271091	107.472290
95.719994	141.333008	95.725883	37.055817	107.494690
95.739998	137.000000	95.745888	34.538467	107.517334
95.759995	142.333008	95.765877	30.943575	107.540100

95.779999	136.000000	95.785881	26.810202	107.562988
95.800003	129.500000	95.805885	22.730843	107.586060
95.820000	127.833000	95.825867	19.122234	107.609253
95.840004	118.000000	95.845871	16.150444	107.632568
95.860001	120.333000	95.865868	13.819293	107.656067
95.880005	122.333000	95.885864	12.048482	107.679688
95.900002	131.167007	95.905861	10.755012	107.703491
95.919998	122.333000	95.925850	9.889134	107.727295
95.940002	128.500000	95.945854	9.444956	107.751343
95.959999	134.167007	95.965851	9.462437	107.775452
95.980003	126.500000	95.985847	10.020676	107.799744
96.000000	131.333008	96.005844	11.232542	107.824158
96.019997	123.667000	96.025841	13.232399	107.848755
96.040001	137.333008	96.045837	16.152357	107.873413
96.059998	133.500000	96.065834	20.078838	107.898193
96.080002	138.667007	96.085838	24.985865	107.923096
96.099998	146.500000	96.105827	30.635826	107.948120
96.119995	148.500000	96.125824	36.520832	107.973328
96.139999	157.667007	96.145828	41.838150	107.998596
96.159996	154.167007	96.165817	45.636143	108.023926
96.180000	162.500000	96.185822	47.145157	108.049438
96.199997	160.333008	96.205811	46.119911	108.075073
96.219994	162.500000	96.225807	43.015350	108.100769
96.239998	158.000000	96.245811	38.800861	108.126709
96.259995	147.167007	96.265800	34.558582	108.152588
96.279999	152.167007	96.285805	31.114985	108.178711
96.300003	145.167007	96.305809	28.954140	108.204834
96.320000	145.000000	96.325798	28.300632	108.231079
96.340004	145.500000	96.345802	29.281727	108.257568
96.360001	150.000000	96.365799	32.044594	108.283997
96.380005	151.167007	96.385796	36.791817	108.310608
96.400002	165.833008	96.405792	43.743008	108.337280
96.419998	173.667007	96.425789	53.038738	108.364014
96.440002	189.500000	96.445786	64.611168	108.390869
96.459999	193.167007	96.465782	78.070526	108.417847
96.480003	218.167007	96.485779	92.690163	108.444946
96.500000	233.500000	96.505775	107.567360	108.472046
96.519997	253.833008	96.525772	121.931961	108.499268

96.540001	275.166992	96.545769	135.389725	108.526611
96.559998	280.500000	96.565765	147.824158	108.554077
96.580002	292.332977	96.585770	158.948715	108.581543
96.599998	289.000000	96.605759	167.896667	108.609070
96.619995	293.500000	96.625755	173.419601	108.636719
96.639999	285.832977	96.645760	174.553329	108.664490
96.659996	291.666992	96.665749	171.276016	108.692322
96.680000	295.500000	96.685753	164.434448	108.720154
96.699997	287.000000	96.705750	155.061157	108.748169
96.719994	280.166992	96.725739	143.744217	108.776123
96.739998	279.000000	96.745743	130.646759	108.804260
96.759995	255.500000	96.765732	116.068779	108.832397
96.779999	245.333008	96.785736	100.737732	108.860657
96.800003	222.167007	96.805740	85.748474	108.888977
96.820000	200.167007	96.825729	72.091942	108.917358
96.840004	182.000000	96.845734	60.299702	108.945801
96.860001	171.833008	96.865730	50.480576	108.974243
96.880005	161.000000	96.885727	42.433010	109.002808
96.900002	154.167007	96.905724	35.861622	109.031433
96.919998	152.667007	96.925720	30.496223	109.060059
96.940002	145.500000	96.945717	26.140932	109.088745
96.959999	142.667007	96.965714	22.677464	109.117493
96.980003	141.667007	96.985710	20.050627	109.146362
97.000000	143.167007	97.005707	18.251085	109.175171
97.019997	146.000000	97.025703	17.299906	109.204102
97.040001	134.667007	97.045700	17.233122	109.233032
97.059998	127.333000	97.065697	18.082331	109.262085
97.080002	138.500000	97.085701	19.848022	109.291138
97.099998	130.167007	97.105690	22.458158	109.320190
97.119995	145.667007	97.125687	25.731831	109.349243
97.139999	147.667007	97.145691	29.330185	109.378418
97.159996	155.167007	97.165680	32.738045	109.407593
97.180000	152.333008	97.185684	35.328251	109.436829
97.199997	148.667007	97.205681	36.495750	109.466064
97.219994	148.500000	97.225670	35.897552	109.495300
97.239998	142.833008	97.245674	33.622585	109.524658
97.259995	144.333008	97.265663	30.186403	109.553955
97.279999	141.833008	97.285667	26.295650	109.583252

97.300003	136.000000	97.305672	22.583328	109.612671
97.320000	134.500000	97.325661	19.435999	109.642029
97.340004	130.333008	97.345665	16.987957	109.671448
97.360001	125.833000	97.365662	15.234063	109.700867
97.380005	121.667000	97.385658	14.124060	109.730286
97.400002	128.167007	97.405655	13.639427	109.759705
97.419998	124.333000	97.425652	13.826086	109.789185
97.440002	123.667000	97.445648	14.803743	109.818604
97.459999	133.833008	97.465645	16.761992	109.848083
97.480003	129.833008	97.485641	19.946106	109.877625
97.500000	137.000000	97.505638	24.627449	109.907043
97.519997	140.667007	97.525635	31.047310	109.936523
97.540001	151.333008	97.545631	39.321541	109.966003
97.559998	161.833008	97.565628	49.300819	109.995483
97.580002	169.667007	97.585632	60.411186	110.024963
97.599998	167.000000	97.605621	71.513565	110.054443
97.619995	181.500000	97.625618	80.996002	110.083862
97.639999	188.167007	97.645622	87.070755	110.113342
97.659996	190.833008	97.665611	88.401146	110.142761
97.680000	182.500000	97.685616	84.721222	110.172180
97.699997	182.167007	97.705605	77.021713	110.201599
97.719994	168.500000	97.725601	67.105629	110.231018
97.739998	158.500000	97.745605	56.832920	110.260376
97.759995	150.000000	97.765594	47.517467	110.289734
97.779999	146.000000	97.785599	39.717255	110.319031
97.800003	147.333008	97.805603	33.459637	110.348389
97.820000	132.833008	97.825592	28.498066	110.377625
97.840004	130.000000	97.845596	24.525423	110.406921
97.860001	125.500000	97.865593	21.307697	110.436157
97.880005	126.667000	97.885590	18.695400	110.465271
97.900002	123.833000	97.905586	16.622992	110.494446
97.919998	122.333000	97.925575	15.088202	110.523560
97.940002	127.167000	97.945580	14.132455	110.552612
97.959999	125.667000	97.965576	13.833618	110.581726
97.980003	131.667007	97.985573	14.291677	110.610718
98.000000	124.667000	98.005569	15.618840	110.639709
98.019997	130.500000	98.025566	17.916925	110.668579
98.040001	146.500000	98.045563	21.238407	110.697449

98.059998	143.167007	98.065559	25.526451	110.726257
98.080002	143.667007	98.085564	30.542004	110.755005
98.099998	150.333008	98.105553	35.789955	110.783813
98.119995	156.333008	98.125549	40.538582	110.812439
98.139999	164.333008	98.145546	43.923206	110.841064
98.159996	164.000000	98.165543	45.218731	110.869629
98.180000	168.833008	98.185547	44.142349	110.898132
98.199997	163.333008	98.205536	41.016285	110.926514
98.219994	150.833008	98.225533	36.634781	110.954956
98.239998	154.333008	98.245537	31.932772	110.983276
98.259995	143.000000	98.265526	27.664711	111.011475
98.279999	129.167007	98.285530	24.249002	111.039673
98.300003	136.833008	98.305534	21.842064	111.067810
98.320000	140.833008	98.325523	20.462761	111.095825
98.340004	126.167000	98.345528	20.118378	111.123779
98.360001	132.000000	98.365517	20.885033	111.151672
98.380005	133.833008	98.385521	22.932510	111.179504
98.400002	142.667007	98.405518	26.511948	111.207214
98.419998	149.167007	98.425507	31.920168	111.234863
98.440002	155.167007	98.445511	39.437538	111.262451
98.459999	173.667007	98.465508	49.187954	111.289917
98.480003	179.667007	98.485504	60.999477	111.317383
98.500000	180.833008	98.505501	74.203003	111.344604
98.519997	211.167007	98.525497	87.496941	111.371887
98.540001	217.333008	98.545494	98.996040	111.398987
98.559998	227.833008	98.565491	106.602913	111.426025
98.580002	223.167007	98.585487	108.696815	111.453003
98.599998	226.333008	98.605484	104.849411	111.479858
98.619995	212.500000	98.625481	96.099159	111.506592
98.639999	202.333008	98.645477	84.525421	111.533203
98.659996	180.167007	98.665474	72.376259	111.559814
98.680000	171.167007	98.685478	61.307861	111.586304
98.699997	162.667007	98.705467	52.125404	111.612610
98.719994	154.333008	98.725464	44.893429	111.638855
98.739998	139.667007	98.745468	39.285095	111.664978
98.759995	137.333008	98.765457	34.839409	111.690979
98.779999	147.333008	98.785461	31.103018	111.716919
98.800003	144.500000	98.805458	27.731352	111.742737

98.820000	136.667007	98.825455	24.506611	111.768433
98.840004	144.333008	98.845459	21.352583	111.793945
98.860001	135.833008	98.865448	18.313583	111.819458
98.880005	131.833008	98.885452	15.484801	111.844788
98.900002	134.000000	98.905449	12.965358	111.869995
98.919998	126.667000	98.925438	10.806943	111.895081
98.940002	115.500000	98.945442	9.009078	111.920044
98.959999	117.333000	98.965439	7.540590	111.944885
98.980003	114.333000	98.985435	6.351954	111.969604
99.000000	118.833000	99.005432	5.393580	111.994141
99.019997	118.833000	99.025421	4.622651	112.018555
99.040001	114.667000	99.045425	4.003734	112.042969
99.059998	111.167000	99.065422	3.510744	112.067139
99.080002	114.667000	99.085419	3.122759	112.091187
99.099998	112.667000	99.105415	2.823797	112.115051
99.119995	107.833000	99.125412	2.601354	112.138855
99.139999	110.167000	99.145409	2.445541	112.162476
99.159996	112.167000	99.165405	2.348542	112.185974
99.180000	107.167000	99.185410	2.304302	112.209290
99.199997	114.500000	99.205399	2.308481	112.232544
99.219994	108.833000	99.225395	2.358544	112.255554
99.239998	118.500000	99.245392	2.454302	112.278442
99.259995	106.833000	99.265388	2.598754	112.301270
99.279999	110.833000	99.285393	2.799623	112.323853
99.300003	117.167000	99.305389	3.071234	112.346313
99.320000	112.000000	99.325386	3.438146	112.368591
99.340004	108.167000	99.345390	3.939487	112.390747
99.360001	121.333000	99.365379	4.633697	112.412781
99.380005	114.000000	99.385384	5.608473	112.434631
99.400002	116.333000	99.405373	6.984805	112.456238
99.419998	123.500000	99.425369	8.931473	112.477783
99.440002	121.500000	99.445374	11.668602	112.499084
99.459999	132.500000	99.465363	15.466734	112.520325
99.480003	133.500000	99.485367	20.651733	112.541260
99.500000	140.333008	99.505363	27.554949	112.562134
99.519997	138.333008	99.525352	36.465290	112.582825
99.540001	161.500000	99.545357	47.533081	112.603394
99.559998	168.833008	99.565353	60.579216	112.623718

99.580002	195.167007	99.585350	74.954185	112.643860
99.599998	210.500000	99.605347	89.386147	112.663879
99.619995	222.667007	99.625336	102.017143	112.683716
99.639999	233.333008	99.645340	110.759140	112.703369
99.659996	241.000000	99.665337	113.893181	112.722778
99.680000	234.000000	99.685333	110.818474	112.742065
99.699997	232.000000	99.705330	102.374283	112.761230
99.719994	220.333008	99.725327	90.529678	112.780151
99.739998	199.833008	99.745323	77.575531	112.798889
99.759995	179.000000	99.765320	65.333321	112.817444
99.779999	164.500000	99.785324	54.784180	112.835815
99.800003	152.667007	99.805321	46.168224	112.854004
99.820000	142.833008	99.825317	39.263611	112.872009
99.840004	142.500000	99.845314	33.695564	112.889771
99.860001	144.000000	99.865311	29.111185	112.907471
99.880005	133.333008	99.885315	25.248194	112.924866
99.900002	131.500000	99.905304	21.945803	112.942139
99.919998	131.500000	99.925301	19.108622	112.959167
99.940002	131.667007	99.945305	16.693771	112.976013
99.959999	128.167007	99.965294	14.689265	112.992676
99.980003	137.333008	99.985291	13.096669	113.009094
100.000000	125.333000	100.005280	11.937228	113.025391
100.019997	125.000000	100.025276	11.248948	113.041443
100.040001	133.167007	100.045280	11.097094	113.057312
100.059998	123.500000	100.065269	11.580246	113.072937
100.080002	126.667000	100.085274	12.840920	113.088440
100.099998	120.500000	100.105270	15.068745	113.103699
100.119995	130.500000	100.125259	18.500992	113.118652
100.139999	130.333008	100.145264	23.414459	113.133545
100.159996	137.833008	100.165260	30.072872	113.148193
100.180000	151.000000	100.185257	38.671627	113.162598
100.199997	167.833008	100.205254	49.216965	113.176819
100.219994	187.167007	100.225243	61.372986	113.190796
100.239998	197.833008	100.245247	74.336220	113.204590
100.259995	206.167007	100.265244	86.719635	113.218262
100.279999	212.167007	100.285240	96.735786	113.231567
100.300003	220.333008	100.305244	102.591225	113.244751
100.320000	222.000000	100.325241	103.108360	113.257690

100.340004	227.500000	100.345238	98.270836	113.270386
100.360001	208.500000	100.365234	89.287239	113.282898
100.380005	183.167007	100.385231	78.105942	113.295227
100.400002	179.167007	100.405228	66.661171	113.307312
100.419998	168.333008	100.425224	56.303280	113.319153
100.440002	154.667007	100.445221	47.644459	113.330811
100.459999	146.000000	100.465218	40.732300	113.342285
100.480003	134.667007	100.485222	35.329109	113.353455
100.500000	139.667007	100.505211	31.148203	113.364441
100.519997	140.667007	100.525208	27.959450	113.375183
100.540001	138.667007	100.545204	25.647511	113.385742
100.559998	133.833008	100.565201	24.196554	113.396057
100.580002	143.667007	100.585205	23.666700	113.406128
100.599998	143.333008	100.605194	24.162374	113.416016
100.619995	133.500000	100.625191	25.795231	113.425659
100.639999	152.500000	100.645195	28.639620	113.435059
100.659996	152.333008	100.665184	32.667439	113.444214
100.680000	152.500000	100.685188	37.697243	113.453186
100.699997	158.667007	100.705185	43.319035	113.461914
100.719994	159.333008	100.725174	48.908466	113.470337
100.739998	165.500000	100.745178	53.720253	113.478638
100.759995	165.333008	100.765167	57.061993	113.486694
100.779999	174.167007	100.785172	58.543121	113.494446
100.800003	164.500000	100.805176	58.172550	113.502014
100.820000	170.667007	100.825165	56.288345	113.509338
100.840004	163.667007	100.845169	53.317627	113.516479
100.860001	158.833008	100.865166	49.590805	113.523376
100.880005	160.500000	100.885162	45.302933	113.529968
100.900002	153.000000	100.905159	40.622490	113.536438
100.919998	149.167007	100.925148	35.788925	113.542603
100.940002	134.667007	100.945152	31.097124	113.548523
100.959999	140.000000	100.965149	26.831551	113.554199
100.980003	132.833008	100.985146	23.171537	113.559692
101.000000	129.667007	101.005142	20.185064	113.564941
101.019997	128.833008	101.025139	17.863190	113.569885
101.040001	123.833000	101.045135	16.171188	113.574707
101.059998	136.500000	101.065132	15.086675	113.579163
101.080002	129.167007	101.085129	14.616901	113.583496

101.099998	124.500000	101.105125	14.796839	113.587524
101.119995	124.833000	101.125122	15.674216	113.591309
101.139999	118.833000	101.145119	17.282719	113.594910
101.159996	129.500000	101.165115	19.604271	113.598267
101.180000	133.833008	101.185120	22.524269	113.601318
101.199997	132.500000	101.205109	25.781948	113.604187
101.219994	135.500000	101.225105	28.963877	113.606812
101.239998	140.667007	101.245110	31.532381	113.609131
101.259995	143.000000	101.265099	32.946438	113.611328
101.279999	138.833008	101.285103	32.845383	113.613159
101.300003	139.167007	101.305099	31.195599	113.614929
101.320000	135.833008	101.325096	28.323250	113.616333
101.340004	135.000000	101.345100	24.780693	113.617493
101.360001	128.833008	101.365089	21.139612	113.618408
101.380005	124.333000	101.385094	17.803995	113.619141
101.400002	130.833008	101.405090	14.971280	113.619629
101.419998	118.000000	101.425079	12.659935	113.619812
101.440002	112.500000	101.445084	10.791093	113.619812
101.459999	118.500000	101.465073	9.263720	113.619507
101.480003	118.000000	101.485077	7.983050	113.619019
101.500000	109.500000	101.505074	6.883669	113.618286
101.519997	112.167000	101.525063	5.922864	113.617310
101.540001	110.167000	101.545067	5.075024	113.616150
101.559998	106.833000	101.565063	4.327537	113.614624
101.580002	114.333000	101.585060	3.671906	113.612915
101.599998	115.167000	101.605057	3.101998	113.611023
101.619995	108.333000	101.625046	2.612046	113.608887
101.639999	114.167000	101.645050	2.195042	113.606445
101.659996	115.333000	101.665047	1.844375	113.603760
101.680000	115.667000	101.685043	1.552356	113.600891
101.699997	118.500000	101.705040	1.311391	113.597778
101.719994	103.000000	101.725037	1.114150	113.594421
101.739998	105.667000	101.745033	0.953817	113.590820
101.759995	109.667000	101.765030	0.824256	113.586975
101.779999	109.667000	101.785027	0.720112	113.582886
101.800003	107.167000	101.805031	0.636741	113.578613
101.820000	105.833000	101.825027	0.570451	113.574036
101.840004	107.500000	101.845024	0.518041	113.569275

101.860001	110.000000	101.865021	0.476997	113.564209
101.880005	106.167000	101.885025	0.445307	113.558960
101.900002	111.167000	101.905014	0.421452	113.553528
101.919998	110.000000	101.925011	0.404216	113.547791
101.940002	112.667000	101.945007	0.392718	113.541870
101.959999	110.667000	101.965004	0.386311	113.535645
101.980003	114.500000	101.985008	0.384546	113.529236
102.000000	111.500000	102.004997	0.387133	113.522583
102.019997	111.333000	102.024994	0.393918	113.515686
102.040001	107.500000	102.044998	0.404867	113.508606
102.059998	111.667000	102.064987	0.420027	113.501282
102.080002	119.667000	102.084991	0.439573	113.493652
102.099998	103.167000	102.104980	0.463712	113.485901
102.119995	101.333000	102.124977	0.492786	113.477844
102.139999	109.667000	102.144981	0.527197	113.469604
102.159996	112.500000	102.164970	0.567378	113.461121
102.180000	105.833000	102.184975	0.613947	113.452393
102.199997	108.667000	102.204971	0.667499	113.443481
102.219994	106.833000	102.224960	0.728772	113.434326
102.239998	104.333000	102.244965	0.798713	113.424927
102.259995	112.833000	102.264954	0.878247	113.415283
102.279999	113.333000	102.284958	0.968766	113.405457
102.300003	114.333000	102.304962	1.071826	113.395447
102.320000	108.833000	102.324951	1.189452	113.385193
102.340004	114.833000	102.344955	1.324705	113.374634
102.360001	108.833000	102.364952	1.481446	113.364014
102.380005	115.500000	102.384949	1.665375	113.353027
102.400002	117.333000	102.404945	1.884498	113.341858
102.419998	120.333000	102.424934	2.149918	113.330505
102.440002	112.500000	102.444939	2.477991	113.318909
102.459999	105.500000	102.464935	2.890137	113.307129
102.480003	117.667000	102.484932	3.416059	113.295166
102.500000	117.000000	102.504929	4.094895	113.282959
102.519997	121.000000	102.524925	4.976861	113.270508
102.540001	113.667000	102.544922	6.123897	113.257874
102.559998	117.333000	102.564919	7.608413	113.244995
102.580002	118.833000	102.584915	9.508668	113.231995
102.599998	125.833000	102.604912	11.899132	113.218750

102.619995	130.167007	102.624908	14.834580	113.205261
102.639999	127.167000	102.644905	18.328907	113.191589
102.659996	137.500000	102.664902	22.334421	113.177734
102.680000	137.500000	102.684906	26.736357	113.163635
102.699997	149.833008	102.704895	31.369831	113.149353
102.719994	150.167007	102.724892	36.111187	113.134888
102.739998	160.833008	102.744888	40.975315	113.120178
102.759995	161.500000	102.764885	46.232693	113.105286
102.779999	168.000000	102.784889	52.439133	113.090210
102.800003	172.000000	102.804886	60.323227	113.075012
102.820000	181.500000	102.824883	70.578232	113.059509
102.840004	185.667007	102.844887	83.585625	113.043884
102.860001	215.500000	102.864876	99.136147	113.028015
102.880005	223.500000	102.884872	116.302315	113.011963
102.900002	251.167007	102.904861	133.324066	112.995728
102.919998	270.332977	102.924858	147.832581	112.979370
102.940002	289.666992	102.944862	157.288712	112.962769
102.959999	286.000000	102.964851	159.748169	112.945984
102.980003	283.832977	102.984856	154.616425	112.929016
103.000000	262.000000	103.004852	142.946777	112.911926
103.019997	235.500000	103.024841	127.072823	112.894592
103.040001	225.500000	103.044846	109.700897	112.877075
103.059998	207.167007	103.064835	93.103279	112.859436
103.080002	189.167007	103.084839	78.565102	112.841614
103.099998	173.667007	103.104836	66.507011	112.823608
103.119995	162.500000	103.124825	56.730976	112.805359
103.139999	158.833008	103.144829	48.775181	112.786987
103.159996	148.167007	103.164825	42.179810	112.768494
103.180000	140.667007	103.184822	36.566895	112.749756
103.199997	152.000000	103.204819	31.681982	112.730957
103.219994	139.667007	103.224808	27.373161	112.711914
103.239998	143.000000	103.244812	23.550978	112.692749
103.259995	139.333008	103.264809	20.174454	112.673340
103.279999	134.833008	103.284805	17.214380	112.653870
103.300003	129.833008	103.304810	14.647409	112.634216
103.320000	130.500000	103.324806	12.451674	112.614380
103.340004	118.333000	103.344803	10.598819	112.594421
103.360001	120.667000	103.364799	9.058237	112.574341

103.380005	123.500000	103.384796	7.797335	112.554016
103.400002	130.000000	103.404793	6.783051	112.533569
103.419998	118.000000	103.424789	5.983737	112.513062
103.440002	129.500000	103.444786	5.369816	112.492371
103.459999	123.500000	103.464783	4.915003	112.471497
103.480003	124.500000	103.484787	4.596689	112.450500
103.500000	110.667000	103.504776	4.396890	112.429382
103.519997	121.500000	103.524773	4.301610	112.408081
103.540001	113.833000	103.544769	4.302447	112.386719
103.559998	110.333000	103.564766	4.396854	112.365173
103.580002	117.333000	103.584770	4.589922	112.343506
103.599998	122.833000	103.604759	4.895882	112.321716
103.619995	113.833000	103.624756	5.342105	112.299805
103.639999	116.333000	103.644760	5.972509	112.277832
103.659996	130.000000	103.664749	6.851755	112.255615
103.680000	122.500000	103.684753	8.074685	112.233276
103.699997	131.667007	103.704742	9.767439	112.210938
103.719994	127.167000	103.724739	12.100673	112.188416
103.739998	138.667007	103.744743	15.289505	112.165771
103.759995	128.000000	103.764732	19.589016	112.143066
103.779999	151.167007	103.784737	25.302240	112.120239
103.800003	141.167007	103.804733	32.737347	112.097168
103.820000	150.667007	103.824730	42.193764	112.074097
103.840004	160.500000	103.844734	53.908672	112.050964
103.860001	185.167007	103.864723	67.971794	112.027710
103.880005	197.000000	103.884727	84.306313	112.004272
103.900002	219.500000	103.904724	102.503647	111.980835
103.919998	232.333008	103.924713	121.768829	111.957275
103.940002	258.000000	103.944717	140.818146	111.933594
103.959999	274.500000	103.964706	157.771057	111.909912
103.980003	297.000000	103.984711	170.430801	111.886047
104.000000	292.666992	104.004707	176.679626	111.862122
104.019997	299.166992	104.024696	175.258987	111.838135
104.040001	287.166992	104.044701	166.325317	111.814026
104.059998	276.166992	104.064697	151.554733	111.789917
104.080002	252.833008	104.084694	133.560013	111.765625
104.099998	238.167007	104.104691	115.002777	111.741333
104.119995	221.167007	104.124680	97.857803	111.716919

104.139999	185.500000	104.144684	83.103256	111.692505
104.159996	184.333008	104.164680	70.926697	111.667969
104.180000	164.833008	104.184677	61.002163	111.643372
104.199997	162.833008	104.204674	52.838562	111.618713
104.219994	163.000000	104.224670	45.974297	111.593994
104.239998	151.333008	104.244667	40.060822	111.569214
104.259995	155.000000	104.264664	34.870049	111.544434
104.279999	153.333008	104.284660	30.267963	111.519531
104.300003	144.667007	104.304665	26.180052	111.494629
104.320000	136.000000	104.324661	22.569422	111.469604
104.340004	141.833008	104.344658	19.408539	111.444641
104.360001	138.000000	104.364655	16.674553	111.419556
104.380005	128.000000	104.384651	14.342411	111.394470
104.400002	132.333008	104.404648	12.383451	111.369324
104.419998	129.500000	104.424644	10.766187	111.344177
104.440002	119.500000	104.444641	9.458349	111.318970
104.459999	123.333000	104.464638	8.429430	111.293701
104.480003	128.333008	104.484642	7.653224	111.268433
104.500000	131.000000	104.504631	7.112366	111.243164
104.519997	120.500000	104.524628	6.798953	111.217896
104.540001	112.833000	104.544624	6.721180	111.192505
104.559998	121.833000	104.564621	6.906664	111.167175
104.580002	124.000000	104.584625	7.408644	111.141846
104.599998	124.000000	104.604614	8.310757	111.116455
104.619995	117.667000	104.624611	9.736658	111.091064
104.639999	127.333000	104.644615	11.854735	111.065674
104.659996	125.833000	104.664604	14.878789	111.040283
104.680000	129.667007	104.684608	19.078299	111.014893
104.699997	126.833000	104.704597	24.748280	110.989502
104.719994	144.667007	104.724594	32.204582	110.964111
104.739998	151.333008	104.744598	41.714649	110.938721
104.759995	156.167007	104.764587	53.399723	110.913330
104.779999	167.333008	104.784592	67.157005	110.887939
104.800003	189.167007	104.804588	82.455040	110.862610
104.820000	207.333008	104.824585	98.266785	110.837280
104.840004	232.500000	104.844589	113.033005	110.811951
104.860001	243.167007	104.864578	124.817940	110.786682
104.880005	245.167007	104.884583	131.782104	110.761414

104.900002	255.833008	104.904579	132.697021	110.736206
104.919998	248.500000	104.924568	127.476753	110.710938
104.940002	233.000000	104.944572	117.249153	110.685791
104.959999	222.833008	104.964561	104.007538	110.660645
104.980003	207.167007	104.984566	89.869987	110.635559
105.000000	191.667007	105.004562	76.533066	110.610474
105.019997	171.000000	105.024551	64.935051	110.585510
105.040001	160.833008	105.044556	55.320358	110.560547
105.059998	162.167007	105.064552	47.516659	110.535645
105.080002	146.000000	105.084549	41.149944	110.510742
105.099998	143.333008	105.104546	35.842003	110.485962
105.119995	139.500000	105.124535	31.293255	110.461182
105.139999	136.833008	105.144539	27.294304	110.436523
105.159996	133.333008	105.164536	23.727636	110.411865
105.180000	136.667007	105.184532	20.526150	110.387329
105.199997	136.833008	105.204529	17.657581	110.362793
105.219994	130.000000	105.224518	15.106186	110.338379
105.239998	132.667007	105.244514	12.857888	110.314087
105.259995	119.500000	105.264511	10.900270	110.289795
105.279999	120.333000	105.284508	9.216306	110.265625
105.300003	121.167000	105.304512	7.784688	110.241516
105.320000	121.833000	105.324509	6.583215	110.217468
105.340004	113.500000	105.344505	5.586344	110.193542
105.360001	113.000000	105.364502	4.769127	110.169678
105.380005	110.667000	105.384499	4.107593	110.145935
105.400002	113.167000	105.404495	3.579486	110.122314
105.419998	115.000000	105.424492	3.164663	110.098816
105.440002	114.000000	105.444489	2.845173	110.075317
105.459999	101.000000	105.464485	2.605160	110.052002
105.480003	106.500000	105.484489	2.430585	110.028748
105.500000	113.333000	105.504478	2.309443	110.005615
105.519997	108.500000	105.524475	2.231355	109.982666
105.540001	110.500000	105.544472	2.188940	109.959717
105.559998	113.500000	105.564468	2.178657	109.936951
105.580002	109.833000	105.584473	2.202294	109.914307
105.599998	112.167000	105.604462	2.268086	109.891785
105.619995	108.833000	105.624458	2.391673	109.869385
105.639999	112.500000	105.644455	2.596523	109.847046

105.659996	115.500000	105.664452	2.914909	109.824951
105.680000	114.333000	105.684456	3.389646	109.802979
105.699997	111.500000	105.704445	4.075345	109.781128
105.719994	113.500000	105.724442	5.042328	109.759399
105.739998	114.167000	105.744446	6.376168	109.737793
105.759995	116.500000	105.764435	8.173738	109.716370
105.779999	116.333000	105.784439	10.543530	109.695068
105.800003	116.500000	105.804436	13.582439	109.673950
105.820000	123.667000	105.824432	17.361494	109.652954
105.840004	130.000000	105.844437	21.891191	109.632141
105.860001	139.333008	105.864426	27.073078	109.611511
105.880005	144.500000	105.884430	32.682938	109.591003
105.900002	153.000000	105.904419	38.323257	109.570679
105.919998	151.167007	105.924416	43.478424	109.550537
105.940002	163.167007	105.944420	47.572876	109.530518
105.959999	162.167007	105.964409	50.102524	109.510681
105.980003	167.833008	105.984413	50.775867	109.491089
106.000000	172.500000	106.004410	49.578175	109.471619
106.019997	167.333008	106.024399	46.772079	109.452393
106.040001	159.500000	106.044403	42.806030	109.433228
106.059998	151.333008	106.064392	38.216003	109.414368
106.080002	142.000000	106.084396	33.495182	109.395630
106.099998	134.167007	106.104393	29.037937	109.377075
106.119995	134.000000	106.124382	25.081331	109.358765
106.139999	127.833000	106.144386	21.710447	109.340576
106.159996	124.000000	106.164375	18.908806	109.322693
106.180000	130.333008	106.184380	16.590281	109.304932
106.199997	123.000000	106.204376	14.660405	109.287476
106.219994	118.167000	106.224365	13.034215	109.270142
106.239998	126.167000	106.244370	11.650923	109.252991
106.259995	122.833000	106.264366	10.480302	109.236084
106.279999	126.667000	106.284363	9.513717	109.219421
106.300003	119.667000	106.304367	8.762489	109.202942
106.320000	118.500000	106.324356	8.255545	109.186768
106.340004	117.167000	106.344360	8.035695	109.170715
106.360001	123.500000	106.364357	8.162402	109.154907
106.380005	114.000000	106.384354	8.710657	109.139343
106.400002	118.833000	106.404350	9.771274	109.124023

106.419998	123.500000	106.424339	11.446762	109.108948
106.440002	112.167000	106.444344	13.846663	109.094116
106.459999	124.333000	106.464340	17.063040	109.079468
106.480003	126.333000	106.484337	21.150646	109.065063
106.500000	126.500000	106.504333	26.083242	109.050903
106.519997	136.000000	106.524330	31.706146	109.036987
106.540001	148.000000	106.544327	37.690857	109.023315
106.559998	151.333008	106.564323	43.515987	109.010010
106.580002	149.000000	106.584320	48.504330	108.996826
106.599998	158.500000	106.604317	51.939857	108.983887
106.619995	156.167007	106.624313	53.254322	108.971252
106.639999	153.833008	106.644310	52.221397	108.958923
106.659996	153.667007	106.664307	49.060093	108.946777
106.680000	157.833008	106.684303	44.376278	108.934937
106.699997	143.167007	106.704300	38.959862	108.923340
106.719994	152.333008	106.724297	33.541725	108.912048
106.739998	133.833008	106.744293	28.625265	108.901001
106.759995	134.667007	106.764290	24.442909	108.890259
106.779999	120.333000	106.784294	21.009750	108.879700
106.800003	121.833000	106.804291	18.221750	108.869507
106.820000	125.000000	106.824287	15.930667	108.859558
106.840004	122.167000	106.844284	14.001291	108.849854
106.860001	111.000000	106.864281	12.332036	108.840454
106.880005	117.167000	106.884285	10.856869	108.831421
106.900002	120.333000	106.904274	9.541159	108.822632
106.919998	119.833000	106.924271	8.367170	108.814087
106.940002	119.667000	106.944267	7.331225	108.805847
106.959999	124.667000	106.964264	6.435096	108.797913
106.980003	120.167000	106.984268	5.682455	108.790283
107.000000	110.000000	107.004257	5.077853	108.782898
107.019997	111.000000	107.024254	4.622582	108.775879
107.040001	109.500000	107.044258	4.315754	108.769165
107.059998	110.667000	107.064247	4.151636	108.762695
107.080002	108.167000	107.084251	4.116631	108.756592
107.099998	108.167000	107.104240	4.187438	108.750732
107.119995	110.667000	107.124237	4.328710	108.745239
107.139999	115.000000	107.144241	4.493502	108.739990
107.159996	111.833000	107.164230	4.627727	108.735107

107.180000	110.167000	107.184235	4.680540	108.730591
107.199997	112.833000	107.204224	4.616588	108.726318
107.219994	114.000000	107.224220	4.427185	108.722351
107.239998	108.833000	107.244225	4.132642	108.718750
107.259995	112.000000	107.264214	3.774662	108.715454
107.279999	110.167000	107.284210	3.399720	108.712402
107.300003	107.333000	107.304207	3.045773	108.709778
107.320000	109.167000	107.324203	2.734248	108.707458
107.340004	105.667000	107.344208	2.471189	108.705444
107.360001	106.833000	107.364197	2.254004	108.703796
107.380005	101.667000	107.384201	2.077351	108.702454
107.400002	108.333000	107.404198	1.938601	108.701416
107.419998	102.667000	107.424187	1.838009	108.700745
107.440002	109.500000	107.444191	1.777648	108.700439
107.459999	103.167000	107.464180	1.759517	108.700378
107.480003	95.500000	107.484184	1.783251	108.700684
107.500000	105.000000	107.504181	1.844661	108.701355
107.519997	103.000000	107.524170	1.934477	108.702393
107.540001	113.667000	107.544174	2.037961	108.703735
107.559998	109.833000	107.564163	2.135211	108.705444
107.580002	110.167000	107.584167	2.204158	108.707458
107.599998	108.000000	107.604164	2.225116	108.709839
107.619995	109.833000	107.624153	2.186661	108.712585
107.639999	108.833000	107.644157	2.089565	108.715698
107.659996	109.000000	107.664146	1.947303	108.719116
107.680000	104.333000	107.684151	1.781094	108.722839
107.699997	105.333000	107.704147	1.613724	108.726990
107.719994	103.833000	107.724136	1.462934	108.731506
107.739998	109.000000	107.744141	1.338619	108.736328
107.759995	108.167000	107.764137	1.243813	108.741516
107.779999	105.000000	107.784134	1.176562	108.747009
107.800003	111.000000	107.804138	1.133036	108.752930
107.820000	102.333000	107.824127	1.109507	108.759155
107.840004	113.333000	107.844131	1.103608	108.765808
107.860001	109.167000	107.864128	1.115080	108.772705
107.880005	104.000000	107.884125	1.146053	108.780090
107.900002	110.000000	107.904121	1.201410	108.787842
107.919998	106.667000	107.924110	1.289234	108.795776

107.940002	110.000000	107.944115	1.421744	108.804260
107.959999	114.000000	107.964111	1.615875	108.813049
107.980003	114.000000	107.984108	1.894880	108.822144
108.000000	108.167000	108.004105	2.289416	108.831665
108.019997	106.333000	108.024094	2.838493	108.841553
108.040001	116.333000	108.044098	3.591378	108.851807
108.059998	114.000000	108.064095	4.604639	108.862366
108.080002	111.500000	108.084091	5.942385	108.873291
108.099998	109.333000	108.104088	7.669094	108.884644
108.119995	109.833000	108.124084	9.840246	108.896301
108.139999	125.667000	108.144081	12.486023	108.908447
108.159996	123.000000	108.164078	15.589689	108.920776
108.180000	135.000000	108.184074	19.061306	108.933594
108.199997	124.333000	108.204071	22.714598	108.946777
108.219994	139.667007	108.224068	26.258312	108.960266
108.239998	148.500000	108.244064	29.316525	108.974182
108.259995	147.667007	108.264061	31.491014	108.988464
108.279999	152.333008	108.284058	32.459442	109.003052
108.300003	142.000000	108.304062	32.079235	109.018066
108.320000	141.833008	108.324059	30.449940	109.033447
108.340004	143.000000	108.344055	27.891844	109.049255
108.360001	129.167007	108.364052	24.846739	109.065308
108.380005	122.500000	108.384048	21.747864	109.081787
108.400002	123.333000	108.404045	18.919550	109.098633
108.419998	128.333008	108.424042	16.540274	109.115845
108.440002	126.833000	108.444038	14.663141	109.133423
108.459999	124.500000	108.464035	13.265015	109.151367
108.480003	125.167000	108.484039	12.295753	109.169678
108.500000	125.167000	108.504028	11.714641	109.188354
108.519997	117.333000	108.524025	11.505132	109.207397
108.540001	117.500000	108.544022	11.681868	109.226868
108.559998	115.833000	108.564018	12.284520	109.246582
108.580002	123.667000	108.584023	13.368231	109.266785
108.599998	125.500000	108.604012	14.986073	109.287292
108.619995	129.333008	108.624008	17.174410	109.308228
108.639999	123.333000	108.644005	19.922894	109.329407
108.659996	137.500000	108.664001	23.148836	109.351013
108.680000	137.667007	108.684006	26.671991	109.372986

108.699997	134.667007	108.703995	30.194998	109.395325
108.719994	138.167007	108.723991	33.332962	109.418030
108.739998	143.000000	108.743988	35.657536	109.441040
108.759995	149.000000	108.763985	36.801979	109.464478
108.779999	145.333008	108.783989	36.572109	109.488281
108.800003	143.500000	108.803986	35.025520	109.512390
108.820000	141.167007	108.823982	32.470081	109.536865
108.840004	140.333008	108.843979	29.375607	109.561646
108.860001	136.833008	108.863976	26.239950	109.586914
108.880005	130.333008	108.883980	23.471292	109.612488
108.900002	123.000000	108.903969	21.337061	109.638306
108.919998	131.667007	108.923965	19.962074	109.664612
108.940002	132.500000	108.943970	19.381729	109.691223
108.959999	130.000000	108.963959	19.585615	109.718201
108.980003	124.667000	108.983963	20.549623	109.745483
109.000000	131.000000	109.003952	22.242226	109.773071
109.019997	133.333008	109.023949	24.616913	109.801147
109.040001	140.167007	109.043953	27.582664	109.829529
109.059998	144.167007	109.063942	30.970871	109.858154
109.080002	150.167007	109.083946	34.526394	109.887207
109.099998	159.000000	109.103935	37.887756	109.916565
109.119995	160.833008	109.123924	40.637756	109.946228
109.139999	161.333008	109.143929	42.362194	109.976318
109.159996	163.667007	109.163918	42.743034	110.006714
109.180000	168.667007	109.183922	41.657677	110.037354
109.199997	160.667007	109.203911	39.227116	110.068481
109.219994	156.167007	109.223907	35.788898	110.099854
109.239998	158.000000	109.243912	31.808336	110.131592
109.259995	146.833008	109.263901	27.755854	110.163513
109.279999	138.667007	109.283905	23.984381	110.195923
109.300003	139.667007	109.303902	20.701620	110.228638
109.320000	135.333008	109.323898	17.963959	110.261658
109.340004	127.167000	109.343903	15.729151	110.294983
109.360001	127.833000	109.363892	13.911178	110.328552
109.380005	128.833008	109.383896	12.412674	110.362549
109.400002	123.500000	109.403893	11.157980	110.396851
109.419998	122.833000	109.423882	10.094921	110.431396
109.440002	127.500000	109.443886	9.195766	110.466309

109.459999	123.667000	109.463875	8.456497	110.501465
109.480003	122.000000	109.483879	7.886861	110.536987
109.500000	120.333000	109.503876	7.510579	110.572754
109.519997	122.833000	109.523865	7.359322	110.608887
109.540001	117.833000	109.543869	7.471134	110.645264
109.559998	121.167000	109.563858	7.887667	110.682007
109.580002	118.333000	109.583862	8.650104	110.718994
109.599998	123.167000	109.603859	9.790720	110.756287
109.619995	127.333000	109.623848	11.323936	110.793823
109.639999	127.167000	109.643852	13.234655	110.831665
109.659996	128.167007	109.663841	15.454504	110.869812
109.680000	130.000000	109.683846	17.857994	110.908264
109.699997	129.500000	109.703842	20.244043	110.946899
109.719994	136.333008	109.723831	22.356617	110.985901
109.739998	140.000000	109.743835	23.921162	111.025146
109.759995	130.500000	109.763824	24.699564	111.064636
109.779999	141.333008	109.783829	24.566710	111.104431
109.800003	141.167007	109.803833	23.552156	111.144409
109.820000	134.667007	109.823822	21.843142	111.184692
109.840004	128.333008	109.843826	19.722969	111.225281
109.860001	130.167007	109.863815	17.496925	111.266113
109.880005	127.333000	109.883820	15.406006	111.307190
109.900002	119.667000	109.903816	13.601385	111.348450
109.919998	117.333000	109.923805	12.138426	111.390015
109.940002	127.500000	109.943810	11.006313	111.431763
109.959999	114.500000	109.963806	10.166363	111.473877
109.980003	121.000000	109.983803	9.574849	111.516113
110.000000	116.167000	110.003799	9.202485	111.558594
110.019997	120.500000	110.023788	9.041695	111.601318
110.040001	124.333000	110.043793	9.108223	111.644287
110.059998	127.833000	110.063789	9.440091	111.687439
110.080002	122.500000	110.083786	10.094989	111.730835
110.099998	132.167007	110.103783	11.147178	111.774475
110.119995	138.000000	110.123772	12.682426	111.818237
110.139999	128.667007	110.143776	14.794725	111.862305
110.159996	133.500000	110.163773	17.568895	111.906555
110.180000	141.833008	110.183769	21.071985	111.950928
110.199997	135.667007	110.203766	25.329237	111.995605

110.219994	156.167007	110.223755	30.299730	112.040405
110.239998	158.333008	110.243759	35.867062	112.085388
110.259995	165.000000	110.263756	41.814407	112.130615
110.279999	187.667007	110.283752	47.866814	112.175964
110.300003	173.000000	110.303757	53.739563	112.221558
110.320000	177.333008	110.323746	59.211529	112.267273
110.340004	201.000000	110.343750	64.227684	112.313171
110.360001	196.667007	110.363747	68.903267	112.359253
110.380005	197.500000	110.383743	73.506683	112.405518
110.400002	210.000000	110.403740	78.347717	112.451904
110.419998	203.167007	110.423729	83.662506	112.498413
110.440002	218.333008	110.443733	89.548981	112.545105
110.459999	224.833008	110.463730	95.927689	112.591980
110.480003	232.000000	110.483727	102.574196	112.638977
110.500000	225.500000	110.503723	109.108223	112.686096
110.519997	236.500000	110.523712	114.973228	112.733276
110.540001	227.500000	110.543716	119.447792	112.780701
110.559998	236.000000	110.563713	121.713356	112.828247
110.580002	230.167007	110.583710	121.073380	112.875854
110.599998	224.833008	110.603706	117.195976	112.923645
110.619995	216.667007	110.623695	110.301811	112.971436
110.639999	216.833008	110.643700	101.147148	113.019409
110.659996	203.833008	110.663696	90.851013	113.067505
110.680000	200.667007	110.683693	80.551933	113.115662
110.699997	195.667007	110.703690	71.147682	113.163879
110.719994	193.000000	110.723686	63.152431	113.212280
110.739998	177.000000	110.743683	56.702694	113.260742
110.759995	167.500000	110.763680	51.654388	113.309265
110.779999	173.333008	110.783676	47.702946	113.357849
110.800003	166.000000	110.803673	44.484627	113.406494
110.820000	148.833008	110.823669	41.647583	113.455200
110.840004	154.833008	110.843666	38.900085	113.504028
110.860001	151.833008	110.863663	36.043804	113.552917
110.880005	153.000000	110.883659	32.993027	113.601807
110.900002	150.333008	110.903656	29.773523	113.650757
110.919998	148.667007	110.923653	26.496510	113.699768
110.940002	136.667007	110.943649	23.314369	113.748840
110.959999	137.167007	110.963646	20.373791	113.797852

110.980003	138.833008	110.983643	17.783037	113.847046
111.000000	138.333008	111.003639	15.601039	113.896118
111.019997	130.500000	111.023636	13.844729	113.945251
111.040001	129.833008	111.043633	12.505398	113.994385
111.059998	118.333000	111.063629	11.565040	114.043640
111.080002	127.167000	111.083626	11.007584	114.092773
111.099998	125.500000	111.103622	10.823462	114.141968
111.119995	129.167007	111.123619	11.008091	114.191162
111.139999	128.500000	111.143616	11.555387	114.240295
111.159996	129.833008	111.163612	12.447542	114.289429
111.180000	127.000000	111.183609	13.642456	114.338623
111.199997	137.167007	111.203606	15.061104	114.387695
111.219994	126.333000	111.223602	16.578999	114.436768
111.239998	129.167007	111.243599	18.027176	114.485779
111.259995	136.833008	111.263596	19.208733	114.534790
111.279999	127.333000	111.283592	19.933033	114.583740
111.300003	133.333008	111.303596	20.061962	114.632690
111.320000	129.333008	111.323593	19.552732	114.681519
111.340004	136.833008	111.343590	18.477411	114.730225
111.360001	129.333008	111.363586	17.005363	114.778931
111.380005	134.333008	111.383583	15.354618	114.827637
111.400002	131.500000	111.403580	13.732204	114.876221
111.419998	124.667000	111.423576	12.288138	114.924683
111.440002	134.667007	111.443573	11.096280	114.973083
111.459999	127.667000	111.463570	10.160562	115.021362
111.480003	117.000000	111.483566	9.435747	115.069580
111.500000	116.167000	111.503563	8.851757	115.117676
111.519997	119.833000	111.523560	8.334876	115.165771
111.540001	119.833000	111.543556	7.823654	115.213623
111.559998	119.333000	111.563553	7.279021	115.261353
111.580002	123.000000	111.583549	6.688116	115.309021
111.599998	114.833000	111.603546	6.061526	115.356506
111.619995	117.667000	111.623543	5.425047	115.403870
111.639999	120.667000	111.643539	4.809074	115.451111
111.659996	120.167000	111.663536	4.239452	115.498230
111.680000	112.167000	111.683533	3.732414	115.545166
111.699997	115.833000	111.703529	3.293941	115.591980
111.719994	108.167000	111.723526	2.922085	115.638550

111.739998	118.167000	111.743523	2.610291	115.685059
111.759995	123.833000	111.763519	2.350313	115.731323
111.779999	112.000000	111.783516	2.134134	115.777466
111.800003	118.500000	111.803520	1.954846	115.823425
111.820000	116.333000	111.823517	1.807164	115.869202
111.840004	110.833000	111.843513	1.686911	115.914673
111.860001	114.500000	111.863510	1.591062	115.960083
111.880005	118.167000	111.883507	1.517444	116.005188
111.900002	120.667000	111.903503	1.464598	116.050171
111.919998	112.000000	111.923500	1.431746	116.094849
111.940002	117.000000	111.943497	1.418891	116.139404
111.959999	109.333000	111.963493	1.427036	116.183716
111.980003	105.667000	111.983490	1.458526	116.227783
112.000000	109.667000	112.003487	1.517555	116.271606
112.019997	122.833000	112.023483	1.610796	116.315186
112.040001	121.333000	112.043480	1.748222	116.358521
112.059998	120.667000	112.063477	1.944072	116.401611
112.080002	115.167000	112.083473	2.217983	116.444458
112.099998	111.167000	112.103470	2.596211	116.487061
112.119995	119.667000	112.123466	3.112719	116.529419
112.139999	115.167000	112.143463	3.810434	116.571411
112.159996	115.333000	112.163460	4.741331	116.613159
112.180000	118.500000	112.183456	5.966041	116.654663
112.199997	118.833000	112.203453	7.551525	116.695862
112.219994	127.667000	112.223450	9.566298	116.736755
112.239998	128.167007	112.243446	12.072359	116.777344
112.259995	133.167007	112.263443	15.113278	116.817688
112.279999	131.167007	112.283447	18.700329	116.857727
112.300003	134.167007	112.303444	22.789888	116.897400
112.320000	142.333008	112.323441	27.277632	116.936768
112.340004	147.333008	112.343437	31.988232	116.975830
112.360001	157.333008	112.363426	36.685745	117.014587
112.380005	159.667007	112.383430	41.111622	117.052979
112.400002	162.000000	112.403419	45.007584	117.091003
112.419998	162.833008	112.423416	48.177681	117.128784
112.440002	176.833008	112.443413	50.487240	117.166138
112.459999	175.500000	112.463409	51.858372	117.203186
112.480003	176.333008	112.483414	52.239834	117.239868

112.500000	179.000000	112.503403	51.591221	117.276184
112.519997	175.667007	112.523399	49.900272	117.312134
112.540001	181.333008	112.543396	47.228252	117.347717
112.559998	164.667007	112.563393	43.748764	117.382935
112.580002	158.833008	112.583397	39.744396	117.417786
112.599998	153.667007	112.603386	35.563332	117.452148
112.619995	162.833008	112.623383	31.525606	117.486267
112.639999	139.833008	112.643379	27.872297	117.519958
112.659996	141.667007	112.663376	24.727264	117.553223
112.680000	138.167007	112.683380	22.106060	117.586121
112.699997	129.000000	112.703369	19.952114	117.618530
112.719994	130.833008	112.723366	18.164932	117.650635
112.739998	129.667007	112.743362	16.639969	117.682251
112.759995	126.167000	112.763359	15.284863	117.713562
112.779999	123.833000	112.783363	14.031547	117.744385
112.800003	119.333000	112.803360	12.843086	117.774719
112.820000	127.833000	112.823357	11.710564	117.804688
112.840004	123.167000	112.843353	10.650020	117.834229
112.860001	131.500000	112.863350	9.693618	117.863342
112.880005	132.333008	112.883354	8.879836	117.891968
112.900002	126.167000	112.903343	8.246716	117.920166
112.919998	118.500000	112.923340	7.824067	117.947937
112.940002	121.667000	112.943336	7.634219	117.975220
112.959999	120.500000	112.963333	7.690193	118.002075
112.980003	109.833000	112.983330	7.995192	118.028442
113.000000	114.833000	113.003326	8.540217	118.054382
113.019997	118.333000	113.023323	9.299665	118.079834
113.040001	120.333000	113.043320	10.225801	118.104797
113.059998	124.167000	113.063316	11.244145	118.129272
113.080002	123.667000	113.083313	12.253099	118.153320
113.099998	128.000000	113.103310	13.131083	118.176819
113.119995	118.333000	113.123306	13.753532	118.199951
113.139999	125.000000	113.143303	14.018169	118.222412
113.159996	135.500000	113.163300	13.871910	118.244507
113.180000	127.000000	113.183296	13.328759	118.266174
113.199997	120.000000	113.203293	12.469424	118.287231
113.219994	124.500000	113.223289	11.420869	118.307861
113.239998	117.333000	113.243286	10.323717	118.327881

113.259995	121.333000	113.263283	9.301221	118.347534
113.279999	116.500000	113.283279	8.440804	118.366577
113.300003	114.167000	113.303284	7.791403	118.385132
113.320000	118.833000	113.323280	7.373979	118.403198
113.340004	115.667000	113.343277	7.194575	118.420715
113.360001	123.167000	113.363274	7.259074	118.437744
113.380005	115.500000	113.383270	7.582412	118.454224
113.400002	117.167000	113.403267	8.193424	118.470215
113.419998	123.333000	113.423264	9.135441	118.485657
113.440002	119.000000	113.443260	10.463506	118.500610
113.459999	126.333000	113.463257	12.238592	118.514893
113.480003	127.833000	113.483253	14.518966	118.528809
113.500000	130.167007	113.503250	17.348818	118.542114
113.519997	129.500000	113.523247	20.744787	118.554932
113.540001	135.333008	113.543243	24.682095	118.567139
113.559998	141.167007	113.563240	29.083374	118.578918
113.580002	148.167007	113.583237	33.814034	118.590088
113.599998	150.667007	113.603233	38.687321	118.600647
113.619995	150.000000	113.623230	43.478638	118.610718
113.639999	160.333008	113.643227	47.944160	118.620300
113.659996	169.667007	113.663223	51.835831	118.629272
113.680000	170.167007	113.683220	54.908871	118.637756
113.699997	179.667007	113.703217	56.927647	118.645630
113.719994	176.833008	113.723213	57.684460	118.652954
113.739998	177.667007	113.743210	57.042210	118.659790
113.759995	181.500000	113.763206	54.992168	118.666016
113.779999	169.833008	113.783203	51.698078	118.671753
113.800003	165.833008	113.803207	47.491173	118.676880
113.820000	159.500000	113.823196	42.818108	118.681519
113.840004	168.667007	113.843193	38.126934	118.685486
113.860001	146.667007	113.863190	33.793171	118.689026
113.880005	152.000000	113.883186	30.059330	118.691895
113.900002	141.333008	113.903183	27.033611	118.694336
113.919998	138.833008	113.923180	24.721968	118.696167
113.940002	139.833008	113.943176	23.072857	118.697388
113.959999	136.000000	113.963173	22.016117	118.698120
113.980003	140.333008	113.983170	21.487122	118.698303
114.000000	133.500000	114.003166	21.434862	118.697876

114.019997	136.000000	114.023163	21.817244	118.696899
114.040001	132.667007	114.043159	22.588232	118.695435
114.059998	144.500000	114.063156	23.681358	118.693420
114.080002	148.167007	114.083153	24.994295	118.690796
114.099998	157.333008	114.103149	26.379827	118.687683
114.119995	153.167007	114.123146	27.648930	118.683960
114.139999	150.500000	114.143143	28.590540	118.679749
114.159996	158.333008	114.163139	29.008373	118.674927
114.180000	156.333008	114.183136	28.767519	118.669678
114.199997	151.500000	114.203133	27.835594	118.663757
114.219994	146.167007	114.223129	26.300617	118.657349
114.239998	147.833008	114.243126	24.355024	118.650452
114.259995	146.333008	114.263123	22.250326	118.642944
114.279999	142.000000	114.283119	20.241243	118.634888
114.300003	135.167007	114.303123	18.540806	118.626343
114.320000	137.833008	114.323120	17.303841	118.617188
114.340004	138.500000	114.343117	16.629440	118.607605
114.360001	138.167007	114.363113	16.586172	118.597473
114.380005	134.500000	114.383110	17.237888	118.586792
114.400002	137.333008	114.403107	18.665365	118.575562
114.419998	141.833008	114.423103	20.979006	118.563904
114.440002	145.667007	114.443100	24.321123	118.551697
114.459999	141.167007	114.463097	28.857920	118.538940
114.480003	149.167007	114.483093	34.760864	118.525635
114.500000	157.500000	114.503090	42.176437	118.511902
114.519997	169.833008	114.523079	51.179550	118.497620
114.540001	171.667007	114.543083	61.737629	118.482910
114.559998	180.833008	114.563080	73.611473	118.467590
114.580002	204.833008	114.583076	86.335693	118.451782
114.599998	225.333008	114.603073	99.168533	118.435547
114.619995	228.000000	114.623062	111.112892	118.418884
114.639999	245.167007	114.643066	121.038872	118.401611
114.659996	254.000000	114.663063	127.829193	118.383911
114.680000	269.332977	114.683060	130.667709	118.365723
114.699997	257.332977	114.703056	129.248566	118.347168
114.719994	264.832977	114.723045	123.903526	118.328003
114.739998	258.500000	114.743050	115.529762	118.308472
114.759995	241.833008	114.763046	105.385048	118.288513

114.779999	226.167007	114.783043	94.743362	118.268066
114.800003	204.167007	114.803047	84.622749	118.247070
114.820000	204.667007	114.823036	75.646217	118.225769
114.840004	197.833008	114.843040	67.995789	118.203979
114.860001	186.000000	114.863037	61.563148	118.181763
114.880005	168.667007	114.883034	56.061813	118.159180
114.900002	175.333008	114.903030	51.179737	118.136108
114.919998	164.167007	114.923019	46.670105	118.112671
114.940002	170.500000	114.943024	42.381889	118.088806
114.959999	161.000000	114.963020	38.270615	118.064514
114.980003	154.667007	114.983017	34.349949	118.039917
115.000000	152.667007	115.003014	30.662380	118.014832
115.019997	145.667007	115.023003	27.249846	117.989441
115.040001	150.500000	115.043007	24.132586	117.963623
115.059998	151.000000	115.063004	21.319048	117.937500
115.080002	149.000000	115.083000	18.797564	117.910889
115.099998	138.500000	115.102997	16.549017	117.884033
115.119995	140.000000	115.122986	14.551647	117.856812
115.139999	132.000000	115.142990	12.780870	117.829224
115.159996	128.500000	115.162987	11.218041	117.801331
115.180000	138.667007	115.182983	9.843682	117.773193
115.199997	126.000000	115.202972	8.641314	117.744629
115.219994	124.833000	115.222961	7.595030	117.715820
115.239998	124.333000	115.242966	6.690159	117.686646
115.259995	118.000000	115.262955	5.915265	117.657227
115.279999	123.833000	115.282959	5.257659	117.627441
115.300003	133.500000	115.302963	4.708055	117.597473
115.320000	120.500000	115.322952	4.258768	117.567261
115.340004	121.500000	115.342957	3.903526	117.536621
115.360001	125.333000	115.362946	3.640223	117.505859
115.380005	120.333000	115.382950	3.469355	117.474792
115.400002	122.833000	115.402946	3.396272	117.443604
115.419998	119.333000	115.422935	3.430848	117.412109
115.440002	130.667007	115.442940	3.588749	117.380371
115.459999	124.500000	115.462929	3.891384	117.348511
115.480003	117.333000	115.482933	4.367282	117.316406
115.500000	123.833000	115.502930	5.050218	117.284058
115.519997	115.833000	115.522919	5.979333	117.251587

115.540001	121.500000	115.542923	7.198016	117.218933
115.559998	128.500000	115.562912	8.745344	117.186157
115.580002	122.000000	115.582916	10.657064	117.153198
115.599998	125.000000	115.602913	12.948133	117.120117
115.619995	129.333008	115.622902	15.607460	117.086914
115.639999	144.500000	115.642906	18.587866	117.053467
115.659996	141.500000	115.662895	21.784435	117.020081
115.680000	141.833008	115.682899	25.047682	116.986450
115.699997	148.167007	115.702896	28.174019	116.952881
115.719994	159.833008	115.722885	30.943413	116.919189
115.739998	150.167007	115.742889	33.160126	116.885376
115.759995	162.833008	115.762878	34.695293	116.851562
115.779999	158.333008	115.782883	35.543102	116.817627
115.800003	148.667007	115.802879	35.828819	116.783691
115.820000	155.333008	115.822876	35.791729	116.749695
115.840004	155.833008	115.842880	35.722698	116.715759
115.860001	145.833008	115.862869	35.885658	116.681824
115.880005	152.167007	115.882874	36.446281	116.647827
115.900002	153.167007	115.902863	37.426868	116.613892
115.919998	154.000000	115.922859	38.702858	116.580017
115.940002	158.167007	115.942863	40.028618	116.546082
115.959999	158.500000	115.962852	41.091743	116.512329
115.980003	162.667007	115.982857	41.588799	116.478577
116.000000	161.167007	116.002846	41.292892	116.444946
116.019997	162.333008	116.022842	40.112846	116.411316
116.040001	158.333008	116.042847	38.112751	116.377930
116.059998	150.167007	116.062836	35.494850	116.344543
116.080002	150.667007	116.082840	32.531418	116.311279
116.099998	154.500000	116.102829	29.507887	116.278198
116.119995	139.667007	116.122826	26.648064	116.245300
116.139999	136.500000	116.142830	24.096973	116.212524
116.159996	139.333008	116.162819	21.922478	116.179871
116.180000	132.667007	116.182823	20.130310	116.147522
116.199997	140.167007	116.202812	18.704533	116.115295
116.219994	133.833008	116.222809	17.619093	116.083252
116.239998	133.000000	116.242813	16.855097	116.051453
116.259995	133.500000	116.262802	16.399025	116.020020
116.279999	135.667007	116.282806	16.234636	115.988647

116.300003	124.667000	116.302803	16.336866	115.957642
116.320000	124.667000	116.322800	16.661921	115.927002
116.340004	124.833000	116.342796	17.142208	115.896484
116.360001	131.500000	116.362793	17.684412	115.866333
116.380005	132.500000	116.382797	18.173954	115.836426
116.400002	127.833000	116.402786	18.486135	115.807007
116.419998	130.667007	116.422783	18.507208	115.777771
116.440002	137.000000	116.442780	18.156340	115.748962
116.459999	135.500000	116.462776	17.406984	115.720459
116.480003	131.167007	116.482780	16.295731	115.692383
116.500000	126.333000	116.502769	14.917198	115.664673
116.519997	120.833000	116.522758	13.397313	115.637451
116.540001	131.667007	116.542755	11.865829	115.610474
116.559998	123.333000	116.562752	10.431294	115.584106
116.580002	125.000000	116.582756	9.163485	115.558044
116.599998	118.500000	116.602745	8.094667	115.532471
116.619995	117.833000	116.622742	7.223197	115.507446
116.639999	117.167000	116.642738	6.530326	115.482788
116.659996	120.500000	116.662735	5.989879	115.458679
116.680000	126.167000	116.682739	5.577351	115.435059
116.699997	120.333000	116.702728	5.275739	115.411926
116.719994	114.333000	116.722725	5.076148	115.389343
116.739998	124.000000	116.742722	4.979147	115.367310
116.759995	119.667000	116.762718	4.992834	115.345825
116.779999	119.667000	116.782715	5.131070	115.324890
116.800003	121.500000	116.802719	5.411295	115.304565
116.820000	116.500000	116.822716	5.851297	115.284790
116.840004	119.500000	116.842712	6.466502	115.265564
116.860001	123.167000	116.862709	7.265535	115.247070
116.880005	122.833000	116.882706	8.245555	115.229126
116.900002	121.333000	116.902702	9.387247	115.211792
116.919998	121.000000	116.922699	10.650541	115.195190
116.940002	123.333000	116.942696	11.972669	115.179199
116.959999	131.667007	116.962692	13.270664	115.163818
116.980003	134.833008	116.982689	14.450098	115.149231
117.000000	130.833008	117.002686	15.420337	115.135254
117.019997	139.333008	117.022682	16.114002	115.122009
117.040001	134.167007	117.042679	16.505186	115.109497

117.059998	127.000000	117.062675	16.619362	115.097656
117.080002	127.667000	117.082672	16.529953	115.086670
117.099998	146.500000	117.102669	16.341228	115.076294
117.119995	140.333008	117.122665	16.163330	115.066650
117.139999	131.833008	117.142662	16.087730	115.057861
117.159996	126.833000	117.162659	16.169889	115.049805
117.180000	138.500000	117.182655	16.421413	115.042603
117.199997	137.500000	117.202652	16.810091	115.036133
117.219994	136.833008	117.222641	17.264994	115.030457
117.239998	136.833008	117.242645	17.687147	115.025513
117.259995	134.000000	117.262642	17.962473	115.021484
117.279999	133.167007	117.282639	17.983437	115.018311
117.300003	141.000000	117.302643	17.670662	115.015869
117.320000	145.333008	117.322632	16.993624	115.014343
117.340004	128.000000	117.342636	15.977752	115.013672
117.360001	128.167007	117.362633	14.702461	115.013794
117.380005	132.833008	117.382629	13.278452	115.014893
117.400002	129.833008	117.402626	11.822932	115.016724
117.419998	139.167007	117.422615	10.435763	115.019470
117.440002	128.333008	117.442619	9.182332	115.023071
117.459999	129.667007	117.462616	8.095610	115.027588
117.480003	126.833000	117.482613	7.177031	115.033081
117.500000	119.833000	117.502609	6.409294	115.039307
117.519997	129.667007	117.522598	5.766418	115.046509
117.540001	123.000000	117.542603	5.220557	115.054688
117.559998	123.167000	117.562592	4.749218	115.063721
117.580002	122.000000	117.582596	4.334272	115.073730
117.599998	117.667000	117.602592	3.965044	115.084473
117.619995	116.333000	117.622581	3.635826	115.096252
117.639999	116.833000	117.642586	3.345111	115.108948
117.659996	122.333000	117.662575	3.095723	115.122498
117.680000	121.667000	117.682579	2.892461	115.137085
117.699997	118.000000	117.702576	2.743112	115.152588
117.719994	112.833000	117.722565	2.657244	115.168945
117.739998	113.667000	117.742569	2.646473	115.186340
117.759995	118.167000	117.762558	2.724464	115.204468
117.779999	116.167000	117.782555	2.906724	115.223633
117.800003	117.000000	117.802559	3.210215	115.243713

117.820000	122.167000	117.822548	3.651785	115.264709
117.840004	120.167000	117.842552	4.248201	115.286621
117.860001	122.500000	117.862541	5.010934	115.309326
117.880005	118.833000	117.882545	5.946306	115.333130
117.900002	116.000000	117.902534	7.046304	115.357666
117.919998	123.667000	117.922531	8.288664	115.383240
117.940002	116.167000	117.942535	9.628451	115.409668
117.959999	127.167000	117.962524	10.994490	115.436890
117.980003	131.833008	117.982529	12.295611	115.465088
118.000000	128.500000	118.002518	13.420129	115.494141
118.019997	136.667007	118.022514	14.257182	115.524048
118.040001	130.333008	118.042519	14.711545	115.554810
118.059998	131.167007	118.062508	14.726849	115.586426
118.080002	129.833008	118.082512	14.301748	115.618896
118.099998	128.833008	118.102501	13.494035	115.652100
118.119995	131.833008	118.122498	12.407443	115.686218
118.139999	130.500000	118.142502	11.170171	115.721069
118.159996	128.167007	118.162491	9.908803	115.756714
118.180000	124.167000	118.182495	8.720977	115.793152
118.199997	125.500000	118.202484	7.670933	115.830261
118.219994	126.500000	118.222481	6.782770	115.868164
118.239998	125.833000	118.242477	6.053274	115.906860
118.259995	121.500000	118.262474	5.460999	115.946106
118.279999	114.000000	118.282478	4.977080	115.986084
118.300003	118.500000	118.302475	4.573622	116.026855
118.320000	123.833000	118.322472	4.227069	116.068176
118.340004	118.667000	118.342468	3.920748	116.110107
118.360001	112.500000	118.362465	3.644704	116.152588
118.380005	119.833000	118.382469	3.394755	116.195801
118.400002	120.667000	118.402458	3.171827	116.239502
118.419998	123.500000	118.422455	2.980042	116.283630
118.440002	114.667000	118.442451	2.826866	116.328369
118.459999	112.667000	118.462448	2.722332	116.373535
118.480003	122.000000	118.482452	2.678843	116.419189
118.500000	121.833000	118.502441	2.711331	116.465210
118.519997	125.333000	118.522438	2.837324	116.511597
118.540001	114.833000	118.542435	3.077020	116.558350
118.559998	118.167000	118.562431	3.453107	116.605469

118.580002	118.500000	118.582428	3.990123	116.652893
118.599998	116.833000	118.602425	4.713129	116.700562
118.619995	119.833000	118.622421	5.645563	116.748413
118.639999	117.833000	118.642418	6.805576	116.796387
118.659996	121.667000	118.662415	8.201319	116.844482
118.680000	127.000000	118.682411	9.824753	116.892822
118.699997	124.833000	118.702408	11.644703	116.941162
118.719994	125.333000	118.722404	13.600343	116.989441
118.739998	129.333008	118.742401	15.597017	117.037720
118.759995	129.667007	118.762398	17.507231	117.085938
118.779999	138.167007	118.782394	19.179514	117.134033
118.800003	143.833008	118.802399	20.457163	117.181885
118.820000	138.833008	118.822388	21.202765	117.229614
118.840004	130.000000	118.842392	21.331465	117.277100
118.860001	138.333008	118.862389	20.830450	117.324097
118.880005	138.833008	118.882385	19.769415	117.370789
118.900002	133.167007	118.902382	18.287783	117.416992
118.919998	145.500000	118.922371	16.566082	117.462769
118.940002	123.833000	118.942375	14.785372	117.507996
118.959999	127.333000	118.962372	13.101340	117.552490
118.980003	118.167000	118.982361	11.618716	117.596375
119.000000	128.833008	119.002357	10.388973	117.639465
119.019997	121.667000	119.022346	9.422045	117.681763
119.040001	122.333000	119.042351	8.696825	117.723267
119.059998	127.000000	119.062347	8.179540	117.763794
119.080002	121.833000	119.082344	7.831487	117.803223
119.099998	124.333000	119.102341	7.616895	117.841553
119.119995	120.000000	119.122330	7.504628	117.878723
119.139999	118.833000	119.142334	7.467108	117.914673
119.159996	118.000000	119.162323	7.478198	117.949280
119.180000	125.167000	119.182327	7.510542	117.982483
119.199997	123.667000	119.202324	7.534310	118.014160
119.219994	125.167000	119.222313	7.517998	118.044312
119.239998	128.833008	119.242317	7.431198	118.072815
119.259995	127.167000	119.262306	7.249740	118.099487
119.279999	123.000000	119.282310	6.960374	118.124268
119.300003	119.500000	119.302315	6.565493	118.147278
119.320000	121.667000	119.322304	6.083751	118.168091

119.340004	127.167000	119.342308	5.545330	118.186890
119.360001	122.667000	119.362297	4.988183	118.203369
119.380005	123.000000	119.382301	4.447462	118.217529
119.400002	122.500000	119.402290	3.952767	118.229248
119.419998	120.833000	119.422287	3.522015	118.238403
119.440002	117.333000	119.442291	3.163613	118.244873
119.459999	123.000000	119.462280	2.878475	118.248657
119.480003	120.667000	119.482285	2.662350	118.249512
119.500000	119.167000	119.502274	2.510751	118.247314
119.519997	120.333000	119.522270	2.420277	118.242065
119.540001	127.333000	119.542274	2.391191	118.233398
119.559998	127.333000	119.562263	2.428001	118.221558
119.580002	112.000000	119.582268	2.539971	118.206116
119.599998	126.667000	119.602257	2.740768	118.187134
119.619995	127.167000	119.622253	3.048698	118.164307
119.639999	119.167000	119.642250	3.485462	118.137573
119.659996	118.000000	119.662247	4.075362	118.106812
119.680000	123.333000	119.682251	4.843892	118.071899
119.699997	122.333000	119.702240	5.813586	118.032715
119.719994	126.833000	119.722237	7.003748	117.989014
119.739998	134.000000	119.742233	8.422388	117.940613
119.759995	134.000000	119.762230	10.061980	117.887451
119.779999	131.000000	119.782234	11.893328	117.829346
119.800003	129.833008	119.802231	13.856638	117.766235
119.820000	126.833000	119.822227	15.861775	117.697876
119.840004	132.667007	119.842224	17.786377	117.623962
119.860001	145.333008	119.862221	19.484409	117.544556
119.880005	135.667007	119.882217	20.802750	117.459351
119.900002	143.000000	119.902214	21.605400	117.368286
119.919998	142.167007	119.922211	21.800953	117.270996
119.940002	147.333008	119.942207	21.365593	117.167419
119.959999	142.167007	119.962204	20.353161	117.057312
119.980003	139.167007	119.982201	18.887117	116.940552
120.000000	138.667007	120.002197	17.135361	116.816956

_reflns_number_total 288

_reflns_limit_h_min 0

_reflns_limit_h_max 10

```
_reflns_limit_k_min       -8
_reflns_limit_k_max       0
_reflns_limit_l_min       0
_reflns_limit_l_max       7
_reflns_d_resolution_high 8.153
_reflns_d_resolution_low  .890

loop_
      _refln_index_h
      _refln_index_k
      _refln_index_l
      _refln_F_squared_meas
      _refln_d_spacing
      _refln_intensity_meas
   1   0   0      124.970      8.15338    74754.930
   1   0   1       59.638      5.25963    29224.963
   2  -1   0       88.449      4.70736    17243.973
   2   0   0      313.506      4.07669    45334.098
   2  -1   1      174.032      3.88562    45520.652
   2   0   1       97.578      3.50766    20572.176
   0   0   2     6013.157      3.44167   202950.484
   1   0   2      304.329      3.17076    51744.184
   3  -2   0      584.192      3.08169    46717.996
   3  -1   0      582.578      3.08169    46588.895
   3  -2   1     2243.224      2.81267   294442.938
   3  -1   1     2240.557      2.81267   294092.812
   2  -1   2     2412.882      2.77830   308337.750
   3   0   0     5974.161      2.71779   363783.875
   2   0   2     1261.457      2.62981   142925.703
   3   0   1      302.399      2.52788    31400.221
   4  -2   0        0.177      2.35368        7.837
   3  -2   2      218.359      2.29584    18289.391
   3  -1   2      218.286      2.29584    18283.254
   4  -3   0     1823.694      2.26134    73813.406
   4  -1   0     1837.975      2.26134    74391.422
   4  -2   1      189.644      2.22708    14831.069
   1   0   3      114.554      2.20866     8791.773
   4  -3   1      317.420      2.14838    22877.516
```

4	-1	1	317.333	2.14838	22871.219
3	0	2	208.742	2.13294	14799.594
2	-1	3	585.897	2.06249	38469.527
4	0	0	465.453	2.03835	14873.264
2	0	3	553.747	1.99951	33857.105
4	0	1	942.308	1.95445	54661.512
4	-2	2	3681.127	1.94281	210603.750
4	-3	2	907.601	1.88990	48704.070
4	-1	2	905.439	1.88990	48588.039
5	-3	0	640.499	1.87052	16778.240
5	-2	0	639.782	1.87051	16759.453
3	-2	3	2293.396	1.84037	115690.102
3	-1	3	2292.946	1.84037	115667.367
5	-3	1	1252.644	1.80505	60394.867
5	-2	1	1251.749	1.80505	60351.691
5	-4	0	1971.797	1.77921	45957.703
5	-1	0	1972.753	1.77921	45979.938
4	0	2	2046.402	1.75383	92236.070
3	0	3	2046.089	1.75321	92145.344
5	-4	1	1347.172	1.72260	58214.879
5	-1	1	1347.481	1.72260	58228.246
0	0	4	16146.854	1.72083	116011.883
1	0	4	257.172	1.68374	10533.504
5	-3	2	589.901	1.64347	22826.480
5	-2	2	589.743	1.64347	22820.367
4	-2	3	1189.595	1.64296	45998.266
5	0	0	260.840	1.63068	4954.887
2	-1	4	751.169	1.61623	27948.143
4	-3	3	432.603	1.61059	15964.054
4	-1	3	432.447	1.61059	15958.297
5	0	1	570.715	1.58676	20337.307
2	0	4	642.118	1.58538	22835.129
5	-4	2	223.624	1.58051	7895.473
5	-1	2	223.614	1.58051	7895.114
6	-3	0	487.658	1.56912	8464.289
6	-4	0	1246.388	1.54085	20733.518
6	-2	0	1245.130	1.54084	20712.578
6	-3	1	907.745	1.52987	29701.359

4	0	3	375.292	1.52386	12167.445
6	-4	1	984.454	1.50363	30941.146
6	-2	1	985.057	1.50363	30960.084
3	-2	4	980.487	1.50246	30760.604
3	-1	4	980.432	1.50246	30758.871
5	0	2	2378.113	1.47364	71339.727
6	-5	0	747.375	1.46439	11048.812
6	-1	0	746.784	1.46439	11040.065
3	0	4	3705.530	1.45390	107767.039
5	-2	3	1648.640	1.44979	47637.078
5	-3	3	1648.640	1.44979	47637.098
6	-5	1	1260.856	1.43234	35438.523
6	-1	1	1262.370	1.43234	35481.055
6	-3	2	1755.463	1.42773	48980.527
6	-4	2	476.259	1.40634	12842.099
6	-2	2	475.675	1.40634	12826.348
5	-4	3	463.994	1.40602	12504.942
5	-1	3	463.823	1.40601	12500.333
4	-2	4	98.269	1.38915	2577.699
4	-3	4	53.546	1.36942	1360.633
4	-1	4	53.546	1.36942	1360.632
6	0	0	196.956	1.35890	2460.271
1	0	5	111.434	1.35745	2777.461
6	-5	2	347.384	1.34749	8520.267
6	-1	2	349.095	1.34749	8562.227
7	-4	0	155.968	1.34041	1890.965
7	-3	0	156.046	1.34041	1891.912
6	0	1	59.748	1.33317	1431.954
5	0	3	67.929	1.32918	1617.586
2	-1	5	528.896	1.32132	12435.902
7	-4	1	802.345	1.31569	18695.305
7	-3	1	802.946	1.31569	18709.297
4	0	4	1592.091	1.31491	37050.043
7	-5	0	1188.323	1.30559	13621.287
7	-2	0	1189.163	1.30559	13630.918
2	0	5	1125.902	1.30431	25758.510
6	-3	3	156.280	1.29521	3523.634
7	-5	1	800.605	1.28272	17694.859

7	-2	1	800.659	1.28272	17696.041
6	-4	3	924.498	1.27917	20318.115
6	-2	3	924.264	1.27917	20312.955
5	-3	4	456.856	1.26643	9840.050
5	-2	4	456.656	1.26643	9835.747
6	0	2	995.075	1.26394	21348.785
3	-1	5	1090.892	1.25695	23148.832
3	-2	5	1090.892	1.25695	23148.832
7	-4	2	652.424	1.24902	13674.297
7	-3	2	652.153	1.24902	13668.603
7	-6	0	1162.948	1.24338	12080.954
7	-1	0	1160.885	1.24338	12059.518
5	-4	4	1871.400	1.23694	38496.500
5	-1	4	1870.761	1.23694	38483.348
6	-5	3	1754.590	1.23440	35953.453
6	-1	3	1754.466	1.23440	35950.914
3	0	5	395.640	1.22810	8029.375
7	-6	1	1037.149	1.22358	20904.611
7	-1	1	1037.286	1.22358	20907.365
7	-5	2	1462.997	1.22071	29360.230
7	-2	2	1466.226	1.22071	29425.037
4	-2	5	301.458	1.18832	5768.571
5	0	4	380.189	1.18365	7227.019
8	-4	0	2509.864	1.17684	23628.283
4	-3	5	620.729	1.17590	11672.062
4	-1	5	620.729	1.17590	11672.062
7	-6	2	192.976	1.16941	3596.392
7	-1	2	192.254	1.16941	3582.923
6	0	3	383.914	1.16922	7152.987
8	-5	0	325.127	1.16477	3010.617
8	-3	0	324.961	1.16477	3009.080
7	0	0	324.961	1.16477	3009.080
8	-4	1	1912.631	1.16001	35196.359
6	-3	4	2371.925	1.15947	43617.062
7	-4	3	1177.474	1.15738	21592.775
7	-3	3	1177.047	1.15738	21584.934
8	-5	1	865.660	1.14844	15691.479
8	-3	1	865.875	1.14844	15695.383

7	0	1	865.983	1.14844	15697.332
6	-4	4	1350.330	1.14792	24460.523
6	-2	4	1349.839	1.14792	24451.625
0	0	6	15554.542	1.14722	46918.684
4	0	5	249.199	1.14084	4474.057
1	0	6	1074.694	1.13603	19180.771
7	-5	3	554.711	1.13474	9884.787
7	-2	3	554.711	1.13474	9884.785
8	-6	0	365.609	1.13067	3241.628
8	-2	0	365.576	1.13067	3241.339
8	-6	1	1686.896	1.11572	29404.143
8	-2	1	1687.347	1.11572	29411.990
6	-5	4	1823.343	1.11524	31765.707
6	-1	4	1830.446	1.11524	31889.445
2	-1	6	3635.795	1.11460	63297.172
8	-4	2	3577.241	1.11354	62205.703
5	-2	5	889.930	1.10875	15395.763
5	-3	5	889.930	1.10875	15395.768
2	0	6	3791.088	1.10433	65283.738
8	-5	2	1260.505	1.10330	21683.377
8	-3	2	1272.860	1.10330	21895.914
7	0	2	1277.033	1.10330	21967.686
7	-6	3	141.750	1.09318	2414.212
7	-1	3	141.375	1.09318	2407.836
5	-4	5	50.556	1.08880	857.543
5	-1	5	50.547	1.08880	857.384
8	-7	0	1256.042	1.07994	10570.117
8	-1	0	1257.315	1.07994	10580.832
3	-2	6	399.666	1.07514	6700.295
3	-1	6	399.770	1.07514	6702.033
8	-6	2	401.408	1.07419	6724.418
8	-2	2	400.038	1.07419	6701.472
8	-7	1	176.919	1.06689	2947.466
8	-1	1	176.956	1.06689	2948.082
6	0	4	341.225	1.06647	5683.081
7	-4	4	218.320	1.05746	3614.143
7	-3	4	218.055	1.05746	3609.753
3	0	6	415.939	1.05692	6883.266

5	0	5	285.095	1.05193	4703.988
8	-4	3	54.551	1.04714	897.752
9	-5	0	191.514	1.04393	1573.380
9	-4	0	191.514	1.04393	1573.380
7	-5	4	411.352	1.04011	6747.225
7	-2	4	411.136	1.04011	6743.680
8	-5	3	289.546	1.03860	4746.283
8	-3	3	289.525	1.03860	4745.940
7	0	3	289.484	1.03860	4745.257
6	-3	5	1237.040	1.03484	20248.086
9	-5	1	1486.659	1.03213	24310.967
9	-4	1	1486.792	1.03213	24313.141
4	-2	6	3248.068	1.03124	53099.590
8	-7	2	1570.708	1.03041	25671.254
8	-1	2	1569.579	1.03041	25652.812
9	-3	0	917.629	1.02723	7491.932
9	-6	0	917.629	1.02723	7491.932
6	-4	5	398.772	1.02661	6510.441
6	-2	5	398.741	1.02661	6509.934
4	-3	6	549.668	1.02309	8966.670
4	-1	6	549.486	1.02309	8963.699
8	0	0	1729.481	1.01917	14096.433
9	-6	1	774.072	1.01598	12613.213
9	-3	1	773.202	1.01598	12599.029
8	-6	3	478.777	1.01421	7800.216
8	-2	3	478.728	1.01421	7799.418
8	0	1	1496.745	1.00818	24380.072
7	-6	4	776.181	1.00783	12643.076
7	-1	4	775.188	1.00783	12626.909
6	-5	5	718.097	1.00303	11700.234
6	-1	5	717.839	1.00303	11696.044
4	0	6	982.877	0.99975	16020.969
9	-5	2	476.768	0.99899	7772.302
9	-4	2	476.689	0.99899	7771.009
9	-7	0	466.346	0.99609	3803.303
9	-2	0	465.255	0.99609	3794.407
9	-7	1	780.261	0.98583	12767.331
9	-2	1	780.405	0.98583	12769.685

9	-6	2	1137.894	0.98432	18630.975
9	-3	2	1137.484	0.98432	18624.256
5	-2	6	1251.484	0.97794	20555.795
5	-3	6	1251.484	0.97794	20555.795
8	0	2	2546.999	0.97723	41851.793
8	-7	3	1282.379	0.97712	21073.107
8	-1	3	1279.422	0.97712	21024.516
1	0	7	1738.929	0.97626	28589.918
8	-4	4	1890.158	0.97140	31173.926
6	0	5	312.627	0.96711	5172.616
8	-5	4	312.885	0.96458	5187.623
8	-3	4	313.272	0.96458	5194.043
7	0	4	313.272	0.96458	5194.044
5	-4	6	478.841	0.96417	7941.982
5	-1	6	478.763	0.96417	7940.696
2	-1	7	694.949	0.96256	11542.658
7	-3	5	431.511	0.96037	7181.559
7	-4	5	431.511	0.96037	7181.559
9	-7	2	150.934	0.95683	2520.821
9	-2	2	150.915	0.95683	2520.496
2	0	7	233.534	0.95592	3904.067
9	-8	0	219.785	0.95428	1840.347
9	-1	0	219.758	0.95428	1840.125
9	-5	3	360.569	0.95021	6066.718
9	-4	3	360.569	0.95021	6066.721
7	-5	5	422.572	0.94732	7135.412
7	-2	5	422.518	0.94732	7134.494
9	-8	1	519.376	0.94524	8793.867
9	-1	1	519.618	0.94524	8797.964
8	-6	4	506.904	0.94495	8586.052
8	-2	4	505.985	0.94495	8570.489
10	-5	0	1239.009	0.94147	10544.131
5	0	6	2136.673	0.93828	36539.133
9	-6	3	1179.459	0.93756	20192.463
9	-3	3	1179.459	0.93756	20192.465
3	-2	7	1029.390	0.93680	17644.186
3	-1	7	1029.390	0.93680	17644.186
10	-6	0	1325.599	0.93526	11388.625

10	-4	0	1325.518	0.93526	11387.935
10	-5	1	503.960	0.93279	8694.846
8	0	3	340.019	0.93142	5880.141
10	-6	1	447.081	0.92674	7797.324
10	-4	1	447.104	0.92674	7797.733
6	-3	6	887.914	0.92610	15504.534
3	0	7	268.005	0.92467	4692.761
7	-6	5	68.493	0.92274	1203.926
7	-1	5	68.441	0.92274	1203.016
6	-4	6	417.105	0.92018	7370.239
6	-2	6	417.288	0.92018	7373.475
9	-8	2	423.588	0.91959	7494.240
9	-1	2	424.894	0.91959	7517.347
10	-7	0	577.622	0.91733	5134.775
10	-3	0	576.839	0.91733	5127.819
8	-7	4	957.212	0.91473	17118.047
8	-1	4	956.137	0.91473	17098.834
9	-7	3	799.754	0.91371	14336.241
9	-2	3	799.614	0.91371	14333.736
10	-7	1	302.518	0.90929	5481.145
10	-3	1	302.538	0.90929	5481.514
10	-5	2	721.770	0.90811	13116.432
4	-2	7	417.338	0.90733	7599.229
9	0	0	499.931	0.90593	4568.228
6	-5	6	177.546	0.90309	3269.678
6	-1	6	176.542	0.90309	3251.183
10	-6	2	184.247	0.90253	3398.378
10	-4	2	184.151	0.90253	3396.601
4	-3	7	143.369	0.90176	2650.037
4	-1	7	143.346	0.90176	2649.603
9	0	1	169.152	0.89819	3159.076
8	-4	5	153.679	0.89454	2902.189
9	-5	4	0.413	0.89254	7.843
9	-4	4	0.413	0.89254	7.843
10	-8	0	86.072	0.88961	825.830
10	-2	0	86.094	0.88961	826.041

La3-HAp CIF FILE

```
#==============================================================================
data_global
#==============================================================================
_publ_contact_author_name  'Dr. Francesco Capitelli'
_publ_contact_author_address
;
  Institute of Crystallography - CNR
  V. Salaria Km 29.300, 00015 Monterotondo (Rome), Italy
;
_publ_contact_author_email    francesco.capitelli@ic.cnr.it
_publ_contact_author_fax     '+39 06 90672616'
_publ_contact_author_phone   '+39 06 90672630'
_journale_name_full          ?
_publ_requested_category     FI
_audit_creation_method              Expo2014

_chemical_name_systematic     ?
_chemical_formula_moiety      'H Ca5 La0.04 O13 P3'
_chemical_formula_sum         'H Ca5 La0.04 O13 P3'
_chemical_formula_weight      505.974

loop_
     _atom_type_symbol
     _atom_type_description
     _atom_type_scat_source
    'H'   'Hydrogen'   'International Tables Vol C Tables 4.2.6.8 and 6.1.1.4'
    'O'   'Oxygen'   'International Tables Vol C Tables 4.2.6.8 and 6.1.1.4'
    'P'   'Phosphorus'   'International Tables Vol C Tables 4.2.6.8 and 6.1.1.4'
    'Ca'   'Calcium'   'International Tables Vol C Tables 4.2.6.8 and 6.1.1.4'
    'La'   'Lanthanum'   'International Tables Vol C Tables 4.2.6.8 and 6.1.1.4'

_cell_length_a                  9.41670(19)
_cell_length_b                  9.41670(19)
_cell_length_c                  6.8835(3)
_cell_angle_alpha               90.000
```

```
_cell_angle_beta                  90.000
_cell_angle_gamma                 120.000
_cell_volume                      528.61(3)
_cell_formula_units_Z             2
_exptl_crystal_description    powder
_exptl_crystal_colour           ?
_cell_measurement_temperature    ?

_exptl_crystal_density_diffrn         3.179
_exptl_crystal_density_meas      ?
_exptl_crystal_density_method 'not measured'
_exptl_absorpt_coefficient_mu        28.215
_symmetry_Int_Tables_number         176
_symmetry_cell_setting              hexagonal
_symmetry_space_group_name_H-M      'P 63/m'
_symmetry_space_group_name_hall     '-P 6c'

loop_
    _symmetry_equiv_pos_site_id
    _symmetry_equiv_pos_as_xyz
    1   'x, y, z'
    2   'x-y, x, z+1/2'
    3   '-y, x-y, z'
    4   '-x, -y, z+1/2'
    5   '-x+y, -x, z'
    6   'y, -x+y, z+1/2'
    7   '-x, -y, -z'
    8   '-x+y, -x, -z+1/2'
    9   'y, -x+y, -z'
   10   'x, y, -z+1/2'
   11   'x-y, x, -z'
   12   '-y, x-y, -z+1/2'

loop_
    _atom_site_type_symbol
    _atom_site_label
    _atom_site_fract_x
    _atom_site_fract_y
```

```
    _atom_site_fract_z
    _atom_site_U_iso_or_equiv
    _atom_site_occupancy
    _atom_site_adp_type
Ca  Ca1 0.6667 0.3333 0.9978(6) 0.0061(6) 1.0000       Uiso
Ca  Ca2 0.2523(3) 0.2447(3) 0.7500 0.0040 0.9876(18) Uiso
La  La1 0.2523 0.2447 0.7500 0.0040(6) 0.0124(18) Uiso
P   P   0.3979(4) 0.3673(4) 1.2500 0.0019(8) 1.0000       Uiso
O   O1  0.3272(7) 0.4835(8) 1.2500 0.004(2) 1.0000       Uiso
O   O2  0.5890(8) 0.4648(9) 1.2500 0.007(2) 1.0000       Uiso
O   O3  0.3372(5) 0.2527(6) 1.0710(6) 0.0166(15) 1.0000       Uiso
O   O-H 0.0000 0.0000 0.701(3) 0.001(4) 0.50000      Uiso
H   H   0.0000 0.0000 0.5622 0.0006 0.50000      Uiso

loop_
    _geom_bond_atom_site_label_1
    _geom_bond_atom_site_label_2
    _geom_bond_distance
    _geom_bond_site_symmetry_2
Ca1 O1  2.406(5) 6_554
Ca1 O1  2.406(6) 4_664
Ca1 O1  2.406(6) 2_654
Ca1 O2  2.447(7) .
Ca1 O2  2.447(6) 5_665
Ca1 O2  2.447(5) 3_655
Ca1 O3  2.846(5) .
Ca1 O3  2.846(4) 5_665
Ca1 O3  2.846(6) 3_655
Ca2 O1  2.691(9) 6_554
Ca2 O2  2.372(7) 4_664
Ca2 O3  2.338(4) 10_556
Ca2 O3  2.480(6) 2_554
Ca2 O3  2.480(6) 11_557
Ca2 O3  2.338(4) .
Ca2 O-H  2.365(4) .
P O1  1.538(10) .
P O2  1.559(7) .
P O3  1.547(5) .
```

```
P O3  1.547(5) 10_557
O1 Ca1  2.406(6) 7_667
O1 Ca1  2.406(6) 2_555
O1 Ca2  2.691(6) 2_555
O2 Ca1  2.447(7) 8_667
O2 Ca2  2.372(7) 4_665
O3 Ca2  2.480(5) 6_555
O-H Ca2  2.365(4) 3_555
O-H Ca2  2.365(4) 5_555
O-H H  0.957489 .

loop_
_geom_angle_atom_site_label_1
_geom_angle_atom_site_label_2
_geom_angle_atom_site_label_3
_geom_angle
_geom_angle_site_symmetry_1
_geom_angle_site_symmetry_3
O1 Ca1 O1 75.3(2) 6_554 4_664
O1 Ca1 O1 75.3(2) 6_554 2_654
O1 Ca1 O2 124.2(2) 6_554 .
O1 Ca1 O2 154.3(2) 6_554 5_665
O1 Ca1 O2 92.7(2) 6_554 3_655
O1 Ca1 O3 68.43(18) 6_554 .
O1 Ca1 O3 142.7(2) 6_554 5_665
O1 Ca1 O3 87.07(18) 6_554 3_655
O1 Ca1 O1 75.3(2) 4_664 2_654
O1 Ca1 O2 92.7(2) 4_664 .
O1 Ca1 O2 124.2(2) 4_664 5_665
O1 Ca1 O2 154.3(2) 4_664 3_655
O1 Ca1 O3 87.07(18) 4_664 .
O1 Ca1 O3 68.43(18) 4_664 5_665
O1 Ca1 O3 142.7(2) 4_664 3_655
O1 Ca1 O2 154.3(2) 2_654 .
O1 Ca1 O2 92.7(2) 2_654 5_665
O1 Ca1 O2 124.2(2) 2_654 3_655
O1 Ca1 O3 142.7(2) 2_654 .
O1 Ca1 O3 87.07(18) 2_654 5_665
```

O1 Ca1 O3 68.43(18) 2_654 3_655

O2 Ca1 O2 75.2(2) . 5_665

O2 Ca1 O2 75.2(2) . 3_655

O2 Ca1 O3 56.47(19) . .

O2 Ca1 O3 67.31(19) . 5_665

O2 Ca1 O3 124.1(2) . 3_655

Ca1 O2 P 104.5(3) . .

Ca1 O2 Ca1 90.4(2) . 8_667

Ca1 O2 Ca2 114.1(3) . 4_665

O2 Ca1 O2 75.2(2) 5_665 3_655

O2 Ca1 O3 124.1(2) 5_665 .

O2 Ca1 O3 56.47(19) 5_665 5_665

O2 Ca1 O3 67.31(19) 5_665 3_655

O2 Ca1 O3 67.31(19) 3_655 .

O2 Ca1 O3 124.1(2) 3_655 5_665

O2 Ca1 O3 56.47(19) 3_655 3_655

O3 Ca1 O3 116.93(16) . 5_665

O3 Ca1 O3 116.93(16) . 3_655

Ca1 O3 Ca2 98.67(18) . .

Ca1 O3 P 89.0(2) . .

Ca1 O3 Ca2 98.55(17) . 6_555

O3 Ca1 O3 116.93(16) 5_665 3_655

O1 Ca2 O2 102.5(2) 6_554 4_664

O1 Ca2 O3 72.16(18) 6_554 10_556

O1 Ca2 O3 149.84(19) 6_554 2_554

O1 Ca2 O3 149.84(19) 6_554 11_557

O1 Ca2 O3 72.16(18) 6_554 .

O1 Ca2 O-H 105.8(4) 6_554 .

O2 Ca2 O3 87.4(2) 4_664 10_556

O2 Ca2 O3 74.9(2) 4_664 2_554

O2 Ca2 O3 74.9(2) 4_664 11_557

O2 Ca2 O3 87.4(2) 4_664 .

O2 Ca2 O-H 150.5(4) 4_664 .

O3 Ca2 O3 77.69(17) 10_556 2_554

O3 Ca2 O3 136.53(18) 10_556 11_557

O3 Ca2 O3 141.84(19) 10_556 .

O3 Ca2 O-H 93.3(4) 10_556 .

O3 Ca2 O3 59.57(16) 2_554 11_557

```
O3 Ca2 O3 136.53(18) 2_554 .
O3 Ca2 O-H 76.4(3) 2_554 .
O3 Ca2 O3 77.69(17) 11_557 .
O3 Ca2 O-H 84.6(3) 11_557 .
O3 Ca2 O-H 109.0(4) . .
Ca2 O3 P 140.9(3) . .
Ca2 O3 Ca2 119.5(2) . 6_555
Ca2 O-H Ca2 118.0(6) . 3_555
Ca2 O-H Ca2 118.0(6) . 5_555
Ca2 O-H H      98.15       .       .
O1 P O2 111.3(4) . .
O1 P O3 110.8(3) . .
O1 P O3 110.8(3) . 10_557
P O1 Ca1 130.2(3) . 7_667
P O1 Ca1 130.2(3) . 2_555
P O1 Ca2 97.6(3) . 2_555
O2 P O3 109.0(3) . .
O2 P O3 109.0(3) . 10_557
P O2 Ca1 104.5(3) . 8_667
P O2 Ca2 123.7(4) . 4_665
O3 P O3 105.6(3) . 10_557
P O3 Ca2 96.8(2) . 6_555
Ca1 O1 Ca1 90.3(2) 7_667 2_555
Ca1 O1 Ca2 101.3(2) 7_667 2_555
Ca1 O1 Ca2 101.3(2) 2_555 2_555
Ca1 O2 Ca2 114.1(3) 8_667 4_665
Ca2 O-H Ca2 118.0(6) 3_555 5_555
Ca2 O-H H      98.15   3_555       .
Ca2 O-H H      98.15   5_555       .

loop_
   _geom_torsion_atom_site_label_1
   _geom_torsion_atom_site_label_2
   _geom_torsion_atom_site_label_3
   _geom_torsion_atom_site_label_4
   _geom_torsion
   _geom_torsion_site_symmetry_1
   _geom_torsion_site_symmetry_2
```

_geom_torsion_site_symmetry_3
_geom_torsion_site_symmetry_4
O1 Ca1 O1 Ca2 -62.4(2) 4_664 . 6_554 .
O1 Ca1 O1 Ca2 -140.8(2) 2_654 . 6_554 .
O2 Ca1 O1 Ca2 20.7(3) . . 6_554 .
O2 Ca1 O1 Ca2 155.0(5) 5_665 . 6_554 .
O2 Ca1 O1 Ca2 94.6(2) 3_655 . 6_554 .
O3 Ca1 O1 Ca2 30.26(17) . . 6_554 .
O3 Ca1 O1 Ca2 -76.3(3) 5_665 . 6_554 .
O3 Ca1 O1 Ca2 150.78(18) 3_655 . 6_554 .
O1 Ca1 O2 P 17.0(4) 6_554 . . .
O1 Ca1 O2 Ca1 122.2(2) 6_554 . . 8_667
O1 Ca1 O2 Ca2 -121.1(3) 6_554 . . 4_665
O1 Ca1 O2 P 91.0(3) 4_664 . . .
O1 Ca1 O2 Ca1 -163.8(2) 4_664 . . 8_667
O1 Ca1 O2 Ca2 -47.1(2) 4_664 . . 4_665
O1 Ca1 O2 P 151.7(4) 2_654 . . .
O1 Ca1 O2 Ca1 -103.2(5) 2_654 . . 8_667
O1 Ca1 O2 Ca2 13.6(6) 2_654 . . 4_665
O2 Ca1 O2 P -144.3(3) 5_665 . . .
O2 Ca1 O2 Ca1 -39.1(2) 5_665 . . 8_667
O2 Ca1 O2 Ca2 77.6(2) 5_665 . . 4_665
O2 Ca1 O2 P -66.0(3) 3_655 . . .
O2 Ca1 O2 Ca1 39.1(2) 3_655 . . 8_667
O2 Ca1 O2 Ca2 155.9(3) 3_655 . . 4_665
O3 Ca1 O2 P 6.3(2) . . . .
O3 Ca1 O2 Ca1 111.5(2) . . . 8_667
O3 Ca1 O2 Ca2 -131.8(3) . . . 4_665
O3 Ca1 O2 P 156.3(3) 5_665 . . .
O3 Ca1 O2 Ca1 -98.5(2) 5_665 . . 8_667
O3 Ca1 O2 Ca2 18.2(2) 5_665 . . 4_665
O3 Ca1 O2 P -95.6(3) 3_655 . . .
O3 Ca1 O2 Ca1 9.5(3) 3_655 . . 8_667
O3 Ca1 O2 Ca2 126.3(2) 3_655 . . 4_665
O1 Ca1 O3 Ca2 -35.13(19) 6_554 . . .
O1 Ca1 O3 P -176.6(3) 6_554 . . .
O1 Ca1 O3 Ca2 86.6(2) 6_554 . . 6_555
O1 Ca1 O3 Ca2 40.2(2) 4_664 . . .

O1 Ca1 O3 P -101.3(2) 4_664 . . .

O1 Ca1 O3 Ca2 161.99(19) 4_664 . . 6_555

O1 Ca1 O3 Ca2 -20.7(4) 2_654 . . .

O1 Ca1 O3 P -162.2(3) 2_654 . . .

O1 Ca1 O3 Ca2 101.1(3) 2_654 . . 6_555

O2 Ca1 O3 Ca2 135.3(3) . . . .

O2 Ca1 O3 P -6.2(2) . . . .

O2 Ca1 O3 Ca2 -102.9(2) . . . 6_555

O2 Ca1 O3 Ca2 170.4(2) 5_665 . . .

O2 Ca1 O3 P 28.9(3) 5_665 . . .

O2 Ca1 O3 Ca2 -67.9(3) 5_665 . . 6_555

O2 Ca1 O3 Ca2 -137.8(2) 3_655 . . .

O2 Ca1 O3 P 80.7(2) 3_655 . . .

O2 Ca1 O3 Ca2 -16.0(2) 3_655 . . 6_555

O3 Ca1 O3 Ca2 104.2(2) 5_665 . . .

O3 Ca1 O3 P -37.3(3) 5_665 . . .

O3 Ca1 O3 Ca2 -134.04(18) 5_665 . . 6_555

O3 Ca1 O3 Ca2 -109.9(2) 3_655 . . .

O3 Ca1 O3 P 108.6(2) 3_655 . . .

O3 Ca1 O3 Ca2 11.9(2) 3_655 . . 6_555

O2 Ca2 O1 Ca1 46.3(2) 4_664 . 6_554 .

O3 Ca2 O1 Ca1 129.4(2) 10_556 . 6_554 .

O3 Ca2 O1 Ca1 127.7(3) 2_554 . 6_554 .

O3 Ca2 O1 Ca1 -35.1(4) 11_557 . 6_554 .

O3 Ca2 O1 Ca1 -36.82(19) . . 6_554 .

O-H Ca2 O1 Ca1 -142.2(5) . . 6_554 .

O1 Ca2 O3 Ca1 30.17(17) 6_554 . . .

O1 Ca2 O3 P 129.3(4) 6_554 . . .

O1 Ca2 O3 Ca2 -74.8(2) 6_554 . . 6_555

O2 Ca2 O3 Ca1 -73.8(2) 4_664 . . .

O2 Ca2 O3 P 25.3(4) 4_664 . . .

O2 Ca2 O3 Ca2 -178.9(2) 4_664 . . 6_555

O3 Ca2 O3 Ca1 8.7(3) 10_556 . . .

O3 Ca2 O3 P 107.8(4) 10_556 . . .

O3 Ca2 O3 Ca2 -96.4(3) 10_556 . . 6_555

O3 Ca2 O3 Ca1 -138.6(2) 2_554 . . .

O3 Ca2 O3 P -39.5(5) 2_554 . . .

O3 Ca2 O3 Ca2 116.4(3) 2_554 . . 6_555

O3 Ca2 O3 Ca1 -148.96(19) 11_557 . . .

O3 Ca2 O3 P -49.8(4) 11_557 . . .

O3 Ca2 O3 Ca2 106.0(2) 11_557 . . 6_555

O-H Ca2 O3 Ca1 131.2(5) . . . .

O-H Ca2 O3 P -129.7(6) . . . .

O-H Ca2 O3 Ca2 26.2(6) . . . 6_555

O1 Ca2 O-H Ca2 168.5(5) 6_554 . . 3_555

O1 Ca2 O-H Ca2 16.1(6) 6_554 . . 5_555

O1 Ca2 O-H H -87.68 6_554 . . .

O2 Ca2 O-H Ca2 -28.4(11) 4_664 . . 3_555

O2 Ca2 O-H Ca2 179.1(4) 4_664 . . 5_555

O2 Ca2 O-H H 75.35 4_664 . . .

O3 Ca2 O-H Ca2 -119.1(6) 10_556 . . 3_555

O3 Ca2 O-H Ca2 88.5(6) 10_556 . . 5_555

O3 Ca2 O-H H -15.28 10_556 . . .

O3 Ca2 O-H Ca2 -42.6(5) 2_554 . . 3_555

O3 Ca2 O-H Ca2 165.0(6) 2_554 . . 5_555

O3 Ca2 O-H H 61.20 2_554 . . .

O3 Ca2 O-H Ca2 17.4(5) 11_557 . . 3_555

O3 Ca2 O-H Ca2 -135.0(6) 11_557 . . 5_555

O3 Ca2 O-H H 121.17 11_557 . . .

O3 Ca2 O-H Ca2 92.4(6) . . . 3_555

O3 Ca2 O-H Ca2 -60.1(6) . . . 5_555

O3 Ca2 O-H H -163.84 . . . .

O2 P O1 Ca1 68.2(4) . . . 7_667

O2 P O1 Ca1 -68.2(4) . . . 2_555

O2 P O1 Ca2 180.0(3) . . . 2_555

O3 P O1 Ca1 -53.3(4) . . . 7_667

O3 P O1 Ca1 170.3(3) . . . 2_555

O3 P O1 Ca2 58.5(3) . . . 2_555

O3 P O1 Ca1 -170.3(3) 10_557 . . 7_667

O3 P O1 Ca1 53.3(4) 10_557 . . 2_555

O3 P O1 Ca2 -58.5(3) 10_557 . . 2_555

O1 P O2 Ca1 -132.9(3) . . . .

O1 P O2 Ca1 132.9(3) . . . 8_667

O1 P O2 Ca2 0.0(4) . . . 4_665

O3 P O2 Ca1 -10.3(3) . . . .

O3 P O2 Ca1 -104.5(3) . . . 8_667

```
O3 P O2 Ca2 122.6(3) . . . 4_665
O3 P O2 Ca1 104.5(3) 10_557 . . .
O3 P O2 Ca1 10.3(3) 10_557 . . 8_667
O3 P O2 Ca2 -122.6(3) 10_557 . . 4_665
O1 P O3 Ca1 131.4(2) . . . .
O1 P O3 Ca2 28.9(5) . . . .
O1 P O3 Ca2 -130.1(2) . . . 6_555
O2 P O3 Ca1 8.5(3) . . . .
O2 P O3 Ca2 -94.0(4) . . . .
O2 P O3 Ca2 107.0(3) . . . 6_555
O3 P O3 Ca1 -108.5(2) 10_557 . . .
O3 P O3 Ca2 149.0(3) 10_557 . . .
O3 P O3 Ca2 -10.0(3) 10_557 . . 6_555

_diffrn_ambient_temperature     ?
_diffrn_radiation_wavelength     1.540560
_diffrn_radiation_type          'Cu K\a~1~'
_diffrn_measurement_device_type ?

# POWDER PROFILE

_pd_meas_2theta_range_min      6.000
_pd_meas_2theta_range_max    120.000
_pd_proc_number_of_points       5701

_pd_proc_ls_prof_R_factor           0.02543
_pd_proc_ls_prof_wR_factor          0.04270
_pd_proc_ls_prof_wR_expected        0.04906

_refine_ls_number_reflns      288
_refine_ls_number_parameters  57
_refine_ls_number_restraints  0
_refine_ls_hydrogen_treatment  constr
_refine_ls_R_I_factor               0.10012
_refine_ls_goodness_of_fit_all      0.87032
_refine_ls_restrained_S_all         0.87032

loop_
```

_pd_meas_2theta_scan

_pd_meas_counts_total

_pd_proc_2theta_corrected

_pd_calc_intensity_total

_pd_proc_intensity_bkg_calc

6.000000	3530.000000	6.010715	0.000000	3521.757812
6.020000	3510.330078	6.030715	0.000000	3510.520020
6.040000	3566.330078	6.050714	0.000000	3499.322266
6.060000	3475.670166	6.070714	0.000000	3488.159180
6.080000	3469.670166	6.090714	0.000000	3477.034180
6.100000	3485.670166	6.110714	0.000000	3465.949707
6.120000	3465.000000	6.130714	0.000000	3454.903320
6.140000	3468.500000	6.150714	0.000000	3443.893066
6.160000	3447.670166	6.170714	0.000000	3432.923340
6.180000	3445.170166	6.190714	0.000000	3421.991211
6.200000	3420.500000	6.210713	0.000000	3411.099121
6.220000	3388.830078	6.230713	0.000000	3400.243652
6.240000	3376.000000	6.250713	0.000000	3389.429199
6.260000	3405.500000	6.270713	0.000000	3378.653320
6.280000	3359.500000	6.290713	0.000000	3367.915039
6.300000	3365.330078	6.310714	0.000000	3357.216797
6.320000	3334.000000	6.330714	0.000000	3346.557617
6.340000	3364.500000	6.350713	0.000000	3335.939453
6.360000	3337.500000	6.370713	0.000000	3325.357910
6.380000	3284.330078	6.390713	0.000000	3314.815430
6.400000	3292.000000	6.410713	0.000000	3304.313477
6.420000	3304.000000	6.430713	0.000000	3293.852539
6.440000	3270.000000	6.450713	0.000000	3283.429688
6.460000	3252.830078	6.470713	0.000000	3273.045410
6.480000	3259.500000	6.490713	0.000000	3262.700684
6.500000	3291.500000	6.510713	0.000000	3252.397461
6.520000	3253.830078	6.530713	0.000000	3242.132812
6.540000	3212.500000	6.550713	0.000000	3231.906250
6.560000	3209.170166	6.570713	0.000000	3221.720215
6.580000	3233.500000	6.590713	0.000000	3211.575195
6.600000	3193.000000	6.610713	0.000000	3201.469238
6.620000	3219.330078	6.630712	0.000000	3191.402344

6.640000	3213.500000	6.650712	0.000000	3181.376953
6.660000	3218.500000	6.670712	0.000000	3171.389160
6.680000	3185.000000	6.690712	0.000000	3161.441895
6.700000	3147.830078	6.710712	0.000000	3151.531738
6.720000	3135.000000	6.730712	0.000000	3141.665039
6.740000	3127.330078	6.750712	0.000000	3131.835449
6.760000	3109.670166	6.770711	0.000000	3122.046387
6.780000	3115.330078	6.790711	0.000000	3112.298340
6.800000	3151.500000	6.810712	0.000000	3102.586914
6.820000	3093.670166	6.830712	0.000000	3092.916016
6.840000	3090.170166	6.850712	0.000000	3083.285156
6.860000	3088.500000	6.870712	0.000000	3073.692871
6.880000	3035.500000	6.890712	0.000000	3064.142578
6.900000	3030.170166	6.910711	0.000000	3054.628418
6.920000	3009.330078	6.930711	0.000000	3045.153809
6.940000	3008.000000	6.950711	0.000000	3035.720703
6.960000	3002.170166	6.970711	0.000000	3026.326172
6.980000	3027.500000	6.990711	0.000000	3016.969727
7.000000	2994.830078	7.010711	0.000000	3007.653809
7.020000	3003.500000	7.030711	0.000000	2998.376465
7.040000	3023.500000	7.050711	0.000000	2989.138184
7.060000	3002.330078	7.070711	0.000000	2979.937500
7.080000	2984.170166	7.090711	0.000000	2970.776855
7.100000	2972.830078	7.110711	0.000000	2961.656250
7.120000	2951.330078	7.130711	0.000000	2952.572266
7.140000	2966.830078	7.150711	0.000000	2943.528320
7.160000	2933.830078	7.170711	0.000000	2934.521484
7.180000	2890.670166	7.190710	0.000000	2925.555664
7.200000	2945.500000	7.210710	0.000000	2916.626465
7.220000	2897.670166	7.230710	0.000000	2907.735840
7.240000	2909.830078	7.250710	0.000000	2898.885254
7.260000	2851.500000	7.270710	0.000000	2890.070801
7.280000	2880.830078	7.290710	0.000000	2881.294434
7.300000	2849.000000	7.310710	0.000000	2872.556641
7.320000	2865.830078	7.330710	0.000000	2863.858398
7.340000	2817.670166	7.350710	0.000000	2855.196777
7.360000	2832.170166	7.370710	0.000000	2846.572266
7.380000	2806.500000	7.390710	0.000000	2837.985840

7.400000	2818.000000	7.410710	0.000000	2829.437988
7.420000	2790.330078	7.430710	0.000000	2820.925781
7.440000	2827.330078	7.450710	0.000000	2812.452637
7.460000	2798.830078	7.470709	0.000000	2804.016602
7.480000	2784.170166	7.490709	0.000000	2795.617676
7.500000	2754.500000	7.510709	0.000000	2787.255371
7.520000	2794.330078	7.530709	0.000000	2778.929688
7.540000	2743.170166	7.550709	0.000000	2770.642578
7.560000	2745.500000	7.570709	0.000000	2762.390625
7.580000	2712.330078	7.590709	0.000000	2754.176270
7.600000	2739.000000	7.610709	0.000000	2745.997559
7.620000	2756.170166	7.630709	0.000000	2737.856934
7.640000	2744.500000	7.650709	0.000000	2729.751465
7.660000	2687.830078	7.670709	0.000000	2721.682129
7.680000	2699.670166	7.690709	0.000000	2713.648438
7.700000	2677.000000	7.710709	0.000000	2705.652344
7.720000	2680.500000	7.730709	0.000000	2697.691406
7.740000	2717.670166	7.750708	0.000000	2689.765625
7.760000	2679.170166	7.770708	0.000000	2681.876953
7.780000	2666.670166	7.790708	0.000000	2674.022461
7.800000	2645.500000	7.810709	0.000000	2666.202637
7.820000	2666.500000	7.830709	0.000000	2658.419434
7.840000	2623.330078	7.850708	0.000000	2650.670410
7.860000	2643.000000	7.870708	0.000000	2642.958008
7.880000	2654.170166	7.890708	0.000000	2635.278809
7.900000	2606.330078	7.910708	0.000000	2627.635254
7.920000	2607.000000	7.930708	0.000000	2620.026855
7.940000	2610.000000	7.950708	0.000000	2612.452637
7.960000	2589.830078	7.970708	0.000000	2604.911621
7.980000	2618.670166	7.990708	0.000000	2597.405273
8.000000	2558.170166	8.010708	0.000000	2589.934570
8.020000	2554.500000	8.030708	0.000000	2582.496094
8.040000	2560.830078	8.050708	0.000000	2575.092773
8.060000	2532.500000	8.070708	0.000000	2567.720703
8.080000	2543.830078	8.090708	0.000000	2560.385742
8.100000	2542.670166	8.110708	0.000000	2553.081543
8.120001	2539.170166	8.130709	0.000000	2545.811523
8.140000	2528.670166	8.150708	0.000000	2538.574219

8.160001	2525.330078	8.170709	0.000000	2531.370117
8.180000	2518.670166	8.190708	0.000000	2524.200195
8.200000	2544.670166	8.210708	0.000000	2517.062012
8.220000	2503.330078	8.230708	0.000000	2509.955566
8.240000	2507.670166	8.250707	0.000000	2502.884277
8.260000	2477.330078	8.270707	0.000000	2495.843750
8.280000	2463.670166	8.290707	0.000000	2488.834961
8.300000	2476.830078	8.310707	0.000000	2481.858887
8.320001	2444.500000	8.330708	0.000000	2474.914551
8.340000	2436.830078	8.350707	0.000000	2468.001953
8.360001	2469.830078	8.370708	0.000000	2461.119629
8.380000	2415.000000	8.390707	0.000000	2454.271484
8.400000	2412.500000	8.410707	0.000000	2447.453125
8.420000	2439.000000	8.430707	0.000000	2440.666016
8.440000	2423.830078	8.450706	0.000000	2433.909668
8.460000	2434.000000	8.470707	0.000000	2427.186035
8.480000	2440.830078	8.490706	0.000000	2420.491211
8.500000	2400.000000	8.510707	0.000000	2413.827148
8.520000	2393.000000	8.530706	0.000000	2407.193848
8.540000	2436.000000	8.550706	0.000000	2400.592285
8.560000	2387.170166	8.570706	0.000000	2394.019531
8.580000	2410.000000	8.590706	0.000000	2387.477051
8.600000	2391.170166	8.610706	0.000000	2380.964355
8.620001	2362.830078	8.630707	0.000000	2374.481934
8.640000	2345.670166	8.650706	0.000000	2368.029297
8.660001	2374.830078	8.670707	0.000000	2361.605957
8.680000	2351.330078	8.690706	0.000000	2355.211914
8.700000	2363.670166	8.710706	0.000000	2348.846191
8.720000	2299.830078	8.730706	0.000000	2342.510254
8.740000	2318.830078	8.750706	0.000000	2336.203125
8.760000	2321.830078	8.770706	0.000000	2329.924805
8.780000	2341.500000	8.790706	0.000000	2323.675781
8.800000	2346.330078	8.810705	0.000000	2317.454590
8.820001	2347.330078	8.830706	0.000000	2311.261719
8.840000	2303.000000	8.850705	0.000000	2305.097656
8.860001	2272.000000	8.870706	0.000000	2298.960938
8.880000	2304.170166	8.890705	0.000000	2292.851562
8.900000	2256.670166	8.910705	0.000000	2286.770996

8.920000	2290.830078	8.930705	0.000000	2280.718262
8.940000	2275.670166	8.950705	0.000000	2274.691895
8.960000	2284.830078	8.970705	0.000000	2268.692871
8.980000	2278.330078	8.990705	0.000000	2262.722656
9.000000	2249.170166	9.010705	0.000000	2256.777344
9.020000	2266.500000	9.030705	0.000000	2250.859863
9.040000	2243.670166	9.050705	0.000000	2244.968750
9.060000	2241.330078	9.070705	0.000000	2239.104492
9.080000	2226.170166	9.090704	0.000000	2233.268066
9.100000	2255.000000	9.110704	0.000000	2227.456543
9.120001	2209.170166	9.130705	0.000000	2221.670898
9.140000	2237.000000	9.150704	0.000000	2215.912598
9.160001	2200.670166	9.170705	0.000000	2210.179688
9.180000	2195.500000	9.190704	0.000000	2204.471680
9.200000	2193.000000	9.210704	0.000000	2198.790039
9.220000	2204.670166	9.230704	0.000000	2193.133789
9.240000	2191.330078	9.250704	0.000000	2187.502441
9.260000	2177.500000	9.270704	0.000000	2181.896484
9.280000	2180.830078	9.290704	0.000000	2176.316406
9.300000	2193.830078	9.310704	0.000000	2170.759766
9.320001	2194.830078	9.330705	0.000000	2165.229004
9.340000	2187.500000	9.350704	0.000000	2159.721680
9.360001	2140.170166	9.370704	0.067703	2154.240723
9.380000	2135.830078	9.390703	0.070037	2148.782715
9.400000	2127.500000	9.410703	0.072486	2143.349121
9.420000	2153.000000	9.430703	0.075058	2137.938477
9.440000	2111.670166	9.450703	0.077759	2132.554199
9.460000	2097.000000	9.470703	0.080598	2127.192383
9.480000	2108.000000	9.490703	0.083585	2121.854004
9.500000	2121.500000	9.510703	0.086730	2116.540527
9.520000	2110.670166	9.530704	0.090044	2111.248535
9.540000	2109.500000	9.550703	0.093538	2105.980469
9.560000	2119.170166	9.570704	0.097225	2100.735352
9.580000	2095.000000	9.590703	0.101120	2095.513672
9.600000	2096.830078	9.610703	0.105237	2090.315430
9.620001	2088.670166	9.630704	0.109594	2085.138184
9.640000	2054.170166	9.650703	0.114208	2079.984863
9.660001	2082.330078	9.670703	0.119099	2074.854004

9.680000	2055.830078	9.690702	0.124290	2069.745117
9.700000	2084.170166	9.710702	0.129803	2064.658203
9.720000	2050.830078	9.730702	0.135667	2059.592773
9.740000	2083.000000	9.750702	0.141909	2054.550781
9.760000	2034.669922	9.770702	0.148562	2049.529785
9.780000	2036.669922	9.790702	0.155660	2044.530151
9.800000	2027.830078	9.810702	0.163245	2039.552124
9.820001	2023.500000	9.830703	0.171359	2034.596069
9.840000	2048.000000	9.850702	0.180049	2029.661011
9.860001	2044.500000	9.870703	0.189372	2024.746216
9.880000	2029.330078	9.890702	0.199386	2019.854126
9.900000	2024.169922	9.910702	0.210159	2014.981079
9.920000	2005.500000	9.930702	0.221768	2010.129517
9.940000	2017.330078	9.950701	0.234296	2005.299927
9.960000	2004.330078	9.970701	0.247842	2000.488892
9.980000	2030.830078	9.990701	0.262512	1995.698364
10.000000	1989.669922	10.010701	0.278431	1990.929077
10.020000	1983.830078	10.030702	0.295739	1986.179321
10.040000	1997.669922	10.050701	0.314594	1981.450562
10.060000	1990.000000	10.070702	0.335181	1976.740356
10.080000	1965.500000	10.090701	0.357708	1972.049927
10.100000	1954.330078	10.110702	0.382420	1967.380005
10.120001	1962.830078	10.130702	0.409595	1962.728638
10.140000	1955.000000	10.150702	0.439557	1958.098022
10.160001	1985.000000	10.170702	0.472688	1953.485962
10.180000	1958.169922	10.190701	0.509430	1948.892700
10.200000	1984.169922	10.210701	0.550307	1944.319214
10.220000	1959.669922	10.230700	0.595937	1939.764282
10.240000	1952.169922	10.250700	0.647056	1935.227661
10.260000	1938.830078	10.270700	0.704547	1930.710571
10.280000	1945.000000	10.290700	0.769460	1926.211060
10.300000	1923.000000	10.310700	0.843085	1921.730591
10.320001	1932.000000	10.330701	0.926979	1917.268677
10.340000	1917.000000	10.350700	1.023055	1912.824829
10.360001	1919.000000	10.370701	1.133697	1908.399048
10.380000	1918.330078	10.390700	1.261858	1903.991089
10.400000	1923.669922	10.410700	1.411277	1899.599976
10.420000	1884.000000	10.430700	1.586719	1895.228394

10.440000	1849.000000	10.450700	1.794286	1890.874146
10.460000	1888.669922	10.470700	2.041970	1886.537231
10.480000	1881.169922	10.490700	2.340272	1882.217407
10.500000	1898.830078	10.510699	2.703296	1877.915161
10.520000	1903.169922	10.530700	3.150251	1873.630005
10.540000	1896.830078	10.550699	3.707691	1869.362427
10.560000	1873.669922	10.570700	4.413305	1865.110962
10.580000	1870.000000	10.590699	5.321685	1860.876831
10.600000	1898.169922	10.610700	6.515646	1856.658813
10.620001	1888.169922	10.630700	8.131339	1852.458130
10.640000	1854.669922	10.650700	10.430826	1848.274536
10.660001	1884.830078	10.670700	14.017559	1844.106567
10.680000	1890.830078	10.690700	20.377457	1839.955200
10.700000	1923.330078	10.710699	32.858463	1835.820435
10.720000	1943.830078	10.730700	57.540642	1831.701294
10.740000	1927.000000	10.750699	102.347626	1827.598267
10.760000	2022.830078	10.770700	173.592804	1823.511108
10.780000	2085.000000	10.790698	275.208893	1819.440308
10.800000	2201.330078	10.810699	418.306152	1815.384399
10.820001	2318.830078	10.830699	570.591614	1811.344849
10.840000	2284.330078	10.850698	477.990845	1807.319946
10.860001	2138.670166	10.870699	208.927979	1803.312134
10.880000	1949.669922	10.890697	57.386978	1799.318481
10.900000	1858.830078	10.910697	13.611397	1795.339722
10.920000	1848.500000	10.930697	11.490376	1791.377563
10.940000	1806.330078	10.950697	16.135181	1787.429077
10.960000	1800.330078	10.970697	17.357635	1783.495728
10.980000	1814.169922	10.990697	15.457729	1779.578003
11.000000	1780.000000	11.010697	12.588259	1775.674683
11.020000	1786.669922	11.030698	9.971284	1771.785522
11.040000	1785.830078	11.050697	7.922654	1767.911011
11.060000	1758.669922	11.070697	6.383876	1764.051880
11.080000	1754.830078	11.090696	5.225480	1760.206177
11.100000	1747.830078	11.110697	4.339502	1756.375122
11.120001	1733.830078	11.130697	3.649821	1752.557983
11.140000	1756.669922	11.150697	3.104137	1748.755493
11.160001	1734.000000	11.170697	2.666042	1744.966675
11.180000	1750.330078	11.190697	2.309808	1741.191528

11.200000	1753.500000	11.210696	2.016786	1737.430786
11.220000	1702.669922	11.230697	1.773263	1733.683472
11.240000	1763.169922	11.250696	1.569014	1729.949585
11.260000	1727.330078	11.270697	1.396246	1726.229614
11.280000	1735.500000	11.290696	1.249004	1722.522827
11.300000	1747.669922	11.310697	1.122626	1718.829956
11.320001	1711.330078	11.330697	1.013465	1715.150513
11.340000	1697.500000	11.350696	0.918629	1711.483032
11.360001	1691.669922	11.370696	0.835773	1707.830688
11.380000	1707.330078	11.390696	0.763030	1704.189575
11.400000	1685.500000	11.410695	0.698862	1700.562134
11.420000	1699.330078	11.430696	0.642011	1696.947388
11.440000	1696.169922	11.450695	0.591441	1693.346802
11.460000	1709.669922	11.470695	0.546283	1689.757690
11.480000	1672.000000	11.490695	0.505819	1686.181519
11.500000	1692.000000	11.510695	0.469435	1682.617554
11.520000	1679.500000	11.530696	0.436618	1679.066772
11.540000	1682.500000	11.550695	0.406933	1675.527710
11.560000	1661.669922	11.570696	0.380002	1672.001587
11.580000	1700.830078	11.590695	0.355507	1668.487183
11.600000	1656.500000	11.610696	0.333171	1664.985229
11.620001	1667.169922	11.630696	0.312755	1661.495728
11.640000	1676.830078	11.650695	0.294053	1658.017700
11.660001	1671.669922	11.670695	0.276882	1654.551880
11.680000	1625.330078	11.690695	0.261087	1651.098511
11.700000	1651.500000	11.710694	0.246527	1647.656372
11.720000	1628.000000	11.730695	0.233081	1644.225464
11.740000	1626.000000	11.750694	0.220642	1640.807495
11.760000	1644.669922	11.770695	0.209115	1637.400269
11.780000	1658.169922	11.790694	0.198416	1634.004761
11.800000	1672.169922	11.810695	0.188469	1630.620728
11.820001	1610.000000	11.830695	0.179208	1627.247437
11.840000	1624.330078	11.850695	0.170574	1623.886353
11.860001	1602.830078	11.870695	0.162512	1620.536499
11.880000	1612.000000	11.890695	0.154975	1617.197388
11.900000	1630.330078	11.910694	0.147920	1613.869995
11.920000	1642.169922	11.930694	0.141307	1610.552856
11.940000	1627.000000	11.950693	0.135102	1607.246948

11.960000	1601.330078	11.970694	0.129272	1603.952271
11.980000	1618.669922	11.990693	0.123790	1600.668335
12.000000	1610.500000	12.010694	0.118628	1597.394897
12.020000	1614.169922	12.030694	0.113764	1594.132446
12.040000	1618.830078	12.050694	0.109175	1590.880493
12.060000	1614.500000	12.070694	0.104841	1587.638794
12.080000	1631.330078	12.090693	0.100746	1584.407837
12.100000	1596.500000	12.110694	0.096871	1581.187378
12.120001	1588.330078	12.130694	0.093202	1577.977661
12.140000	1585.330078	12.150694	0.089725	1574.778442
12.160001	1575.000000	12.170694	0.086428	1571.588745
12.180000	1562.330078	12.190694	0.083299	1568.409058
12.200000	1547.500000	12.210692	0.080326	1565.240601
12.220000	1559.830078	12.230693	0.077500	1562.081909
12.240000	1550.830078	12.250692	0.074811	1558.932495
12.260000	1555.000000	12.270693	0.072252	1555.793335
12.280000	1533.830078	12.290692	0.069814	1552.664673
12.300000	1529.169922	12.310693	0.067490	1549.545288
12.320001	1532.830078	12.330693	0.000000	1546.435425
12.340000	1537.169922	12.350693	0.000000	1543.336304
12.360001	1525.669922	12.370693	0.000000	1540.246704
12.380000	1523.830078	12.390693	0.000000	1537.165894
12.400000	1536.669922	12.410692	0.000000	1534.095581
12.420000	1498.169922	12.430693	0.000000	1531.034302
12.440000	1526.500000	12.450692	0.000000	1527.982056
12.460000	1548.500000	12.470693	0.000000	1524.939575
12.480000	1520.000000	12.490691	0.000000	1521.906860
12.500000	1514.330078	12.510692	0.000000	1518.883423
12.520000	1506.500000	12.530692	0.000000	1515.868774
12.540000	1509.000000	12.550692	0.000000	1512.863159
12.560000	1517.669922	12.570692	0.000000	1509.867310
12.580000	1498.830078	12.590692	0.000000	1506.880737
12.600000	1503.000000	12.610692	0.000000	1503.901978
12.620001	1493.830078	12.630692	0.000000	1500.933472
12.640000	1500.830078	12.650692	0.000000	1497.972778
12.660001	1490.000000	12.670692	0.000000	1495.022339
12.680000	1494.669922	12.690692	0.000000	1492.079712
12.700000	1495.330078	12.710691	0.000000	1489.146362

12.720000	1452.830078	12.730692	0.000000	1486.221802
12.740000	1463.330078	12.750691	0.000000	1483.306030
12.760000	1440.169922	12.770692	0.000000	1480.398315
12.780000	1464.669922	12.790690	0.000000	1477.499878
12.800000	1460.330078	12.810691	0.000000	1474.609497
12.820001	1499.830078	12.830691	0.000000	1471.728149
12.840000	1430.330078	12.850691	0.000000	1468.854370
12.860001	1464.500000	12.870691	0.000000	1465.989868
12.880000	1467.169922	12.890691	0.000000	1463.133667
12.900000	1467.830078	12.910690	0.000000	1460.285522
12.920000	1459.500000	12.930691	0.000000	1457.446167
12.940000	1460.500000	12.950690	0.000000	1454.614624
12.960000	1446.830078	12.970691	0.000000	1451.791626
12.980000	1417.830078	12.990690	0.000000	1448.976196
13.000000	1448.830078	13.010691	0.000000	1446.169067
13.020000	1447.000000	13.030691	0.000000	1443.369751
13.040000	1426.500000	13.050691	0.000000	1440.579712
13.060000	1437.500000	13.070690	0.000000	1437.796509
13.080000	1407.830078	13.090690	0.000000	1435.022339
13.100000	1448.500000	13.110690	0.000000	1432.255249
13.120001	1410.169922	13.130691	0.000000	1429.495972
13.140000	1392.669922	13.150690	0.000000	1426.744751
13.160001	1429.669922	13.170691	0.000000	1424.001099
13.180000	1411.669922	13.190690	0.000000	1421.265991
13.200000	1392.830078	13.210690	0.000000	1418.537964
13.220000	1430.000000	13.230690	0.000000	1415.817749
13.240000	1414.830078	13.250690	0.000000	1413.105347
13.260000	1380.169922	13.270690	0.000000	1410.400513
13.280000	1412.169922	13.290689	0.000000	1407.703247
13.300000	1381.169922	13.310690	0.000000	1405.013062
13.320001	1396.830078	13.330690	0.000000	1402.331177
13.340000	1410.330078	13.350689	0.000000	1399.656860
13.360001	1386.330078	13.370689	0.000000	1396.989380
13.380000	1392.330078	13.390689	0.000000	1394.329712
13.400000	1368.669922	13.410688	0.000000	1391.676880
13.420000	1389.330078	13.430689	0.000000	1389.031372
13.440000	1373.830078	13.450688	0.000000	1386.393921
13.460000	1356.500000	13.470689	0.000000	1383.762817

13.480000	1383.669922	13.490688	0.000000	1381.139771
13.500000	1388.500000	13.510689	0.000000	1378.523560
13.520000	1381.830078	13.530689	0.000000	1375.914673
13.540000	1383.500000	13.550689	0.000000	1373.313110
13.560000	1379.169922	13.570689	0.000000	1370.718628
13.580000	1390.330078	13.590689	0.000000	1368.130737
13.600000	1375.669922	13.610689	0.000000	1365.549927
13.620001	1418.669922	13.630690	0.000000	1362.975952
13.640000	1388.330078	13.650688	0.000000	1360.409790
13.660001	1378.500000	13.670689	0.000000	1357.850220
13.680000	1353.169922	13.690688	0.000000	1355.297241
13.700000	1352.830078	13.710688	0.000000	1352.751831
13.720000	1332.000000	13.730688	0.000000	1350.212769
13.740000	1359.330078	13.750688	0.000000	1347.680298
13.760000	1329.669922	13.770688	0.000000	1345.155640
13.780000	1332.669922	13.790688	0.000000	1342.636841
13.800000	1316.500000	13.810688	0.000000	1340.124878
13.820001	1328.830078	13.830688	0.000000	1337.619995
13.840000	1327.000000	13.850688	0.000000	1335.121460
13.860001	1326.330078	13.870688	0.000000	1332.630249
13.880000	1299.669922	13.890688	0.000000	1330.145142
13.900000	1316.330078	13.910687	0.000000	1327.666626
13.920000	1321.000000	13.930687	0.000000	1325.195435
13.940000	1299.000000	13.950686	0.000000	1322.729858
13.960000	1328.330078	13.970687	0.000000	1320.271362
13.980000	1307.669922	13.990686	0.000000	1317.819458
14.000000	1321.669922	14.010687	0.000000	1315.373657
14.020000	1333.000000	14.030687	0.000000	1312.934448
14.040000	1309.669922	14.050687	0.000000	1310.501343
14.060000	1311.669922	14.070687	0.000000	1308.074829
14.080000	1320.830078	14.090687	0.000000	1305.655640
14.100000	1298.000000	14.110687	0.000000	1303.241577
14.120001	1316.500000	14.130688	0.000000	1300.834595
14.140000	1321.169922	14.150687	0.000000	1298.433472
14.160001	1310.669922	14.170688	0.000000	1296.038940
14.180000	1319.830078	14.190687	0.000000	1293.651001
14.200000	1309.500000	14.210686	0.000000	1291.269165
14.220000	1290.330078	14.230686	0.000000	1288.893188

14.240000	1279.000000	14.250686	0.000000	1286.523315
14.260000	1296.169922	14.270686	0.000000	1284.160034
14.280000	1267.500000	14.290686	0.000000	1281.802368
14.300000	1276.330078	14.310686	0.000000	1279.451782
14.320001	1270.830078	14.330687	0.000000	1277.106812
14.340000	1256.330078	14.350686	0.000000	1274.767700
14.360001	1275.830078	14.370687	0.000000	1272.434692
14.380000	1253.669922	14.390686	0.000000	1270.108521
14.400000	1270.669922	14.410686	0.000000	1267.787720
14.420000	1272.830078	14.430686	0.000000	1265.472778
14.440000	1262.330078	14.450686	0.000000	1263.164185
14.460000	1253.169922	14.470686	0.000000	1260.861938
14.480000	1225.169922	14.490685	0.000000	1258.564819
14.500000	1253.830078	14.510685	0.000000	1256.274048
14.520000	1228.000000	14.530685	0.000000	1253.989380
14.540000	1248.669922	14.550685	0.000000	1251.710327
14.560000	1242.669922	14.570685	0.000000	1249.437134
14.580000	1230.830078	14.590685	0.000000	1247.170288
14.600000	1242.669922	14.610685	0.000000	1244.908325
14.620001	1241.500000	14.630686	0.000000	1242.653198
14.640000	1229.830078	14.650685	0.000000	1240.403442
14.660001	1242.830078	14.670686	0.000000	1238.159546
14.680000	1216.169922	14.690685	0.000000	1235.921509
14.700000	1235.830078	14.710685	0.000000	1233.689087
14.720000	1226.330078	14.730685	0.000000	1231.462280
14.740000	1200.000000	14.750685	0.000000	1229.241821
14.760000	1202.169922	14.770685	0.000000	1227.026001
14.780000	1223.830078	14.790684	0.000000	1224.817017
14.800000	1218.169922	14.810684	0.000000	1222.613159
14.820001	1208.000000	14.830685	0.000000	1220.414673
14.840000	1207.500000	14.850684	0.000000	1218.222046
14.860001	1187.330078	14.870685	0.000000	1216.035278
14.880000	1201.669922	14.890684	0.000000	1213.853882
14.900000	1215.500000	14.910684	0.000000	1211.678345
14.920000	1202.669922	14.930684	0.000000	1209.508179
14.940000	1181.330078	14.950684	0.000000	1207.343384
14.960000	1219.500000	14.970684	0.000000	1205.184692
14.980000	1196.830078	14.990684	0.000000	1203.031128

15.000000	1201.669922	15.010684	0.000000	1200.883179
15.020000	1218.669922	15.030684	0.000000	1198.740112
15.040000	1173.330078	15.050684	0.000000	1196.603149
15.060000	1184.000000	15.070683	0.000000	1194.472290
15.080000	1194.330078	15.090683	0.000000	1192.345825
15.100000	1200.830078	15.110683	0.000000	1190.225220
15.120001	1210.169922	15.130684	0.000000	1188.110229
15.140000	1202.500000	15.150683	0.000000	1186.000122
15.160001	1200.669922	15.170684	0.000000	1183.895874
15.180000	1176.500000	15.190683	0.000000	1181.796753
15.200000	1160.330078	15.210683	0.000000	1179.703491
15.220000	1161.000000	15.230683	0.000000	1177.615112
15.240000	1160.830078	15.250683	0.000000	1175.532349
15.260000	1150.000000	15.270683	0.000000	1173.454224
15.280000	1170.830078	15.290683	0.000000	1171.382202
15.300000	1173.830078	15.310683	0.000000	1169.315552
15.320001	1181.669922	15.330684	0.000000	1167.253540
15.340000	1186.000000	15.350683	0.000000	1165.196899
15.360001	1171.500000	15.370683	0.450599	1163.146362
15.380000	1146.669922	15.390682	0.459841	1161.100220
15.400000	1139.169922	15.410682	0.469405	1159.059937
15.420000	1157.330078	15.430682	0.479308	1157.024536
15.440000	1133.000000	15.450682	0.489564	1154.994263
15.460000	1139.330078	15.470682	0.500194	1152.969116
15.480000	1135.500000	15.490682	0.511216	1150.949585
15.500000	1155.000000	15.510682	0.522651	1148.934692
15.520000	1154.500000	15.530683	0.534520	1146.925659
15.540000	1120.169922	15.550682	0.546847	1144.921021
15.560000	1144.169922	15.570683	0.559658	1142.921997
15.580000	1146.669922	15.590682	0.572979	1140.928101
15.600000	1158.669922	15.610682	0.586840	1138.939087
15.620001	1144.169922	15.630683	0.601272	1136.955200
15.640000	1109.500000	15.650681	0.616306	1134.976685
15.660001	1113.000000	15.670682	0.631981	1133.003296
15.680000	1115.830078	15.690681	0.648335	1131.034790
15.700000	1129.830078	15.710681	0.665410	1129.071167
15.720000	1120.000000	15.730681	0.683252	1127.112915
15.740000	1108.669922	15.750681	0.701909	1125.159546

15.760000	1136.000000	15.770681	0.721435	1123.211060
15.780000	1099.169922	15.790681	0.741887	1121.267944
15.800000	1117.000000	15.810681	0.763329	1119.329468
15.820001	1125.169922	15.830682	0.785829	1117.396362
15.840000	1110.830078	15.850681	0.809460	1115.468140
15.860001	1096.830078	15.870682	0.834307	1113.544800
15.880000	1093.830078	15.890681	0.860456	1111.626587
15.900000	1076.330078	15.910681	0.888005	1109.713013
15.920000	1105.669922	15.930681	0.917066	1107.804565
15.940000	1069.500000	15.950680	0.947750	1105.901489
15.960000	1085.500000	15.970680	0.980196	1104.002563
15.980000	1102.000000	15.990680	1.014543	1102.108765
16.000000	1110.669922	16.010681	1.050956	1100.220093
16.020000	1101.669922	16.030682	1.089609	1098.336060
16.040001	1085.330078	16.050682	1.130700	1096.457153
16.059999	1062.830078	16.070681	1.174449	1094.583374
16.080000	1080.169922	16.090679	1.221105	1092.714233
16.100000	1083.169922	16.110680	1.270953	1090.849976
16.120001	1091.830078	16.130680	1.324302	1088.990112
16.140001	1088.000000	16.150681	1.381507	1087.135376
16.160000	1112.330078	16.170679	1.442964	1085.285522
16.180000	1080.669922	16.190680	1.509142	1083.440552
16.200001	1083.169922	16.210680	1.580560	1081.600464
16.220001	1077.830078	16.230680	1.657817	1079.764771
16.240002	1068.330078	16.250681	1.741607	1077.933960
16.260000	1092.669922	16.270679	1.832720	1076.108276
16.280001	1054.830078	16.290680	1.932106	1074.287476
16.299999	1061.500000	16.310678	2.040836	1072.470581
16.320000	1068.500000	16.330679	2.160212	1070.659302
16.340000	1072.830078	16.350679	2.291742	1068.852417
16.359999	1073.330078	16.370678	2.437219	1067.050415
16.379999	1063.000000	16.390678	2.598837	1065.253052
16.400000	1067.000000	16.410679	2.779205	1063.460327
16.420000	1065.330078	16.430679	2.981515	1061.672241
16.440001	1049.669922	16.450680	3.209698	1059.888794
16.459999	1053.500000	16.470678	3.468605	1058.110474
16.480000	1057.169922	16.490679	3.764417	1056.336548
16.500000	1037.500000	16.510679	4.104902	1054.567261

16.520000	1063.169922	16.530680	4.500103	1052.802368
16.540001	1034.830078	16.550680	4.963161	1051.042603
16.559999	1028.000000	16.570679	5.511564	1049.287231
16.580000	1025.330078	16.590679	6.169637	1047.536743
16.600000	1035.830078	16.610680	6.973226	1045.790649
16.620001	1062.669922	16.630680	7.983336	1044.048950
16.640001	1035.830078	16.650681	9.322667	1042.312134
16.660000	1031.500000	16.670677	11.258163	1040.580444
16.680000	1052.669922	16.690678	14.333151	1038.852173
16.700001	1068.500000	16.710678	19.443331	1037.129517
16.720001	1066.500000	16.730679	27.630877	1035.410767
16.740002	1054.000000	16.750679	39.508602	1033.696899
16.760000	1077.669922	16.770678	55.023075	1031.987671
16.780001	1125.000000	16.790678	75.246132	1030.282593
16.799999	1147.000000	16.810677	106.773659	1028.582153
16.820000	1179.669922	16.830677	152.242081	1026.886841
16.840000	1186.000000	16.850677	139.450195	1025.194946
16.859999	1120.000000	16.870676	74.701759	1023.508667
16.879999	1071.500000	16.890676	35.451214	1021.826294
16.900000	1056.830078	16.910677	18.590765	1020.148315
16.920000	1067.500000	16.930677	13.277893	1018.475220
16.940001	1059.330078	16.950678	12.428895	1016.806030
16.959999	1064.669922	16.970676	12.264639	1015.141968
16.980000	1071.330078	16.990677	11.559698	1013.482300
17.000000	1053.500000	17.010677	10.390894	1011.826294
17.020000	1034.669922	17.030678	9.117343	1010.175415
17.040001	1025.500000	17.050678	7.962587	1008.528931
17.059999	1023.000000	17.070677	6.991564	1006.886841
17.080000	1011.169983	17.090677	6.191727	1005.249146
17.100000	1006.000000	17.110678	5.531072	1003.615356
17.120001	1005.830017	17.130678	4.979660	1001.986450
17.140001	1001.669983	17.150679	4.514133	1000.361694
17.160000	986.666992	17.170677	4.116972	998.741577
17.180000	988.333008	17.190678	3.774860	997.126099
17.200001	998.333008	17.210678	3.477713	995.514282
17.220001	1009.500000	17.230679	3.217674	993.907349
17.240002	989.666992	17.250677	2.988587	992.304810
17.260000	983.833008	17.270676	2.785511	990.706177

17.280001	1007.000000	17.290676	2.604474	989.111938
17.299999	1007.000000	17.310675	2.442296	987.522339
17.320000	993.333008	17.330675	2.296309	985.936646
17.340000	978.666992	17.350676	2.375324	984.355347
17.359999	975.166992	17.370674	2.260150	982.778687
17.379999	989.833008	17.390675	2.155810	981.205933
17.400000	975.333008	17.410675	2.061037	979.637573
17.420000	979.166992	17.430676	1.974742	978.073364
17.440001	945.166992	17.450676	1.895998	976.513550
17.459999	960.500000	17.470675	1.824018	974.958130
17.480000	979.000000	17.490675	1.758103	973.406860
17.500000	978.833008	17.510675	1.697668	971.859497
17.520000	961.000000	17.530676	1.642200	970.316528
17.540001	955.333008	17.550676	1.591252	968.777954
17.559999	966.000000	17.570675	1.544438	967.243530
17.580000	943.333008	17.590675	1.501409	965.713257
17.600000	971.666992	17.610676	1.461871	964.186890
17.620001	962.333008	17.630676	1.425561	962.665405
17.640001	961.500000	17.650677	1.392249	961.147095
17.660000	959.833008	17.670675	1.361737	959.633911
17.680000	970.333008	17.690676	1.333841	958.124634
17.700001	960.333008	17.710676	1.308410	956.618774
17.720001	921.666992	17.730677	1.285309	955.117798
17.740002	948.500000	17.750677	1.264420	953.620728
17.760000	962.666992	17.770676	1.245646	952.127808
17.780001	946.833008	17.790676	1.228896	950.639038
17.799999	935.500000	17.810675	1.214101	949.154175
17.820000	938.166992	17.830673	1.201202	947.673706
17.840000	947.833008	17.850674	1.190151	946.197144
17.859999	937.333008	17.870672	1.180916	944.724731
17.879999	933.833008	17.890673	1.173471	943.256226
17.900000	927.833008	17.910673	1.167806	941.791626
17.920000	936.500000	17.930674	1.163921	940.331421
17.940001	953.333008	17.950674	1.161828	938.874878
17.959999	941.833008	17.970673	1.161549	937.422974
17.980000	936.166992	17.990673	1.163121	935.974487
18.000000	927.500000	18.010674	1.166593	934.530151
18.020000	934.000000	18.030674	1.172029	933.089966

18.040001	938.333008	18.050674	1.179507	931.653442
18.059999	915.833008	18.070673	1.189123	930.221069
18.080000	925.166992	18.090673	1.200994	928.792847
18.100000	924.666992	18.110674	1.215255	927.368286
18.120001	926.500000	18.130674	1.232067	925.947876
18.140001	920.666992	18.150675	1.251618	924.531372
18.160000	935.333008	18.170673	1.274125	923.119019
18.180000	915.166992	18.190674	1.299851	921.710327
18.200001	921.833008	18.210674	1.329091	920.305542
18.220001	925.333008	18.230675	1.362198	918.904663
18.240002	917.500000	18.250675	1.399581	917.507690
18.260000	912.833008	18.270674	1.441718	916.114624
18.280001	903.000000	18.290674	1.489184	914.725464
18.299999	919.500000	18.310673	1.542643	913.340454
18.320000	918.333008	18.330673	1.160952	911.958862
18.340000	904.500000	18.350674	1.237620	910.581177
18.359999	914.000000	18.370672	1.322901	909.207642
18.379999	916.000000	18.390673	1.418217	907.837524
18.400000	913.333008	18.410673	1.525273	906.471313
18.420000	915.666992	18.430672	1.646158	905.109131
18.440001	911.166992	18.450672	1.783503	903.750610
18.459999	900.833008	18.470671	1.940554	902.395874
18.480000	908.166992	18.490671	2.121489	901.044800
18.500000	919.166992	18.510672	2.331626	899.697632
18.520000	904.833008	18.530672	2.577920	898.354248
18.540001	898.833008	18.550673	2.869598	897.014648
18.559999	891.500000	18.570671	3.219203	895.678589
18.580000	907.333008	18.590672	3.644809	894.346313
18.600000	894.666992	18.610672	4.174846	893.017944
18.620001	906.833008	18.630672	4.861348	891.692993
18.640001	912.833008	18.650673	5.811022	890.371826
18.660000	882.166992	18.670671	7.241842	889.054565
18.680000	910.333008	18.690672	9.545174	887.740967
18.700001	888.500000	18.710672	13.271458	886.430786
18.720001	906.333008	18.730673	18.955231	885.124512
18.740002	935.666992	18.750673	26.909830	883.821533
18.760000	920.500000	18.770672	37.586887	882.522827
18.780001	944.833008	18.790672	53.262810	881.227417

18.799999	968.000000	18.810671	78.749809	879.935547
18.820000	976.166992	18.830671	97.649673	878.647339
18.840000	978.000000	18.850672	67.904808	877.362671
18.859999	930.500000	18.870670	34.530083	876.081665
18.879999	896.666992	18.890671	17.532398	874.804443
18.900000	900.500000	18.910671	10.568363	873.530518
18.920000	867.333008	18.930672	8.293841	872.260010
18.940001	881.000000	18.950672	7.604700	870.993164
18.959999	869.500000	18.970671	7.085944	869.730103
18.980000	877.000000	18.990671	6.389359	868.470337
19.000000	891.333008	19.010670	5.601189	867.214478
19.020000	872.000000	19.030670	4.855483	865.961426
19.040001	863.000000	19.050671	4.216058	864.712524
19.059999	872.000000	19.070669	3.689084	863.466675
19.080000	870.833008	19.090670	3.257622	862.224487
19.100000	863.500000	19.110670	2.901794	860.985840
19.120001	852.166992	19.130671	2.604999	859.750488
19.140001	875.833008	19.150671	2.354615	858.518799
19.160000	878.833008	19.170670	2.141209	857.290527
19.180000	864.166992	19.190670	1.957600	856.065186
19.200001	851.500000	19.210670	1.798338	854.843750
19.220001	850.833008	19.230671	1.659171	853.625610
19.240002	865.666992	19.250671	1.536749	852.411011
19.260000	850.500000	19.270670	1.428416	851.199707
19.280001	845.000000	19.290670	1.332002	849.991333
19.299999	852.000000	19.310669	1.245782	848.786865
19.320000	855.166992	19.330669	1.168304	847.585815
19.340000	852.166992	19.350670	1.098393	846.387695
19.359999	850.500000	19.370668	1.035069	845.192993
19.379999	871.333008	19.390669	0.977489	844.001709
19.400000	843.666992	19.410669	0.924962	842.813599
19.420000	848.166992	19.430670	0.876891	841.629150
19.440001	852.000000	19.450670	0.832771	840.447632
19.459999	843.333008	19.470669	0.792167	839.269531
19.480000	845.500000	19.490669	0.754696	838.094727
19.500000	850.500000	19.510670	0.720036	836.922974
19.520000	844.666992	19.530670	0.687903	835.754761
19.540001	848.333008	19.550671	0.658047	834.589355

19.559999	858.000000	19.570669	0.630254	833.427490
19.580000	842.833008	19.590670	0.604326	832.269043
19.600000	834.000000	19.610668	0.580099	831.113281
19.620001	835.333008	19.630669	0.557417	829.961060
19.640001	827.833008	19.650669	0.536148	828.812134
19.660000	822.166992	19.670668	0.516177	827.666016
19.680000	826.500000	19.690668	0.497390	826.523071
19.700001	840.166992	19.710669	0.479696	825.383545
19.720001	841.500000	19.730669	0.463007	824.246948
19.740002	826.833008	19.750669	0.447247	823.113525
19.760000	804.666992	19.770668	0.432346	821.983154
19.780001	834.000000	19.790668	0.418238	820.855957
19.799999	804.000000	19.810667	0.404869	819.731934
19.820000	834.166992	19.830667	0.392182	818.610718
19.840000	831.500000	19.850668	0.380133	817.492920
19.859999	826.500000	19.870667	0.368678	816.378052
19.879999	828.166992	19.890667	0.357775	815.265991
19.900000	829.000000	19.910667	0.347390	814.157104
19.920000	825.833008	19.930668	0.337489	813.051392
19.940001	817.500000	19.950668	0.328040	811.948486
19.959999	806.000000	19.970665	0.319018	810.848999
19.980000	810.333008	19.990665	0.310392	809.752197
20.000000	802.666992	20.010666	0.302142	808.658203
20.020000	829.333008	20.030666	0.294244	807.567139
20.040001	803.500000	20.050667	0.286678	806.479126
20.059999	808.166992	20.070665	0.279425	805.394409
20.080000	810.500000	20.090666	0.272467	804.312256
20.100000	819.166992	20.110666	0.265787	803.233032
20.120001	828.833008	20.130667	0.607238	802.156738
20.140001	822.333008	20.150667	0.608769	801.083496
20.160000	795.333008	20.170666	0.610804	800.013062
20.180000	799.333008	20.190664	0.613345	798.945435
20.200001	813.000000	20.210665	0.616393	797.880859
20.220001	782.000000	20.230665	0.619952	796.818970
20.240002	811.000000	20.250666	0.624027	795.759766
20.260000	785.000000	20.270664	0.628625	794.703735
20.280001	791.166992	20.290665	0.633756	793.650024
20.299999	807.666992	20.310663	0.639428	792.599731

20.320000	803.666992	20.330664	0.438653	791.552124
20.340000	792.166992	20.350664	0.449726	790.506592
20.359999	782.166992	20.370663	0.461238	789.464478
20.379999	796.333008	20.390663	0.473218	788.425293
20.400000	785.500000	20.410664	0.485689	787.388428
20.420000	791.166992	20.430664	0.498680	786.354492
20.440001	794.166992	20.450665	0.512220	785.323120
20.459999	794.500000	20.470663	0.526340	784.294556
20.480000	781.166992	20.490664	0.541077	783.268799
20.500000	796.333008	20.510664	0.556467	782.245361
20.520000	784.500000	20.530664	0.572550	781.225098
20.540001	781.000000	20.550665	0.589369	780.207275
20.559999	789.166992	20.570663	0.606968	779.191895
20.580000	766.666992	20.590664	0.625401	778.179443
20.600000	776.666992	20.610664	0.644721	777.169434
20.620001	778.666992	20.630665	0.664986	776.162354
20.640001	793.833008	20.650665	0.686262	775.157471
20.660000	775.333008	20.670664	0.708614	774.155396
20.680000	775.666992	20.690664	0.732124	773.155884
20.700001	773.333008	20.710665	0.756871	772.159058
20.720001	775.666992	20.730665	0.782946	771.164673
20.740002	779.500000	20.750666	0.810447	770.172607
20.760000	767.833008	20.770664	0.839479	769.183228
20.780001	777.000000	20.790663	0.870164	768.196655
20.799999	760.333008	20.810661	0.902632	767.212769
20.820000	789.333008	20.830662	0.937029	766.230835
20.840000	790.000000	20.850662	0.973512	765.251587
20.859999	784.500000	20.870661	1.012252	764.274780
20.879999	760.000000	20.890661	1.053450	763.300537
20.900000	760.666992	20.910662	1.097320	762.328491
20.920000	761.166992	20.930662	1.144101	761.359131
20.940001	754.666992	20.950663	1.194063	760.392212
20.959999	780.500000	20.970661	1.247502	759.427734
20.980000	750.833008	20.990662	1.304764	758.465454
21.000000	754.833008	21.010662	1.366225	757.505859
21.020000	760.333008	21.030663	1.432314	756.548340
21.040001	770.666992	21.050663	1.503517	755.593384
21.059999	746.666992	21.070662	1.580379	754.640869

21.080000	777.166992	21.090662	1.663544	753.690674
21.100000	759.000000	21.110662	1.753726	752.742554
21.120001	771.500000	21.130663	1.851753	751.796997
21.140001	756.500000	21.150663	1.958581	750.853394
21.160000	746.833008	21.170662	2.075305	749.912720
21.180000	746.666992	21.190662	2.203235	748.973999
21.200001	762.833008	21.210663	2.343871	748.037476
21.220001	746.833008	21.230663	2.708050	747.103394
21.240002	753.833008	21.250664	2.884642	746.171265
21.260000	730.166992	21.270662	3.080467	745.241699
21.280001	731.666992	21.290663	3.298561	744.314453
21.299999	753.500000	21.310661	3.542500	743.389160
21.320000	754.500000	21.330662	3.816727	742.466187
21.340000	731.833008	21.350662	4.126604	741.545532
21.359999	741.666992	21.370661	4.478752	740.626709
21.379999	750.000000	21.390659	4.881504	739.710571
21.400000	751.000000	21.410660	5.345387	738.796265
21.420000	762.166992	21.430660	5.883745	737.884155
21.440001	754.666992	21.450661	6.513881	736.973877
21.459999	748.166992	21.470659	7.258424	736.066406
21.480000	739.500000	21.490660	8.147843	735.160767
21.500000	752.000000	21.510660	9.223594	734.257080
21.520000	739.500000	21.530661	10.544977	733.355347
21.540001	752.666992	21.550661	12.203507	732.456055
21.559999	743.833008	21.570660	14.357158	731.558716
21.580000	741.000000	21.590660	17.308815	730.663330
21.600000	742.666992	21.610661	21.648489	729.770020
21.620001	735.500000	21.630661	28.437563	728.878906
21.640001	749.000000	21.650661	39.294708	727.989624
21.660000	786.000000	21.670660	56.200817	727.102417
21.680000	801.000000	21.690660	81.213844	726.217285
21.700001	829.333008	21.710661	117.214424	725.333984
21.720001	880.500000	21.730661	171.563126	724.452759
21.740002	998.500000	21.750662	258.803223	723.573608
21.760000	1056.500000	21.770660	352.751343	722.696289
21.780001	1037.500000	21.790661	302.397186	721.821045
21.799999	928.000000	21.810659	170.450226	720.947632
21.820000	858.833008	21.830660	88.546654	720.076172

21.840000	799.666992	21.850660	50.666756	719.206543
21.859999	758.500000	21.870659	35.028912	718.338867
21.879999	754.166992	21.890659	28.612312	717.473022
21.900000	735.500000	21.910660	25.033356	716.609131
21.920000	710.666992	21.930660	21.949839	715.747070
21.940001	731.000000	21.950661	18.966637	714.886963
21.959999	714.500000	21.970659	16.256376	714.028564
21.980000	726.000000	21.990658	13.963552	713.171997
22.000000	719.333008	22.010658	12.100255	712.317505
22.020000	703.666992	22.030659	10.605281	711.464600
22.040001	716.833008	22.050659	9.402267	710.613403
22.059999	720.166992	22.070658	8.425603	709.764160
22.080000	710.333008	22.090658	7.624826	708.916626
22.100000	726.833008	22.110659	6.962766	708.071045
22.120001	722.666992	22.130659	6.411771	707.226685
22.140001	709.666992	22.150660	5.951114	706.384521
22.160000	717.500000	22.170658	5.565103	705.544189
22.180000	730.166992	22.190659	5.241624	704.705322
22.200001	731.833008	22.210659	4.971432	703.868286
22.220001	715.500000	22.230659	4.747349	703.032837
22.240002	739.333008	22.250660	4.563846	702.198853
22.260000	726.000000	22.270658	4.416718	701.367065
22.280001	722.500000	22.290659	4.302796	700.536499
22.299999	709.166992	22.310658	4.219880	699.707886
22.320000	707.666992	22.330658	4.166531	698.880859
22.340000	701.333008	22.350658	4.142081	698.055664
22.359999	690.666992	22.370657	4.146565	697.231567
22.379999	695.833008	22.390657	4.180755	696.409302
22.400000	694.000000	22.410658	4.246209	695.588745
22.420000	700.000000	22.430658	4.345376	694.769775
22.440001	698.000000	22.450659	4.481750	693.952271
22.459999	688.000000	22.470657	4.660089	693.136719
22.480000	721.000000	22.490658	4.886822	692.322388
22.500000	691.666992	22.510658	5.170424	691.509521
22.520000	683.500000	22.530659	5.522169	690.698120
22.540001	701.333008	22.550659	5.957113	689.888550
22.559999	713.666992	22.570658	6.495528	689.080078
22.580000	705.833008	22.590656	7.165408	688.273682

22.600000	695.666992	22.610657	8.006598	687.468506
22.620001	686.166992	22.630657	9.079221	686.664917
22.640001	702.500000	22.650658	10.483638	685.862549
22.660000	705.500000	22.670656	12.404767	685.061646
22.680000	728.000000	22.690657	15.199270	684.262451
22.700001	734.333008	22.710657	19.520472	683.464600
22.720001	752.666992	22.730658	26.422787	682.668091
22.740002	760.000000	22.750658	37.330948	681.873169
22.760000	750.833008	22.770657	53.897980	681.079590
22.780001	777.500000	22.790657	78.303131	680.287231
22.799999	826.333008	22.810656	115.121201	679.496460
22.820000	883.333008	22.830656	174.141159	678.707031
22.840000	957.166992	22.850657	252.679886	677.918823
22.859999	968.333008	22.870655	258.654114	677.132202
22.879999	915.166992	22.890656	162.986755	676.346924
22.900000	817.666992	22.910656	85.942978	675.562622
22.920000	761.666992	22.930656	47.408665	674.780029
22.940001	754.666992	22.950657	30.483587	673.998657
22.959999	723.833008	22.970655	23.256674	673.218506
22.980000	710.500000	22.990656	19.586550	672.439819
23.000000	705.500000	23.010656	16.900202	671.662109
23.020000	680.833008	23.030657	14.481216	670.885986
23.040001	690.666992	23.050657	12.305515	670.110962
23.059999	667.333008	23.070656	10.454246	669.337524
23.080000	687.000000	23.090656	8.941762	668.565063
23.100000	665.000000	23.110657	7.725743	667.793701
23.120001	669.000000	23.130657	6.746493	667.023926
23.140001	672.666992	23.150658	5.949984	666.255249
23.160000	670.833008	23.170656	5.294081	665.487549
23.180000	637.833008	23.190655	4.747314	664.721191
23.200001	663.500000	23.210655	4.286406	663.956055
23.220001	655.500000	23.230656	3.894037	663.192261
23.240002	669.166992	23.250656	3.557020	662.429321
23.260000	660.666992	23.270655	3.265239	661.667603
23.280001	657.000000	23.290655	3.010727	660.907104
23.299999	659.833008	23.310654	2.787296	660.147827
23.320000	668.000000	23.330654	2.589921	659.389771
23.340000	679.833008	23.350655	2.414614	658.632446

23.359999	682.333008	23.370653	2.258128	657.876709
23.379999	670.500000	23.390654	2.117757	657.121826
23.400000	650.333008	23.410654	1.991305	656.368042
23.420000	651.000000	23.430655	1.531020	655.615356
23.440001	640.000000	23.450655	1.434561	654.863647
23.459999	655.000000	23.470654	1.689542	654.113159
23.480000	664.666992	23.490654	1.616152	653.363892
23.500000	646.333008	23.510654	1.549898	652.615234
23.520000	637.000000	23.530655	1.489986	651.868042
23.540001	649.666992	23.550655	1.435738	651.121460
23.559999	648.500000	23.570654	1.386572	650.376343
23.580000	651.500000	23.590654	1.341970	649.631714
23.600000	640.666992	23.610655	1.301496	648.888428
23.620001	650.666992	23.630655	1.264765	648.146240
23.640001	657.500000	23.650656	1.231441	647.404907
23.660000	651.666992	23.670654	1.201230	646.664673
23.680000	631.166992	23.690655	1.173868	645.925293
23.700001	643.500000	23.710655	1.149130	645.186768
23.720001	628.833008	23.730656	1.126813	644.449463
23.740002	655.500000	23.750656	1.154167	643.712646
23.760000	635.000000	23.770655	1.137430	642.977295
23.780001	642.500000	23.790653	1.122687	642.242676
23.799999	655.500000	23.810652	1.109817	641.508911
23.820000	663.666992	23.830652	1.098711	640.776001
23.840000	636.666992	23.850653	1.089276	640.044312
23.859999	643.833008	23.870651	1.081431	639.313354
23.879999	648.000000	23.890652	1.075100	638.583130
23.900000	643.500000	23.910652	1.070221	637.853760
23.920000	644.500000	23.930653	1.066738	637.125610
23.940001	624.000000	23.950653	1.064604	636.397949
23.959999	652.666992	23.970652	1.063779	635.671265
23.980000	642.500000	23.990652	1.064229	634.945190
24.000000	638.000000	24.010653	1.065926	634.220337
24.020000	646.500000	24.030653	1.068848	633.496216
24.040001	644.000000	24.050653	1.072978	632.772827
24.059999	658.500000	24.070652	1.078305	632.050415
24.080000	680.500000	24.090652	1.084823	631.328735
24.100000	684.833008	24.110653	1.092530	630.607666

24.120001	663.166992	24.130653	1.101429	629.887451
24.140001	650.500000	24.150654	1.111528	629.167969
24.160000	619.333008	24.170652	1.122838	628.449463
24.180000	635.833008	24.190653	1.135379	627.731567
24.200001	633.166992	24.210653	1.149171	627.014404
24.220001	633.666992	24.230654	1.164239	626.298096
24.240002	628.000000	24.250654	1.180617	625.582520
24.260000	638.500000	24.270653	1.198338	624.867798
24.280001	635.166992	24.290653	1.217447	624.153687
24.299999	633.333008	24.310652	1.237989	623.440063
24.320000	651.333008	24.330652	1.260020	622.727539
24.340000	647.000000	24.350653	1.283598	622.015503
24.359999	611.666992	24.370651	1.308787	621.304443
24.379999	641.333008	24.390652	1.335666	620.593628
24.400000	651.333008	24.410650	1.364310	619.884033
24.420000	616.000000	24.430651	1.394814	619.174561
24.440001	608.500000	24.450651	1.427277	618.466064
24.459999	620.333008	24.470650	1.461802	617.758179
24.480000	617.166992	24.490650	1.498518	617.050903
24.500000	625.333008	24.510651	1.331423	616.344360
24.520000	610.333008	24.530651	1.377556	615.638306
24.540001	602.166992	24.550652	1.426160	614.932983
24.559999	622.833008	24.570650	1.477417	614.228394
24.580000	611.500000	24.590651	1.531541	613.524170
24.600000	606.833008	24.610651	1.588754	612.820923
24.620001	614.500000	24.630651	1.649305	612.117798
24.640001	612.833008	24.650652	1.713472	611.415649
24.660000	609.333008	24.670650	1.781558	610.714111
24.680000	600.166992	24.690651	1.853921	610.012939
24.700001	608.666992	24.710651	1.930940	609.312378
24.720001	613.333008	24.730652	2.013049	608.612671
24.740002	597.166992	24.750652	2.100741	607.912964
24.760000	596.500000	24.770651	2.194562	607.214355
24.780001	599.833008	24.790651	2.295163	606.516235
24.799999	605.500000	24.810650	2.403251	605.818481
24.820000	586.333008	24.830650	2.519683	605.121338
24.840000	580.166992	24.850651	2.645416	604.424927
24.859999	598.000000	24.870649	2.781566	603.728638

24.879999	626.500000	24.890650	2.929484	603.033203
24.900000	600.500000	24.910650	3.090727	602.337891
24.920000	587.500000	24.930651	3.267166	601.643433
24.940001	595.333008	24.950651	3.461060	600.949341
24.959999	587.500000	24.970650	3.675136	600.255981
24.980000	592.833008	24.990650	3.912821	599.562988
25.000000	603.666992	25.010649	4.178296	598.870605
25.020000	599.166992	25.030649	4.476960	598.178467
25.040001	586.833008	25.050650	4.815672	597.486938
25.059999	598.500000	25.070648	5.203412	596.796021
25.080000	603.000000	25.090649	5.652362	596.105225
25.100000	602.333008	25.110649	6.179355	595.415039
25.120001	583.500000	25.130650	6.809297	594.725342
25.140001	570.833008	25.150650	7.582204	594.036011
25.160000	587.666992	25.170649	8.568023	593.347290
25.180000	583.500000	25.190649	9.894889	592.658691
25.200001	585.166992	25.210649	11.789995	591.970825
25.220001	588.000000	25.230650	14.622736	591.283203
25.240002	580.500000	25.250650	18.927942	590.596191
25.260000	600.666992	25.270649	25.425909	589.909546
25.280001	605.500000	25.290649	35.190289	589.223267
25.299999	600.333008	25.310648	50.239258	588.537354
25.320000	662.166992	25.330648	74.128586	587.852051
25.340000	688.833008	25.350649	105.550148	587.166748
25.359999	691.500000	25.370647	113.803551	586.482178
25.379999	678.166992	25.390648	81.459770	585.797974
25.400000	610.500000	25.410648	50.327354	585.113892
25.420000	588.666992	25.430649	34.111126	584.430542
25.440001	591.166992	25.450649	27.315964	583.747192
25.459999	572.500000	25.470648	25.105890	583.064453
25.480000	592.333008	25.490648	24.966274	582.382202
25.500000	583.333008	25.510649	25.823990	581.699951
25.520000	588.333008	25.530649	27.359104	581.018311
25.540001	588.833008	25.550650	29.582298	580.336670
25.559999	601.500000	25.570648	32.624382	579.655884
25.580000	597.666992	25.590649	36.675800	578.975220
25.600000	600.666992	25.610649	42.005360	578.294922
25.620001	592.833008	25.630648	49.031975	577.614746

25.640001	594.000000	25.650648	58.471512	576.935303
25.660000	621.833008	25.670647	71.620766	576.255981
25.680000	646.500000	25.690647	90.889389	575.576782
25.700001	653.833008	25.710648	120.555046	574.898193
25.720001	705.833008	25.730648	167.553558	574.219604
25.740002	782.333008	25.750648	241.978455	573.541504
25.760000	905.833008	25.770647	357.763184	572.863770
25.780001	1104.830078	25.790648	536.464355	572.186157
25.799999	1385.330078	25.810646	818.682556	571.509033
25.820000	1832.330078	25.830647	1270.327637	570.832031
25.840000	2240.670166	25.850647	1836.177368	570.155273
25.859999	2213.830078	25.870646	1909.136841	569.479126
25.879999	1800.000000	25.890646	1272.624146	568.802979
25.900000	1282.169922	25.910646	694.962891	568.127197
25.920000	950.833008	25.930647	385.351501	567.451660
25.940001	792.666992	25.950647	239.470901	566.776367
25.959999	708.000000	25.970646	170.668747	566.101440
25.980000	677.166992	25.990646	133.930939	565.426758
26.000000	628.666992	26.010647	109.460236	564.752197
26.020000	614.500000	26.030647	90.202278	564.078003
26.040001	605.166992	26.050648	74.318436	563.404175
26.059999	589.000000	26.070646	61.399593	562.730347
26.080000	605.000000	26.090647	51.112198	562.056885
26.100000	560.166992	26.110647	43.000507	561.383545
26.120001	575.166992	26.130648	36.588009	560.710571
26.140001	574.000000	26.150648	31.468496	560.037720
26.160000	578.666992	26.170647	27.330093	559.365356
26.180000	578.333008	26.190647	23.941740	558.693237
26.200001	557.166992	26.210648	21.135294	558.021118
26.220001	571.500000	26.230648	18.786247	557.349121
26.240002	556.333008	26.250647	16.801548	556.677490
26.260000	547.500000	26.270645	15.110169	556.006348
26.280001	548.333008	26.290646	13.745562	555.335083
26.299999	542.666992	26.310644	12.491592	554.663940
26.320000	541.500000	26.330645	11.400405	553.993286
26.340000	546.166992	26.350645	10.445426	553.322632
26.359999	561.000000	26.370644	9.605256	552.652344
26.379999	555.500000	26.390644	8.862309	551.982178

26.400000	553.666992	26.410645	8.202409	551.312256
26.420000	533.666992	26.430645	7.613813	550.642578
26.440001	548.666992	26.450645	7.086769	549.972778
26.459999	566.833008	26.470644	6.613175	549.303467
26.480000	573.666992	26.490644	6.186100	548.634277
26.500000	565.833008	26.510645	5.799806	547.965088
26.520000	558.333008	26.530645	5.449384	547.296387
26.540001	546.500000	26.550646	5.130636	546.627686
26.559999	539.833008	26.570644	4.839994	545.959229
26.580000	543.833008	26.590645	4.574305	545.291138
26.600000	545.333008	26.610645	4.330918	544.622803
26.620001	537.833008	26.630646	4.107508	543.954956
26.640001	533.166992	26.650646	3.902047	543.287231
26.660000	540.500000	26.670645	3.712782	542.619629
26.680000	527.666992	26.690645	3.538124	541.952271
26.700001	546.166992	26.710646	3.376724	541.284790
26.720001	550.333008	26.730646	3.227379	540.617798
26.740002	520.666992	26.750647	3.089023	539.950806
26.760000	533.500000	26.770645	2.960726	539.284180
26.780001	520.500000	26.790646	2.841625	538.617676
26.799999	538.500000	26.810644	2.730999	537.951172
26.820000	542.500000	26.830645	2.628164	537.284790
26.840000	530.833008	26.850645	2.532539	536.618652
26.859999	532.000000	26.870642	2.443614	535.952881
26.879999	516.833008	26.890642	2.360884	535.286865
26.900000	523.333008	26.910643	2.283947	534.621338
26.920000	521.333008	26.930643	2.212427	533.955566
26.940001	531.833008	26.950644	2.251451	533.290283
26.959999	522.833008	26.970642	2.192367	532.625122
26.980000	515.833008	26.990643	2.090925	531.959961
27.000000	536.500000	27.010643	2.042021	531.295044
27.020000	531.333008	27.030643	1.997261	530.630371
27.040001	523.166992	27.050644	1.956475	529.965576
27.059999	521.000000	27.070642	1.919525	529.301025
27.080000	519.500000	27.090643	1.886283	528.636719
27.100000	510.666992	27.110643	1.856656	527.972412
27.120001	526.000000	27.130644	1.830573	527.308228
27.140001	514.333008	27.150644	1.807983	526.644287

27.160000	513.333008	27.170643	1.788861	525.980347
27.180000	508.832977	27.190643	1.773197	525.316895
27.200001	510.000000	27.210644	1.761016	524.653198
27.220001	509.166992	27.230644	1.752360	523.989746
27.240002	505.666992	27.250645	1.747303	523.326294
27.260000	498.832977	27.270643	1.761163	522.663208
27.280001	514.833008	27.290644	1.764077	522.000122
27.299999	498.000000	27.310642	1.771007	521.337402
27.320000	509.832977	27.330643	1.782160	520.674561
27.340000	511.832977	27.350643	1.797787	520.011963
27.359999	498.500000	27.370642	1.818187	519.349243
27.379999	514.666992	27.390642	1.843722	518.687012
27.400000	513.333008	27.410643	1.874815	518.024536
27.420000	533.500000	27.430643	1.911969	517.362427
27.440001	529.000000	27.450644	1.955779	516.700439
27.459999	517.000000	27.470642	2.006942	516.038574
27.480000	505.500000	27.490643	2.066300	515.376831
27.500000	516.500000	27.510641	2.134832	514.715332
27.520000	505.832977	27.530642	2.213726	514.053589
27.540001	497.166992	27.550642	2.304385	513.392334
27.559999	508.666992	27.570641	2.408487	512.731079
27.580000	501.000000	27.590641	2.528092	512.069824
27.600000	492.666992	27.610641	2.665668	511.408936
27.620001	513.000000	27.630642	2.824241	510.748047
27.640001	501.166992	27.650642	3.007537	510.087158
27.660000	506.166992	27.670641	3.220156	509.426636
27.680000	505.000000	27.690641	3.467923	508.765991
27.700001	545.833008	27.710642	3.758124	508.105713
27.720001	563.666992	27.730642	4.100087	507.445312
27.740002	587.166992	27.750643	4.505855	506.785278
27.760000	627.500000	27.770641	4.991127	506.125366
27.780001	642.166992	27.790642	5.576876	505.465332
27.799999	626.500000	27.810638	6.291063	504.806030
27.820000	594.166992	27.830639	7.172693	504.146118
27.840000	541.500000	27.850639	8.276527	503.486816
27.859999	525.000000	27.870638	9.683702	502.827393
27.879999	522.833008	27.890638	11.522731	502.168091
27.900000	524.666992	27.910639	14.010651	501.509033

27.920000	512.666992	27.930639	17.533413	500.849976
27.940001	501.000000	27.950640	22.778234	500.191162
27.959999	513.000000	27.970638	30.913239	499.532593
27.980000	527.333008	27.990639	43.792892	498.873901
28.000000	532.000000	28.010639	64.200645	498.215576
28.020000	585.166992	28.030640	96.415291	497.557007
28.040001	639.166992	28.050640	147.763306	496.899048
28.059999	717.666992	28.070639	230.949539	496.240967
28.080000	847.166992	28.090639	355.798035	495.583008
28.100000	910.833008	28.110640	464.198181	494.925171
28.120001	882.500000	28.130638	409.237610	494.267822
28.140001	763.666992	28.150639	253.462250	493.610107
28.160000	632.833008	28.170637	140.553406	492.952881
28.180000	558.333008	28.190638	81.723907	492.295532
28.200001	531.500000	28.210638	53.106983	491.638550
28.220001	526.166992	28.230639	38.594002	490.981567
28.240002	521.666992	28.250639	30.212347	490.324829
28.260000	513.333008	28.270638	24.178240	489.668213
28.280001	503.666992	28.290638	19.880724	489.011963
28.299999	495.832977	28.310637	16.532335	488.355347
28.320000	481.832977	28.330637	13.942890	487.699219
28.340000	487.000000	28.350637	12.043797	487.043152
28.359999	482.332977	28.370636	10.563699	486.387390
28.379999	475.500000	28.390636	9.462779	485.731567
28.400000	477.332977	28.410637	8.655665	485.076111
28.420000	480.666992	28.430637	8.080379	484.420471
28.440001	490.832977	28.450638	7.693927	483.765381
28.459999	484.500000	28.470636	7.467678	483.110107
28.480000	481.666992	28.490637	7.383804	482.455200
28.500000	463.666992	28.510637	7.433143	481.800415
28.520000	473.832977	28.530638	7.613863	481.145691
28.540001	485.332977	28.550638	7.931068	480.491272
28.559999	470.832977	28.570637	8.397069	479.836975
28.580000	478.500000	28.590637	9.032651	479.182861
28.600000	483.832977	28.610638	9.868856	478.529114
28.620001	470.666992	28.630638	10.950494	477.875061
28.640001	465.000000	28.650639	12.341548	477.221375
28.660000	486.166992	28.670637	14.134162	476.567993

28.680000	479.166992	28.690638	16.466009	475.914673
28.700001	467.332977	28.710638	19.551979	475.261536
28.720001	479.666992	28.730639	23.750729	474.608643
28.740002	483.500000	28.750639	29.691420	473.955750
28.760000	489.666992	28.770636	38.489368	473.303528
28.780001	496.332977	28.790636	52.070675	472.651001
28.799999	500.332977	28.810635	73.553146	471.998962
28.820000	542.666992	28.830635	107.756226	471.346924
28.840000	603.666992	28.850636	162.142273	470.695068
28.859999	686.000000	28.870634	249.222000	470.043457
28.879999	851.833008	28.890635	390.765656	469.392151
28.900000	1078.669922	28.910635	610.712830	468.740723
28.920000	1301.000000	28.930635	848.137085	468.089783
28.940001	1250.669922	28.950636	839.136719	467.438843
28.959999	1039.330078	28.970634	562.852295	466.788452
28.980000	785.166992	28.990635	316.857666	466.137939
29.000000	611.000000	29.010635	179.925720	465.487671
29.020000	552.666992	29.030636	111.906288	464.837708
29.040001	523.000000	29.050636	77.515327	464.188049
29.059999	507.000000	29.070635	58.244499	463.538147
29.080000	486.000000	29.090635	45.705704	462.888916
29.100000	476.166992	29.110636	36.514893	462.239685
29.120001	473.500000	29.130636	29.414221	461.590942
29.140001	464.500000	29.150637	23.881752	460.942139
29.160000	466.166992	29.170635	19.590855	460.293701
29.180000	466.166992	29.190636	16.266411	459.645508
29.200001	457.500000	29.210636	13.676193	458.997437
29.220001	450.000000	29.230637	11.636109	458.349548
29.240002	469.832977	29.250637	10.077295	457.702087
29.260000	462.832977	29.270636	8.763039	457.054688
29.280001	450.166992	29.290636	7.761243	456.407593
29.299999	466.166992	29.310635	6.873883	455.760742
29.320000	453.666992	29.330635	6.133095	455.114197
29.340000	437.832977	29.350636	5.509284	454.467896
29.359999	461.000000	29.370634	4.979866	453.821777
29.379999	444.500000	29.390635	4.527255	453.175842
29.400000	450.500000	29.410633	4.137886	452.530212
29.420000	446.166992	29.430634	3.800901	451.885132

29.440001	453.166992	29.450634	3.507742	451.240051
29.459999	449.666992	29.470633	3.251512	450.595093
29.480000	445.832977	29.490633	3.026551	449.950500
29.500000	452.000000	29.510633	2.828296	449.306274
29.520000	456.832977	29.530634	2.652970	448.662231
29.540001	454.832977	29.550634	2.497440	448.018372
29.559999	460.332977	29.570633	2.359110	447.375061
29.580000	470.000000	29.590633	2.235761	446.731873
29.600000	480.500000	29.610634	2.125563	446.088989
29.620001	480.166992	29.630634	2.026951	445.446289
29.640001	484.500000	29.650635	1.938593	444.804016
29.660000	476.332977	29.670633	1.915220	444.161865
29.680000	477.166992	29.690634	1.845045	443.520081
29.700001	471.166992	29.710634	1.782190	442.878540
29.720001	445.666992	29.730635	1.725930	442.237488
29.740002	459.666992	29.750635	1.675637	441.596436
29.760000	444.000000	29.770634	1.630768	440.956116
29.780001	451.166992	29.790634	1.590839	440.315796
29.799999	442.332977	29.810633	1.555444	439.675720
29.820000	446.832977	29.830633	1.524214	439.036255
29.840000	454.666992	29.850634	1.496836	438.396973
29.859999	434.332977	29.870632	1.473036	437.757996
29.879999	444.166992	29.890633	1.452567	437.119141
29.900000	431.832977	29.910633	1.435222	436.480957
29.920000	447.166992	29.930634	1.420815	435.842834
29.940001	440.332977	29.950634	1.409187	435.205139
29.959999	433.500000	29.970633	1.314853	434.567810
29.980000	433.000000	29.990633	1.310477	433.930664
30.000000	446.832977	30.010633	1.308447	433.294128
30.020000	434.666992	30.030634	1.308679	432.657654
30.040001	429.332977	30.050632	1.311103	432.021667
30.059999	430.000000	30.070631	1.315666	431.386169
30.080000	426.666992	30.090631	1.322323	430.750732
30.100000	435.666992	30.110632	1.331043	430.115845
30.120001	429.832977	30.130632	1.341805	429.481262
30.140001	429.500000	30.150633	1.354597	428.846924
30.160000	436.666992	30.170631	1.369417	428.213257
30.180000	421.666992	30.190632	1.386277	427.579773

30.200001	430.666992	30.210632	1.405193	426.946655
30.220001	438.500000	30.230633	1.426194	426.313843
30.240002	432.832977	30.250633	1.449316	425.681458
30.260000	434.500000	30.270632	1.474602	425.049438
30.280001	450.166992	30.290632	1.502115	424.417847
30.299999	441.332977	30.310631	1.531914	423.786743
30.320000	438.332977	30.330631	1.564081	423.155823
30.340000	443.666992	30.350632	1.598700	422.525452
30.359999	433.332977	30.370630	1.635867	421.895447
30.379999	422.000000	30.390631	1.675697	421.265747
30.400000	428.666992	30.410631	1.718311	420.636475
30.420000	434.332977	30.430632	1.763844	420.007568
30.440001	417.332977	30.450632	1.812448	419.379211
30.459999	431.666992	30.470631	1.864283	418.751465
30.480000	427.500000	30.490631	1.919543	418.123657
30.500000	434.500000	30.510632	1.978426	417.496521
30.520000	432.166992	30.530632	2.041153	416.869751
30.540001	448.832977	30.550632	2.107970	416.243530
30.559999	447.000000	30.570631	2.179137	415.617493
30.580000	467.166992	30.590631	2.254966	414.991943
30.600000	449.666992	30.610632	2.335773	414.367004
30.620001	458.500000	30.630632	2.421919	413.742371
30.640001	456.666992	30.650633	2.499302	413.118347
30.660000	462.832977	30.670631	2.597734	412.494690
30.680000	479.500000	30.690630	2.702799	411.871399
30.700001	511.332977	30.710630	2.815041	411.248657
30.720001	526.000000	30.730631	2.935040	410.626282
30.740002	539.500000	30.750631	3.063449	410.004395
30.760000	512.000000	30.770630	3.200982	409.383118
30.780001	483.832977	30.790630	3.348473	408.762268
30.799999	465.166992	30.810629	3.506795	408.141663
30.820000	448.000000	30.830629	3.676985	407.521729
30.840000	452.000000	30.850630	3.860155	406.902161
30.859999	419.166992	30.870628	4.057550	406.283203
30.879999	437.832977	30.890629	4.270629	405.664612
30.900000	443.500000	30.910629	4.500980	405.046631
30.920000	467.500000	30.930630	4.750416	404.428894
30.940001	475.832977	30.950630	4.917349	403.812012

30.959999	496.000000	30.970629	5.213784	403.195251
30.980000	557.333008	30.990629	5.536306	402.579346
31.000000	613.500000	31.010630	5.887945	401.963623
31.020000	658.166992	31.030630	6.272202	401.348633
31.040001	647.333008	31.050631	6.693116	400.734009
31.059999	607.166992	31.070629	7.155306	400.120178
31.080000	542.833008	31.090630	7.664280	399.506531
31.100000	515.666992	31.110630	8.226313	398.893555
31.120001	468.332977	31.130630	8.848804	398.281067
31.140001	437.000000	31.150631	9.540458	397.669189
31.160000	439.500000	31.170630	10.311494	397.057739
31.180000	447.666992	31.190630	11.174288	396.446838
31.200001	421.000000	31.210630	12.143410	395.836548
31.220001	429.666992	31.230631	13.236453	395.226746
31.240002	436.166992	31.250631	14.474713	394.617554
31.260000	442.666992	31.270630	15.884011	394.008850
31.280001	434.166992	31.290630	17.496489	393.400757
31.299999	435.832977	31.310629	19.351269	392.793213
31.320000	427.666992	31.330627	21.497719	392.186340
31.340000	408.332977	31.350628	23.998087	391.579712
31.359999	410.332977	31.370626	26.930779	390.973877
31.379999	423.332977	31.390627	30.397804	390.368713
31.400000	407.666992	31.410627	34.530937	389.763855
31.420000	426.000000	31.430628	39.504253	389.159729
31.440001	432.832977	31.450628	45.550541	388.556091
31.459999	428.166992	31.470627	52.985695	387.953247
31.480000	428.166992	31.490627	62.250889	387.350769
31.500000	448.000000	31.510628	73.973686	386.749023
31.520000	449.332977	31.530628	89.087433	386.147766
31.540001	475.666992	31.550629	109.051079	385.547241
31.559999	478.666992	31.570627	136.267426	384.947021
31.580000	519.666992	31.590628	174.865784	384.347778
31.600000	575.666992	31.610628	231.987320	383.748962
31.620001	647.166992	31.630629	319.784851	383.150879
31.640001	755.333008	31.650629	458.318573	382.553223
31.660000	944.333008	31.670628	680.098511	381.956360
31.680000	1306.169922	31.690628	1038.799927	381.360046
31.700001	1893.330078	31.710629	1625.672363	380.764404

31.720001	2964.500000	31.730629	2584.963135	380.169250
31.740002	4403.500000	31.750629	4017.389404	379.574829
31.760000	5545.169922	31.770628	5405.708984	378.981018
31.780001	5350.000000	31.790628	5248.089844	378.387939
31.799999	3893.330078	31.810627	3598.477539	377.795532
31.820000	2411.170166	31.830627	2094.550537	377.203735
31.840000	1500.000000	31.850628	1220.049561	376.612366
31.859999	1024.500000	31.870626	767.455688	376.021912
31.879999	816.500000	31.890627	530.578491	375.431946
31.900000	674.666992	31.910627	397.462280	374.842773
31.920000	608.833008	31.930628	315.594391	374.254150
31.940001	560.500000	31.950628	262.204773	373.666321
31.959999	537.000000	31.970627	228.060471	373.079163
31.980000	538.500000	31.990625	209.877182	372.492554
32.000000	520.500000	32.010628	207.557480	371.906738
32.020000	543.000000	32.030628	223.876389	371.321594
32.040001	577.500000	32.050629	265.392334	370.737061
32.060001	639.833008	32.070629	344.257111	370.153320
32.080002	773.000000	32.090630	481.544128	369.570007
32.099998	1014.830017	32.110626	713.701233	368.987671
32.119999	1410.500000	32.130627	1101.696899	368.406128
32.139999	2005.000000	32.150627	1713.972290	367.825073
32.160000	2598.000000	32.170628	2448.564697	367.244812
32.180000	2848.830078	32.190628	2691.539062	366.665222
32.200001	2249.000000	32.210629	2057.894775	366.086182
32.220001	1516.169922	32.230629	1248.597656	365.508057
32.240002	1034.330078	32.250629	723.853210	364.930603
32.260002	749.833008	32.270630	443.603058	364.353943
32.280003	646.666992	32.290630	296.448181	363.777832
32.299999	557.666992	32.310627	214.215622	363.202576
32.320000	505.166992	32.330624	163.421997	362.628296
32.340000	492.500000	32.350624	128.880035	362.054382
32.360001	471.166992	32.370625	103.894501	361.481323
32.380001	464.666992	32.390625	85.336311	360.908936
32.400002	448.332977	32.410625	71.474274	360.337463
32.420002	454.332977	32.430626	61.146908	359.766541
32.440002	445.000000	32.450626	53.499027	359.196533
32.460003	444.666992	32.470627	47.900154	358.627075

32.480003	422.000000	32.490627	43.901627	358.058411
32.500000	419.332977	32.510624	41.199211	357.490784
32.520000	410.666992	32.530624	39.598652	356.923706
32.540001	394.500000	32.550625	38.994987	356.357361
32.560001	393.166992	32.570625	39.356529	355.791992
32.580002	394.000000	32.590626	40.721252	355.227234
32.599998	388.166992	32.610622	43.201809	354.663452
32.619999	388.166992	32.630623	47.004993	354.100098
32.639999	386.166992	32.650623	52.464680	353.537964
32.660000	388.832977	32.670624	60.112011	352.976196
32.680000	407.000000	32.690624	70.806725	352.415344
32.700001	415.500000	32.710625	85.987083	351.855286
32.720001	428.666992	32.730625	108.123283	351.296204
32.740002	457.000000	32.750626	141.482224	350.737671
32.760002	487.500000	32.770626	193.327423	350.179993
32.780003	568.500000	32.790627	275.758026	349.623413
32.799999	679.333008	32.810623	408.710327	349.067322
32.820000	898.333008	32.830624	625.681335	348.512085
32.840000	1284.500000	32.850624	983.366821	347.957703
32.860001	1951.169922	32.870625	1567.289795	347.404175
32.880001	2775.170166	32.890625	2421.199951	346.851440
32.900002	3411.830078	32.910625	3206.125488	346.299438
32.920002	3310.170166	32.930626	3076.916748	345.748413
32.940002	2462.500000	32.950626	2117.583252	345.198242
32.960003	1557.169922	32.970627	1242.745972	344.648682
32.980003	1007.330017	32.990627	725.473511	344.100220
33.000000	719.833008	33.010624	452.035431	343.552551
33.020000	559.666992	33.030624	305.092712	343.005554
33.040001	485.332977	33.050625	219.927429	342.459473
33.060001	459.000000	33.070625	165.535507	341.914185
33.080002	426.332977	33.090626	127.817436	341.369873
33.099998	397.332977	33.110622	100.333557	340.826355
33.119999	385.832977	33.130623	79.838707	340.283813
33.139999	375.666992	33.150623	64.407333	339.741821
33.160000	364.000000	33.170624	52.700722	339.201050
33.180000	378.166992	33.190624	43.734177	338.660828
33.200001	364.166992	33.210625	36.783386	338.121643
33.220001	357.666992	33.230625	31.323927	337.583374

33.240002	364.000000	33.250626	26.979933	337.045837
33.260002	342.166992	33.270626	23.481899	336.509338
33.280003	346.832977	33.290627	20.634958	335.973450
33.299999	337.666992	33.310623	18.296864	335.438660
33.320000	336.332977	33.330624	16.360918	334.904785
33.340000	343.332977	33.350624	14.748098	334.371643
33.360001	341.000000	33.370625	13.398010	333.839539
33.380001	349.166992	33.390625	12.264363	333.308228
33.400002	347.166992	33.410625	11.311436	332.777832
33.420002	347.166992	33.430626	10.511566	332.248352
33.440002	352.500000	33.450626	9.843352	331.719727
33.460003	343.000000	33.470627	9.290359	331.192078
33.480003	361.500000	33.490627	8.840189	330.665222
33.500000	342.000000	33.510624	8.483893	330.139465
33.520000	335.666992	33.530624	8.215299	329.614441
33.540001	355.332977	33.550625	8.041475	329.090393
33.560001	334.166992	33.570625	7.941421	328.567261
33.580002	335.332977	33.590626	7.927205	328.044983
33.599998	343.000000	33.610622	8.003838	327.523804
33.619999	326.666992	33.630623	8.179868	327.003357
33.639999	332.000000	33.650620	8.467981	326.483948
33.660000	327.500000	33.670620	8.886430	325.965454
33.680000	338.000000	33.690620	9.460403	325.447876
33.700001	337.666992	33.710621	10.224722	324.931152
33.720001	325.166992	33.730621	11.227527	324.415466
33.740002	345.500000	33.750622	12.536009	323.900635
33.760002	340.000000	33.770622	14.245506	323.386780
33.780003	336.666992	33.790623	16.494774	322.873718
33.799999	342.000000	33.810619	19.492474	322.361938
33.820000	357.000000	33.830620	23.569609	321.850952
33.840000	343.166992	33.850620	29.268620	321.340698
33.860001	354.166992	33.870621	37.510357	320.831604
33.880001	364.166992	33.890621	49.871658	320.323547
33.900002	383.166992	33.910622	69.031578	319.816223
33.920002	410.166992	33.930622	99.485725	319.310059
33.940002	445.332977	33.950623	148.763580	318.804688
33.960003	530.500000	33.970623	229.624557	318.300354
33.980003	684.000000	33.990623	363.459198	317.796936

34.000000	884.666992	34.010620	580.242737	317.294678
34.020000	1215.330078	34.030621	885.989746	316.793152
34.040001	1396.500000	34.050621	1139.719360	316.292786
34.060001	1333.669922	34.070621	1062.767700	315.793213
34.080002	1034.000000	34.090622	725.842041	315.294739
34.099998	750.833008	34.110619	429.253479	314.797241
34.119999	594.500000	34.130619	253.181305	314.300598
34.139999	489.000000	34.150620	158.703613	313.805054
34.160000	482.500000	34.170620	107.044594	313.310486
34.180000	460.166992	34.190620	76.704620	312.816772
34.200001	448.832977	34.210621	57.269913	312.324219
34.220001	429.000000	34.230621	43.881088	311.832520
34.240002	411.332977	34.250622	34.224842	311.341919
34.260002	415.332977	34.270622	27.094488	310.852234
34.280003	418.832977	34.290623	21.691446	310.363586
34.299999	441.666992	34.310619	17.668400	309.876038
34.320000	475.000000	34.330620	14.596321	309.389343
34.340000	477.832977	34.350620	12.220332	308.903687
34.360001	452.832977	34.370621	10.357092	308.419006
34.380001	434.000000	34.390621	8.875833	307.935364
34.400002	408.166992	34.410622	7.683009	307.452698
34.420002	358.000000	34.430622	6.711081	306.971069
34.440002	357.166992	34.450623	5.910636	306.490479
34.460003	339.166992	34.470623	5.245016	306.010803
34.480003	334.832977	34.490623	4.686649	305.532166
34.500000	329.332977	34.510620	4.214612	305.054688
34.520000	323.500000	34.530621	3.812534	304.578064
34.540001	315.332977	34.550621	3.467894	304.102600
34.560001	325.500000	34.570621	3.170760	303.628052
34.580002	340.166992	34.590622	2.913251	303.154480
34.599998	332.166992	34.610619	2.689095	302.682068
34.619999	344.500000	34.630619	2.493119	302.210632
34.639999	336.000000	34.650620	2.321243	301.740112
34.660000	319.832977	34.670620	2.170103	301.270813
34.680000	318.332977	34.690620	2.036944	300.802368
34.700001	306.000000	34.710621	1.919505	300.335083
34.720001	300.000000	34.730621	1.815927	299.868774
34.740002	307.666992	34.750622	1.724688	299.403503

34.760002	304.500000	34.770622	1.644545	298.939209
34.780003	308.332977	34.790623	1.574497	298.476013
34.799999	305.666992	34.810619	1.513760	298.013916
34.820000	307.000000	34.830620	1.461706	297.552795
34.840000	294.666992	34.850620	1.417913	297.092712
34.860001	292.666992	34.870621	1.382111	296.633728
34.880001	297.166992	34.890621	1.354194	296.175720
34.900002	290.500000	34.910622	1.334226	295.718689
34.920002	292.666992	34.930622	1.322449	295.262817
34.940002	290.666992	34.950623	1.319315	294.807922
34.960003	289.666992	34.970623	1.325511	294.354126
34.980003	304.500000	34.990620	1.342011	293.901489
35.000000	306.832977	35.010616	1.370144	293.449890
35.020000	298.000000	35.030617	1.411705	292.999268
35.040001	306.666992	35.050617	1.469056	292.549622
35.060001	311.166992	35.070618	1.545338	292.101074
35.080002	301.832977	35.090618	1.644726	291.653564
35.099998	309.332977	35.110615	1.772786	291.207214
35.119999	308.332977	35.130615	1.937127	290.761902
35.139999	304.832977	35.150616	2.080590	290.317627
35.160000	298.666992	35.170616	2.353785	289.874390
35.180000	291.000000	35.190617	2.708565	289.432251
35.200001	296.666992	35.210617	3.175090	288.991028
35.220001	290.832977	35.230618	3.799279	288.550964
35.240002	284.832977	35.250618	4.584156	288.112061
35.260002	289.666992	35.270618	5.790701	287.674133
35.280003	293.332977	35.290619	7.551247	287.237244
35.299999	294.166992	35.310616	10.211920	286.801636
35.320000	294.000000	35.330616	14.363798	286.366882
35.340000	307.666992	35.350616	21.007610	285.933228
35.360001	317.666992	35.370617	31.847544	285.500671
35.380001	326.332977	35.390617	49.785690	285.069214
35.400002	361.000000	35.410618	79.482933	284.638794
35.420002	404.166992	35.430618	126.215103	284.209412
35.440002	458.666992	35.450619	185.533508	283.781128
35.460003	524.833008	35.470619	220.241776	283.353882
35.480003	478.166992	35.490620	188.749863	282.927795
35.500000	407.000000	35.510616	124.106064	282.502930

35.520000	365.000000	35.530617	73.642052	282.078918
35.540001	342.332977	35.550617	44.275345	281.656006
35.560001	330.832977	35.570618	28.254368	281.234192
35.580002	318.166992	35.590618	19.236881	280.813477
35.599998	314.500000	35.610615	13.797273	280.393921
35.619999	309.166992	35.630615	10.260024	279.975342
35.639999	293.332977	35.650616	7.819629	279.557861
35.660000	284.000000	35.670616	6.070632	279.141479
35.680000	281.000000	35.690617	4.789382	278.726196
35.700001	284.500000	35.710617	3.837548	278.311890
35.720001	283.166992	35.730618	3.121825	277.898743
35.740002	262.166992	35.750618	2.576676	277.486694
35.760002	276.332977	35.770618	2.155635	277.075745
35.780003	273.000000	35.790619	1.825785	276.665833
35.799999	277.166992	35.810616	1.563823	276.257141
35.820000	272.332977	35.830616	1.352990	275.849365
35.840000	276.166992	35.850616	1.181312	275.442749
35.860001	282.000000	35.870617	1.039991	275.037231
35.880001	275.666992	35.890617	0.922500	274.632751
35.900002	275.332977	35.910618	0.823929	274.229431
35.920002	269.666992	35.930618	0.740538	273.827148
35.940002	275.166992	35.950619	0.669442	273.426025
35.960003	275.332977	35.970619	0.608397	273.025879
35.980003	265.500000	35.990620	0.555634	272.626892
36.000000	269.832977	36.010616	0.509755	272.229004
36.020000	270.332977	36.030617	0.469617	271.832214
36.040001	274.166992	36.050617	0.434321	271.436401
36.060001	271.000000	36.070618	0.403125	271.041809
36.080002	272.166992	36.090618	0.375422	270.648193
36.099998	267.832977	36.110615	0.350717	270.255859
36.119999	269.000000	36.130615	0.328583	269.864502
36.139999	258.332977	36.150616	0.308679	269.474182
36.160000	264.832977	36.170616	0.290713	269.085083
36.180000	261.332977	36.190617	0.219375	268.696960
36.200001	251.833008	36.210617	0.205472	268.309814
36.220001	260.332977	36.230618	0.192854	267.924011
36.240002	264.166992	36.250618	0.181370	267.539124
36.260002	266.832977	36.270618	0.170889	267.155457

36.280003	270.666992	36.290619	0.161771	266.772766
36.299999	258.832977	36.310616	0.152991	266.391357
36.320000	246.833008	36.330616	0.144919	266.010803
36.340000	239.333008	36.350616	0.137483	265.631470
36.360001	244.167007	36.370613	0.130619	265.253296
36.380001	249.833008	36.390614	0.124268	264.876099
36.400002	243.833008	36.410614	0.079929	264.500122
36.420002	268.832977	36.430614	0.075347	264.125122
36.440002	256.332977	36.450615	0.071119	263.751160
36.460003	262.000000	36.470615	0.067212	263.378235
36.480003	257.500000	36.490616	0.063596	263.006592
36.500000	257.666992	36.510612	0.060246	262.635986
36.520000	252.833008	36.530613	0.057136	262.266418
36.540001	250.500000	36.550613	0.054246	261.897949
36.560001	252.667007	36.570614	0.051557	261.530640
36.580002	252.000000	36.590614	0.049053	261.164368
36.599998	252.167007	36.610607	0.046719	260.799377
36.619999	250.167007	36.630608	0.044540	260.435181
36.639999	257.666992	36.650608	0.042504	260.072144
36.660000	250.000000	36.670609	0.040600	259.710327
36.680000	247.167007	36.690609	0.038818	259.349426
36.700001	252.500000	36.710609	0.037149	258.989746
36.720001	246.833008	36.730610	0.035584	258.631104
36.740002	242.333008	36.750610	0.034117	258.273499
36.760002	253.167007	36.770611	0.032740	257.916931
36.780003	247.333008	36.790611	0.031448	257.561584
36.799999	254.167007	36.810608	0.030234	257.207275
36.820000	250.000000	36.830608	0.029093	256.854065
36.840000	241.667007	36.850609	0.028022	256.502014
36.860001	240.833008	36.870609	0.027015	256.150879
36.880001	248.167007	36.890610	0.026068	255.800964
36.900002	245.667007	36.910610	0.025180	255.452026
36.920002	252.000000	36.930611	0.024345	255.104126
36.940002	251.500000	36.950611	0.023561	254.757385
36.960003	243.667007	36.970612	0.022827	254.411804
36.980003	246.333008	36.990612	0.022138	254.067200
37.000000	242.667007	37.010609	0.021494	253.723755
37.020000	239.333008	37.030609	0.020893	253.381348

37.040001	245.667007	37.050610	0.020332	253.039978
37.060001	247.667007	37.070610	0.019811	252.699707
37.080002	249.333008	37.090611	0.019329	252.360474
37.099998	243.667007	37.110607	0.018884	252.022461
37.119999	238.333008	37.130608	0.018476	251.685364
37.139999	251.000000	37.150608	0.018104	251.349487
37.160000	239.000000	37.170609	0.022627	251.014648
37.180000	237.167007	37.190609	0.022475	250.680664
37.200001	242.833008	37.210609	0.022366	250.347961
37.220001	245.167007	37.230610	0.022300	250.016357
37.240002	244.000000	37.250610	0.023800	249.685669
37.260002	247.000000	37.270611	0.023871	249.356079
37.280003	258.000000	37.290611	0.023992	249.027649
37.299999	255.333008	37.310608	0.024164	248.700317
37.320000	260.666992	37.330608	0.024389	248.373962
37.340000	262.332977	37.350609	0.037439	248.048706
37.360001	262.332977	37.370609	0.038107	247.724548
37.380001	241.333008	37.390610	0.038851	247.401367
37.400002	243.000000	37.410610	0.039677	247.079285
37.420002	243.000000	37.430611	0.030735	246.758179
37.440002	249.167007	37.450611	0.032026	246.438293
37.460003	237.333008	37.470612	0.033412	246.119263
37.480003	243.167007	37.490612	0.034903	245.801453
37.500000	235.833008	37.510609	0.036513	245.484741
37.520000	238.667007	37.530609	0.038255	245.169006
37.540001	239.500000	37.550610	0.040149	244.854309
37.560001	226.833008	37.570610	0.042213	244.540649
37.580002	230.000000	37.590611	0.044474	244.228088
37.599998	240.333008	37.610607	0.046959	243.916626
37.619999	244.000000	37.630608	0.049705	243.606140
37.639999	235.167007	37.650608	0.052755	243.296753
37.660000	242.833008	37.670609	0.056160	242.988281
37.680000	247.000000	37.690609	0.059985	242.680908
37.700001	229.500000	37.710609	0.064310	242.374695
37.720001	239.833008	37.730610	0.069234	242.069397
37.740002	244.833008	37.750610	0.079909	241.765137
37.760002	235.833008	37.770607	0.086599	241.462097
37.780003	246.833008	37.790607	0.094395	241.159973

37.799999	248.667007	37.810604	0.103566	240.859009
37.820000	247.167007	37.830605	0.114478	240.558838
37.840000	238.833008	37.850605	0.127616	240.259827
37.860001	253.833008	37.870605	0.143645	239.961853
37.880001	240.667007	37.890606	0.163489	239.664978
37.900002	237.833008	37.910606	0.188463	239.369019
37.920002	242.833008	37.930607	0.220486	239.074036
37.940002	241.333008	37.950607	0.262460	238.780151
37.960003	235.333008	37.970608	0.318927	238.487366
37.980003	237.333008	37.990608	0.397257	238.195557
38.000000	228.667007	38.010605	0.509735	237.904785
38.020000	239.000000	38.030605	0.677359	237.614990
38.040001	223.500000	38.050606	0.936170	237.326294
38.060001	219.000000	38.070606	1.348545	237.038574
38.080002	226.000000	38.090607	2.022390	236.751831
38.099998	231.500000	38.110603	3.140670	236.466248
38.119999	230.167007	38.130604	4.988268	236.181580
38.139999	233.667007	38.150604	7.863107	235.897888
38.160000	237.500000	38.170605	11.492414	235.615173
38.180000	233.833008	38.190605	13.851241	235.333496
38.200001	234.167007	38.210606	12.460032	235.052917
38.220001	241.833008	38.230606	8.673439	234.773285
38.240002	227.000000	38.250607	5.369276	234.494659
38.260002	230.333008	38.270607	3.318051	234.217041
38.280003	217.667007	38.290607	2.158837	233.940491
38.299999	224.500000	38.310604	1.496653	233.664886
38.320000	224.167007	38.330605	1.099234	233.390228
38.340000	230.000000	38.350605	0.847688	233.116608
38.360001	232.667007	38.370605	0.681753	232.843964
38.380001	217.500000	38.390606	0.569663	232.572235
38.400002	209.500000	38.410606	0.493458	232.301636
38.420002	218.667007	38.430607	0.444732	232.032043
38.440002	222.667007	38.450607	0.411433	231.763245
38.460003	221.500000	38.470608	0.391315	231.495544
38.480003	219.667007	38.490608	0.381217	231.228851
38.500000	219.500000	38.510605	0.379032	230.963104
38.520000	218.000000	38.530605	0.383383	230.698425
38.540001	223.667007	38.550606	0.393417	230.434692

38.560001	215.000000	38.570606	0.408662	230.171875
38.580002	219.333008	38.590607	0.428933	229.910065
38.599998	218.833008	38.610603	0.454284	229.649292
38.619999	216.667007	38.630604	0.484995	229.389313
38.639999	220.667007	38.650604	0.521540	229.130463
38.660000	230.167007	38.670605	0.564620	228.872528
38.680000	211.333008	38.690605	0.615182	228.615509
38.700001	212.833008	38.710606	0.674475	228.359497
38.720001	229.000000	38.730606	0.744112	228.104370
38.740002	231.167007	38.750607	0.826182	227.850342
38.760002	222.333008	38.770607	0.923384	227.597198
38.780003	218.500000	38.790607	1.039235	227.345032
38.799999	220.333008	38.810604	1.178319	227.093811
38.820000	217.500000	38.830605	1.347046	226.843506
38.840000	225.833008	38.850605	1.553177	226.594177
38.860001	226.500000	38.870605	1.808164	226.345795
38.880001	222.667007	38.890606	2.127597	226.098358
38.900002	218.167007	38.910606	2.533551	225.851868
38.920002	231.667007	38.930607	3.058086	225.606232
38.940002	224.667007	38.950607	3.749180	225.361694
38.960003	231.667007	38.970608	4.681124	225.118011
38.980003	235.833008	38.990608	5.973102	224.875244
39.000000	228.333008	39.010605	7.822038	224.633392
39.020000	236.333008	39.030605	10.563396	224.392517
39.040001	241.667007	39.050606	14.773917	224.152557
39.060001	254.000000	39.070606	21.456352	223.913544
39.080002	249.333008	39.090607	32.353371	223.675415
39.099998	272.166992	39.110603	50.434135	223.438232
39.119999	302.000000	39.130604	80.400589	223.201904
39.139999	361.000000	39.150604	127.723938	222.966614
39.160000	437.500000	39.170605	190.870438	222.732117
39.180000	520.500000	39.190601	241.873108	222.498657
39.200001	481.000000	39.210602	233.547836	222.266022
39.220001	413.000000	39.230602	172.020279	222.034332
39.240002	346.666992	39.250603	108.967255	221.803406
39.260002	283.332977	39.270603	67.146210	221.573547
39.280003	275.000000	39.290604	42.989403	221.344452
39.299999	251.833008	39.310600	29.192301	221.116425

39.320000	245.833008	39.330601	21.018644	220.889252
39.340000	229.667007	39.350601	15.967943	220.662933
39.360001	225.333008	39.370602	12.761776	220.437439
39.380001	236.167007	39.390602	10.730048	220.212830
39.400002	218.333008	39.410603	9.503877	219.989288
39.420002	232.833008	39.430603	8.872066	219.766510
39.440002	219.500000	39.450603	8.714918	219.544556
39.460003	217.000000	39.470604	8.973538	219.323547
39.480003	229.667007	39.490604	9.636152	219.103363
39.500000	224.500000	39.510601	10.733871	218.884216
39.520000	228.000000	39.530602	12.345482	218.665771
39.540001	229.833008	39.550602	14.609259	218.448273
39.560001	226.333008	39.570602	17.750517	218.231537
39.580002	226.333008	39.590603	22.131683	218.015839
39.599998	248.333008	39.610600	28.342857	217.800873
39.619999	236.667007	39.630600	37.373867	217.586792
39.639999	264.000000	39.650600	50.908062	217.373566
39.660000	272.166992	39.670601	71.861992	217.161255
39.680000	310.332977	39.690601	105.324875	216.949768
39.700001	357.500000	39.710602	160.158279	216.739044
39.720001	456.500000	39.730602	251.432556	216.529266
39.740002	621.333008	39.750603	402.613281	216.320251
39.760002	872.166992	39.770603	639.812073	216.112183
39.780003	1210.169922	39.790604	951.288696	215.904846
39.799999	1430.169922	39.810600	1194.597656	215.698578
39.820000	1352.669922	39.830601	1145.111328	215.492920
39.840000	1068.500000	39.850601	841.757202	215.288147
39.860001	763.833008	39.870602	532.853394	215.084167
39.880001	536.833008	39.890602	326.598328	214.881073
39.900002	409.500000	39.910603	206.100388	214.678741
39.920002	318.166992	39.930603	136.221954	214.477356
39.940002	290.166992	39.950603	93.985603	214.276642
39.960003	273.666992	39.970604	67.105392	214.076813
39.980003	252.167007	39.990604	49.220284	213.877777
40.000000	245.000000	40.010601	36.920345	213.679688
40.020000	241.833008	40.030602	28.253633	213.482300
40.040001	234.500000	40.050602	22.036827	213.285706
40.060001	226.167007	40.070602	17.510195	213.089844

40.080002	231.000000	40.090603	14.171525	212.894897
40.099998	224.833008	40.110600	11.683775	212.700775
40.119999	218.833008	40.130600	9.817301	212.507385
40.139999	207.667007	40.150600	8.419365	212.314819
40.160000	213.000000	40.170601	7.386853	212.123016
40.180000	220.500000	40.190601	6.654782	211.931976
40.200001	222.000000	40.210602	6.188302	211.741730
40.220001	210.167007	40.230602	5.980741	211.552307
40.240002	219.333008	40.250603	6.057833	211.363647
40.260002	215.500000	40.270603	6.491408	211.175781
40.280003	213.000000	40.290604	7.422652	210.988647
40.299999	212.167007	40.310600	9.116908	210.802368
40.320000	221.500000	40.330601	12.051358	210.616821
40.340000	220.667007	40.350601	17.066477	210.432068
40.360001	244.500000	40.370602	25.594522	210.248016
40.380001	233.833008	40.390602	39.844223	210.064758
40.400002	269.666992	40.410603	62.192150	209.882233
40.420002	287.666992	40.430603	91.311028	209.700409
40.440002	313.832977	40.450603	113.782280	209.519470
40.460003	316.832977	40.470604	109.084114	209.339233
40.480003	264.500000	40.490604	80.849800	209.159729
40.500000	243.333008	40.510601	51.824707	208.981018
40.520000	225.000000	40.530602	32.216461	208.802979
40.540001	215.667007	40.550602	20.648743	208.625732
40.560001	204.167007	40.570602	13.892584	208.449188
40.580002	208.500000	40.590603	9.796906	208.273407
40.599998	203.167007	40.610600	7.202923	208.098358
40.619999	202.667007	40.630600	5.509147	207.924042
40.639999	205.833008	40.650597	4.400780	207.750458
40.660000	195.667007	40.670597	3.712406	207.577637
40.680000	207.333008	40.690598	3.371879	207.405396
40.700001	201.833008	40.710598	3.381308	207.233978
40.720001	208.167007	40.730598	3.821045	207.063232
40.740002	203.167007	40.750599	4.854784	206.893280
40.760002	223.667007	40.770599	6.661110	206.723969
40.780003	228.500000	40.790600	9.069667	206.555328
40.799999	215.333008	40.810596	10.839944	206.387512
40.820000	216.667007	40.830597	10.222780	206.220276

40.840000	210.667007	40.850597	7.641931	206.053802
40.860001	206.833008	40.870598	5.057065	205.888031
40.880001	207.667007	40.890598	3.306085	205.722900
40.900002	215.667007	40.910599	2.255921	205.558533
40.920002	203.667007	40.930599	1.626790	205.394836
40.940002	201.000000	40.950600	1.232872	205.231781
40.960003	205.000000	40.970600	0.973183	205.069397
40.980003	213.833008	40.990601	0.794341	204.907745
41.000000	209.833008	41.010597	0.667036	204.746765
41.020000	218.167007	41.030598	0.574064	204.586487
41.040001	229.000000	41.050598	0.504805	204.426849
41.060001	227.500000	41.070599	0.452352	204.267914
41.080002	213.667007	41.090599	0.412102	204.109558
41.099998	225.167007	41.110596	0.380937	203.951965
41.119999	221.167007	41.130596	0.356707	203.794891
41.139999	212.500000	41.150597	0.337937	203.638580
41.160000	210.333008	41.170597	0.322110	203.482910
41.180000	204.500000	41.190598	0.311516	203.327850
41.200001	196.667007	41.210598	0.304124	203.173523
41.220001	201.667007	41.230598	0.299592	203.019806
41.240002	202.833008	41.250599	0.292964	202.866730
41.260002	205.500000	41.270599	0.293735	202.714294
41.280003	205.500000	41.290600	0.296983	202.562469
41.299999	212.667007	41.310596	0.302740	202.411346
41.320000	209.833008	41.330597	0.311111	202.260742
41.340000	210.500000	41.350597	0.322270	202.110870
41.360001	196.667007	41.370598	0.336463	201.961609
41.380001	200.167007	41.390598	0.354023	201.812927
41.400002	209.000000	41.410599	0.375377	201.664825
41.420002	201.167007	41.430599	0.401069	201.517456
41.440002	190.500000	41.450600	0.431787	201.370636
41.460003	205.833008	41.470600	0.468390	201.224457
41.480003	211.500000	41.490601	0.511961	201.078888
41.500000	205.333008	41.510597	0.563855	200.933929
41.520000	196.667007	41.530598	0.625822	200.789520
41.540001	205.667007	41.550598	0.700061	200.645752
41.560001	209.500000	41.570599	0.789415	200.502594
41.580002	215.333008	41.590599	0.897570	200.360046

41.599998	221.000000	41.610596	1.029335	200.218018
41.619999	216.333008	41.630596	1.191175	200.076630
41.639999	220.333008	41.650597	1.394617	199.935852
41.660000	225.667007	41.670597	1.645604	199.795654
41.680000	231.500000	41.690598	1.963199	199.656006
41.700001	227.000000	41.710598	2.370205	199.516937
41.720001	226.833008	41.730598	2.899335	199.378418
41.740002	222.833008	41.750599	3.598671	199.240570
41.760002	219.833008	41.770599	4.540939	199.103180
41.780003	216.500000	41.790600	5.839925	198.966400
41.799999	217.167007	41.810596	7.679836	198.830231
41.820000	225.667007	41.830597	10.371392	198.694550
41.840000	222.333008	41.850597	14.449347	198.559448
41.860001	231.500000	41.870598	20.851622	198.424927
41.880001	226.167007	41.890598	31.224424	198.290924
41.900002	255.333008	41.910599	48.395897	198.157562
41.920002	275.166992	41.930599	76.947876	198.024628
41.940002	323.332977	41.950600	122.921791	197.892303
41.960003	398.500000	41.970600	189.178024	197.760437
41.980003	480.666992	41.990601	259.922882	197.629272
42.000000	504.666992	42.010597	288.542114	197.498535
42.020000	461.000000	42.030598	245.537003	197.368378
42.040001	381.832977	42.050598	169.837631	197.238739
42.060001	312.832977	42.070599	107.311386	197.109619
42.080002	261.666992	42.090599	67.660858	196.980988
42.099998	245.667007	42.110596	44.303810	196.852966
42.119999	228.000000	42.130592	30.451780	196.725311
42.139999	223.167007	42.150593	22.038120	196.598389
42.160000	218.167007	42.170593	16.938553	196.471771
42.180000	214.667007	42.190594	14.111901	196.345856
42.200001	219.167007	42.210594	13.152318	196.220306
42.220001	222.667007	42.230595	14.122435	196.095306
42.240002	214.500000	42.250595	17.528051	195.970825
42.260002	227.667007	42.270596	24.216286	195.846802
42.280003	250.000000	42.290596	34.603817	195.723267
42.299999	259.000000	42.310593	46.136356	195.600311
42.320000	261.332977	42.330593	51.076466	195.477844
42.340000	258.500000	42.350594	44.010765	195.355743

42.360001	232.833008	42.370594	30.971329	195.234253
42.380001	225.000000	42.390594	19.910883	195.113159
42.400002	200.333008	42.410595	12.759797	194.992584
42.420002	198.667007	42.430595	8.466688	194.872528
42.440002	186.333008	42.450596	5.854928	194.752899
42.460003	193.167007	42.470596	4.198183	194.633759
42.480003	198.000000	42.490597	3.101793	194.515106
42.500000	192.833008	42.510593	2.348780	194.396912
42.520000	203.333008	42.530594	1.821154	194.279144
42.540001	193.667007	42.550594	1.442080	194.161865
42.560001	187.833008	42.570595	1.164222	194.045013
42.580002	190.000000	42.590595	0.956585	193.928711
42.599998	188.333008	42.610592	0.798542	193.812775
42.619999	190.500000	42.630592	0.676085	193.697327
42.639999	199.500000	42.650593	0.579703	193.582336
42.660000	195.833008	42.670593	0.502764	193.467773
42.680000	190.500000	42.690594	0.440583	193.353699
42.700001	183.333008	42.710594	0.389791	193.239990
42.720001	180.833008	42.730595	0.347925	193.126709
42.740002	200.500000	42.750595	0.313157	193.013885
42.760002	190.000000	42.770596	0.284110	192.901520
42.780003	195.000000	42.790596	0.259734	192.789581
42.799999	186.000000	42.810593	0.239224	192.678131
42.820000	185.000000	42.830593	0.221721	192.567047
42.840000	187.000000	42.850594	0.207185	192.456329
42.860001	194.333008	42.870594	0.195000	192.346100
42.880001	188.333008	42.890594	0.184858	192.236176
42.900002	192.333008	42.910595	0.176516	192.126770
42.920002	186.667007	42.930595	0.169785	192.017792
42.940002	193.667007	42.950596	0.164517	191.909271
42.960003	194.833008	42.970596	0.160600	191.800995
42.980003	190.333008	42.990597	0.157954	191.693176
43.000000	195.167007	43.010593	0.156524	191.585846
43.020000	188.000000	43.030594	0.156280	191.478851
43.040001	194.000000	43.050594	0.157219	191.372253
43.060001	186.000000	43.070595	0.161473	191.265991
43.080002	189.167007	43.090595	0.164913	191.160187
43.099998	197.000000	43.110592	0.169659	191.054810

43.119999	180.667007	43.130592	0.175802	190.949768
43.139999	188.833008	43.150593	0.183463	190.845062
43.160000	179.500000	43.170593	0.192797	190.740814
43.180000	179.667007	43.190594	0.203999	190.636963
43.200001	188.500000	43.210594	0.217310	190.533325
43.220001	184.000000	43.230595	0.233031	190.430237
43.240002	189.500000	43.250595	0.251529	190.327423
43.260002	181.000000	43.270596	0.273261	190.225037
43.280003	179.833008	43.290596	0.298789	190.123047
43.299999	188.000000	43.310593	0.328808	190.021301
43.320000	181.333008	43.330593	0.364203	189.919952
43.340000	195.000000	43.350594	0.406064	189.819000
43.360001	192.833008	43.370594	0.455779	189.718384
43.380001	197.167007	43.390594	0.515114	189.618103
43.400002	202.833008	43.410595	0.586341	189.518158
43.420002	198.833008	43.430595	0.672409	189.418610
43.440002	191.167007	43.450596	0.777182	189.319366
43.460003	187.000000	43.470596	0.905789	189.220520
43.480003	193.667007	43.490597	1.065112	189.121948
43.500000	199.167007	43.510593	1.268945	189.023743
43.520000	200.833008	43.530594	1.521438	188.925903
43.540001	196.833008	43.550594	1.845043	188.828278
43.560001	198.333008	43.570595	2.265590	188.731110
43.580002	198.667007	43.590595	2.820712	188.634216
43.599998	192.667007	43.610592	3.566559	188.537720
43.619999	218.167007	43.630592	4.590533	188.441406
43.639999	201.833008	43.650589	6.032363	188.345551
43.660000	201.667007	43.670589	8.127106	188.249878
43.680000	210.833008	43.690590	11.279750	188.154663
43.700001	212.500000	43.710590	16.203388	188.059631
43.720001	220.833008	43.730591	24.153606	187.965027
43.740002	229.333008	43.750591	37.291340	187.870636
43.760002	256.666992	43.770592	59.098728	187.776611
43.780003	282.500000	43.790592	94.245537	187.682861
43.799999	344.332977	43.810589	145.568878	187.589417
43.820000	410.500000	43.830589	203.806320	187.496307
43.840000	430.666992	43.850590	236.428741	187.403442
43.860001	426.666992	43.870590	213.824738	187.310883

43.880001	368.332977	43.890591	155.733627	187.218567
43.900002	315.332977	43.910591	101.125008	187.126587
43.920002	269.166992	43.930592	64.129913	187.034973
43.940002	229.000000	43.950592	41.588432	186.943512
43.960003	222.833008	43.970592	27.918312	186.852386
43.980003	210.333008	43.990593	19.361860	186.761566
44.000000	206.167007	44.010590	13.816117	186.670990
44.020000	203.500000	44.030590	10.123808	186.580750
44.040001	190.833008	44.050591	7.624550	186.490723
44.060001	183.500000	44.070591	5.918535	186.400909
44.080002	175.667007	44.090591	4.753667	186.311462
44.099998	184.833008	44.110588	3.974291	186.222260
44.119999	184.333008	44.130589	3.480756	186.133331
44.139999	186.667007	44.150589	3.218300	186.044617
44.160000	191.500000	44.170589	3.162730	185.956238
44.180000	189.333008	44.190590	3.332638	185.868042
44.200001	184.000000	44.210590	3.777551	185.780151
44.220001	185.333008	44.230591	4.612759	185.692505
44.240002	203.667007	44.250591	6.058473	185.605011
44.260002	192.000000	44.270592	8.514011	185.517883
44.280003	194.333008	44.290592	12.672707	185.430939
44.299999	201.667007	44.310589	19.645117	185.344269
44.320000	206.667007	44.330589	30.895336	185.257843
44.340000	224.167007	44.350590	47.217403	185.171661
44.360001	229.500000	44.370590	65.459587	185.085724
44.380001	262.000000	44.390591	75.329689	185.000000
44.400002	236.500000	44.410591	67.920990	184.914490
44.420002	238.167007	44.430592	49.609203	184.829224
44.440002	216.000000	44.450592	32.416283	184.744232
44.460003	214.833008	44.470592	20.705568	184.659393
44.480003	208.833008	44.490593	13.519756	184.574829
44.500000	200.833008	44.510590	9.131800	184.490479
44.520000	190.333008	44.530590	6.366457	184.406342
44.540001	190.667007	44.550591	4.562453	184.322449
44.560001	194.667007	44.570591	3.353045	184.238770
44.580002	183.000000	44.590591	2.526103	184.155273
44.599998	191.667007	44.610588	1.951959	184.071991
44.619999	181.333008	44.630589	1.547650	183.988922

44.639999	179.167007	44.650589	1.259344	183.906067
44.660000	185.667007	44.670589	1.051544	183.823456
44.680000	171.667007	44.690590	0.900801	183.740997
44.700001	182.667007	44.710590	0.791555	183.658752
44.720001	190.667007	44.730591	0.713431	183.576721
44.740002	181.667007	44.750591	0.659494	183.494873
44.760002	186.667007	44.770592	0.625147	183.413208
44.780003	186.833008	44.790592	0.607439	183.331757
44.799999	175.167007	44.810589	0.604646	183.250549
44.820000	185.667007	44.830589	0.616022	183.169434
44.840000	177.500000	44.850590	0.641683	183.088593
44.860001	179.833008	44.870590	0.682575	183.007904
44.880001	177.833008	44.890591	0.740544	182.927368
44.900002	179.833008	44.910591	0.818495	182.847015
44.920002	178.000000	44.930592	0.920670	182.766937
44.940002	179.000000	44.950592	1.053083	182.686920
44.960003	176.667007	44.970592	1.224183	182.607117
44.980003	179.500000	44.990593	1.445862	182.527466
45.000000	180.000000	45.010590	1.734910	182.448029
45.020000	168.833008	45.030590	2.115637	182.368774
45.040001	168.833008	45.050591	2.623408	182.289734
45.060001	178.500000	45.070591	3.311451	182.210754
45.080002	187.667007	45.090591	4.262687	182.131958
45.099998	189.000000	45.110588	5.611542	182.053375
45.119999	175.500000	45.130589	7.586519	181.974976
45.139999	183.000000	45.150589	10.585210	181.896637
45.160000	192.000000	45.170589	15.310721	181.818542
45.180000	193.667007	45.190590	22.995802	181.740570
45.200001	208.500000	45.210587	35.721352	181.662750
45.220001	239.833008	45.230587	56.705185	181.585114
45.240002	275.332977	45.250587	89.775215	181.507568
45.260002	326.500000	45.270588	135.731781	181.430237
45.280003	381.832977	45.290588	182.804504	181.352966
45.299999	391.500000	45.310585	201.916794	181.275940
45.320000	350.832977	45.330585	175.530945	181.199036
45.340000	311.000000	45.350586	125.845924	181.122253
45.360001	258.166992	45.370586	82.042709	181.045593
45.380001	241.167007	45.390587	52.651012	180.969055

45.400002	204.167007	45.410587	34.528812	180.892700
45.420002	201.167007	45.430588	23.335705	180.816437
45.440002	195.667007	45.450588	16.206621	180.740387
45.460003	192.667007	45.470589	11.520055	180.664429
45.480003	189.333008	45.490589	8.364252	180.588593
45.500000	174.500000	45.510586	6.201750	180.512878
45.520000	168.167007	45.530586	4.696970	180.437286
45.540001	176.167007	45.550587	3.634192	180.361877
45.560001	181.333008	45.570587	2.871410	180.286560
45.580002	174.000000	45.590588	2.314795	180.211334
45.599998	176.833008	45.610584	1.904579	180.136292
45.619999	180.333008	45.630585	1.594329	180.061279
45.639999	175.167007	45.650585	1.358348	179.986481
45.660000	172.000000	45.670586	1.177318	179.911743
45.680000	166.167007	45.690586	1.037733	179.837128
45.700001	174.500000	45.710587	0.931729	179.762634
45.720001	169.833008	45.730587	0.848967	179.688171
45.740002	174.667007	45.750587	0.786086	179.613922
45.760002	170.333008	45.770588	0.739382	179.539764
45.780003	169.000000	45.790588	0.706125	179.465698
45.799999	173.000000	45.810585	0.684313	179.391693
45.820000	160.667007	45.830582	0.672486	179.317841
45.840000	170.667007	45.850582	0.669620	179.244110
45.860001	171.167007	45.870583	0.675035	179.170441
45.880001	182.500000	45.890583	0.688338	179.096893
45.900002	176.000000	45.910583	0.709380	179.023468
45.920002	173.833008	45.930584	0.738240	178.950073
45.940002	169.167007	45.950584	0.775212	178.876801
45.960003	168.833008	45.970585	0.820810	178.803650
45.980003	174.667007	45.990585	0.875782	178.730560
46.000000	176.333008	46.010582	0.941120	178.657562
46.020000	171.000000	46.030582	1.018156	178.584686
46.040001	171.167007	46.050583	1.108539	178.511871
46.060001	173.667007	46.070583	1.214370	178.439117
46.080002	178.667007	46.090584	1.335467	178.366486
46.099998	176.667007	46.110580	1.480864	178.293945
46.119999	172.500000	46.130581	1.651941	178.221405
46.139999	177.500000	46.150581	1.854102	178.149078

46.160000	173.500000	46.170582	2.094409	178.076752
46.180000	171.667007	46.190582	2.382246	178.004456
46.200001	176.333008	46.210583	2.730456	177.932404
46.220001	164.667007	46.230583	3.157254	177.860321
46.240002	172.167007	46.250584	3.689498	177.788300
46.260002	175.500000	46.270584	4.369356	177.716339
46.280003	178.833008	46.290585	5.257946	177.644440
46.299999	187.167007	46.310581	6.454018	177.572693
46.320000	188.833008	46.330582	8.099265	177.500946
46.340000	193.833008	46.350582	10.362000	177.429260
46.360001	193.000000	46.370583	13.306115	177.357727
46.380001	212.167007	46.390583	16.633804	177.286194
46.400002	206.667007	46.410583	19.630405	177.214722
46.420002	215.833008	46.430584	22.096783	177.143372
46.440002	213.500000	46.450584	25.050331	177.072021
46.460003	218.000000	46.470585	29.781403	177.000732
46.480003	230.000000	46.490585	37.261147	176.929535
46.500000	239.500000	46.510582	48.623585	176.858368
46.520000	241.333008	46.530582	65.857841	176.787292
46.540001	285.832977	46.550583	92.589455	176.716309
46.560001	322.332977	46.570583	135.332138	176.645325
46.580002	375.832977	46.590584	205.427200	176.574402
46.599998	471.166992	46.610580	321.498444	176.503448
46.619999	655.000000	46.630581	510.496185	176.432617
46.639999	941.500000	46.650581	798.606384	176.361877
46.660000	1315.500000	46.670582	1172.012085	176.291199
46.680000	1645.000000	46.690582	1502.196411	176.220520
46.700001	1711.000000	46.710583	1564.560303	176.149872
46.720001	1471.500000	46.730583	1299.597290	176.079285
46.740002	1080.000000	46.750584	914.686157	176.008759
46.760002	772.666992	46.770584	598.061890	175.938232
46.780003	558.000000	46.790585	388.290039	175.867798
46.799999	428.666992	46.810577	257.578064	175.797424
46.820000	364.666992	46.830578	175.358749	175.727020
46.840000	313.500000	46.850578	122.136787	175.656677
46.860001	291.332977	46.870579	86.736160	175.586365
46.880001	277.832977	46.890579	62.725140	175.516174
46.900002	277.666992	46.910580	46.201286	175.445923

46.920002	272.500000	46.930580	34.676952	175.375732
46.940002	265.332977	46.950581	26.519650	175.305603
46.960003	255.000000	46.970581	20.647469	175.235535
46.980003	241.000000	46.990582	16.342777	175.165436
47.000000	235.167007	47.010578	13.129780	175.095337
47.020000	217.333008	47.030579	10.689263	175.025391
47.040001	215.833008	47.050579	8.806979	174.955383
47.060001	197.500000	47.070580	7.335156	174.885498
47.080002	193.500000	47.090580	6.170249	174.815491
47.099998	193.833008	47.110577	5.238474	174.745605
47.119999	185.000000	47.130577	4.485759	174.675720
47.139999	179.167007	47.150578	3.872674	174.605927
47.160000	180.000000	47.170578	3.369591	174.536133
47.180000	178.167007	47.190578	2.954096	174.466309
47.200001	166.000000	47.210579	2.609061	174.396484
47.220001	169.833008	47.230579	2.321270	174.326721
47.240002	175.333008	47.250580	2.080443	174.257019
47.260002	174.333008	47.270580	1.878535	174.187317
47.280003	173.000000	47.290581	1.709213	174.117676
47.299999	167.333008	47.310577	1.567503	174.048035
47.320000	167.500000	47.330578	1.449401	173.978333
47.340000	165.833008	47.350578	1.351827	173.908691
47.360001	165.333008	47.370579	1.272353	173.839081
47.380001	158.000000	47.390579	1.209116	173.769531
47.400002	167.833008	47.410580	1.160738	173.699890
47.420002	157.667007	47.430580	1.126262	173.630310
47.440002	172.667007	47.450581	1.105117	173.560730
47.460003	167.167007	47.470581	1.097105	173.491272
47.480003	163.833008	47.490582	1.102392	173.421692
47.500000	169.000000	47.510578	1.121532	173.352173
47.520000	156.833008	47.530579	1.155519	173.282684
47.540001	176.167007	47.550579	1.205833	173.213226
47.560001	162.667007	47.570580	1.274547	173.143707
47.580002	159.333008	47.590580	1.362453	173.074219
47.599998	163.000000	47.610577	1.477333	173.004761
47.619999	166.000000	47.630577	1.622069	172.935272
47.639999	163.500000	47.650578	1.803113	172.865784
47.660000	172.000000	47.670578	2.029016	172.796387

47.680000	170.833008	47.690578	2.311148	172.726959
47.700001	170.500000	47.710579	2.664739	172.657501
47.720001	170.500000	47.730579	3.110380	172.588074
47.740002	175.333008	47.750580	3.676186	172.518646
47.760002	179.833008	47.770580	4.400950	172.449219
47.780003	180.167007	47.790581	5.338786	172.379791
47.799999	188.500000	47.810577	6.565965	172.310364
47.820000	176.167007	47.830578	8.193304	172.240906
47.840000	188.833008	47.850578	10.384400	172.171509
47.860001	198.833008	47.870579	13.392361	172.102112
47.880001	203.167007	47.890579	17.631256	172.032654
47.900002	209.000000	47.910580	23.789898	171.963226
47.920002	224.167007	47.930580	33.091137	171.893799
47.940002	247.500000	47.950581	47.692234	171.824402
47.960003	278.166992	47.970581	71.358276	171.754944
47.980003	321.666992	47.990582	110.378815	171.685547
48.000000	356.332977	48.010578	174.254181	171.616058
48.020000	458.000000	48.030579	274.150726	171.546631
48.040001	613.500000	48.050579	413.036530	171.477142
48.060001	765.166992	48.070580	561.126099	171.407745
48.080002	841.500000	48.090580	639.961426	171.338257
48.099998	769.166992	48.110577	586.659180	171.268860
48.119999	636.666992	48.130577	443.976746	171.199341
48.139999	490.000000	48.150578	300.763031	171.129883
48.160000	366.832977	48.170578	197.146896	171.060425
48.180000	297.832977	48.190578	130.566177	170.990875
48.200001	259.666992	48.210579	88.555351	170.921417
48.220001	217.000000	48.230579	61.505661	170.851929
48.240002	213.667007	48.250580	43.645493	170.782440
48.260002	213.333008	48.270580	31.644115	170.712830
48.280003	197.333008	48.290581	23.508257	170.643311
48.299999	199.333008	48.310577	17.983643	170.573761
48.320000	207.833008	48.330578	14.252869	170.504211
48.340000	199.833008	48.350578	11.787086	170.434631
48.360001	216.500000	48.370579	10.253590	170.365082
48.380001	202.000000	48.390579	9.464450	170.295532
48.400002	193.333008	48.410580	9.349846	170.225891
48.420002	179.333008	48.430580	9.958026	170.156281

48.440002	182.500000	48.450577	11.488975	170.086670
48.460003	185.167007	48.470577	14.377872	170.017029
48.480003	191.667007	48.490578	19.442305	169.947327
48.500000	202.833008	48.510574	28.104755	169.877686
48.520000	228.500000	48.530575	42.631081	169.807983
48.540001	248.667007	48.550575	66.000511	169.738342
48.560001	288.666992	48.570576	100.273071	169.668640
48.580002	335.666992	48.590576	141.387604	169.598938
48.599998	370.332977	48.610573	172.399231	169.529114
48.619999	377.332977	48.630573	171.402252	169.459412
48.639999	343.500000	48.650574	138.782104	169.389618
48.660000	277.500000	48.670574	97.686119	169.319824
48.680000	243.833008	48.690575	64.972816	169.250061
48.700001	214.833008	48.710575	43.209354	169.180237
48.720001	183.000000	48.730576	29.409250	169.110352
48.740002	176.833008	48.750576	20.574263	169.040527
48.760002	170.167007	48.770576	14.782063	168.970642
48.780003	180.833008	48.790577	10.910471	168.900818
48.799999	171.333008	48.810574	8.298711	168.830841
48.820000	165.167007	48.830574	6.533147	168.760925
48.840000	174.000000	48.850574	5.345751	168.690979
48.860001	164.333008	48.870575	4.558300	168.621094
48.880001	158.333008	48.890575	4.052875	168.551117
48.900002	163.167007	48.910576	3.752762	168.481079
48.920002	162.333008	48.930576	3.609466	168.411041
48.940002	165.667007	48.950577	3.593835	168.341003
48.960003	158.500000	48.970577	3.690252	168.270996
48.980003	163.833008	48.990578	3.893081	168.200867
49.000000	164.000000	49.010574	4.204728	168.130859
49.020000	166.833008	49.030575	4.635247	168.060791
49.040001	177.667007	49.050575	5.202341	167.990601
49.060001	165.167007	49.070576	5.932894	167.920471
49.080002	175.833008	49.090576	6.865275	167.850281
49.099998	167.833008	49.110573	8.052785	167.780121
49.119999	174.667007	49.130573	9.570122	167.709900
49.139999	176.667007	49.150574	11.520134	167.639709
49.160000	188.833008	49.170574	14.046596	167.569458
49.180000	191.333008	49.190575	17.352118	167.499207

49.200001	204.333008	49.210575	21.727194	167.428894
49.220001	197.833008	49.230576	27.600338	167.358582
49.240002	222.167007	49.250576	35.630436	167.288269
49.260002	234.000000	49.270576	46.882652	167.217896
49.280003	249.167007	49.290577	63.162762	167.147583
49.299999	275.832977	49.310574	87.629089	167.077209
49.320000	310.332977	49.330574	125.878937	167.006836
49.340000	346.832977	49.350574	187.655090	166.936371
49.360001	449.500000	49.370575	289.198486	166.865906
49.380001	591.333008	49.390575	455.090515	166.795471
49.400002	858.666992	49.410576	714.697083	166.724915
49.420002	1263.000000	49.430576	1079.532715	166.654388
49.440002	1622.000000	49.450577	1484.674805	166.583893
49.460003	1868.169922	49.470577	1739.436768	166.513367
49.480003	1796.669922	49.490578	1656.927124	166.442749
49.500000	1447.000000	49.510574	1301.272949	166.372192
49.520000	1077.169922	49.530575	903.770996	166.301605
49.540001	784.333008	49.550575	599.575256	166.230957
49.560001	550.833008	49.570576	398.484894	166.160339
49.580002	425.666992	49.590576	269.902710	166.089691
49.599998	347.166992	49.610573	186.516678	166.018982
49.619999	301.000000	49.630573	131.089417	165.948242
49.639999	283.832977	49.650574	93.544617	165.877563
49.660000	246.667007	49.670574	67.799126	165.806824
49.680000	227.333008	49.690575	49.985619	165.736023
49.700001	226.000000	49.710575	37.542316	165.665222
49.720001	206.333008	49.730576	28.743097	165.594421
49.740002	208.167007	49.750576	22.424360	165.523560
49.760002	200.333008	49.770576	17.808315	165.452728
49.780003	197.333008	49.790577	14.377017	165.381866
49.799999	198.000000	49.810574	11.785534	165.311035
49.820000	189.167007	49.830574	9.800427	165.240112
49.840000	187.333008	49.850574	8.263336	165.169189
49.860001	182.833008	49.870575	7.063645	165.098206
49.880001	183.000000	49.890575	6.123044	165.027222
49.900002	181.333008	49.910576	5.385311	164.956268
49.920002	172.167007	49.930576	4.805398	164.885254
49.940002	170.000000	49.950577	4.362644	164.814240

49.960003	170.500000	49.970577	4.031483	164.743225
49.980003	174.833008	49.990578	3.797265	164.672119
50.000000	175.500000	50.010574	3.650601	164.601135
50.020000	169.333008	50.030575	3.586540	164.529968
50.040001	173.333008	50.050575	3.604420	164.458862
50.060001	169.167007	50.070576	3.707804	164.387726
50.080002	166.833008	50.090576	3.904954	164.316589
50.099998	166.667007	50.110573	4.209670	164.245422
50.119999	161.000000	50.130573	4.643007	164.174255
50.139999	179.833008	50.150574	5.235184	164.103027
50.160000	169.833008	50.170570	6.028922	164.031860
50.180000	175.333008	50.190571	7.084900	163.960632
50.200001	172.667007	50.210571	8.489878	163.889404
50.220001	166.167007	50.230572	10.361605	163.818115
50.240002	185.500000	50.250572	12.878414	163.746826
50.260002	187.167007	50.270573	16.307226	163.675568
50.280003	190.167007	50.290573	21.071745	163.604248
50.299999	196.667007	50.310570	27.878082	163.532898
50.320000	216.333008	50.330570	37.957474	163.461609
50.340000	229.833008	50.350571	53.480022	163.390198
50.360001	248.333008	50.370571	78.254150	163.318848
50.380001	284.166992	50.390572	118.735031	163.247467
50.400002	338.500000	50.410572	185.085129	163.176086
50.420002	446.500000	50.430573	290.890472	163.104675
50.440002	603.333008	50.450573	447.094299	163.033264
50.460003	793.666992	50.470573	641.896484	162.961792
50.480003	963.500000	50.490574	810.056152	162.890350
50.500000	1011.669983	50.510571	848.501892	162.818909
50.520000	888.666992	50.530571	726.633240	162.747406
50.540001	727.333008	50.550571	533.027222	162.675934
50.560001	528.166992	50.570572	361.605896	162.604431
50.580002	398.166992	50.590572	241.144958	162.532898
50.599998	326.332977	50.610569	162.806473	162.461426
50.619999	267.166992	50.630569	112.035927	162.389832
50.639999	234.167007	50.650570	78.454247	162.318298
50.660000	221.500000	50.670570	55.784180	162.246735
50.680000	210.667007	50.690571	40.277855	162.175171
50.700001	197.500000	50.710571	29.590420	162.103607

50.720001	187.333008	50.730572	22.180389	162.032043
50.740002	171.833008	50.750572	17.006105	161.960449
50.760002	175.500000	50.770573	13.360255	161.888794
50.780003	178.000000	50.790573	10.766622	161.817169
50.799999	159.667007	50.810570	8.908716	161.745605
50.820000	163.167007	50.830570	7.577303	161.673950
50.840000	160.000000	50.850571	6.636674	161.602295
50.860001	158.833008	50.870571	5.999173	161.530640
50.880001	158.333008	50.890572	5.610339	161.459045
50.900002	155.333008	50.910572	5.439822	161.387329
50.920002	151.000000	50.930573	5.476356	161.315674
50.940002	172.167007	50.950573	5.725926	161.244019
50.960003	162.833008	50.970573	6.212271	161.172333
50.980003	159.333008	50.990574	6.978593	161.100616
51.000000	150.167007	51.010571	8.098276	161.028931
51.020000	155.667007	51.030571	9.679677	160.957214
51.040001	169.500000	51.050571	11.891117	160.885529
51.060001	176.500000	51.070572	15.001471	160.813873
51.080002	176.333008	51.090572	19.459301	160.742126
51.099998	184.667007	51.110569	26.040581	160.670410
51.119999	201.667007	51.130569	36.121418	160.598694
51.139999	211.500000	51.150570	52.117664	160.527008
51.160000	242.667007	51.170570	78.155952	160.455261
51.180000	306.166992	51.190571	120.841370	160.383514
51.200001	373.000000	51.210571	189.401260	160.311829
51.220001	474.166992	51.230572	292.751434	160.240112
51.240002	627.000000	51.250572	428.252319	160.168335
51.260002	758.666992	51.270573	560.617737	160.096619
51.280003	810.500000	51.290573	619.179626	160.024902
51.299999	753.500000	51.310570	560.700012	159.953186
51.320000	616.166992	51.330570	428.810852	159.881470
51.340000	483.332977	51.350571	297.221252	159.809753
51.360001	363.500000	51.370571	199.555405	159.738037
51.380001	289.166992	51.390572	134.726151	159.666321
51.400002	250.833008	51.410572	92.551308	159.594604
51.420002	225.500000	51.430573	64.680588	159.522858
51.440002	194.333008	51.450573	45.880409	159.451141
51.460003	207.000000	51.470573	33.018604	159.379456

51.480003	189.333008	51.490574	24.152479	159.307709
51.500000	179.333008	51.510571	18.013016	159.236023
51.520000	174.667007	51.530571	13.738378	159.164307
51.540001	159.833008	51.550571	10.742315	159.092590
51.560001	160.000000	51.570572	8.625875	159.020996
51.580002	164.833008	51.590572	7.121851	158.949219
51.599998	162.667007	51.610569	6.053853	158.877625
51.619999	156.833008	51.630569	5.306196	158.805908
51.639999	155.833008	51.650570	4.804930	158.734253
51.660000	164.667007	51.670570	4.503859	158.662598
51.680000	159.667007	51.690571	4.376714	158.590973
51.700001	159.000000	51.710571	4.412656	158.519287
51.720001	152.000000	51.730572	4.614268	158.447693
51.740002	158.500000	51.750572	4.997483	158.376068
51.760002	170.333008	51.770573	5.593237	158.304382
51.780003	163.667007	51.790573	6.451053	158.232849
51.799999	159.667007	51.810570	7.645201	158.161224
51.820000	174.000000	51.830570	9.287070	158.089630
51.840000	167.667007	51.850571	11.545025	158.018066
51.860001	175.667007	51.870571	14.686011	157.946533
51.880001	172.500000	51.890572	19.153915	157.874969
51.900002	193.000000	51.910572	25.716757	157.803467
51.920002	196.500000	51.930569	35.726746	157.731964
51.940002	214.167007	51.950569	51.563774	157.660400
51.960003	231.167007	51.970570	77.267952	157.588928
51.980003	279.332977	51.990570	119.274406	157.517456
52.000000	361.832977	52.010567	186.507385	157.445984
52.020000	465.666992	52.030567	287.629456	157.374573
52.040001	596.666992	52.050568	420.499023	157.303131
52.060001	758.000000	52.070568	552.835571	157.231689
52.080002	806.166992	52.090569	618.834167	157.160278
52.099998	779.333008	52.110565	573.806946	157.088867
52.119999	650.333008	52.130566	451.121063	157.017578
52.139999	519.833008	52.150566	319.530365	156.946228
52.160000	376.832977	52.170567	216.982956	156.874878
52.180000	314.500000	52.190567	147.019775	156.803528
52.200001	254.833008	52.210567	100.976212	156.732239
52.220001	227.500000	52.230568	70.431107	156.660950

52.240002	207.167007	52.250568	49.786373	156.589722
52.260002	191.000000	52.270569	35.628517	156.518433
52.280003	182.500000	52.290569	25.837584	156.447235
52.299999	175.333008	52.310566	19.031536	156.376068
52.320000	161.500000	52.330566	14.270107	156.304901
52.340000	161.500000	52.350567	10.909598	156.233734
52.360001	160.000000	52.370567	8.507619	156.162628
52.380001	155.500000	52.390568	6.764246	156.091492
52.400002	159.667007	52.410568	5.478221	156.020447
52.420002	156.333008	52.430569	4.515038	155.949341
52.440002	155.000000	52.450569	3.784445	155.878326
52.460003	146.000000	52.470570	3.225205	155.807373
52.480003	148.833008	52.490570	2.795117	155.736328
52.500000	155.833008	52.510567	2.464697	155.665375
52.520000	154.000000	52.530567	2.212892	155.594421
52.540001	151.667007	52.550568	2.024869	155.523560
52.560001	150.833008	52.570568	1.890085	155.452637
52.580002	155.167007	52.590569	1.801274	155.381775
52.599998	151.167007	52.610565	1.753755	155.311035
52.619999	158.000000	52.630566	1.741995	155.240234
52.639999	142.333008	52.650566	1.771389	155.169464
52.660000	153.667007	52.670567	1.840115	155.098694
52.680000	146.500000	52.690567	1.951325	155.027954
52.700001	158.333008	52.710567	2.110362	154.957336
52.720001	155.667007	52.730568	2.325207	154.886658
52.740002	151.833008	52.750568	2.607166	154.816071
52.760002	150.167007	52.770569	2.971898	154.745483
52.780003	161.667007	52.790569	3.440868	154.674927
52.799999	161.167007	52.810566	4.043310	154.604462
52.820000	173.833008	52.830566	4.819694	154.533936
52.840000	168.000000	52.850567	5.825421	154.463470
52.860001	180.833008	52.870567	7.137959	154.393066
52.880001	177.833008	52.890568	8.867643	154.322693
52.900002	187.333008	52.910568	11.176690	154.252380
52.920002	207.833008	52.930569	14.313809	154.182068
52.940002	215.000000	52.950569	18.678471	154.111755
52.960003	241.500000	52.970570	24.938038	154.041504
52.980003	251.500000	52.990570	34.231934	153.971283

53.000000	263.832977	53.010567	48.495007	153.901154
53.020000	286.500000	53.030567	70.917091	153.830994
53.040001	321.500000	53.050568	106.356735	153.760895
53.060001	354.000000	53.070568	161.360580	153.690857
53.080002	432.666992	53.090569	243.289932	153.620911
53.099998	542.166992	53.110565	358.328674	153.550903
53.119999	676.333008	53.130566	505.127502	153.480896
53.139999	809.500000	53.150566	655.521606	153.411011
53.160000	843.500000	53.170567	740.904358	153.341187
53.180000	807.333008	53.190567	701.045410	153.271362
53.200001	689.000000	53.210567	561.074219	153.201599
53.220001	565.833008	53.230568	401.837860	153.131866
53.240002	435.000000	53.250568	274.699951	153.062134
53.260002	336.832977	53.270569	186.996796	152.992523
53.280003	285.500000	53.290569	128.878510	152.922913
53.299999	248.833008	53.310566	90.099304	152.853333
53.320000	211.833008	53.330566	63.733238	152.783813
53.340000	198.500000	53.350567	45.555138	152.714355
53.360001	196.167007	53.370567	32.926903	152.644928
53.380001	184.000000	53.390568	24.117817	152.575531
53.400002	175.333008	53.410568	17.944353	152.506226
53.420002	169.167007	53.430569	13.583911	152.436890
53.440002	174.833008	53.450569	10.466712	152.367676
53.460003	166.333008	53.470570	8.203068	152.298462
53.480003	164.667007	53.490570	6.529800	152.229279
53.500000	167.333008	53.510567	5.270571	152.160217
53.520000	163.500000	53.530567	4.306279	152.091187
53.540001	168.500000	53.550568	3.556633	152.022156
53.560001	173.500000	53.570564	2.966099	151.953186
53.580002	164.833008	53.590565	2.495257	151.884308
53.599998	158.833008	53.610561	2.116113	151.815430
53.619999	176.167007	53.630562	1.807887	151.746643
53.639999	170.333008	53.650562	1.555297	151.677856
53.660000	169.833008	53.670563	1.346758	151.609161
53.680000	157.333008	53.690563	1.173429	151.540466
53.700001	153.167007	53.710564	1.028494	151.471924
53.720001	149.167007	53.730564	0.906655	151.403320
53.740002	150.167007	53.750565	0.803759	151.334808

53.760002	154.333008	53.770565	0.716528	151.266388
53.780003	143.167007	53.790562	0.642377	151.197998
53.799999	160.167007	53.810558	0.579221	151.129669
53.820000	160.333008	53.830559	0.524205	151.061401
53.840000	145.333008	53.850559	0.478454	150.993195
53.860001	153.833008	53.870560	0.439699	150.924988
53.880001	140.000000	53.890560	0.407128	150.856873
53.900002	142.167007	53.910561	0.378604	150.788757
53.920002	150.167007	53.930561	0.356786	150.720734
53.940002	147.333008	53.950562	0.339835	150.652832
53.960003	146.167007	53.970562	0.327646	150.584930
53.980003	129.333008	53.990562	0.320291	150.517090
54.000000	142.000000	54.010559	0.318038	150.449310
54.020000	152.167007	54.030560	0.321392	150.381592
54.040001	139.333008	54.050560	0.331159	150.313934
54.060001	139.667007	54.070560	0.348522	150.246338
54.080002	139.167007	54.090561	0.375175	150.178772
54.099998	147.000000	54.110558	0.413480	150.111267
54.119999	140.500000	54.130558	0.466751	150.043884
54.139999	135.667007	54.150558	0.539585	149.976501
54.160000	137.667007	54.170559	0.638520	149.909241
54.180000	141.500000	54.190559	0.773165	149.841949
54.200001	147.833008	54.210560	0.958375	149.774780
54.220001	146.000000	54.230560	1.218453	149.707642
54.240002	140.333008	54.250561	1.595052	149.640564
54.260002	140.667007	54.270561	2.161304	149.573547
54.280003	153.833008	54.290562	3.045448	149.506653
54.299999	151.167007	54.310558	4.466094	149.439819
54.320000	151.333008	54.330559	6.777164	149.372955
54.340000	162.500000	54.350559	10.490735	149.306213
54.360001	162.500000	54.370560	16.188236	149.239502
54.380001	174.167007	54.390560	24.094692	149.172913
54.400002	186.500000	54.410561	33.092625	149.106384
54.420002	192.000000	54.430561	39.826160	149.039856
54.440002	185.167007	54.450562	40.318909	148.973480
54.460003	187.000000	54.470562	34.262260	148.907104
54.480003	171.833008	54.490562	25.548651	148.840820
54.500000	162.000000	54.510559	17.823900	148.774567

54.520000	152.833008	54.530560	12.228324	148.708435
54.540001	159.167007	54.550560	8.453776	148.642334
54.560001	154.167007	54.570560	5.923069	148.576324
54.580002	149.500000	54.590561	4.199630	148.510376
54.599998	149.167007	54.610558	3.008256	148.444489
54.619999	136.667007	54.630558	2.177502	148.378693
54.639999	148.000000	54.650558	1.596475	148.312927
54.660000	142.833008	54.670559	1.189260	148.247223
54.680000	139.833008	54.690559	0.902642	148.181641
54.700001	144.333008	54.710560	0.699235	148.116089
54.720001	138.833008	54.730560	0.553087	148.050629
54.740002	146.333008	54.750561	0.446465	147.985168
54.760002	141.500000	54.770561	0.367403	147.919861
54.780003	138.167007	54.790562	0.307864	147.854614
54.799999	134.500000	54.810558	0.262438	147.789459
54.820000	140.833008	54.830559	0.227412	147.724304
54.840000	129.333008	54.850559	0.200230	147.659241
54.860001	143.333008	54.870560	0.179077	147.594238
54.880001	145.833008	54.890560	0.162649	147.529388
54.900002	135.333008	54.910561	0.148197	147.464539
54.920002	145.333008	54.930561	0.138658	147.399780
54.940002	146.833008	54.950562	0.131668	147.335144
54.960003	144.333008	54.970562	0.126845	147.270508
54.980003	150.333008	54.990562	0.123910	147.205963
55.000000	139.833008	55.010559	0.122664	147.141479
55.020000	147.500000	55.030560	0.122972	147.077087
55.040001	141.000000	55.050560	0.124755	147.012787
55.060001	146.833008	55.070560	0.127981	146.948547
55.080002	138.000000	55.090561	0.132658	146.884338
55.099998	153.333008	55.110558	0.138834	146.820282
55.119999	147.500000	55.130558	0.146601	146.756287
55.139999	140.000000	55.150558	0.156089	146.692322
55.160000	133.833008	55.170559	0.167477	146.628479
55.180000	140.667007	55.190559	0.180994	146.564667
55.200001	141.167007	55.210560	0.196932	146.500977
55.220001	134.667007	55.230560	0.215654	146.437256
55.240002	135.167007	55.250561	0.237613	146.373718
55.260002	137.833008	55.270561	0.263369	146.310272

55.280003	141.167007	55.290562	0.293618	146.246857
55.299999	130.667007	55.310558	0.329219	146.183563
55.320000	136.167007	55.330559	0.371267	146.120270
55.340000	140.000000	55.350559	0.421117	146.057129
55.360001	132.333008	55.370560	0.480487	145.993988
55.380001	134.000000	55.390560	0.551569	145.930969
55.400002	137.500000	55.410561	0.637394	145.868103
55.420002	137.500000	55.430561	0.741154	145.805176
55.440002	133.500000	55.450562	0.867803	145.742432
55.460003	136.500000	55.470562	1.023564	145.679688
55.480003	133.167007	55.490562	1.216671	145.617096
55.500000	132.167007	55.510559	1.458038	145.554535
55.520000	144.833008	55.530560	1.762388	145.492126
55.540001	134.500000	55.550560	2.149302	145.429688
55.560001	142.167007	55.570560	2.645644	145.367401
55.580002	138.833008	55.590561	3.287723	145.305176
55.599998	136.333008	55.610558	4.126547	145.243073
55.619999	144.000000	55.630558	5.237426	145.180969
55.639999	143.667007	55.650558	6.736546	145.119019
55.660000	145.667007	55.670559	8.815215	145.057037
55.680000	144.833008	55.690559	11.802378	144.995209
55.700001	152.333008	55.710556	16.273975	144.933533
55.720001	156.000000	55.730556	23.236938	144.871887
55.740002	171.333008	55.750557	34.389435	144.810303
55.760002	186.167007	55.770557	52.431644	144.748779
55.780003	200.333008	55.790558	81.189186	144.687408
55.799999	257.166992	55.810555	124.822472	144.626068
55.820000	310.166992	55.830555	184.617172	144.564789
55.840000	374.166992	55.850555	251.901657	144.503601
55.860001	433.332977	55.870556	302.256653	144.442566
55.880001	448.332977	55.890556	307.249878	144.381531
55.900002	407.832977	55.910557	263.987213	144.320648
55.920002	337.166992	55.930557	199.588242	144.259796
55.940002	269.666992	55.950558	140.954514	144.198975
55.960003	240.167007	55.970558	97.595238	144.138306
55.980003	205.333008	55.990559	67.896873	144.077728
56.000000	177.000000	56.010555	47.769684	144.017242
56.020000	170.000000	56.030556	33.943314	143.956818

56.040001	160.833008	56.050556	24.317005	143.896484
56.060001	160.333008	56.070557	17.569323	143.836243
56.080002	149.833008	56.090557	12.835449	143.776031
56.099998	152.500000	56.110554	9.521091	143.715973
56.119999	153.333008	56.130554	7.205345	143.655975
56.139999	150.833008	56.150555	5.593569	143.596039
56.160000	141.500000	56.170555	4.483098	143.536255
56.180000	141.833008	56.190556	3.750191	143.476410
56.200001	143.667007	56.210556	3.326661	143.416779
56.220001	141.500000	56.230556	3.200490	143.357147
56.240002	141.000000	56.250557	3.411488	143.297638
56.260002	142.500000	56.270557	4.042606	143.238251
56.280003	134.500000	56.290558	5.178331	143.178894
56.299999	146.833008	56.310555	6.781552	143.119751
56.320000	142.833008	56.330555	8.480913	143.060516
56.340000	142.833008	56.350555	9.494247	143.001465
56.360001	145.667007	56.370556	9.142469	142.942474
56.380001	138.833008	56.390556	7.607955	142.883606
56.400002	145.667007	56.410557	5.727366	142.824707
56.420002	139.000000	56.430557	4.131518	142.765961
56.440002	139.500000	56.450558	2.977490	142.707336
56.460003	134.500000	56.470558	2.186166	142.648804
56.480003	130.167007	56.490559	1.645162	142.590271
56.500000	135.833008	56.510555	1.271706	142.531921
56.520000	140.833008	56.530556	1.013023	142.473572
56.540001	136.000000	56.550556	0.835955	142.415344
56.560001	133.167007	56.570557	0.718632	142.357178
56.580002	133.000000	56.590557	0.646185	142.299133
56.599998	131.500000	56.610554	0.608557	142.241211
56.619999	134.000000	56.630554	0.599339	142.183289
56.639999	145.500000	56.650555	0.615143	142.125488
56.660000	137.333008	56.670555	0.655241	142.067749
56.680000	134.833008	56.690556	0.721606	142.010162
56.700001	133.500000	56.710556	0.819466	141.952606
56.720001	136.167007	56.730556	0.958650	141.895142
56.740002	139.333008	56.750557	1.156087	141.837769
56.760002	133.500000	56.770557	1.439819	141.780426
56.780003	136.500000	56.790558	1.854581	141.723236

56.799999	132.333008	56.810555	2.467328	141.666138
56.820000	143.167007	56.830555	3.367566	141.609131
56.840000	144.833008	56.850555	4.644324	141.552185
56.860001	145.000000	56.870556	6.313533	141.495300
56.880001	143.000000	56.890556	8.192778	141.438538
56.900002	142.833008	56.910557	9.874458	141.381836
56.920002	144.500000	56.930557	11.076499	141.325256
56.940002	144.833008	56.950558	12.161430	141.268707
56.960003	153.833008	56.970558	14.090740	141.212280
56.980003	148.333008	56.990559	18.005966	141.155945
57.000000	166.167007	57.010555	25.198900	141.099670
57.020000	177.333008	57.030556	37.363396	141.043518
57.040001	197.000000	57.050556	56.516342	140.987396
57.060001	229.333008	57.070557	83.934151	140.931396
57.080002	276.000000	57.090557	117.352196	140.875427
57.099998	305.332977	57.110554	147.414612	140.819641
57.119999	327.166992	57.130554	159.537384	140.763885
57.139999	309.332977	57.150555	146.263977	140.708191
57.160000	271.166992	57.170555	116.311058	140.652618
57.180000	227.667007	57.190556	84.584129	140.597137
57.200001	194.333008	57.210556	59.312916	140.541718
57.220001	179.167007	57.230556	41.447102	140.486389
57.240002	176.500000	57.250557	29.214911	140.431122
57.260002	160.000000	57.270557	20.787876	140.375977
57.280003	146.000000	57.290558	14.899619	140.320831
57.299999	146.000000	57.310555	10.747865	140.265930
57.320000	154.500000	57.330555	7.811467	140.210999
57.340000	146.667007	57.350555	5.736666	140.156219
57.360001	150.833008	57.370556	4.271387	140.101440
57.380001	149.667007	57.390556	3.234026	140.046814
57.400002	144.667007	57.410557	2.494699	139.992188
57.420002	142.333008	57.430557	1.962125	139.937683
57.440002	136.667007	57.450558	1.573355	139.883301
57.460003	137.667007	57.470558	1.285627	139.828979
57.480003	143.333008	57.490559	1.070117	139.774719
57.500000	138.333008	57.510555	0.907426	139.720551
57.520000	143.667007	57.530556	0.784326	139.666504
57.540001	128.500000	57.550556	0.691866	139.612579

57.560001	136.500000	57.570557	0.623901	139.558624
57.580002	138.000000	57.590557	0.576287	139.504822
57.599998	142.667007	57.610554	0.546753	139.451050
57.619999	141.667007	57.630554	0.533054	139.397461
57.639999	131.500000	57.650555	0.535124	139.343842
57.660000	128.333008	57.670555	0.553502	139.290344
57.680000	130.500000	57.690556	0.589762	139.236938
57.700001	130.500000	57.710556	0.646657	139.183624
57.720001	133.667007	57.730556	0.728367	139.130371
57.740002	134.333008	57.750553	0.840868	139.077209
57.760002	128.333008	57.770554	0.992696	139.024170
57.780003	135.667007	57.790554	1.196004	138.971130
57.799999	133.500000	57.810551	1.468859	138.918274
57.820000	128.333008	57.830551	1.839863	138.865387
57.840000	131.000000	57.850552	2.356264	138.812592
57.860001	137.833008	57.870552	3.099520	138.759979
57.880001	136.500000	57.890553	4.211722	138.707336
57.900002	134.333008	57.910553	5.937731	138.654846
57.920002	154.667007	57.930553	8.684610	138.602448
57.940002	153.667007	57.950554	13.086051	138.550049
57.960003	160.000000	57.970554	20.016117	138.497772
57.980003	165.667007	57.990555	30.392479	138.445648
58.000000	186.833008	58.010551	44.452118	138.393494
58.020000	206.333008	58.030552	60.314468	138.341492
58.040001	211.333008	58.050552	72.866913	138.289581
58.060001	221.500000	58.070553	76.122444	138.237671
58.080002	201.000000	58.090553	68.978966	138.185883
58.099998	193.333008	58.110550	56.388428	138.134186
58.119999	185.667007	58.130550	43.657021	138.082520
58.139999	166.667007	58.150551	33.041996	138.030975
58.160000	170.167007	58.170551	24.990822	137.979523
58.180000	163.500000	58.190552	19.516321	137.928131
58.200001	144.500000	58.210552	16.489264	137.876801
58.220001	151.333008	58.230553	15.755164	137.825562
58.240002	154.000000	58.250553	17.167137	137.774353
58.260002	164.833008	58.270554	20.314562	137.723328
58.280003	170.167007	58.290554	23.997969	137.672302
58.299999	179.167007	58.310551	26.104797	137.621368

58.320000	162.000000	58.330551	24.890293	137.570465
58.340000	165.833008	58.350552	20.739990	137.519745
58.360001	160.833008	58.370552	15.678638	137.468994
58.380001	155.000000	58.390553	11.316555	137.418365
58.400002	142.500000	58.410553	8.110737	137.367859
58.420002	151.500000	58.430553	5.888546	137.317383
58.440002	136.000000	58.450554	4.363335	137.266998
58.460003	133.167007	58.470554	3.315045	137.216644
58.480003	136.333008	58.490555	2.602189	137.166382
58.500000	133.833008	58.510551	2.136888	137.116241
58.520000	131.667007	58.530552	1.864644	137.066162
58.540001	130.167007	58.550552	1.755317	137.016113
58.560001	124.667000	58.570553	1.801206	136.966217
58.580002	131.500000	58.590553	2.022084	136.916290
58.599998	131.667007	58.610550	2.476776	136.866486
58.619999	135.833008	58.630550	3.283125	136.816803
58.639999	130.500000	58.650551	4.644173	136.767151
58.660000	137.500000	58.670551	6.871522	136.717529
58.680000	138.500000	58.690552	10.374456	136.668091
58.700001	151.167007	58.710552	15.511662	136.618591
58.720001	156.167007	58.730553	22.174479	136.569275
58.740002	157.333008	58.750553	29.087368	136.519958
58.760002	164.667007	58.770554	33.576630	136.470764
58.780003	165.000000	58.790554	33.185596	136.421600
58.799999	162.333008	58.810551	28.231260	136.372528
58.820000	159.167007	58.830551	21.502436	136.323547
58.840000	149.667007	58.850552	15.456150	136.274597
58.860001	151.000000	58.870552	10.926340	136.225708
58.880001	143.000000	58.890553	7.753680	136.176941
58.900002	135.000000	58.910553	5.551750	136.128174
58.920002	138.333008	58.930553	4.006261	136.079529
58.940002	135.000000	58.950554	2.909445	136.030945
58.960003	133.500000	58.970554	2.127591	135.982452
58.980003	129.667007	58.990555	1.570826	135.934052
59.000000	134.500000	59.010551	1.175609	135.885681
59.020000	133.000000	59.030552	0.895480	135.837341
59.040001	130.000000	59.050552	0.696717	135.789124
59.060001	134.000000	59.070553	0.554985	135.740936

59.080002	128.500000	59.090553	0.453125	135.692841
59.099998	131.500000	59.110550	0.379289	135.644806
59.119999	130.833008	59.130550	0.325376	135.596863
59.139999	137.167007	59.150551	0.285944	135.548920
59.160000	133.333008	59.170551	0.257312	135.501129
59.180000	131.500000	59.190552	0.236984	135.453400
59.200001	134.167007	59.210552	0.223258	135.405670
59.220001	128.333008	59.230553	0.214980	135.358032
59.240002	135.667007	59.250553	0.211377	135.310425
59.260002	133.333008	59.270554	0.211957	135.262878
59.280003	139.833008	59.290554	0.216450	135.215485
59.299999	143.667007	59.310551	0.224765	135.168121
59.320000	130.833008	59.330551	0.236977	135.120819
59.340000	132.000000	59.350552	0.253315	135.073547
59.360001	135.333008	59.370552	0.274169	135.026367
59.380001	140.333008	59.390553	0.300109	134.979248
59.400002	138.167007	59.410553	0.331914	134.932190
59.420002	141.000000	59.430553	0.370610	134.885162
59.440002	145.667007	59.450554	0.417540	134.838257
59.460003	145.833008	59.470554	0.474445	134.791412
59.480003	154.667007	59.490555	0.543561	134.744568
59.500000	150.333008	59.510551	0.627845	134.697815
59.520000	150.833008	59.530552	0.731133	134.651123
59.540001	159.333008	59.550552	0.858419	134.604492
59.560001	150.667007	59.570553	1.016277	134.557922
59.580002	153.833008	59.590553	1.213349	134.511444
59.599998	154.500000	59.610550	1.460950	134.465027
59.619999	154.500000	59.630550	1.774159	134.418610
59.639999	153.167007	59.650551	2.172825	134.372284
59.660000	149.833008	59.670551	2.683829	134.326050
59.680000	144.167007	59.690552	3.344809	134.279816
59.700001	143.667007	59.710552	4.211399	134.233673
59.720001	149.333008	59.730553	5.371276	134.187561
59.740002	156.000000	59.750553	6.970682	134.141510
59.760002	150.000000	59.770554	9.261642	134.095520
59.780003	157.000000	59.790554	12.684793	134.049622
59.799999	157.000000	59.810551	17.991228	134.003754
59.820000	170.000000	59.830551	26.419249	133.957947

59.840000	172.833008	59.850552	39.869247	133.912231
59.860001	191.500000	59.870552	60.922791	133.866516
59.880001	238.500000	59.890549	92.242142	133.820892
59.900002	273.832977	59.910549	134.543182	133.775269
59.920002	315.666992	59.930550	182.492111	133.729706
59.940002	354.000000	59.950550	221.343094	133.684265
59.960003	358.000000	59.970551	232.487091	133.638855
59.980003	347.000000	59.990551	209.889267	133.593475
60.000000	303.666992	60.010548	167.242188	133.548187
60.020000	278.332977	60.030548	123.440834	133.502930
60.040001	225.833008	60.050549	88.406921	133.457703
60.060001	204.000000	60.070549	63.231853	133.412537
60.080002	179.167007	60.090549	45.690479	133.367462
60.099998	173.500000	60.110546	33.453098	133.322388
60.119999	154.000000	60.130547	24.870302	133.277374
60.139999	158.167007	60.150547	18.888748	133.232452
60.160000	146.500000	60.170547	14.824605	133.187592
60.180000	153.000000	60.190548	12.223569	133.142670
60.200001	147.833008	60.210548	10.793695	133.097870
60.220001	141.500000	60.230549	10.387510	133.053162
60.240002	153.000000	60.250549	11.018485	133.008484
60.260002	146.000000	60.270550	12.910056	132.963745
60.280003	154.167007	60.290550	16.580877	132.919189
60.299999	165.333008	60.310547	22.959648	132.874603
60.320000	169.167007	60.330547	33.492149	132.830139
60.340000	194.167007	60.350548	50.063801	132.785645
60.360001	219.667007	60.370544	74.367157	132.741211
60.380001	252.333008	60.390545	106.103958	132.696899
60.400002	288.332977	60.410545	139.897049	132.652588
60.420002	316.000000	60.430546	163.899933	132.608337
60.440002	303.332977	60.450546	165.963089	132.564087
60.460003	281.332977	60.470547	145.255447	132.519897
60.480003	251.000000	60.490547	113.499954	132.475769
60.500000	217.833008	60.510544	83.140724	132.431702
60.520000	197.833008	60.530544	59.546909	132.387634
60.540001	167.667007	60.550545	42.716713	132.343597
60.560001	163.333008	60.570545	30.973076	132.299652
60.580002	154.667007	60.590546	22.782635	132.255768

60.599998	159.000000	60.610542	17.113485	132.211884
60.619999	138.833008	60.630543	13.311901	132.168060
60.639999	143.167007	60.650543	10.929849	132.124268
60.660000	133.333008	60.670544	9.560495	132.080505
60.680000	130.500000	60.690544	8.722689	132.036865
60.700001	125.167000	60.710545	7.896613	131.993225
60.720001	128.833008	60.730545	6.777638	131.949585
60.740002	130.167007	60.750546	5.454216	131.906006
60.760002	122.833000	60.770546	4.204405	131.862457
60.780003	134.000000	60.790546	3.200228	131.818939
60.799999	136.500000	60.810543	2.455436	131.775482
60.820000	128.667007	60.830544	1.915522	131.732117
60.840000	128.833008	60.850544	1.522275	131.688660
60.860001	134.500000	60.870544	1.232775	131.645325
60.880001	138.000000	60.890545	1.018344	131.602020
60.900002	134.500000	60.910545	0.859695	131.558777
60.920002	137.167007	60.930546	0.743309	131.515533
60.940002	136.500000	60.950546	0.659383	131.472351
60.960003	136.167007	60.970547	0.600651	131.429230
60.980003	130.000000	60.990547	0.562061	131.386108
61.000000	127.333000	61.010544	0.539899	131.343018
61.020000	127.167000	61.030544	0.531768	131.299957
61.040001	123.667000	61.050545	0.536689	131.257019
61.060001	132.500000	61.070545	0.553330	131.214020
61.080002	136.333008	61.090546	0.582249	131.171082
61.099998	130.500000	61.110542	0.624280	131.128174
61.119999	135.500000	61.130543	0.680974	131.085297
61.139999	127.500000	61.150543	0.754626	131.042419
61.160000	128.333008	61.170544	0.848457	130.999664
61.180000	129.667007	61.190544	0.966823	130.956879
61.200001	123.333000	61.210545	1.115534	130.914185
61.220001	129.500000	61.230545	1.302266	130.871460
61.240002	128.500000	61.250546	1.537120	130.828766
61.260002	126.833000	61.270546	1.833361	130.786163
61.280003	133.333008	61.290546	2.208536	130.743530
61.299999	135.167007	61.310543	2.685663	130.700958
61.320000	131.000000	61.330544	3.296737	130.658417
61.340000	135.000000	61.350544	4.086782	130.615845

61.360001	132.333008	61.370544	5.123432	130.573364
61.380001	133.500000	61.390545	6.513950	130.530945
61.400002	141.667007	61.410545	8.436091	130.488525
61.420002	142.833008	61.430546	11.191119	130.446136
61.440002	146.500000	61.450546	15.288857	130.403748
61.460003	151.833008	61.470547	21.571993	130.361450
61.480003	172.500000	61.490547	31.365261	130.319092
61.500000	179.667007	61.510544	46.574997	130.276855
61.520000	208.333008	61.530544	69.543396	130.234558
61.540001	234.667007	61.550545	102.184868	130.192352
61.560001	287.500000	61.570545	144.062027	130.150116
61.580002	333.332977	61.590546	190.182495	130.107941
61.599998	374.832977	61.610542	231.741959	130.065796
61.619999	416.500000	61.630543	261.235931	130.023682
61.639999	422.000000	61.650543	274.593658	129.981598
61.660000	421.666992	61.670544	267.316986	129.939514
61.680000	397.332977	61.690544	236.963547	129.897461
61.700001	338.832977	61.710545	191.151627	129.855438
61.720001	290.166992	61.730545	143.851242	129.813416
61.740002	253.833008	61.750546	104.541031	129.771423
61.760002	220.333008	61.770546	75.294312	129.729492
61.780003	198.667007	61.790546	54.386017	129.687592
61.799999	176.167007	61.810543	39.490379	129.645660
61.820000	164.333008	61.830544	28.791969	129.603790
61.840000	156.167007	61.850544	21.065210	129.561951
61.860001	152.333008	61.870544	15.478975	129.520111
61.880001	151.000000	61.890545	11.449857	129.478302
61.900002	149.333008	61.910545	8.551694	129.436462
61.920002	143.833008	61.930546	6.468322	129.394684
61.940002	132.333008	61.950546	4.966075	129.352936
61.960003	138.000000	61.970547	3.874903	129.311188
61.980003	138.333008	61.990547	3.073415	129.269470
62.000000	126.833000	62.010544	2.476583	129.227783
62.020000	131.833008	62.030544	2.025245	129.186127
62.040001	125.167000	62.050545	1.678957	129.144470
62.060001	129.667007	62.070545	1.409729	129.102814
62.080002	130.667007	62.090546	1.198077	129.061218
62.099998	123.667000	62.110542	1.030277	129.019623

62.119999	127.333000	62.130543	0.896470	128.978058
62.139999	128.167007	62.150543	0.789326	128.936462
62.160000	131.833008	62.170544	0.703578	128.894897
62.180000	132.500000	62.190540	0.635240	128.853394
62.200001	131.000000	62.210541	0.581270	128.811859
62.220001	131.500000	62.230541	0.539410	128.770355
62.240002	128.333008	62.250542	0.507957	128.728882
62.260002	126.167000	62.270542	0.485661	128.687439
62.280003	133.000000	62.290543	0.471639	128.645996
62.299999	131.333008	62.310539	0.465319	128.604553
62.320000	128.500000	62.330540	0.466516	128.563141
62.340000	130.000000	62.350540	0.474957	128.521698
62.360001	130.333008	62.370541	0.490956	128.480316
62.380001	123.000000	62.390541	0.514974	128.438965
62.400002	126.833000	62.410542	0.547751	128.397583
62.420002	127.333000	62.430542	0.590350	128.356232
62.440002	121.667000	62.450542	0.644216	128.314911
62.460003	116.167000	62.470543	0.711265	128.273590
62.480003	125.333000	62.490543	0.794001	128.232300
62.500000	127.333000	62.510540	0.895657	128.190979
62.520000	129.000000	62.530540	1.020496	128.149689
62.540001	135.833008	62.550541	1.173987	128.108398
62.560001	126.333000	62.570541	1.363261	128.067169
62.580002	124.500000	62.590542	1.597596	128.025909
62.599998	131.333008	62.610538	1.888995	127.984650
62.619999	135.333008	62.630539	2.253231	127.943481
62.639999	128.500000	62.650539	2.710568	127.902222
62.660000	131.500000	62.670540	3.287382	127.861023
62.680000	137.167007	62.690540	4.018463	127.819824
62.700001	134.667007	62.710541	4.950687	127.778625
62.720001	130.667007	62.730541	6.150526	127.737488
62.740002	138.500000	62.750542	7.718376	127.696320
62.760002	148.167007	62.770542	9.816055	127.655121
62.780003	156.833008	62.790543	12.717239	127.613983
62.799999	152.500000	62.810539	16.893940	127.572876
62.820000	161.833008	62.830540	23.161522	127.531708
62.840000	168.667007	62.850540	32.883663	127.490601
62.860001	182.500000	62.870541	48.240711	127.449463

62.880001	203.333008	62.890541	72.460320	127.408356
62.900002	248.333008	62.910542	109.703705	127.367279
62.920002	295.332977	62.930542	163.896011	127.326172
62.940002	385.500000	62.950542	235.329742	127.285095
62.960003	457.666992	62.970543	314.689117	127.243988
62.980003	528.500000	62.990543	378.904205	127.202911
63.000000	559.166992	63.010540	399.806915	127.161865
63.020000	528.166992	63.030540	367.173737	127.120789
63.040001	470.500000	63.050541	299.776917	127.079742
63.060001	379.666992	63.070541	226.729568	127.038666
63.080002	331.500000	63.090542	165.648010	126.997650
63.099998	266.500000	63.110538	120.207108	126.956604
63.119999	242.667007	63.130539	87.653015	126.915588
63.139999	213.167007	63.150539	64.357681	126.874542
63.160000	184.500000	63.170540	47.554996	126.833527
63.180000	176.667007	63.190540	35.407681	126.792511
63.200001	168.500000	63.210541	26.700256	126.751434
63.220001	161.500000	63.230541	20.599188	126.710480
63.240002	156.000000	63.250542	16.528528	126.669464
63.260002	151.333008	63.270542	14.123311	126.628479
63.280003	154.833008	63.290543	13.225788	126.587433
63.299999	163.667007	63.310539	13.902223	126.546448
63.320000	157.833008	63.330540	16.461494	126.505463
63.340000	157.667007	63.350540	21.419188	126.464478
63.360001	173.833008	63.370541	29.307190	126.423462
63.380001	177.833008	63.390541	40.143406	126.382477
63.400002	196.667007	63.410542	52.497883	126.341461
63.420002	210.667007	63.430542	62.792667	126.300507
63.440002	211.333008	63.450542	66.509293	126.259491
63.460003	204.000000	63.470543	61.733486	126.218506
63.480003	194.167007	63.490543	51.157494	126.177582
63.500000	178.000000	63.510540	39.408558	126.136566
63.520000	172.333008	63.530540	29.470501	126.095581
63.540001	160.500000	63.550541	22.079052	126.054626
63.560001	157.333008	63.570541	16.855597	126.013641
63.580002	153.167007	63.590542	13.230403	125.972687
63.599998	139.833008	63.610538	10.764630	125.931702
63.619999	134.167007	63.630539	9.172526	125.890747

63.639999	144.500000	63.650539	8.278185	125.849701
63.660000	132.667007	63.670540	7.975852	125.808807
63.680000	135.167007	63.690540	8.213211	125.767761
63.700001	144.667007	63.710541	8.991273	125.726807
63.720001	141.833008	63.730541	10.380175	125.685822
63.740002	142.333008	63.750542	12.555017	125.644836
63.760002	154.167007	63.770542	15.861188	125.603851
63.780003	160.333008	63.790543	20.921923	125.562897
63.799999	159.167007	63.810539	28.796770	125.521912
63.820000	184.667007	63.830540	41.193218	125.480927
63.840000	198.833008	63.850540	60.641083	125.439972
63.860001	224.333008	63.870541	90.438988	125.398956
63.880001	281.832977	63.890541	133.832718	125.357971
63.900002	338.000000	63.910542	191.574356	125.316986
63.920002	402.332977	63.930542	257.470001	125.276001
63.940002	469.832977	63.950542	314.738922	125.234985
63.960003	493.166992	63.970543	341.141968	125.194000
63.980003	463.666992	63.990543	326.072449	125.152985
64.000000	420.666992	64.010536	282.192596	125.112030
64.019997	391.500000	64.030533	234.162628	125.071045
64.040001	348.166992	64.050537	200.525772	125.029999
64.059998	332.166992	64.070534	189.028244	124.988983
64.080002	336.000000	64.090538	200.972275	124.947998
64.099998	369.332977	64.110535	233.717926	124.906982
64.119995	401.332977	64.130531	278.363342	124.865967
64.139999	443.166992	64.150536	316.924164	124.824921
64.159996	446.832977	64.170532	327.624481	124.783905
64.180000	414.832977	64.190536	300.754913	124.742889
64.199997	384.666992	64.210533	247.779587	124.701874
64.219994	337.000000	64.230530	189.533386	124.660858
64.239998	274.500000	64.250534	139.854980	124.619781
64.259995	243.667007	64.270531	102.309265	124.578735
64.279999	209.000000	64.290535	75.081451	124.537720
64.300003	186.667007	64.310539	55.393368	124.496643
64.320000	173.167007	64.330536	41.025314	124.455597
64.340004	168.167007	64.350540	30.457211	124.414520
64.360001	158.167007	64.370537	22.684362	124.373474
64.380005	148.167007	64.390541	16.986485	124.332397

64.400002	140.000000	64.410538	12.838193	124.291321
64.419998	138.667007	64.430534	9.833157	124.250275
64.440002	146.833008	64.450539	7.662806	124.209198
64.459999	134.000000	64.470535	6.097497	124.168121
64.480003	138.667007	64.490540	4.967130	124.127014
64.500000	138.500000	64.510536	4.151261	124.085938
64.519997	131.500000	64.530533	3.565496	124.044861
64.540001	127.167000	64.550537	3.152284	124.003754
64.559998	131.833008	64.570534	2.873829	123.962646
64.580002	124.833000	64.590538	2.706530	123.921539
64.599998	136.833008	64.610535	2.636568	123.880432
64.619995	127.667000	64.630531	2.658053	123.839294
64.639999	136.667007	64.650536	2.771795	123.798187
64.659996	137.500000	64.670532	2.984469	123.757050
64.680000	131.000000	64.690536	3.309130	123.715912
64.699997	135.500000	64.710533	3.765350	123.674774
64.719994	133.000000	64.730530	4.381931	123.633636
64.739998	132.500000	64.750534	5.200927	123.592468
64.759995	141.167007	64.770531	6.284764	123.551361
64.779999	140.333008	64.790535	7.735004	123.510162
64.800003	150.833008	64.810539	9.720074	123.468994
64.820000	140.833008	64.830536	12.528915	123.427795
64.840004	150.333008	64.850540	16.663342	123.386627
64.860001	153.333008	64.870537	22.963585	123.345490
64.880005	167.667007	64.890541	32.805561	123.304291
64.900002	184.000000	64.910538	48.277256	123.263123
64.919998	209.167007	64.930534	72.283989	123.221924
64.940002	242.167007	64.950539	108.176170	123.180695
64.959999	289.166992	64.970535	158.186340	123.139526
64.980003	378.666992	64.990540	220.153275	123.098297
65.000000	420.832977	65.010536	282.886505	123.057068
65.019997	457.666992	65.030533	325.899597	123.015869
65.040001	484.500000	65.050537	330.174652	122.974640
65.059998	443.000000	65.070534	294.825531	122.933411
65.080002	394.832977	65.090538	238.446899	122.892181
65.099998	327.500000	65.110535	182.296310	122.850952
65.119995	283.500000	65.130531	137.403259	122.809692
65.139999	247.500000	65.150536	105.564331	122.768433

65.159996	235.667007	65.170532	84.992271	122.727203
65.180000	214.000000	65.190536	73.610016	122.685944
65.199997	204.000000	65.210533	69.787308	122.644684
65.219994	207.667007	65.230530	71.579964	122.603424
65.239998	215.667007	65.250534	75.710106	122.562134
65.259995	203.167007	65.270531	77.528572	122.520874
65.279999	213.167007	65.290535	73.250854	122.479614
65.300003	199.833008	65.310539	62.862846	122.438293
65.320000	176.500000	65.330536	49.807495	122.397003
65.340004	164.500000	65.350540	37.634991	122.355743
65.360001	156.167007	65.370537	27.954580	122.314423
65.380005	148.000000	65.390541	20.767738	122.273102
65.400002	144.000000	65.410538	15.523582	122.231812
65.419998	139.333008	65.430534	11.670243	122.190491
65.440002	135.833008	65.450539	8.809646	122.149170
65.459999	130.167007	65.470535	6.676570	122.107849
65.480003	126.667000	65.490540	5.084966	122.066528
65.500000	125.167000	65.510536	3.901877	122.025208
65.519997	127.000000	65.530533	3.024783	121.983887
65.540001	114.000000	65.550537	2.374950	121.942535
65.559998	128.500000	65.570534	1.892668	121.901215
65.580002	123.333000	65.590538	1.532193	121.859833
65.599998	121.500000	65.610535	1.260360	121.818542
65.619995	118.333000	65.630531	1.052848	121.777191
65.639999	117.000000	65.650536	0.892403	121.735809
65.659996	117.667000	65.670532	0.767016	121.694489
65.680000	131.000000	65.690536	0.668055	121.653107
65.699997	117.333000	65.710533	0.589577	121.611755
65.719994	126.333000	65.730530	0.527264	121.570374
65.739998	119.833000	65.750534	0.478042	121.528992
65.759995	120.000000	65.770531	0.439753	121.487640
65.779999	119.833000	65.790535	0.410806	121.446289
65.800003	116.167000	65.810539	0.390139	121.404907
65.820000	117.333000	65.830536	0.377071	121.363556
65.840004	124.833000	65.850540	0.371241	121.322144
65.860001	118.167000	65.870537	0.372615	121.280792
65.880005	118.667000	65.890541	0.381464	121.239380
65.900002	118.167000	65.910538	0.398383	121.197998

65.919998	122.833000	65.930534	0.424349	121.156616
65.940002	127.167000	65.950539	0.460814	121.115204
65.959999	114.667000	65.970535	0.509663	121.073853
65.980003	120.167000	65.990540	0.573569	121.032440
66.000000	118.000000	66.010529	0.655878	120.991089
66.019997	120.833000	66.030525	0.761119	120.949677
66.040001	116.333000	66.050529	0.895048	120.908295
66.059998	119.333000	66.070526	1.064965	120.866943
66.080002	124.833000	66.090530	1.280744	120.825531
66.099998	120.500000	66.110527	1.555568	120.784119
66.119995	120.667000	66.130524	1.908899	120.742737
66.139999	127.667000	66.150528	2.371091	120.701355
66.159996	123.833000	66.170525	2.991431	120.659943
66.180000	136.833008	66.190529	3.854862	120.618561
66.199997	130.500000	66.210526	5.105236	120.577179
66.219994	131.833008	66.230522	6.986166	120.535767
66.239998	139.000000	66.250526	9.894241	120.494385
66.259995	137.833008	66.270523	14.437419	120.453003
66.279999	147.833008	66.290527	21.477798	120.411621
66.300003	166.667007	66.310532	32.051033	120.370209
66.320000	175.667007	66.330528	47.016022	120.328857
66.340004	199.500000	66.350533	66.242844	120.287476
66.360001	235.833008	66.370529	87.317863	120.246063
66.380005	249.500000	66.390533	104.932693	120.204681
66.400002	253.333008	66.410530	112.629982	120.163330
66.419998	236.000000	66.430527	107.223770	120.121979
66.440002	233.167007	66.450531	91.581314	120.080566
66.459999	205.333008	66.470528	72.206833	120.039246
66.480003	180.833008	66.490532	54.404499	119.997833
66.500000	170.333008	66.510529	40.305199	119.956482
66.519997	154.833008	66.530525	29.810505	119.915131
66.540001	149.000000	66.550529	22.105370	119.873779
66.559998	134.500000	66.570526	16.420755	119.832428
66.580002	125.500000	66.590530	12.193751	119.791107
66.599998	122.500000	66.610527	9.047948	119.749756
66.619995	123.167000	66.630524	6.714904	119.708405
66.639999	125.000000	66.650528	4.995883	119.667084
66.659996	119.333000	66.670525	3.739004	119.625732

66.680000	120.000000	66.690529	2.823673	119.584442
66.699997	118.833000	66.710526	2.158276	119.543121
66.719994	121.833000	66.730522	1.672688	119.501831
66.739998	114.500000	66.750526	1.315404	119.460510
66.759995	107.333000	66.770523	1.049669	119.419250
66.779999	117.500000	66.790527	0.849081	119.377930
66.800003	113.667000	66.810532	0.695447	119.336639
66.820000	117.667000	66.830528	0.576077	119.295349
66.840004	109.667000	66.850533	0.482007	119.254089
66.860001	117.500000	66.870529	0.407073	119.212830
66.880005	108.833000	66.890533	0.346767	119.171570
66.900002	112.500000	66.910530	0.297918	119.130341
66.919998	114.833000	66.930527	0.258133	119.089081
66.940002	109.333000	66.950531	0.225644	119.047852
66.959999	108.833000	66.970528	0.199161	119.006622
66.980003	113.667000	66.990532	0.177681	118.965393
67.000000	108.500000	67.010529	0.160522	118.924225
67.019997	109.167000	67.030525	0.147136	118.883057
67.040001	108.333000	67.050529	0.137249	118.841858
67.059998	114.333000	67.070526	0.130808	118.800690
67.080002	121.667000	67.090530	0.127993	118.759521
67.099998	120.667000	67.110527	0.129686	118.718384
67.119995	115.167000	67.130524	0.137322	118.677216
67.139999	105.667000	67.150528	0.153666	118.636078
67.159996	118.000000	67.170525	0.183386	118.594971
67.180000	123.167000	67.190529	0.233495	118.553864
67.199997	110.333000	67.210526	0.315679	118.512787
67.219994	111.833000	67.230522	0.443382	118.471680
67.239998	115.667000	67.250526	0.630265	118.430634
67.259995	120.000000	67.270523	0.880118	118.389587
67.279999	117.167000	67.290527	1.170628	118.348511
67.300003	119.667000	67.310532	1.437312	118.307495
67.320000	110.167000	67.330528	1.585740	118.266479
67.340004	112.667000	67.350533	1.551559	118.225464
67.360001	122.333000	67.370529	1.355859	118.184479
67.380005	127.000000	67.390533	1.087792	118.143494
67.400002	116.667000	67.410530	0.831217	118.102539
67.419998	115.667000	67.430527	0.623884	118.061615

67.440002	117.833000	67.450531	0.468047	118.020660
67.459999	126.000000	67.470528	0.353104	117.979736
67.480003	120.167000	67.490532	0.267818	117.938843
67.500000	130.500000	67.510529	0.204074	117.897980
67.519997	115.833000	67.530525	0.156292	117.857117
67.540001	111.833000	67.550529	0.120609	117.816254
67.559998	112.833000	67.570526	0.094199	117.775452
67.580002	114.167000	67.590530	0.074832	117.734650
67.599998	110.667000	67.610527	0.060783	117.693817
67.619995	107.167000	67.630524	0.050682	117.653076
67.639999	118.333000	67.650528	0.043499	117.612305
67.659996	110.833000	67.670525	0.038448	117.571594
67.680000	116.667000	67.690529	0.034977	117.530853
67.699997	117.667000	67.710526	0.032700	117.490173
67.719994	114.167000	67.730522	0.031350	117.449493
67.739998	109.167000	67.750526	0.030753	117.408844
67.759995	112.667000	67.770523	0.030803	117.368195
67.779999	114.167000	67.790527	0.031442	117.327545
67.800003	105.833000	67.810532	0.032652	117.286957
67.820000	110.167000	67.830528	0.034443	117.246368
67.840004	107.333000	67.850533	0.036856	117.205841
67.860001	111.833000	67.870529	0.039958	117.165314
67.880005	111.333000	67.890533	0.043850	117.124817
67.900002	116.667000	67.910530	0.048666	117.084290
67.919998	113.500000	67.930527	0.054585	117.043823
67.940002	115.167000	67.950531	0.061843	117.003418
67.959999	110.667000	67.970528	0.070735	116.962982
67.980003	109.333000	67.990532	0.081577	116.922577
68.000000	111.333000	68.010529	0.095009	116.882263
68.019997	107.000000	68.030525	0.111580	116.841919
68.040001	115.333000	68.050529	0.132089	116.801575
68.059998	114.000000	68.070526	0.157515	116.761292
68.080002	105.500000	68.090530	0.189135	116.721008
68.099998	113.833000	68.110527	0.228552	116.680817
68.119995	113.833000	68.130524	0.277972	116.640625
68.139999	108.667000	68.150528	0.340518	116.600433
68.159996	107.000000	68.170525	0.420948	116.560242
68.180000	111.500000	68.190529	0.527151	116.520142

68.199997	109.500000	68.210526	0.672293	116.480042
68.219994	108.167000	68.230522	0.879221	116.440002
68.239998	109.833000	68.250526	1.185892	116.399902
68.259995	112.167000	68.270523	1.654167	116.359924
68.279999	112.333000	68.290527	2.382614	116.319916
68.300003	110.833000	68.310532	3.511278	116.279968
68.320000	114.667000	68.330528	5.219682	116.240051
68.340004	115.500000	68.350533	7.689425	116.200165
68.360001	115.833000	68.370529	10.987150	116.160278
68.380005	120.000000	68.390533	14.867453	116.120453
68.400002	126.000000	68.410530	18.557581	116.080658
68.419998	121.833000	68.430527	20.864727	116.040894
68.440002	131.333008	68.450531	20.839375	116.001190
68.459999	124.500000	68.470528	18.571005	115.961426
68.480003	120.667000	68.490532	15.123233	115.921814
68.500000	122.500000	68.510529	11.652617	115.882172
68.519997	115.500000	68.530525	8.767244	115.842590
68.540001	112.167000	68.550529	6.567976	115.803009
68.559998	106.500000	68.570526	4.937336	115.763489
68.580002	109.833000	68.590530	3.725918	115.723969
68.599998	116.000000	68.610527	2.820581	115.684570
68.619995	115.667000	68.630524	2.142966	115.645111
68.639999	112.000000	68.650528	1.639401	115.605743
68.659996	107.000000	68.670525	1.271294	115.566406
68.680000	107.000000	68.690529	1.008485	115.527069
68.699997	108.667000	68.710526	0.827903	115.487793
68.719994	114.500000	68.730522	0.711682	115.448578
68.739998	104.167000	68.750526	0.647131	115.409363
68.759995	109.000000	68.770523	0.626646	115.370209
68.779999	115.333000	68.790527	0.648029	115.331085
68.800003	111.833000	68.810532	0.715853	115.291992
68.820000	110.167000	68.830528	0.843849	115.252991
68.840004	118.833000	68.850533	1.059283	115.213989
68.860001	113.333000	68.870529	1.408384	115.175018
68.880005	118.167000	68.890533	1.963796	115.136078
68.900002	120.333000	68.910530	2.827528	115.097198
68.919998	112.833000	68.930527	4.124103	115.058411
68.940002	109.500000	68.950531	5.964090	115.019562

68.959999	116.167000	68.970528	8.350322	114.980865
68.980003	118.167000	68.990532	11.039845	114.942139
69.000000	112.500000	69.010529	13.436838	114.903473
69.019997	117.333000	69.030525	14.761651	114.864868
69.040001	118.333000	69.050529	14.551933	114.826294
69.059998	118.667000	69.070526	13.068163	114.787750
69.080002	118.333000	69.090530	11.047961	114.749237
69.099998	109.167000	69.110527	9.101723	114.710846
69.119995	111.833000	69.130524	7.435218	114.672424
69.139999	110.333000	69.150528	6.011317	114.634094
69.159996	106.500000	69.170525	4.781035	114.595795
69.180000	107.167000	69.190529	3.748696	114.557526
69.199997	112.000000	69.210526	2.930054	114.519318
69.219994	109.167000	69.230522	2.314065	114.481201
69.239998	116.500000	69.250526	1.870135	114.443054
69.259995	113.000000	69.270523	1.564950	114.404999
69.279999	115.167000	69.290527	1.370471	114.367004
69.300003	108.667000	69.310532	1.266741	114.329041
69.320000	109.667000	69.330528	1.240476	114.291138
69.340004	112.000000	69.350533	1.284043	114.253235
69.360001	103.667000	69.370529	1.394818	114.215454
69.380005	110.167000	69.390533	1.575383	114.177673
69.400002	114.333000	69.410530	1.834479	114.139954
69.419998	109.500000	69.430527	2.190182	114.102295
69.440002	112.333000	69.450523	2.675953	114.064697
69.459999	109.000000	69.470520	3.350820	114.027161
69.480003	112.167000	69.490524	4.318267	113.989624
69.500000	111.500000	69.510521	5.752616	113.952209
69.519997	123.000000	69.530518	7.941070	113.914795
69.540001	117.500000	69.550522	11.333267	113.877441
69.559998	120.667000	69.570518	16.579834	113.840149
69.580002	135.500000	69.590523	24.538454	113.802917
69.599998	142.667007	69.610519	36.106438	113.765747
69.619995	158.500000	69.630516	51.785751	113.728638
69.639999	174.500000	69.650520	70.796181	113.691589
69.659996	186.833008	69.670517	90.004089	113.654541
69.680000	205.000000	69.690521	103.951508	113.617615
69.699997	205.833008	69.710518	107.309395	113.580750

69.719994	215.667007	69.730515	98.922165	113.543945
69.739998	194.500000	69.750519	82.890694	113.507141
69.759995	174.333008	69.770515	65.140877	113.470459
69.779999	154.500000	69.790520	49.575642	113.433716
69.800003	142.500000	69.810524	37.386070	113.397156
69.820000	138.667007	69.830521	28.238710	113.360596
69.840004	131.500000	69.850525	21.407141	113.324097
69.860001	119.833000	69.870522	16.280727	113.287720
69.880005	122.333000	69.890526	12.426655	113.251343
69.900002	121.500000	69.910522	9.562376	113.215088
69.919998	111.667000	69.930519	7.493708	113.178833
69.940002	106.833000	69.950523	6.092080	113.142639
69.959999	116.167000	69.970520	5.284347	113.106506
69.980003	107.667000	69.990524	5.047434	113.070496
70.000000	114.833000	70.010521	5.405589	113.034546
70.019997	106.333000	70.030518	6.405775	112.998596
70.040001	113.000000	70.050522	8.056707	112.962708
70.059998	117.833000	70.070518	10.210245	112.926941
70.080002	120.333000	70.090523	12.435377	112.891174
70.099998	120.167000	70.110519	14.017598	112.855530
70.119995	120.167000	70.130516	14.285746	112.819946
70.139999	122.667000	70.150520	13.101174	112.784424
70.159996	111.833000	70.170517	10.983687	112.748962
70.180000	117.833000	70.190521	8.668861	112.713562
70.199997	116.667000	70.210518	6.643584	112.678223
70.219994	114.667000	70.230515	5.052163	112.642944
70.239998	111.000000	70.250519	3.850383	112.607788
70.259995	112.167000	70.270515	2.947460	112.572632
70.279999	106.833000	70.290520	2.263847	112.537598
70.300003	106.500000	70.310524	1.744444	112.502563
70.320000	111.833000	70.330521	1.351591	112.467712
70.340004	99.666695	70.350525	1.058242	112.432800
70.360001	109.000000	70.370522	0.844888	112.398071
70.380005	112.167000	70.390526	0.696816	112.363342
70.400002	112.333000	70.410522	0.604300	112.328735
70.419998	113.167000	70.430519	0.561655	112.294128
70.440002	109.500000	70.450523	0.567410	112.259644
70.459999	108.500000	70.470520	0.622012	112.225220

70.480003	102.000000	70.490524	0.723913	112.190857
70.500000	105.167000	70.510521	0.862036	112.156616
70.519997	102.667000	70.530518	1.008902	112.122375
70.540001	113.667000	70.550522	1.122395	112.088257
70.559998	108.833000	70.570518	1.167991	112.054199
70.580002	113.500000	70.590523	1.147959	112.020203
70.599998	117.500000	70.610519	1.106603	111.986328
70.619995	109.500000	70.630516	1.104998	111.952515
70.639999	106.333000	70.650520	1.198109	111.918701
70.659996	104.667000	70.670517	1.434205	111.885071
70.680000	111.833000	70.690521	1.864894	111.851440
70.699997	107.333000	70.710518	2.544730	111.817932
70.719994	110.167000	70.730515	3.510503	111.784485
70.739998	106.000000	70.750519	4.730409	111.751099
70.759995	113.833000	70.770515	6.033621	111.717773
70.779999	107.500000	70.790520	7.088865	111.684570
70.800003	108.667000	70.810524	7.519634	111.651489
70.820000	108.667000	70.830521	7.158860	111.618408
70.840004	109.167000	70.850525	6.192502	111.585388
70.860001	107.667000	70.870522	5.004881	111.552490
70.880005	106.500000	70.890526	3.905576	111.519653
70.900002	109.167000	70.910522	3.023651	111.486938
70.919998	104.167000	70.930519	2.359676	111.454285
70.940002	112.833000	70.950523	1.870100	111.421692
70.959999	106.333000	70.970520	1.512000	111.389221
70.980003	102.333000	70.990524	1.253802	111.356750
71.000000	104.167000	71.010521	1.075189	111.324463
71.019997	101.833000	71.030518	0.962925	111.292175
71.040001	106.000000	71.050522	0.909204	111.260010
71.059998	101.333000	71.070518	0.911238	111.227905
71.080002	110.333000	71.090523	0.972236	111.195923
71.099998	110.000000	71.110519	1.103645	111.163940
71.119995	112.333000	71.130516	1.328555	111.132141
71.139999	107.333000	71.150520	1.685695	111.100403
71.159996	113.000000	71.170517	2.231862	111.068726
71.180000	111.833000	71.190521	3.041251	111.037109
71.199997	112.833000	71.210518	4.188882	111.005615
71.219994	120.667000	71.230515	5.712437	110.974243

71.239998	115.000000	71.250519	7.539130	110.942871
71.259995	123.333000	71.270515	9.404484	110.911682
71.279999	122.500000	71.290520	10.868206	110.880493
71.300003	120.333000	71.310524	11.517344	110.849426
71.320000	119.667000	71.330521	11.282678	110.818420
71.340004	126.500000	71.350525	10.523351	110.787537
71.360001	115.500000	71.370522	9.765902	110.756714
71.380005	117.667000	71.390526	9.413461	110.725952
71.400002	123.667000	71.410522	9.700165	110.695312
71.419998	128.333008	71.430519	10.815529	110.664795
71.440002	131.667007	71.450523	13.051375	110.634338
71.459999	132.167007	71.470520	16.910875	110.604004
71.480003	140.167007	71.490524	23.204884	110.573730
71.500000	144.667007	71.510521	33.091076	110.543518
71.519997	157.833008	71.530518	48.030495	110.513428
71.540001	175.667007	71.550522	69.452194	110.483398
71.559998	218.833008	71.570518	97.861168	110.453491
71.580002	247.833008	71.590523	131.417755	110.423645
71.599998	273.666992	71.610519	164.395370	110.393921
71.619995	313.166992	71.630516	187.792953	110.364258
71.639999	301.332977	71.650520	193.608292	110.334778
71.659996	284.166992	71.670517	180.584122	110.305298
71.680000	262.166992	71.690521	155.061768	110.275879
71.699997	241.167007	71.710518	125.750443	110.246643
71.719994	217.000000	71.730515	98.621712	110.217468
71.739998	193.000000	71.750519	76.058304	110.188354
71.759995	177.167007	71.770515	58.271420	110.159363
71.779999	154.667007	71.790520	44.558411	110.130432
71.800003	138.833008	71.810524	34.065678	110.101624
71.820000	143.000000	71.830521	26.051989	110.072937
71.840004	141.667007	71.850525	19.945501	110.044312
71.860001	132.000000	71.870522	15.331860	110.015747
71.880005	125.833000	71.890526	11.889276	109.987305
71.900002	119.500000	71.910522	9.370938	109.958984
71.919998	117.833000	71.930519	7.577013	109.930725
71.940002	120.833000	71.950523	6.351213	109.902588
71.959999	119.000000	71.970520	5.576728	109.874573
71.980003	117.333000	71.990524	5.170811	109.846619

72.000000	107.500000	72.010521	5.088383	109.818726
72.019997	116.833000	72.030518	5.324497	109.791016
72.040001	115.167000	72.050522	5.926480	109.763306
72.059998	117.000000	72.070518	7.013219	109.735718
72.080002	121.500000	72.090523	8.810159	109.708313
72.099998	127.500000	72.110519	11.691175	109.680908
72.119995	129.167007	72.130516	16.234034	109.653625
72.139999	127.333000	72.150520	23.255644	109.626465
72.159996	154.500000	72.170517	33.757927	109.599365
72.180000	165.000000	72.190521	48.714245	109.572388
72.199997	172.833008	72.210518	68.444489	109.545532
72.219994	209.667007	72.230515	91.643326	109.518738
72.239998	215.333008	72.250519	114.384827	109.492065
72.259995	228.000000	72.270508	130.449310	109.465454
72.279999	236.833008	72.290512	134.353394	109.438965
72.300003	228.667007	72.310516	125.268417	109.412598
72.320000	221.833008	72.330513	107.797409	109.386292
72.340004	192.167007	72.350517	88.165337	109.360107
72.360001	183.167007	72.370514	70.395729	109.334045
72.380005	165.500000	72.390518	55.624439	109.308044
72.400002	156.667007	72.410515	43.591061	109.282166
72.419998	141.833008	72.430511	33.826294	109.256348
72.440002	139.333008	72.450516	26.003714	109.230713
72.459999	138.833008	72.470512	19.857647	109.205078
72.480003	127.000000	72.490517	15.101169	109.179626
72.500000	112.833000	72.510513	11.463336	109.154236
72.519997	118.833000	72.530510	8.702418	109.128906
72.540001	111.833000	72.550514	6.621241	109.103760
72.559998	112.500000	72.570511	5.064326	109.078674
72.580002	105.500000	72.590515	3.905805	109.053711
72.599998	112.000000	72.610512	3.049004	109.028809
72.619995	109.167000	72.630508	2.417929	109.004089
72.639999	109.833000	72.650513	1.955203	108.979370
72.659996	110.333000	72.670509	1.618919	108.954834
72.680000	112.667000	72.690514	1.378827	108.930359
72.699997	110.167000	72.710510	1.215439	108.906006
72.719994	106.333000	72.730507	1.118189	108.881775
72.739998	104.833000	72.750511	1.085830	108.857605

72.759995	117.667000	72.770508	1.127511	108.833557
72.779999	112.333000	72.790512	1.264989	108.809631
72.800003	111.500000	72.810516	1.535217	108.785767
72.820000	109.167000	72.830513	1.991184	108.762024
72.840004	112.833000	72.850517	2.697621	108.738403
72.860001	114.333000	72.870514	3.710227	108.714844
72.880005	115.500000	72.890518	5.034097	108.691406
72.900002	123.833000	72.910515	6.552533	108.668091
72.919998	121.833000	72.930511	7.974761	108.644897
72.940002	124.500000	72.950516	8.882203	108.621765
72.959999	118.167000	72.970512	8.942369	108.598755
72.980003	121.333000	72.990517	8.156537	108.575867
73.000000	125.333000	73.010513	6.863929	108.553040
73.019997	118.833000	73.030510	5.481447	108.530334
73.040001	110.333000	73.050514	4.271896	108.507751
73.059998	109.333000	73.070511	3.314153	108.485291
73.080002	111.000000	73.090515	2.583192	108.462891
73.099998	111.167000	73.110512	2.028573	108.440613
73.119995	109.500000	73.130508	1.605663	108.418457
73.139999	106.500000	73.150513	1.282958	108.396362
73.159996	107.167000	73.170509	1.039190	108.374390
73.180000	111.833000	73.190514	0.858699	108.352539
73.199997	108.667000	73.210510	0.729740	108.330811
73.219994	113.167000	73.230507	0.642756	108.309143
73.239998	111.667000	73.250511	0.590177	108.287598
73.259995	104.333000	73.270508	0.566245	108.266174
73.279999	117.333000	73.290512	0.566792	108.244873
73.300003	112.667000	73.310516	0.589302	108.223633
73.320000	115.500000	73.330513	0.632742	108.202515
73.340004	113.500000	73.350517	0.697174	108.181519
73.360001	114.500000	73.370514	0.784157	108.160583
73.380005	114.000000	73.390518	0.896481	108.139771
73.400002	108.000000	73.410515	1.038044	108.119080
73.419998	109.500000	73.430511	1.214426	108.098511
73.440002	110.167000	73.450516	1.433073	108.078003
73.459999	116.667000	73.470512	1.704569	108.057617
73.480003	117.667000	73.490517	2.045368	108.037354
73.500000	108.833000	73.510513	2.480704	108.017212

73.519997	113.000000	73.530510	3.053330	107.997192
73.540001	112.833000	73.550514	3.834727	107.977173
73.559998	114.500000	73.570511	4.942269	107.957336
73.580002	118.000000	73.590515	6.567681	107.937622
73.599998	128.667007	73.610512	9.006596	107.917969
73.619995	124.833000	73.630508	12.696811	107.898438
73.639999	122.833000	73.650513	18.235407	107.879028
73.659996	138.500000	73.670509	26.325397	107.859680
73.680000	147.833008	73.690514	37.606617	107.840515
73.699997	152.833008	73.710510	52.200607	107.821411
73.719994	173.667007	73.730507	69.054832	107.802429
73.739998	185.667007	73.750511	85.337433	107.783508
73.759995	209.333008	73.770508	96.787163	107.764771
73.779999	210.667007	73.790512	99.813065	107.746033
73.800003	208.667007	73.810516	94.105286	107.727478
73.820000	192.833008	73.830513	83.137390	107.708984
73.840004	181.167007	73.850517	71.757942	107.690674
73.860001	179.833008	73.870514	63.678493	107.672363
73.880005	168.500000	73.890518	60.855026	107.654236
73.900002	169.167007	73.910515	64.224190	107.636169
73.919998	185.000000	73.930511	74.281746	107.618225
73.940002	193.333008	73.950516	90.910805	107.600403
73.959999	219.500000	73.970512	112.436829	107.582642
73.980003	248.167007	73.990517	134.633926	107.565063
74.000000	264.500000	74.010513	150.837250	107.547485
74.019997	274.666992	74.030510	154.717865	107.530151
74.040001	267.832977	74.050514	144.389145	107.512756
74.059998	238.667007	74.070511	123.918480	107.495605
74.080002	211.833008	74.090515	100.101700	107.478455
74.099998	192.167007	74.110512	78.198143	107.461487
74.119995	170.000000	74.130508	60.331543	107.444580
74.139999	161.000000	74.150513	46.482292	107.427795
74.159996	148.000000	74.170509	35.869240	107.411133
74.180000	150.333008	74.190514	27.678446	107.394531
74.199997	145.500000	74.210510	21.315109	107.378113
74.219994	122.000000	74.230507	16.361118	107.361694
74.239998	115.833000	74.250511	12.520493	107.345459
74.259995	117.667000	74.270508	9.570935	107.329285

74.279999	110.000000	74.290512	7.327264	107.313232
74.300003	112.667000	74.310516	5.638371	107.297241
74.320000	113.333000	74.330513	4.378222	107.281433
74.340004	106.500000	74.350517	3.443048	107.265686
74.360001	111.000000	74.370514	2.752371	107.250000
74.380005	106.500000	74.390518	2.242813	107.234497
74.400002	109.167000	74.410515	1.868072	107.219055
74.419998	106.167000	74.430511	1.593788	107.203735
74.440002	101.667000	74.450516	1.395601	107.188477
74.459999	99.666695	74.470512	1.256835	107.173340
74.480003	107.333000	74.490517	1.165973	107.158325
74.500000	101.667000	74.510513	1.115865	107.143433
74.519997	101.167000	74.530510	1.102290	107.128601
74.540001	100.667000	74.550514	1.123612	107.113892
74.559998	99.666695	74.570511	1.180433	107.099243
74.580002	106.833000	74.590515	1.276057	107.084717
74.599998	102.167000	74.610512	1.417353	107.070312
74.619995	106.833000	74.630508	1.617111	107.055969
74.639999	103.000000	74.650513	1.897883	107.041748
74.659996	107.000000	74.670509	2.297643	107.027649
74.680000	113.000000	74.690514	2.880296	107.013672
74.699997	104.167000	74.710510	3.747416	106.999756
74.719994	107.500000	74.730507	5.056007	106.985962
74.739998	112.000000	74.750511	7.034484	106.972229
74.759995	117.500000	74.770508	9.984595	106.958618
74.779999	116.500000	74.790512	14.259084	106.945068
74.800003	129.167007	74.810516	20.153315	106.931702
74.820000	130.167007	74.830513	27.688061	106.918335
74.840004	147.000000	74.850517	36.297626	106.905151
74.860001	152.333008	74.870514	44.569641	106.892029
74.880005	162.000000	74.890518	50.531502	106.879028
74.900002	163.500000	74.910515	52.663025	106.866089
74.919998	160.000000	74.930511	51.107231	106.853271
74.940002	150.833008	74.950516	47.747307	106.840515
74.959999	161.167007	74.970512	44.971554	106.827881
74.980003	154.167007	74.990517	44.318630	106.815369
75.000000	154.333008	75.010513	45.951523	106.802917
75.019997	164.833008	75.030510	48.755768	106.790649

75.040001	165.667007	75.050514	50.786465	106.778381
75.059998	161.667007	75.070511	50.152100	106.766235
75.080002	154.333008	75.090515	46.139500	106.754150
75.099998	154.667007	75.110512	39.647408	106.742249
75.119995	147.333008	75.130508	32.400776	106.730408
75.139999	131.833008	75.150513	25.804932	106.718628
75.159996	126.667000	75.170509	20.474310	106.706970
75.180000	128.000000	75.190514	16.419424	106.695374
75.199997	126.000000	75.210510	13.425957	106.683899
75.219994	127.167000	75.230507	11.263495	106.672485
75.239998	122.667000	75.250511	9.764446	106.661194
75.259995	131.000000	75.270508	8.828197	106.650024
75.279999	121.000000	75.290512	8.407934	106.638916
75.300003	133.667007	75.310516	8.512577	106.627930
75.320000	129.333008	75.330513	9.218562	106.616943
75.340004	139.167007	75.350517	10.701848	106.606140
75.360001	135.333008	75.370514	13.282372	106.595459
75.380005	147.500000	75.390518	17.491236	106.584778
75.400002	146.333008	75.410515	24.128092	106.574219
75.419998	162.500000	75.430511	34.309273	106.563782
75.440002	181.000000	75.450516	49.407028	106.553406
75.459999	196.333008	75.470512	70.727303	106.543091
75.480003	225.000000	75.490517	98.836304	106.532959
75.500000	256.832977	75.510513	132.272583	106.522827
75.519997	282.166992	75.530510	166.321548	106.512817
75.540001	312.666992	75.550514	192.986740	106.502869
75.559998	321.000000	75.570511	203.910370	106.493042
75.580002	325.666992	75.590515	195.559799	106.483337
75.599998	298.832977	75.610512	171.950638	106.473633
75.619995	273.500000	75.630508	141.659424	106.464111
75.639999	240.167007	75.650513	112.269600	106.454651
75.659996	208.500000	75.670509	87.566406	106.445251
75.680000	197.167007	75.690514	68.116508	106.435913
75.699997	172.000000	75.710510	53.110138	106.426697
75.719994	171.167007	75.730507	41.497387	106.417542
75.739998	156.500000	75.750511	32.450977	106.408508
75.759995	144.833008	75.770508	25.406006	106.399536
75.779999	137.667007	75.790512	19.960352	106.390625

75.800003	130.833008	75.810516	15.824224	106.381897
75.820000	131.667007	75.830513	12.769855	106.373169
75.840004	125.833000	75.850517	10.615743	106.364502
75.860001	122.833000	75.870514	9.234888	106.355957
75.880005	125.833000	75.890518	8.558089	106.347473
75.900002	131.833008	75.910515	8.595100	106.339111
75.919998	123.667000	75.930511	9.454950	106.330811
75.940002	130.000000	75.950516	11.375223	106.322571
75.959999	129.833008	75.970512	14.742012	106.314453
75.980003	139.000000	75.990517	20.096153	106.306335
76.000000	147.167007	76.010513	28.053816	106.298401
76.019997	162.333008	76.030510	39.110561	106.290466
76.040001	170.833008	76.050514	53.215496	106.282654
76.059998	189.500000	76.070511	69.139465	106.274902
76.080002	206.000000	76.090515	84.068138	106.267273
76.099998	211.333008	76.110512	94.026260	106.259705
76.119995	212.833008	76.130508	95.776367	106.252197
76.139999	201.167007	76.150513	88.980049	106.244751
76.159996	198.667007	76.170509	76.523575	106.237366
76.180000	178.333008	76.190514	62.424500	106.230103
76.199997	168.000000	76.210510	49.599720	106.222900
76.219994	161.167007	76.230507	39.183552	106.215759
76.239998	150.000000	76.250511	31.153013	106.208740
76.259995	139.833008	76.270508	25.093460	106.201721
76.279999	139.833008	76.290512	20.599623	106.194824
76.300003	132.000000	76.310516	17.424168	106.187988
76.320000	130.833008	76.330513	15.475296	106.181213
76.340004	123.000000	76.350517	14.801241	106.174561
76.360001	138.000000	76.370514	15.587224	106.167908
76.380005	138.667007	76.390518	18.146950	106.161377
76.400002	151.167007	76.410515	22.880081	106.154907
76.419998	150.833008	76.430511	30.150728	106.148499
76.440002	166.500000	76.450516	40.011608	106.142151
76.459999	179.833008	76.470512	51.751198	106.135925
76.480003	198.333008	76.490517	63.510948	106.129700
76.500000	197.500000	76.510513	72.370232	106.123596
76.519997	196.833008	76.530510	75.518921	106.117554
76.540001	197.333008	76.550514	71.974525	106.111572

76.559998	186.500000	76.570511	63.344742	106.105652
76.580002	179.500000	76.590515	52.645264	106.099731
76.599998	161.667007	76.610512	42.453030	106.093994
76.619995	159.667007	76.630508	34.002899	106.088257
76.639999	147.833008	76.650513	27.470957	106.082581
76.659996	144.500000	76.670509	22.582005	106.077026
76.680000	147.167007	76.690514	18.993586	106.071472
76.699997	137.833008	76.710510	16.460056	106.065979
76.719994	135.333008	76.730507	14.841773	106.060547
76.739998	128.333008	76.750511	14.100258	106.055237
76.759995	143.333008	76.770508	14.298695	106.049988
76.779999	131.667007	76.790512	15.625762	106.044739
76.800003	137.500000	76.810516	18.444040	106.039551
76.820000	139.333008	76.830513	23.352390	106.034485
76.840004	162.667007	76.850517	31.259060	106.029419
76.860001	166.833008	76.870514	43.389587	106.024475
76.880005	188.333008	76.890518	61.212337	106.019531
76.900002	220.667007	76.910515	86.027870	106.014648
76.919998	245.167007	76.930511	118.197441	106.009888
76.940002	286.332977	76.950516	155.861038	106.005066
76.959999	322.332977	76.970512	193.709000	106.000427
76.980003	362.166992	76.990517	223.463562	105.995789
77.000000	361.500000	77.010513	237.162537	105.991211
77.019997	361.166992	77.030510	232.416473	105.986694
77.040001	329.166992	77.050514	214.689285	105.982178
77.059998	310.332977	77.070511	193.984177	105.977783
77.080002	290.666992	77.090515	179.037430	105.973389
77.099998	296.666992	77.110512	174.090591	105.969055
77.119995	302.666992	77.130508	178.659821	105.964844
77.139999	315.166992	77.150513	188.439026	105.960632
77.159996	333.332977	77.170509	196.494507	105.956482
77.180000	322.332977	77.190514	195.765060	105.952332
77.199997	301.832977	77.210510	182.690994	105.948303
77.219994	285.332977	77.230507	159.313766	105.944275
77.239998	258.166992	77.250511	131.433411	105.940308
77.259995	231.833008	77.270508	104.681206	105.936401
77.279999	201.667007	77.290512	82.034950	105.932495
77.300003	186.333008	77.310516	64.057411	105.928711

77.320000	163.667007	77.330513	50.095791	105.924927
77.340004	147.667007	77.350517	39.234249	105.921204
77.360001	156.667007	77.370514	30.729250	105.917480
77.380005	147.833008	77.390518	24.036547	105.913818
77.400002	138.667007	77.410515	18.784849	105.910278
77.419998	129.833008	77.430511	14.695266	105.906677
77.440002	119.667000	77.450516	11.551972	105.903198
77.459999	115.333000	77.470512	9.184647	105.899719
77.480003	111.667000	77.490517	7.453900	105.896301
77.500000	113.833000	77.510513	6.256732	105.892883
77.519997	110.333000	77.530510	5.519853	105.889526
77.540001	111.167000	77.550514	5.197876	105.886230
77.559998	116.000000	77.570511	5.260990	105.882935
77.580002	104.500000	77.590515	5.671723	105.879700
77.599998	114.667000	77.610512	6.348771	105.876526
77.619995	113.667000	77.630508	7.133753	105.873352
77.639999	113.333000	77.650513	7.792198	105.870239
77.659996	114.833000	77.670509	8.088841	105.867188
77.680000	110.500000	77.690514	7.919268	105.864136
77.699997	119.167000	77.710510	7.383322	105.861084
77.719994	110.167000	77.730507	6.717928	105.858093
77.739998	109.000000	77.750511	6.157335	105.855164
77.759995	112.833000	77.770508	5.854375	105.852234
77.779999	108.500000	77.790512	5.893914	105.849304
77.800003	109.833000	77.810516	6.348298	105.846436
77.820000	121.167000	77.830513	7.321304	105.843628
77.840004	121.000000	77.850517	8.971774	105.840820
77.860001	118.167000	77.870514	11.507777	105.838074
77.880005	125.333000	77.890518	15.156143	105.835327
77.900002	124.000000	77.910515	20.066019	105.832642
77.919998	129.333008	77.930511	26.161480	105.829895
77.940002	138.500000	77.950516	32.953957	105.827271
77.959999	149.000000	77.970512	39.472397	105.824585
77.980003	153.667007	77.990517	44.589363	105.822021
78.000000	160.667007	78.010513	47.736435	105.819458
78.019997	167.667007	78.030510	49.613632	105.816895
78.040001	159.333008	78.050514	52.174919	105.814331
78.059998	173.167007	78.070511	57.924660	105.811829

78.080002	182.333008	78.090515	69.207069	105.809326
78.099998	202.833008	78.110512	87.795319	105.806885
78.119995	243.833008	78.130508	114.518684	105.804382
78.139999	269.332977	78.150513	148.410217	105.802002
78.159996	308.666992	78.170509	185.455826	105.799561
78.180000	348.166992	78.190514	218.172974	105.797180
78.199997	363.332977	78.210510	237.336441	105.794800
78.219994	365.332977	78.230507	236.614777	105.792419
78.239998	347.666992	78.250511	216.724091	105.790100
78.259995	334.500000	78.270508	185.029648	105.787781
78.279999	289.832977	78.290512	150.537079	105.785461
78.300003	240.500000	78.310516	119.404510	105.783142
78.320000	209.333008	78.330513	93.896667	105.780884
78.340004	195.500000	78.350517	73.783783	105.778625
78.360001	176.667007	78.370514	58.023743	105.776306
78.380005	167.000000	78.390518	45.568485	105.774170
78.400002	151.500000	78.410515	35.659935	105.771851
78.419998	148.833008	78.430511	27.763958	105.769653
78.440002	135.500000	78.450516	21.500786	105.767456
78.459999	127.167000	78.470512	16.581495	105.765198
78.480003	113.167000	78.490517	12.756588	105.763062
78.500000	119.667000	78.510513	9.816611	105.760803
78.519997	116.667000	78.530510	7.576506	105.758667
78.540001	110.000000	78.550514	5.880529	105.756470
78.559998	107.833000	78.570511	4.601980	105.754272
78.580002	103.000000	78.590515	3.637324	105.752136
78.599998	110.333000	78.610512	2.907448	105.750000
78.619995	109.000000	78.630508	2.351154	105.747803
78.639999	108.667000	78.650513	1.922948	105.745667
78.659996	105.000000	78.670509	1.589815	105.743530
78.680000	108.000000	78.690514	1.327230	105.741333
78.699997	103.000000	78.710510	1.117838	105.739258
78.719994	103.167000	78.730507	0.948813	105.737061
78.739998	105.500000	78.750511	0.810850	105.734924
78.759995	93.500000	78.770508	0.697243	105.732727
78.779999	104.167000	78.790512	0.602831	105.730591
78.800003	103.000000	78.810516	0.523822	105.728455
78.820000	96.500000	78.830513	0.457297	105.726318

78.840004	102.833000	78.850517	0.400916	105.724121
78.860001	98.000000	78.870514	0.352917	105.721985
78.880005	100.167000	78.890518	0.311828	105.719788
78.900002	102.667000	78.910515	0.276523	105.717590
78.919998	99.000000	78.930511	0.246051	105.715454
78.940002	100.000000	78.950516	0.219654	105.713196
78.959999	101.000000	78.970512	0.196696	105.710999
78.980003	95.500000	78.990517	0.176648	105.708801
79.000000	97.666695	79.010513	0.159098	105.706604
79.019997	102.833000	79.030510	0.143681	105.704407
79.040001	98.666695	79.050514	0.130092	105.702148
79.059998	96.500000	79.070511	0.118088	105.699829
79.080002	98.166695	79.090515	0.107392	105.697632
79.099998	95.500000	79.110512	0.097943	105.695312
79.119995	103.167000	79.130508	0.089529	105.693054
79.139999	104.667000	79.150513	0.082014	105.690735
79.159996	97.000000	79.170509	0.075292	105.688416
79.180000	94.333298	79.190514	0.069263	105.686096
79.199997	96.833298	79.210510	0.063732	105.683716
79.219994	95.500000	79.230507	0.058861	105.681335
79.239998	91.166695	79.250511	0.054466	105.678955
79.259995	103.167000	79.270508	0.050498	105.676575
79.279999	98.500000	79.290512	0.046907	105.674133
79.300003	94.000000	79.310516	0.043653	105.671692
79.320000	90.500000	79.330513	0.040726	105.669250
79.340004	93.666695	79.350517	0.038122	105.666748
79.360001	96.333298	79.370514	0.035689	105.664185
79.380005	99.000000	79.390518	0.033492	105.661682
79.400002	103.167000	79.410515	0.031477	105.659180
79.419998	98.500000	79.430511	0.029641	105.656555
79.440002	96.500000	79.450516	0.027969	105.653931
79.459999	97.166695	79.470512	0.026445	105.651306
79.480003	100.000000	79.490517	0.025058	105.648682
79.500000	94.000000	79.510513	0.023795	105.645996
79.519997	94.833298	79.530510	0.022648	105.643311
79.540001	89.666695	79.550514	0.021606	105.640503
79.559998	91.833298	79.570511	0.020661	105.637817
79.580002	100.000000	79.590515	0.019812	105.635010

79.599998	96.000000	79.610512	0.019047	105.632202
79.619995	100.333000	79.630508	0.018363	105.629272
79.639999	96.166695	79.650513	0.017755	105.626465
79.659996	91.666695	79.670509	0.017220	105.623535
79.680000	89.666695	79.690514	0.016756	105.620544
79.699997	97.500000	79.710510	0.016279	105.617554
79.719994	103.167000	79.730507	0.015951	105.614563
79.739998	101.500000	79.750511	0.015689	105.611511
79.759995	95.166695	79.770508	0.015491	105.608398
79.779999	93.833298	79.790512	0.015359	105.605286
79.800003	89.500000	79.810516	0.015293	105.602112
79.820000	98.000000	79.830513	0.015295	105.598877
79.840004	99.666695	79.850517	0.015367	105.595703
79.860001	98.833298	79.870514	0.015513	105.592407
79.880005	105.333000	79.890518	0.015737	105.589111
79.900002	90.166695	79.910515	0.016045	105.585754
79.919998	96.500000	79.930511	0.016443	105.582336
79.940002	92.000000	79.950516	0.016939	105.578857
79.959999	100.333000	79.970512	0.017543	105.575439
79.980003	98.000000	79.990517	0.018266	105.571899
80.000000	97.666695	80.010513	0.019123	105.568359
80.019997	104.500000	80.030510	0.020131	105.564758
80.040001	99.500000	80.050514	0.021279	105.561035
80.059998	101.000000	80.070511	0.022614	105.557373
80.080002	97.166695	80.090515	0.024213	105.553650
80.099998	102.000000	80.110512	0.026070	105.549866
80.119995	101.167000	80.130508	0.028228	105.546021
80.139999	109.667000	80.150505	0.030738	105.542114
80.159996	97.500000	80.170502	0.033662	105.538147
80.180000	98.500000	80.190506	0.037077	105.534180
80.199997	100.500000	80.210503	0.041072	105.530151
80.219994	97.166695	80.230499	0.045812	105.526062
80.239998	99.833298	80.250504	0.051333	105.521973
80.259995	100.500000	80.270500	0.057844	105.517700
80.279999	99.000000	80.290504	0.065584	105.513489
80.300003	98.166695	80.310509	0.074716	105.509155
80.320000	97.500000	80.330505	0.085553	105.504822
80.340004	95.166695	80.350510	0.098457	105.500427

80.360001	99.833298	80.370506	0.113734	105.495972
80.380005	98.833298	80.390511	0.131881	105.491455
80.400002	100.833000	80.410507	0.153435	105.486877
80.419998	101.000000	80.430504	0.179107	105.482300
80.440002	95.166695	80.450508	0.209889	105.477539
80.459999	96.166695	80.470505	0.247233	105.472839
80.480003	92.833298	80.490509	0.293518	105.468018
80.500000	96.333298	80.510506	0.352586	105.463135
80.519997	101.000000	80.530502	0.430878	105.458252
80.540001	99.000000	80.550507	0.538960	105.453247
80.559998	97.166695	80.570503	0.693534	105.448242
80.580002	98.166695	80.590508	0.920595	105.443115
80.599998	103.167000	80.610504	1.258079	105.437927
80.619995	96.666695	80.630501	1.758885	105.432739
80.639999	95.333298	80.650505	2.490922	105.427429
80.659996	98.166695	80.670502	3.528251	105.422119
80.680000	98.500000	80.690506	4.932666	105.416687
80.699997	103.667000	80.710503	6.709239	105.411194
80.719994	107.833000	80.730499	8.750269	105.405640
80.739998	111.167000	80.750504	10.783426	105.400024
80.759995	110.167000	80.770500	12.386402	105.394348
80.779999	112.167000	80.790504	13.137102	105.388550
80.800003	114.333000	80.810509	12.838312	105.382751
80.820000	114.167000	80.830505	11.654922	105.376831
80.840004	111.000000	80.850510	10.006285	105.370911
80.860001	110.500000	80.870506	8.321326	105.364868
80.880005	111.500000	80.890511	6.862904	105.358765
80.900002	104.500000	80.910507	5.723631	105.352661
80.919998	114.500000	80.930504	4.899973	105.346375
80.940002	108.500000	80.950508	4.369014	105.340088
80.959999	107.000000	80.970505	4.129487	105.333679
80.980003	103.833000	80.990509	4.214545	105.327209
81.000000	109.667000	81.010506	4.695108	105.320679
81.019997	110.833000	81.030502	5.674568	105.314087
81.040001	105.667000	81.050507	7.272853	105.307434
81.059998	115.000000	81.070503	9.584282	105.300659
81.080002	113.167000	81.090508	12.608390	105.293823
81.099998	121.167000	81.110504	16.137650	105.286865

81.119995	118.500000	81.130501	19.675537	105.279907
81.139999	122.500000	81.150505	22.465727	105.272827
81.159996	124.500000	81.170502	23.744333	105.265686
81.180000	136.833008	81.190506	23.150047	105.258484
81.199997	129.333008	81.210503	20.960293	105.251160
81.219994	121.667000	81.230499	17.912457	105.243835
81.239998	117.000000	81.250504	14.764579	105.236328
81.259995	114.833000	81.270500	11.988526	105.228821
81.279999	106.333000	81.290504	9.734869	105.221191
81.300003	99.166695	81.310509	7.971456	105.213501
81.320000	104.167000	81.330505	6.606840	105.205688
81.340004	95.000000	81.350510	5.555199	105.197876
81.360001	102.333000	81.370506	4.757499	105.189880
81.380005	105.000000	81.390511	4.175450	105.181885
81.400002	111.333000	81.410507	3.787704	105.173706
81.419998	100.667000	81.430504	3.584689	105.165527
81.440002	102.500000	81.450508	3.570697	105.157288
81.459999	105.167000	81.470505	3.768773	105.148926
81.480003	100.333000	81.490509	4.230594	105.140442
81.500000	111.000000	81.510506	5.049406	105.131836
81.519997	101.333000	81.530502	6.378756	105.123230
81.540001	119.167000	81.550507	8.452511	105.114563
81.559998	124.333000	81.570503	11.597281	105.105774
81.580002	124.000000	81.590508	16.237480	105.096802
81.599998	133.000000	81.610504	22.840723	105.087891
81.619995	138.333008	81.630501	31.803837	105.078796
81.639999	159.000000	81.650505	43.209160	105.069641
81.659996	164.667007	81.670502	56.461761	105.060425
81.680000	173.667007	81.690506	70.026039	105.051025
81.699997	184.000000	81.710503	81.484596	105.041626
81.719994	194.667007	81.730499	88.376541	105.032104
81.739998	188.167007	81.750504	89.432381	105.022461
81.759995	190.000000	81.770500	85.292809	105.012817
81.779999	181.167007	81.790504	77.896736	105.002991
81.800003	176.833008	81.810509	69.135391	104.993042
81.820000	166.167007	81.830505	60.064999	104.983093
81.840004	157.000000	81.850510	51.095913	104.973022
81.860001	147.667007	81.870506	42.533405	104.962830

81.880005	135.500000	81.890511	34.731426	104.952576
81.900002	137.667007	81.910507	27.983152	104.942261
81.919998	128.667007	81.930504	22.377075	104.931763
81.940002	122.167000	81.950508	17.831854	104.921204
81.959999	128.500000	81.970505	14.192818	104.910583
81.980003	118.167000	81.990509	11.293555	104.899841
82.000000	106.167000	82.010506	8.997752	104.888977
82.019997	117.167000	82.030502	7.194366	104.878052
82.040001	102.833000	82.050507	5.796513	104.867004
82.059998	103.833000	82.070503	4.737042	104.855896
82.080002	99.833298	82.090508	3.962659	104.844666
82.099998	102.500000	82.110504	3.437294	104.833313
82.119995	101.667000	82.130501	3.142452	104.821899
82.139999	103.333000	82.150505	3.081392	104.810364
82.159996	101.333000	82.170502	3.283960	104.798706
82.180000	105.500000	82.190506	3.811105	104.786987
82.199997	107.167000	82.210503	4.755994	104.775146
82.219994	106.000000	82.230499	6.238999	104.763245
82.239998	103.500000	82.250504	8.384878	104.751221
82.259995	117.000000	82.270500	11.267240	104.739136
82.279999	109.333000	82.290504	14.826154	104.726868
82.300003	109.000000	82.310509	18.752335	104.714539
82.320000	113.833000	82.330505	22.429102	104.702087
82.340004	126.000000	82.350510	25.038797	104.689575
82.360001	119.500000	82.370506	25.878925	104.676880
82.380005	118.333000	82.390511	24.762646	104.664185
82.400002	125.833000	82.410507	22.146664	104.651306
82.419998	120.000000	82.430504	18.846136	104.638367
82.440002	112.667000	82.450508	15.594178	104.625305
82.459999	111.333000	82.470497	12.804570	104.612183
82.480003	106.000000	82.490501	10.587104	104.598877
82.500000	107.667000	82.510498	8.907246	104.585571
82.519997	108.167000	82.530495	7.696979	104.572083
82.540001	106.833000	82.550499	6.919906	104.558472
82.559998	109.000000	82.570496	6.589917	104.544800
82.580002	113.000000	82.590500	6.770375	104.531006
82.599998	105.167000	82.610497	7.569162	104.517151
82.619995	106.000000	82.630493	9.124121	104.503174

82.639999	120.000000	82.650497	11.569885	104.489014
82.659996	118.000000	82.670494	14.964907	104.474792
82.680000	116.833000	82.690498	19.188343	104.460449
82.699997	116.833000	82.710495	23.804634	104.446106
82.719994	120.500000	82.730492	28.024042	104.431519
82.739998	129.000000	82.750496	30.856537	104.416870
82.759995	124.000000	82.770493	31.521967	104.402100
82.779999	133.333008	82.790497	29.899427	104.387329
82.800003	122.167000	82.810501	26.610794	104.372314
82.820000	119.667000	82.830498	22.640165	104.357300
82.840004	113.500000	82.850502	18.817434	104.342102
82.860001	117.667000	82.870499	15.583178	104.326782
82.880005	109.500000	82.890503	13.036900	104.311401
82.900002	109.500000	82.910500	11.119624	104.295898
82.919998	117.833000	82.930496	9.741798	104.280273
82.940002	109.500000	82.950500	8.851311	104.264587
82.959999	111.500000	82.970497	8.452514	104.248718
82.980003	110.333000	82.990501	8.606996	104.232788
83.000000	116.333000	83.010498	9.435026	104.216736
83.019997	115.333000	83.030495	11.114324	104.200562
83.040001	116.833000	83.050499	13.870471	104.184326
83.059998	118.500000	83.070496	17.935856	104.167969
83.080002	131.500000	83.090500	23.479143	104.151489
83.099998	140.833008	83.110497	30.461857	104.134888
83.119995	152.667007	83.130493	38.501469	104.118164
83.139999	157.167007	83.150497	46.794624	104.101318
83.159996	173.000000	83.170494	54.247486	104.084412
83.180000	174.333008	83.190498	59.888786	104.067383
83.199997	171.333008	83.210495	63.300240	104.050232
83.219994	167.833008	83.230492	64.865623	104.032959
83.239998	181.833008	83.250496	65.708839	104.015564
83.259995	184.500000	83.270493	67.468483	103.998108
83.279999	176.500000	83.290497	71.929939	103.980530
83.300003	190.833008	83.310501	80.501801	103.962830
83.320000	204.167007	83.330498	93.640259	103.945007
83.340004	221.833008	83.350502	110.359467	103.927063
83.360001	242.500000	83.370499	127.893318	103.908997
83.380005	270.000000	83.390503	142.038010	103.890869

83.400002	256.166992	83.410500	148.438217	103.872681
83.419998	268.000000	83.430496	144.710220	103.854248
83.440002	247.667007	83.450500	131.818726	103.835815
83.459999	229.333008	83.470497	113.481125	103.817139
83.480003	203.333008	83.490501	93.945320	103.798462
83.500000	187.333008	83.510498	76.167114	103.779663
83.519997	171.500000	83.530495	61.309895	103.760742
83.540001	157.167007	83.550499	49.340134	103.741699
83.559998	151.167007	83.570496	39.762497	103.722595
83.580002	145.000000	83.590500	32.033562	103.703369
83.599998	140.500000	83.610497	25.747681	103.683960
83.619995	129.333008	83.630493	20.618273	103.664490
83.639999	134.500000	83.650497	16.447311	103.644897
83.659996	111.500000	83.670494	13.089572	103.625122
83.680000	116.500000	83.690498	10.418933	103.605347
83.699997	113.167000	83.710495	8.328172	103.585449
83.719994	112.000000	83.730492	6.718043	103.565430
83.739998	109.833000	83.750496	5.501250	103.545288
83.759995	112.000000	83.770493	4.603896	103.525024
83.779999	108.833000	83.790497	3.962869	103.504639
83.800003	106.167000	83.810501	3.528509	103.484192
83.820000	104.667000	83.830498	3.262153	103.463562
83.840004	100.500000	83.850502	3.135139	103.442932
83.860001	111.667000	83.870499	3.128152	103.422119
83.880005	104.500000	83.890503	3.230060	103.401184
83.900002	111.167000	83.910500	3.438050	103.380127
83.919998	107.667000	83.930496	3.759267	103.359009
83.940002	104.333000	83.950500	4.214462	103.337769
83.959999	109.500000	83.970497	4.843852	103.316406
83.980003	108.500000	83.990501	5.719243	103.294922
84.000000	118.833000	84.010498	6.958067	103.273315
84.019997	115.500000	84.030495	8.747771	103.251709
84.040001	119.000000	84.050499	11.374154	103.229858
84.059998	127.667000	84.070496	15.248561	103.207947
84.080002	129.000000	84.090500	20.941418	103.185974
84.099998	139.833008	84.110497	29.169445	103.163879
84.119995	146.333008	84.130493	40.750450	103.141663
84.139999	154.167007	84.150497	56.436810	103.119263

84.159996	187.167007	84.170494	76.571594	103.096863
84.180000	217.167007	84.190498	100.661476	103.074341
84.199997	243.000000	84.210495	126.828934	103.051636
84.219994	271.500000	84.230492	151.728287	103.028870
84.239998	285.666992	84.250496	171.175400	103.006042
84.259995	303.500000	84.270493	181.666260	102.983032
84.279999	299.832977	84.290497	181.974365	102.959961
84.300003	275.166992	84.310501	173.380981	102.936707
84.320000	252.333008	84.330498	158.508438	102.913391
84.340004	246.000000	84.350502	139.961258	102.890076
84.360001	223.833008	84.370499	119.933418	102.866516
84.380005	212.000000	84.390503	100.236687	102.842896
84.400002	198.667007	84.410500	82.276436	102.819153
84.419998	184.167007	84.430496	66.797836	102.795288
84.440002	167.167007	84.450500	53.922905	102.771423
84.459999	163.333008	84.470497	43.399494	102.747375
84.480003	142.833008	84.490501	34.829533	102.723267
84.500000	139.167007	84.510498	27.855301	102.698975
84.519997	130.167007	84.530495	22.182339	102.674622
84.540001	127.667000	84.550499	17.584778	102.650146
84.559998	117.667000	84.570496	13.887747	102.625610
84.580002	116.833000	84.590500	10.939240	102.600952
84.599998	115.333000	84.610497	8.612432	102.576172
84.619995	117.167000	84.630493	6.793256	102.551331
84.639999	106.167000	84.650497	5.383632	102.526367
84.659996	97.666695	84.670494	4.302077	102.501282
84.680000	105.000000	84.690498	3.480021	102.476074
84.699997	107.333000	84.710495	2.864597	102.450867
84.719994	99.833298	84.730492	2.415098	102.425476
84.739998	101.000000	84.750496	2.102698	102.400024
84.759995	96.500000	84.770493	1.908494	102.374451
84.779999	101.000000	84.790497	1.819886	102.348755
84.800003	103.667000	84.810501	1.826908	102.322998
84.820000	99.666695	84.830498	1.915781	102.297119
84.840004	102.667000	84.850502	2.062143	102.271118
84.860001	103.333000	84.870499	2.226010	102.245056
84.880005	95.000000	84.890503	2.355473	102.218933
84.900002	94.166695	84.910500	2.401858	102.192627

84.919998	104.667000	84.930496	2.342428	102.166260
84.940002	110.667000	84.950500	2.192650	102.139832
84.959999	102.667000	84.970497	1.996871	102.113281
84.980003	104.833000	84.990501	1.803299	102.086609
85.000000	99.666695	85.010498	1.645287	102.059875
85.019997	92.333298	85.030495	1.537004	102.033020
85.040001	95.166695	85.050499	1.480552	102.006104
85.059998	97.166695	85.070496	1.474389	101.979126
85.080002	95.833298	85.090500	1.518943	101.951965
85.099998	89.666695	85.110497	1.619725	101.924805
85.119995	99.833298	85.130493	1.789579	101.897461
85.139999	98.166695	85.150497	2.051337	101.869995
85.159996	93.833298	85.170494	2.441086	101.842529
85.180000	102.833000	85.190498	3.013814	101.814941
85.199997	97.166695	85.210495	3.847342	101.787292
85.219994	103.000000	85.230492	5.047626	101.759521
85.239998	109.833000	85.250496	6.748467	101.731689
85.259995	112.333000	85.270493	9.100546	101.703735
85.279999	110.500000	85.290497	12.257454	101.675720
85.300003	114.500000	85.310501	16.343035	101.647583
85.320000	122.500000	85.330498	21.429447	101.619385
85.340004	114.167000	85.350502	27.536503	101.591125
85.360001	132.667007	85.370499	34.606171	101.562744
85.380005	141.833008	85.390503	42.442913	101.534241
85.400002	148.167007	85.410500	50.503990	101.505676
85.419998	162.167007	85.430496	57.733521	101.477051
85.440002	169.167007	85.450500	62.648827	101.448364
85.459999	182.500000	85.470497	63.898079	101.419495
85.480003	179.333008	85.490501	61.035568	101.390625
85.500000	162.167007	85.510498	54.861069	101.361633
85.519997	143.167007	85.530495	46.982052	101.332581
85.540001	136.667007	85.550499	38.978596	101.303467
85.559998	132.667007	85.570496	31.844574	101.274292
85.580002	121.667000	85.590500	25.914661	101.244873
85.599998	119.167000	85.610497	21.134542	101.215576
85.619995	114.667000	85.630493	17.304155	101.186096
85.639999	115.833000	85.650497	14.234059	101.156555
85.659996	125.000000	85.670494	11.794541	101.126953

85.680000	109.000000	85.690498	9.905069	101.097168
85.699997	100.500000	85.710495	8.522616	101.067444
85.719994	102.000000	85.730492	7.615004	101.037598
85.739998	100.667000	85.750496	7.142802	101.007690
85.759995	106.667000	85.770493	7.036771	100.977661
85.779999	108.833000	85.790497	7.177009	100.947632
85.800003	103.833000	85.810501	7.389040	100.917480
85.820000	106.667000	85.830498	7.472799	100.887207
85.840004	111.333000	85.850502	7.269284	100.856934
85.860001	105.000000	85.870499	6.730391	100.826599
85.880005	106.000000	85.890503	5.934968	100.796143
85.900002	111.500000	85.910500	5.034204	100.765564
85.919998	98.333298	85.930496	4.166993	100.735046
85.940002	101.333000	85.950500	3.412208	100.704346
85.959999	101.167000	85.970497	2.790380	100.673645
85.980003	97.666695	85.990501	2.287234	100.642822
86.000000	101.500000	86.010498	1.879313	100.612000
86.019997	104.500000	86.030495	1.544972	100.581055
86.040001	96.666695	86.050499	1.268500	100.550110
86.059998	100.500000	86.070496	1.039477	100.519043
86.080002	101.333000	86.090500	0.850272	100.487915
86.099998	94.833298	86.110497	0.695278	100.456726
86.119995	96.333298	86.130493	0.569519	100.425537
86.139999	99.000000	86.150497	0.468538	100.394165
86.159996	95.000000	86.170494	0.388362	100.362793
86.180000	94.333298	86.190498	0.325277	100.331360
86.199997	100.333000	86.210495	0.276093	100.299866
86.219994	97.666695	86.230492	0.238065	100.268311
86.239998	97.833298	86.250496	0.208875	100.236755
86.259995	104.167000	86.270493	0.186689	100.205078
86.279999	100.333000	86.290497	0.170023	100.173340
86.300003	83.000000	86.310501	0.157748	100.141541
86.320000	100.167000	86.330498	0.149006	100.109680
86.340004	100.333000	86.350502	0.143151	100.077820
86.360001	101.333000	86.370499	0.139722	100.045898
86.380005	97.833298	86.390503	0.138391	100.013855
86.400002	100.667000	86.410500	0.138938	99.981812
86.419998	102.333000	86.430496	0.141225	99.949707

86.440002	105.667000	86.450500	0.145185	99.917542
86.459999	109.000000	86.470497	0.150801	99.885376
86.480003	102.000000	86.490501	0.158115	99.853088
86.500000	106.000000	86.510498	0.167235	99.820740
86.519997	120.000000	86.530495	0.178225	99.788391
86.540001	112.000000	86.550499	0.191297	99.755981
86.559998	110.167000	86.570496	0.206664	99.723511
86.580002	108.333000	86.590500	0.224611	99.691040
86.599998	108.500000	86.610497	0.245483	99.658508
86.619995	108.500000	86.630493	0.269685	99.625916
86.639999	111.000000	86.650497	0.297771	99.593323
86.659996	107.500000	86.670494	0.330323	99.560608
86.680000	113.833000	86.690498	0.368108	99.527954
86.699997	103.667000	86.710495	0.411993	99.495178
86.719994	107.167000	86.730492	0.463068	99.462402
86.739998	107.833000	86.750496	0.522641	99.429565
86.759995	101.167000	86.770493	0.592199	99.396667
86.779999	101.667000	86.790497	0.673608	99.363770
86.800003	108.333000	86.810501	0.768972	99.330811
86.820000	106.333000	86.830498	0.880726	99.297852
86.840004	107.000000	86.850502	1.011818	99.264832
86.860001	105.500000	86.870499	1.165428	99.231812
86.880005	106.667000	86.890503	1.345430	99.198730
86.900002	101.333000	86.910500	1.556013	99.165588
86.919998	109.500000	86.930496	1.802352	99.132446
86.940002	109.167000	86.950500	2.090887	99.099304
86.959999	111.667000	86.970497	2.429752	99.066101
86.980003	105.833000	86.990501	2.831073	99.032837
87.000000	107.333000	87.010498	3.312478	98.999573
87.019997	102.333000	87.030495	3.902291	98.966309
87.040001	114.000000	87.050499	4.645840	98.932983
87.059998	115.167000	87.070496	5.614216	98.899658
87.080002	112.500000	87.090500	6.920428	98.866333
87.099998	118.500000	87.110497	8.734884	98.832886
87.119995	121.833000	87.130493	11.311093	98.799561
87.139999	125.333000	87.150497	15.009725	98.766113
87.159996	126.833000	87.170494	20.311348	98.732666
87.180000	134.833008	87.190498	27.827045	98.699158

87.199997	150.167007	87.210495	38.238911	98.665649
87.219994	161.333008	87.230492	52.205742	98.632202
87.239998	184.500000	87.250496	70.156303	98.598694
87.259995	213.333008	87.270493	91.978813	98.565186
87.279999	239.167007	87.290497	116.794472	98.531616
87.300003	270.332977	87.310501	142.800171	98.498047
87.320000	301.000000	87.330498	167.522980	98.464478
87.340004	311.500000	87.350502	188.357651	98.430908
87.360001	328.166992	87.370499	202.966965	98.397278
87.380005	326.000000	87.390503	209.559357	98.363647
87.400002	318.666992	87.410500	207.103363	98.330078
87.419998	306.000000	87.430496	195.908600	98.296448
87.440002	290.166992	87.450500	177.963593	98.262817
87.459999	281.500000	87.470497	156.408569	98.229187
87.480003	254.667007	87.490501	134.236374	98.195557
87.500000	239.000000	87.510498	113.435913	98.161865
87.519997	218.000000	87.530495	94.872849	98.128235
87.540001	199.667007	87.550499	78.750160	98.094604
87.559998	179.500000	87.570496	65.007355	98.060913
87.580002	186.333008	87.590500	53.450932	98.027283
87.599998	159.000000	87.610497	43.853031	97.993652
87.619995	152.333008	87.630493	35.955410	97.960022
87.639999	145.167007	87.650497	29.519051	97.926392
87.659996	140.833008	87.670494	24.349159	97.892700
87.680000	138.833008	87.690498	20.286171	97.859070
87.699997	127.500000	87.710495	17.223608	97.825439
87.719994	121.167000	87.730492	15.099573	97.791870
87.739998	128.333008	87.750496	13.911280	97.758240
87.759995	128.000000	87.770493	13.727071	97.724609
87.779999	128.333008	87.790497	14.697221	97.691040
87.800003	116.000000	87.810501	17.062351	97.657471
87.820000	125.000000	87.830498	21.139679	97.623840
87.840004	123.833000	87.850502	27.282154	97.590271
87.860001	143.000000	87.870499	35.755676	97.556763
87.880005	156.000000	87.890503	46.566029	97.523193
87.900002	166.500000	87.910500	59.171555	97.489685
87.919998	185.333008	87.930496	72.274872	97.456238
87.940002	198.333008	87.950500	83.807968	97.422791

87.959999	207.500000	87.970497	91.380592	97.389282
87.980003	205.167007	87.990501	93.228561	97.355896
88.000000	201.500000	88.010498	89.094139	97.322449
88.019997	185.667007	88.030495	80.406105	97.289062
88.040001	184.167007	88.050499	69.503227	97.255676
88.059998	166.667007	88.070496	58.549812	97.222290
88.080002	151.167007	88.090500	48.838989	97.188965
88.099998	152.333008	88.110497	40.812309	97.155701
88.119995	145.833008	88.130493	34.375271	97.122437
88.139999	140.833008	88.150497	29.254307	97.089233
88.159996	130.500000	88.170494	25.198389	97.056030
88.180000	132.000000	88.190498	22.036278	97.022827
88.199997	126.000000	88.210495	19.694906	96.989685
88.219994	127.833000	88.230492	18.180058	96.956604
88.239998	134.333008	88.250496	17.579521	96.923523
88.259995	125.833000	88.270493	18.074884	96.890503
88.279999	136.667007	88.290497	19.966232	96.857483
88.300003	143.167007	88.310501	23.703587	96.824463
88.320000	143.000000	88.330498	29.910177	96.791565
88.340004	152.000000	88.350502	39.394218	96.758667
88.360001	164.000000	88.370499	53.074066	96.725830
88.380005	191.167007	88.390503	71.851654	96.692993
88.400002	209.667007	88.410500	96.257271	96.660278
88.419998	239.500000	88.430496	125.996300	96.627502
88.440002	276.166992	88.450500	159.311340	96.594849
88.459999	310.666992	88.470497	192.459488	96.562256
88.480003	345.500000	88.490501	220.007629	96.529602
88.500000	362.166992	88.510498	236.147003	96.497070
88.519997	362.166992	88.530495	237.158188	96.464600
88.540001	342.500000	88.550499	223.334000	96.432129
88.559998	319.500000	88.570496	198.954010	96.399780
88.580002	282.000000	88.590500	170.016983	96.367432
88.599998	257.500000	88.610497	141.648834	96.335144
88.619995	227.333008	88.630493	116.680389	96.302917
88.639999	215.667007	88.650497	95.882225	96.270752
88.659996	197.333008	88.670494	78.884552	96.238647
88.680000	181.500000	88.690498	64.927673	96.206543
88.699997	169.833008	88.710495	53.341873	96.174561

88.719994	166.500000	88.730492	43.630577	96.142639
88.739998	154.000000	88.750496	35.466396	96.110779
88.759995	145.167007	88.770493	28.635408	96.078918
88.779999	130.500000	88.790497	22.964867	96.047180
88.800003	127.667000	88.810501	18.312054	96.015503
88.820000	116.167000	88.830498	14.541338	95.983887
88.840004	115.667000	88.850502	11.518730	95.952271
88.860001	105.167000	88.870499	9.123060	95.920776
88.880005	111.000000	88.890503	7.239571	95.889404
88.900002	105.667000	88.910500	5.769897	95.858032
88.919998	97.666695	88.930496	4.627743	95.826782
88.940002	98.500000	88.950500	3.741652	95.795532
88.959999	99.166695	88.970497	3.054535	95.764404
88.980003	100.167000	88.990501	2.519978	95.733337
89.000000	94.666695	89.010498	2.102687	95.702332
89.019997	93.333298	89.030495	1.775067	95.671448
89.040001	101.333000	89.050499	1.516323	95.640564
89.059998	100.833000	89.070496	1.311122	95.609802
89.080002	107.667000	89.090500	1.147738	95.579102
89.099998	94.500000	89.110497	1.017721	95.548523
89.119995	98.666695	89.130493	0.914670	95.518066
89.139999	94.500000	89.150497	0.833922	95.487610
89.159996	97.500000	89.170494	0.772238	95.457275
89.180000	95.500000	89.190498	0.727410	95.427002
89.199997	98.000000	89.210495	0.698440	95.396790
89.219994	100.167000	89.230492	0.685584	95.366699
89.239998	97.333298	89.250496	0.690845	95.336670
89.259995	92.333298	89.270493	0.718654	95.306763
89.279999	95.833298	89.290497	0.776952	95.276978
89.300003	92.166695	89.310501	0.878585	95.247192
89.320000	97.166695	89.330498	1.043043	95.217529
89.340004	103.667000	89.350502	1.298670	95.187988
89.360001	90.500000	89.370499	1.683722	95.158508
89.380005	93.000000	89.390503	2.247435	95.129150
89.400002	100.500000	89.410500	3.045664	95.099915
89.419998	94.500000	89.430496	4.133101	95.070679
89.440002	100.500000	89.450500	5.544602	95.041626
89.459999	96.333298	89.470497	7.263567	95.012695

89.480003	95.166695	89.490501	9.189901	94.983826
89.500000	102.000000	89.510498	11.109290	94.955078
89.519997	97.666695	89.530495	12.712472	94.926392
89.540001	102.833000	89.550499	13.672668	94.897827
89.559998	106.500000	89.570496	13.776774	94.869385
89.580002	94.833298	89.590500	13.035451	94.840942
89.599998	96.666695	89.610497	11.681586	94.812744
89.619995	99.666695	89.630493	10.050324	94.784546
89.639999	95.166695	89.650497	8.433043	94.756592
89.659996	90.500000	89.670494	7.000206	94.728638
89.680000	99.166695	89.690498	5.802870	94.700806
89.699997	90.333298	89.710495	4.825062	94.673096
89.719994	89.000000	89.730492	4.026340	94.645508
89.739998	83.500000	89.750496	3.369429	94.618042
89.759995	90.166695	89.770485	2.829547	94.590759
89.779999	93.833298	89.790489	2.391745	94.563477
89.800003	96.000000	89.810493	2.050786	94.536377
89.820000	87.000000	89.830490	1.806897	94.509338
89.840004	90.000000	89.850494	1.664163	94.482483
89.860001	88.000000	89.870491	1.629719	94.455688
89.880005	86.666695	89.890495	1.711336	94.429077
89.900002	86.500000	89.910492	1.913644	94.402588
89.919998	90.666695	89.930489	2.231706	94.376099
89.940002	86.333298	89.950493	2.642204	94.349854
89.959999	91.833298	89.970490	3.094541	94.323730
89.980003	88.500000	89.990494	3.511035	94.297668
90.000000	88.333298	90.010490	3.799967	94.271790
90.019997	95.666695	90.030487	3.887382	94.245972
90.040001	89.833298	90.050491	3.749598	94.220337
90.059998	94.166695	90.070488	3.425899	94.194824
90.080002	90.333298	90.090492	2.997259	94.169495
90.099998	90.500000	90.110489	2.548331	94.144287
90.119995	88.000000	90.130486	2.136814	94.119141
90.139999	89.333298	90.150490	1.787182	94.094116
90.159996	91.000000	90.170486	1.500625	94.069336
90.180000	88.333298	90.190491	1.267300	94.044617
90.199997	92.000000	90.210487	1.076252	94.020020
90.219994	84.166695	90.230484	0.918592	93.995605

90.239998	94.166695	90.250488	0.788391	93.971313
90.259995	87.333298	90.270485	0.682032	93.947144
90.279999	81.333298	90.290489	0.596976	93.923096
90.300003	88.000000	90.310493	0.531425	93.899292
90.320000	92.500000	90.330490	0.483825	93.875549
90.340004	88.166695	90.350494	0.452715	93.851929
90.360001	84.833298	90.370491	0.436846	93.828491
90.380005	84.500000	90.390495	0.435123	93.805176
90.400002	83.666695	90.410492	0.446711	93.782043
90.419998	92.666695	90.430489	0.471050	93.759033
90.440002	85.333298	90.450493	0.507896	93.736206
90.459999	84.000000	90.470490	0.557256	93.713501
90.480003	85.333298	90.490494	0.619496	93.690918
90.500000	85.166695	90.510490	0.695217	93.668518
90.519997	85.500000	90.530487	0.785499	93.646301
90.540001	90.166695	90.550491	0.892070	93.624146
90.559998	88.833298	90.570488	1.017596	93.602234
90.580002	92.833298	90.590492	1.166769	93.580444
90.599998	98.500000	90.610489	1.347247	93.558838
90.619995	91.000000	90.630486	1.572126	93.537354
90.639999	89.500000	90.650490	1.863025	93.515991
90.659996	100.000000	90.670486	2.254251	93.494873
90.680000	92.000000	90.690491	2.799933	93.473877
90.699997	94.500000	90.710487	3.580769	93.453003
90.719994	98.166695	90.730484	4.714711	93.432312
90.739998	93.333298	90.750488	6.366392	93.411804
90.759995	106.000000	90.770485	8.750784	93.391418
90.779999	102.000000	90.790489	12.135192	93.371216
90.800003	111.833000	90.810493	16.810652	93.351196
90.820000	113.000000	90.830490	23.035427	93.331360
90.840004	125.667000	90.850494	30.938625	93.311646
90.860001	136.167007	90.870491	40.345806	93.292114
90.880005	150.500000	90.890495	50.629627	93.272766
90.900002	155.000000	90.910492	60.587795	93.253540
90.919998	155.833008	90.930489	68.599808	93.234497
90.940002	160.667007	90.950493	73.063217	93.215637
90.959999	156.500000	90.970490	73.045029	93.196960
90.980003	167.833008	90.990494	68.760513	93.178467

91.000000	150.000000	91.010490	61.483231	93.160095
91.019997	140.833008	91.030487	52.917297	93.141907
91.040001	142.500000	91.050491	44.499493	93.123901
91.059998	128.167007	91.070488	37.053986	93.106079
91.080002	127.333000	91.090492	30.816059	93.088501
91.099998	111.167000	91.110489	25.693264	93.070984
91.119995	115.833000	91.130486	21.471773	93.053711
91.139999	114.500000	91.150490	17.951748	93.036621
91.159996	112.000000	91.170486	14.991600	93.019592
91.180000	104.833000	91.190491	12.496232	93.002869
91.199997	113.167000	91.210487	10.409409	92.986328
91.219994	104.333000	91.230484	8.691555	92.969910
91.239998	96.166695	91.250488	7.314375	92.953674
91.259995	98.666695	91.270485	6.258871	92.937622
91.279999	97.666695	91.290489	5.513259	92.921814
91.300003	93.000000	91.310493	5.079880	92.906128
91.320000	95.000000	91.330490	4.978300	92.890625
91.340004	95.500000	91.350494	5.250425	92.875366
91.360001	91.500000	91.370491	5.963365	92.860229
91.380005	95.833298	91.390495	7.209510	92.845276
91.400002	98.500000	91.410492	9.096741	92.830566
91.419998	108.000000	91.430489	11.734114	92.816040
91.440002	100.167000	91.450493	15.202822	92.801636
91.459999	112.000000	91.470490	19.509264	92.787476
91.480003	117.500000	91.490494	24.541504	92.773499
91.500000	122.333000	91.510490	29.995695	92.759644
91.519997	118.833000	91.530487	35.340782	92.746094
91.540001	125.333000	91.550491	39.827259	92.732666
91.559998	129.167007	91.570488	42.628426	92.719482
91.580002	137.667007	91.590492	43.137913	92.706421
91.599998	130.833008	91.610489	41.257347	92.693604
91.619995	124.167000	91.630486	37.483284	92.680969
91.639999	129.167007	91.650490	32.680496	92.668457
91.659996	121.667000	91.670486	27.714127	92.656250
91.680000	115.333000	91.690491	23.161360	92.644165
91.699997	111.500000	91.710487	19.268997	92.632324
91.719994	113.000000	91.730484	16.039845	92.620728
91.739998	102.667000	91.750488	13.370248	92.609253

91.759995	108.333000	91.770485	11.143351	92.597961
91.779999	100.000000	91.790489	9.262367	92.586853
91.800003	99.166695	91.810493	7.663283	92.575989
91.820000	96.000000	91.830490	6.304585	92.565308
91.840004	96.666695	91.850494	5.156205	92.554871
91.860001	97.000000	91.870491	4.196088	92.544617
91.880005	93.166695	91.890495	3.402627	92.534546
91.900002	90.500000	91.910492	2.756112	92.524658
91.919998	90.166695	91.930489	2.236226	92.515015
91.940002	88.500000	91.950493	1.823714	92.505554
91.959999	86.333298	91.970490	1.501166	92.496338
91.980003	91.000000	91.990494	1.252469	92.487305
92.000000	86.833298	92.010490	1.064163	92.478394
92.019997	91.500000	92.030487	0.924824	92.469788
92.040001	91.500000	92.050491	0.825491	92.461304
92.059998	93.166695	92.070488	0.759731	92.453125
92.080002	95.333298	92.090492	0.723520	92.445068
92.099998	87.333298	92.110489	0.715728	92.437256
92.119995	93.333298	92.130486	0.738515	92.429688
92.139999	89.666695	92.150490	0.798267	92.422241
92.159996	93.166695	92.170486	0.906656	92.415039
92.180000	91.833298	92.190491	1.082333	92.408081
92.199997	95.333298	92.210487	1.352135	92.401245
92.219994	89.666695	92.230484	1.752813	92.394714
92.239998	95.833298	92.250488	2.330951	92.388306
92.259995	100.833000	92.270485	3.139739	92.382202
92.279999	99.000000	92.290489	4.233482	92.376221
92.300003	95.333298	92.310493	5.652264	92.370483
92.320000	98.000000	92.330490	7.401460	92.364990
92.340004	99.000000	92.350494	9.428495	92.359680
92.360001	111.167000	92.370491	11.594860	92.354614
92.380005	106.000000	92.390495	13.674905	92.349731
92.400002	107.167000	92.410492	15.374146	92.345032
92.419998	116.833000	92.430489	16.400698	92.340576
92.440002	103.000000	92.450493	16.558611	92.336365
92.459999	98.166695	92.470490	15.834836	92.332275
92.480003	101.500000	92.490494	14.416318	92.328491
92.500000	102.167000	92.510490	12.615819	92.324890

92.519997	96.666695	92.530487	10.743980	92.321533
92.540001	106.000000	92.550491	9.015555	92.318359
92.559998	98.333298	92.570488	7.527628	92.315308
92.580002	91.666695	92.590492	6.286274	92.312561
92.599998	91.666695	92.610489	5.257163	92.310059
92.619995	90.833298	92.630486	4.396116	92.307739
92.639999	94.666695	92.650490	3.666888	92.305664
92.659996	94.333298	92.670486	3.045103	92.303772
92.680000	90.500000	92.690491	2.514101	92.302063
92.699997	96.333298	92.710487	2.063364	92.300659
92.719994	88.000000	92.730484	1.684155	92.299316
92.739998	84.166695	92.750488	1.368773	92.298340
92.759995	92.666695	92.770485	1.110021	92.297546
92.779999	89.500000	92.790489	0.900308	92.296936
92.800003	89.666695	92.810493	0.732629	92.296570
92.820000	84.333298	92.830490	0.600291	92.296387
92.840004	86.666695	92.850494	0.497118	92.296448
92.860001	83.333298	92.870491	0.417873	92.296753
92.880005	85.166695	92.890495	0.357988	92.297241
92.900002	88.500000	92.910492	0.313750	92.297974
92.919998	88.500000	92.930489	0.282190	92.298889
92.940002	93.333298	92.950493	0.261023	92.300049
92.959999	85.833298	92.970490	0.248432	92.301392
92.980003	85.166695	92.990494	0.243084	92.302979
93.000000	92.000000	93.010490	0.244041	92.304810
93.019997	90.666695	93.030487	0.250685	92.306824
93.040001	89.666695	93.050491	0.262720	92.309021
93.059998	90.166695	93.070488	0.280179	92.311462
93.080002	89.666695	93.090492	0.303579	92.314209
93.099998	88.166695	93.110489	0.334086	92.317017
93.119995	89.500000	93.130486	0.373935	92.320190
93.139999	96.333298	93.150490	0.426989	92.323486
93.159996	86.166695	93.170486	0.499475	92.327026
93.180000	87.000000	93.190491	0.601274	92.330811
93.199997	96.166695	93.210487	0.747126	92.334778
93.219994	101.667000	93.230484	0.958600	92.338989
93.239998	100.333000	93.250488	1.265843	92.343384
93.259995	87.500000	93.270485	1.708582	92.348022

93.279999	99.000000	93.290489	2.337120	92.352844
93.300003	95.666695	93.310493	3.208515	92.357910
93.320000	87.000000	93.330490	4.378768	92.363220
93.340004	95.500000	93.350494	5.888953	92.368713
93.360001	98.000000	93.370491	7.736948	92.374451
93.380005	95.833298	93.390495	9.850634	92.380371
93.400002	103.333000	93.410492	12.055458	92.386536
93.419998	108.833000	93.430489	14.078610	92.392944
93.440002	104.500000	93.450493	15.594251	92.399536
93.459999	111.500000	93.470490	16.322397	92.406311
93.480003	108.000000	93.490494	16.145533	92.413330
93.500000	108.667000	93.510490	15.161501	92.420532
93.519997	104.500000	93.530487	13.635931	92.427979
93.540001	90.833298	93.550491	11.882144	92.435730
93.559998	96.500000	93.570488	10.153579	92.443604
93.580002	101.667000	93.590492	8.593024	92.451660
93.599998	96.000000	93.610489	7.251235	92.460022
93.619995	87.333298	93.630486	6.120491	92.468506
93.639999	90.666695	93.650490	5.170440	92.477295
93.659996	99.666695	93.670486	4.369036	92.486267
93.680000	87.333298	93.690491	3.688803	92.495422
93.699997	97.833298	93.710487	3.110987	92.504822
93.719994	95.666695	93.730484	2.622277	92.514404
93.739998	89.166695	93.750488	2.213665	92.524292
93.759995	87.166695	93.770485	1.879189	92.534302
93.779999	87.166695	93.790489	1.614181	92.544556
93.800003	86.666695	93.810493	1.416321	92.554993
93.820000	86.000000	93.830490	1.285878	92.565674
93.840004	85.833298	93.850494	1.226867	92.576538
93.860001	84.333298	93.870491	1.248859	92.587646
93.880005	88.166695	93.890495	1.368345	92.598999
93.900002	87.666695	93.910492	1.609998	92.610474
93.919998	98.500000	93.930489	2.007308	92.622192
93.940002	98.000000	93.950493	2.601220	92.634155
93.959999	101.167000	93.970490	3.434118	92.646301
93.980003	94.166695	93.990494	4.541046	92.658630
94.000000	93.000000	94.010490	5.929424	92.671265
94.019997	95.500000	94.030487	7.557784	92.684021

94.040001	98.333298	94.050491	9.310975	92.697021
94.059998	98.833298	94.070488	10.988276	92.710205
94.080002	102.833000	94.090492	12.332121	92.723572
94.099998	98.666695	94.110489	13.091828	92.737183
94.119995	97.000000	94.130486	13.121418	92.751038
94.139999	89.500000	94.150490	12.444968	92.765076
94.159996	94.500000	94.170486	11.250367	92.779297
94.180000	100.833000	94.190491	9.802756	92.793762
94.199997	97.000000	94.210487	8.343174	92.808350
94.219994	94.166695	94.230484	7.021676	92.823242
94.239998	96.500000	94.250488	5.894724	92.838257
94.259995	91.000000	94.270485	4.957590	92.853516
94.279999	95.833298	94.290489	4.177481	92.868958
94.300003	99.166695	94.310493	3.520188	92.884644
94.320000	94.666695	94.330490	2.959024	92.900452
94.340004	91.166695	94.350494	2.475757	92.916504
94.360001	95.166695	94.370491	2.059745	92.932739
94.380005	97.666695	94.390495	1.703570	92.949219
94.400002	95.166695	94.410492	1.402188	92.965881
94.419998	92.000000	94.430489	1.150704	92.982788
94.440002	88.000000	94.450493	0.944378	92.999817
94.459999	89.166695	94.470490	0.778727	93.017090
94.480003	89.833298	94.490494	0.649140	93.034485
94.500000	90.166695	94.510490	0.551547	93.052124
94.519997	85.833298	94.530487	0.482087	93.069946
94.540001	86.333298	94.550491	0.437199	93.088013
94.559998	84.500000	94.570488	0.413238	93.106262
94.580002	86.166695	94.590492	0.406006	93.124695
94.599998	91.500000	94.610489	0.410327	93.143250
94.619995	84.000000	94.630486	0.419884	93.162109
94.639999	81.166695	94.650490	0.427930	93.181030
94.659996	87.333298	94.670486	0.428685	93.200256
94.680000	89.333298	94.690491	0.419150	93.219666
94.699997	88.166695	94.710487	0.400154	93.239197
94.719994	91.333298	94.730484	0.375836	93.258972
94.739998	85.333298	94.750488	0.351860	93.278931
94.759995	83.333298	94.770485	0.333576	93.299072
94.779999	94.833298	94.790489	0.325074	93.319336

94.800003	89.666695	94.810493	0.329463	93.339844
94.820000	93.000000	94.830490	0.349650	93.360535
94.840004	89.333298	94.850494	0.389157	93.381409
94.860001	81.166695	94.870491	0.452410	93.402466
94.880005	89.500000	94.890495	0.544672	93.423706
94.900002	92.000000	94.910492	0.670960	93.445129
94.919998	89.666695	94.930489	0.834581	93.466675
94.940002	85.500000	94.950493	1.034570	93.488464
94.959999	90.166695	94.970490	1.262400	93.510437
94.980003	89.500000	94.990494	1.500205	93.532593
95.000000	91.666695	95.010490	1.720432	93.554871
95.019997	92.500000	95.030487	1.891499	93.577393
95.040001	90.666695	95.050491	1.987231	93.599976
95.059998	95.500000	95.070488	1.997589	93.622803
95.080002	96.333298	95.090492	1.934054	93.645874
95.099998	92.000000	95.110489	1.825118	93.669006
95.119995	96.500000	95.130486	1.704622	93.692383
95.139999	89.000000	95.150490	1.601042	93.715942
95.159996	92.833298	95.170486	1.533013	93.739563
95.180000	93.833298	95.190491	1.511245	93.763428
95.199997	91.333298	95.210487	1.544222	93.787476
95.219994	100.000000	95.230484	1.643835	93.811707
95.239998	95.333298	95.250488	1.830144	93.836060
95.259995	95.000000	95.270485	2.134590	93.860596
95.279999	98.333298	95.290489	2.603479	93.885254
95.300003	94.333298	95.310493	3.299331	93.910156
95.320000	92.000000	95.330490	4.301220	93.935181
95.340004	99.166695	95.350494	5.702963	93.960327
95.360001	100.667000	95.370491	7.599974	93.985657
95.380005	101.333000	95.390495	10.073140	94.011108
95.400002	102.667000	95.410492	13.149128	94.036804
95.419998	115.000000	95.430489	16.761387	94.062622
95.440002	110.667000	95.450493	20.703857	94.088562
95.459999	126.000000	95.470490	24.608696	94.114685
95.480003	123.833000	95.490494	28.002792	94.140991
95.500000	122.667000	95.510490	30.425325	94.167358
95.519997	124.000000	95.530487	31.616341	94.193970
95.540001	132.500000	95.550491	31.645420	94.220703

95.559998	120.167000	95.570488	30.902624	94.247559
95.580002	117.667000	95.590492	29.925735	94.274536
95.599998	127.333000	95.610489	29.164591	94.301758
95.619995	121.167000	95.630486	28.806522	94.329102
95.639999	114.667000	95.650490	28.738327	94.356567
95.659996	131.333008	95.670486	28.629162	94.384155
95.680000	127.167000	95.690491	28.088612	94.411865
95.699997	121.667000	95.710487	26.840353	94.439697
95.719994	114.333000	95.730484	24.837904	94.467773
95.739998	125.833000	95.750488	22.269590	94.495911
95.759995	116.833000	95.770485	19.460522	94.524170
95.779999	119.667000	95.790489	16.725716	94.552673
95.800003	119.500000	95.810493	14.281235	94.581177
95.820000	106.000000	95.830490	12.220929	94.609985
95.840004	110.500000	95.850494	10.548862	94.638794
95.860001	100.000000	95.870491	9.233405	94.667725
95.880005	106.167000	95.890495	8.239752	94.696838
95.900002	106.333000	95.910492	7.554344	94.726074
95.919998	104.500000	95.930489	7.189419	94.755432
95.940002	106.333000	95.950493	7.185564	94.784912
95.959999	108.333000	95.970490	7.608893	94.814575
95.980003	110.167000	95.990494	8.546306	94.844238
96.000000	108.667000	96.010490	10.093607	94.874084
96.019997	119.500000	96.030487	12.337684	94.904053
96.040001	121.833000	96.050491	15.323371	94.934204
96.059998	117.833000	96.070488	19.001162	94.964355
96.080002	127.833000	96.090492	23.181660	94.994690
96.099998	136.667007	96.110489	27.486425	95.025146
96.119995	140.000000	96.130486	31.374048	95.055664
96.139999	146.000000	96.150490	34.238018	95.086426
96.159996	142.500000	96.170486	35.591782	95.117126
96.180000	144.667007	96.190491	35.273159	95.148071
96.199997	140.167007	96.210487	33.531368	95.179077
96.219994	128.833008	96.230484	30.940723	95.210144
96.239998	147.333008	96.250488	28.186264	95.241333
96.259995	138.500000	96.270485	25.863428	95.272705
96.279999	138.667007	96.290489	24.377489	95.304138
96.300003	128.000000	96.310493	23.971458	95.335632

96.320000	136.833008	96.330490	24.794901	95.367310
96.340004	138.667007	96.350494	26.966780	95.399048
96.360001	144.833008	96.370491	30.586912	95.430847
96.380005	152.833008	96.390495	35.710861	95.462769
96.400002	157.167007	96.410492	42.278702	95.494751
96.419998	158.333008	96.430489	50.072571	95.526855
96.440002	176.667007	96.450493	58.709328	95.559082
96.459999	179.000000	96.470490	67.715645	95.591370
96.480003	198.500000	96.490494	76.720070	95.623779
96.500000	204.667007	96.510490	85.584145	95.656250
96.519997	218.000000	96.530487	94.429955	95.688782
96.540001	222.667007	96.550491	103.405106	95.721436
96.559998	238.167007	96.570488	112.330765	95.754150
96.580002	231.167007	96.590492	120.517387	95.786926
96.599998	242.667007	96.610489	126.862923	95.819824
96.619995	251.500000	96.630486	130.292221	95.852783
96.639999	244.167007	96.650490	130.182632	95.885864
96.659996	247.500000	96.670486	126.540901	95.918945
96.680000	252.167007	96.690491	119.847023	95.952148
96.699997	243.500000	96.710487	110.795364	95.985413
96.719994	226.167007	96.730484	100.126106	96.018738
96.739998	220.667007	96.750488	88.610954	96.052124
96.759995	202.167007	96.770485	77.044334	96.085571
96.779999	191.167007	96.790489	66.105217	96.119141
96.800003	180.667007	96.810493	56.261662	96.152832
96.820000	164.000000	96.830490	47.710632	96.186462
96.840004	154.167007	96.850494	40.424252	96.220215
96.860001	150.833008	96.870491	34.272194	96.254028
96.880005	140.333008	96.890495	29.087996	96.287842
96.900002	139.500000	96.910492	24.738676	96.321777
96.919998	130.667007	96.930489	21.126968	96.355713
96.940002	146.000000	96.950493	18.196363	96.389709
96.959999	128.000000	96.970490	15.924200	96.423828
96.980003	126.167000	96.990494	14.305010	96.457947
97.000000	115.167000	97.010490	13.348870	96.492126
97.019997	115.333000	97.030487	13.067308	96.526306
97.040001	123.167000	97.050491	13.464639	96.560547
97.059998	119.000000	97.070488	14.520055	96.594910

97.080002	117.500000	97.090492	16.167768	96.629272
97.099998	122.833000	97.110489	18.268501	96.663696
97.119995	123.167000	97.130486	20.591143	96.698120
97.139999	126.667000	97.150490	22.807360	96.732544
97.159996	129.667007	97.170486	24.525467	96.767090
97.180000	123.333000	97.190491	25.385693	96.801636
97.199997	121.000000	97.210487	25.176573	96.836243
97.219994	129.333008	97.230484	23.927637	96.870850
97.239998	125.500000	97.250488	21.901102	96.905457
97.259995	117.167000	97.270485	19.489006	96.940186
97.279999	114.333000	97.290489	17.064552	96.974854
97.300003	113.667000	97.310493	14.889994	97.009583
97.320000	107.333000	97.330490	13.094725	97.044312
97.340004	116.000000	97.350494	11.712893	97.079041
97.360001	111.500000	97.370491	10.744190	97.113892
97.380005	107.000000	97.390495	10.194866	97.148682
97.400002	109.500000	97.410492	10.106481	97.183533
97.419998	111.500000	97.430489	10.564023	97.218323
97.440002	114.667000	97.450493	11.696932	97.253174
97.459999	105.333000	97.470490	13.667736	97.288086
97.480003	119.333000	97.490494	16.655859	97.322876
97.500000	124.667000	97.510490	20.814899	97.357788
97.519997	125.167000	97.530487	26.220804	97.392700
97.540001	135.000000	97.550499	32.791946	97.427612
97.559998	143.333008	97.570496	40.178654	97.462524
97.580002	145.000000	97.590500	47.734375	97.497375
97.599998	152.833008	97.610497	54.510235	97.532349
97.619995	152.333008	97.630493	59.445515	97.567200
97.639999	153.167007	97.650497	61.661022	97.602051
97.659996	153.333008	97.670494	60.788498	97.636963
97.680000	151.000000	97.690498	57.139435	97.671875
97.699997	142.667007	97.710495	51.584972	97.706726
97.719994	146.333008	97.730492	45.199596	97.741577
97.739998	132.000000	97.750496	38.897995	97.776489
97.759995	120.167000	97.770493	33.244705	97.811279
97.779999	129.333008	97.790497	28.441710	97.846069
97.800003	122.500000	97.810501	24.465441	97.880920
97.820000	116.833000	97.830498	21.195135	97.915710

97.840004	117.500000	97.850502	18.504211	97.950439
97.860001	118.000000	97.870499	16.312412	97.985229
97.880005	112.667000	97.890503	14.587208	98.019958
97.900002	110.000000	97.910500	13.344341	98.054688
97.919998	107.167000	97.930496	12.631895	98.089355
97.940002	111.167000	97.950500	12.521630	98.124023
97.959999	115.667000	97.970497	13.095028	98.158569
97.980003	118.667000	97.990501	14.425105	98.193176
98.000000	110.667000	98.010498	16.546549	98.227722
98.019997	114.333000	98.030495	19.419256	98.262207
98.040001	123.833000	98.050499	22.882261	98.296692
98.059998	129.167007	98.070496	26.612143	98.331116
98.080002	124.000000	98.090500	30.133759	98.365540
98.099998	135.000000	98.110497	32.879311	98.399902
98.119995	141.833008	98.130493	34.342484	98.434204
98.139999	143.500000	98.150497	34.247681	98.468445
98.159996	138.333008	98.170494	32.667358	98.502625
98.180000	140.167007	98.190498	29.997187	98.536865
98.199997	131.167007	98.210495	26.803596	98.570923
98.219994	124.333000	98.230492	23.622154	98.604980
98.239998	116.333000	98.250496	20.832441	98.638977
98.259995	125.333000	98.270493	18.641174	98.672974
98.279999	121.500000	98.290497	17.131557	98.706848
98.300003	124.667000	98.310501	16.347460	98.740662
98.320000	118.333000	98.330498	16.349783	98.774414
98.340004	119.500000	98.350502	17.247885	98.808167
98.360001	135.167007	98.370499	19.200775	98.841736
98.380005	133.333008	98.390503	22.398314	98.875366
98.400002	131.333008	98.410500	27.009422	98.908875
98.419998	140.000000	98.430496	33.118298	98.942322
98.440002	145.667007	98.450500	40.627731	98.975647
98.459999	153.333008	98.470497	49.144341	99.008972
98.480003	161.333008	98.490501	57.927803	99.042236
98.500000	169.667007	98.510498	65.891914	99.075317
98.519997	177.667007	98.530495	71.811508	99.108398
98.540001	184.667007	98.550499	74.647980	99.141357
98.559998	184.667007	98.570496	73.925850	99.174255
98.580002	175.500000	98.590500	69.946297	99.207092

98.599998	170.333008	98.610497	63.680550	99.239868
98.619995	162.500000	98.630493	56.383095	99.272461
98.639999	153.333008	98.650490	49.167778	99.304993
98.659996	153.167007	98.670486	42.744682	99.337463
98.680000	137.500000	98.690491	37.385433	99.369873
98.699997	136.167007	98.710487	33.047989	99.402100
98.719994	129.167007	98.730484	29.516775	99.434326
98.739998	128.167007	98.750488	26.529203	99.466431
98.759995	126.167000	98.770485	23.850924	99.498413
98.779999	132.667007	98.790489	21.309040	99.530273
98.800003	112.000000	98.810493	18.819040	99.562134
98.820000	117.833000	98.830490	16.379620	99.593750
98.840004	112.667000	98.850494	14.045320	99.625366
98.860001	110.167000	98.870491	11.894280	99.656860
98.880005	113.667000	98.890495	9.986012	99.688171
98.900002	107.167000	98.910492	8.349846	99.719482
98.919998	102.000000	98.930489	6.980229	99.750610
98.940002	104.500000	98.950493	5.850543	99.781677
98.959999	103.500000	98.970490	4.926636	99.812561
98.980003	97.666695	98.990494	4.172982	99.843323
99.000000	101.667000	99.010490	3.560000	99.874023
99.019997	96.500000	99.030487	3.063011	99.904602
99.040001	103.833000	99.050491	2.662759	99.935059
99.059998	99.000000	99.070488	2.344564	99.965332
99.080002	99.166695	99.090492	2.096272	99.995544
99.099998	104.500000	99.110489	1.908420	100.025635
99.119995	100.167000	99.130486	1.772983	100.055542
99.139999	95.000000	99.150490	1.683328	100.085388
99.159996	91.833298	99.170486	1.634241	100.115051
99.180000	97.000000	99.190491	1.621692	100.144592
99.199997	93.666695	99.210487	1.643285	100.174072
99.219994	96.333298	99.230484	1.698730	100.203247
99.239998	97.500000	99.250488	1.790833	100.232483
99.259995	100.667000	99.270485	1.926743	100.261475
99.279999	107.000000	99.290489	2.120322	100.290344
99.300003	96.833298	99.310493	2.394722	100.319092
99.320000	93.666695	99.330490	2.786258	100.347656
99.340004	104.000000	99.350494	3.349901	100.376038

99.360001	107.000000	99.370491	4.163838	100.404358
99.380005	109.000000	99.390495	5.337553	100.432495
99.400002	111.167000	99.410492	7.014649	100.460510
99.419998	108.667000	99.430489	9.378308	100.488342
99.440002	111.500000	99.450493	12.647422	100.516052
99.459999	115.333000	99.470490	17.056158	100.543579
99.480003	122.167000	99.490494	22.829758	100.570923
99.500000	133.333008	99.510490	30.111221	100.598206
99.519997	135.833008	99.530487	38.888195	100.625244
99.540001	142.000000	99.550491	48.883167	100.652161
99.559998	163.833008	99.570488	59.446476	100.678894
99.580002	178.000000	99.590492	69.559639	100.705505
99.599998	181.833008	99.610489	77.919159	100.731934
99.619995	202.833008	99.630486	83.250580	100.758179
99.639999	217.667007	99.650490	84.689430	100.784241
99.659996	198.667007	99.670486	82.118912	100.810181
99.680000	184.500000	99.690491	76.239212	100.835938
99.699997	181.500000	99.710487	68.309311	100.861511
99.719994	162.500000	99.730484	59.678474	100.886902
99.739998	157.833008	99.750488	51.396164	100.912170
99.759995	149.500000	99.770485	44.053173	100.937195
99.779999	142.667007	99.790489	37.811630	100.962158
99.800003	130.167007	99.810493	32.585327	100.986816
99.820000	129.333008	99.830490	28.187258	101.011353
99.840004	119.833000	99.850494	24.428190	101.035706
99.860001	128.167007	99.870491	21.171329	101.059875
99.880005	123.833000	99.890495	18.326149	101.083862
99.900002	119.333000	99.910492	15.847336	101.107605
99.919998	120.000000	99.930489	13.712720	101.131226
99.940002	126.667000	99.950493	11.916701	101.154663
99.959999	115.167000	99.970490	10.466585	101.177979
99.980003	107.333000	99.990494	9.377905	101.200989
100.000000	113.000000	100.010490	8.682096	101.223877
100.019997	111.333000	100.030487	8.428823	101.246521
100.040001	110.667000	100.050491	8.694330	101.269043
100.059998	110.333000	100.070488	9.585935	101.291260
100.080002	113.333000	100.090492	11.245725	101.313354
100.099998	116.500000	100.110489	13.843354	101.335205

100.119995	113.000000	100.130486	17.564024	101.356934
100.139999	116.000000	100.150490	22.575369	101.378418
100.159996	130.333008	100.170486	28.963934	101.399780
100.180000	139.500000	100.190491	36.670624	101.420776
100.199997	150.000000	100.210487	45.379974	101.441711
100.219994	163.167007	100.230484	54.467445	101.462402
100.239998	163.500000	100.250488	62.989838	101.482849
100.259995	173.167007	100.270485	69.801079	101.503174
100.279999	178.500000	100.290489	73.842964	101.523254
100.300003	187.333008	100.310493	74.467224	101.543091
100.320000	181.500000	100.330490	71.702393	101.562744
100.340004	175.000000	100.350494	66.251450	101.582153
100.360001	164.500000	100.370491	59.236340	101.601379
100.380005	158.333008	100.390495	51.792038	101.620483
100.400002	150.667007	100.410492	44.776768	101.639221
100.419998	142.833008	100.430489	38.642132	101.657837
100.440002	138.833008	100.450493	33.507893	101.676208
100.459999	129.667007	100.470490	29.308187	101.694336
100.480003	118.667000	100.490494	25.910273	101.712280
100.500000	126.500000	100.510490	23.210125	101.729980
100.519997	133.000000	100.530487	21.157280	101.747498
100.540001	127.833000	100.550491	19.762957	101.764771
100.559998	119.000000	100.570488	19.087965	101.781860
100.580002	125.667000	100.590492	19.219929	101.798706
100.599998	132.167007	100.610489	20.250525	101.815308
100.619995	128.167007	100.630486	22.244081	101.831665
100.639999	132.667007	100.650490	25.200880	101.847900
100.659996	127.167000	100.670486	29.010212	101.863770
100.680000	131.000000	100.690491	33.425266	101.879517
100.699997	145.500000	100.710487	38.046036	101.895020
100.719994	147.167007	100.730484	42.373192	101.910278
100.739998	147.000000	100.750488	45.896801	101.925293
100.759995	149.000000	100.770485	48.210270	101.940125
100.779999	150.167007	100.790489	49.104599	101.954712
100.800003	154.000000	100.810493	48.573120	101.968994
100.820000	142.000000	100.830490	46.763241	101.983093
100.840004	149.833008	100.850494	43.906322	101.996948
100.860001	137.333008	100.870491	40.287422	102.010559

100.880005	129.333008	100.890495	36.223515	102.023987
100.900002	132.333008	100.910492	32.048450	102.037170
100.919998	123.167000	100.930489	28.054836	102.050110
100.940002	125.000000	100.950493	24.452549	102.062805
100.959999	117.667000	100.970490	21.356522	102.075195
100.980003	112.500000	100.990494	18.796715	102.087463
101.000000	118.000000	101.010490	16.765003	102.099426
101.019997	112.333000	101.030487	15.242546	102.111206
101.040001	115.333000	101.050491	14.224290	102.122681
101.059998	111.833000	101.070488	13.723927	102.133972
101.080002	108.333000	101.090492	13.763407	102.145020
101.099998	106.000000	101.110489	14.356527	102.155762
101.119995	117.833000	101.130486	15.483746	102.166260
101.139999	113.833000	101.150490	17.064997	102.176575
101.159996	118.500000	101.170486	18.933138	102.186646
101.180000	122.833000	101.190491	20.831186	102.196411
101.199997	109.333000	101.210487	22.431847	102.205994
101.219994	123.333000	101.230484	23.407320	102.215332
101.239998	120.000000	101.250488	23.518557	102.224365
101.259995	122.833000	101.270485	22.698696	102.233215
101.279999	118.500000	101.290489	21.078699	102.241760
101.300003	113.667000	101.310493	18.937719	102.250122
101.320000	108.333000	101.330490	16.600306	102.258179
101.340004	110.500000	101.350494	14.333531	102.266113
101.360001	108.667000	101.370491	12.301051	102.273621
101.380005	108.000000	101.390495	10.558146	102.281006
101.400002	101.167000	101.410492	9.091596	102.288086
101.419998	105.500000	101.430489	7.853765	102.294922
101.440002	96.166695	101.450493	6.793168	102.301514
101.459999	90.333298	101.470490	5.869330	102.307861
101.480003	105.167000	101.490494	5.053498	102.313965
101.500000	104.500000	101.510490	4.329399	102.319824
101.519997	103.500000	101.530487	3.687144	102.325378
101.540001	99.166695	101.550491	3.120779	102.330688
101.559998	98.333298	101.570488	2.626341	102.335815
101.580002	102.167000	101.590492	2.199090	102.340637
101.599998	99.000000	101.610489	1.834340	102.345215
101.619995	96.500000	101.630486	1.526351	102.349609

101.639999	95.500000	101.650490	1.268929	102.353638
101.659996	96.666695	101.670486	1.056025	102.357483
101.680000	102.500000	101.690491	0.881345	102.361084
101.699997	95.500000	101.710487	0.739246	102.364380
101.719994	97.500000	101.730484	0.624388	102.367493
101.739998	95.500000	101.750488	0.532071	102.370361
101.759995	90.333298	101.770485	0.458333	102.372864
101.779999	90.166695	101.790489	0.399705	102.375244
101.800003	92.000000	101.810493	0.353386	102.377319
101.820000	91.833298	101.830490	0.317082	102.379150
101.840004	95.166695	101.850494	0.288916	102.380737
101.860001	92.166695	101.870491	0.267453	102.382019
101.880005	102.833000	101.890495	0.251544	102.383057
101.900002	97.333298	101.910492	0.240339	102.383911
101.919998	93.333298	101.930489	0.233191	102.384460
101.940002	89.500000	101.950493	0.229637	102.384766
101.959999	88.666695	101.970490	0.229362	102.384827
101.980003	96.166695	101.990494	0.232169	102.384644
102.000000	93.166695	102.010490	0.237958	102.384216
102.019997	89.666695	102.030487	0.246716	102.383545
102.040001	93.000000	102.050491	0.258497	102.382568
102.059998	91.333298	102.070488	0.273427	102.381409
102.080002	100.167000	102.090492	0.291685	102.379944
102.099998	89.500000	102.110489	0.313481	102.378235
102.119995	87.833298	102.130486	0.339093	102.376282
102.139999	91.166695	102.150490	0.368850	102.374084
102.159996	90.833298	102.170486	0.403090	102.371643
102.180000	90.833298	102.190491	0.442254	102.368958
102.199997	97.166695	102.210487	0.486792	102.366028
102.219994	99.666695	102.230484	0.537294	102.362854
102.239998	93.500000	102.250488	0.594504	102.359375
102.259995	91.166695	102.270485	0.659331	102.355713
102.279999	99.000000	102.290489	0.733136	102.351746
102.300003	86.833298	102.310493	0.817771	102.347595
102.320000	95.333298	102.330490	0.915919	102.343140
102.340004	104.167000	102.350494	1.031641	102.338501
102.360001	94.166695	102.370491	1.170661	102.333557
102.380005	96.666695	102.390495	1.341466	102.328430

102.400002	95.666695	102.410492	1.555822	102.322998
102.419998	99.666695	102.430489	1.830324	102.317383
102.440002	100.500000	102.450493	2.187577	102.311523
102.459999	96.333298	102.470490	2.656922	102.305298
102.480003	108.500000	102.490494	3.276415	102.299011
102.500000	102.500000	102.510490	4.091425	102.292358
102.519997	107.000000	102.530487	5.154595	102.285522
102.540001	108.833000	102.550491	6.521540	102.278442
102.559998	112.333000	102.570488	8.241267	102.271118
102.580002	107.167000	102.590492	10.348781	102.263550
102.599998	114.667000	102.610489	12.846010	102.255737
102.619995	116.167000	102.630486	15.695328	102.247681
102.639999	116.500000	102.650490	18.816288	102.239380
102.659996	125.667000	102.670486	22.100735	102.230835
102.680000	129.167007	102.690491	25.467915	102.222168
102.699997	131.167007	102.710487	28.921570	102.213135
102.719994	134.333008	102.730484	32.620544	102.203918
102.739998	145.833008	102.750488	36.893463	102.194458
102.759995	146.833008	102.770485	42.178738	102.184814
102.779999	152.500000	102.790489	48.911343	102.174866
102.800003	157.667007	102.810493	57.344967	102.164795
102.820000	175.000000	102.830490	67.395943	102.154358
102.840004	189.833008	102.850494	78.544281	102.143799
102.860001	189.167007	102.870491	89.778099	102.132935
102.880005	212.333008	102.890495	99.726654	102.121887
102.900002	220.167007	102.910492	106.888184	102.110657
102.919998	224.833008	102.930489	110.047089	102.099121
102.940002	218.333008	102.950493	108.649361	102.087402
102.959999	211.667007	102.970490	103.020500	102.075439
102.980003	203.833008	102.990494	94.251869	102.063354
103.000000	190.667007	103.010490	83.830475	102.050903
103.019997	184.500000	103.030487	73.152702	102.038330
103.040001	173.500000	103.050491	63.205246	102.025513
103.059998	159.500000	103.070488	54.484615	102.012512
103.080002	154.500000	103.090492	47.067348	101.999268
103.099998	141.667007	103.110489	40.808369	101.985840
103.119995	137.333008	103.130486	35.474045	101.972107
103.139999	136.167007	103.150490	30.847891	101.958252

103.159996	129.667007	103.170486	26.773531	101.944153
103.180000	133.000000	103.190491	23.145750	101.929871
103.199997	127.500000	103.210487	19.909224	101.915344
103.219994	131.000000	103.230484	17.031261	101.900635
103.239998	129.500000	103.250488	14.492305	101.885681
103.259995	122.333000	103.270485	12.278930	101.870605
103.279999	113.333000	103.290489	10.372360	101.855286
103.300003	115.833000	103.310493	8.752916	101.839722
103.320000	106.167000	103.330490	7.397124	101.824036
103.340004	110.667000	103.350494	6.277701	101.808105
103.360001	111.333000	103.370491	5.368335	101.791992
103.380005	111.833000	103.390495	4.641533	101.775696
103.400002	105.500000	103.410492	4.072481	101.759216
103.419998	106.833000	103.430489	3.637704	101.742493
103.440002	98.500000	103.450493	3.316655	101.725586
103.459999	109.167000	103.470490	3.092199	101.708557
103.480003	108.667000	103.490494	2.950165	101.691284
103.500000	104.667000	103.510490	2.880434	101.673828
103.519997	109.833000	103.530487	2.877096	101.656250
103.540001	102.833000	103.550491	2.939673	101.638367
103.559998	110.833000	103.570488	3.074438	101.620422
103.580002	108.333000	103.590492	3.296843	101.602295
103.599998	109.167000	103.610489	3.634073	101.583862
103.619995	110.500000	103.630486	4.129460	101.565369
103.639999	111.500000	103.650490	4.847396	101.546692
103.659996	109.500000	103.670486	5.877641	101.527893
103.680000	114.667000	103.690491	7.343276	101.508789
103.699997	119.333000	103.710487	9.402071	101.489563
103.719994	115.833000	103.730484	12.252026	101.470215
103.739998	120.000000	103.750488	16.127890	101.450684
103.759995	123.833000	103.770485	21.283484	101.431030
103.779999	143.333008	103.790489	27.979057	101.411133
103.800003	144.333008	103.810493	36.428673	101.391113
103.820000	155.500000	103.830490	46.745903	101.370911
103.840004	170.667007	103.850494	58.886696	101.350586
103.860001	186.667007	103.870491	72.540833	101.330078
103.880005	195.833008	103.890495	87.096687	101.309448
103.900002	222.000000	103.910492	101.562485	101.288696

103.919998	228.000000	103.930489	114.639198	101.267761
103.940002	239.667007	103.950493	124.855522	101.246643
103.959999	243.833008	103.970490	130.851135	101.225464
103.980003	243.833008	103.990494	131.777130	101.204102
104.000000	236.333008	104.010490	127.591560	101.182617
104.019997	237.500000	104.030487	119.136322	101.160950
104.040001	221.000000	104.050491	107.882141	101.139221
104.059998	201.667007	104.070488	95.491074	101.117310
104.080002	194.500000	104.090492	83.340034	101.095215
104.099998	169.833008	104.110489	72.308304	101.072998
104.119995	164.333008	104.130486	62.736401	101.050720
104.139999	155.833008	104.150490	54.590225	101.028320
104.159996	152.667007	104.170486	47.655720	101.005798
104.180000	136.500000	104.190491	41.666405	100.983154
104.199997	148.167007	104.210487	36.407085	100.960327
104.219994	129.000000	104.230484	31.723312	100.937500
104.239998	135.167007	104.250488	27.523020	100.914429
104.259995	141.333008	104.270485	23.761200	100.891235
104.279999	137.667007	104.290489	20.409866	100.868042
104.300003	134.667007	104.310493	17.454144	100.844727
104.320000	122.500000	104.330490	14.878808	100.821228
104.340004	120.000000	104.350494	12.662024	100.797729
104.360001	114.833000	104.370491	10.781065	100.774048
104.380005	113.667000	104.390495	9.206484	100.750244
104.400002	115.500000	104.410492	7.909727	100.726440
104.419998	115.000000	104.430489	6.860838	100.702576
104.440002	114.500000	104.450493	6.033064	100.678528
104.459999	106.333000	104.470490	5.405745	100.654419
104.480003	113.500000	104.490494	4.964944	100.630188
104.500000	104.667000	104.510490	4.708219	100.605957
104.519997	109.167000	104.530487	4.646607	100.581543
104.540001	105.000000	104.550491	4.809331	100.557129
104.559998	102.167000	104.570488	5.247772	100.532593
104.580002	112.000000	104.590492	6.040966	100.508057
104.599998	109.333000	104.610489	7.298698	100.483337
104.619995	118.333000	104.630486	9.166430	100.458679
104.639999	111.333000	104.650490	11.825215	100.433838
104.659996	116.833000	104.670486	15.480914	100.408997

104.680000	128.500000	104.690491	20.354959	100.384094
104.699997	128.500000	104.710487	26.639898	100.359131
104.719994	134.667007	104.730484	34.458488	100.334106
104.739998	143.333008	104.750488	43.787727	100.309021
104.759995	163.333008	104.770485	54.360096	100.283936
104.779999	167.833008	104.790489	65.614616	100.258728
104.800003	188.833008	104.810493	76.644402	100.233459
104.820000	201.667007	104.830490	86.281471	100.208252
104.840004	209.500000	104.850494	93.306915	100.182922
104.860001	215.000000	104.870491	96.731125	100.157532
104.880005	215.167007	104.890495	96.120163	100.132202
104.900002	205.833008	104.910492	91.751465	100.106812
104.919998	196.500000	104.930489	84.538010	100.081360
104.940002	179.167007	104.950493	75.726006	100.055908
104.959999	178.000000	104.970490	66.535591	100.030396
104.980003	157.167007	104.990494	57.855988	100.004944
105.000000	150.333008	105.010490	50.172241	99.979431
105.019997	136.667007	105.030487	43.600899	99.953857
105.040001	135.667007	105.050491	38.036789	99.928345
105.059998	130.667007	105.070488	33.290512	99.902832
105.080002	121.833000	105.090492	29.164804	99.877258
105.099998	121.500000	105.110489	25.512321	99.851685
105.119995	121.667000	105.130486	22.232853	99.826111
105.139999	123.000000	105.150490	19.269485	99.800598
105.159996	128.500000	105.170486	16.596813	99.775024
105.180000	119.167000	105.190491	14.199821	99.749512
105.199997	117.333000	105.210487	12.072362	99.723938
105.219994	115.000000	105.230484	10.204719	99.698486
105.239998	104.833000	105.250488	8.584234	99.672974
105.259995	111.667000	105.270485	7.196141	99.647522
105.279999	102.667000	105.290489	6.020091	99.622070
105.300003	107.833000	105.310493	5.035281	99.596619
105.320000	99.833298	105.330490	4.219731	99.571289
105.340004	104.167000	105.350494	3.550822	99.545898
105.360001	103.333000	105.370491	3.008154	99.520508
105.380005	99.166695	105.390495	2.572066	99.495239
105.400002	93.166695	105.410492	2.225665	99.470032
105.419998	92.833298	105.430489	1.953677	99.444824

105.440002	101.500000	105.450493	1.743102	99.419678
105.459999	94.833298	105.470490	1.583303	99.394531
105.480003	95.500000	105.490494	1.465486	99.369507
105.500000	99.833298	105.510490	1.383326	99.344482
105.519997	95.166695	105.530487	1.332882	99.319580
105.540001	96.500000	105.550491	1.313237	99.294678
105.559998	102.000000	105.570488	1.327184	99.269897
105.580002	90.333298	105.590492	1.381868	99.245117
105.599998	95.333298	105.610489	1.489752	99.220459
105.619995	103.333000	105.630486	1.669801	99.195984
105.639999	95.666695	105.650490	1.948833	99.171448
105.659996	99.500000	105.670486	2.362419	99.147034
105.680000	99.333298	105.690491	2.956870	99.122681
105.699997	100.500000	105.710487	3.788183	99.098389
105.719994	103.667000	105.730484	4.922052	99.074219
105.739998	98.666695	105.750488	6.429481	99.050171
105.759995	105.167000	105.770485	8.376137	99.026245
105.779999	104.500000	105.790489	10.813082	99.002319
105.800003	98.166695	105.810493	13.752646	98.978577
105.820000	114.333000	105.830490	17.146767	98.954895
105.840004	118.167000	105.850494	20.870165	98.931396
105.860001	119.167000	105.870491	24.700094	98.907898
105.880005	125.667000	105.890495	28.338253	98.884583
105.900002	124.000000	105.910492	31.439833	98.861450
105.919998	134.833008	105.930489	33.689362	98.838379
105.940002	130.000000	105.950493	34.867626	98.815430
105.959999	137.333008	105.970490	34.902115	98.792603
105.980003	133.500000	105.990494	33.876465	98.769897
106.000000	132.667007	106.010490	31.994976	98.747314
106.019997	128.833008	106.030487	29.523977	98.724915
106.040001	130.167007	106.050491	26.737093	98.702637
106.059998	130.667007	106.070488	23.878960	98.680542
106.080002	119.167000	106.090492	21.132992	98.658447
106.099998	124.000000	106.110489	18.619371	98.636719
106.119995	107.833000	106.130486	16.390635	98.614990
106.139999	109.833000	106.150490	14.449545	98.593506
106.159996	116.167000	106.170486	12.771000	98.572144
106.180000	111.667000	106.190491	11.316444	98.550903

106.199997	109.833000	106.210487	10.053108	98.529968
106.219994	111.833000	106.230484	8.957496	98.509094
106.239998	110.833000	106.250488	8.019931	98.488403
106.259995	100.333000	106.270485	7.245109	98.467896
106.279999	105.333000	106.290489	6.648771	98.447632
106.300003	104.167000	106.310493	6.259283	98.427429
106.320000	95.000000	106.330490	6.116361	98.407471
106.340004	102.333000	106.350494	6.271668	98.387756
106.360001	98.833298	106.370491	6.788859	98.368225
106.380005	102.000000	106.390495	7.742596	98.348755
106.400002	103.500000	106.410492	9.212100	98.329712
106.419998	111.167000	106.430489	11.273663	98.310669
106.440002	117.833000	106.450493	13.984197	98.291870
106.459999	111.667000	106.470490	17.353188	98.273376
106.480003	106.000000	106.490494	21.320415	98.255066
106.500000	129.167007	106.510490	25.715137	98.236938
106.519997	128.167007	106.530487	30.246161	98.218994
106.540001	119.667000	106.550491	34.504066	98.201294
106.559998	135.833008	106.570488	38.004761	98.183960
106.580002	135.333008	106.590492	40.293545	98.166626
106.599998	128.167007	106.610489	41.054008	98.149658
106.619995	137.833008	106.630486	40.214458	98.132935
106.639999	135.000000	106.650490	37.973804	98.116333
106.659996	136.167007	106.670486	34.746666	98.100098
106.680000	124.667000	106.690491	31.029829	98.083984
106.699997	129.333008	106.710487	27.276068	98.068298
106.719994	119.333000	106.730484	23.795790	98.052734
106.739998	120.000000	106.750488	20.740229	98.037476
106.759995	109.167000	106.770485	18.135420	98.022461
106.779999	111.500000	106.790489	15.927019	98.007690
106.800003	107.333000	106.810493	14.036096	97.993164
106.820000	107.167000	106.830490	12.387392	97.978882
106.840004	107.667000	106.850494	10.922685	97.964966
106.860001	110.167000	106.870491	9.607451	97.951294
106.880005	109.000000	106.890495	8.422623	97.937866
106.900002	105.167000	106.910492	7.362912	97.924744
106.919998	106.833000	106.930489	6.428301	97.911865
106.940002	103.333000	106.950485	5.621899	97.899231

106.959999	100.167000	106.970482	4.946832	97.886963
106.980003	94.333298	106.990486	4.404333	97.875000
107.000000	102.667000	107.010483	3.993175	97.863281
107.019997	91.500000	107.030479	3.707171	97.851868
107.040001	98.000000	107.050484	3.534593	97.840759
107.059998	100.833000	107.070480	3.456839	97.829956
107.080002	93.000000	107.090485	3.447387	97.819458
107.099998	101.500000	107.110481	3.472992	97.809265
107.119995	91.000000	107.130478	3.496936	97.799438
107.139999	100.667000	107.150482	3.484953	97.789856
107.159996	92.833298	107.170479	3.412475	97.780640
107.180000	98.333298	107.190483	3.270442	97.771729
107.199997	95.333298	107.210480	3.067213	97.763062
107.219994	97.833298	107.230476	2.824393	97.754883
107.239998	95.000000	107.250481	2.569086	97.746826
107.259995	96.833298	107.270477	2.325979	97.739258
107.279999	92.500000	107.290482	2.111275	97.732056
107.300003	99.333298	107.310486	1.932331	97.725098
107.320000	88.333298	107.330482	1.789675	97.718506
107.340004	89.500000	107.350487	1.680667	97.712280
107.360001	90.666695	107.370483	1.603122	97.706299
107.380005	92.666695	107.390488	1.556639	97.700806
107.400002	87.166695	107.410484	1.542742	97.695557
107.419998	88.166695	107.430481	1.563283	97.690674
107.440002	92.000000	107.450485	1.618510	97.686218
107.459999	95.833298	107.470482	1.704938	97.682129
107.480003	91.833298	107.490486	1.814077	97.678345
107.500000	90.500000	107.510483	1.931777	97.674927
107.519997	92.666695	107.530479	2.039580	97.671875
107.540001	96.166695	107.550484	2.117454	97.669189
107.559998	90.666695	107.570480	2.148066	97.666931
107.580002	89.333298	107.590485	2.121598	97.665039
107.599998	100.667000	107.610481	2.038745	97.663452
107.619995	102.667000	107.630478	1.910582	97.662231
107.639999	99.000000	107.650482	1.755019	97.661499
107.659996	89.333298	107.670479	1.591696	97.661133
107.680000	85.833298	107.690483	1.436661	97.661133
107.699997	95.166695	107.710480	1.300183	97.661499

107.719994	97.166695	107.730476	1.186240	97.662292
107.739998	99.500000	107.750488	1.094339	97.663452
107.759995	101.667000	107.770485	1.021890	97.665039
107.779999	92.333298	107.790489	0.965670	97.666992
107.800003	93.000000	107.810493	0.923419	97.669434
107.820000	96.166695	107.830490	0.894388	97.672241
107.840004	95.166695	107.850494	0.879621	97.675354
107.860001	99.166695	107.870491	0.882360	97.678955
107.880005	92.000000	107.890495	0.908272	97.682983
107.900002	89.666695	107.910492	0.965938	97.687439
107.919998	97.333298	107.930489	1.067587	97.692261
107.940002	100.000000	107.950493	1.229973	97.697510
107.959999	98.500000	107.970490	1.475016	97.703186
107.980003	94.833298	107.990494	1.831084	97.709351
108.000000	99.333298	108.010490	2.332564	97.715820
108.019997	106.833000	108.030487	3.020210	97.722778
108.040001	97.000000	108.050491	3.939143	97.730225
108.059998	92.833298	108.070488	5.133284	97.738037
108.080002	96.666695	108.090492	6.640899	97.746338
108.099998	104.833000	108.110489	8.480121	97.754883
108.119995	106.000000	108.130486	10.638574	97.764038
108.139999	105.333000	108.150490	13.056597	97.773621
108.159996	111.500000	108.170486	15.611088	97.783691
108.180000	114.500000	108.190491	18.118689	97.794067
108.199997	115.000000	108.210487	20.342251	97.804993
108.219994	117.000000	108.230484	22.033649	97.816284
108.239998	121.333000	108.250488	22.984507	97.828125
108.259995	125.833000	108.270485	23.083586	97.840332
108.279999	121.000000	108.290489	22.355574	97.853027
108.300003	115.333000	108.310493	20.957150	97.866211
108.320000	126.333000	108.330490	19.133085	97.879761
108.340004	111.667000	108.350494	17.145342	97.893799
108.360001	107.500000	108.370491	15.216712	97.908325
108.380005	112.333000	108.390495	13.491932	97.923218
108.400002	111.500000	108.410492	12.042291	97.938721
108.419998	98.500000	108.430489	10.881860	97.954590
108.440002	111.333000	108.450493	9.997957	97.970886
108.459999	103.333000	108.470490	9.375386	97.987732

108.480003	101.000000	108.490494	9.009344	98.005066
108.500000	102.000000	108.510490	8.913390	98.022766
108.519997	106.167000	108.530487	9.117547	98.041016
108.540001	110.667000	108.550491	9.663824	98.059692
108.559998	109.500000	108.570488	10.596062	98.078857
108.580002	115.833000	108.590492	11.949386	98.098511
108.599998	117.000000	108.610489	13.731750	98.118591
108.619995	115.667000	108.630486	15.909289	98.139160
108.639999	113.333000	108.650490	18.388094	98.160278
108.659996	127.167000	108.670486	20.999657	98.181763
108.680000	131.500000	108.690491	23.510639	98.203735
108.699997	133.000000	108.710487	25.640409	98.226196
108.719994	135.333008	108.730484	27.117365	98.249207
108.739998	125.667000	108.750488	27.740244	98.272644
108.759995	122.500000	108.770485	27.438187	98.296631
108.779999	121.833000	108.790489	26.299892	98.321045
108.800003	123.833000	108.810493	24.554176	98.345947
108.820000	125.333000	108.830490	22.509365	98.371338
108.840004	124.500000	108.850494	20.473740	98.397217
108.860001	117.333000	108.870491	18.700541	98.423523
108.880005	118.167000	108.890495	17.355736	98.450317
108.900002	113.833000	108.910492	16.528580	98.477661
108.919998	115.500000	108.930489	16.250570	98.505493
108.940002	115.167000	108.950493	16.521620	98.533813
108.959999	122.000000	108.970490	17.322765	98.562561
108.980003	121.167000	108.990494	18.618395	98.591797
109.000000	128.667007	109.010490	20.344318	98.621582
109.019997	128.667007	109.030487	22.396605	98.651794
109.040001	134.333008	109.050491	24.619017	98.682556
109.059998	133.833008	109.070488	26.798687	98.713745
109.080002	138.500000	109.090492	28.686991	98.745483
109.099998	146.500000	109.110489	30.027237	98.777649
109.119995	140.667007	109.130486	30.608101	98.810364
109.139999	141.833008	109.150490	30.310734	98.843506
109.159996	144.833008	109.170486	29.145052	98.877136
109.180000	134.167007	109.190491	27.251001	98.911316
109.199997	132.167007	109.210487	24.867008	98.945923
109.219994	131.500000	109.230484	22.264585	98.981079

109.239998	126.167000	109.250488	19.686584	99.016724
109.259995	118.333000	109.270485	17.307171	99.052856
109.279999	126.333000	109.290489	15.212457	99.089355
109.300003	121.667000	109.310493	13.420282	99.126465
109.320000	118.333000	109.330490	11.903316	99.164062
109.340004	114.833000	109.350494	10.614237	99.202087
109.360001	111.500000	109.370491	9.508963	99.240601
109.380005	115.500000	109.390495	8.553116	99.279663
109.400002	108.500000	109.410492	7.728967	99.319092
109.419998	116.500000	109.430489	7.031957	99.359131
109.440002	124.833000	109.450493	6.469548	99.399597
109.459999	111.333000	109.470490	6.058783	99.440552
109.480003	108.833000	109.490494	5.822351	99.481934
109.500000	114.833000	109.510490	5.787371	99.523926
109.519997	121.167000	109.530487	5.981857	99.566223
109.540001	115.833000	109.550491	6.431680	99.609131
109.559998	112.167000	109.570488	7.154327	99.652466
109.580002	106.667000	109.590492	8.153055	99.696289
109.599998	114.167000	109.610489	9.405713	99.740601
109.619995	115.333000	109.630486	10.857927	99.785278
109.639999	116.833000	109.650490	12.415740	99.830566
109.659996	119.500000	109.670486	13.943926	99.876282
109.680000	120.000000	109.690491	15.281898	99.922485
109.699997	120.667000	109.710487	16.264572	99.969116
109.719994	119.500000	109.730484	16.761147	100.016113
109.739998	128.167007	109.750488	16.707525	100.063843
109.759995	113.667000	109.770485	16.127193	100.111816
109.779999	116.333000	109.790489	15.125605	100.160278
109.800003	118.000000	109.810493	13.861262	100.209229
109.820000	111.333000	109.830490	12.502617	100.258667
109.840004	118.333000	109.850494	11.188155	100.308594
109.860001	112.667000	109.870491	10.009354	100.358887
109.880005	114.333000	109.890495	9.005967	100.409668
109.900002	108.000000	109.910492	8.182301	100.460876
109.919998	116.833000	109.930489	7.522008	100.512573
109.940002	114.333000	109.950493	7.005063	100.564697
109.959999	112.000000	109.970490	6.618646	100.617310
109.980003	119.000000	109.990494	6.361415	100.670288

110.000000	110.167000	110.010490	6.246682	100.723755
110.019997	108.833000	110.030487	6.301606	100.777527
110.040001	113.000000	110.050491	6.566926	100.831848
110.059998	118.167000	110.070488	7.095008	100.886597
110.080002	116.500000	110.090492	7.948769	100.941833
110.099998	117.833000	110.110489	9.196531	100.997375
110.119995	120.000000	110.130486	10.908308	101.053467
110.139999	130.833008	110.150490	13.146796	101.109802
110.159996	127.333000	110.170486	15.951574	101.166687
110.180000	131.167007	110.190491	19.330448	101.223999
110.199997	136.333008	110.210487	23.235479	101.281677
110.219994	137.167007	110.230484	27.560846	101.339722
110.239998	143.667007	110.250488	32.140865	101.398315
110.259995	152.833008	110.270485	36.763035	101.457153
110.279999	159.500000	110.290489	41.218906	101.516479
110.300003	165.333008	110.310493	45.342491	101.576172
110.320000	174.000000	110.330490	49.061840	101.636230
110.340004	172.667007	110.350494	52.423664	101.696777
110.360001	184.000000	110.370491	55.568829	101.757568
110.380005	182.833008	110.390495	58.698662	101.818909
110.400002	188.500000	110.410492	62.011066	101.880493
110.419998	193.833008	110.430489	65.663353	101.942505
110.440002	204.333008	110.450493	69.735153	102.004883
110.459999	197.000000	110.470490	74.191597	102.067627
110.480003	203.500000	110.490494	78.859161	102.130676
110.500000	201.333008	110.510490	83.385544	102.194153
110.519997	208.167007	110.530487	87.261971	102.257996
110.540001	204.167007	110.550491	89.889946	102.322144
110.559998	203.833008	110.570488	90.716682	102.386658
110.580002	204.333008	110.590492	89.403214	102.451538
110.599998	204.333008	110.610489	85.946693	102.516724
110.619995	193.333008	110.630486	80.703896	102.582275
110.639999	191.167007	110.650490	74.298103	102.648071
110.659996	183.167007	110.670486	67.457886	102.714294
110.680000	169.500000	110.690491	60.820053	102.780823
110.699997	176.167007	110.710487	54.826561	102.847656
110.719994	165.167007	110.730484	49.668568	102.914734
110.739998	166.667007	110.750488	45.328224	102.982239

110.759995	158.167007	110.770485	41.654015	103.049988
110.779999	154.500000	110.790489	38.427948	103.118042
110.800003	155.667007	110.810493	35.442020	103.186401
110.820000	146.333008	110.830490	32.538967	103.255005
110.840004	135.167007	110.850494	29.632311	103.323914
110.860001	138.500000	110.870491	26.715191	103.393066
110.880005	136.333008	110.890495	23.834570	103.462585
110.900002	132.167007	110.910492	21.072561	103.532288
110.919998	126.167000	110.930489	18.510719	103.602295
110.940002	124.167000	110.950493	16.213564	103.672485
110.959999	124.333000	110.970490	14.222597	103.742981
110.980003	126.167000	110.990494	12.552378	103.813721
111.000000	118.833000	111.010490	11.204114	103.884644
111.019997	122.500000	111.030487	10.169497	103.955872
111.040001	123.333000	111.050491	9.439320	104.027344
111.059998	116.333000	111.070488	9.006455	104.098999
111.080002	108.833000	111.090492	8.863147	104.170837
111.099998	110.000000	111.110489	8.998280	104.242920
111.119995	110.167000	111.130486	9.390446	104.315186
111.139999	122.167000	111.150490	10.001419	104.387695
111.159996	117.333000	111.170486	10.768829	104.460327
111.180000	115.833000	111.190491	11.605346	104.533203
111.199997	110.167000	111.210487	12.400405	104.606201
111.219994	114.667000	111.230484	13.034945	104.679443
111.239998	123.500000	111.250488	13.400899	104.752808
111.259995	119.500000	111.270485	13.425232	104.826294
111.279999	114.333000	111.290489	13.090099	104.900024
111.300003	126.167000	111.310493	12.438528	104.973877
111.320000	116.167000	111.330490	11.563431	105.047852
111.340004	119.000000	111.350494	10.580589	105.121948
111.360001	113.333000	111.370491	9.601473	105.196167
111.380005	106.000000	111.390495	8.707530	105.270508
111.400002	110.667000	111.410492	7.942649	105.344971
111.419998	106.333000	111.430489	7.312786	105.419434
111.440002	109.167000	111.450493	6.797058	105.494141
111.459999	112.833000	111.470490	6.360639	105.568848
111.480003	106.333000	111.490494	5.964952	105.643738
111.500000	109.833000	111.510490	5.577904	105.718628

111.519997	109.667000	111.530487	5.178290	105.793579
111.540001	99.333298	111.550491	4.758342	105.868591
111.559998	101.833000	111.570488	4.322967	105.943726
111.580002	106.333000	111.590492	3.884732	106.018799
111.599998	99.833298	111.610489	3.460048	106.093933
111.619995	103.667000	111.630486	3.063328	106.169067
111.639999	107.333000	111.650490	2.704554	106.244324
111.659996	108.667000	111.670486	2.388762	106.319519
111.680000	102.000000	111.690491	2.115870	106.394775
111.699997	101.500000	111.710487	1.883161	106.469910
111.719994	99.500000	111.730484	1.686090	106.545166
111.739998	102.000000	111.750488	1.519854	106.620361
111.759995	103.667000	111.770485	1.380220	106.695496
111.779999	102.833000	111.790489	1.263404	106.770630
111.800003	100.833000	111.810493	1.166514	106.845703
111.820000	96.333298	111.830490	1.087282	106.920715
111.840004	100.167000	111.850494	1.023907	106.995605
111.860001	107.500000	111.870491	0.975220	107.070557
111.880005	102.000000	111.890495	0.940498	107.145386
111.900002	98.833298	111.910492	0.919724	107.219971
111.919998	100.833000	111.930489	0.913687	107.294556
111.940002	93.333298	111.950493	0.924353	107.369141
111.959999	102.500000	111.970490	0.955249	107.443481
111.980003	100.333000	111.990494	1.012051	107.517700
112.000000	98.500000	112.010490	1.103135	107.591919
112.019997	100.667000	112.030487	1.240486	107.665894
112.040001	108.833000	112.050491	1.440556	107.739685
112.059998	105.000000	112.070488	1.724826	107.813354
112.080002	104.167000	112.090492	2.121174	107.886841
112.099998	111.000000	112.110489	2.663392	107.960083
112.119995	109.333000	112.130486	3.391965	108.033203
112.139999	109.167000	112.150490	4.352735	108.106201
112.159996	107.167000	112.170486	5.592782	108.178833
112.180000	107.333000	112.190491	7.158545	108.251343
112.199997	117.667000	112.210487	9.084924	108.323608
112.219994	115.833000	112.230484	11.389927	108.395569
112.239998	120.500000	112.250488	14.063090	108.467407
112.259995	122.167000	112.270485	17.052416	108.538818

112.279999	123.333000	112.290489	20.266869	108.610107
112.300003	121.333000	112.310493	23.572269	108.681030
112.320000	133.000000	112.330490	26.809235	108.751709
112.340004	135.333008	112.350494	29.821640	108.822021
112.360001	135.667007	112.370491	32.475620	108.892090
112.380005	138.833008	112.390495	34.687817	108.961792
112.400002	152.667007	112.410492	36.417931	109.031250
112.419998	143.833008	112.430489	37.655476	109.100220
112.440002	147.667007	112.450493	38.386982	109.168945
112.459999	146.833008	112.470490	38.575703	109.237183
112.480003	148.667007	112.490494	38.167240	109.305176
112.500000	149.167007	112.510490	37.118816	109.372681
112.519997	149.000000	112.530487	35.438599	109.439819
112.540001	152.167007	112.550491	33.212070	109.506592
112.559998	139.000000	112.570488	30.602905	109.572998
112.580002	131.833008	112.590492	27.814386	109.638855
112.599998	136.500000	112.610489	25.050465	109.704285
112.619995	127.833000	112.630486	22.467924	109.769287
112.639999	131.333008	112.650490	20.159126	109.833862
112.659996	121.833000	112.670486	18.155041	109.897949
112.680000	119.500000	112.690491	16.435019	109.961548
112.699997	125.333000	112.710487	14.953616	110.024536
112.719994	116.167000	112.730484	13.654587	110.087158
112.739998	118.167000	112.750488	12.486711	110.149292
112.759995	114.167000	112.770485	11.413048	110.210693
112.779999	115.167000	112.790489	10.411703	110.271729
112.800003	107.000000	112.810493	9.479219	110.332153
112.820000	114.167000	112.830490	8.626901	110.391968
112.840004	110.667000	112.850494	7.875685	110.451172
112.860001	116.000000	112.870491	7.253099	110.509888
112.880005	115.833000	112.890495	6.785951	110.567932
112.900002	109.167000	112.910492	6.498260	110.625366
112.919998	111.167000	112.930489	6.407125	110.682190
112.940002	111.167000	112.950493	6.521564	110.738403
112.959999	109.333000	112.970490	6.839844	110.793945
112.980003	114.000000	112.990494	7.347191	110.848755
113.000000	114.167000	113.010490	8.011465	110.902954
113.019997	106.333000	113.030487	8.781540	110.956421

113.040001	110.833000	113.050491	9.586412	111.009277
113.059998	113.333000	113.070488	10.338061	111.061340
113.080002	113.000000	113.090492	10.943424	111.112671
113.099998	108.667000	113.110489	11.317697	111.163330
113.119995	111.833000	113.130486	11.403822	111.213196
113.139999	110.500000	113.150490	11.185784	111.262268
113.159996	109.333000	113.170486	10.694515	111.310547
113.180000	107.667000	113.190491	10.000023	111.358154
113.199997	108.500000	113.210487	9.195548	111.404846
113.219994	110.000000	113.230484	8.375014	111.450745
113.239998	110.000000	113.250488	7.616864	111.495850
113.259995	110.833000	113.270485	6.976836	111.540161
113.279999	109.167000	113.290489	6.487105	111.583496
113.300003	113.167000	113.310493	6.165482	111.626099
113.320000	106.667000	113.330490	6.023959	111.667725
113.340004	104.667000	113.350494	6.077374	111.708496
113.360001	111.333000	113.370491	6.349387	111.748352
113.380005	109.833000	113.390495	6.875261	111.787292
113.400002	114.333000	113.410492	7.700586	111.825256
113.419998	112.167000	113.430489	8.879388	111.862305
113.440002	116.333000	113.450493	10.468564	111.898438
113.459999	107.333000	113.470490	12.517474	111.933594
113.480003	118.167000	113.490494	15.062305	111.967773
113.500000	122.667000	113.510490	18.107824	112.000916
113.519997	119.167000	113.530487	21.621656	112.033081
113.540001	128.500000	113.550491	25.522085	112.064209
113.559998	133.167007	113.570488	29.667704	112.094238
113.580002	135.000000	113.590492	33.869995	112.123352
113.599998	144.500000	113.610489	37.891529	112.151245
113.619995	145.667007	113.630486	41.475166	112.178223
113.639999	155.000000	113.650490	44.362617	112.203979
113.659996	158.167007	113.670486	46.320896	112.228699
113.680000	155.333008	113.690491	47.181473	112.252319
113.699997	154.500000	113.710487	46.867428	112.274780
113.719994	149.667007	113.730484	45.422070	112.296021
113.739998	151.000000	113.750488	43.012241	112.316162
113.759995	148.000000	113.770485	39.910908	112.335144
113.779999	144.333008	113.790489	36.441467	112.353027

113.800003	146.833008	113.810493	32.924454	112.369568
113.820000	140.667007	113.830490	29.621529	112.385010
113.840004	133.500000	113.850494	26.703199	112.399109
113.860001	125.833000	113.870491	24.256662	112.412048
113.880005	122.833000	113.890495	22.296862	112.423767
113.900002	131.667007	113.910492	20.801489	112.434204
113.919998	121.500000	113.930489	19.730015	112.443237
113.940002	123.167000	113.950493	19.042282	112.451172
113.959999	123.333000	113.970490	18.703939	112.457581
113.980003	134.833008	113.990494	18.681713	112.462769
114.000000	129.000000	114.010498	18.936239	112.466675
114.019997	135.333008	114.030495	19.410990	112.469238
114.040001	137.667007	114.050499	20.025261	112.470459
114.059998	132.167007	114.070496	20.670465	112.470093
114.080002	135.833008	114.090500	21.218269	112.468628
114.099998	132.333008	114.110497	21.535307	112.465576
114.119995	130.167007	114.130493	21.508900	112.461121
114.139999	136.167007	114.150497	21.071922	112.455261
114.159996	134.667007	114.170494	20.222631	112.447998
114.180000	133.500000	114.190498	19.026958	112.439209
114.199997	128.333008	114.210495	17.606998	112.429016
114.219994	125.500000	114.230492	16.111296	112.417358
114.239998	127.833000	114.250496	14.686649	112.404175
114.259995	134.667007	114.270493	13.458057	112.389404
114.279999	124.667000	114.290497	12.516862	112.373169
114.300003	124.500000	114.310501	11.928889	112.355347
114.320000	136.500000	114.330498	11.745228	112.336060
114.340004	125.833000	114.350502	12.017521	112.315125
114.360001	124.833000	114.370499	12.810243	112.292603
114.380005	129.667007	114.390503	14.208595	112.268555
114.400002	123.333000	114.410500	16.316505	112.242920
114.419998	130.000000	114.430496	19.253586	112.215576
114.440002	131.667007	114.450500	23.141632	112.186646
114.459999	137.333008	114.470497	28.077864	112.156128
114.480003	158.167007	114.490501	34.117805	112.123779
114.500000	164.333008	114.510498	41.225220	112.089966
114.519997	169.000000	114.530495	49.250042	112.054321
114.540001	173.500000	114.550499	57.891293	112.017029

114.559998	185.333008	114.570496	66.671867	111.978027
114.580002	201.833008	114.590500	74.980026	111.937256
114.599998	206.667007	114.610497	82.106392	111.894836
114.619995	216.333008	114.630493	87.374352	111.850647
114.639999	227.000000	114.650497	90.262520	111.804626
114.659996	217.333008	114.670494	90.529984	111.756836
114.680000	226.667007	114.690498	88.291542	111.707336
114.699997	211.000000	114.710495	83.997147	111.655945
114.719994	203.333008	114.730492	78.318527	111.602783
114.739998	198.000000	114.750496	71.986496	111.547729
114.759995	189.000000	114.770493	65.641891	111.490967
114.779999	182.333008	114.790497	59.713970	111.432251
114.800003	178.000000	114.810501	54.408627	111.371582
114.820000	171.667007	114.830498	49.737453	111.309204
114.840004	170.667007	114.850494	45.588764	111.244873
114.860001	153.167007	114.870491	41.811653	111.178589
114.880005	159.333008	114.890495	38.271957	111.110474
114.900002	148.000000	114.910492	34.888340	111.040405
114.919998	147.500000	114.930489	31.628311	110.968384
114.940002	138.333008	114.950493	28.499075	110.894348
114.959999	141.500000	114.970490	25.531034	110.818359
114.980003	152.833008	114.990494	22.752266	110.740417
115.000000	139.833008	115.010490	20.186928	110.660522
115.019997	135.333008	115.030487	17.844053	110.578613
115.040001	143.000000	115.050491	15.722221	110.494629
115.059998	132.667007	115.070488	13.814867	110.408691
115.080002	122.333000	115.090492	12.108108	110.320740
115.099998	126.167000	115.110489	10.589100	110.230652
115.119995	120.500000	115.130486	9.242834	110.138550
115.139999	123.833000	115.150490	8.054989	110.044312
115.159996	122.000000	115.170486	7.013284	109.948120
115.180000	126.000000	115.190491	6.104450	109.849731
115.199997	116.833000	115.210487	5.317487	109.749268
115.219994	117.667000	115.230484	4.641192	109.646729
115.239998	109.333000	115.250488	4.065598	109.541931
115.259995	108.000000	115.270485	3.582750	109.435059
115.279999	110.167000	115.290489	3.185652	109.326050
115.300003	108.167000	115.310493	2.870111	109.214844

115.320000	111.167000	115.330490	2.634580	109.101562
115.340004	106.500000	115.350494	2.480615	108.986084
115.360001	107.500000	115.370491	2.414035	108.868408
115.380005	102.167000	115.390495	2.445066	108.748535
115.400002	107.167000	115.410492	2.589048	108.626404
115.419998	105.000000	115.430489	2.866752	108.502197
115.440002	123.000000	115.450493	3.304542	108.375549
115.459999	107.833000	115.470490	3.932982	108.246704
115.480003	109.167000	115.490494	4.786616	108.115723
115.500000	119.333000	115.510490	5.899109	107.982422
115.519997	117.000000	115.530487	7.300785	107.846863
115.540001	113.500000	115.550491	9.011744	107.708984
115.559998	126.500000	115.570488	11.031294	107.568787
115.580002	119.667000	115.590492	13.333755	107.426392
115.599998	120.833000	115.610489	15.854089	107.281738
115.619995	134.000000	115.630486	18.489162	107.134644
115.639999	128.500000	115.650490	21.097786	106.985229
115.659996	134.833008	115.670486	23.510479	106.833618
115.680000	134.500000	115.690491	25.560724	106.679565
115.699997	142.500000	115.710487	27.111073	106.523254
115.719994	129.500000	115.730484	28.092737	106.364624
115.739998	132.667007	115.750488	28.525320	106.203369
115.759995	139.667007	115.770485	28.518930	106.039978
115.779999	131.333008	115.790489	28.251169	105.874146
115.800003	137.333008	115.810493	27.922977	105.705872
115.820000	129.833008	115.830490	27.708406	105.535278
115.840004	140.833008	115.850494	27.712524	105.362305
115.860001	126.000000	115.870491	27.950064	105.186890
115.880005	131.167007	115.890495	28.347031	105.009155
115.900002	140.667007	115.910492	28.762640	104.828918
115.919998	145.167007	115.930489	29.026661	104.646240
115.940002	128.833008	115.950493	28.980749	104.461182
115.959999	132.167007	115.970490	28.516830	104.273804
115.980003	133.000000	115.990494	27.601440	104.083679
116.000000	132.333008	116.010490	26.282213	103.891357
116.019997	131.000000	116.030487	24.669836	103.696655
116.040001	137.167007	116.050491	22.907707	103.499268
116.059998	123.500000	116.070488	21.139511	103.299561

116.080002	121.500000	116.090492	19.477129	103.097290
116.099998	123.333000	116.110489	17.992962	102.892700
116.119995	117.833000	116.130486	16.717463	102.685547
116.139999	122.167000	116.150490	15.653589	102.475830
116.159996	121.333000	116.170486	14.792912	102.263855
116.180000	114.667000	116.190491	14.125124	102.049194
116.199997	122.000000	116.210487	13.646379	101.832153
116.219994	114.000000	116.230484	13.356112	101.612671
116.239998	115.000000	116.250488	13.251994	101.390625
116.259995	119.000000	116.270485	13.321680	101.166077
116.279999	113.000000	116.290489	13.535570	100.938965
116.300003	116.667000	116.310493	13.843042	100.709351
116.320000	116.333000	116.330490	14.173381	100.477295
116.340004	106.167000	116.350494	14.442880	100.242798
116.360001	114.500000	116.370491	14.565853	100.005859
116.380005	114.500000	116.390495	14.470490	99.766357
116.400002	122.833000	116.410492	14.113577	99.524292
116.419998	114.000000	116.430489	13.490590	99.279785
116.440002	105.000000	116.450493	12.636857	99.032715
116.459999	112.667000	116.470490	11.620441	98.783142
116.480003	106.167000	116.490494	10.523908	98.531006
116.500000	108.833000	116.510490	9.429317	98.276489
116.519997	104.667000	116.530487	8.401015	98.019409
116.540001	116.500000	116.550491	7.479645	97.759827
116.559998	110.000000	116.570488	6.683184	97.497742
116.580002	109.333000	116.590492	6.009907	97.233154
116.599998	106.667000	116.610489	5.448349	96.966064
116.619995	102.167000	116.630486	4.982519	96.696594
116.639999	100.833000	116.650490	4.598151	96.424500
116.659996	103.833000	116.670486	4.286105	96.150024
116.680000	98.833298	116.690491	4.042679	95.872925
116.699997	102.333000	116.710487	3.870531	95.593384
116.719994	99.333298	116.730484	3.777182	95.311401
116.739998	102.667000	116.750488	3.774241	95.026855
116.759995	100.333000	116.770485	3.875774	94.739990
116.779999	99.000000	116.790489	4.096676	94.450500
116.800003	109.167000	116.810493	4.450369	94.158447
116.820000	105.833000	116.830490	4.946149	93.864258

116.840004	98.166695	116.850494	5.586845	93.567383
116.860001	106.667000	116.870491	6.364069	93.268127
116.880005	103.667000	116.890495	7.257051	92.966431
116.900002	100.500000	116.910492	8.228340	92.662354
116.919998	111.000000	116.930489	9.226363	92.355835
116.940002	109.167000	116.950493	10.188161	92.046814
116.959999	107.000000	116.970490	11.045904	91.735596
116.980003	111.167000	116.990494	11.740340	91.421753
117.000000	108.667000	117.010490	12.230399	91.105652
117.019997	102.500000	117.030487	12.504930	90.787109
117.040001	115.500000	117.050491	12.585475	90.466125
117.059998	110.667000	117.070488	12.522704	90.142822
117.080002	95.666695	117.090492	12.385687	89.817139
117.099998	105.667000	117.110489	12.247304	89.489136
117.119995	104.500000	117.130486	12.169518	89.158813
117.139999	111.167000	117.150490	12.192588	88.826050
117.159996	98.000000	117.170486	12.328635	88.491211
117.180000	103.000000	117.190491	12.560261	88.153809
117.199997	103.833000	117.210487	12.842870	87.814270
117.219994	102.667000	117.230484	13.112000	87.472412
117.239998	107.833000	117.250488	13.293557	87.128235
117.259995	106.833000	117.270485	13.317008	86.781982
117.279999	103.333000	117.290489	13.129819	86.433350
117.300003	101.167000	117.310493	12.708654	86.082458
117.320000	100.833000	117.330490	12.065100	85.729431
117.340004	97.000000	117.350494	11.241642	85.374023
117.360001	91.833298	117.370491	10.303130	85.016724
117.380005	92.666695	117.390495	9.319852	84.657043
117.400002	89.333298	117.410492	8.356281	84.295410
117.419998	97.833298	117.430489	7.458939	83.931519
117.440002	100.000000	117.450493	6.654105	83.565430
117.459999	81.500000	117.470490	5.950510	83.197449
117.480003	85.166695	117.490494	5.342377	82.827148
117.500000	87.666695	117.510490	4.817567	82.454956
117.519997	91.000000	117.530487	4.361055	82.080811
117.540001	79.000000	117.550491	3.959313	81.704468
117.559998	84.833298	117.570488	3.602609	81.326233
117.580002	85.500000	117.590492	3.284653	80.945923

117.599998	91.500000	117.610489	3.003601	80.563843
117.619995	83.500000	117.630486	2.760707	80.179688
117.639999	81.500000	117.650490	2.560230	79.793579
117.659996	88.166695	117.670486	2.409156	79.405640
117.680000	81.000000	117.690491	2.316384	79.015747
117.699997	83.833298	117.710487	2.292933	78.624084
117.719994	81.500000	117.730484	2.351387	78.230591
117.739998	80.500000	117.750488	2.505733	77.835205
117.759995	77.500000	117.770485	2.770353	77.438110
117.779999	85.166695	117.790489	3.159374	77.039185
117.800003	81.833298	117.810493	3.684270	76.638550
117.820000	82.166695	117.830490	4.351434	76.236328
117.840004	82.333298	117.850494	5.159972	75.832275
117.860001	83.833298	117.870491	6.096154	75.426636
117.880005	77.500000	117.890495	7.132389	75.019348
117.900002	81.666695	117.910492	8.222001	74.610657
117.919998	80.500000	117.930489	9.301909	74.200500
117.940002	82.166695	117.950493	10.295052	73.788574
117.959999	81.500000	117.970490	11.117380	73.375122
117.980003	82.666695	117.990494	11.692506	72.960205
118.000000	78.000000	118.010490	11.963445	72.543945
118.019997	80.500000	118.030487	11.906728	72.126221
118.040001	85.166695	118.050491	11.537720	71.707153
118.059998	82.666695	118.070488	10.909324	71.286682
118.080002	82.500000	118.090492	10.099838	70.864868
118.099998	66.333298	118.110489	9.198337	70.441956
118.119995	76.666695	118.130486	8.285964	70.017700
118.139999	76.833298	118.150490	7.424575	69.592163
118.159996	74.833298	118.170486	6.652352	69.165527
118.180000	74.166695	118.190491	5.982824	68.737671
118.199997	74.333298	118.210487	5.412809	68.308960
118.219994	70.166695	118.230484	4.927742	67.879150
118.239998	66.666695	118.250488	4.509161	67.448120
118.259995	70.500000	118.270485	4.139817	67.016235
118.279999	70.000000	118.290489	3.805327	66.583252
118.300003	68.166695	118.310493	3.496248	66.149475
118.320000	70.166695	118.330490	3.207527	65.714905
118.340004	66.333298	118.350494	2.937565	65.279236

118.360001	63.833298	118.370491	2.688161	64.843140
118.380005	64.500000	118.390495	2.462976	64.406067
118.400002	63.666702	118.410492	2.267785	63.968506
118.419998	63.833298	118.430489	2.109596	63.530151
118.440002	63.166702	118.450493	1.996904	63.091187
118.459999	60.166702	118.470490	1.939843	62.651733
118.480003	60.666702	118.490494	1.950074	62.211609
118.500000	61.666702	118.510490	2.041022	61.771240
118.519997	58.333298	118.530487	2.227761	61.330444
118.540001	61.500000	118.550491	2.526761	60.889099
118.559998	65.666695	118.570488	2.954528	60.447693
118.580002	60.166702	118.590492	3.527025	60.005737
118.599998	65.500000	118.610489	4.256079	59.563660
118.619995	62.333298	118.630486	5.147670	59.121460
118.639999	65.666695	118.650490	6.197746	58.679016
118.659996	64.333298	118.670486	7.386558	58.236572
118.680000	65.666695	118.690491	8.677804	57.794067
118.699997	67.333298	118.710487	10.012863	57.351624
118.719994	61.333298	118.730484	11.314884	56.909363
118.739998	63.500000	118.750488	12.492421	56.467163
118.759995	61.333298	118.770485	13.448296	56.025146
118.779999	67.166695	118.790489	14.096969	55.583252
118.800003	62.666702	118.810493	14.377794	55.141846
118.820000	65.166695	118.830490	14.270148	54.700806
118.840004	65.500000	118.850494	13.798811	54.260010
118.860001	64.333298	118.870491	13.030807	53.820190
118.880005	60.000000	118.890495	12.060788	53.380493
118.900002	62.833298	118.910492	10.994246	52.941467
118.919998	58.000000	118.930489	9.926392	52.503174
118.940002	54.833298	118.950493	8.929717	52.065552
118.959999	52.333298	118.970490	8.049420	51.628906
118.980003	56.666702	118.990501	7.302125	51.192810
119.000000	56.833298	119.010498	6.685991	50.757812
119.019997	56.333298	119.030495	6.184895	50.323792
119.040001	52.333298	119.050499	5.777904	49.890808
119.059998	57.166702	119.070496	5.444300	49.459045
119.080002	50.166702	119.090500	5.165298	49.028320
119.099998	45.833298	119.110497	4.925813	48.599060

119.119995	53.166702	119.130493	4.713211	48.171021
119.139999	48.500000	119.150497	4.516694	47.744324
119.159996	58.666702	119.170494	4.326683	47.319153
119.180000	51.666702	119.190498	4.134003	46.895447
119.199997	50.666702	119.210495	3.930881	46.473694
119.219994	51.833298	119.230492	3.711360	46.053406
119.239998	56.500000	119.250496	3.472612	45.634766
119.259995	48.500000	119.270493	3.216027	45.218201
119.279999	49.166702	119.290497	2.946605	44.803223
119.300003	48.333298	119.310501	2.672773	44.390381
119.320000	44.333298	119.330498	2.404524	43.979675
119.340004	44.666702	119.350502	2.151311	43.571045
119.360001	46.500000	119.370499	1.921270	43.164673
119.380005	50.833298	119.390503	1.719689	42.760681
119.400002	45.666702	119.410500	1.549732	42.359009
119.419998	46.666702	119.430496	1.412543	41.959900
119.440002	47.666702	119.450500	1.308548	41.563232
119.459999	46.833298	119.470497	1.238606	41.169250
119.480003	48.333298	119.490501	1.204683	40.778015
119.500000	42.000000	119.510498	1.210777	40.389709
119.519997	40.500000	119.530495	1.263246	40.004272
119.540001	41.500000	119.550499	1.371147	39.621704
119.559998	44.166702	119.570496	1.546027	39.242249
119.580002	43.000000	119.590500	1.802030	38.865906
119.599998	42.000000	119.610497	2.154773	38.492920
119.619995	44.500000	119.630493	2.620984	38.123352
119.639999	45.500000	119.650497	3.216892	37.757019
119.659996	49.333298	119.670494	3.955198	37.394287
119.680000	44.166702	119.690498	4.843914	37.035095
119.699997	46.166702	119.710495	5.880599	36.679810
119.719994	48.666702	119.730492	7.050729	36.328308
119.739998	43.833298	119.750496	8.323320	35.980591
119.759995	45.333298	119.770493	9.646875	35.636963
119.779999	49.000000	119.790497	10.952929	35.297363
119.800003	47.000000	119.810501	12.156382	34.961975
119.820000	47.833298	119.830498	13.165507	34.631165
119.840004	45.666702	119.850502	13.895385	34.304565
119.860001	43.666702	119.870499	14.280084	33.982666

119.880005	45.166702	119.890503	14.288142	33.665161
119.900002	40.500000	119.910500	13.929409	33.352417
119.919998	42.833298	119.930496	13.254919	33.044678
119.940002	42.333298	119.950500	12.346788	32.741760
119.959999	42.833298	119.970497	11.303632	32.443848
119.980003	45.000000	119.990501	10.220097	32.151123
120.000000	44.000000	120.010498	9.175078	31.863647

```
_reflns_number_total      288
_reflns_limit_h_min       0
_reflns_limit_h_max       10
_reflns_limit_k_min       -8
_reflns_limit_k_max       0
_reflns_limit_l_min       0
_reflns_limit_l_max       7
_reflns_d_resolution_high 8.155
_reflns_d_resolution_low  .890

loop_
      _refln_index_h
      _refln_index_k
      _refln_index_l
      _refln_F_squared_meas
      _refln_d_spacing
      _refln_intensity_meas
```

1	0	0	95.145	8.15510	56938.422
1	0	1	76.146	5.26014	37321.953
2	-1	0	131.680	4.70835	25683.457
2	0	0	316.638	4.07755	45807.016
2	-1	1	180.148	3.88620	47135.250
2	0	1	66.473	3.50822	14019.099
0	0	2	5794.031	3.44173	195562.312
1	0	2	286.500	3.17091	48717.703
3	-2	0	550.609	3.08234	44052.277
3	-1	0	550.425	3.08234	44037.527
3	-2	1	2226.083	2.81317	292306.625
3	-1	1	2225.571	2.81317	292239.281
2	-1	2	2280.620	2.77854	291490.406

3	0	0	5634.548	2.71837	343262.062
2	0	2	1260.743	2.63007	142875.766
3	0	1	287.200	2.52835	29834.268
4	-2	0	53.834	2.35418	2385.179
3	-2	2	231.582	2.29613	19402.389
3	-1	2	231.554	2.29613	19400.021
4	-3	0	1838.675	2.26182	74455.102
4	-1	0	1837.652	2.26182	74413.656
4	-2	1	226.062	2.22750	17686.779
1	0	3	136.294	2.20873	10461.018
4	-3	1	336.468	2.14879	24260.930
4	-1	1	336.375	2.14879	24254.244
3	0	2	267.284	2.13323	18956.117
2	-1	3	640.172	2.06260	42038.469
4	0	0	564.112	2.03878	18034.574
2	0	3	540.663	1.99964	33062.047
4	0	1	1127.369	1.95483	65426.047
4	-2	2	3744.898	1.94310	214326.219
4	-3	2	920.421	1.89019	49409.559
4	-1	2	919.997	1.89019	49386.758
5	-3	0	652.904	1.87091	17111.568
5	-2	0	652.173	1.87091	17092.410
3	-2	3	2378.959	1.84053	120030.656
3	-1	3	2378.493	1.84053	120007.062
5	-3	1	1266.938	1.80541	61112.191
5	-2	1	1266.033	1.80541	61068.543
5	-4	0	2048.070	1.77959	47758.938
5	-1	0	2047.517	1.77959	47745.996
4	0	2	2148.154	1.75411	96858.672
3	0	3	2144.784	1.75338	96612.281
5	-4	1	1390.266	1.72294	60105.105
5	-1	1	1390.586	1.72294	60118.879
0	0	4	16465.340	1.72086	118305.211
1	0	4	228.170	1.68378	9346.197
5	-3	2	593.364	1.64375	22969.467
5	-2	2	593.205	1.64375	22963.318
4	-2	3	1188.682	1.64314	45975.047
5	0	0	202.374	1.63102	3846.174

2	-1	4	722.899	1.61629	26898.922
4	-3	3	417.548	1.61077	15412.665
4	-1	3	417.398	1.61077	15407.112
5	0	1	558.167	1.58708	19899.510
2	0	4	618.639	1.58545	22002.584
5	-4	2	200.581	1.58078	7084.693
5	-1	2	200.571	1.58078	7084.372
6	-3	0	503.473	1.56945	8743.091
6	-4	0	1329.253	1.54117	22122.814
6	-2	0	1327.911	1.54117	22100.480
6	-3	1	1053.745	1.53018	34494.625
4	0	3	399.619	1.52405	12959.937
6	-4	1	976.703	1.50394	30711.865
6	-2	1	977.301	1.50393	30730.660
3	-2	4	986.259	1.50255	30946.309
3	-1	4	986.204	1.50255	30944.559
5	0	2	2620.087	1.47389	78630.336
6	-5	0	840.964	1.46470	12438.400
6	-1	0	840.298	1.46470	12428.542
3	0	4	3759.001	1.45400	109340.523
5	-2	3	1643.339	1.44998	47498.453
5	-3	3	1643.339	1.44998	47498.473
6	-5	1	1323.886	1.43263	37227.227
6	-1	1	1321.711	1.43263	37166.055
6	-3	2	1712.104	1.42799	47789.988
6	-4	2	509.745	1.40659	13750.562
6	-2	2	509.120	1.40659	13733.692
5	-4	3	501.343	1.40621	13515.725
5	-1	3	501.158	1.40621	13510.739
4	-2	4	126.453	1.38927	3317.624
4	-3	4	59.655	1.36954	1516.152
4	-1	4	59.655	1.36954	1516.152
6	0	0	207.466	1.35918	2592.748
1	0	5	78.667	1.35748	1960.842
6	-5	2	371.213	1.34773	9108.301
6	-1	2	373.042	1.34773	9153.164
7	-4	0	117.293	1.34069	1422.714
7	-3	0	117.464	1.34069	1424.794

6	0	1	37.781	1.33344	905.874
5	0	3	20.156	1.32938	480.110
2	-1	5	637.571	1.32137	14992.236
7	-4	1	850.403	1.31596	19823.633
7	-3	1	850.199	1.31596	19818.877
4	0	4	1666.111	1.31504	38780.648
7	-5	0	1192.903	1.30586	13679.827
7	-2	0	1192.092	1.30586	13670.518
2	0	5	1076.851	1.30435	24638.262
6	-3	3	208.531	1.29540	4703.177
7	-5	1	838.977	1.28298	18550.672
7	-2	1	839.033	1.28298	18551.910
6	-4	3	885.417	1.27936	19465.162
6	-2	3	885.192	1.27936	19460.221
5	-3	4	450.092	1.26656	9696.438
5	-2	4	450.125	1.26656	9697.150
6	0	2	977.920	1.26418	20988.436
3	-1	5	1156.230	1.25701	24537.734
3	-2	5	1156.230	1.25701	24537.734
7	-4	2	664.979	1.24925	13942.452
7	-3	2	664.702	1.24925	13936.646
7	-6	0	1317.888	1.24364	13696.059
7	-1	0	1317.186	1.24364	13688.750
5	-4	4	2015.470	1.23707	41468.918
5	-1	4	2014.995	1.23707	41459.141
6	-5	3	1796.287	1.23460	36818.684
6	-1	3	1796.171	1.23459	36816.281
3	0	5	476.385	1.22817	9669.103
7	-6	1	1186.162	1.22383	23917.137
7	-1	1	1186.333	1.22383	23920.578
7	-5	2	1527.388	1.22093	30662.986
7	-2	2	1525.843	1.22093	30631.977
4	-2	5	347.973	1.18840	6659.433
5	0	4	458.960	1.18379	8726.128
8	-4	0	2815.894	1.17709	26518.436
4	-3	5	657.452	1.17598	12363.998
4	-1	5	657.452	1.17598	12363.998
7	-6	2	240.603	1.16963	4485.330

7	-1	2	240.520	1.16963	4483.775
6	0	3	499.141	1.16941	9302.256
8	-5	0	403.910	1.16501	3741.376
8	-3	0	404.347	1.16501	3745.420
7	0	0	404.347	1.16501	3745.420
8	-4	1	2139.520	1.16025	39384.027
6	-3	4	2543.022	1.15961	46772.207
7	-4	3	1234.640	1.15757	22646.674
7	-3	3	1234.486	1.15757	22643.840
8	-5	1	920.332	1.14868	16687.537
8	-3	1	920.672	1.14868	16693.703
7	0	1	920.843	1.14868	16696.783
6	-4	4	1437.290	1.14806	26040.523
6	-2	4	1437.022	1.14806	26035.666
0	0	6	16233.412	1.14724	48967.742
4	0	5	241.123	1.14093	4329.542
1	0	6	1114.375	1.13606	19889.611
7	-5	3	558.740	1.13493	9958.824
7	-2	3	558.740	1.13493	9958.820
8	-6	0	432.109	1.13091	3832.335
8	-2	0	432.059	1.13091	3831.890
8	-6	1	1771.472	1.11595	30886.197
8	-2	1	1772.088	1.11595	30896.934
6	-5	4	1907.097	1.11538	33230.156
6	-1	4	1904.391	1.11538	33182.992
2	-1	6	3776.450	1.11463	65748.266
8	-4	2	3699.572	1.11375	64347.816
5	-2	5	931.248	1.10884	16112.188
5	-3	5	931.248	1.10884	16112.189
2	0	6	3929.388	1.10436	67667.797
8	-5	2	1314.038	1.10351	22609.115
8	-3	2	1312.509	1.10351	22582.816
7	0	2	1312.000	1.10351	22574.053
7	-6	3	118.711	1.09337	2022.171
7	-1	3	118.616	1.09337	2020.557
5	-4	5	37.962	1.08889	643.976
5	-1	5	37.953	1.08889	643.819
8	-7	0	1194.543	1.08017	10054.506

8	-1	0	1198.155	1.08017	10084.903
3	-2	6	377.220	1.07518	6324.221
3	-1	6	377.526	1.07518	6329.345
8	-6	2	380.343	1.07439	6372.568
8	-2	2	380.017	1.07439	6367.107
8	-7	1	190.445	1.06711	3173.302
8	-1	1	190.668	1.06711	3177.029
6	0	4	373.036	1.06662	6213.528
7	-4	4	170.593	1.05761	2824.309
7	-3	4	170.543	1.05761	2823.489
3	0	6	295.505	1.05697	4890.375
5	0	5	294.003	1.05203	4851.252
8	-4	3	43.042	1.04731	708.403
9	-5	0	160.215	1.04415	1316.382
9	-4	0	160.215	1.04415	1316.382
7	-5	4	446.093	1.04026	7317.533
7	-2	4	445.660	1.04026	7310.424
8	-5	3	290.751	1.03878	4766.389
8	-3	3	290.728	1.03878	4766.008
7	0	3	290.682	1.03878	4765.250
6	-3	5	1572.109	1.03495	25733.543
9	-5	1	1608.561	1.03234	26306.254
9	-4	1	1608.704	1.03234	26308.605
4	-2	6	3345.447	1.03130	54692.527
8	-7	2	1611.992	1.03060	26347.617
8	-1	2	1610.833	1.03060	26328.684
9	-3	0	996.707	1.02745	8138.029
9	-6	0	996.707	1.02745	8138.028
6	-4	5	423.773	1.02671	6918.808
6	-2	5	423.740	1.02671	6918.269
4	-3	6	599.757	1.02315	9783.884
4	-1	6	599.558	1.02315	9780.646
8	0	0	2046.829	1.01939	16683.578
9	-6	1	827.614	1.01619	13485.960
9	-3	1	827.238	1.01619	13479.824
8	-6	3	451.899	1.01439	7362.419
8	-2	3	451.856	1.01439	7361.726
8	0	1	1651.769	1.00839	26905.135

7	-6	4	855.681	1.00797	13938.000
7	-1	4	855.026	1.00797	13927.331
6	-5	5	773.983	1.00314	12610.674
6	-1	5	773.886	1.00314	12609.109
4	0	6	1105.072	0.99982	18012.574
9	-5	2	528.171	0.99918	8610.008
9	-4	2	528.059	0.99918	8608.172
9	-7	0	474.994	0.99630	3873.655
9	-2	0	474.838	0.99630	3872.384
9	-7	1	838.774	0.98603	13723.639
9	-2	1	838.945	0.98603	13726.439
9	-6	2	1100.061	0.98451	18010.035
9	-3	2	1099.858	0.98451	18006.697
5	-2	6	1283.613	0.97801	21082.697
5	-3	6	1283.613	0.97801	21082.691
8	0	2	2603.586	0.97742	42776.953
8	-7	3	1305.840	0.97729	21456.514
8	-1	3	1304.840	0.97729	21440.096
1	0	7	1520.322	0.97628	24995.475
8	-4	4	1901.456	0.97155	31357.086
6	0	5	200.277	0.96722	3313.423
8	-5	4	256.184	0.96473	4247.005
8	-3	4	255.935	0.96473	4242.870
7	0	4	255.935	0.96473	4242.870
5	-4	6	374.628	0.96424	6213.144
5	-1	6	374.586	0.96424	6212.438
2	-1	7	601.195	0.96258	9985.237
7	-3	5	400.575	0.96049	6665.985
7	-4	5	400.575	0.96049	6665.986
9	-7	2	52.216	0.95701	871.916
9	-2	2	52.210	0.95701	871.813
2	0	7	58.621	0.95595	979.949
9	-8	0	85.584	0.95448	716.474
9	-1	0	85.619	0.95448	716.761
9	-5	3	273.999	0.95037	4609.218
9	-4	3	273.999	0.95037	4609.218
7	-5	5	459.177	0.94743	7752.398
7	-2	5	459.142	0.94743	7751.807

9	-8	1	520.172	0.94544	8805.051
9	-1	1	519.210	0.94544	8788.761
8	-6	4	514.904	0.94509	8719.869
8	-2	4	514.709	0.94509	8716.578
10	-5	0	1349.805	0.94167	11483.754
5	0	6	2377.029	0.93836	40644.691
9	-6	3	1285.102	0.93772	21995.391
9	-3	3	1285.102	0.93772	21995.391
3	-2	7	1106.483	0.93683	18964.600
3	-1	7	1106.483	0.93683	18964.600
10	-6	0	1439.103	0.93545	12359.835
10	-4	0	1439.015	0.93545	12359.088
10	-5	1	472.518	0.93298	8149.709
8	0	3	248.749	0.93159	4300.511
10	-6	1	423.194	0.92693	7378.068
10	-4	1	423.775	0.92693	7388.187
6	-3	6	793.728	0.92618	13857.799
3	0	7	238.614	0.92471	4177.809
7	-6	5	49.348	0.92285	867.207
7	-1	5	49.341	0.92285	867.075
6	-4	6	374.433	0.92026	6615.105
6	-2	6	374.261	0.92026	6612.072
9	-8	2	386.988	0.91977	6844.075
9	-1	2	386.785	0.91977	6840.480
10	-7	0	503.238	0.91752	4471.641
10	-3	0	503.180	0.91752	4471.121
8	-7	4	928.912	0.91487	16606.502
8	-1	4	928.432	0.91487	16597.947
9	-7	3	680.645	0.91387	12196.433
9	-2	3	680.544	0.91387	12194.633
10	-7	1	268.152	0.90948	4856.203
10	-3	1	268.176	0.90948	4856.647
10	-5	2	515.670	0.90829	9366.779
4	-2	7	256.612	0.90737	4672.094
9	0	0	305.038	0.90612	2785.958
6	-5	6	115.532	0.90317	2127.149
6	-1	6	115.478	0.90317	2126.157
10	-6	2	111.828	0.90271	2061.614

10	-4	2	111.803	0.90271	2061.159
4	-3	7	40.778	0.90181	753.641
4	-1	7	40.774	0.90181	753.574
9	0	1	19.255	0.89837	359.403
8	-4	5	58.287	0.89465	1100.330
9	-5	4	0.509	0.89268	9.676
9	-4	4	0.509	0.89268	9.676
10	-8	0	41.970	0.88979	402.431
10	-2	0	41.967	0.88979	402.408

La4-HAp CIF FILE

```
#==============================================================================
data_global
#==============================================================================
_publ_contact_author_name  'Dr. Francesco Capitelli'
_publ_contact_author_address
;
  Institute of Crystallography - CNR
  V. Salaria Km 29.300, 00015 Monterotondo (Rome), Italy
;
_publ_contact_author_email    francesco.capitelli@ic.cnr.it
_publ_contact_author_fax     '+39 06 90672616'
_publ_contact_author_phone   '+39 06 90672630'
_journale_name_full          ?
_publ_requested_category     FI
_audit_creation_method              Expo2014

_chemical_name_systematic     ?
_chemical_formula_moiety      'H Ca4.87 La0.13 O13 P3'
_chemical_formula_sum         'H Ca4.87 La0.13 O13 P3'
_chemical_formula_weight      515.649

loop_
     _atom_type_symbol
     _atom_type_description
```

```
 _atom_type_scat_source
 'H'   'Hydrogen'   'International Tables Vol C Tables 4.2.6.8 and 6.1.1.4'
 'O'   'Oxygen'   'International Tables Vol C Tables 4.2.6.8 and 6.1.1.4'
 'P'   'Phosphorus'   'International Tables Vol C Tables 4.2.6.8 and 6.1.1.4'
 'Ca'   'Calcium'   'International Tables Vol C Tables 4.2.6.8 and 6.1.1.4'
 'La'   'Lanthanum'   'International Tables Vol C Tables 4.2.6.8 and 6.1.1.4'

_cell_length_a                   9.4194(3)
_cell_length_b                   9.4194(3)
_cell_length_c                   6.8853(4)
_cell_angle_alpha                90.000
_cell_angle_beta                 90.000
_cell_angle_gamma                120.000
_cell_volume                     529.06(4)
_cell_formula_units_Z            2
_exptl_crystal_description    powder
_exptl_crystal_colour           ?
_cell_measurement_temperature    ?

_exptl_crystal_density_diffrn         3.237
_exptl_crystal_density_meas      ?
_exptl_crystal_density_method 'not measured'
_exptl_absorpt_coefficient_mu        30.737
_symmetry_Int_Tables_number         176
_symmetry_cell_setting              hexagonal
_symmetry_space_group_name_H-M      'P 63/m'
_symmetry_space_group_name_hall     '-P 6c'

loop_
    _symmetry_equiv_pos_site_id
    _symmetry_equiv_pos_as_xyz
    1   'x, y, z'
    2   'x-y, x, z+1/2'
    3   '-y, x-y, z'
    4   '-x, -y, z+1/2'
    5   '-x+y, -x, z'
    6   'y, -x+y, z+1/2'
    7   '-x, -y, -z'
```

```
    8   '-x+y, -x, -z+1/2'
    9   'y, -x+y, -z'
   10   'x, y, -z+1/2'
   11   'x-y, x, -z'
   12   '-y, x-y, -z+1/2'

loop_
    _atom_site_type_symbol
    _atom_site_label
    _atom_site_fract_x
    _atom_site_fract_y
    _atom_site_fract_z
    _atom_site_U_iso_or_equiv
    _atom_site_occupancy
    _atom_site_adp_type
Ca   Ca1 0.6667 0.3333 0.9993(9) 0.0043(9) 1.0000        Uiso
Ca   Ca2 0.2521(4) 0.2447(4) 0.7500 0.0005 0.955(3) Uiso
La   La1 0.2521 0.2447 0.7500 0.0005(8) 0.045(3) Uiso
P    P   0.3985(6) 0.3674(6) 1.2500 0.0115(12) 1.0000        Uiso
O    O1  0.3243(11) 0.4835(11) 1.2500 0.014(3) 1.0000        Uiso
O    O2  0.5895(13) 0.4660(13) 1.2500 0.020(3) 1.0000        Uiso
O    O3  0.3386(8) 0.2530(8) 1.0699(9) 0.021(2) 1.0000        Uiso
O    O-H 0.0000 0.0000 0.706(4) 0.018(6) 0.50000       Uiso
H    H   0.0000 0.0000 0.5668 0.0213 0.50000       Uiso

loop_
    _geom_bond_atom_site_label_1
    _geom_bond_atom_site_label_2
    _geom_bond_distance
    _geom_bond_site_symmetry_2
Ca1 O1  2.405(7) 6_554
Ca1 O1  2.405(9) 4_664
Ca1 O1  2.405(9) 2_654
Ca1 O2  2.445(11) .
Ca1 O2  2.445(9) 5_665
Ca1 O2  2.445(8) 3_655
Ca1 O3  2.832(7) .
Ca1 O3  2.832(6) 5_665
```

```
Ca1 O3  2.832(9) 3_655
Ca2 O1  2.675(13) 6_554
Ca2 O2  2.364(11) 4_664
Ca2 O3  2.336(7) 10_556
Ca2 O3  2.483(9) 2_554
Ca2 O3  2.483(9) 11_557
Ca2 O3  2.336(7) .
Ca2 O-H  2.360(5) .
P O1  1.565(14) .
P O2  1.559(11) .
P O3  1.552(7) .
P O3  1.552(7) 10_557
O1 Ca1  2.405(9) 7_667
O1 Ca1  2.405(9) 2_555
O1 Ca2  2.675(8) 2_555
O2 Ca1  2.445(11) 8_667
O2 Ca2  2.364(11) 4_665
O3 Ca2  2.483(8) 6_555
O-H Ca2  2.360(5) 3_555
O-H Ca2  2.360(5) 5_555
O-H H  0.957748 .

loop_
_geom_angle_atom_site_label_1
_geom_angle_atom_site_label_2
_geom_angle_atom_site_label_3
_geom_angle
_geom_angle_site_symmetry_1
_geom_angle_site_symmetry_3
O1 Ca1 O1 74.7(3) 6_554 4_664
O1 Ca1 O1 74.7(3) 6_554 2_654
O1 Ca1 O2 123.9(3) 6_554 .
O1 Ca1 O2 154.7(3) 6_554 5_665
O1 Ca1 O2 92.9(3) 6_554 3_655
O1 Ca1 O3 68.0(3) 6_554 .
O1 Ca1 O3 141.8(3) 6_554 5_665
O1 Ca1 O3 87.5(3) 6_554 3_655
O1 Ca1 O1 74.7(3) 4_664 2_654
```

O1 Ca1 O2 92.9(3) 4_664 .

O1 Ca1 O2 123.9(3) 4_664 5_665

O1 Ca1 O2 154.7(3) 4_664 3_655

O1 Ca1 O3 87.5(3) 4_664 .

O1 Ca1 O3 68.0(3) 4_664 5_665

O1 Ca1 O3 141.8(3) 4_664 3_655

O1 Ca1 O2 154.7(3) 2_654 .

O1 Ca1 O2 92.9(3) 2_654 5_665

O1 Ca1 O2 123.9(3) 2_654 3_655

O1 Ca1 O3 141.8(3) 2_654 .

O1 Ca1 O3 87.5(3) 2_654 5_665

O1 Ca1 O3 68.0(3) 2_654 3_655

O2 Ca1 O2 75.7(3) . 5_665

O2 Ca1 O2 75.7(3) . 3_655

O2 Ca1 O3 56.8(3) . .

O2 Ca1 O3 67.3(3) . 5_665

O2 Ca1 O3 124.8(3) . 3_655

Ca1 O2 P 104.2(5) . .

Ca1 O2 Ca1 89.8(3) . 8_667

Ca1 O2 Ca2 114.3(4) . 4_665

O2 Ca1 O2 75.7(3) 5_665 3_655

O2 Ca1 O3 124.8(3) 5_665 .

O2 Ca1 O3 56.8(3) 5_665 5_665

O2 Ca1 O3 67.3(3) 5_665 3_655

O2 Ca1 O3 67.3(3) 3_655 .

O2 Ca1 O3 124.8(3) 3_655 5_665

O2 Ca1 O3 56.8(3) 3_655 3_655

O3 Ca1 O3 117.1(2) . 5_665

O3 Ca1 O3 117.1(2) . 3_655

Ca1 O3 Ca2 99.4(3) . .

Ca1 O3 P 89.0(3) . .

Ca1 O3 Ca2 98.7(3) . 6_555

O3 Ca1 O3 117.1(2) 5_665 3_655

O1 Ca2 O2 102.0(3) 6_554 4_664

O1 Ca2 O3 71.7(3) 6_554 10_556

O1 Ca2 O3 149.5(3) 6_554 2_554

O1 Ca2 O3 149.5(3) 6_554 11_557

O1 Ca2 O3 71.7(3) 6_554 .

O1 Ca2 O-H 106.2(5) 6_554 .

O2 Ca2 O3 87.3(3) 4_664 10_556

O2 Ca2 O3 74.7(3) 4_664 2_554

O2 Ca2 O3 74.7(3) 4_664 11_557

O2 Ca2 O3 87.3(3) 4_664 .

O2 Ca2 O-H 150.8(5) 4_664 .

O3 Ca2 O3 77.8(3) 10_556 2_554

O3 Ca2 O3 137.0(3) 10_556 11_557

O3 Ca2 O3 141.0(3) 10_556 .

O3 Ca2 O-H 94.3(5) 10_556 .

O3 Ca2 O3 59.9(2) 2_554 11_557

O3 Ca2 O3 137.0(3) 2_554 .

O3 Ca2 O-H 77.1(5) 2_554 .

O3 Ca2 O3 77.8(3) 11_557 .

O3 Ca2 O-H 84.6(5) 11_557 .

O3 Ca2 O-H 108.5(5) . .

Ca2 O3 P 140.9(4) . .

Ca2 O3 Ca2 119.5(3) . 6_555

Ca2 O-H Ca2 118.4(7) . 3_555

Ca2 O-H Ca2 118.4(7) . 5_555

Ca2 O-H H 97.39 . .

O1 P O2 111.7(5) . .

O1 P O3 110.6(5) . .

O1 P O3 110.6(5) . 10_557

P O1 Ca1 129.3(5) . 7_667

P O1 Ca1 129.3(5) . 2_555

P O1 Ca2 97.8(4) . 2_555

O2 P O3 108.9(5) . .

O2 P O3 108.9(5) . 10_557

P O2 Ca1 104.2(5) . 8_667

P O2 Ca2 124.2(6) . 4_665

O3 P O3 106.0(4) . 10_557

P O3 Ca2 96.5(4) . 6_555

Ca1 O1 Ca1 91.1(3) 7_667 2_555

Ca1 O1 Ca2 102.1(3) 7_667 2_555

Ca1 O1 Ca2 102.1(3) 2_555 2_555

Ca1 O2 Ca2 114.3(4) 8_667 4_665

Ca2 O-H Ca2 118.4(7) 3_555 5_555

```
Ca2 O-H H      97.39   3_555       .

Ca2 O-H H      97.39   5_555       .


loop_
   _geom_torsion_atom_site_label_1
   _geom_torsion_atom_site_label_2
   _geom_torsion_atom_site_label_3
   _geom_torsion_atom_site_label_4
   _geom_torsion
   _geom_torsion_site_symmetry_1
   _geom_torsion_site_symmetry_2
   _geom_torsion_site_symmetry_3
   _geom_torsion_site_symmetry_4
O1 Ca1 O1 Ca2 -63.6(3) 4_664 . 6_554 .
O1 Ca1 O1 Ca2 -141.6(3) 2_654 . 6_554 .
O2 Ca1 O1 Ca2 19.4(4) . . 6_554 .
O2 Ca1 O1 Ca2 155.7(7) 5_665 . 6_554 .
O2 Ca1 O1 Ca2 94.0(3) 3_655 . 6_554 .
O3 Ca1 O1 Ca2 29.9(2) . . 6_554 .
O3 Ca1 O1 Ca2 -76.4(5) 5_665 . 6_554 .
O3 Ca1 O1 Ca2 150.6(3) 3_655 . 6_554 .
O1 Ca1 O2 P 18.4(5) 6_554 . . .
O1 Ca1 O2 Ca1 123.0(3) 6_554 . . 8_667
O1 Ca1 O2 Ca2 -120.2(4) 6_554 . . 4_665
O1 Ca1 O2 P 91.8(4) 4_664 . . .
O1 Ca1 O2 Ca1 -163.5(3) 4_664 . . 8_667
O1 Ca1 O2 Ca2 -46.8(4) 4_664 . . 4_665
O1 Ca1 O2 P 151.0(7) 2_654 . . .
O1 Ca1 O2 Ca1 -104.4(7) 2_654 . . 8_667
O1 Ca1 O2 Ca2 12.3(9) 2_654 . . 4_665
O2 Ca1 O2 P -143.9(5) 5_665 . . .
O2 Ca1 O2 Ca1 -39.3(3) 5_665 . . 8_667
O2 Ca1 O2 Ca2 77.5(4) 5_665 . . 4_665
O2 Ca1 O2 P -65.3(4) 3_655 . . .
O2 Ca1 O2 Ca1 39.3(3) 3_655 . . 8_667
O2 Ca1 O2 Ca2 156.0(4) 3_655 . . 4_665
O3 Ca1 O2 P 6.7(3) . . . .
O3 Ca1 O2 Ca1 111.3(3) . . . 8_667
```

O3 Ca1 O2 Ca2 -131.9(4) . . . 4_665

O3 Ca1 O2 P 156.5(4) 5_665 . . .

O3 Ca1 O2 Ca1 -98.9(3) 5_665 . . 8_667

O3 Ca1 O2 Ca2 17.9(3) 5_665 . . 4_665

O3 Ca1 O2 P -95.3(4) 3_655 . . .

O3 Ca1 O2 Ca1 9.3(4) 3_655 . . 8_667

O3 Ca1 O2 Ca2 126.0(3) 3_655 . . 4_665

O1 Ca1 O3 Ca2 -34.5(3) 6_554 . . .

O1 Ca1 O3 P -176.1(4) 6_554 . . .

O1 Ca1 O3 Ca2 87.5(3) 6_554 . . 6_555

O1 Ca1 O3 Ca2 40.0(3) 4_664 . . .

O1 Ca1 O3 P -101.6(3) 4_664 . . .

O1 Ca1 O3 Ca2 162.0(3) 4_664 . . 6_555

O1 Ca1 O3 Ca2 -21.2(5) 2_654 . . .

O1 Ca1 O3 P -162.8(4) 2_654 . . .

O1 Ca1 O3 Ca2 100.8(5) 2_654 . . 6_555

O2 Ca1 O3 Ca2 135.1(4) . . . .

O2 Ca1 O3 P -6.5(4) . . . .

O2 Ca1 O3 Ca2 -103.0(4) . . . 6_555

O2 Ca1 O3 Ca2 170.4(4) 5_665 . . .

O2 Ca1 O3 P 28.8(5) 5_665 . . .

O2 Ca1 O3 Ca2 -67.6(4) 5_665 . . 6_555

O2 Ca1 O3 Ca2 -137.7(3) 3_655 . . .

O2 Ca1 O3 P 80.8(4) 3_655 . . .

O2 Ca1 O3 Ca2 -15.7(3) 3_655 . . 6_555

O3 Ca1 O3 Ca2 103.6(3) 5_665 . . .

O3 Ca1 O3 P -37.9(4) 5_665 . . .

O3 Ca1 O3 Ca2 -134.4(3) 5_665 . . 6_555

O3 Ca1 O3 Ca2 -109.5(3) 3_655 . . .

O3 Ca1 O3 P 109.0(3) 3_655 . . .

O3 Ca1 O3 Ca2 12.5(3) 3_655 . . 6_555

O2 Ca2 O1 Ca1 46.9(3) 4_664 . 6_554 .

O3 Ca2 O1 Ca1 130.0(3) 10_556 . 6_554 .

O3 Ca2 O1 Ca1 127.0(5) 2_554 . 6_554 .

O3 Ca2 O1 Ca1 -33.3(6) 11_557 . 6_554 .

O3 Ca2 O1 Ca1 -36.2(3) . . 6_554 .

O-H Ca2 O1 Ca1 -140.8(7) . . 6_554 .

O1 Ca2 O3 Ca1 29.8(3) 6_554 . . .

O1 Ca2 O3 P 129.9(6) 6_554 . . .

O1 Ca2 O3 Ca2 -75.9(3) 6_554 . . 6_555

O2 Ca2 O3 Ca1 -73.7(3) 4_664 . . .

O2 Ca2 O3 P 26.3(6) 4_664 . . .

O2 Ca2 O3 Ca2 -179.4(4) 4_664 . . 6_555

O3 Ca2 O3 Ca1 8.6(5) 10_556 . . .

O3 Ca2 O3 P 108.7(6) 10_556 . . .

O3 Ca2 O3 Ca2 -97.1(5) 10_556 . . 6_555

O3 Ca2 O3 Ca1 -137.8(3) 2_554 . . .

O3 Ca2 O3 P -37.7(7) 2_554 . . .

O3 Ca2 O3 Ca2 116.5(4) 2_554 . . 6_555

O3 Ca2 O3 Ca1 -148.7(3) 11_557 . . .

O3 Ca2 O3 P -48.6(6) 11_557 . . .

O3 Ca2 O3 Ca2 105.6(3) 11_557 . . 6_555

O-H Ca2 O3 Ca1 131.3(7) . . . .

O-H Ca2 O3 P -128.6(8) . . . .

O-H Ca2 O3 Ca2 25.6(7) . . . 6_555

O1 Ca2 O-H Ca2 169.6(6) 6_554 . . 3_555

O1 Ca2 O-H Ca2 14.7(8) 6_554 . . 5_555

O1 Ca2 O-H H -87.84 6_554 . . .

O2 Ca2 O-H Ca2 -26.0(15) 4_664 . . 3_555

O2 Ca2 O-H Ca2 179.1(6) 4_664 . . 5_555

O2 Ca2 O-H H 76.59 4_664 . . .

O3 Ca2 O-H Ca2 -118.2(7) 10_556 . . 3_555

O3 Ca2 O-H Ca2 86.9(7) 10_556 . . 5_555

O3 Ca2 O-H H -15.61 10_556 . . .

O3 Ca2 O-H Ca2 -41.7(7) 2_554 . . 3_555

O3 Ca2 O-H Ca2 163.4(8) 2_554 . . 5_555

O3 Ca2 O-H H 60.86 2_554 . . .

O3 Ca2 O-H Ca2 18.7(7) 11_557 . . 3_555

O3 Ca2 O-H Ca2 -136.2(8) 11_557 . . 5_555

O3 Ca2 O-H H 121.20 11_557 . . .

O3 Ca2 O-H Ca2 93.9(7) . . . 3_555

O3 Ca2 O-H Ca2 -61.0(8) . . . 5_555

O3 Ca2 O-H H -163.57 . . . .

O2 P O1 Ca1 67.3(6) . . . 7_667

O2 P O1 Ca1 -67.3(6) . . . 2_555

O2 P O1 Ca2 180.0(4) . . . 2_555

```
O3 P O1 Ca1 -54.1(6) . . . 7_667
O3 P O1 Ca1 171.3(4) . . . 2_555
O3 P O1 Ca2 58.6(4) . . . 2_555
O3 P O1 Ca1 -171.3(4) 10_557 . . 7_667
O3 P O1 Ca1 54.1(6) 10_557 . . 2_555
O3 P O1 Ca2 -58.6(4) 10_557 . . 2_555
O1 P O2 Ca1 -133.3(4) . . . .
O1 P O2 Ca1 133.3(4) . . . 8_667
O1 P O2 Ca2 0.0(6) . . . 4_665
O3 P O2 Ca1 -10.9(5) . . . .
O3 P O2 Ca1 -104.3(4) . . . 8_667
O3 P O2 Ca2 122.4(5) . . . 4_665
O3 P O2 Ca1 104.3(4) 10_557 . . .
O3 P O2 Ca1 10.9(5) 10_557 . . 8_667
O3 P O2 Ca2 -122.4(5) 10_557 . . 4_665
O1 P O3 Ca1 132.1(4) . . . .
O1 P O3 Ca2 28.4(7) . . . .
O1 P O3 Ca2 -129.2(4) . . . 6_555
O2 P O3 Ca1 9.1(4) . . . .
O2 P O3 Ca2 -94.6(6) . . . .
O2 P O3 Ca2 107.8(4) . . . 6_555
O3 P O3 Ca1 -108.0(3) 10_557 . . .
O3 P O3 Ca2 148.3(5) 10_557 . . .
O3 P O3 Ca2 -9.3(4) 10_557 . . 6_555

_diffrn_ambient_temperature     ?
_diffrn_radiation_wavelength     1.540560
_diffrn_radiation_type          'Cu K\a~1~'
_diffrn_measurement_device_type ?

# POWDER PROFILE

_pd_meas_2theta_range_min      6.000
_pd_meas_2theta_range_max    120.000
_pd_proc_number_of_points       5701

_pd_proc_ls_prof_R_factor           0.04750
_pd_proc_ls_prof_wR_factor          0.07636
```

```
_pd_proc_ls_prof_wR_expected        0.05438

_refine_ls_number_reflns      288
_refine_ls_number_parameters  57
_refine_ls_number_restraints  0
_refine_ls_hydrogen_treatment  constr
_refine_ls_R_I_factor               0.21709
_refine_ls_goodness_of_fit_all      1.40421
_refine_ls_restrained_S_all         1.40421

loop_
     _pd_meas_2theta_scan
     _pd_meas_counts_total
     _pd_proc_2theta_corrected
     _pd_calc_intensity_total
     _pd_proc_intensity_bkg_calc

        6.000000     2181.330078        6.006184        0.000000     2176.454590
        6.020000     2174.830078        6.026183        0.000000     2169.656738
        6.040000     2173.670166        6.046183        0.000000     2162.898926
        6.060000     2154.000000        6.066183        0.000000     2156.176270
        6.080000     2170.500000        6.086183        0.000000     2149.491455
        6.100000     2160.000000        6.106183        0.000000     2142.843506
        6.120000     2148.670166        6.126183        0.000000     2136.231445
        6.140000     2147.170166        6.146183        0.000000     2129.657227
        6.160000     2126.830078        6.166183        0.000000     2123.116699
        6.180000     2124.000000        6.186182        0.000000     2116.611816
        6.200000     2071.330078        6.206182        0.000000     2110.143066
        6.220000     2082.170166        6.226182        0.000000     2103.710449
        6.240000     2050.170166        6.246182        0.000000     2097.310059
        6.260000     2084.170166        6.266182        0.000000     2090.946777
        6.280000     2094.000000        6.286182        0.000000     2084.616211
        6.300000     2100.330078        6.306182        0.000000     2078.320068
        6.320000     2100.170166        6.326182        0.000000     2072.058105
        6.340000     2051.830078        6.346182        0.000000     2065.828613
        6.360000     2059.670166        6.366182        0.000000     2059.632324
        6.380000     2030.830078        6.386181        0.000000     2053.470947
        6.400000     2057.170166        6.406181        0.000000     2047.339966
```

6.420000	2047.330078	6.426181	0.000000	2041.242554
6.440000	2038.669922	6.446181	0.000000	2035.177612
6.460000	2046.169922	6.466181	0.000000	2029.144409
6.480000	1985.669922	6.486181	0.000000	2023.142700
6.500000	2016.000000	6.506181	0.000000	2017.173218
6.520000	2009.000000	6.526180	0.000000	2011.234009
6.540000	2035.000000	6.546181	0.000000	2005.326538
6.560000	2038.330078	6.566181	0.000000	1999.449829
6.580000	1976.669922	6.586181	0.000000	1993.602905
6.600000	1995.330078	6.606180	0.000000	1987.786499
6.620000	1955.500000	6.626180	0.000000	1982.001831
6.640000	1984.500000	6.646180	0.000000	1976.245483
6.660000	1948.500000	6.666180	0.000000	1970.518921
6.680000	1936.500000	6.686180	0.000000	1964.822632
6.700000	1980.830078	6.706180	0.000000	1959.154419
6.720000	1968.500000	6.726180	0.000000	1953.515015
6.740000	1948.000000	6.746179	0.000000	1947.905884
6.760000	1937.169922	6.766179	0.000000	1942.325073
6.780000	1934.330078	6.786179	0.000000	1936.772339
6.800000	1920.669922	6.806180	0.000000	1931.246948
6.820000	1931.000000	6.826179	0.000000	1925.750122
6.840000	1901.669922	6.846179	0.000000	1920.281860
6.860000	1914.169922	6.866179	0.000000	1914.839478
6.880000	1938.169922	6.886179	0.000000	1909.424927
6.900000	1886.169922	6.906179	0.000000	1904.038452
6.920000	1905.500000	6.926179	0.000000	1898.678101
6.940000	1912.669922	6.946179	0.000000	1893.344116
6.960000	1888.169922	6.966178	0.000000	1888.038208
6.980000	1890.330078	6.986178	0.000000	1882.757202
7.000000	1871.669922	7.006178	0.000000	1877.502319
7.020000	1864.669922	7.026178	0.000000	1872.274292
7.040000	1863.830078	7.046178	0.000000	1867.071167
7.060000	1828.000000	7.066178	0.000000	1861.893677
7.080000	1847.500000	7.086178	0.000000	1856.742798
7.100000	1862.830078	7.106177	0.000000	1851.615356
7.120000	1826.000000	7.126177	0.000000	1846.513306
7.140000	1835.000000	7.146177	0.000000	1841.437378
7.160000	1850.330078	7.166177	0.000000	1836.384399

7.180000	1846.669922	7.186177	0.000000	1831.357056
7.200000	1824.669922	7.206177	0.000000	1826.354126
7.220000	1854.330078	7.226177	0.000000	1821.374146
7.240000	1808.330078	7.246177	0.000000	1816.419312
7.260000	1805.330078	7.266177	0.000000	1811.487671
7.280000	1825.330078	7.286177	0.000000	1806.579468
7.300000	1759.330078	7.306177	0.000000	1801.694458
7.320000	1805.830078	7.326177	0.000000	1796.833862
7.340000	1806.330078	7.346177	0.000000	1791.995483
7.360000	1797.330078	7.366177	0.000000	1787.180054
7.380000	1769.169922	7.386176	0.000000	1782.387817
7.400000	1742.330078	7.406176	0.000000	1777.618042
7.420000	1745.330078	7.426176	0.000000	1772.870972
7.440000	1784.669922	7.446176	0.000000	1768.145386
7.460000	1774.500000	7.466176	0.000000	1763.442017
7.480000	1780.330078	7.486176	0.000000	1758.761353
7.500000	1757.169922	7.506176	0.000000	1754.101685
7.520000	1769.330078	7.526176	0.000000	1749.463501
7.540000	1729.500000	7.546175	0.000000	1744.848022
7.560000	1728.169922	7.566175	0.000000	1740.252563
7.580000	1728.500000	7.586175	0.000000	1735.679077
7.600000	1733.669922	7.606175	0.000000	1731.126587
7.620000	1748.169922	7.626175	0.000000	1726.594849
7.640000	1726.169922	7.646175	0.000000	1722.083374
7.660000	1731.330078	7.666175	0.000000	1717.593872
7.680000	1723.000000	7.686174	0.000000	1713.123413
7.700000	1710.500000	7.706174	0.000000	1708.673462
7.720000	1716.669922	7.726174	0.000000	1704.244507
7.740000	1686.000000	7.746174	0.000000	1699.834839
7.760000	1693.330078	7.766174	0.000000	1695.445923
7.780000	1700.000000	7.786174	0.000000	1691.075562
7.800000	1675.830078	7.806175	0.000000	1686.725220
7.820000	1654.330078	7.826174	0.000000	1682.393921
7.840000	1677.169922	7.846174	0.000000	1678.083130
7.860000	1697.169922	7.866174	0.000000	1673.790405
7.880000	1677.330078	7.886174	0.000000	1669.517456
7.900000	1671.669922	7.906174	0.000000	1665.263306
7.920000	1665.669922	7.926174	0.000000	1661.027710

7.940000	1648.669922	7.946174	0.000000	1656.810669
7.960000	1670.669922	7.966173	0.000000	1652.613159
7.980000	1645.500000	7.986173	0.000000	1648.432983
8.000000	1646.330078	8.006173	0.000000	1644.271606
8.020000	1643.000000	8.026174	0.000000	1640.128540
8.040000	1621.669922	8.046173	0.000000	1636.003296
8.060000	1633.500000	8.066174	0.000000	1631.895630
8.080000	1640.830078	8.086172	0.000000	1627.806519
8.100000	1637.330078	8.106173	0.000000	1623.734253
8.120001	1630.669922	8.126173	0.000000	1619.679810
8.140000	1633.000000	8.146173	0.000000	1615.643677
8.160001	1628.000000	8.166173	0.000000	1611.623657
8.180000	1596.669922	8.186172	0.000000	1607.621704
8.200000	1595.169922	8.206173	0.000000	1603.636108
8.220000	1599.500000	8.226172	0.000000	1599.667847
8.240000	1591.500000	8.246172	0.000000	1595.716919
8.260000	1602.830078	8.266172	0.000000	1591.782104
8.280000	1564.500000	8.286172	0.000000	1587.863892
8.300000	1585.830078	8.306172	0.000000	1583.962280
8.320001	1566.500000	8.326173	0.000000	1580.077515
8.340000	1576.669922	8.346172	0.000000	1576.208374
8.360001	1586.000000	8.366172	0.000000	1572.356323
8.380000	1562.330078	8.386171	0.000000	1568.519653
8.400000	1547.000000	8.406171	0.000000	1564.699829
8.420000	1554.500000	8.426171	0.000000	1560.894653
8.440000	1564.000000	8.446171	0.000000	1557.105591
8.460000	1537.330078	8.466171	0.000000	1553.332153
8.480000	1559.000000	8.486171	0.000000	1549.574829
8.500000	1561.169922	8.506170	0.000000	1545.832642
8.520000	1507.000000	8.526171	0.000000	1542.105835
8.540000	1536.169922	8.546170	0.000000	1538.394409
8.560000	1542.669922	8.566171	0.000000	1534.697632
8.580000	1525.500000	8.586170	0.000000	1531.016968
8.600000	1532.669922	8.606171	0.000000	1527.350464
8.620001	1528.169922	8.626171	0.000000	1523.698364
8.640000	1525.500000	8.646170	0.000000	1520.062134
8.660001	1516.669922	8.666170	0.000000	1516.440063
8.680000	1507.669922	8.686170	0.000000	1512.832397

8.700000	1508.000000	8.706169	0.000000	1509.239624
8.720000	1492.500000	8.726170	0.000000	1505.661011
8.740000	1505.169922	8.746170	0.000000	1502.096558
8.760000	1477.000000	8.766171	0.000000	1498.546021
8.780000	1477.500000	8.786170	0.000000	1495.010864
8.800000	1481.330078	8.806170	0.000000	1491.488647
8.820001	1509.169922	8.826170	0.000000	1487.980347
8.840000	1490.330078	8.846169	0.000000	1484.486694
8.860001	1484.669922	8.866170	0.000000	1481.005981
8.880000	1476.500000	8.886169	0.000000	1477.539185
8.900000	1473.000000	8.906169	0.000000	1474.086060
8.920000	1450.830078	8.926169	0.000000	1470.646851
8.940000	1454.000000	8.946168	0.000000	1467.220825
8.960000	1458.000000	8.966168	0.000000	1463.807739
8.980000	1473.669922	8.986168	0.000000	1460.407837
9.000000	1454.669922	9.006168	0.000000	1457.022095
9.020000	1466.830078	9.026169	0.000000	1453.648560
9.040000	1466.169922	9.046168	0.000000	1450.287720
9.060000	1451.830078	9.066169	0.000000	1446.940552
9.080000	1432.330078	9.086167	0.000000	1443.606201
9.100000	1449.000000	9.106168	0.000000	1440.284180
9.120001	1429.669922	9.126168	0.000000	1436.975342
9.140000	1433.000000	9.146168	0.000000	1433.678955
9.160001	1431.000000	9.166168	0.000000	1430.394653
9.180000	1407.830078	9.186168	0.000000	1427.123535
9.200000	1430.330078	9.206167	0.000000	1423.864258
9.220000	1414.500000	9.226167	0.000000	1420.617798
9.240000	1397.169922	9.246167	0.000000	1417.383057
9.260000	1445.000000	9.266168	0.000000	1414.160278
9.280000	1439.830078	9.286167	0.000000	1410.949951
9.300000	1431.169922	9.306168	0.000000	1407.751953
9.320001	1387.330078	9.326168	0.000000	1404.565308
9.340000	1372.669922	9.346168	0.000000	1401.390503
9.360001	1392.500000	9.366167	0.000000	1398.228149
9.380000	1387.330078	9.386167	0.000000	1395.076904
9.400000	1408.330078	9.406166	0.000000	1391.938232
9.420000	1373.830078	9.426167	0.000000	1388.810059
9.440000	1364.330078	9.446166	0.000000	1385.693848

9.460000	1383.830078	9.466166	0.000000	1382.588745
9.480000	1387.830078	9.486166	0.000000	1379.495728
9.500000	1384.830078	9.506166	0.000000	1376.413574
9.520000	1378.330078	9.526166	0.000000	1373.342041
9.540000	1355.000000	9.546165	0.000000	1370.283081
9.560000	1352.169922	9.566166	0.000000	1367.234253
9.580000	1375.330078	9.586165	0.000000	1364.197021
9.600000	1337.000000	9.606166	0.000000	1361.170532
9.620001	1353.169922	9.626166	0.000000	1358.154907
9.640000	1351.830078	9.646166	0.000000	1355.150024
9.660001	1357.000000	9.666165	0.000000	1352.156372
9.680000	1349.500000	9.686165	0.000000	1349.172852
9.700000	1353.000000	9.706165	0.000000	1346.200195
9.720000	1352.000000	9.726166	0.000000	1343.238647
9.740000	1333.330078	9.746165	0.000000	1340.287109
9.760000	1323.330078	9.766166	0.000000	1337.346191
9.780000	1337.500000	9.786165	0.000000	1334.416138
9.800000	1322.500000	9.806165	0.000000	1331.495850
9.820001	1324.330078	9.826165	0.000000	1328.586060
9.840000	1324.330078	9.846165	0.000000	1325.687012
9.860001	1329.330078	9.866165	0.000000	1322.797363
9.880000	1295.169922	9.886165	0.000000	1319.918091
9.900000	1300.000000	9.906164	0.000000	1317.049561
9.920000	1316.830078	9.926165	0.000000	1314.190430
9.940000	1315.669922	9.946163	0.000000	1311.341553
9.960000	1322.169922	9.966164	0.000000	1308.502441
9.980000	1286.500000	9.986163	0.000000	1305.673584
10.000000	1320.000000	10.006164	0.000000	1302.854004
10.020000	1308.830078	10.026164	0.000000	1300.044189
10.040000	1304.500000	10.046164	0.000000	1297.243774
10.060000	1296.500000	10.066164	0.000000	1294.454224
10.080000	1288.169922	10.086163	0.000000	1291.673340
10.100000	1293.169922	10.106163	0.000000	1288.902466
10.120001	1294.000000	10.126163	0.000000	1286.140625
10.140000	1273.169922	10.146163	0.000000	1283.388306
10.160001	1272.330078	10.166164	0.000000	1280.645264
10.180000	1300.169922	10.186164	0.000000	1277.911621
10.200000	1272.330078	10.206163	0.000000	1275.187866

10.220000	1266.330078	10.226164	0.000000	1272.472656
10.240000	1280.169922	10.246162	0.000000	1269.767090
10.260000	1272.000000	10.266163	0.000000	1267.070557
10.280000	1264.830078	10.286162	0.000000	1264.383545
10.300000	1239.830078	10.306163	0.000000	1261.704712
10.320001	1273.500000	10.326163	0.000000	1259.035034
10.340000	1248.330078	10.346163	0.000000	1256.374634
10.360001	1256.500000	10.366163	0.000000	1253.722778
10.380000	1242.830078	10.386162	0.000000	1251.080322
10.400000	1240.830078	10.406161	0.000000	1248.446777
10.420000	1247.169922	10.426162	0.000000	1245.821411
10.440000	1242.500000	10.446161	0.000000	1243.204834
10.460000	1231.330078	10.466162	0.000000	1240.597412
10.480000	1220.500000	10.486161	0.000000	1237.998291
10.500000	1243.000000	10.506162	0.000000	1235.407593
10.520000	1231.500000	10.526161	0.000000	1232.826050
10.540000	1243.000000	10.546161	0.000001	1230.252441
10.560000	1235.330078	10.566161	0.000003	1227.687500
10.580000	1206.830078	10.586162	0.000010	1225.131104
10.600000	1219.330078	10.606162	0.000032	1222.582642
10.620001	1228.169922	10.626163	0.000110	1220.042847
10.640000	1231.000000	10.646162	0.000415	1217.511230
10.660001	1218.500000	10.666162	0.001718	1214.988037
10.680000	1219.169922	10.686161	0.007949	1212.473022
10.700000	1257.669922	10.706161	0.042342	1209.966553
10.720000	1253.669922	10.726161	0.273365	1207.467407
10.740000	1271.830078	10.746161	2.127624	1204.976685
10.760000	1300.830078	10.766161	16.729122	1202.494019
10.780000	1336.000000	10.786160	99.823181	1200.019409
10.800000	1422.669922	10.806161	344.516937	1197.552734
10.820001	1507.669922	10.826160	575.876221	1195.094360
10.840000	1487.169922	10.846160	402.198273	1192.643311
10.860001	1361.000000	10.866160	82.092804	1190.200195
10.880000	1257.169922	10.886160	1.102842	1187.765381
10.900000	1197.669922	10.906159	0.907206	1185.337891
10.920000	1174.330078	10.926160	0.719181	1182.918213
10.940000	1186.500000	10.946159	0.197435	1180.506592
10.960000	1157.830078	10.966159	0.041276	1178.101929

10.980000	1176.000000	10.986158	0.008410	1175.705811
11.000000	1164.169922	11.006160	0.001839	1173.316772
11.020000	1175.169922	11.026160	0.000443	1170.935181
11.040000	1162.669922	11.046160	0.000117	1168.560913
11.060000	1149.000000	11.066160	0.000034	1166.194580
11.080000	1146.500000	11.086160	0.000011	1163.835693
11.100000	1157.500000	11.106159	0.000004	1161.484253
11.120001	1165.669922	11.126160	0.000001	1159.140381
11.140000	1129.169922	11.146159	0.000000	1156.803589
11.160001	1164.169922	11.166160	0.000000	1154.473999
11.180000	1153.169922	11.186159	0.000000	1152.152100
11.200000	1182.330078	11.206159	0.000000	1149.836914
11.220000	1150.330078	11.226159	0.000000	1147.529175
11.240000	1143.500000	11.246158	0.000000	1145.228882
11.260000	1169.830078	11.266158	0.000000	1142.935425
11.280000	1130.330078	11.286158	0.000000	1140.649658
11.300000	1136.169922	11.306158	0.000000	1138.370361
11.320001	1129.330078	11.326159	0.000000	1136.098145
11.340000	1128.000000	11.346158	0.000000	1133.833252
11.360001	1126.669922	11.366158	0.000000	1131.575317
11.380000	1130.000000	11.386158	0.000000	1129.324463
11.400000	1117.500000	11.406157	0.000000	1127.079834
11.420000	1120.330078	11.426158	0.000000	1124.843018
11.440000	1118.169922	11.446157	0.000000	1122.612427
11.460000	1134.000000	11.466158	0.000000	1120.389160
11.480000	1122.500000	11.486157	0.000000	1118.172852
11.500000	1125.000000	11.506158	0.000000	1115.962646
11.520000	1129.330078	11.526158	0.000000	1113.759644
11.540000	1106.669922	11.546157	0.000000	1111.563843
11.560000	1101.000000	11.566157	0.000000	1109.373901
11.580000	1098.330078	11.586157	0.000000	1107.191162
11.600000	1111.330078	11.606157	0.000000	1105.014771
11.620001	1105.330078	11.626158	0.000000	1102.844971
11.640000	1088.669922	11.646157	0.000000	1100.681763
11.660001	1108.830078	11.666158	0.000000	1098.525391
11.680000	1109.669922	11.686156	0.000000	1096.375000
11.700000	1113.500000	11.706156	0.000000	1094.231812
11.720000	1105.330078	11.726156	0.000000	1092.094971

11.740000	1078.000000	11.746156	0.000000	1089.964111
11.760000	1086.669922	11.766156	0.000000	1087.840332
11.780000	1090.500000	11.786157	0.000000	1085.722534
11.800000	1065.000000	11.806157	0.000000	1083.610840
11.820001	1087.500000	11.826157	0.000000	1081.506104
11.840000	1084.830078	11.846156	0.000000	1079.407593
11.860001	1060.830078	11.866157	0.000000	1077.314941
11.880000	1089.500000	11.886156	0.000000	1075.228760
11.900000	1051.830078	11.906156	0.000000	1073.149048
11.920000	1072.669922	11.926156	0.000000	1071.075439
11.940000	1087.169922	11.946156	0.000000	1069.008301
11.960000	1059.169922	11.966156	0.000000	1066.946899
11.980000	1075.000000	11.986155	0.000000	1064.892090
12.000000	1070.169922	12.006155	0.000000	1062.843018
12.020000	1096.330078	12.026155	0.000000	1060.799927
12.040000	1079.330078	12.046155	0.000000	1058.763184
12.060000	1089.500000	12.066155	0.000000	1056.732300
12.080000	1085.000000	12.086155	0.000000	1054.707764
12.100000	1091.330078	12.106155	0.000000	1052.688965
12.120001	1072.500000	12.126155	0.000000	1050.676514
12.140000	1043.330078	12.146154	0.000000	1048.669800
12.160001	1048.000000	12.166156	0.000000	1046.668701
12.180000	1019.169983	12.186155	0.000000	1044.673706
12.200000	1036.669922	12.206155	0.000000	1042.685059
12.220000	1043.330078	12.226155	0.000000	1040.701904
12.240000	1045.500000	12.246155	0.000000	1038.724243
12.260000	1041.500000	12.266154	0.000000	1036.753296
12.280000	1026.000000	12.286154	0.000000	1034.787598
12.300000	1029.169922	12.306154	0.000000	1032.827759
12.320001	1022.330017	12.326155	0.000000	1030.873535
12.340000	1037.669922	12.346154	0.000000	1028.925171
12.360001	1043.330078	12.366155	0.000000	1026.982422
12.380000	997.000000	12.386154	0.000000	1025.045654
12.400000	1043.000000	12.406154	0.000000	1023.114258
12.420000	1016.669983	12.426153	0.000000	1021.188965
12.440000	1030.500000	12.446153	0.000000	1019.269043
12.460000	1025.669922	12.466153	0.000000	1017.354980
12.480000	1029.169922	12.486153	0.000000	1015.445923

12.500000	1002.669983	12.506153	0.000000	1013.542847
12.520000	996.166992	12.526155	0.000000	1011.644897
12.540000	984.666992	12.546154	0.000000	1009.752930
12.560000	991.000000	12.566154	0.000000	1007.866577
12.580000	1026.500000	12.586153	0.000000	1005.985474
12.600000	1001.330017	12.606153	0.000000	1004.110107
12.620001	994.666992	12.626154	0.000000	1002.239868
12.640000	1000.169983	12.646153	0.000000	1000.375000
12.660001	1001.000000	12.666154	0.000000	998.515625
12.680000	1021.330017	12.686153	0.000000	996.662109
12.700000	1024.830078	12.706152	0.000000	994.813965
12.720000	987.833008	12.726152	0.000000	992.970581
12.740000	971.500000	12.746152	0.000000	991.132690
12.760000	978.666992	12.766152	0.000000	989.300171
12.780000	980.833008	12.786152	0.000000	987.473267
12.800000	980.333008	12.806152	0.000000	985.651489
12.820001	998.000000	12.826153	0.000000	983.834717
12.840000	996.833008	12.846151	0.000000	982.023438
12.860001	997.500000	12.866152	0.000000	980.217407
12.880000	967.500000	12.886152	0.000000	978.416260
12.900000	985.000000	12.906152	0.000000	976.620972
12.920000	963.833008	12.926152	0.000000	974.830566
12.940000	976.500000	12.946152	0.000000	973.045410
12.960000	999.500000	12.966152	0.000000	971.265259
12.980000	969.666992	12.986152	0.000000	969.490112
13.000000	971.000000	13.006151	0.000000	967.720337
13.020000	973.500000	13.026152	0.000000	965.955444
13.040000	949.500000	13.046151	0.000000	964.195679
13.060000	956.000000	13.066152	0.000000	962.440918
13.080000	956.666992	13.086151	0.000000	960.691528
13.100000	961.833008	13.106152	0.000000	958.946899
13.120001	955.666992	13.126152	0.000000	957.207275
13.140000	944.666992	13.146151	0.000000	955.472900
13.160001	942.166992	13.166151	0.000000	953.743164
13.180000	945.166992	13.186151	0.000000	952.019043
13.200000	958.333008	13.206150	0.000000	950.299072
13.220000	964.333008	13.226151	0.000000	948.584351
13.240000	934.000000	13.246151	0.000000	946.874634

13.260000	939.166992	13.266151	0.000000	945.169678
13.280000	959.166992	13.286150	0.000000	943.469604
13.300000	961.166992	13.306150	0.000000	941.774902
13.320001	953.500000	13.326151	0.000000	940.084595
13.340000	931.166992	13.346150	0.000000	938.399170
13.360001	933.333008	13.366151	0.000000	936.718384
13.380000	940.666992	13.386150	0.000000	935.042969
13.400000	923.333008	13.406150	0.000000	933.371826
13.420000	918.166992	13.426150	0.000000	931.705811
13.440000	913.833008	13.446149	0.000000	930.044312
13.460000	946.500000	13.466149	0.000000	928.387695
13.480000	927.333008	13.486149	0.000000	926.735962
13.500000	941.500000	13.506149	0.000000	925.088745
13.520000	948.166992	13.526150	0.000000	923.446167
13.540000	933.000000	13.546149	0.000000	921.808716
13.560000	920.333008	13.566151	0.000000	920.175293
13.580000	931.000000	13.586149	0.000000	918.546997
13.600000	933.500000	13.606150	0.000000	916.923340
13.620001	934.166992	13.626150	0.000000	915.303955
13.640000	930.666992	13.646150	0.000000	913.689697
13.660001	935.333008	13.666150	0.000000	912.079712
13.680000	896.833008	13.686150	0.000000	910.474487
13.700000	897.833008	13.706149	0.000000	908.873901
13.720000	900.500000	13.726149	0.000000	907.277832
13.740000	901.166992	13.746148	0.000000	905.686157
13.760000	896.666992	13.766149	0.000000	904.099121
13.780000	902.333008	13.786148	0.000000	902.516846
13.800000	897.166992	13.806149	0.000000	900.938721
13.820001	902.000000	13.826149	0.000000	899.365234
13.840000	904.166992	13.846148	0.000000	897.796631
13.860001	905.000000	13.866149	0.000000	896.231812
13.880000	894.333008	13.886148	0.000000	894.671875
13.900000	868.833008	13.906148	0.000000	893.116333
13.920000	904.000000	13.926148	0.000000	891.565430
13.940000	898.666992	13.946148	0.000000	890.018555
13.960000	897.833008	13.966148	0.000000	888.476440
13.980000	902.833008	13.986148	0.000000	886.938354
14.000000	905.333008	14.006148	0.000000	885.404907

14.020000	888.666992	14.026148	0.000000	883.875854
14.040000	897.333008	14.046147	0.000000	882.351074
14.060000	880.166992	14.066148	0.000000	880.830566
14.080000	892.500000	14.086147	0.000000	879.314697
14.100000	881.166992	14.106148	0.000000	877.802856
14.120001	875.666992	14.126148	0.000000	876.295410
14.140000	894.000000	14.146148	0.000000	874.792603
14.160001	867.666992	14.166147	0.000000	873.293457
14.180000	871.666992	14.186147	0.000000	871.799194
14.200000	878.166992	14.206147	0.000000	870.308960
14.220000	868.500000	14.226148	0.000000	868.822754
14.240000	875.333008	14.246147	0.000000	867.340942
14.260000	865.333008	14.266148	0.000000	865.863647
14.280000	858.666992	14.286147	0.000000	864.390137
14.300000	846.000000	14.306148	0.000000	862.920898
14.320001	873.000000	14.326147	0.000000	861.456299
14.340000	895.333008	14.346147	0.000000	859.995361
14.360001	860.833008	14.366147	0.000000	858.538818
14.380000	857.500000	14.386147	0.000000	857.086548
14.400000	861.333008	14.406146	0.000000	855.638184
14.420000	873.333008	14.426147	0.000000	854.194092
14.440000	859.000000	14.446146	0.000000	852.754150
14.460000	849.166992	14.466146	0.000000	851.318237
14.480000	870.333008	14.486145	0.000000	849.886230
14.500000	850.333008	14.506145	0.000000	848.458984
14.520000	841.666992	14.526147	0.000000	847.035034
14.540000	827.500000	14.546146	0.000000	845.615601
14.560000	824.000000	14.566147	0.000000	844.199829
14.580000	840.833008	14.586146	0.000000	842.788696
14.600000	839.000000	14.606146	0.000000	841.381226
14.620001	828.666992	14.626146	0.000000	839.978027
14.640000	831.500000	14.646146	0.000000	838.578613
14.660001	839.500000	14.666146	0.000000	837.183350
14.680000	844.833008	14.686146	0.000000	835.791992
14.700000	826.500000	14.706145	0.000000	834.404541
14.720000	834.000000	14.726146	0.000000	833.020996
14.740000	826.166992	14.746145	0.000000	831.641846
14.760000	849.000000	14.766145	0.000000	830.266113

14.780000	841.333008	14.786144	0.000000	828.894653
14.800000	833.333008	14.806145	0.000000	827.526978
14.820001	836.500000	14.826146	0.000000	826.163330
14.840000	830.333008	14.846146	0.000000	824.803345
14.860001	832.166992	14.866146	0.000000	823.447510
14.880000	800.166992	14.886146	0.000000	822.095581
14.900000	824.833008	14.906144	0.000000	820.747314
14.920000	821.666992	14.926145	0.000000	819.403076
14.940000	796.333008	14.946144	0.000000	818.062744
14.960000	803.833008	14.966145	0.000000	816.726074
14.980000	799.166992	14.986144	0.000000	815.393677
15.000000	818.833008	15.006145	0.000000	814.064453
15.020000	822.166992	15.026145	0.000000	812.739380
15.040000	805.666992	15.046144	0.000000	811.418091
15.060000	805.500000	15.066144	0.000000	810.100464
15.080000	795.333008	15.086143	0.000000	808.786499
15.100000	809.333008	15.106144	0.000000	807.476807
15.120001	804.000000	15.126145	0.000000	806.170410
15.140000	803.000000	15.146145	0.000000	804.868042
15.160001	791.166992	15.166145	0.000000	803.569336
15.180000	799.833008	15.186145	0.000000	802.274414
15.200000	810.333008	15.206143	0.000000	800.983032
15.220000	798.166992	15.226144	0.000000	799.695801
15.240000	770.333008	15.246143	0.000000	798.411865
15.260000	775.000000	15.266144	0.000000	797.131592
15.280000	772.333008	15.286143	0.000000	795.855225
15.300000	779.166992	15.306144	0.000000	794.582397
15.320001	793.500000	15.326144	0.000000	793.313232
15.340000	761.000000	15.346143	0.000000	792.047852
15.360001	775.833008	15.366143	0.000000	790.786011
15.380000	789.333008	15.386143	0.000000	789.527710
15.400000	788.166992	15.406142	0.000000	788.273315
15.420000	778.333008	15.426144	0.000000	787.022217
15.440000	797.000000	15.446143	0.000000	785.774780
15.460000	796.000000	15.466144	0.000000	784.531128
15.480000	765.500000	15.486143	0.000000	783.291138
15.500000	788.500000	15.506143	0.000000	782.054199
15.520000	764.833008	15.526143	0.000000	780.821411

15.540000	796.500000	15.546143	0.000000	779.591919
15.560000	782.500000	15.566143	0.000000	778.365967
15.580000	780.000000	15.586143	0.000000	777.143677
15.600000	795.166992	15.606143	0.076346	775.924683
15.620001	782.000000	15.626143	0.079093	774.709229
15.640000	763.666992	15.646142	0.081986	773.497559
15.660001	773.166992	15.666142	0.085037	772.289307
15.680000	761.000000	15.686142	0.088257	771.084229
15.700000	757.666992	15.706142	0.091658	769.882935
15.720000	773.500000	15.726143	0.095255	768.684814
15.740000	774.000000	15.746142	0.099062	767.490601
15.760000	773.333008	15.766143	0.103096	766.299316
15.780000	763.500000	15.786141	0.107375	765.112061
15.800000	769.333008	15.806142	0.111919	763.927612
15.820001	761.166992	15.826142	0.116750	762.747437
15.840000	755.500000	15.846142	0.121892	761.570068
15.860001	756.500000	15.866142	0.127373	760.396118
15.880000	762.666992	15.886142	0.133221	759.225708
15.900000	767.833008	15.906141	0.139471	758.058472
15.920000	758.166992	15.926142	0.146159	756.894653
15.940000	757.000000	15.946140	0.153327	755.734619
15.960000	745.666992	15.966141	0.161022	754.577637
15.980000	744.833008	15.986141	0.169295	753.423706
16.000000	762.333008	16.006142	0.178205	752.273682
16.020000	749.500000	16.026142	0.187818	751.126709
16.040001	748.500000	16.046143	0.198209	749.982910
16.059999	739.500000	16.066141	0.209462	748.842773
16.080000	744.166992	16.086142	0.221676	747.705688
16.100000	749.500000	16.106142	0.234960	746.571899
16.120001	746.666992	16.126143	0.249442	745.441406
16.140001	760.166992	16.146143	0.265267	744.314209
16.160000	752.000000	16.166142	0.282604	743.190430
16.180000	743.833008	16.186142	0.301651	742.069824
16.200001	739.333008	16.206142	0.322636	740.952393
16.220001	728.333008	16.226143	0.345827	739.838379
16.240002	729.666992	16.246143	0.371539	738.727417
16.260000	748.166992	16.266142	0.400142	737.619629
16.280001	736.666992	16.286142	0.432087	736.515259

16.299999	743.833008	16.306139	0.467895	735.414185
16.320000	720.000000	16.326139	0.508222	734.315918
16.340000	727.833008	16.346140	0.553840	733.221191
16.359999	729.166992	16.366138	0.605695	732.129395
16.379999	733.666992	16.386139	0.664972	731.040894
16.400000	724.166992	16.406141	0.733131	729.955444
16.420000	744.000000	16.426142	0.811995	728.873169
16.440001	709.000000	16.446142	0.903896	727.794189
16.459999	712.666992	16.466141	1.011808	726.718262
16.480000	708.333008	16.486141	1.139636	725.645386
16.500000	715.500000	16.506142	1.292485	724.575684
16.520000	710.166992	16.526142	1.477207	723.509155
16.540001	735.666992	16.546143	1.703130	722.445679
16.559999	706.000000	16.566141	1.983181	721.385132
16.580000	730.166992	16.586142	2.335801	720.327881
16.600000	707.166992	16.606140	2.787712	719.273560
16.620001	716.833008	16.626141	3.379404	718.222412
16.640001	710.000000	16.646141	4.175786	717.174438
16.660000	709.666992	16.666140	5.296424	716.129517
16.680000	708.666992	16.686140	7.012664	715.087280
16.700001	711.833008	16.706141	10.018544	714.048340
16.720001	714.333008	16.726141	15.955708	713.012207
16.740002	726.333008	16.746141	27.867338	711.979126
16.760000	740.166992	16.766140	49.794464	710.949341
16.780001	771.333008	16.786140	86.267029	709.922363
16.799999	814.166992	16.806139	145.299530	708.898438
16.820000	845.666992	16.826139	218.793961	707.877197
16.840000	832.500000	16.846140	175.525146	706.859253
16.859999	756.666992	16.866138	67.640877	705.844238
16.879999	745.333008	16.886139	22.000549	704.831909
16.900000	728.000000	16.906137	11.293618	703.822876
16.920000	726.833008	16.926138	10.264072	702.816528
16.940001	732.333008	16.946140	9.758321	701.813110
16.959999	728.666992	16.966139	8.342519	700.812744
16.980000	726.333008	16.986139	6.674960	699.815186
17.000000	713.666992	17.006140	5.256223	698.820435
17.020000	721.833008	17.026140	4.182967	697.828613
17.040001	724.333008	17.046141	3.389808	696.839600

17.059999	708.833008	17.066139	2.796185	695.853882
17.080000	706.166992	17.086140	2.342359	694.870728
17.100000	707.333008	17.106140	1.988350	693.890381
17.120001	701.000000	17.126141	1.707246	692.912964
17.140001	694.333008	17.146141	1.480551	691.938110
17.160000	689.500000	17.166140	1.295253	690.966553
17.180000	678.000000	17.186140	1.141938	689.997681
17.200001	695.166992	17.206139	1.013755	689.031494
17.220001	687.166992	17.226139	0.905537	688.067993
17.240002	680.666992	17.246140	0.813398	687.107544
17.260000	677.833008	17.266138	0.734347	686.149780
17.280001	677.833008	17.286139	0.666032	685.194702
17.299999	665.000000	17.306137	0.606625	684.242554
17.320000	679.666992	17.326138	0.554651	683.293091
17.340000	705.166992	17.346138	0.508938	682.346191
17.359999	683.500000	17.366137	0.468533	681.402466
17.379999	675.000000	17.386137	0.432648	680.461304
17.400000	673.000000	17.406137	0.400644	679.522705
17.420000	670.500000	17.426138	0.371986	678.586792
17.440001	676.666992	17.446138	0.346230	677.653564
17.459999	673.666992	17.466139	0.323000	676.723145
17.480000	667.333008	17.486139	0.301981	675.795532
17.500000	673.000000	17.506138	0.282906	674.870483
17.520000	658.666992	17.526138	0.265541	673.948120
17.540001	653.333008	17.546139	0.249692	673.028564
17.559999	683.833008	17.566137	0.537827	672.111328
17.580000	673.000000	17.586138	0.531662	671.197021
17.600000	662.500000	17.606138	0.526859	670.285278
17.620001	678.333008	17.626139	0.523321	669.376221
17.640001	671.000000	17.646139	0.520972	668.469604
17.660000	647.500000	17.666138	0.519744	667.565796
17.680000	655.333008	17.686138	0.519583	666.664429
17.700001	660.500000	17.706139	0.520446	665.765869
17.720001	666.666992	17.726139	0.522299	664.869385
17.740002	674.833008	17.746140	0.525115	663.975952
17.760000	661.166992	17.766138	0.528876	663.084961
17.780001	670.833008	17.786139	0.533573	662.196899
17.799999	652.166992	17.806135	0.539200	661.311035

17.820000	636.333008	17.826136	0.545764	660.427734
17.840000	659.500000	17.846136	0.553273	659.546997
17.859999	650.166992	17.866135	0.561743	658.668701
17.879999	652.333008	17.886135	0.571200	657.792969
17.900000	663.000000	17.906136	0.581674	656.919800
17.920000	646.666992	17.926136	0.593201	656.049072
17.940001	642.166992	17.946136	0.605829	655.180664
17.959999	651.500000	17.966137	0.619611	654.315186
17.980000	647.833008	17.986137	0.634610	653.451782
18.000000	629.666992	18.006138	0.650899	652.591064
18.020000	647.833008	18.026138	0.668560	651.732666
18.040001	641.333008	18.046139	0.687691	650.876709
18.059999	635.000000	18.066137	0.708398	650.023438
18.080000	636.833008	18.086138	0.657030	649.172241
18.100000	661.500000	18.106136	0.683766	648.323730
18.120001	654.666992	18.126137	0.712391	647.477661
18.140001	634.666992	18.146137	0.743096	646.633911
18.160000	647.666992	18.166136	0.776095	645.792603
18.180000	649.833008	18.186136	0.811644	644.953613
18.200001	653.666992	18.206137	0.850026	644.117188
18.220001	647.500000	18.226137	0.891569	643.282715
18.240002	632.333008	18.246138	0.936651	642.450806
18.260000	635.000000	18.266136	0.985706	641.621582
18.280001	637.500000	18.286137	1.039256	640.794312
18.299999	639.500000	18.306135	1.097891	639.969482
18.320000	646.166992	18.326136	1.162328	639.147217
18.340000	635.000000	18.346136	1.233402	638.326904
18.359999	653.000000	18.366135	1.312108	637.509033
18.379999	644.000000	18.386135	1.399667	636.693481
18.400000	650.833008	18.406134	1.497525	635.880493
18.420000	637.500000	18.426134	1.607492	635.069580
18.440001	632.666992	18.446135	1.731771	634.260742
18.459999	636.500000	18.466135	1.873124	633.454346
18.480000	639.000000	18.486135	2.035038	632.650146
18.500000	629.500000	18.506136	2.221977	631.848389
18.520000	616.500000	18.526136	2.439728	631.048706
18.540001	635.333008	18.546137	2.695925	630.251343
18.559999	631.333008	18.566135	3.000791	629.456299

18.580000	626.666992	18.586136	3.368455	628.663330
18.600000	622.500000	18.606136	3.818854	627.872559
18.620001	633.833008	18.626137	4.382362	627.083984
18.640001	646.000000	18.646137	5.113228	626.297607
18.660000	614.166992	18.666136	6.132415	625.513672
18.680000	629.166992	18.686136	7.738413	624.731567
18.700001	623.166992	18.706135	10.580327	623.951904
18.720001	625.833008	18.726135	15.696521	623.174316
18.740002	630.000000	18.746136	24.073130	622.398804
18.760000	645.666992	18.766134	36.104923	621.625610
18.780001	662.333008	18.786135	52.963581	620.854492
18.799999	677.666992	18.806133	80.013451	620.085449
18.820000	702.166992	18.826134	97.633583	619.318481
18.840000	693.500000	18.846134	52.782295	618.553711
18.859999	638.666992	18.866133	20.982771	617.791138
18.879999	642.000000	18.886133	9.833327	617.030396
18.900000	631.500000	18.906134	7.513769	616.271851
18.920000	619.333008	18.926134	7.627564	615.515503
18.940001	610.500000	18.946136	7.527985	614.761108
18.959999	616.500000	18.966135	6.840406	614.008667
18.980000	601.333008	18.986135	5.929321	613.258423
19.000000	610.166992	19.006134	5.080970	612.510132
19.020000	630.333008	19.026134	4.381878	611.764160
19.040001	610.333008	19.046135	3.822307	611.019897
19.059999	598.666992	19.066133	3.371759	610.277954
19.080000	606.500000	19.086134	3.003556	609.537720
19.100000	596.166992	19.106134	2.698212	608.799561
19.120001	599.166992	19.126135	2.441660	608.063477
19.140001	606.500000	19.146135	2.223628	607.329468
19.160000	607.000000	19.166134	2.036479	606.597412
19.180000	606.500000	19.186134	1.874377	605.867065
19.200001	597.666992	19.206135	1.732869	605.138794
19.220001	608.333008	19.226135	1.608461	604.412720
19.240002	600.500000	19.246136	1.498385	603.688354
19.260000	605.333008	19.266134	1.400434	602.965942
19.280001	617.833008	19.286135	1.312796	602.245605
19.299999	612.666992	19.306131	1.234030	601.526978
19.320000	595.333008	19.326132	1.162896	600.810425

19.340000	586.333008	19.346132	1.098406	600.095825
19.359999	601.500000	19.366131	1.039726	599.383057
19.379999	601.833008	19.386131	0.986136	598.672119
19.400000	602.500000	19.406132	0.937041	597.963135
19.420000	601.000000	19.426134	0.891923	597.255859
19.440001	604.666992	19.446135	0.850353	596.550537
19.459999	604.500000	19.466133	0.811950	595.847046
19.480000	599.333008	19.486134	0.776379	595.145386
19.500000	582.500000	19.506134	0.743357	594.445801
19.520000	603.166992	19.526134	0.712635	593.747803
19.540001	581.500000	19.546135	0.683993	593.051636
19.559999	608.666992	19.566133	0.657242	592.357422
19.580000	598.500000	19.586134	0.632206	591.664917
19.600000	595.166992	19.606133	0.608739	590.974243
19.620001	585.833008	19.626133	0.586701	590.285400
19.640001	575.166992	19.646133	0.565975	589.598267
19.660000	588.000000	19.666132	0.546456	588.912964
19.680000	600.500000	19.686132	0.528043	588.229492
19.700001	586.833008	19.706133	0.510653	587.547485
19.720001	590.833008	19.726133	0.494207	586.867432
19.740002	591.166992	19.746134	0.478634	586.189087
19.760000	591.333008	19.766132	0.463872	585.512573
19.780001	588.000000	19.786133	0.449860	584.837524
19.799999	583.000000	19.806131	0.436549	584.164551
19.820000	565.333008	19.826132	0.423887	583.493164
19.840000	593.166992	19.846132	0.411832	582.823242
19.859999	595.500000	19.866133	0.400344	582.155273
19.879999	580.666992	19.886133	0.389386	581.488892
19.900000	581.333008	19.906132	0.378925	580.824097
19.920000	587.666992	19.926132	0.368929	580.161133
19.940001	600.333008	19.946133	0.359369	579.499756
19.959999	595.166992	19.966131	0.350220	578.840210
19.980000	583.833008	19.986132	0.341457	578.182007
20.000000	574.000000	20.006132	0.333056	577.525635
20.020000	591.666992	20.026133	0.324999	576.870728
20.040001	591.666992	20.046133	0.317264	576.217651
20.059999	568.500000	20.066132	0.309836	575.566040
20.080000	578.166992	20.086132	0.302695	574.916016

20.100000	579.666992	20.106133	0.000000	574.267700
20.120001	583.500000	20.126133	0.000000	573.620972
20.140001	597.000000	20.146133	0.000000	572.975708
20.160000	587.166992	20.166132	0.000000	572.332153
20.180000	583.833008	20.186132	0.000000	571.690063
20.200001	591.166992	20.206131	0.000000	571.049561
20.220001	589.333008	20.226131	0.000000	570.410522
20.240002	580.833008	20.246132	0.000000	569.773193
20.260000	564.666992	20.266130	0.000000	569.137207
20.280001	549.166992	20.286131	0.000000	568.503052
20.299999	572.500000	20.306129	0.000000	567.870239
20.320000	559.833008	20.326132	0.000000	567.238770
20.340000	576.000000	20.346132	0.980942	566.609009
20.359999	567.666992	20.366131	1.000438	565.980591
20.379999	584.333008	20.386131	1.020611	565.353882
20.400000	565.500000	20.406132	1.041493	564.728394
20.420000	570.833008	20.426132	1.063120	564.104370
20.440001	569.333008	20.446133	1.085530	563.482178
20.459999	550.833008	20.466131	1.108761	562.861206
20.480000	562.333008	20.486132	1.132861	562.241577
20.500000	573.500000	20.506130	1.157873	561.623413
20.520000	576.000000	20.526131	1.183852	561.006836
20.540001	568.500000	20.546131	1.210847	560.391602
20.559999	571.333008	20.566130	1.238915	559.777832
20.580000	562.833008	20.586130	1.268121	559.165283
20.600000	571.166992	20.606131	1.298531	558.554321
20.620001	560.333008	20.626131	1.330215	557.944702
20.640001	547.166992	20.646132	1.363249	557.336548
20.660000	548.500000	20.666130	1.397715	556.729614
20.680000	546.666992	20.686131	1.433709	556.124268
20.700001	555.333008	20.706131	1.471324	555.520020
20.720001	558.833008	20.726131	1.510666	554.917236
20.740002	554.166992	20.746132	1.551850	554.315796
20.760000	570.833008	20.766132	1.595001	553.715698
20.780001	565.000000	20.786133	1.640254	553.116943
20.799999	553.833008	20.806129	1.687748	552.519531
20.820000	538.500000	20.826130	1.737664	551.923706
20.840000	550.833008	20.846130	1.790169	551.328735

20.859999	537.666992	20.866129	1.845454	550.735229
20.879999	553.333008	20.886129	1.903742	550.143066
20.900000	546.000000	20.906130	1.965265	549.552124
20.920000	548.166992	20.926130	2.030282	548.962524
20.940001	576.000000	20.946131	2.099083	548.374146
20.959999	551.666992	20.966129	2.171980	547.786987
20.980000	574.333008	20.986130	2.249341	547.201294
21.000000	559.500000	21.006130	2.331558	546.616699
21.020000	554.833008	21.026131	2.419072	546.033203
21.040001	534.666992	21.046131	2.512382	545.451050
21.059999	542.333008	21.066130	2.612037	544.870239
21.080000	548.166992	21.086130	2.718687	544.290405
21.100000	551.833008	21.106131	2.833038	543.712036
21.120001	560.500000	21.126129	2.955890	543.135010
21.140001	539.500000	21.146130	3.088193	542.558838
21.160000	560.166992	21.166128	3.230983	541.984009
21.180000	542.666992	21.186131	3.385525	541.410278
21.200001	563.833008	21.206131	3.553187	540.837769
21.220001	555.333008	21.226131	3.735624	540.266479
21.240002	528.166992	21.246132	3.934749	539.696167
21.260000	548.000000	21.266130	4.152790	539.127197
21.280001	553.166992	21.286131	4.392444	538.559448
21.299999	560.333008	21.306129	4.656823	537.992798
21.320000	540.000000	21.326130	4.949759	537.427002
21.340000	563.666992	21.346130	5.275795	536.862671
21.359999	548.000000	21.366129	5.640449	536.299438
21.379999	532.333008	21.386129	6.050614	535.737061
21.400000	534.333008	21.406130	6.514725	535.176025
21.420000	550.500000	21.426128	7.043347	534.615967
21.440001	545.833008	21.446129	7.650067	534.057007
21.459999	544.500000	21.466127	8.352127	533.499146
21.480000	549.166992	21.486128	9.172355	532.942505
21.500000	539.166992	21.506128	10.140882	532.386719
21.520000	541.166992	21.526129	11.516895	531.832031
21.540001	551.333008	21.546129	12.927169	531.278442
21.559999	534.000000	21.566128	14.673192	530.726074
21.580000	547.000000	21.586128	16.914196	530.174316
21.600000	526.166992	21.606131	19.995350	529.623901

21.620001	541.500000	21.626131	24.709633	529.074585
21.640001	549.166992	21.646132	32.657467	528.526001
21.660000	539.833008	21.666130	46.253208	527.978638
21.680000	553.666992	21.686131	67.790504	527.432251
21.700001	606.333008	21.706131	98.421242	526.886841
21.720001	652.666992	21.726130	140.956894	526.342407
21.740002	741.833008	21.746130	208.681015	525.799072
21.760000	819.000000	21.766129	288.308319	525.256592
21.780001	774.833008	21.786129	202.922974	524.715210
21.799999	662.833008	21.806128	91.528069	524.174805
21.820000	593.666992	21.826128	44.008747	523.635132
21.840000	571.333008	21.846128	28.769371	523.096558
21.859999	538.166992	21.866127	25.810680	522.559082
21.879999	556.833008	21.886127	25.168245	522.022217
21.900000	538.333008	21.906128	23.528448	521.486450
21.920000	541.500000	21.926128	20.972546	520.951660
21.940001	523.333008	21.946129	18.298820	520.417725
21.959999	528.333008	21.966127	15.957719	519.884766
21.980000	525.000000	21.986128	14.037979	519.352661
22.000000	532.000000	22.006128	12.482491	518.821289
22.020000	541.666992	22.026131	11.211513	518.291016
22.040001	519.333008	22.046129	10.159912	517.761597
22.059999	532.833008	22.066128	9.278961	517.233154
22.080000	523.666992	22.086128	8.533044	516.705444
22.100000	504.500000	22.106129	7.895795	516.178589
22.120001	539.666992	22.126129	7.347208	515.652710
22.140001	532.666992	22.146130	6.871911	515.127686
22.160000	521.333008	22.166128	6.457978	514.603271
22.180000	541.666992	22.186129	6.095915	514.080078
22.200001	537.333008	22.206129	5.778281	513.557373
22.220001	548.000000	22.226130	5.499085	513.035767
22.240002	549.666992	22.246130	5.253511	512.514771
22.260000	545.833008	22.266129	5.037696	511.994659
22.280001	518.333008	22.286129	4.848472	511.475433
22.299999	515.833008	22.306128	4.683377	510.957062
22.320000	516.833008	22.326128	4.540415	510.439240
22.340000	530.500000	22.346127	4.418108	509.922455
22.359999	517.500000	22.366125	4.315351	509.406403

22.379999	513.500000	22.386126	4.231437	508.891083
22.400000	494.166992	22.406126	4.166049	508.376434
22.420000	514.333008	22.426128	4.119248	507.862701
22.440001	525.000000	22.446129	4.091520	507.349518
22.459999	513.333008	22.466127	4.083806	506.837311
22.480000	496.500000	22.486128	4.097601	506.325897
22.500000	512.333008	22.506128	4.135082	505.815033
22.520000	516.666992	22.526129	4.199280	505.305023
22.540001	510.332977	22.546129	4.294351	504.795624
22.559999	515.500000	22.566128	4.425952	504.287079
22.580000	492.332977	22.586128	4.601877	503.779205
22.600000	499.666992	22.606129	4.832892	503.272064
22.620001	520.166992	22.626129	5.134337	502.765594
22.640001	505.832977	22.646128	5.529339	502.259857
22.660000	508.332977	22.666126	6.057693	501.754974
22.680000	535.666992	22.686127	6.800825	501.250580
22.700001	545.166992	22.706127	7.941273	500.746857
22.720001	565.166992	22.726128	9.859491	500.243744
22.740002	559.833008	22.746128	13.191040	499.741486
22.760000	554.833008	22.766127	18.689774	499.239899
22.780001	562.166992	22.786127	26.966223	498.738922
22.799999	583.166992	22.806126	38.858902	498.238495
22.820000	654.500000	22.826128	57.413979	497.738739
22.840000	707.666992	22.846128	84.222183	497.239655
22.859999	712.666992	22.866127	77.013847	496.741302
22.879999	618.000000	22.886127	38.526546	496.243561
22.900000	565.833008	22.906128	18.360262	495.746368
22.920000	556.000000	22.926128	10.975231	495.249847
22.940001	551.666992	22.946129	8.887650	494.753937
22.959999	529.000000	22.966125	8.315009	494.258698
22.980000	518.500000	22.986126	7.761419	493.763885
23.000000	501.832977	23.006126	6.987537	493.269806
23.020000	507.500000	23.026127	6.158963	492.776337
23.040001	495.000000	23.046127	5.417322	492.283295
23.059999	503.832977	23.066126	4.804171	491.791046
23.080000	477.666992	23.086126	4.307317	491.299347
23.100000	482.666992	23.106127	3.902240	490.808014
23.120001	503.666992	23.126127	3.567317	490.317474

23.140001	495.000000	23.146128	3.286350	489.827362
23.160000	494.832977	23.166126	3.047557	489.337860
23.180000	488.166992	23.186127	2.842211	488.848907
23.200001	492.666992	23.206127	1.691250	488.360443
23.220001	488.832977	23.226130	1.553464	487.872650
23.240002	495.332977	23.246130	1.433279	487.385162
23.260000	504.500000	23.266127	1.327724	486.898468
23.280001	500.666992	23.286127	1.234398	486.412201
23.299999	499.832977	23.306126	1.151436	485.926483
23.320000	488.000000	23.326126	1.077289	485.441254
23.340000	497.666992	23.346127	1.010709	484.956451
23.359999	505.166992	23.366125	0.950668	484.472382
23.379999	494.666992	23.386126	0.896294	483.988495
23.400000	520.500000	23.406126	0.846872	483.505280
23.420000	493.832977	23.426126	0.801797	483.022614
23.440001	484.832977	23.446127	0.760552	482.540192
23.459999	479.332977	23.466125	0.722703	482.058502
23.480000	486.666992	23.486126	0.687865	481.577240
23.500000	488.666992	23.506126	0.655718	481.096466
23.520000	484.332977	23.526127	0.625980	480.615936
23.540001	495.000000	23.546127	0.598406	480.136017
23.559999	472.000000	23.566126	0.572787	479.656647
23.580000	485.166992	23.586124	0.548930	479.177582
23.600000	489.500000	23.606127	0.526669	478.699249
23.620001	498.666992	23.626127	0.505863	478.221039
23.640001	483.332977	23.646128	0.486381	477.743378
23.660000	478.500000	23.666126	0.468111	477.266083
23.680000	470.000000	23.686127	0.450947	476.789276
23.700001	477.000000	23.706127	0.434798	476.312958
23.720001	481.832977	23.726128	0.419584	475.836884
23.740002	466.332977	23.746128	0.405230	475.361420
23.760000	475.000000	23.766127	0.391671	474.886383
23.780001	477.332977	23.786127	0.378845	474.411591
23.799999	474.332977	23.806126	0.366700	473.937286
23.820000	484.332977	23.826126	0.692729	473.463226
23.840000	488.000000	23.846127	0.688974	472.989716
23.859999	475.666992	23.866125	0.685990	472.516632
23.879999	457.666992	23.886124	0.683751	472.043732

23.900000	473.166992	23.906124	0.682231	471.571442
23.920000	492.832977	23.926125	0.681410	471.099518
23.940001	462.500000	23.946125	0.681272	470.627716
23.959999	479.000000	23.966124	0.681800	470.156586
23.980000	481.000000	23.986126	0.729990	469.685455
24.000000	466.666992	24.006126	0.733228	469.214935
24.020000	479.000000	24.026127	0.737170	468.744843
24.040001	486.832977	24.046127	0.741816	468.274872
24.059999	480.500000	24.066126	0.747167	467.805450
24.080000	509.500000	24.086126	0.753228	467.336273
24.100000	508.666992	24.106127	0.760007	466.867401
24.120001	493.666992	24.126127	0.767512	466.398956
24.140001	484.332977	24.146128	0.775757	465.930695
24.160000	465.666992	24.166126	0.784755	465.462921
24.180000	466.000000	24.186127	0.794525	464.995453
24.200001	484.166992	24.206125	0.590986	464.528168
24.220001	467.666992	24.226126	0.607129	464.061432
24.240002	467.332977	24.246126	0.623939	463.594757
24.260000	468.000000	24.266125	0.641453	463.128632
24.280001	463.666992	24.286125	0.659713	462.662567
24.299999	495.166992	24.306124	0.678761	462.196930
24.320000	479.000000	24.326124	0.698646	461.731537
24.340000	453.166992	24.346127	0.719418	461.266327
24.359999	473.832977	24.366125	0.741126	460.801666
24.379999	472.832977	24.386126	0.763835	460.337189
24.400000	462.832977	24.406126	0.787605	459.872955
24.420000	465.832977	24.426126	0.812505	459.408905
24.440001	477.666992	24.446127	0.838610	458.945160
24.459999	466.666992	24.466125	0.865996	458.481842
24.480000	473.832977	24.486126	0.894759	458.018768
24.500000	467.666992	24.506126	0.924990	457.555634
24.520000	455.500000	24.526125	0.956791	457.093231
24.540001	458.332977	24.546125	0.990282	456.630951
24.559999	457.832977	24.566124	1.025583	456.168732
24.580000	463.000000	24.586124	1.062839	455.706879
24.600000	449.666992	24.606125	1.102197	455.245331
24.620001	447.666992	24.626125	1.143826	454.783844
24.640001	455.332977	24.646126	1.187912	454.322723

24.660000	459.000000	24.666124	1.234656	453.861847
24.680000	455.666992	24.686125	1.284299	453.401154
24.700001	451.666992	24.706125	1.337092	452.940704
24.720001	462.000000	24.726128	1.393332	452.480438
24.740002	450.000000	24.746128	1.453331	452.020416
24.760000	449.666992	24.766127	1.517458	451.560638
24.780001	451.000000	24.786127	1.586143	451.101044
24.799999	445.666992	24.806126	1.659850	450.641754
24.820000	443.832977	24.826124	1.739135	450.182526
24.840000	441.666992	24.846125	1.824638	449.723663
24.859999	445.000000	24.866123	1.917081	449.264923
24.879999	438.832977	24.886124	2.017342	448.806488
24.900000	442.832977	24.906124	2.126429	448.348236
24.920000	449.500000	24.926125	2.245553	447.889862
24.940001	441.000000	24.946125	2.376165	447.431976
24.959999	439.832977	24.966124	2.520015	446.974274
24.980000	431.000000	24.986124	2.679292	446.516693
25.000000	443.000000	25.006124	2.856677	446.059357
25.020000	430.832977	25.026125	3.055562	445.602081
25.040001	436.500000	25.046125	3.280302	445.145111
25.059999	447.166992	25.066124	3.536548	444.688263
25.080000	447.666992	25.086126	3.831949	444.231476
25.100000	437.666992	25.106127	4.176756	443.774994
25.120001	446.000000	25.126127	4.585599	443.318634
25.140001	431.832977	25.146126	5.080330	442.862518
25.160000	440.500000	25.166124	5.697562	442.406525
25.180000	463.000000	25.186125	6.507624	441.950653
25.200001	441.332977	25.206125	7.657747	441.494965
25.220001	443.166992	25.226126	9.446887	441.039398
25.240002	434.332977	25.246126	12.402469	440.583954
25.260000	453.000000	25.266125	17.290094	440.128693
25.280001	453.166992	25.286125	25.092842	439.673676
25.299999	459.332977	25.306124	37.354805	439.218658
25.320000	485.500000	25.326124	57.150784	438.763824
25.340000	518.000000	25.346125	83.429962	438.309235
25.359999	525.833008	25.366123	81.547523	437.854767
25.379999	488.832977	25.386124	47.631001	437.400177
25.400000	455.166992	25.406124	26.411938	436.946014

25.420000	444.332977	25.426125	18.486786	436.491852
25.440001	454.332977	25.446127	16.404335	436.037811
25.459999	433.500000	25.466124	16.342405	435.583893
25.480000	438.666992	25.486124	16.860561	435.130219
25.500000	436.500000	25.506124	17.652864	434.676666
25.520000	440.166992	25.526125	18.798668	434.222992
25.540001	438.832977	25.546125	20.428999	433.769623
25.559999	427.666992	25.566124	22.657795	433.316376
25.580000	431.000000	25.586124	25.612888	432.863068
25.600000	434.666992	25.606125	29.477699	432.410126
25.620001	419.000000	25.626125	34.536644	431.957123
25.640001	439.500000	25.646126	41.248692	431.504303
25.660000	444.332977	25.666124	50.421494	431.051605
25.680000	454.500000	25.686125	63.661156	430.598846
25.700001	452.832977	25.706125	84.368240	430.146332
25.720001	470.332977	25.726126	119.450218	429.693878
25.740002	543.166992	25.746126	181.142822	429.241486
25.760000	624.166992	25.766123	287.613373	428.789337
25.780001	780.166992	25.786125	463.608490	428.337189
25.799999	1080.830078	25.806124	749.407471	427.885162
25.820000	1591.669922	25.826124	1217.876953	427.433136
25.840000	2043.169922	25.846125	1801.127441	426.981293
25.859999	1906.669922	25.866123	1672.875732	426.529510
25.879999	1333.169922	25.886124	887.210144	426.077667
25.900000	817.333008	25.906124	402.818939	425.626129
25.920000	556.333008	25.926125	209.760345	425.174591
25.940001	486.166992	25.946125	141.483200	424.723175
25.959999	457.166992	25.966124	113.767365	424.271820
25.980000	437.666992	25.986124	95.197983	423.820343
26.000000	436.666992	26.006124	78.374779	423.369293
26.020000	433.332977	26.026125	63.486588	422.917999
26.040001	435.832977	26.046125	51.351715	422.467010
26.059999	425.500000	26.066124	41.941090	422.016083
26.080000	416.500000	26.086123	34.739857	421.565094
26.100000	419.500000	26.106123	29.186600	421.114105
26.120001	414.332977	26.126123	24.838013	420.663361
26.140001	419.166992	26.146126	21.376143	420.212616
26.160000	413.666992	26.166124	18.579069	419.761932

26.180000	423.832977	26.186125	16.287992	419.311432
26.200001	418.666992	26.206125	14.389179	418.860687
26.220001	426.832977	26.226126	12.798778	418.410309
26.240002	429.500000	26.246126	11.454048	417.959808
26.260000	432.832977	26.266125	10.307433	417.509430
26.280001	417.166992	26.286125	9.322005	417.059174
26.299999	413.666992	26.306124	8.469328	416.609039
26.320000	424.000000	26.326124	7.726652	416.158783
26.340000	416.000000	26.346125	7.076059	415.708588
26.359999	411.832977	26.366123	6.503109	415.258514
26.379999	410.332977	26.386124	5.995934	414.808502
26.400000	409.500000	26.406122	5.544999	414.358490
26.420000	408.666992	26.426123	5.142282	413.908722
26.440001	410.166992	26.446123	4.781234	413.458771
26.459999	423.666992	26.466122	4.456374	413.008881
26.480000	420.000000	26.486124	4.162980	412.559174
26.500000	418.500000	26.506124	3.929788	412.109283
26.520000	411.832977	26.526125	3.689422	411.659698
26.540001	404.332977	26.546125	3.470537	411.209991
26.559999	408.666992	26.566124	3.270715	410.760406
26.580000	410.832977	26.586124	3.087813	410.310883
26.600000	420.332977	26.606125	2.920032	409.861359
26.620001	399.500000	26.626125	2.765791	409.411835
26.640001	411.000000	26.646126	2.623712	408.962433
26.660000	411.500000	26.666124	2.492600	408.513031
26.680000	395.000000	26.686125	2.371373	408.063690
26.700001	385.500000	26.706125	2.259111	407.614410
26.720001	414.666992	26.726124	2.155002	407.165070
26.740002	401.000000	26.746124	2.012183	406.715851
26.760000	404.832977	26.766123	1.923566	406.266693
26.780001	418.832977	26.786123	1.841077	405.817596
26.799999	396.666992	26.806122	1.764227	405.368439
26.820000	395.500000	26.826124	1.692539	404.919281
26.840000	399.000000	26.846125	1.625629	404.470245
26.859999	399.832977	26.866123	1.563132	404.021149
26.879999	410.332977	26.886124	1.504710	403.572296
26.900000	392.832977	26.906124	1.450078	403.123260
26.920000	389.332977	26.926125	1.398975	402.674347

26.940001	384.000000	26.946125	1.351165	402.225555
26.959999	392.000000	26.966124	1.306444	401.776642
26.980000	398.332977	26.986124	1.264614	401.327911
27.000000	386.166992	27.006124	1.225512	400.879120
27.020000	396.500000	27.026125	1.188990	400.430206
27.040001	399.832977	27.046124	1.154918	399.981598
27.059999	403.666992	27.066122	1.123176	399.533051
27.080000	396.000000	27.086123	1.093663	399.084320
27.100000	392.666992	27.106123	1.066293	398.635712
27.120001	389.500000	27.126123	1.040996	398.187103
27.140001	401.666992	27.146126	1.017709	397.738495
27.160000	402.166992	27.166124	0.996394	397.290009
27.180000	397.500000	27.186125	0.977012	396.841522
27.200001	394.166992	27.206125	0.981821	396.393097
27.220001	393.666992	27.226126	0.967032	395.944672
27.240002	390.666992	27.246126	0.954202	395.496246
27.260000	391.500000	27.266125	0.943362	395.047821
27.280001	383.332977	27.286125	0.934554	394.599579
27.299999	387.332977	27.306124	0.927847	394.151337
27.320000	399.666992	27.326124	0.923328	393.702972
27.340000	399.666992	27.346125	0.921108	393.254791
27.359999	402.832977	27.366121	0.921328	392.806610
27.379999	393.500000	27.386122	0.924159	392.358368
27.400000	398.000000	27.406122	0.929811	391.910309
27.420000	393.332977	27.426123	0.938540	391.462189
27.440001	403.500000	27.446123	0.950651	391.014069
27.459999	405.832977	27.466122	0.966513	390.566071
27.480000	397.166992	27.486124	0.990231	390.118073
27.500000	392.666992	27.506124	1.015182	389.670013
27.520000	380.500000	27.526125	1.045532	389.222137
27.540001	389.500000	27.546125	1.082075	388.774139
27.559999	388.832977	27.566124	1.125776	388.326324
27.580000	396.832977	27.586124	1.177830	387.878510
27.600000	391.500000	27.606125	1.239703	387.430573
27.620001	374.666992	27.626125	1.313219	386.982941
27.640001	386.832977	27.646126	1.400664	386.535187
27.660000	405.332977	27.666124	1.504913	386.087555
27.680000	395.666992	27.686123	1.629656	385.639801

27.700001	395.000000	27.706123	1.779648	385.192291
27.720001	422.166992	27.726124	1.961057	384.744659
27.740002	449.500000	27.746124	2.182038	384.297028
27.760000	485.000000	27.766123	2.453465	383.849579
27.780001	501.332977	27.786123	2.790199	383.402130
27.799999	473.500000	27.806124	3.212675	382.954681
27.820000	459.832977	27.826124	3.749699	382.507294
27.840000	423.332977	27.846125	4.442741	382.059906
27.859999	410.166992	27.866123	5.353138	381.612640
27.879999	393.500000	27.886124	6.576123	381.165375
27.900000	389.832977	27.906124	8.269670	380.718170
27.920000	377.332977	27.926125	10.399626	380.271027
27.940001	382.000000	27.946125	14.212809	379.823883
27.959999	389.832977	27.966124	20.608543	379.376740
27.980000	394.832977	27.986124	32.036308	378.929718
28.000000	413.500000	28.006123	52.891403	378.482758
28.020000	423.832977	28.026123	90.378822	378.035858
28.040001	493.000000	28.046124	156.175385	377.588898
28.059999	609.833008	28.066122	269.505157	377.142181
28.080000	760.166992	28.086123	442.489716	376.695343
28.100000	846.666992	28.106123	560.179871	376.248566
28.120001	737.500000	28.126125	414.828552	375.801727
28.140001	568.000000	28.146126	204.092758	375.355133
28.160000	443.000000	28.166124	93.598175	374.908600
28.180000	398.332977	28.186125	50.100662	374.462067
28.200001	379.166992	28.206125	33.244804	374.015472
28.220001	367.332977	28.226126	25.005697	373.569122
28.240002	382.666992	28.246126	19.393885	373.122711
28.260000	375.332977	28.266125	14.996397	372.676483
28.280001	373.832977	28.286125	11.594501	372.230255
28.299999	376.666992	28.306124	9.069054	371.784027
28.320000	371.332977	28.326122	7.236957	371.337921
28.340000	373.832977	28.346123	5.911692	370.891876
28.359999	373.500000	28.366121	4.946265	370.445953
28.379999	354.000000	28.386122	4.237257	369.999908
28.400000	364.166992	28.406122	3.715453	369.554108
28.420000	369.500000	28.426123	3.334659	369.108246
28.440001	365.832977	28.446125	3.064045	368.662445

28.459999	352.500000	28.466124	2.883325	368.216827
28.480000	370.500000	28.486124	2.779519	367.771271
28.500000	364.832977	28.506124	2.745371	367.325653
28.520000	362.500000	28.526125	2.778260	366.880280
28.540001	374.166992	28.546125	2.879854	366.434784
28.559999	375.666992	28.566124	3.056240	365.989532
28.580000	356.166992	28.586124	3.318704	365.544281
28.600000	374.000000	28.606125	3.685008	365.099152
28.620001	368.332977	28.626125	4.181806	364.654083
28.640001	350.832977	28.646124	4.848377	364.209198
28.660000	364.166992	28.666122	5.743093	363.764252
28.680000	373.166992	28.686123	6.954077	363.319366
28.700001	362.332977	28.706123	8.618629	362.874664
28.720001	368.166992	28.726124	10.964385	362.429962
28.740002	367.000000	28.746124	14.404263	361.985443
28.760000	363.332977	28.766125	19.761665	361.540924
28.780001	381.332977	28.786125	28.758757	361.096466
28.799999	386.332977	28.806124	44.905575	360.652252
28.820000	394.500000	28.826124	74.825119	360.208038
28.840000	449.332977	28.846125	129.959290	359.763824
28.859999	535.333008	28.866123	229.394760	359.319916
28.879999	708.500000	28.886124	404.587463	358.875946
28.900000	985.833008	28.906124	684.571899	358.432098
28.920000	1182.669922	28.926125	941.334595	357.988434
28.940001	1072.169922	28.946125	798.819824	357.544708
28.959999	752.166992	28.966124	425.091797	357.101288
28.980000	515.166992	28.986122	193.167984	356.657867
29.000000	413.666992	29.006123	96.534836	356.214569
29.020000	378.166992	29.026123	59.036945	355.771271
29.040001	373.500000	29.046124	41.936684	355.328278
29.059999	356.832977	29.066122	31.391016	354.885345
29.080000	373.166992	29.086124	23.588652	354.442413
29.100000	352.666992	29.106125	17.691694	353.999664
29.120001	359.832977	29.126125	13.360308	353.556915
29.140001	350.332977	29.146126	10.241827	353.114410
29.160000	356.666992	29.166124	7.997169	352.672150
29.180000	348.332977	29.186125	6.359266	352.229767
29.200001	360.832977	29.206125	5.141450	351.787628

29.220001	364.500000	29.226126	4.218323	351.345551
29.240002	342.166992	29.246126	3.506012	350.903595
29.260000	348.332977	29.266125	2.947602	350.461884
29.280001	348.500000	29.286125	2.503502	350.020233
29.299999	350.500000	29.306122	2.145900	349.578827
29.320000	357.500000	29.326122	1.854537	349.137360
29.340000	359.832977	29.346123	1.614733	348.696075
29.359999	334.500000	29.366121	1.415529	348.255096
29.379999	353.666992	29.386124	1.248588	347.813934
29.400000	349.332977	29.406124	1.107632	347.373077
29.420000	353.166992	29.426125	0.987748	346.932343
29.440001	365.666992	29.446125	0.885114	346.491852
29.459999	341.332977	29.466124	0.796718	346.051483
29.480000	352.000000	29.486124	0.720141	345.611176
29.500000	353.166992	29.506124	0.653462	345.171051
29.520000	343.000000	29.526125	0.595119	344.731049
29.540001	355.166992	29.546125	0.543838	344.291229
29.559999	357.332977	29.566124	0.498578	343.851593
29.580000	367.332977	29.586124	0.458467	343.412079
29.600000	363.166992	29.606125	0.425561	342.972687
29.620001	360.166992	29.626123	0.393817	342.533600
29.640001	369.832977	29.646124	0.365405	342.094513
29.660000	382.166992	29.666122	0.339905	341.655731
29.680000	381.000000	29.686123	0.316951	341.216949
29.700001	375.332977	29.706125	0.298018	340.778351
29.720001	380.000000	29.726126	0.247407	340.340118
29.740002	360.332977	29.746126	0.231516	339.901886
29.760000	343.000000	29.766125	0.217139	339.463837
29.780001	350.332977	29.786125	0.204108	339.026031
29.799999	343.832977	29.806124	0.192280	338.588409
29.820000	342.332977	29.826124	0.181530	338.150909
29.840000	339.832977	29.846125	0.171748	337.713593
29.859999	328.500000	29.866123	0.162841	337.276520
29.879999	349.500000	29.886124	0.154723	336.839630
29.900000	346.832977	29.906124	0.147322	336.402863
29.920000	329.500000	29.926125	0.140573	335.966339
29.940001	333.166992	29.946125	0.134420	335.529938
29.959999	338.832977	29.966122	0.128813	335.093781

29.980000	335.000000	29.986122	0.123707	334.657867
30.000000	333.166992	30.006124	0.119063	334.221954
30.020000	331.832977	30.026125	0.114847	333.786469
30.040001	342.332977	30.046125	0.111030	333.351105
30.059999	338.166992	30.066124	0.107584	332.915863
30.080000	325.000000	30.086124	0.104485	332.480988
30.100000	343.332977	30.106125	0.101714	332.046234
30.120001	353.666992	30.126125	0.099252	331.611664
30.140001	342.666992	30.146126	0.097083	331.177338
30.160000	351.500000	30.166124	0.095196	330.743256
30.180000	343.166992	30.186125	0.093577	330.309296
30.200001	338.166992	30.206125	0.092219	329.875702
30.220001	331.332977	30.226126	0.091979	329.442108
30.240002	338.000000	30.246126	0.091145	329.008942
30.260000	362.666992	30.266125	0.090555	328.576019
30.280001	366.332977	30.286123	0.090205	328.143280
30.299999	357.000000	30.306124	0.090095	327.710663
30.320000	345.500000	30.326124	0.090224	327.278351
30.340000	335.166992	30.346125	0.090596	326.846283
30.359999	340.000000	30.366123	0.091213	326.414398
30.379999	336.666992	30.386124	0.092082	325.982819
30.400000	348.166992	30.406124	0.089715	325.551483
30.420000	327.166992	30.426125	0.091241	325.120331
30.440001	331.666992	30.446125	0.093037	324.689484
30.459999	328.832977	30.466124	0.095116	324.258820
30.480000	326.666992	30.486124	0.097493	323.828400
30.500000	324.666992	30.506124	0.100185	323.398346
30.520000	336.000000	30.526125	0.103212	322.968353
30.540001	334.332977	30.546125	0.106597	322.538849
30.559999	355.000000	30.566124	0.110366	322.109467
30.580000	356.332977	30.586124	0.114547	321.680328
30.600000	373.666992	30.606127	0.119175	321.251556
30.620001	353.666992	30.626125	0.124285	320.822906
30.640001	357.666992	30.646126	0.129921	320.394623
30.660000	362.666992	30.666124	0.113951	319.966583
30.680000	375.166992	30.686125	0.121513	319.538788
30.700001	400.166992	30.706125	0.129732	319.111298
30.720001	428.666992	30.726126	0.138678	318.684113

30.740002	442.832977	30.746126	0.148434	318.257111
30.760000	433.332977	30.766125	0.159088	317.830536
30.780001	407.166992	30.786125	0.170745	317.404205
30.799999	375.832977	30.806124	0.183522	316.978180
30.820000	351.666992	30.826124	0.197556	316.552277
30.840000	340.832977	30.846125	0.212999	316.126862
30.859999	332.500000	30.866123	0.230027	315.701691
30.879999	322.000000	30.886126	0.248848	315.276703
30.900000	342.666992	30.906126	0.269692	314.852020
30.920000	343.166992	30.926126	0.292835	314.427704
30.940001	366.332977	30.946125	0.318592	314.003754
30.959999	393.500000	30.966124	0.347333	313.580048
30.980000	430.166992	30.986124	0.379497	313.156647
31.000000	490.666992	31.006124	0.415592	312.733490
31.020000	513.500000	31.026125	0.456220	312.310822
31.040001	522.000000	31.046125	0.502097	311.888214
31.059999	494.666992	31.066124	0.554070	311.466156
31.080000	434.500000	31.086124	0.613168	311.044342
31.100000	398.332977	31.106125	0.680616	310.622711
31.120001	369.666992	31.126125	0.757900	310.201508
31.140001	341.500000	31.146126	0.846828	309.780609
31.160000	344.666992	31.166124	0.949604	309.360077
31.180000	340.832977	31.186127	1.068986	308.939850
31.200001	338.832977	31.206127	1.208326	308.519928
31.220001	334.666992	31.226128	1.371862	308.100372
31.240002	351.000000	31.246128	1.564917	307.681183
31.260000	343.666992	31.266127	1.794225	307.262299
31.280001	346.166992	31.286125	2.068459	306.843781
31.299999	339.666992	31.306124	2.398809	306.425629
31.320000	330.000000	31.326124	2.799941	306.007843
31.340000	323.832977	31.346125	3.291152	305.590240
31.359999	315.500000	31.366123	3.898219	305.173187
31.379999	322.332977	31.386124	4.656265	304.756378
31.400000	317.332977	31.406124	5.613348	304.339874
31.420000	312.000000	31.426125	6.836633	303.923859
31.440001	309.832977	31.446125	8.421490	303.508087
31.459999	317.666992	31.466125	10.505948	303.092682
31.480000	328.332977	31.486126	13.294045	302.677765

31.500000	327.500000	31.506126	17.094887	302.263031
31.520000	326.332977	31.526127	22.391262	301.848846
31.540001	322.332977	31.546127	29.969908	301.434906
31.559999	331.332977	31.566126	41.193481	301.021393
31.580000	346.166992	31.586126	58.628098	300.608307
31.600000	364.332977	31.606127	87.483841	300.195526
31.620001	375.000000	31.626125	138.788132	299.783112
31.640001	450.000000	31.646126	235.675171	299.371185
31.660000	563.833008	31.666124	424.352844	298.959534
31.680000	865.500000	31.686125	792.126648	298.548340
31.700001	1486.500000	31.706125	1494.342773	298.137451
31.720001	2792.500000	31.726126	2765.483154	297.726990
31.740002	4525.329590	31.746126	4677.359863	297.316895
31.760000	5566.829590	31.766127	6094.283691	296.907318
31.780001	4598.669922	31.786127	5036.406250	296.497955
31.799999	2676.500000	31.806126	2743.912598	296.089142
31.820000	1276.169922	31.826126	1258.487671	295.680725
31.840000	687.500000	31.846127	607.853455	295.272675
31.859999	482.166992	31.866125	344.083862	294.865021
31.879999	403.166992	31.886126	223.263794	294.457825
31.900000	363.166992	31.906126	155.247131	294.050995
31.920000	355.166992	31.926126	111.683960	293.644562
31.940001	343.666992	31.946125	83.477005	293.238678
31.959999	311.666992	31.966124	66.600342	292.833130
31.980000	328.832977	31.986124	58.840298	292.427948
32.000000	327.832977	32.006126	59.737770	292.023254
32.020000	323.666992	32.026127	71.563766	291.618866
32.040001	357.000000	32.046127	101.447098	291.215027
32.060001	395.332977	32.066128	165.455292	290.811554
32.080002	504.500000	32.086128	295.659882	290.408539
32.099998	689.333008	32.106125	551.328308	290.006012
32.119999	1109.500000	32.126122	1030.182007	289.603973
32.139999	1922.500000	32.146122	1820.041138	289.202271
32.160000	2635.830078	32.166122	2656.088867	288.801056
32.180000	2571.830078	32.186127	2605.799316	288.400177
32.200001	1693.669922	32.206127	1616.932861	287.999756
32.220001	884.833008	32.226128	768.816101	287.599915
32.240002	523.666992	32.246128	357.143860	287.200348

32.260002	406.166992	32.266129	188.632553	286.801392
32.280003	342.500000	32.286129	115.536583	286.402832
32.299999	335.500000	32.306126	77.114822	286.004883
32.320000	322.000000	32.326126	53.118690	285.607178
32.340000	327.000000	32.346127	37.056194	285.210022
32.360001	324.000000	32.366127	26.233337	284.813324
32.380001	322.832977	32.386127	18.987127	284.417084
32.400002	328.832977	32.406128	14.130240	284.021271
32.420002	349.500000	32.426128	10.843967	283.625977
32.440002	336.666992	32.446129	8.595279	283.231201
32.460003	339.000000	32.466129	7.048670	282.836823
32.480003	330.166992	32.486130	5.995669	282.442963
32.500000	318.500000	32.506126	5.308117	282.049591
32.520000	305.832977	32.526127	4.909630	281.656769
32.540001	299.000000	32.546127	4.760638	281.264221
32.560001	300.500000	32.566128	4.850114	280.872345
32.580002	290.666992	32.586128	5.193827	280.480865
32.599998	291.332977	32.606125	5.837535	280.090027
32.619999	290.832977	32.626125	6.867199	279.699524
32.639999	285.166992	32.646126	8.427535	279.309570
32.660000	306.666992	32.666126	10.758890	278.920044
32.680000	302.832977	32.686127	14.268944	278.531067
32.700001	300.166992	32.706127	19.685791	278.142548
32.720001	300.332977	32.726128	28.407583	277.754608
32.740002	297.500000	32.746132	43.316929	277.367035
32.760002	306.666992	32.766132	70.559975	276.980133
32.780003	363.500000	32.786129	123.164864	276.593689
32.799999	410.500000	32.806126	227.660522	276.207886
32.820000	601.500000	32.826126	435.231140	275.822479
32.840000	970.166992	32.846127	837.453552	275.437469
32.860001	1678.830078	32.866127	1564.698975	275.053131
32.880001	2610.000000	32.886127	2618.294434	274.669220
32.900002	3050.830078	32.906128	3316.307617	274.285858
32.920002	2557.000000	32.926128	2692.567383	273.903137
32.940002	1613.000000	32.946129	1470.499023	273.520721
32.960003	861.000000	32.966129	676.726257	273.138977
32.980003	531.500000	32.986130	323.459564	272.757690
33.000000	390.500000	33.006126	177.788956	272.377075

33.020000	346.666992	33.026127	110.490456	271.996887
33.040001	309.332977	33.046127	72.977448	271.617279
33.060001	308.832977	33.066128	49.232067	271.238129
33.080002	280.500000	33.086128	33.577477	270.859589
33.099998	285.000000	33.106125	23.237013	270.481628
33.119999	278.666992	33.126125	16.405001	270.104187
33.139999	279.666992	33.146126	11.848493	269.727173
33.160000	287.332977	33.166126	8.753603	269.350891
33.180000	278.166992	33.186127	6.604351	268.975067
33.200001	281.166992	33.206127	5.078036	268.599731
33.220001	274.000000	33.226128	3.971344	268.225006
33.240002	272.332977	33.246128	3.153744	267.850830
33.260002	272.000000	33.266129	2.539551	267.477203
33.280003	271.332977	33.286133	2.071181	267.104065
33.299999	268.166992	33.306129	1.709480	266.731628
33.320000	273.000000	33.326130	1.426735	266.359711
33.340000	270.832977	33.346130	1.203531	265.988342
33.360001	270.832977	33.366131	1.025842	265.617615
33.380001	270.832977	33.386131	0.883442	265.247284
33.400002	268.000000	33.406132	0.768803	264.877686
33.420002	282.500000	33.426132	0.676336	264.508606
33.440002	273.166992	33.446133	0.601881	264.140045
33.460003	273.166992	33.466129	0.542357	263.772186
33.480003	279.166992	33.486130	0.495478	263.404877
33.500000	276.832977	33.506126	0.459656	263.038147
33.520000	273.500000	33.526127	0.433820	262.671936
33.540001	269.000000	33.546127	0.417415	262.306366
33.560001	261.500000	33.566128	0.410351	261.941315
33.580002	271.666992	33.586128	0.413036	261.576843
33.599998	269.832977	33.606125	0.426448	261.213074
33.619999	269.666992	33.626125	0.452282	260.849792
33.639999	264.500000	33.646126	0.493156	260.487213
33.660000	265.332977	33.666126	0.552976	260.125061
33.680000	257.500000	33.686127	0.637485	259.763641
33.700001	265.832977	33.706127	0.755129	259.402740
33.720001	253.833008	33.726128	0.918448	259.042450
33.740002	268.000000	33.746128	1.146342	258.682800
33.760002	266.332977	33.766129	1.467860	258.323700

33.780003	263.166992	33.786129	1.928771	257.965271
33.799999	262.000000	33.806126	2.603390	257.607452
33.820000	259.166992	33.826130	3.618982	257.250153
33.840000	271.000000	33.846130	5.204264	256.893494
33.860001	276.666992	33.866131	7.806495	256.537445
33.880001	275.332977	33.886131	12.356722	256.182068
33.900002	260.666992	33.906132	20.860918	255.827209
33.920002	286.500000	33.926132	37.612759	255.473022
33.940002	311.332977	33.946133	71.470764	255.119446
33.960003	366.166992	33.966133	139.730209	254.766510
33.980003	511.000000	33.986134	273.133148	254.414215
34.000000	774.500000	34.006130	511.594879	254.062592
34.020000	1186.830078	34.026131	838.838013	253.711456
34.040001	1362.500000	34.046131	1021.431763	253.361023
34.060001	1134.330078	34.066132	805.208313	253.011139
34.080002	713.000000	34.086132	437.225647	252.661926
34.099998	437.832977	34.106129	201.910706	252.313446
34.119999	354.332977	34.126129	96.230240	251.965515
34.139999	327.332977	34.146130	52.014923	251.618225
34.160000	332.832977	34.166126	31.474134	251.271637
34.180000	325.832977	34.186127	20.196381	250.925537
34.200001	333.666992	34.206127	13.264947	250.580231
34.220001	325.500000	34.226128	8.830783	250.235443
34.240002	316.832977	34.246128	5.975716	249.891266
34.260002	303.666992	34.266129	4.130316	249.547882
34.280003	320.000000	34.286129	2.923108	249.204956
34.299999	355.666992	34.306126	2.117928	248.862915
34.320000	366.666992	34.326126	1.568189	248.521332
34.340000	381.666992	34.346127	1.184169	248.180450
34.360001	371.332977	34.366131	0.910022	247.840149
34.380001	353.000000	34.386131	0.710522	247.500549
34.400002	324.332977	34.406132	0.562731	247.161682
34.420002	286.832977	34.426132	0.451492	246.823273
34.440002	267.500000	34.446133	0.366554	246.485748
34.460003	277.000000	34.466133	0.300845	246.148712
34.480003	264.166992	34.486134	0.249404	245.812408
34.500000	260.166992	34.506130	0.208701	245.476776
34.520000	257.500000	34.526131	0.176159	245.141846

34.540001	259.666992	34.546131	0.149911	244.807526
34.560001	258.332977	34.566132	0.128564	244.473816
34.580002	267.000000	34.586132	0.111070	244.140747
34.599998	280.166992	34.606129	0.096638	243.808533
34.619999	280.000000	34.626129	0.084656	243.476868
34.639999	278.666992	34.646130	0.074658	243.145874
34.660000	263.332977	34.666130	0.066277	242.815460
34.680000	247.667007	34.686131	0.057531	242.485840
34.700001	246.667007	34.706131	0.051641	242.156830
34.720001	248.000000	34.726131	0.046681	241.828552
34.740002	241.000000	34.746132	0.040626	241.500885
34.760002	243.000000	34.766132	0.037183	241.173889
34.780003	239.333008	34.786133	0.034335	240.847565
34.799999	244.000000	34.806129	0.032020	240.522003
34.820000	235.167007	34.826130	0.030193	240.197083
34.840000	241.667007	34.846127	0.028825	239.872864
34.860001	228.000000	34.866127	0.027905	239.549255
34.880001	221.833008	34.886131	0.027436	239.226349
34.900002	240.500000	34.906132	0.027443	238.904114
34.920002	238.500000	34.926132	0.027968	238.582581
34.940002	236.500000	34.946133	0.029081	238.261719
34.960003	229.167007	34.966133	0.030884	237.941559
34.980003	237.833008	34.986134	0.033522	237.622101
35.000000	232.333008	35.006130	0.037200	237.303345
35.020000	244.000000	35.026131	0.042203	236.985260
35.040001	240.833008	35.046131	0.048931	236.667877
35.060001	253.333008	35.066132	0.057949	236.351105
35.080002	238.500000	35.086132	0.070067	236.035126
35.099998	255.667007	35.106129	0.086454	235.719818
35.119999	252.167007	35.126129	0.108845	235.405212
35.139999	246.000000	35.146130	0.139833	235.091309
35.160000	247.833008	35.166130	0.183403	234.778015
35.180000	236.167007	35.186131	0.245813	234.465454
35.200001	246.833008	35.206131	0.337191	234.153625
35.220001	233.500000	35.226131	0.474534	233.842468
35.240002	232.167007	35.246132	0.687824	233.532013
35.260002	241.167007	35.266132	1.033471	233.222290
35.280003	229.333008	35.286133	1.625332	232.913269

35.299999	223.000000	35.306129	2.706006	232.604858
35.320000	234.000000	35.326130	4.805676	232.297272
35.340000	243.667007	35.346130	9.065540	231.990387
35.360001	250.333008	35.366131	17.848795	231.684082
35.380001	269.332977	35.386131	35.729362	231.378540
35.400002	292.166992	35.406136	70.320023	231.073700
35.420002	346.332977	35.426136	128.478989	230.769562
35.440002	407.000000	35.446136	194.832733	230.466125
35.460003	435.332977	35.466137	209.463638	230.163452
35.480003	368.666992	35.486137	147.830246	229.861511
35.500000	297.000000	35.506134	76.289055	229.560272
35.520000	264.000000	35.526134	35.154354	229.259705
35.540001	250.333008	35.546131	17.022179	228.959900
35.560001	250.667007	35.566132	9.242817	228.660736
35.580002	254.833008	35.586132	5.508180	228.362305
35.599998	261.832977	35.606129	3.444812	228.064667
35.619999	248.667007	35.626129	2.201249	227.767609
35.639999	237.167007	35.646130	1.429559	227.471405
35.660000	233.833008	35.666130	0.946196	227.175812
35.680000	240.000000	35.686131	0.640776	226.880951
35.700001	232.167007	35.706131	0.444709	226.586792
35.720001	218.167007	35.726131	0.316087	226.293335
35.740002	231.333008	35.746132	0.229663	226.000702
35.760002	227.000000	35.766132	0.170211	225.708679
35.780003	225.167007	35.786133	0.128410	225.417419
35.799999	219.000000	35.806129	0.098436	225.126953
35.820000	232.000000	35.826130	0.076547	224.837067
35.840000	225.833008	35.846130	0.060304	224.548035
35.860001	216.500000	35.866131	0.048073	224.259674
35.880001	222.833008	35.886131	0.038739	223.971985
35.900002	219.000000	35.906132	0.031529	223.684998
35.920002	216.000000	35.926136	0.025894	223.398773
35.940002	218.667007	35.946136	0.021447	223.113281
35.960003	206.833008	35.966137	0.017443	222.828522
35.980003	219.833008	35.986137	0.014613	222.544495
36.000000	220.667007	36.006134	0.012322	222.261169
36.020000	219.167007	36.026134	0.010453	221.978577
36.040001	214.833008	36.046135	0.008917	221.696716

36.060001	225.333008	36.066135	0.007646	221.415588
36.080002	220.667007	36.086136	0.006589	221.135193
36.099998	208.500000	36.106133	0.005705	220.855530
36.119999	221.333008	36.126133	0.004960	220.576599
36.139999	209.500000	36.146133	0.004331	220.298340
36.160000	214.167007	36.166134	0.003796	220.020874
36.180000	211.667007	36.186134	0.003339	219.744080
36.200001	210.500000	36.206135	0.002947	219.468018
36.220001	215.667007	36.226135	0.002610	219.192688
36.240002	218.167007	36.246132	0.002318	218.918121
36.260002	208.667007	36.266132	0.002066	218.644287
36.280003	219.667007	36.286133	0.001846	218.371155
36.299999	211.167007	36.306129	0.001653	218.098785
36.320000	208.667007	36.326130	0.001485	217.827118
36.340000	218.167007	36.346130	0.001337	217.556183
36.360001	210.500000	36.366131	0.001206	217.285950
36.380001	214.333008	36.386131	0.001091	217.016479
36.400002	211.667007	36.406132	0.000989	216.747742
36.420002	221.667007	36.426136	0.000898	216.479675
36.440002	217.833008	36.446136	0.000817	216.212372
36.460003	203.000000	36.466137	0.000745	215.945831
36.480003	210.167007	36.486137	0.000681	215.679962
36.500000	206.000000	36.506134	0.000623	215.414917
36.520000	208.167007	36.526134	0.000571	215.150604
36.540001	202.667007	36.546135	0.000524	214.886932
36.560001	204.000000	36.566135	0.000483	214.624023
36.580002	203.500000	36.586136	0.000445	214.361877
36.599998	210.833008	36.606133	0.000410	214.100494
36.619999	215.500000	36.626133	0.000379	213.839813
36.639999	205.333008	36.646133	0.000351	213.579773
36.660000	206.500000	36.666134	0.000325	213.320587
36.680000	207.167007	36.686134	0.000301	213.062103
36.700001	203.000000	36.706135	0.000280	212.804291
36.720001	200.500000	36.726135	0.000260	212.547241
36.740002	208.000000	36.746136	0.000242	212.290924
36.760002	214.000000	36.766136	0.000226	212.035370
36.780003	207.667007	36.786137	0.000211	211.780518
36.799999	206.333008	36.806133	0.000197	211.526428

36.820000	206.000000	36.826134	0.000185	211.273071
36.840000	200.500000	36.846134	0.000173	211.020386
36.860001	202.500000	36.866135	0.000162	210.768433
36.880001	203.000000	36.886135	0.000153	210.517303
36.900002	198.000000	36.906136	0.000144	210.266846
36.920002	193.833008	36.926140	0.000135	210.017059
36.940002	207.333008	36.946140	0.000128	209.768066
36.960003	198.333008	36.966137	0.000121	209.519836
36.980003	202.000000	36.986137	0.000114	209.272278
37.000000	190.833008	37.006134	0.000108	209.025543
37.020000	195.667007	37.026134	0.000103	208.779449
37.040001	196.500000	37.046135	0.000098	208.534119
37.060001	196.167007	37.066135	0.000093	208.289520
37.080002	202.500000	37.086136	0.000089	208.045624
37.099998	192.667007	37.106133	0.000085	207.802521
37.119999	199.500000	37.126133	0.000009	207.560120
37.139999	203.667007	37.146133	0.000010	207.318420
37.160000	202.833008	37.166134	0.000011	207.077423
37.180000	201.500000	37.186134	0.000012	206.837189
37.200001	205.333008	37.206135	0.000014	206.597717
37.220001	199.500000	37.226135	0.000015	206.358978
37.240002	204.000000	37.246136	0.000017	206.120880
37.260002	197.000000	37.266136	0.000019	205.883545
37.280003	209.667007	37.286137	0.000021	205.646973
37.299999	211.833008	37.306133	0.000024	205.411133
37.320000	204.833008	37.326134	0.000027	205.176025
37.340000	217.000000	37.346134	0.000031	204.941620
37.360001	212.833008	37.366135	0.000035	204.707886
37.380001	201.333008	37.386135	0.000039	204.474915
37.400002	207.500000	37.406139	0.000045	204.242615
37.420002	195.833008	37.426140	0.000052	204.011139
37.440002	193.000000	37.446140	0.000059	203.780365
37.460003	200.167007	37.466141	0.000068	203.550293
37.480003	201.833008	37.486141	0.000085	203.320892
37.500000	196.500000	37.506138	0.000098	203.092316
37.520000	193.667007	37.526138	0.000117	202.864441
37.540001	191.167007	37.546139	0.000136	202.637329
37.560001	198.333008	37.566139	0.000160	202.410797

37.580002	186.333008	37.586140	0.000188	202.185089
37.599998	193.500000	37.606136	0.000223	201.960114
37.619999	204.500000	37.626137	0.000266	201.735779
37.639999	196.000000	37.646137	0.000319	201.512238
37.660000	191.667007	37.666134	0.000386	201.289429
37.680000	198.000000	37.686134	0.000470	201.067230
37.700001	187.167007	37.706135	0.000577	200.845825
37.720001	193.333008	37.726135	0.000715	200.625122
37.740002	196.500000	37.746136	0.000895	200.405121
37.760002	191.833008	37.766136	0.001141	200.185913
37.780003	201.500000	37.786137	0.001458	199.967316
37.799999	202.167007	37.806133	0.001884	199.749481
37.820000	202.833008	37.826134	0.002470	199.532379
37.840000	192.500000	37.846134	0.003285	199.315948
37.860001	205.833008	37.866135	0.004441	199.100220
37.880001	195.667007	37.886139	0.006114	198.885193
37.900002	198.167007	37.906139	0.008588	198.670959
37.920002	188.833008	37.926140	0.012337	198.457367
37.940002	196.500000	37.946140	0.018184	198.244507
37.960003	194.667007	37.966141	0.027630	198.032379
37.980003	192.667007	37.986141	0.043572	197.820923
38.000000	182.667007	38.006138	0.071972	197.610229
38.020000	189.500000	38.026138	0.125764	197.400177
38.040001	194.667007	38.046139	0.233627	197.190857
38.060001	191.000000	38.066139	0.458756	196.982300
38.080002	189.500000	38.086140	0.935301	196.774353
38.099998	192.667007	38.106136	1.926429	196.567169
38.119999	190.833008	38.126137	3.861547	196.360687
38.139999	199.000000	38.146137	7.085229	196.154877
38.160000	190.167007	38.166138	10.727380	195.949768
38.180000	200.833008	38.186138	11.765237	195.745361
38.200001	180.000000	38.206139	8.754799	195.541656
38.220001	184.000000	38.226139	4.771246	195.338654
38.240002	177.833008	38.246140	2.246351	195.136414
38.260002	181.833008	38.266140	1.062976	194.934784
38.280003	185.667007	38.286140	0.546342	194.733887
38.299999	189.333008	38.306137	0.305340	194.533691
38.320000	189.000000	38.326138	0.179853	194.334167

38.340000	185.333008	38.346138	0.109054	194.135376
38.360001	184.000000	38.366142	0.067543	193.937225
38.380001	180.167007	38.386139	0.042829	193.739807
38.400002	190.500000	38.406139	0.027932	193.542999
38.420002	189.000000	38.426140	0.018819	193.346985
38.440002	189.500000	38.446140	0.013142	193.151642
38.460003	181.000000	38.466141	0.009544	192.956940
38.480003	186.667007	38.486141	0.007237	192.762970
38.500000	183.333008	38.506138	0.005759	192.569672
38.520000	175.667007	38.526138	0.004834	192.377045
38.540001	178.333008	38.546139	0.004296	192.185150
38.560001	190.667007	38.566139	0.004047	191.993927
38.580002	182.667007	38.586140	0.004029	191.803314
38.599998	177.833008	38.606136	0.004214	191.613495
38.619999	181.333008	38.626137	0.004598	191.424286
38.639999	178.667007	38.646137	0.005192	191.235779
38.660000	187.167007	38.666138	0.006027	191.047913
38.680000	186.833008	38.686138	0.007155	190.860748
38.700001	170.167007	38.706139	0.008654	190.674255
38.720001	179.667007	38.726139	0.010636	190.488434
38.740002	179.000000	38.746140	0.013266	190.303284
38.760002	179.667007	38.766140	0.016773	190.118835
38.780003	184.833008	38.786140	0.021498	189.935089
38.799999	187.667007	38.806137	0.027938	189.751953
38.820000	181.500000	38.826138	0.036844	189.569458
38.840000	187.833008	38.846142	0.049354	189.387634
38.860001	191.000000	38.866142	0.067229	189.206573
38.880001	188.167007	38.886143	0.093278	189.026093
38.900002	183.500000	38.906143	0.132073	188.846283
38.920002	188.833008	38.926144	0.191284	188.667175
38.940002	185.000000	38.946144	0.284253	188.488708
38.960003	180.500000	38.966145	0.435265	188.310883
38.980003	179.333008	38.986145	0.691097	188.133820
39.000000	173.833008	39.006142	1.147453	187.957336
39.020000	176.167007	39.026142	2.011356	187.781586
39.040001	191.667007	39.046143	3.742893	187.606354
39.060001	187.833008	39.066143	7.365654	187.431885
39.080002	190.667007	39.086143	15.088283	187.258057

39.099998	211.833008	39.106140	31.349220	187.084900
39.119999	259.500000	39.126137	63.747673	186.912384
39.139999	343.000000	39.146137	120.019623	186.740448
39.160000	449.166992	39.166138	190.789703	186.569275
39.180000	479.666992	39.186138	226.610672	186.398682
39.200001	424.000000	39.206139	185.206375	186.228668
39.220001	328.500000	39.226139	108.497833	186.059418
39.240002	250.167007	39.246140	52.607708	185.890717
39.260002	206.500000	39.266140	24.636911	185.722778
39.280003	196.000000	39.286140	12.271816	185.555389
39.299999	189.333008	39.306141	6.639717	185.388672
39.320000	182.167007	39.326141	3.818336	185.222534
39.340000	187.333008	39.346142	2.282639	185.057129
39.360001	178.667007	39.366142	1.410492	184.892242
39.380001	174.167007	39.386143	0.909779	184.728149
39.400002	173.833008	39.406143	0.625504	184.564606
39.420002	185.667007	39.426144	0.471518	184.401703
39.440002	182.500000	39.446144	0.400891	184.239410
39.460003	181.833008	39.466145	0.390472	184.077759
39.480003	186.833008	39.486145	0.432766	183.916748
39.500000	186.833008	39.506142	0.532708	183.756439
39.520000	182.333008	39.526142	0.708634	183.596649
39.540001	180.333008	39.546143	0.997737	183.437500
39.560001	183.167007	39.566143	1.469285	183.279022
39.580002	177.167007	39.586143	2.253230	183.121094
39.599998	183.667007	39.606140	3.603733	182.963898
39.619999	193.833008	39.626141	6.047985	182.807220
39.639999	188.167007	39.646141	10.730666	182.651184
39.660000	190.333008	39.666142	20.208353	182.495758
39.680000	217.667007	39.686142	40.179886	182.340942
39.700001	260.666992	39.706142	82.939545	182.186829
39.720001	350.832977	39.726143	173.074295	182.033264
39.740002	546.333008	39.746143	351.752228	181.880310
39.760002	886.333008	39.766148	657.179932	181.727905
39.780003	1218.669922	39.786148	1028.052368	181.576172
39.799999	1332.830078	39.806145	1196.877441	181.425079
39.820000	1122.669922	39.826145	963.785706	181.274567
39.840000	782.833008	39.846146	561.770691	181.124603

39.860001	504.332977	39.866142	272.810425	180.975311
39.880001	318.832977	39.886143	127.967537	180.826599
39.900002	251.167007	39.906143	63.555355	180.678467
39.920002	219.500000	39.926144	34.088459	180.530945
39.940002	203.667007	39.946144	19.334902	180.384033
39.960003	196.500000	39.966145	11.332440	180.237640
39.980003	186.333008	39.986145	6.791516	180.091949
40.000000	183.167007	40.006142	4.159059	179.946808
40.020000	187.167007	40.026142	2.609791	179.802277
40.040001	183.833008	40.046143	1.682747	179.658325
40.060001	182.500000	40.066143	1.116500	179.514923
40.080002	182.833008	40.086143	0.763038	179.372131
40.099998	182.000000	40.106140	0.538329	179.229980
40.119999	178.667007	40.126141	0.394180	179.088348
40.139999	176.000000	40.146141	0.303045	178.947296
40.160000	187.333008	40.166142	0.249587	178.806793
40.180000	174.833008	40.186142	0.226631	178.666931
40.200001	181.833008	40.206142	0.233775	178.527588
40.220001	181.500000	40.226147	0.278559	178.388855
40.240002	172.833008	40.246147	0.381473	178.250702
40.260002	184.667007	40.266148	0.589223	178.113068
40.280003	179.167007	40.286148	1.006583	177.976074
40.299999	175.167007	40.306145	1.869984	177.839630
40.320000	189.833008	40.326145	3.709800	177.703766
40.340000	176.333008	40.346146	7.671915	177.568481
40.360001	189.667007	40.366146	16.043547	177.433655
40.380001	203.667007	40.386147	32.609913	177.299530
40.400002	240.167007	40.406147	60.709923	177.165894
40.420002	280.666992	40.426147	94.376274	177.032837
40.440002	275.166992	40.446148	109.353905	176.900299
40.460003	262.666992	40.466148	88.190407	176.768341
40.480003	225.167007	40.486149	51.754791	176.636963
40.500000	198.333008	40.506145	25.299290	176.506104
40.520000	181.667007	40.526146	11.890593	176.375824
40.540001	179.167007	40.546146	5.886463	176.246063
40.560001	162.000000	40.566147	3.142121	176.116821
40.580002	164.833008	40.586147	1.783371	175.988190
40.599998	166.167007	40.606140	1.065999	175.860107

40.619999	169.167007	40.626141	0.685830	175.732574
40.639999	169.000000	40.646141	0.512965	175.605560
40.660000	169.333008	40.666142	0.506842	175.479004
40.680000	178.333008	40.686146	0.706316	175.353058
40.700001	175.333008	40.706146	1.263257	175.227661
40.720001	172.167007	40.726147	2.514040	175.102753
40.740002	178.000000	40.746147	5.023748	174.978485
40.760002	182.000000	40.766148	9.270282	174.854584
40.780003	199.333008	40.786148	14.293456	174.731323
40.799999	199.500000	40.806145	16.440018	174.608612
40.820000	187.000000	40.826145	13.210810	174.486359
40.840000	183.167007	40.846146	7.758978	174.364655
40.860001	179.833008	40.866146	3.802805	174.243500
40.880001	183.000000	40.886147	1.790477	174.122772
40.900002	170.833008	40.906147	0.885236	174.002716
40.920002	179.833008	40.926147	0.469924	173.883026
40.940002	180.833008	40.946148	0.263383	173.763947
40.960003	179.833008	40.966148	0.152722	173.645386
40.980003	179.833008	40.986149	0.090743	173.527252
41.000000	185.667007	41.006145	0.055221	173.409729
41.020000	193.667007	41.026146	0.034509	173.292633
41.040001	188.000000	41.046146	0.022205	173.176056
41.060001	189.000000	41.066147	0.014725	173.060028
41.080002	183.833008	41.086147	0.010059	172.944458
41.099998	191.833008	41.106144	0.007073	172.829437
41.119999	180.000000	41.126148	0.005114	172.714935
41.139999	177.000000	41.146149	0.003803	172.600922
41.160000	170.333008	41.166149	0.002909	172.487366
41.180000	166.000000	41.186150	0.002294	172.374329
41.200001	165.167007	41.206150	0.001867	172.261749
41.220001	174.833008	41.226151	0.001575	172.149658
41.240002	170.000000	41.246151	0.001380	172.038086
41.260002	173.167007	41.266151	0.001259	171.927032
41.280003	180.667007	41.286152	0.001198	171.816437
41.299999	163.333008	41.306149	0.001189	171.706329
41.320000	180.167007	41.326149	0.001228	171.596710
41.340000	183.833008	41.346149	0.001314	171.487549
41.360001	173.333008	41.366146	0.001452	171.378906

41.380001	167.000000	41.386147	0.001648	171.270782
41.400002	166.500000	41.406147	0.001915	171.162994
41.420002	173.000000	41.426147	0.002269	171.055817
41.440002	169.833008	41.446148	0.002733	170.949005
41.460003	168.333008	41.466148	0.003341	170.842743
41.480003	173.167007	41.486149	0.004139	170.736938
41.500000	164.333008	41.506145	0.005191	170.631561
41.520000	176.333008	41.526146	0.006590	170.526703
41.540001	169.333008	41.546146	0.008467	170.422272
41.560001	164.500000	41.566151	0.011008	170.318268
41.580002	172.167007	41.586151	0.014502	170.214813
41.599998	178.833008	41.606148	0.019362	170.111755
41.619999	184.000000	41.626148	0.026225	170.009186
41.639999	198.333008	41.646149	0.036076	169.907013
41.660000	192.333008	41.666149	0.050469	169.805359
41.680000	200.000000	41.686150	0.071922	169.704132
41.700001	194.000000	41.706150	0.104600	169.603363
41.720001	199.000000	41.726151	0.155605	169.503052
41.740002	198.833008	41.746151	0.237451	169.403137
41.760002	185.000000	41.766151	0.373112	169.303711
41.780003	189.000000	41.786152	0.606925	169.204773
41.799999	183.667007	41.806149	1.029259	169.106201
41.820000	179.333008	41.826149	1.834792	169.008087
41.840000	186.500000	41.846149	3.457339	168.910431
41.860001	182.667007	41.866150	6.875757	168.813141
41.880001	189.833008	41.886150	14.258376	168.716400
41.900002	202.833008	41.906151	30.142033	168.619995
41.920002	228.000000	41.926151	62.861908	168.524078
41.940002	303.000000	41.946152	123.281639	168.428558
41.960003	403.500000	41.966152	211.230820	168.333496
41.980003	466.666992	41.986153	285.961823	168.238800
42.000000	459.832977	42.006153	279.170288	168.144623
42.020000	388.832977	42.026154	193.690750	168.050812
42.040001	285.000000	42.046154	104.187813	167.957428
42.060001	231.667007	42.066154	49.809383	167.864410
42.080002	182.333008	42.086155	23.794502	167.771851
42.099998	176.500000	42.106152	12.065782	167.679779
42.119999	168.500000	42.126148	6.566949	167.588043

42.139999	180.167007	42.146149	3.854458	167.496674
42.160000	169.167007	42.166149	2.569039	167.405792
42.180000	171.000000	42.186150	2.227099	167.315338
42.200001	165.500000	42.206150	2.842441	167.225250
42.220001	180.833008	42.226151	4.929854	167.135529
42.240002	184.667007	42.246151	9.655981	167.046234
42.260002	200.333008	42.266151	18.646236	166.957367
42.280003	223.333008	42.286152	32.010269	166.868927
42.299999	238.500000	42.306149	43.858532	166.780823
42.320000	235.333008	42.326149	43.651867	166.693115
42.340000	222.167007	42.346149	30.921097	166.605804
42.360001	191.000000	42.366150	16.889803	166.518951
42.380001	167.500000	42.386150	8.126783	166.432404
42.400002	159.500000	42.406151	3.874209	166.346313
42.420002	168.167007	42.426151	1.946201	166.260559
42.440002	164.000000	42.446156	1.037753	166.175140
42.460003	167.000000	42.466156	0.577966	166.090179
42.480003	157.000000	42.486156	0.331359	166.005585
42.500000	153.167007	42.506153	0.194543	165.921417
42.520000	162.167007	42.526154	0.117035	165.837555
42.540001	160.333008	42.546154	0.072329	165.754120
42.560001	159.667007	42.566154	0.045989	165.671051
42.580002	156.833008	42.586155	0.030080	165.588287
42.599998	162.500000	42.606152	0.020211	165.506012
42.619999	157.167007	42.626152	0.013921	165.424042
42.639999	157.833008	42.646152	0.009810	165.342407
42.660000	157.333008	42.666153	0.007057	165.261200
42.680000	156.167007	42.686153	0.005175	165.180298
42.700001	159.667007	42.706154	0.003861	165.099823
42.720001	162.500000	42.726154	0.002928	165.019684
42.740002	153.833008	42.746155	0.002254	164.939911
42.760002	167.167007	42.766155	0.001760	164.860443
42.780003	163.333008	42.786156	0.001393	164.781403
42.799999	160.500000	42.806152	0.001118	164.702667
42.820000	157.167007	42.826153	0.000909	164.624298
42.840000	159.500000	42.846153	0.000749	164.546265
42.860001	162.333008	42.866158	0.000627	164.468597
42.880001	166.500000	42.886158	0.000532	164.391266

42.900002	156.833008	42.906155	0.000460	164.314301
42.920002	161.500000	42.926155	0.000405	164.237640
42.940002	169.833008	42.946156	0.000365	164.161346
42.960003	163.500000	42.966156	0.000336	164.085388
42.980003	157.833008	42.986156	0.000317	164.009796
43.000000	162.833008	43.006153	0.000307	163.934479
43.020000	169.000000	43.026154	0.000307	163.859589
43.040001	164.833008	43.046154	0.000314	163.784943
43.060001	164.000000	43.066154	0.000331	163.710693
43.080002	167.667007	43.086155	0.000357	163.636749
43.099998	161.667007	43.106152	0.000394	163.563141
43.119999	158.000000	43.126152	0.000444	163.489838
43.139999	156.500000	43.146152	0.000509	163.416901
43.160000	167.667007	43.166153	0.000593	163.344238
43.180000	166.667007	43.186153	0.000700	163.271881
43.200001	164.167007	43.206154	0.000836	163.199860
43.220001	162.000000	43.226154	0.001009	163.128204
43.240002	156.833008	43.246155	0.001231	163.056824
43.260002	150.667007	43.266155	0.001517	162.985779
43.280003	154.500000	43.286160	0.001886	162.915009
43.299999	160.000000	43.306156	0.002367	162.844574
43.320000	160.667007	43.326157	0.002999	162.774445
43.340000	158.833008	43.346157	0.003838	162.704651
43.360001	170.167007	43.366158	0.004962	162.635162
43.380001	167.333008	43.386158	0.006484	162.565918
43.400002	169.500000	43.406158	0.008570	162.497040
43.420002	167.333008	43.426159	0.011468	162.428436
43.440002	173.000000	43.446159	0.015552	162.360138
43.460003	170.667007	43.466160	0.021393	162.292145
43.480003	181.667007	43.486160	0.029892	162.224396
43.500000	174.667007	43.506157	0.042481	162.157074
43.520000	162.167007	43.526157	0.061515	162.089935
43.540001	164.833008	43.546158	0.090928	162.023102
43.560001	167.833008	43.566158	0.137504	161.956573
43.580002	172.667007	43.586159	0.213321	161.890350
43.599998	167.500000	43.606155	0.340710	161.824371
43.619999	164.500000	43.626156	0.563104	161.758698
43.639999	163.000000	43.646156	0.969232	161.693298

43.660000	165.833008	43.666157	1.749960	161.628204
43.680000	167.167007	43.686153	3.330889	161.563385
43.700001	161.833008	43.706158	6.678271	161.498840
43.720001	175.000000	43.726158	13.945841	161.434540
43.740002	188.500000	43.746159	29.700037	161.370575
43.760002	224.500000	43.766159	62.508968	161.306854
43.780003	275.000000	43.786160	124.254807	161.243378
43.799999	368.500000	43.806156	218.146439	161.180206
43.820000	440.666992	43.826157	309.197754	161.117340
43.840000	444.332977	43.846157	324.252930	161.054657
43.860001	382.832977	43.866158	244.281723	160.992310
43.880001	292.332977	43.886158	140.420654	160.930206
43.900002	236.833008	43.906158	69.328331	160.868378
43.920002	209.833008	43.926159	33.003517	160.806732
43.940002	179.833008	43.946159	16.253588	160.745392
43.960003	171.833008	43.966160	8.437861	160.684387
43.980003	168.000000	43.986160	4.573496	160.623535
44.000000	163.833008	44.006157	2.556127	160.563019
44.020000	154.667007	44.026157	1.465624	160.502716
44.040001	154.333008	44.046158	0.865044	160.442657
44.060001	157.667007	44.066158	0.531044	160.382874
44.080002	159.500000	44.086159	0.345902	160.323334
44.099998	159.167007	44.106155	0.247895	160.264038
44.119999	158.000000	44.126160	0.206616	160.204987
44.139999	158.167007	44.146160	0.211457	160.146179
44.160000	160.167007	44.166161	0.268433	160.087616
44.180000	158.500000	44.186161	0.405960	160.029266
44.200001	157.333008	44.206161	0.695175	159.971222
44.220001	154.000000	44.226162	1.301252	159.913361
44.240002	163.167007	44.246162	2.602162	159.855743
44.260002	171.333008	44.266163	5.442368	159.798370
44.280003	172.667007	44.286163	11.600370	159.741180
44.299999	180.667007	44.306160	24.355764	159.684296
44.320000	192.667007	44.326160	48.061451	159.627686
44.340000	212.833008	44.346161	83.238808	159.571198
44.360001	224.000000	44.366161	115.737297	159.515015
44.380001	224.833008	44.386162	118.983101	159.459015
44.400002	203.667007	44.406162	88.373962	159.403290

44.420002	192.500000	44.426163	50.507782	159.347748
44.440002	182.500000	44.446163	24.948814	159.292450
44.460003	183.500000	44.466160	11.906510	159.237335
44.480003	167.667007	44.486160	5.867290	159.182465
44.500000	170.500000	44.506157	3.039322	159.127838
44.520000	174.000000	44.526157	1.639849	159.073364
44.540001	171.500000	44.546162	0.910574	159.019135
44.560001	164.167007	44.566162	0.517549	158.965118
44.580002	153.167007	44.586163	0.301057	158.911346
44.599998	157.167007	44.606159	0.179640	158.857758
44.619999	163.000000	44.626160	0.110143	158.804413
44.639999	156.500000	44.646160	0.069447	158.751251
44.660000	157.833008	44.666161	0.045018	158.698273
44.680000	156.000000	44.686161	0.029992	158.645508
44.700001	158.500000	44.706161	0.020541	158.592987
44.720001	156.000000	44.726162	0.014491	158.540649
44.740002	160.833008	44.746162	0.010576	158.488495
44.760002	161.167007	44.766163	0.008049	158.436554
44.780003	161.333008	44.786163	0.006463	158.384796
44.799999	147.167007	44.806160	0.005556	158.333252
44.820000	151.500000	44.826160	0.005184	158.281952
44.840000	151.333008	44.846161	0.005288	158.230743
44.860001	160.833008	44.866161	0.005880	158.179840
44.880001	164.000000	44.886162	0.007047	158.129059
44.900002	154.500000	44.906162	0.008966	158.078522
44.920002	156.000000	44.926163	0.011946	158.028107
44.940002	150.833008	44.946167	0.016503	157.977905
44.960003	148.500000	44.966167	0.023489	157.927887
44.980003	163.833008	44.986168	0.034334	157.878082
45.000000	157.500000	45.006165	0.051485	157.828461
45.020000	155.667007	45.026165	0.079260	157.779022
45.040001	144.167007	45.046165	0.125494	157.729706
45.060001	144.333008	45.066166	0.205023	157.680634
45.080002	151.833008	45.086166	0.347289	157.631714
45.099998	160.500000	45.106163	0.613667	157.582977
45.119999	153.000000	45.126163	1.138419	157.534424
45.139999	150.333008	45.146164	2.223558	157.486053
45.160000	150.833008	45.166164	4.553729	157.437836

45.180000	164.500000	45.186165	9.644253	157.389801
45.200001	171.833008	45.206165	20.626245	157.341919
45.220001	198.000000	45.226166	43.032303	157.294220
45.240002	258.832977	45.246166	83.325546	157.246735
45.260002	316.666992	45.266163	139.504059	157.199371
45.280003	383.666992	45.286163	185.127594	157.152161
45.299999	380.666992	45.306160	181.187881	157.105194
45.320000	320.166992	45.326160	129.615845	157.058319
45.340000	256.166992	45.346161	72.712807	157.011627
45.360001	199.333008	45.366165	35.815468	156.965057
45.380001	174.167007	45.386166	17.166323	156.918732
45.400002	162.833008	45.406166	8.488115	156.872528
45.420002	160.167007	45.426167	4.391672	156.826447
45.440002	161.500000	45.446167	2.358299	156.780548
45.460003	159.500000	45.466167	1.301278	156.734833
45.480003	152.833008	45.486168	0.734602	156.689240
45.500000	161.500000	45.506165	0.424473	156.643799
45.520000	162.167007	45.526165	0.251515	156.598541
45.540001	154.833008	45.546165	0.153073	156.553436
45.560001	153.667007	45.566166	0.095704	156.508453
45.580002	160.833008	45.586166	0.061409	156.463654
45.599998	154.833008	45.606163	0.040377	156.418976
45.619999	144.833008	45.626163	0.027150	156.374451
45.639999	151.667007	45.646164	0.018641	156.330048
45.660000	147.333008	45.666164	0.013050	156.285858
45.680000	153.833008	45.686165	0.009306	156.241730
45.700001	148.500000	45.706165	0.006755	156.197784
45.720001	148.333008	45.726166	0.004990	156.153961
45.740002	146.000000	45.746166	0.003754	156.110321
45.760002	151.833008	45.766171	0.002880	156.066742
45.780003	153.333008	45.786171	0.002260	156.023407
45.799999	160.500000	45.806168	0.001819	155.980133
45.820000	151.667007	45.826168	0.001509	155.937042
45.840000	155.000000	45.846169	0.001297	155.894043
45.860001	152.167007	45.866169	0.001162	155.851227
45.880001	150.000000	45.886169	0.001091	155.808502
45.900002	148.833008	45.906170	0.001074	155.765930
45.920002	156.333008	45.926170	0.001109	155.723480

45.940002	150.333008	45.946171	0.001198	155.681152
45.960003	150.000000	45.966171	0.001346	155.638977
45.980003	150.333008	45.986172	0.001565	155.596893
46.000000	152.167007	46.006168	0.001873	155.554962
46.020000	155.167007	46.026169	0.002298	155.513153
46.040001	150.333008	46.046169	0.002886	155.471497
46.060001	150.167007	46.066170	0.003702	155.429901
46.080002	145.833008	46.086166	0.004854	155.388519
46.099998	142.333008	46.106163	0.006515	155.347198
46.119999	149.333008	46.126163	0.008974	155.306030
46.139999	148.667007	46.146164	0.012735	155.264923
46.160000	148.833008	46.166168	0.018726	155.223969
46.180000	147.667007	46.186169	0.028729	155.183167
46.200001	144.333008	46.206169	0.046398	155.142456
46.220001	142.000000	46.226170	0.079584	155.101837
46.240002	158.167007	46.246170	0.145736	155.061371
46.260002	149.500000	46.266171	0.283922	155.020966
46.280003	147.000000	46.286171	0.579322	154.980713
46.299999	143.500000	46.306168	1.203573	154.940613
46.320000	160.833008	46.326168	2.446018	154.900543
46.340000	163.000000	46.346169	4.599940	154.860657
46.360001	169.833008	46.366169	7.437165	154.820831
46.380001	171.833008	46.386169	9.523204	154.781158
46.400002	177.000000	46.406170	9.150616	154.741547
46.420002	172.833008	46.426170	6.795025	154.702057
46.440002	167.333008	46.446171	4.567881	154.662659
46.460003	172.333008	46.466171	3.676626	154.623383
46.480003	162.667007	46.486172	4.306529	154.584259
46.500000	166.333008	46.506168	6.767671	154.545197
46.520000	169.667007	46.526169	12.358788	154.506256
46.540001	178.000000	46.546169	24.458937	154.467377
46.560001	207.833008	46.566174	50.913151	154.428619
46.580002	267.166992	46.586174	108.838776	154.389984
46.599998	360.000000	46.606171	232.148346	154.351440
46.619999	596.833008	46.626171	475.176880	154.312958
46.639999	1036.330078	46.646172	883.095093	154.274597
46.660000	1540.000000	46.666172	1383.570435	154.236359
46.680000	1832.500000	46.686172	1684.719482	154.198212

46.700001	1664.330078	46.706173	1512.290405	154.160156
46.720001	1165.669922	46.726173	1014.656372	154.122131
46.740002	703.333008	46.746174	551.636108	154.084259
46.760002	411.666992	46.766174	270.510773	154.046478
46.780003	289.000000	46.786175	130.644165	154.008789
46.799999	230.667007	46.806171	65.038605	153.971191
46.820000	217.000000	46.826172	33.662937	153.933685
46.840000	213.000000	46.846172	17.990334	153.896271
46.860001	217.500000	46.866173	9.856596	153.858948
46.880001	216.500000	46.886173	5.522897	153.821686
46.900002	211.833008	46.906170	3.168576	153.784546
46.920002	210.500000	46.926170	1.864636	153.747467
46.940002	207.000000	46.946175	1.126941	153.710541
46.960003	217.833008	46.966175	0.699589	153.673615
46.980003	211.833008	46.986176	0.445546	153.636780
47.000000	195.000000	47.006172	0.290633	153.600067
47.020000	180.833008	47.026173	0.193782	153.563446
47.040001	169.667007	47.046173	0.131837	153.526886
47.060001	158.833008	47.066174	0.091365	153.490387
47.080002	170.333008	47.086174	0.064400	153.454010
47.099998	156.000000	47.106171	0.046110	153.417725
47.119999	152.500000	47.126171	0.033493	153.381500
47.139999	152.000000	47.146172	0.024657	153.345306
47.160000	140.333008	47.166172	0.018381	153.309235
47.180000	141.833008	47.186172	0.013867	153.273285
47.200001	151.667007	47.206173	0.010580	153.237366
47.220001	146.167007	47.226173	0.008163	153.201538
47.240002	146.000000	47.246174	0.006368	153.165741
47.260002	141.833008	47.266174	0.005025	153.130096
47.280003	148.000000	47.286175	0.004014	153.094452
47.299999	144.333008	47.306171	0.003251	153.058929
47.320000	140.833008	47.326172	0.002676	153.023468
47.340000	146.667007	47.346176	0.002245	152.988068
47.360001	141.500000	47.366177	0.001929	152.952789
47.380001	144.000000	47.386177	0.001706	152.917542
47.400002	140.333008	47.406178	0.001564	152.882355
47.420002	145.500000	47.426178	0.001494	152.847260
47.440002	147.000000	47.446178	0.001494	152.812225

47.460003	147.000000	47.466179	0.001567	152.777313
47.480003	148.500000	47.486179	0.001721	152.742401
47.500000	148.833008	47.506176	0.001974	152.707550
47.520000	144.167007	47.526176	0.002348	152.672821
47.540001	147.833008	47.546177	0.002883	152.638153
47.560001	146.667007	47.566177	0.003634	152.603546
47.580002	142.333008	47.586178	0.004682	152.568970
47.599998	146.000000	47.606174	0.006149	152.534485
47.619999	146.333008	47.626175	0.008216	152.500092
47.639999	143.167007	47.646175	0.011156	152.465729
47.660000	148.000000	47.666176	0.015391	152.431427
47.680000	150.500000	47.686176	0.021576	152.397217
47.700001	158.500000	47.706177	0.030754	152.363068
47.720001	151.000000	47.726181	0.044616	152.328949
47.740002	152.667007	47.746178	0.065946	152.294922
47.760002	155.500000	47.766178	0.099481	152.260956
47.780003	156.833008	47.786179	0.153432	152.227020
47.799999	161.167007	47.806175	0.242461	152.193146
47.820000	163.500000	47.826176	0.393726	152.159363
47.840000	160.500000	47.846176	0.659339	152.125641
47.860001	163.167007	47.866177	1.143981	152.092010
47.880001	165.167007	47.886177	2.067846	152.058350
47.900002	162.500000	47.906178	3.913629	152.024811
47.920002	177.500000	47.926178	7.768967	151.991333
47.940002	182.333008	47.946178	16.098082	151.957886
47.960003	205.333008	47.966179	34.345642	151.924530
47.980003	246.500000	47.986179	73.711411	151.891205
48.000000	338.666992	48.006176	153.797287	151.857910
48.020000	470.500000	48.026176	297.480072	151.824707
48.040001	720.500000	48.046177	499.751556	151.791565
48.060001	885.166992	48.066177	675.265015	151.758453
48.080002	883.666992	48.086178	688.453125	151.725464
48.099998	718.000000	48.106178	522.243225	151.692474
48.119999	494.500000	48.126179	310.610992	151.659515
48.139999	327.500000	48.146179	159.280502	151.626617
48.160000	227.000000	48.166180	77.398254	151.593781
48.180000	179.833008	48.186180	37.924088	151.561005
48.200001	162.333008	48.206181	19.190329	151.528351

48.220001	153.333008	48.226181	10.031487	151.495667
48.240002	152.333008	48.246181	5.386755	151.463013
48.260002	150.167007	48.266182	2.964943	151.430450
48.280003	162.667007	48.286182	1.679024	151.397949
48.299999	157.833008	48.306179	0.989457	151.365509
48.320000	161.167007	48.326180	0.621872	151.333099
48.340000	167.500000	48.346180	0.438348	151.300751
48.360001	163.333008	48.366180	0.375208	151.268433
48.380001	166.000000	48.386181	0.416662	151.236145
48.400002	166.500000	48.406181	0.592288	151.203949
48.420002	159.167007	48.426182	1.001790	151.171783
48.440002	153.500000	48.446182	1.888761	151.139679
48.460003	154.000000	48.466183	3.817197	151.107605
48.480003	157.333008	48.486183	8.054210	151.075592
48.500000	173.000000	48.506184	17.285759	151.043640
48.520000	198.500000	48.526184	36.534836	151.011688
48.540001	231.000000	48.546185	72.820381	150.979828
48.560001	292.332977	48.566185	129.050888	150.947998
48.580002	345.000000	48.586185	188.992432	150.916229
48.599998	358.332977	48.606178	213.187988	150.884491
48.619999	334.500000	48.626179	179.143387	150.852814
48.639999	291.832977	48.646179	115.472321	150.821198
48.660000	221.167007	48.666180	61.999802	150.789581
48.680000	180.000000	48.686180	30.549171	150.758026
48.700001	154.500000	48.706181	14.877791	150.726501
48.720001	146.833008	48.726181	7.427559	150.695038
48.740002	147.000000	48.746181	3.828688	150.663635
48.760002	142.000000	48.766182	2.028666	150.632263
48.780003	146.833008	48.786182	1.100246	150.600952
48.799999	148.667007	48.806179	0.610516	150.569672
48.820000	155.167007	48.826180	0.347307	150.538422
48.840000	143.833008	48.846180	0.203280	150.507233
48.860001	144.000000	48.866184	0.122911	150.476074
48.880001	140.667007	48.886185	0.077221	150.444946
48.900002	138.833008	48.906185	0.050878	150.413910
48.920002	147.167007	48.926186	0.035708	150.382874
48.940002	141.333008	48.946186	0.027310	150.351898
48.960003	142.167007	48.966187	0.023338	150.320953

48.980003	143.333008	48.986187	0.022622	150.290070
49.000000	155.833008	49.006184	0.024739	150.259216
49.020000	146.000000	49.026184	0.029836	150.228424
49.040001	136.167007	49.046185	0.038621	150.197632
49.060001	145.500000	49.066185	0.052501	150.166901
49.080002	137.667007	49.086185	0.073917	150.136200
49.099998	137.000000	49.106182	0.106960	150.105560
49.119999	139.833008	49.126183	0.158544	150.074982
49.139999	147.833008	49.146183	0.240473	150.044434
49.160000	146.667007	49.166183	0.373397	150.013885
49.180000	142.333008	49.186184	0.594432	149.983398
49.200001	144.333008	49.206184	0.972443	149.952972
49.220001	145.500000	49.226185	1.640070	149.922577
49.240002	152.333008	49.246189	2.864157	149.892212
49.260002	158.333008	49.266190	5.204243	149.861908
49.280003	153.167007	49.286190	9.884906	149.831604
49.299999	169.833008	49.306187	19.655834	149.801361
49.320000	183.667007	49.326187	40.749523	149.771210
49.340000	228.833008	49.346188	86.952209	149.740997
49.360001	325.832977	49.366188	186.798569	149.710876
49.380001	555.333008	49.386189	391.125916	149.680786
49.400002	935.833008	49.406189	762.804199	149.650757
49.420002	1564.500000	49.426189	1303.791016	149.620758
49.440002	2093.500000	49.446190	1817.093140	149.590790
49.460003	2219.670166	49.466190	1939.072021	149.560883
49.480003	1803.830078	49.486187	1551.416382	149.530975
49.500000	1184.169922	49.506184	968.279968	149.501160
49.520000	703.000000	49.526184	512.883972	149.471344
49.540001	424.332977	49.546185	252.624252	149.441559
49.560001	279.000000	49.566185	123.598587	149.411835
49.580002	210.333008	49.586185	61.923683	149.382172
49.599998	188.833008	49.606182	31.941586	149.352478
49.619999	179.833008	49.626186	16.892729	149.322876
49.639999	174.833008	49.646187	9.134357	149.293304
49.660000	160.833008	49.666187	5.048725	149.263763
49.680000	158.667007	49.686188	2.857362	149.234283
49.700001	148.167007	49.706188	1.659006	149.204834
49.720001	150.333008	49.726189	0.989129	149.175385

49.740002	154.167007	49.746189	0.605489	149.146027
49.760002	159.000000	49.766190	0.380155	149.116669
49.780003	151.167007	49.786190	0.244445	149.087372
49.799999	148.167007	49.806187	0.160751	149.058075
49.820000	154.167007	49.826187	0.107958	149.028870
49.840000	159.167007	49.846188	0.073991	148.999664
49.860001	158.000000	49.866188	0.051751	148.970551
49.880001	147.333008	49.886189	0.036981	148.941437
49.900002	152.833008	49.906189	0.027080	148.912323
49.920002	147.667007	49.926189	0.020438	148.883270
49.940002	143.833008	49.946190	0.016051	148.854309
49.960003	137.667007	49.966190	0.013299	148.825348
49.980003	151.500000	49.986195	0.011821	148.796417
50.000000	146.167007	50.006191	0.011444	148.767517
50.020000	154.833008	50.026192	0.012150	148.738708
50.040001	158.667007	50.046192	0.014084	148.709869
50.060001	150.500000	50.066193	0.017582	148.681122
50.080002	153.667007	50.086193	0.023258	148.652374
50.099998	162.500000	50.106190	0.032147	148.623718
50.119999	153.667007	50.126190	0.045980	148.595062
50.139999	145.333008	50.146191	0.067659	148.566437
50.160000	145.833008	50.166191	0.102133	148.537872
50.180000	142.167007	50.186192	0.158023	148.509308
50.200001	150.333008	50.206192	0.250743	148.480835
50.220001	153.000000	50.226192	0.408684	148.452362
50.240002	148.167007	50.246193	0.685996	148.423920
50.260002	151.000000	50.266193	1.190159	148.395569
50.280003	152.167007	50.286194	2.143860	148.367218
50.299999	159.500000	50.306190	4.027319	148.338898
50.320000	159.833008	50.326191	7.912355	148.310638
50.340000	164.500000	50.346191	16.221004	148.282410
50.360001	185.333008	50.366192	34.369770	148.254242
50.380001	233.833008	50.386192	73.891403	148.226105
50.400002	302.166992	50.406193	156.709473	148.197968
50.420002	495.500000	50.426193	315.095093	148.169891
50.440002	718.166992	50.446194	569.190796	148.141876
50.460003	977.000000	50.466194	864.004211	148.113861
50.480003	1061.830078	50.486195	1030.932373	148.085938

50.500000	972.833008	50.506191	929.955444	148.058014
50.520000	761.166992	50.526192	643.254211	148.030121
50.540001	543.000000	50.546192	364.573425	148.002289
50.560001	349.000000	50.566193	185.009369	147.974518
50.580002	254.000000	50.586193	90.711037	147.946747
50.599998	200.667007	50.606190	44.927635	147.919037
50.619999	166.167007	50.626190	22.813530	147.891388
50.639999	156.833008	50.646191	11.878286	147.863739
50.660000	169.167007	50.666191	6.322945	147.836151
50.680000	158.167007	50.686192	3.438073	147.808594
50.700001	160.167007	50.706192	1.912249	147.781067
50.720001	155.167007	50.726196	1.090278	147.753571
50.740002	152.500000	50.746197	0.638608	147.726166
50.760002	151.333008	50.766197	0.384761	147.698761
50.780003	152.333008	50.786198	0.238803	147.671387
50.799999	141.500000	50.806194	0.153150	147.644104
50.820000	138.167007	50.826195	0.102181	147.616852
50.840000	148.500000	50.846195	0.071957	147.589630
50.860001	134.833008	50.866196	0.054853	147.562408
50.880001	135.333008	50.886196	0.046862	147.535278
50.900002	144.000000	50.906197	0.046273	147.508179
50.920002	149.500000	50.926197	0.053136	147.481110
50.940002	137.333008	50.946198	0.069324	147.454132
50.960003	139.667007	50.966198	0.099223	147.427124
50.980003	136.667007	50.986198	0.151373	147.400177
51.000000	140.000000	51.006195	0.241911	147.373291
51.020000	134.333008	51.026196	0.401853	147.346466
51.040001	138.667007	51.046196	0.692501	147.319641
51.060001	141.333008	51.066196	1.240035	147.292877
51.080002	149.333008	51.086201	2.315042	147.266174
51.099998	138.167007	51.106197	4.515346	147.239471
51.119999	156.667007	51.126198	9.192205	147.212860
51.139999	166.167007	51.146198	19.375675	147.186249
51.160000	202.167007	51.166199	41.602200	147.159698
51.180000	266.500000	51.186199	88.683563	147.133179
51.200001	388.832977	51.206200	180.960617	147.106720
51.220001	573.833008	51.226200	336.338501	147.080292
51.240002	743.833008	51.246201	534.736389	147.053925

51.260002	853.333008	51.266197	680.411255	147.027618
51.280003	818.833008	51.286198	661.284058	147.001312
51.299999	660.166992	51.306194	490.726593	146.975128
51.320000	505.500000	51.326195	293.045532	146.948883
51.340000	348.166992	51.346195	153.040329	146.922760
51.360001	249.833008	51.366196	75.679939	146.896606
51.380001	185.000000	51.386196	37.342808	146.870575
51.400002	178.333008	51.406197	18.799932	146.844543
51.420002	167.333008	51.426197	9.692853	146.818573
51.440002	157.333008	51.446201	5.107104	146.792633
51.460003	160.333008	51.466202	2.747664	146.766724
51.480003	152.833008	51.486202	1.510915	146.740906
51.500000	151.500000	51.506199	0.851200	146.715118
51.520000	156.500000	51.526199	0.492297	146.689392
51.540001	147.167007	51.546200	0.292877	146.663727
51.560001	143.000000	51.566200	0.179584	146.638062
51.580002	146.333008	51.586201	0.113905	146.612457
51.599998	141.167007	51.606197	0.075324	146.586884
51.619999	146.333008	51.626198	0.052739	146.561401
51.639999	142.833008	51.646198	0.040149	146.535919
51.660000	143.500000	51.666199	0.034408	146.510498
51.680000	138.500000	51.686199	0.034135	146.485138
51.700001	135.833008	51.706200	0.039258	146.459778
51.720001	134.167007	51.726200	0.050983	146.434509
51.740002	137.500000	51.746201	0.072192	146.409302
51.760002	137.500000	51.766201	0.108429	146.384094
51.780003	146.000000	51.786201	0.169955	146.359009
51.799999	147.167007	51.806202	0.275879	146.333923
51.820000	144.833008	51.826202	0.462710	146.308899
51.840000	149.833008	51.846203	0.802674	146.283905
51.860001	146.833008	51.866203	1.444443	146.258972
51.880001	147.000000	51.886204	2.706394	146.234100
51.900002	153.333008	51.906204	5.292851	146.209259
51.920002	161.333008	51.926205	10.789593	146.184448
51.940002	163.500000	51.946205	22.746977	146.159729
51.960003	185.833008	51.966206	48.802307	146.135040
51.980003	238.167007	51.986206	103.879112	146.110413
52.000000	341.832977	52.006203	211.595566	146.085815

52.020000	548.500000	52.026203	393.054993	146.061310
52.040001	750.000000	52.046204	626.772095	146.036835
52.060001	906.500000	52.066204	805.835754	146.012360
52.080002	899.333008	52.086205	799.896301	145.988007
52.099998	727.833008	52.106201	612.546204	145.963654
52.119999	513.666992	52.126202	378.808441	145.939392
52.139999	360.832977	52.146202	203.516281	145.915161
52.160000	249.833008	52.166206	102.186081	145.890991
52.180000	194.000000	52.186203	50.596119	145.866852
52.200001	174.167007	52.206203	25.375090	145.842804
52.220001	159.667007	52.226204	12.995611	145.818756
52.240002	148.833008	52.246204	6.796504	145.794800
52.260002	158.500000	52.266205	3.627289	145.770905
52.280003	147.333008	52.286205	1.977304	145.747040
52.299999	146.500000	52.306202	1.103196	145.723236
52.320000	139.667007	52.326202	0.631006	145.699493
52.340000	137.500000	52.346203	0.370428	145.675781
52.360001	143.500000	52.366203	0.223178	145.652130
52.380001	142.333008	52.386204	0.137882	145.628510
52.400002	142.833008	52.406204	0.087240	145.605011
52.420002	129.667007	52.426205	0.056452	145.581512
52.440002	135.333008	52.446205	0.037316	145.558075
52.460003	140.500000	52.466206	0.025180	145.534698
52.480003	127.167000	52.486206	0.017345	145.511383
52.500000	142.333008	52.506203	0.012214	145.488129
52.520000	135.000000	52.526207	0.008819	145.464905
52.540001	140.833008	52.546207	0.006571	145.441742
52.560001	150.500000	52.566208	0.005104	145.418640
52.580002	145.667007	52.586208	0.004192	145.395599
52.599998	136.833008	52.606205	0.003706	145.372620
52.619999	137.167007	52.626205	0.003577	145.349670
52.639999	142.833008	52.646206	0.003791	145.326813
52.660000	136.667007	52.666206	0.004384	145.303986
52.680000	131.500000	52.686207	0.005449	145.281219
52.700001	138.167007	52.706207	0.007157	145.258514
52.720001	132.833008	52.726208	0.009796	145.235870
52.740002	128.667007	52.746208	0.013839	145.213287
52.760002	139.167007	52.766209	0.020068	145.190704

52.780003	142.333008	52.786209	0.029788	145.168243
52.799999	147.833008	52.806206	0.045218	145.145844
52.820000	144.333008	52.826206	0.070242	145.123444
52.840000	151.333008	52.846207	0.111810	145.101135
52.860001	154.000000	52.866211	0.182795	145.078888
52.880001	159.500000	52.886211	0.307793	145.056732
52.900002	158.667007	52.906212	0.535752	145.034576
52.920002	172.833008	52.926212	0.967582	145.012512
52.940002	177.500000	52.946213	1.817857	144.990509
52.960003	185.500000	52.966213	3.550913	144.968536
52.980003	208.667007	52.986214	7.167534	144.946655
53.000000	226.833008	53.006210	14.749102	144.924805
53.020000	240.333008	53.026211	30.326429	144.903046
53.040001	270.000000	53.046211	60.871136	144.881348
53.060001	333.166992	53.066212	117.183907	144.859650
53.080002	424.000000	53.086212	214.961578	144.838074
53.099998	589.833008	53.106209	371.834106	144.816528
53.119999	801.833008	53.126205	580.955933	144.795074
53.139999	926.000000	53.146206	762.460693	144.773651
53.160000	939.500000	53.166206	788.845398	144.752289
53.180000	811.000000	53.186207	630.657043	144.730988
53.200001	618.666992	53.206207	402.809387	144.709778
53.220001	449.666992	53.226212	220.461823	144.688568
53.240002	314.332977	53.246212	111.624039	144.667480
53.260002	228.500000	53.266212	55.352886	144.646393
53.280003	188.167007	53.286213	27.695774	144.625427
53.299999	166.833008	53.306210	14.118648	144.604523
53.320000	155.167007	53.326210	7.337692	144.583649
53.340000	151.333008	53.346210	3.887441	144.562836
53.360001	152.667007	53.366211	2.101708	144.542084
53.380001	151.667007	53.386211	1.161949	144.521393
53.400002	142.667007	53.406212	0.658246	144.500793
53.420002	142.667007	53.426212	0.382563	144.480286
53.440002	146.833008	53.446213	0.228137	144.459747
53.460003	145.000000	53.466213	0.139490	144.439301
53.480003	141.667007	53.486214	0.087334	144.418976
53.500000	148.833008	53.506210	0.055908	144.398651
53.520000	147.667007	53.526211	0.036530	144.378448

53.540001	146.333008	53.546211	0.024327	144.358276
53.560001	161.833008	53.566216	0.016486	144.338165
53.580002	164.333008	53.586216	0.011357	144.318085
53.599998	159.333008	53.606213	0.007943	144.298126
53.619999	157.333008	53.626213	0.005633	144.278198
53.639999	153.833008	53.646214	0.004048	144.258331
53.660000	153.833008	53.666214	0.002945	144.238556
53.680000	146.667007	53.686214	0.002167	144.218842
53.700001	137.667007	53.706215	0.001613	144.199158
53.720001	138.833008	53.726215	0.001213	144.179565
53.740002	144.833008	53.746216	0.000922	144.160034
53.760002	139.167007	53.766216	0.000708	144.140564
53.780003	140.833008	53.786217	0.000550	144.121155
53.799999	134.833008	53.806213	0.000433	144.101837
53.820000	143.833008	53.826214	0.000347	144.082550
53.840000	136.167007	53.846214	0.000283	144.063324
53.860001	138.167007	53.866215	0.000238	144.044189
53.880001	140.333008	53.886215	0.000208	144.025116
53.900002	140.167007	53.906219	0.000192	144.006134
53.920002	131.500000	53.926220	0.000189	143.987152
53.940002	132.833008	53.946220	0.000201	143.968292
53.960003	127.500000	53.966221	0.000231	143.949463
53.980003	128.000000	53.986221	0.000285	143.930695
54.000000	129.833008	54.006218	0.000374	143.912048
54.020000	127.833000	54.026218	0.000516	143.893402
54.040001	134.500000	54.046219	0.000739	143.874878
54.060001	138.500000	54.066219	0.001092	143.856415
54.080002	129.833008	54.086216	0.001659	143.837982
54.099998	136.000000	54.106213	0.002585	143.819641
54.119999	131.167007	54.126213	0.004130	143.801361
54.139999	128.500000	54.146214	0.006769	143.783142
54.160000	131.333008	54.166214	0.011404	143.764984
54.180000	125.667000	54.186214	0.019797	143.746918
54.200001	134.000000	54.206215	0.035529	143.728882
54.220001	132.500000	54.226215	0.066154	143.710907
54.240002	135.333008	54.246216	0.128159	143.693054
54.260002	138.500000	54.266220	0.258357	143.675262
54.280003	134.333008	54.286221	0.539042	143.657501

54.299999	138.833008	54.306217	1.149715	143.639801
54.320000	139.833008	54.326218	2.455883	143.622223
54.340000	149.167007	54.346218	5.095883	143.604645
54.360001	168.000000	54.366219	9.855412	143.587189
54.380001	173.667007	54.386219	16.852926	143.569794
54.400002	187.333008	54.406219	24.009581	143.552460
54.420002	185.667007	54.426220	27.053768	143.535156
54.440002	184.833008	54.446220	23.566286	143.517975
54.460003	175.500000	54.466221	16.213827	143.500854
54.480003	160.667007	54.486221	9.354544	143.483765
54.500000	141.833008	54.506218	4.874150	143.466766
54.520000	144.333008	54.526218	2.439935	143.449829
54.540001	139.000000	54.546219	1.218304	143.432953
54.560001	143.167007	54.566219	0.616298	143.416168
54.580002	139.833008	54.586220	0.317093	143.399475
54.599998	132.167007	54.606220	0.166052	143.382782
54.619999	134.000000	54.626221	0.088608	143.366180
54.639999	128.167007	54.646221	0.048282	143.349701
54.660000	138.667007	54.666222	0.026926	143.333221
54.680000	138.167007	54.686222	0.015394	143.316833
54.700001	135.333008	54.706223	0.009028	143.300507
54.720001	129.500000	54.726223	0.005429	143.284271
54.740002	129.167007	54.746223	0.003345	143.268097
54.760002	131.667007	54.766224	0.002108	143.251984
54.780003	138.500000	54.786224	0.001358	143.235931
54.799999	140.167007	54.806221	0.000892	143.219971
54.820000	130.500000	54.826221	0.000597	143.204071
54.840000	129.333008	54.846222	0.000407	143.188232
54.860001	132.333008	54.866222	0.000282	143.172485
54.880001	135.500000	54.886223	0.000199	143.156769
54.900002	136.667007	54.906223	0.000142	143.141144
54.920002	140.833008	54.926224	0.000104	143.125580
54.940002	141.000000	54.946228	0.000078	143.110077
54.960003	148.833008	54.966228	0.000059	143.094666
54.980003	144.000000	54.986229	0.000047	143.079315
55.000000	139.000000	55.006226	0.000039	143.064026
55.020000	145.333008	55.026226	0.000033	143.048798
55.040001	139.500000	55.046227	0.000030	143.033661

55.060001	141.500000	55.066223	0.000029	143.018585
55.080002	139.167007	55.086224	0.000030	143.003571
55.099998	136.000000	55.106220	0.000033	142.988617
55.119999	135.833008	55.126221	0.000037	142.973755
55.139999	137.667007	55.146221	0.000044	142.958954
55.160000	134.833008	55.166222	0.000053	142.944214
55.180000	136.500000	55.186222	0.000066	142.929535
55.200001	140.167007	55.206223	0.000083	142.914917
55.220001	130.167007	55.226223	0.000106	142.900391
55.240002	120.500000	55.246223	0.000136	142.885956
55.260002	127.333000	55.266224	0.000178	142.871552
55.280003	135.000000	55.286228	0.000235	142.857208
55.299999	131.333008	55.306225	0.000313	142.842987
55.320000	128.500000	55.326225	0.000422	142.828796
55.340000	129.167007	55.346226	0.000573	142.814667
55.360001	135.833008	55.366226	0.000789	142.800598
55.380001	132.667007	55.386227	0.001098	142.786621
55.400002	131.833008	55.406227	0.001547	142.772736
55.420002	133.000000	55.426228	0.002209	142.758850
55.440002	125.500000	55.446228	0.003199	142.745087
55.460003	134.833008	55.466228	0.004704	142.731384
55.480003	132.000000	55.486229	0.007031	142.717712
55.500000	128.833008	55.506226	0.010696	142.704163
55.520000	137.667007	55.526226	0.016585	142.690643
55.540001	139.333008	55.546227	0.026251	142.677185
55.560001	128.333008	55.566227	0.042485	142.663849
55.580002	134.500000	55.586227	0.070448	142.650543
55.599998	132.833008	55.606228	0.119963	142.637268
55.619999	131.667007	55.626228	0.210384	142.624115
55.639999	134.333008	55.646229	0.381234	142.611023
55.660000	138.000000	55.666229	0.716192	142.597992
55.680000	137.000000	55.686230	1.397989	142.584991
55.700001	136.167007	55.706230	2.833379	142.572113
55.720001	144.000000	55.726231	5.927480	142.559265
55.740002	145.000000	55.746231	12.634618	142.546509
55.760002	167.500000	55.766232	26.860741	142.533813
55.780003	205.500000	55.786232	55.231861	142.521149
55.799999	279.500000	55.806229	105.392929	142.508606

55.820000	364.166992	55.826229	177.304916	142.496124
55.840000	455.666992	55.846230	248.509674	142.483704
55.860001	482.332977	55.866230	276.730042	142.471313
55.880001	454.832977	55.886230	240.212479	142.459015
55.900002	365.332977	55.906231	166.154968	142.446777
55.920002	274.332977	55.926231	96.930916	142.434601
55.940002	220.167007	55.946236	51.101986	142.422516
55.960003	184.333008	55.966236	25.812277	142.410461
55.980003	156.500000	55.986237	12.945896	142.398529
56.000000	146.667007	56.006233	6.551993	142.386597
56.020000	139.833008	56.026234	3.363040	142.374786
56.040001	137.167007	56.046234	1.754649	142.363007
56.060001	142.667007	56.066231	0.933345	142.351288
56.080002	136.333008	56.086231	0.508921	142.339630
56.099998	142.833008	56.106228	0.288165	142.328033
56.119999	137.833008	56.126228	0.175088	142.316528
56.139999	147.333008	56.146229	0.124007	142.305084
56.160000	138.000000	56.166229	0.117977	142.293732
56.180000	140.833008	56.186230	0.162709	142.282379
56.200001	143.833008	56.206230	0.293064	142.271149
56.220001	148.500000	56.226231	0.593899	142.259949
56.240002	143.667007	56.246231	1.233250	142.248840
56.260002	138.000000	56.266232	2.475237	142.237762
56.280003	138.667007	56.286236	4.560011	142.226776
56.299999	144.667007	56.306232	7.292463	142.215790
56.320000	140.167007	56.326233	9.581644	142.204956
56.340000	144.333008	56.346233	9.931907	142.194153
56.360001	135.000000	56.366234	8.060751	142.183411
56.380001	138.333008	56.386234	5.293758	142.172699
56.400002	137.333008	56.406235	2.992513	142.162079
56.420002	133.000000	56.426235	1.556868	142.151550
56.440002	131.167007	56.446236	0.784874	142.141052
56.460003	134.167007	56.466236	0.394829	142.130615
56.480003	134.500000	56.486237	0.200658	142.120239
56.500000	136.333008	56.506233	0.103500	142.109894
56.520000	137.000000	56.526234	0.054343	142.099701
56.540001	136.667007	56.546234	0.029209	142.089478
56.560001	137.333008	56.566235	0.016256	142.079346

56.580002	131.333008	56.586235	0.009599	142.069305
56.599998	135.833008	56.606236	0.006328	142.059296
56.619999	138.167007	56.626236	0.005064	142.049347
56.639999	143.000000	56.646236	0.005290	142.039459
56.660000	140.833008	56.666237	0.007116	142.029633
56.680000	144.500000	56.686237	0.011377	142.019867
56.700001	139.333008	56.706238	0.020179	142.010162
56.720001	130.500000	56.726238	0.038286	142.000519
56.740002	136.000000	56.746239	0.076327	141.990936
56.760002	128.667007	56.766239	0.157684	141.981445
56.780003	130.333008	56.786240	0.331679	141.971924
56.799999	134.500000	56.806236	0.692772	141.962555
56.820000	132.167007	56.826237	1.389279	141.953186
56.840000	134.167007	56.846237	2.560208	141.943909
56.860001	137.667007	56.866238	4.117485	141.934692
56.880001	140.000000	56.886238	5.491969	141.925507
56.900002	144.833008	56.906239	5.890643	141.916382
56.920002	140.833008	56.926239	5.194211	141.907318
56.940002	140.333008	56.946243	4.275588	141.898315
56.960003	138.833008	56.966244	4.284655	141.889374
56.980003	151.333008	56.986244	6.300282	141.880493
57.000000	141.333008	57.006241	11.921288	141.871674
57.020000	160.333008	57.026241	24.055616	141.862885
57.040001	203.333008	57.046242	46.763428	141.854156
57.060001	254.667007	57.066242	82.137794	141.845490
57.080002	312.500000	57.086243	123.041153	141.836884
57.099998	368.666992	57.106236	149.354797	141.828339
57.119999	357.666992	57.126236	142.613098	141.819855
57.139999	286.000000	57.146236	107.773560	141.811401
57.160000	248.667007	57.166237	67.309410	141.803009
57.180000	202.833008	57.186237	37.039360	141.794678
57.200001	169.500000	57.206238	19.098612	141.786407
57.220001	150.667007	57.226238	9.634869	141.778198
57.240002	130.833008	57.246239	4.864501	141.769989
57.260002	130.500000	57.266243	2.480173	141.761871
57.280003	136.333008	57.286243	1.281691	141.753784
57.299999	132.000000	57.306240	0.672895	141.745758
57.320000	131.833008	57.326241	0.359746	141.737823

57.340000	132.167007	57.346241	0.196406	141.729919
57.360001	131.833008	57.366241	0.109744	141.722046
57.380001	147.333008	57.386242	0.062842	141.714264
57.400002	143.333008	57.406242	0.036892	141.706482
57.420002	140.333008	57.426243	0.022195	141.698792
57.440002	144.167007	57.446243	0.013676	141.691132
57.460003	142.500000	57.466244	0.008626	141.683533
57.480003	136.167007	57.486244	0.005570	141.675995
57.500000	138.500000	57.506241	0.003690	141.668488
57.520000	135.500000	57.526241	0.002519	141.661041
57.540001	137.667007	57.546242	0.001792	141.653625
57.560001	140.500000	57.566242	0.001352	141.646271
57.580002	139.167007	57.586246	0.001114	141.638977
57.599998	133.833008	57.606243	0.001034	141.631683
57.619999	141.167007	57.626244	0.001099	141.624481
57.639999	138.833008	57.646244	0.001330	141.617310
57.660000	131.000000	57.666245	0.001781	141.610229
57.680000	131.333008	57.686245	0.002563	141.603149
57.700001	129.000000	57.706245	0.003874	141.596130
57.720001	139.500000	57.726246	0.006076	141.589142
57.740002	135.333008	57.746246	0.009821	141.582245
57.760002	128.167007	57.766247	0.016327	141.575348
57.780003	137.500000	57.786247	0.027925	141.568481
57.799999	143.167007	57.806244	0.049206	141.561707
57.820000	121.833000	57.826244	0.089568	141.554962
57.840000	138.667007	57.846245	0.168834	141.548248
57.860001	128.333008	57.866245	0.330073	141.541626
57.880001	132.833008	57.886246	0.668375	141.535004
57.900002	133.167007	57.906250	1.393282	141.528412
57.920002	134.500000	57.926250	2.950520	141.521881
57.940002	144.500000	57.946251	6.216322	141.515411
57.960003	159.667007	57.966251	12.646188	141.508942
57.980003	182.333008	57.986252	23.887764	141.502533
58.000000	216.500000	58.006248	39.983330	141.496185
58.020000	219.500000	58.026249	56.606266	141.489899
58.040001	229.833008	58.046249	65.767296	141.483612
58.060001	234.167007	58.066250	63.288033	141.477386
58.080002	206.167007	58.086250	53.340168	141.471191

58.099998	192.833008	58.106247	41.823181	141.465027
58.119999	174.167007	58.126247	30.656919	141.458923
58.139999	157.667007	58.146248	20.448061	141.452850
58.160000	149.667007	58.166245	12.625771	141.446838
58.180000	151.167007	58.186245	8.124770	141.440796
58.200001	143.333008	58.206245	6.937233	141.434845
58.220001	149.333008	58.226250	8.919828	141.428955
58.240002	162.833008	58.246250	13.994714	141.423065
58.260002	171.167007	58.266251	21.108618	141.417236
58.280003	168.500000	58.286251	27.032343	141.411407
58.299999	171.833008	58.306248	27.772089	141.405609
58.320000	172.667007	58.326248	22.639168	141.399902
58.340000	169.667007	58.346249	15.080091	141.394196
58.360001	153.167007	58.366249	8.683792	141.388550
58.380001	139.333008	58.386250	4.596676	141.382935
58.400002	136.500000	58.406250	2.346922	141.377319
58.420002	138.500000	58.426250	1.189518	141.371796
58.440002	129.833008	58.446251	0.607291	141.366241
58.460003	130.000000	58.466251	0.315512	141.360779
58.480003	132.500000	58.486252	0.169871	141.355316
58.500000	134.500000	58.506248	0.099395	141.349884
58.520000	135.167007	58.526249	0.070488	141.344513
58.540001	133.667007	58.546253	0.070068	141.339142
58.560001	138.000000	58.566254	0.099516	141.333801
58.580002	127.500000	58.586254	0.177065	141.328552
58.599998	127.333000	58.606251	0.350780	141.323242
58.619999	133.000000	58.626251	0.729734	141.318024
58.639999	140.167007	58.646252	1.543252	141.312836
58.660000	137.333008	58.666252	3.223984	141.307678
58.680000	134.833008	58.686253	6.429904	141.302521
58.700001	149.000000	58.706253	11.732948	141.297424
58.720001	147.333008	58.726254	18.638830	141.292328
58.740002	166.500000	58.746254	24.522533	141.287262
58.760002	167.667007	58.766254	25.763393	141.282227
58.780003	164.000000	58.786255	21.449686	141.277252
58.799999	157.333008	58.806252	14.554587	141.272247
58.820000	143.167007	58.826252	8.497995	141.267334
58.840000	140.000000	58.846252	4.537209	141.262421

58.860001	129.000000	58.866257	2.325502	141.257538
58.880001	145.167007	58.886257	1.178869	141.252655
58.900002	138.833008	58.906258	0.599436	141.247803
58.920002	132.000000	58.926258	0.307617	141.243011
58.940002	127.333000	58.946259	0.159870	141.238220
58.960003	140.833008	58.966259	0.084403	141.233459
58.980003	136.833008	58.986259	0.045406	141.228699
59.000000	134.333008	59.006256	0.024960	141.223999
59.020000	132.500000	59.026257	0.014043	141.219299
59.040001	137.000000	59.046257	0.008096	141.214630
59.060001	139.167007	59.066257	0.004782	141.209961
59.080002	139.500000	59.086258	0.002893	141.205353
59.099998	139.500000	59.106255	0.001791	141.200745
59.119999	144.333008	59.126255	0.001134	141.196136
59.139999	142.167007	59.146255	0.000735	141.191620
59.160000	142.167007	59.166256	0.000487	141.187042
59.180000	148.000000	59.186260	0.000332	141.182495
59.200001	144.667007	59.206261	0.000234	141.178009
59.220001	130.667007	59.226261	0.000172	141.173523
59.240002	139.667007	59.246262	0.000134	141.169067
59.260002	133.667007	59.266258	0.000113	141.164612
59.280003	132.333008	59.286259	0.000105	141.160187
59.299999	132.167007	59.306255	0.000107	141.155762
59.320000	141.333008	59.326256	0.000119	141.151367
59.340000	144.500000	59.346256	0.000142	141.147003
59.360001	136.333008	59.366257	0.000179	141.142639
59.380001	141.667007	59.386257	0.000233	141.138275
59.400002	143.500000	59.406258	0.000312	141.133942
59.420002	148.167007	59.426258	0.000427	141.129639
59.440002	149.667007	59.446259	0.000593	141.125305
59.460003	145.833008	59.466259	0.000836	141.121033
59.480003	157.833008	59.486259	0.001195	141.116760
59.500000	156.333008	59.506260	0.001735	141.112488
59.520000	159.833008	59.526260	0.002556	141.108246
59.540001	156.500000	59.546261	0.003825	141.104034
59.560001	156.167007	59.566261	0.005820	141.099792
59.580002	155.833008	59.586262	0.009016	141.095581
59.599998	154.167007	59.606258	0.014234	141.091370

59.619999	153.167007	59.626259	0.022940	141.087158
59.639999	150.500000	59.646259	0.037793	141.083038
59.660000	147.500000	59.666260	0.063764	141.078827
59.680000	146.000000	59.686260	0.110399	141.074677
59.700001	147.667007	59.706261	0.196611	141.070526
59.720001	149.500000	59.726261	0.361102	141.066406
59.740002	152.833008	59.746262	0.685583	141.062256
59.760002	145.500000	59.766262	1.347093	141.058136
59.780003	150.833008	59.786263	2.734835	141.054016
59.799999	153.833008	59.806259	5.699699	141.049896
59.820000	163.167007	59.826263	12.045040	141.045807
59.840000	176.333008	59.846264	25.277336	141.041687
59.860001	207.167007	59.866264	51.174919	141.037598
59.880001	271.166992	59.886265	96.210983	141.033539
59.900002	325.666992	59.906265	160.407272	141.029449
59.920002	376.000000	59.926266	225.839478	141.025360
59.940002	396.666992	59.946266	257.754944	141.021301
59.960003	368.000000	59.966267	234.187027	141.017242
59.980003	336.500000	59.986267	171.883881	141.013184
60.000000	277.166992	60.006264	106.550835	141.009094
60.020000	239.667007	60.026264	59.105793	141.005066
60.040001	200.167007	60.046265	30.919285	141.001007
60.060001	172.000000	60.066265	15.808219	140.996948
60.080002	154.833008	60.086266	8.056299	140.992920
60.099998	145.000000	60.106262	4.137212	140.988861
60.119999	144.833008	60.126266	2.162644	140.984772
60.139999	141.167007	60.146267	1.176464	140.980743
60.160000	143.167007	60.166267	0.704228	140.976715
60.180000	138.333008	60.186268	0.523168	140.972626
60.200001	149.333008	60.206268	0.552553	140.968567
60.220001	143.167007	60.226269	0.818573	140.964508
60.240002	142.167007	60.246269	1.480853	140.960480
60.260002	149.667007	60.266270	2.942743	140.956421
60.280003	149.500000	60.286270	6.106738	140.952362
60.299999	170.333008	60.306267	12.858109	140.948303
60.320000	189.667007	60.326267	26.753420	140.944214
60.340000	222.000000	60.346268	53.246731	140.940155
60.360001	277.000000	60.366268	97.366943	140.936066

60.380001	304.166992	60.386265	156.067795	140.932007
60.400002	336.166992	60.406265	209.169464	140.927948
60.420002	354.000000	60.426266	226.116486	140.923859
60.440002	325.332977	60.446270	195.102570	140.919739
60.460003	283.832977	60.466270	137.399582	140.915649
60.480003	234.333008	60.486271	82.900078	140.911530
60.500000	197.167007	60.506268	45.382103	140.907410
60.520000	168.333008	60.526268	23.660883	140.903320
60.540001	152.167007	60.546268	12.152855	140.899170
60.560001	143.500000	60.566269	6.319636	140.895020
60.580002	139.500000	60.586269	3.496009	140.890869
60.599998	140.000000	60.606266	2.324870	140.886749
60.619999	138.500000	60.626266	2.173168	140.882568
60.639999	134.000000	60.646267	2.702711	140.878387
60.660000	133.500000	60.666267	3.519885	140.874207
60.680000	135.167007	60.686268	4.038227	140.870026
60.700001	137.500000	60.706268	3.797617	140.865814
60.720001	130.833008	60.726269	2.903999	140.861603
60.740002	132.500000	60.746273	1.866216	140.857361
60.760002	135.000000	60.766273	1.063365	140.853119
60.780003	143.000000	60.786274	0.566258	140.848846
60.799999	127.667000	60.806271	0.292892	140.844604
60.820000	135.333008	60.826271	0.150412	140.840332
60.840000	142.167007	60.846272	0.077567	140.836029
60.860001	140.500000	60.866272	0.040422	140.831696
60.880001	143.500000	60.886272	0.021393	140.827393
60.900002	139.167007	60.906273	0.011554	140.823029
60.920002	148.000000	60.926273	0.006400	140.818695
60.940002	142.833008	60.946274	0.003656	140.814331
60.960003	143.333008	60.966274	0.002170	140.809967
60.980003	132.167007	60.986275	0.001352	140.805542
61.000000	125.333000	61.006271	0.000902	140.801117
61.020000	127.833000	61.026272	0.000660	140.796692
61.040001	137.167007	61.046272	0.000549	140.792267
61.060001	139.500000	61.066277	0.000527	140.787811
61.080002	137.833008	61.086277	0.000581	140.783295
61.099998	138.500000	61.106274	0.000712	140.778809
61.119999	126.000000	61.126274	0.000939	140.774292

61.139999	133.333008	61.146275	0.001301	140.769745
61.160000	134.167007	61.166275	0.001860	140.765167
61.180000	124.833000	61.186275	0.002726	140.760590
61.200001	123.667000	61.206276	0.004080	140.755981
61.220001	129.500000	61.226276	0.006227	140.751343
61.240002	135.333008	61.246277	0.009691	140.746735
61.260002	133.833008	61.266277	0.015392	140.742065
61.280003	138.833008	61.286278	0.024976	140.737396
61.299999	131.000000	61.306274	0.041469	140.732635
61.320000	135.833008	61.326275	0.070592	140.727936
61.340000	130.667007	61.346275	0.123455	140.723175
61.360001	135.167007	61.366280	0.222364	140.718384
61.380001	134.667007	61.386280	0.413348	140.713593
61.400002	145.333008	61.406281	0.794213	140.708771
61.420002	138.500000	61.426281	1.576236	140.703949
61.440002	140.333008	61.446281	3.216473	140.699036
61.460003	145.833008	61.466282	6.679872	140.694122
61.480003	163.167007	61.486282	13.877687	140.689240
61.500000	185.167007	61.506279	28.135725	140.684265
61.520000	223.833008	61.526279	53.964310	140.679291
61.540001	255.833008	61.546280	94.590012	140.674316
61.560001	326.166992	61.566277	147.213562	140.669281
61.580002	372.332977	61.586277	201.714386	140.664215
61.599998	438.166992	61.606274	248.231018	140.659149
61.619999	476.832977	61.626274	281.533264	140.654022
61.639999	518.666992	61.646275	290.775482	140.648865
61.660000	464.000000	61.666279	261.415802	140.643738
61.680000	406.332977	61.686279	198.625458	140.638519
61.700001	319.832977	61.706280	128.913605	140.633331
61.720001	259.666992	61.726280	74.383408	140.628052
61.740002	207.000000	61.746281	39.977535	140.622803
61.760002	200.167007	61.766281	20.762497	140.617462
61.780003	166.333008	61.786282	10.655458	140.612091
61.799999	161.833008	61.806278	5.468026	140.606750
61.820000	146.667007	61.826279	2.823169	140.601349
61.840000	139.667007	61.846279	1.473439	140.595947
61.860001	136.667007	61.866280	0.780450	140.590454
61.880001	142.167007	61.886280	0.421007	140.584961

61.900002	136.000000	61.906281	0.231925	140.579468
61.920002	147.500000	61.926281	0.130701	140.573883
61.940002	141.833008	61.946281	0.075407	140.568298
61.960003	135.500000	61.966286	0.044530	140.562653
61.980003	134.667007	61.986286	0.026902	140.557007
62.000000	132.167007	62.006283	0.016610	140.551331
62.020000	136.167007	62.026283	0.010466	140.545624
62.040001	129.667007	62.046284	0.006722	140.539825
62.060001	135.500000	62.066284	0.004396	140.534058
62.080002	133.667007	62.086285	0.002924	140.528229
62.099998	133.833008	62.106281	0.001977	140.522339
62.119999	131.500000	62.126282	0.001357	140.516479
62.139999	133.833008	62.146282	0.000946	140.510529
62.160000	141.667007	62.166283	0.000670	140.504578
62.180000	140.167007	62.186283	0.000483	140.498566
62.200001	134.667007	62.206284	0.000355	140.492523
62.220001	133.333008	62.226284	0.000267	140.486481
62.240002	133.333008	62.246284	0.000208	140.480347
62.260002	138.333008	62.266289	0.000169	140.474213
62.280003	137.667007	62.286289	0.000145	140.468018
62.299999	134.833008	62.306286	0.000134	140.461792
62.320000	137.833008	62.326286	0.000134	140.455536
62.340000	132.167007	62.346287	0.000146	140.449219
62.360001	136.167007	62.366287	0.000170	140.442902
62.380001	137.333008	62.386288	0.000209	140.436523
62.400002	132.833008	62.406288	0.000269	140.430115
62.420002	135.000000	62.426289	0.000357	140.423645
62.440002	137.667007	62.446289	0.000485	140.417206
62.460003	133.167007	62.466290	0.000670	140.410645
62.480003	136.500000	62.486290	0.000943	140.404053
62.500000	138.667007	62.506287	0.001345	140.397461
62.520000	133.333008	62.526287	0.001948	140.390808
62.540001	132.167007	62.546288	0.002863	140.384125
62.560001	139.167007	62.566288	0.004270	140.377411
62.580002	133.500000	62.586292	0.006472	140.370636
62.599998	132.833008	62.606289	0.009974	140.363831
62.619999	128.333008	62.626289	0.015648	140.356964
62.639999	133.167007	62.646290	0.025022	140.350098

62.660000	139.833008	62.666290	0.040834	140.343140
62.680000	137.000000	62.686291	0.068105	140.336182
62.700001	140.000000	62.706291	0.116275	140.329163
62.720001	131.833008	62.726292	0.203585	140.322083
62.740002	146.500000	62.746292	0.366301	140.315002
62.760002	139.333008	62.766293	0.678693	140.307861
62.780003	134.500000	62.786289	1.296983	140.300690
62.799999	141.000000	62.806286	2.557524	140.293427
62.820000	146.333008	62.826286	5.191944	140.286194
62.840000	159.000000	62.846287	10.774312	140.278870
62.860001	177.500000	62.866287	22.558929	140.271515
62.880001	209.167007	62.886292	46.699280	140.264099
62.900002	291.500000	62.906292	92.871353	140.256653
62.920002	397.000000	62.926292	171.099670	140.249207
62.940002	529.666992	62.946293	279.785614	140.241608
62.960003	659.833008	62.966293	388.603638	140.234039
62.980003	720.500000	62.986294	442.433411	140.226440
63.000000	674.166992	63.006290	406.700958	140.218781
63.020000	555.833008	63.026291	305.844086	140.211090
63.040001	420.166992	63.046291	195.618759	140.203308
63.060001	312.332977	63.066292	111.998779	140.195496
63.080002	254.500000	63.086292	60.160900	140.187653
63.099998	219.167007	63.106289	31.344482	140.179749
63.119999	187.333008	63.126289	16.144951	140.171844
63.139999	166.667007	63.146290	8.313643	140.163879
63.160000	159.667007	63.166294	4.318464	140.155823
63.180000	146.667007	63.186295	2.297125	140.147766
63.200001	153.333008	63.206295	1.298594	140.139648
63.220001	153.167007	63.226295	0.859860	140.131470
63.240002	139.000000	63.246296	0.790738	140.123260
63.260002	142.500000	63.266296	1.091221	140.115021
63.280003	147.000000	63.286297	1.967291	140.106689
63.299999	152.667007	63.306293	3.942999	140.098358
63.320000	164.500000	63.326294	8.055669	140.089966
63.340000	164.167007	63.346294	15.964388	140.081512
63.360001	170.167007	63.366295	29.425213	140.073029
63.380001	197.833008	63.386295	48.279587	140.064484
63.400002	217.833008	63.406296	67.484917	140.055908

63.420002	223.833008	63.426296	77.546860	140.047272
63.440002	214.167007	63.446297	72.092941	140.038605
63.460003	206.333008	63.466301	54.856464	140.029877
63.480003	187.167007	63.486301	35.464508	140.021118
63.500000	175.167007	63.506298	20.481953	140.012268
63.520000	159.333008	63.526299	11.071104	140.003387
63.540001	161.333008	63.546299	5.793073	139.994507
63.560001	143.500000	63.566299	2.994879	139.985504
63.580002	141.500000	63.586300	1.549977	139.976532
63.599998	141.833008	63.606297	0.814828	139.967468
63.619999	133.500000	63.626297	0.447629	139.958374
63.639999	136.000000	63.646297	0.274747	139.949219
63.660000	138.833008	63.666298	0.213040	139.940002
63.680000	133.500000	63.686298	0.231217	139.930756
63.700001	133.000000	63.706299	0.335160	139.921448
63.720001	139.500000	63.726299	0.572475	139.912109
63.740002	142.167007	63.746300	1.061525	139.902740
63.760002	138.333008	63.766304	2.065644	139.893250
63.780003	149.333008	63.786304	4.158877	139.883789
63.799999	152.667007	63.806301	8.576517	139.874237
63.820000	161.333008	63.826302	17.882153	139.864685
63.840000	184.000000	63.846302	36.963619	139.855011
63.860001	228.500000	63.866302	73.728088	139.845337
63.880001	326.500000	63.886303	137.057053	139.835632
63.900002	433.000000	63.906303	227.909454	139.825806
63.920002	517.833008	63.926304	324.805725	139.816010
63.940002	599.666992	63.946304	382.743835	139.806152
63.960003	570.833008	63.966305	366.597412	139.796173
63.980003	492.166992	63.986305	288.976105	139.786224
64.000000	418.000000	64.006302	197.047699	139.776215
64.019997	324.000000	64.026299	128.987381	139.766113
64.040001	290.832977	64.046303	98.562180	139.756012
64.059998	296.666992	64.066299	107.559700	139.745850
64.080002	333.666992	64.086304	155.981094	139.735626
64.099998	405.000000	64.106300	237.235413	139.725372
64.119995	505.166992	64.126297	324.593842	139.715057
64.139999	546.833008	64.146301	371.942169	139.704712
64.159996	517.166992	64.166298	347.549255	139.694275

64.180000	450.500000	64.186302	266.831757	139.683838
64.199997	359.500000	64.206299	174.288376	139.673340
64.219994	271.166992	64.226295	101.621384	139.662781
64.239998	216.667007	64.246300	55.347496	139.652161
64.259995	186.667007	64.266296	29.118195	139.641510
64.279999	174.333008	64.286301	15.084396	139.630798
64.300003	152.667007	64.306305	7.781138	139.620056
64.320000	145.833008	64.326302	4.025280	139.609283
64.340004	139.000000	64.346306	2.098422	139.598450
64.360001	142.333008	64.366302	1.108098	139.587555
64.380005	134.833008	64.386307	0.594811	139.576599
64.400002	139.500000	64.406303	0.325733	139.565582
64.419998	134.500000	64.426300	0.182336	139.554565
64.440002	135.833008	64.446304	0.104457	139.543518
64.459999	140.667007	64.466301	0.061338	139.532379
64.480003	130.500000	64.486305	0.036970	139.521179
64.500000	138.333008	64.506310	0.022971	139.509979
64.519997	141.000000	64.526306	0.014860	139.498688
64.540001	134.333008	64.546310	0.010207	139.487366
64.559998	136.667007	64.566307	0.007718	139.476013
64.580002	140.000000	64.586311	0.006728	139.464569
64.599998	134.167007	64.606308	0.006987	139.453156
64.619995	136.000000	64.626305	0.008563	139.441650
64.639999	135.833008	64.646309	0.011876	139.430084
64.659996	140.333008	64.666306	0.017830	139.418488
64.680000	136.833008	64.686310	0.028157	139.406830
64.699997	142.833008	64.706299	0.046043	139.395172
64.719994	134.167007	64.726295	0.077493	139.383453
64.739998	132.667007	64.746300	0.133963	139.371643
64.759995	137.833008	64.766296	0.237777	139.359802
64.779999	147.333008	64.786301	0.433999	139.347931
64.800003	140.833008	64.806305	0.815524	139.335999
64.820000	139.500000	64.826302	1.578498	139.324066
64.840004	139.167007	64.846306	3.145623	139.312012
64.860001	150.500000	64.866302	6.423061	139.299957
64.880005	171.500000	64.886307	13.322237	139.287872
64.900002	183.833008	64.906303	27.617531	139.275726
64.919998	219.500000	64.926300	55.938694	139.263519

64.940002	298.500000	64.946304	107.390442	139.251282
64.959999	387.000000	64.966301	188.120071	139.239014
64.980003	464.166992	64.986305	288.582397	139.226654
65.000000	529.833008	65.006302	372.676300	139.214325
65.019997	539.333008	65.026299	394.465149	139.201904
65.040001	516.333008	65.046303	340.576324	139.189453
65.059998	437.832977	65.066299	245.185989	139.176971
65.080002	360.332977	65.086311	153.646286	139.164398
65.099998	272.166992	65.106308	88.635353	139.151825
65.119995	243.000000	65.126305	50.448895	139.139160
65.139999	209.500000	65.146309	32.037643	139.126495
65.159996	195.500000	65.166306	27.777517	139.113770
65.180000	192.667007	65.186310	35.146118	139.101013
65.199997	219.500000	65.206306	52.654068	139.088226
65.219994	223.000000	65.226303	75.740227	139.075348
65.239998	239.833008	65.246307	94.130920	139.062500
65.259995	232.500000	65.266304	96.624413	139.049561
65.279999	223.333008	65.286308	81.309532	139.036591
65.300003	193.667007	65.306313	57.359047	139.023590
65.320000	179.500000	65.326309	35.385906	139.010559
65.340004	157.500000	65.346313	19.993559	138.997467
65.360001	151.833008	65.366310	10.751705	138.984314
65.380005	146.167007	65.386314	5.638299	138.971130
65.400002	132.000000	65.406311	2.926165	138.957947
65.419998	138.667007	65.426308	1.515433	138.944672
65.440002	137.333008	65.446312	0.787883	138.931396
65.459999	144.167007	65.466309	0.413612	138.918060
65.480003	136.000000	65.486313	0.220151	138.904694
65.500000	133.333008	65.506310	0.119311	138.891296
65.519997	137.667007	65.526306	0.065998	138.877838
65.540001	138.167007	65.546310	0.037315	138.864319
65.559998	135.333008	65.566307	0.021588	138.850800
65.580002	126.000000	65.586311	0.012773	138.837280
65.599998	129.500000	65.606308	0.007728	138.823669
65.619995	138.333008	65.626305	0.004776	138.810028
65.639999	127.500000	65.646309	0.003013	138.796356
65.659996	137.667007	65.666313	0.001939	138.782623
65.680000	140.833008	65.686317	0.001273	138.768890

65.699997	136.000000	65.706314	0.000853	138.755096
65.719994	135.333008	65.726311	0.000585	138.741302
65.739998	135.833008	65.746315	0.000413	138.727417
65.759995	129.167007	65.766312	0.000303	138.713562
65.779999	140.333008	65.786316	0.000236	138.699615
65.800003	132.333008	65.806320	0.000198	138.685638
65.820000	129.667007	65.826317	0.000185	138.671661
65.840004	132.333008	65.846321	0.000193	138.657623
65.860001	130.667007	65.866318	0.000225	138.643585
65.880005	134.333008	65.886322	0.000287	138.629486
65.900002	140.000000	65.906319	0.000389	138.615326
65.919998	128.500000	65.926315	0.000550	138.601196
65.940002	138.500000	65.946320	0.000804	138.587006
65.959999	136.833008	65.966316	0.001202	138.572784
65.980003	135.167007	65.986320	0.001836	138.558502
66.000000	132.167007	66.006317	0.002859	138.544250
66.019997	135.667007	66.026314	0.004536	138.529907
66.040001	130.333008	66.046318	0.007342	138.515564
66.059998	131.833008	66.066315	0.012127	138.501190
66.080002	135.167007	66.086319	0.020474	138.486755
66.099998	136.667007	66.106316	0.035365	138.472351
66.119995	131.833008	66.126312	0.062606	138.457855
66.139999	137.667007	66.146317	0.113798	138.443359
66.159996	136.500000	66.166313	0.212620	138.428833
66.180000	142.333008	66.186317	0.408883	138.414276
66.199997	146.500000	66.206314	0.808294	138.399689
66.219994	149.667007	66.226311	1.636762	138.385101
66.239998	159.500000	66.246323	3.368773	138.370422
66.259995	161.000000	66.266319	6.939976	138.355774
66.279999	165.833008	66.286324	14.024577	138.341095
66.300003	189.000000	66.306328	27.034718	138.326355
66.320000	225.000000	66.326324	48.076832	138.311615
66.340004	259.666992	66.346329	76.123878	138.296844
66.360001	277.666992	66.366325	103.720772	138.282043
66.380005	308.832977	66.386330	118.593170	138.267242
66.400002	309.500000	66.406326	112.538712	138.252411
66.419998	281.000000	66.426323	89.296410	138.237549
66.440002	231.000000	66.446327	60.776524	138.222626

66.459999	195.833008	66.466324	36.868809	138.207703
66.480003	176.000000	66.486328	20.733715	138.192780
66.500000	162.833008	66.506325	11.162924	138.177826
66.519997	156.667007	66.526321	5.873593	138.162842
66.540001	140.833008	66.546326	3.057395	138.147827
66.559998	135.667007	66.566322	1.587949	138.132812
66.580002	131.500000	66.586327	0.827653	138.117767
66.599998	133.000000	66.606323	0.435441	138.102692
66.619995	129.000000	66.626320	0.232252	138.087646
66.639999	132.500000	66.646324	0.126010	138.072510
66.659996	129.833008	66.666321	0.069753	138.057404
66.680000	128.333008	66.686325	0.039433	138.042267
66.699997	133.333008	66.706322	0.022789	138.027100
66.719994	133.333008	66.726318	0.013458	138.011902
66.739998	137.000000	66.746323	0.008114	137.996735
66.759995	127.833000	66.766319	0.004993	137.981537
66.779999	121.500000	66.786324	0.003131	137.966278
66.800003	131.000000	66.806335	0.001999	137.951050
66.820000	128.500000	66.826332	0.001299	137.935791
66.840004	125.667000	66.846336	0.000859	137.920502
66.860001	130.667007	66.866333	0.000578	137.905243
66.880005	123.833000	66.886337	0.000397	137.889923
66.900002	133.167007	66.906334	0.000280	137.874634
66.919998	128.167007	66.926331	0.000206	137.859314
66.940002	128.500000	66.946335	0.000163	137.843964
66.959999	122.500000	66.966331	0.000144	137.828583
66.980003	134.667007	66.986336	0.000150	137.813232
67.000000	124.333000	67.006332	0.000188	137.797852
67.019997	130.833008	67.026329	0.000275	137.782501
67.040001	130.333008	67.046333	0.000445	137.767090
67.059998	130.667007	67.066330	0.000773	137.751678
67.080002	125.500000	67.086334	0.001406	137.736267
67.099998	124.333000	67.106331	0.002653	137.720825
67.119995	127.833000	67.126328	0.005165	137.705414
67.139999	130.167007	67.146332	0.010334	137.689972
67.159996	129.167007	67.166328	0.021098	137.674500
67.180000	134.167007	67.186333	0.043471	137.659058
67.199997	126.667000	67.206329	0.088759	137.643555

67.219994	130.333008	67.226326	0.175256	137.628113
67.239998	131.333008	67.246330	0.324287	137.612640
67.259995	134.333008	67.266327	0.541607	137.597168
67.279999	131.833008	67.286331	0.785730	137.581665
67.300003	129.333008	67.306335	0.957216	137.566193
67.320000	138.333008	67.326332	0.960505	137.550690
67.340004	134.333008	67.346336	0.795880	137.535217
67.360001	129.333008	67.366341	0.558684	137.519684
67.380005	138.167007	67.386345	0.346111	137.504211
67.400002	140.833008	67.406342	0.197566	137.488708
67.419998	133.167007	67.426338	0.107477	137.473206
67.440002	140.333008	67.446342	0.056943	137.457672
67.459999	128.667007	67.466339	0.029780	137.442230
67.480003	145.667007	67.486343	0.015495	137.426727
67.500000	138.333008	67.506340	0.008078	137.411224
67.519997	135.333008	67.526329	0.004244	137.395721
67.540001	132.167007	67.546333	0.002256	137.380219
67.559998	129.500000	67.566330	0.001219	137.364716
67.580002	130.833008	67.586334	0.000671	137.349213
67.599998	130.833008	67.606331	0.000378	137.333740
67.619995	132.000000	67.626328	0.000217	137.318268
67.639999	125.833000	67.646332	0.000128	137.302795
67.659996	127.667000	67.666328	0.000077	137.287292
67.680000	128.667007	67.686333	0.000048	137.271851
67.699997	135.167007	67.706329	0.000031	137.256409
67.719994	127.000000	67.726326	0.000021	137.240936
67.739998	123.000000	67.746330	0.000015	137.225494
67.759995	130.167007	67.766327	0.000012	137.210052
67.779999	139.333008	67.786331	0.000010	137.194611
67.800003	119.333000	67.806335	0.000011	137.179199
67.820000	126.000000	67.826332	0.000012	137.163788
67.840004	125.500000	67.846336	0.000015	137.148376
67.860001	132.500000	67.866333	0.000020	137.132996
67.880005	129.000000	67.886337	0.000028	137.117615
67.900002	133.333008	67.906334	0.000039	137.102234
67.919998	133.167007	67.926338	0.000057	137.086884
67.940002	121.333000	67.946342	0.000083	137.071533
67.959999	127.000000	67.966339	0.000123	137.056244

67.980003	128.667007	67.986343	0.000185	137.040924
68.000000	129.167007	68.006340	0.000284	137.025635
68.019997	125.333000	68.026337	0.000442	137.010345
68.040001	126.000000	68.046341	0.000699	136.995056
68.059998	124.167000	68.066338	0.001126	136.979828
68.080002	139.833008	68.086342	0.001851	136.964600
68.099998	135.833008	68.106339	0.003102	136.949371
68.119995	135.500000	68.126335	0.005314	136.934174
68.139999	136.833008	68.146339	0.009316	136.918976
68.159996	135.500000	68.166336	0.016730	136.903839
68.180000	120.833000	68.186340	0.030833	136.888702
68.199997	125.167000	68.206337	0.058355	136.873566
68.219994	123.167000	68.226334	0.113451	136.858429
68.239998	127.500000	68.246338	0.226191	136.843384
68.259995	127.833000	68.266335	0.459724	136.828339
68.279999	131.667007	68.286339	0.943138	136.813263
68.300003	130.667007	68.306343	1.920231	136.798279
68.320000	133.667007	68.326340	3.788690	136.783295
68.340004	138.833008	68.346344	7.031556	136.768341
68.360001	142.167007	68.366341	11.840410	136.753387
68.380005	152.333008	68.386345	17.430014	136.738495
68.400002	152.000000	68.406342	21.690615	136.723572
68.419998	155.500000	68.426338	22.357533	136.708740
68.440002	145.167007	68.446342	19.078920	136.693878
68.459999	142.500000	68.466339	13.779913	136.679108
68.480003	139.000000	68.486351	8.745833	136.664307
68.500000	131.167007	68.506348	5.088840	136.649567
68.519997	136.167007	68.526344	2.808252	136.634827
68.540001	137.000000	68.546349	1.503261	136.620148
68.559998	124.333000	68.566345	0.791738	136.605499
68.580002	128.167007	68.586349	0.413766	136.590881
68.599998	122.333000	68.606346	0.216207	136.576263
68.619995	123.500000	68.626343	0.113694	136.561707
68.639999	120.333000	68.646347	0.060597	136.547180
68.659996	128.833008	68.666344	0.033099	136.532715
68.680000	123.000000	68.686348	0.018916	136.518219
68.699997	125.833000	68.706345	0.011886	136.503815
68.719994	121.833000	68.726341	0.009043	136.489441

68.739998	125.333000	68.746346	0.009259	136.475067
68.759995	123.500000	68.766342	0.012735	136.460754
68.779999	123.333000	68.786346	0.021186	136.446442
68.800003	123.500000	68.806351	0.038857	136.432220
68.820000	132.333008	68.826347	0.075056	136.418030
68.840004	130.000000	68.846352	0.149718	136.403839
68.860001	128.333008	68.866348	0.304342	136.389709
68.880005	131.167007	68.886353	0.621853	136.375610
68.900002	137.667007	68.906349	1.252017	136.361603
68.919998	135.667007	68.926346	2.421519	136.347595
68.940002	137.333008	68.946350	4.359491	136.333618
68.959999	140.167007	68.966347	7.046260	136.319702
68.980003	150.167007	68.986351	9.876359	136.305817
69.000000	144.000000	69.006348	11.689327	136.291962
69.019997	135.000000	69.026344	11.607480	136.278198
69.040001	138.000000	69.046356	9.913730	136.264465
69.059998	145.500000	69.066353	7.749290	136.250763
69.080002	130.000000	69.086357	5.983211	136.237091
69.099998	137.667007	69.106354	4.711894	136.223480
69.119995	122.667000	69.126350	3.641300	136.209930
69.139999	135.667007	69.146355	2.613405	136.196411
69.159996	127.167000	69.166351	1.704760	136.182983
69.180000	129.333008	69.186356	1.022053	136.169556
69.199997	126.833000	69.206352	0.577737	136.156189
69.219994	131.500000	69.226349	0.315200	136.142883
69.239998	128.500000	69.246353	0.168831	136.129608
69.259995	128.000000	69.266350	0.090096	136.116394
69.279999	128.333008	69.286354	0.048782	136.103241
69.300003	129.667007	69.306358	0.027825	136.090118
69.320000	119.500000	69.326355	0.018088	136.077057
69.340004	132.000000	69.346359	0.015078	136.064056
69.360001	132.667007	69.366356	0.017144	136.051117
69.380005	130.000000	69.386360	0.024737	136.038239
69.400002	127.167000	69.406357	0.040595	136.025391
69.419998	129.333008	69.426353	0.071074	136.012604
69.440002	121.500000	69.446358	0.129444	135.999908
69.459999	121.167000	69.466354	0.243035	135.987213
69.480003	127.333000	69.486359	0.469192	135.974609

69.500000	129.000000	69.506355	0.928426	135.962067
69.519997	134.500000	69.526352	1.873968	135.949585
69.540001	135.000000	69.546356	3.823076	135.937134
69.559998	142.833008	69.566353	7.759582	135.924774
69.580002	156.833008	69.586365	15.342367	135.912476
69.599998	173.833008	69.606361	28.694248	135.900208
69.619995	210.500000	69.626358	49.105293	135.888031
69.639999	237.167007	69.646362	74.172096	135.875916
69.659996	253.333008	69.666359	95.619675	135.863861
69.680000	254.167007	69.686363	102.855988	135.851868
69.699997	249.833008	69.706360	91.870293	135.839935
69.719994	230.167007	69.726357	69.277191	135.828033
69.739998	207.333008	69.746361	45.607830	135.816223
69.759995	181.167007	69.766357	27.295498	135.804504
69.779999	166.833008	69.786362	15.375417	135.792816
69.800003	149.500000	69.806366	8.355515	135.781219
69.820000	142.000000	69.826363	4.449290	135.769714
69.840004	132.833008	69.846367	2.345606	135.758209
69.860001	136.333008	69.866364	1.239087	135.746796
69.880005	128.000000	69.886368	0.669792	135.735504
69.900002	114.833000	69.906364	0.391957	135.724243
69.919998	124.000000	69.926361	0.284434	135.713043
69.940002	122.833000	69.946365	0.305031	135.701904
69.959999	124.333000	69.966362	0.473816	135.690857
69.980003	130.333008	69.986366	0.878674	135.679901
70.000000	138.167007	70.006363	1.685444	135.668976
70.019997	127.333000	70.026360	3.110705	135.658173
70.040001	146.333008	70.046364	5.273622	135.647430
70.059998	143.833008	70.066360	7.892044	135.636719
70.080002	152.000000	70.086365	10.084992	135.626129
70.099998	147.833008	70.106361	10.762360	135.615601
70.119995	145.667007	70.126358	9.555431	135.605133
70.139999	139.833008	70.146370	7.178264	135.594757
70.159996	136.167007	70.166367	4.721181	135.584473
70.180000	133.000000	70.186371	2.827178	135.574219
70.199997	127.167000	70.206367	1.595763	135.564087
70.219994	133.833008	70.226364	0.869220	135.554016
70.239998	124.000000	70.246368	0.463634	135.544006

70.259995	127.167000	70.266365	0.244823	135.534119
70.279999	130.000000	70.286369	0.129321	135.524292
70.300003	128.667007	70.306374	0.069643	135.514526
70.320000	131.667007	70.326370	0.040065	135.504883
70.340004	125.667000	70.346375	0.027636	135.495270
70.360001	129.833008	70.366371	0.027259	135.485809
70.380005	122.500000	70.386375	0.039707	135.476410
70.400002	123.667000	70.406372	0.072155	135.467041
70.419998	132.500000	70.426369	0.140602	135.457825
70.440002	128.000000	70.446373	0.271970	135.448639
70.459999	122.000000	70.466370	0.498607	135.439545
70.480003	131.333008	70.486374	0.834056	135.430511
70.500000	120.833000	70.506371	1.226960	135.421631
70.519997	131.833008	70.526367	1.538866	135.412811
70.540001	126.333000	70.546371	1.614746	135.404083
70.559998	124.500000	70.566368	1.419536	135.395416
70.580002	123.167000	70.586372	1.075756	135.386871
70.599998	126.000000	70.606369	0.751050	135.378387
70.619995	130.500000	70.626366	0.551736	135.370026
70.639999	122.000000	70.646370	0.527124	135.361725
70.659996	135.167007	70.666367	0.730085	135.353546
70.680000	122.500000	70.686378	1.267519	135.345428
70.699997	124.333000	70.706367	2.303088	135.337402
70.719994	126.167000	70.726364	3.972879	135.329468
70.739998	127.333000	70.746368	6.157516	135.321625
70.759995	131.167007	70.766365	8.245271	135.313873
70.779999	132.167007	70.786369	9.291273	135.306213
70.800003	132.833008	70.806374	8.723614	135.298676
70.820000	128.667007	70.826370	6.898468	135.291199
70.840004	120.500000	70.846375	4.728599	135.283844
70.860001	132.833008	70.866371	2.919637	135.276550
70.880005	126.167000	70.886375	1.682592	135.269379
70.900002	125.167000	70.906372	0.929670	135.262299
70.919998	127.500000	70.926369	0.500637	135.255341
70.940002	122.667000	70.946373	0.265509	135.248413
70.959999	115.333000	70.966370	0.139903	135.241608
70.980003	117.000000	70.986374	0.073835	135.234924
71.000000	129.667007	71.006371	0.039514	135.228333

71.019997	129.000000	71.026367	0.021953	135.221832
71.040001	120.500000	71.046371	0.013423	135.215454
71.059998	132.000000	71.066368	0.010244	135.209137
71.080002	131.667007	71.086372	0.011195	135.202942
71.099998	133.833008	71.106369	0.017082	135.196869
71.119995	127.667000	71.126366	0.031282	135.190857
71.139999	123.833000	71.146370	0.061467	135.184967
71.159996	128.500000	71.166367	0.122404	135.179169
71.180000	133.000000	71.186371	0.238664	135.173462
71.199997	135.833008	71.206375	0.441223	135.167877
71.219994	132.667007	71.226372	0.747539	135.162384
71.239998	143.333008	71.246376	1.121404	135.157013
71.259995	138.833008	71.266373	1.443354	135.151733
71.279999	148.333008	71.286377	1.562147	135.146545
71.300003	147.833008	71.306381	1.419577	135.141449
71.320000	140.667007	71.326378	1.110458	135.136505
71.340004	139.333008	71.346382	0.793420	135.131653
71.360001	133.167007	71.366379	0.580019	135.126892
71.380005	138.000000	71.386383	0.518404	135.122253
71.400002	131.167007	71.406380	0.646912	135.117706
71.419998	134.167007	71.426376	1.059426	135.113251
71.440002	139.667007	71.446381	1.986467	135.108917
71.459999	136.667007	71.466377	3.930932	135.104706
71.480003	161.333008	71.486382	7.916523	135.100586
71.500000	172.167007	71.506378	15.836318	135.096619
71.519997	208.833008	71.526375	30.721872	135.092712
71.540001	242.667007	71.546379	56.196167	135.088959
71.559998	297.832977	71.566376	93.854279	135.085266
71.580002	334.166992	71.586380	138.718979	135.081696
71.599998	367.832977	71.606377	176.884933	135.078217
71.619995	384.666992	71.626373	192.552399	135.074890
71.639999	358.166992	71.646378	180.648193	135.071655
71.659996	336.832977	71.666374	149.931931	135.068542
71.680000	283.000000	71.686378	113.077705	135.065521
71.699997	244.000000	71.706375	78.615227	135.062622
71.719994	211.667007	71.726372	50.648869	135.059814
71.739998	194.000000	71.746384	30.494360	135.057159
71.759995	162.500000	71.766380	17.436838	135.054626

71.779999	152.167007	71.786385	9.622173	135.052185
71.800003	147.167007	71.806389	5.194406	135.049835
71.820000	147.500000	71.826385	2.770664	135.047607
71.840004	136.333008	71.846390	1.470824	135.045502
71.860001	127.167000	71.866386	0.783186	135.043518
71.880005	135.833008	71.886391	0.421729	135.041687
71.900002	124.500000	71.906387	0.232887	135.039886
71.919998	128.000000	71.926384	0.135628	135.038239
71.940002	133.167007	71.946388	0.088703	135.036713
71.959999	127.833000	71.966385	0.072639	135.035309
71.980003	138.167007	71.986389	0.081010	135.034027
72.000000	135.333008	72.006386	0.117818	135.032837
72.019997	136.833008	72.026382	0.199946	135.031799
72.040001	140.833008	72.046387	0.366709	135.030853
72.059998	136.500000	72.066383	0.701826	135.030029
72.080002	139.667007	72.086388	1.381660	135.029327
72.099998	142.667007	72.106384	2.769023	135.028748
72.119995	137.833008	72.126381	5.585277	135.028259
72.139999	149.500000	72.146385	11.156045	135.027924
72.159996	171.167007	72.166382	21.566090	135.027679
72.180000	197.500000	72.186386	39.250824	135.027557
72.199997	220.833008	72.206383	65.094292	135.027588
72.219994	255.667007	72.226379	95.182068	135.027710
72.239998	278.666992	72.246384	119.243538	135.027954
72.259995	296.666992	72.266380	125.803177	135.028320
72.279999	284.000000	72.286392	111.967834	135.028809
72.300003	257.666992	72.306396	86.173309	135.029419
72.320000	234.667007	72.326393	59.948864	135.030151
72.340004	214.167007	72.346397	39.576885	135.031006
72.360001	186.500000	72.366394	25.567513	135.031982
72.380005	169.833008	72.386398	16.165054	135.033051
72.400002	165.667007	72.406395	9.866520	135.034271
72.419998	156.167007	72.426392	5.773180	135.035614
72.440002	149.167007	72.446396	3.256037	135.037079
72.459999	134.667007	72.466393	1.790947	135.038666
72.480003	137.833008	72.486397	0.970464	135.040375
72.500000	130.500000	72.506393	0.522534	135.042206
72.519997	125.833000	72.526390	0.281380	135.044159

72.540001	134.000000	72.546394	0.152493	135.046234
72.559998	125.000000	72.566391	0.083857	135.048401
72.580002	129.500000	72.586395	0.047336	135.050751
72.599998	131.500000	72.606392	0.028131	135.053192
72.619995	123.333000	72.626389	0.018595	135.055756
72.639999	129.333008	72.646393	0.015084	135.058441
72.659996	127.333000	72.666389	0.016400	135.061249
72.680000	128.167007	72.686394	0.023334	135.064209
72.699997	119.167000	72.706390	0.039179	135.067261
72.719994	122.833000	72.726387	0.071615	135.070435
72.739998	128.500000	72.746391	0.137073	135.073761
72.759995	133.167007	72.766388	0.269826	135.077179
72.779999	124.167000	72.786392	0.540335	135.080750
72.800003	135.500000	72.806404	1.086769	135.084442
72.820000	138.500000	72.826401	2.155737	135.088226
72.840004	135.667007	72.846405	4.124207	135.092194
72.860001	133.333008	72.866402	7.392432	135.096252
72.880005	147.000000	72.886406	12.026820	135.100433
72.900002	157.167007	72.906403	17.185181	135.104767
72.919998	141.500000	72.926399	20.986185	135.109192
72.940002	144.667007	72.946404	21.554323	135.113739
72.959999	155.333008	72.966400	18.639456	135.118439
72.980003	145.667007	72.986404	13.837265	135.123260
73.000000	137.833008	73.006401	9.112793	135.128174
73.019997	133.667007	73.026398	5.516737	135.133240
73.040001	137.500000	73.046402	3.160960	135.138458
73.059998	131.167007	73.066399	1.749161	135.143768
73.080002	134.333008	73.086403	0.945947	135.149231
73.099998	129.333008	73.106400	0.504500	135.154785
73.119995	116.167000	73.126396	0.267222	135.160461
73.139999	125.167000	73.146400	0.141492	135.166290
73.159996	125.667000	73.166397	0.075397	135.172241
73.180000	131.333008	73.186401	0.040640	135.178314
73.199997	133.333008	73.206398	0.022284	135.184540
73.219994	135.500000	73.226395	0.012502	135.190857
73.239998	128.500000	73.246399	0.007245	135.197327
73.259995	132.833008	73.266396	0.004423	135.203888
73.279999	129.833008	73.286400	0.002956	135.210602

73.300003	131.667007	73.306404	0.002301	135.217438
73.320000	127.167000	73.326408	0.002207	135.224365
73.340004	130.333008	73.346413	0.002609	135.231476
73.360001	140.333008	73.366409	0.003594	135.238678
73.380005	136.333008	73.386414	0.005419	135.246002
73.400002	134.167007	73.406410	0.008604	135.253479
73.419998	137.000000	73.426407	0.014119	135.261078
73.440002	135.167007	73.446411	0.023768	135.268799
73.459999	130.000000	73.466408	0.040923	135.276611
73.480003	130.000000	73.486412	0.072056	135.284576
73.500000	129.833008	73.506409	0.129733	135.292694
73.519997	135.833008	73.526405	0.239032	135.300903
73.540001	129.333008	73.546410	0.450949	135.309265
73.559998	123.667000	73.566406	0.870096	135.317719
73.580002	126.833000	73.586411	1.713327	135.326324
73.599998	128.167007	73.606407	3.420848	135.335052
73.619995	139.333008	73.626404	6.854020	135.343872
73.639999	154.500000	73.646408	13.561811	135.352875
73.659996	161.333008	73.666405	25.903858	135.361969
73.680000	195.333008	73.686409	46.483154	135.371185
73.699997	210.167007	73.706406	75.913628	135.380524
73.719994	227.167007	73.726402	109.330933	135.389984
73.739998	249.500000	73.746407	135.134949	135.399597
73.759995	249.000000	73.766403	141.005249	135.409332
73.779999	240.500000	73.786407	124.262016	135.419159
73.800003	223.667007	73.806412	94.343834	135.429138
73.820000	208.667007	73.826408	64.171654	135.439209
73.840004	189.000000	73.846420	41.575821	135.449432
73.860001	181.833008	73.866417	28.660982	135.459808
73.880005	187.000000	73.886421	25.170965	135.470245
73.900002	195.833008	73.906418	31.308575	135.480865
73.919998	230.667007	73.926414	48.210270	135.491547
73.940002	263.666992	73.946419	76.095100	135.502411
73.959999	289.666992	73.966415	110.449982	135.513367
73.980003	338.500000	73.986420	139.775024	135.524445
74.000000	343.500000	74.006416	150.187897	135.535675
74.019997	331.666992	74.026413	136.242630	135.546967
74.040001	297.500000	74.046417	105.755623	135.558441

74.059998	265.666992	74.066414	72.297180	135.570007
74.080002	218.833008	74.086418	45.047138	135.581726
74.099998	184.167007	74.106415	26.388832	135.593536
74.119995	172.000000	74.126411	14.849069	135.605469
74.139999	154.833008	74.146416	8.133536	135.617554
74.159996	157.667007	74.166412	4.378233	135.629730
74.180000	145.000000	74.186417	2.332279	135.642029
74.199997	129.333008	74.206413	1.238588	135.654449
74.219994	128.500000	74.226410	0.659787	135.667023
74.239998	131.333008	74.246414	0.354416	135.679688
74.259995	125.667000	74.266411	0.192923	135.692444
74.279999	133.000000	74.286415	0.106710	135.705353
74.300003	125.167000	74.306419	0.060119	135.718384
74.320000	125.000000	74.326416	0.034553	135.731537
74.340004	123.667000	74.346420	0.020273	135.744812
74.360001	120.500000	74.366425	0.012167	135.758179
74.380005	119.167000	74.386429	0.007501	135.771698
74.400002	124.167000	74.406425	0.004800	135.785309
74.419998	124.333000	74.426422	0.003257	135.799011
74.440002	127.000000	74.446426	0.002443	135.812897
74.459999	121.500000	74.466423	0.002144	135.826843
74.480003	125.333000	74.486427	0.002290	135.840942
74.500000	119.667000	74.506424	0.002936	135.855133
74.519997	121.000000	74.526413	0.004282	135.869446
74.540001	123.500000	74.546417	0.006757	135.883911
74.559998	126.833000	74.566414	0.011180	135.898468
74.580002	129.167007	74.586418	0.019113	135.913116
74.599998	121.500000	74.606415	0.033535	135.927917
74.619995	125.333000	74.626411	0.060268	135.942810
74.639999	127.000000	74.646416	0.110903	135.957825
74.659996	121.000000	74.666412	0.208804	135.972931
74.680000	130.667007	74.686417	0.402010	135.988190
74.699997	131.667007	74.706413	0.788720	136.003540
74.719994	127.333000	74.726410	1.567359	136.018982
74.739998	132.667007	74.746414	3.121301	136.034576
74.759995	134.333008	74.766411	6.123924	136.050262
74.779999	148.833008	74.786415	11.582744	136.066071
74.800003	146.833008	74.806419	20.546143	136.082001

74.820000	173.000000	74.826416	33.153061	136.098022
74.840004	187.667007	74.846420	47.238350	136.114166
74.860001	198.167007	74.866417	57.991077	136.130371
74.880005	198.833008	74.886429	60.671730	136.146759
74.900002	199.167007	74.906425	54.760330	136.163239
74.919998	191.667007	74.926422	44.841282	136.179779
74.940002	181.833008	74.946426	37.070511	136.196472
74.959999	173.500000	74.966423	35.623653	136.213257
74.980003	187.167007	74.986427	41.530914	136.230133
75.000000	194.167007	75.006424	52.464558	136.247162
75.019997	187.833008	75.026421	62.877293	136.264282
75.040001	199.167007	75.046425	66.340775	136.281464
75.059998	198.333008	75.066422	60.036129	136.298798
75.080002	185.667007	75.086426	46.843540	136.316223
75.099998	173.667007	75.106422	32.303314	136.333771
75.119995	159.333008	75.126419	20.333464	136.351410
75.139999	144.333008	75.146423	12.030160	136.369141
75.159996	148.667007	75.166420	6.836990	136.386963
75.180000	130.667007	75.186424	3.784488	136.404938
75.199997	136.000000	75.206421	2.066533	136.423004
75.219994	141.833008	75.226418	1.133489	136.441132
75.239998	142.333008	75.246422	0.650017	136.459381
75.259995	141.500000	75.266418	0.427912	136.477753
75.279999	138.167007	75.286423	0.375327	136.496185
75.300003	138.500000	75.306427	0.470535	136.514740
75.320000	141.333008	75.326424	0.758580	136.533417
75.340004	145.333008	75.346428	1.380095	136.552155
75.360001	149.167007	75.366425	2.647684	136.571014
75.380005	147.500000	75.386429	5.211450	136.589966
75.400002	162.333008	75.406433	10.353366	136.609009
75.419998	188.500000	75.426430	20.420509	136.628143
75.440002	218.833008	75.446434	39.199478	136.647369
75.459999	267.500000	75.466431	71.373489	136.666718
75.480003	334.832977	75.486435	119.780159	136.686157
75.500000	397.166992	75.506432	179.640900	136.705688
75.519997	464.000000	75.526428	234.208328	136.725311
75.540001	496.166992	75.546432	260.252197	136.745026
75.559998	470.666992	75.566429	244.935532	136.764832

75.580002	402.332977	75.586433	197.291870	136.784698
75.599998	365.832977	75.606430	139.514526	136.804688
75.619995	297.000000	75.626427	89.486710	136.824768
75.639999	245.333008	75.646431	53.670124	136.844940
75.659996	210.500000	75.666428	30.794083	136.865204
75.680000	195.667007	75.686432	17.137821	136.885559
75.699997	174.000000	75.706429	9.342362	136.905975
75.719994	159.833008	75.726425	5.026013	136.926514
75.739998	153.333008	75.746429	2.688817	136.947144
75.759995	149.167007	75.766426	1.444678	136.967834
75.779999	145.000000	75.786430	0.790778	136.988617
75.800003	135.500000	75.806435	0.455164	137.009491
75.820000	130.000000	75.826431	0.296546	137.030457
75.840004	135.667007	75.846436	0.248929	137.051483
75.860001	132.167007	75.866432	0.294935	137.072601
75.880005	143.000000	75.886436	0.460048	137.093811
75.900002	138.500000	75.906441	0.828323	137.115112
75.919998	143.167007	75.926437	1.587599	137.136505
75.940002	142.167007	75.946442	3.123578	137.157928
75.959999	152.333008	75.966438	6.173172	137.179443
75.980003	168.833008	75.986443	12.021880	137.201050
76.000000	175.500000	76.006439	22.533014	137.222748
76.019997	206.167007	76.026436	39.573288	137.244537
76.040001	225.000000	76.046440	63.221920	137.266388
76.059998	241.000000	76.066437	89.183731	137.288300
76.080002	258.332977	76.086441	108.425438	137.310333
76.099998	266.166992	76.106438	112.006226	137.332367
76.119995	257.832977	76.126434	98.428574	137.354523
76.139999	241.333008	76.146439	74.854416	137.376770
76.159996	212.667007	76.166435	50.735241	137.399048
76.180000	185.500000	76.186440	31.650093	137.421417
76.199997	170.000000	76.206436	18.684618	137.443848
76.219994	160.667007	76.226433	10.644426	137.466370
76.239998	163.667007	76.246437	5.946965	137.488953
76.259995	148.333008	76.266434	3.342516	137.511597
76.279999	152.167007	76.286438	2.008765	137.534332
76.300003	150.000000	76.306442	1.487545	137.557129
76.320000	141.833008	76.326439	1.607797	137.579987

76.340004	145.333008	76.346443	2.471455	137.602875
76.360001	147.333008	76.366440	4.512841	137.625916
76.380005	161.167007	76.386444	8.624700	137.648956
76.400002	176.500000	76.406448	16.221548	137.672089
76.419998	197.333008	76.426445	28.913307	137.695251
76.440002	222.167007	76.446449	47.309669	137.718506
76.459999	239.833008	76.466446	68.910187	137.741821
76.480003	264.332977	76.486450	87.071472	137.765167
76.500000	261.000000	76.506447	93.813728	137.788605
76.519997	249.833008	76.526443	85.939163	137.812103
76.540001	226.333008	76.546448	67.811592	137.835663
76.559998	216.000000	76.566444	47.336071	137.859253
76.580002	199.667007	76.586449	30.177210	137.882904
76.599998	187.833008	76.606445	18.088537	137.906647
76.619995	163.667007	76.626442	10.406271	137.930420
76.639999	167.667007	76.646446	5.826637	137.954254
76.659996	148.500000	76.666443	3.219839	137.978119
76.680000	147.833008	76.686447	1.796423	138.002106
76.699997	143.500000	76.706444	1.069329	138.026062
76.719994	144.667007	76.726440	0.765835	138.050140
76.739998	137.333008	76.746445	0.767748	138.074219
76.759995	145.000000	76.766441	1.084265	138.098358
76.779999	136.667007	76.786446	1.872931	138.122559
76.800003	141.667007	76.806450	3.528439	138.146790
76.820000	150.333008	76.826447	6.879479	138.171082
76.840004	165.667007	76.846451	13.548274	138.195404
76.860001	193.833008	76.866447	26.421665	138.219818
76.880005	222.833008	76.886452	49.963505	138.244232
76.900002	284.832977	76.906448	89.268402	138.268707
76.919998	378.832977	76.926453	146.522934	138.293243
76.940002	438.832977	76.946457	214.573196	138.317810
76.959999	518.166992	76.966454	273.318024	138.342407
76.980003	544.666992	76.986458	298.026855	138.367035
77.000000	519.333008	77.006454	277.979034	138.391724
77.019997	451.166992	77.026451	226.566650	138.416443
77.040001	396.166992	77.046455	170.013382	138.441193
77.059998	347.666992	77.066452	129.663742	138.466003
77.080002	336.832977	77.086456	115.125320	138.490845

77.099998	350.666992	77.106453	126.620132	138.515717
77.119995	376.832977	77.126450	156.396790	138.540619
77.139999	401.500000	77.146454	188.475281	138.565552
77.159996	423.166992	77.166451	203.374908	138.590515
77.180000	401.166992	77.186455	190.050720	138.615509
77.199997	350.332977	77.206451	153.752625	138.640564
77.219994	306.332977	77.226448	109.861061	138.665619
77.239998	252.000000	77.246452	71.395584	138.690704
77.259995	208.667007	77.266449	43.438435	138.715851
77.279999	193.667007	77.286453	25.265224	138.740967
77.300003	175.333008	77.306458	14.241260	138.766144
77.320000	162.000000	77.326454	7.850369	138.791351
77.340004	160.167007	77.346458	4.261005	138.816559
77.360001	157.167007	77.366455	2.294894	138.841827
77.380005	141.000000	77.386459	1.235572	138.867096
77.400002	144.667007	77.406456	0.672119	138.892426
77.419998	146.667007	77.426460	0.376183	138.917725
77.440002	127.333000	77.446465	0.226959	138.943085
77.459999	133.833008	77.466461	0.164713	138.968414
77.480003	128.167007	77.486465	0.167178	138.993805
77.500000	130.167007	77.506462	0.240296	139.019196
77.519997	131.167007	77.526459	0.417806	139.044586
77.540001	134.833008	77.546463	0.762514	139.070038
77.559998	129.333008	77.566460	1.352285	139.095459
77.580002	135.667007	77.586464	2.228763	139.120911
77.599998	132.000000	77.606461	3.298569	139.146393
77.619995	131.333008	77.626457	4.266421	139.171844
77.639999	148.667007	77.646461	4.734807	139.197327
77.659996	129.833008	77.666458	4.485493	139.222839
77.680000	122.000000	77.686462	3.669124	139.248352
77.699997	136.333008	77.706459	2.666909	139.273834
77.719994	135.500000	77.726456	1.802990	139.299347
77.739998	131.167007	77.746460	1.220276	139.324921
77.759995	141.500000	77.766457	0.943234	139.350433
77.779999	125.833000	77.786461	0.986010	139.375977
77.800003	131.833008	77.806465	1.440798	139.401520
77.820000	139.833008	77.826462	2.558654	139.427063
77.840004	144.833008	77.846466	4.853897	139.452606

77.860001	145.833008	77.866463	9.195063	139.478119
77.880005	168.333008	77.886467	16.750092	139.503693
77.900002	173.833008	77.906464	28.433784	139.529236
77.919998	167.500000	77.926468	43.690643	139.554779
77.940002	174.833008	77.946472	59.152229	139.580322
77.959999	174.000000	77.966469	69.191208	139.605835
77.980003	187.000000	77.986473	69.498253	139.631348
78.000000	188.667007	78.006470	60.859680	139.656891
78.019997	179.333008	78.026466	48.821083	139.682373
78.040001	184.000000	78.046471	39.920147	139.707886
78.059998	208.500000	78.066467	39.262054	139.733368
78.080002	236.333008	78.086472	51.047588	139.758850
78.099998	292.000000	78.106468	79.633942	139.784302
78.119995	346.332977	78.126465	128.144669	139.809784
78.139999	417.166992	78.146469	193.381363	139.835236
78.159996	471.666992	78.166466	259.952972	139.860626
78.180000	526.000000	78.186470	302.590240	139.886078
78.199997	500.332977	78.206467	301.261383	139.911469
78.219994	465.500000	78.226463	257.593597	139.936859
78.239998	411.500000	78.246468	192.769379	139.962189
78.259995	341.166992	78.266464	129.960052	139.987549
78.279999	296.500000	78.286469	81.293228	140.012878
78.300003	254.500000	78.306473	48.314423	140.038177
78.320000	218.333008	78.326469	27.710325	140.063446
78.340004	196.000000	78.346474	15.478837	140.088715
78.360001	188.833008	78.366470	8.486020	140.113953
78.380005	172.667007	78.386475	4.595120	140.139160
78.400002	167.333008	78.406479	2.474917	140.164337
78.419998	146.333008	78.426476	1.334721	140.189484
78.440002	142.500000	78.446480	0.724471	140.214600
78.459999	135.167007	78.466476	0.397742	140.239716
78.480003	131.833008	78.486481	0.221484	140.264771
78.500000	131.000000	78.506477	0.125412	140.289795
78.519997	129.167007	78.526474	0.072272	140.314789
78.540001	134.667007	78.546478	0.042395	140.339752
78.559998	125.000000	78.566475	0.025322	140.364655
78.580002	136.500000	78.586479	0.015388	140.389557
78.599998	124.333000	78.606476	0.009511	140.414459

78.619995	131.500000	78.626472	0.005975	140.439240
78.639999	132.333008	78.646477	0.003811	140.464050
78.659996	132.833008	78.666473	0.002467	140.488800
78.680000	131.000000	78.686478	0.001619	140.513519
78.699997	134.333008	78.706474	0.001077	140.538147
78.719994	127.333000	78.726471	0.000726	140.562744
78.739998	125.333000	78.746475	0.000495	140.587372
78.759995	122.500000	78.766472	0.000341	140.611877
78.779999	131.333008	78.786476	0.000238	140.636353
78.800003	129.333008	78.806480	0.000167	140.660797
78.820000	123.500000	78.826477	0.000119	140.685211
78.840004	126.167000	78.846481	0.000085	140.709564
78.860001	127.000000	78.866478	0.000062	140.733826
78.880005	121.667000	78.886482	0.000045	140.758087
78.900002	129.500000	78.906487	0.000033	140.782288
78.919998	130.000000	78.926483	0.000025	140.806427
78.940002	132.000000	78.946487	0.000018	140.830536
78.959999	125.167000	78.966484	0.000014	140.854523
78.980003	129.333008	78.986488	0.000011	140.878510
79.000000	130.500000	79.006485	0.000008	140.902435
79.019997	135.500000	79.026482	0.000006	140.926300
79.040001	117.167000	79.046486	0.000005	140.950104
79.059998	129.667007	79.066483	0.000004	140.973877
79.080002	126.167000	79.086487	0.000003	140.997528
79.099998	124.667000	79.106483	0.000002	141.021179
79.119995	124.667000	79.126480	0.000002	141.044708
79.139999	131.167007	79.146484	0.000001	141.068237
79.159996	128.667007	79.166481	0.000001	141.091644
79.180000	124.500000	79.186485	0.000001	141.115021
79.199997	126.667000	79.206482	0.000001	141.138306
79.219994	118.833000	79.226479	0.000001	141.161560
79.239998	123.500000	79.246483	0.000000	141.184723
79.259995	126.333000	79.266479	0.000000	141.207825
79.279999	117.333000	79.286484	0.000000	141.230835
79.300003	128.667007	79.306488	0.000000	141.253815
79.320000	118.000000	79.326485	0.000000	141.276672
79.340004	125.167000	79.346489	0.000000	141.299469
79.360001	124.833000	79.366486	0.000000	141.322235

79.380005	124.167000	79.386497	0.000000	141.344910
79.400002	125.333000	79.406494	0.000000	141.367493
79.419998	129.833008	79.426491	0.000000	141.389984
79.440002	117.000000	79.446495	0.000000	141.412415
79.459999	126.667000	79.466492	0.000000	141.434753
79.480003	123.833000	79.486496	0.000000	141.457031
79.500000	122.667000	79.506493	0.000000	141.479218
79.519997	120.167000	79.526489	0.000000	141.501312
79.540001	125.333000	79.546494	0.000000	141.523346
79.559998	131.667007	79.566490	0.000000	141.545288
79.580002	130.833008	79.586494	0.000000	141.567139
79.599998	127.167000	79.606483	0.000000	141.588898
79.619995	122.833000	79.626480	0.000000	141.610565
79.639999	134.333008	79.646484	0.000000	141.632172
79.659996	140.000000	79.666481	0.000000	141.653656
79.680000	133.167007	79.686485	0.000000	141.675079
79.699997	135.500000	79.706482	0.000000	141.696411
79.719994	122.667000	79.726479	0.000000	141.717621
79.739998	115.500000	79.746483	0.000000	141.738800
79.759995	131.167007	79.766479	0.000000	141.759827
79.779999	126.833000	79.786484	0.000000	141.780792
79.800003	126.333000	79.806488	0.000000	141.801666
79.820000	120.333000	79.826485	0.000000	141.822388
79.840004	133.500000	79.846489	0.000000	141.843048
79.860001	131.667007	79.866486	0.000000	141.863647
79.880005	128.000000	79.886497	0.000000	141.884125
79.900002	132.667007	79.906494	0.000000	141.904480
79.919998	125.500000	79.926491	0.000000	141.924744
79.940002	131.667007	79.946495	0.000000	141.944916
79.959999	131.833008	79.966492	0.000000	141.964966
79.980003	128.167007	79.986496	0.000000	141.984955
80.000000	130.667007	80.006493	0.000001	142.004791
80.019997	128.333008	80.026489	0.000001	142.024506
80.040001	126.833000	80.046494	0.000001	142.044189
80.059998	134.833008	80.066490	0.000001	142.063721
80.080002	123.833000	80.086494	0.000002	142.083130
80.099998	130.500000	80.106491	0.000002	142.102448
80.119995	128.000000	80.126488	0.000003	142.121674

80.139999	131.167007	80.146492	0.000004	142.140778
80.159996	123.167000	80.166489	0.000006	142.159760
80.180000	123.667000	80.186493	0.000009	142.178619
80.199997	139.333008	80.206490	0.000013	142.197357
80.219994	120.833000	80.226486	0.000018	142.216034
80.239998	128.833008	80.246490	0.000026	142.234558
80.259995	126.167000	80.266487	0.000039	142.252960
80.279999	126.667000	80.286491	0.000059	142.271240
80.300003	121.000000	80.306496	0.000089	142.289429
80.320000	132.333008	80.326492	0.000137	142.307526
80.340004	127.667000	80.346497	0.000213	142.325439
80.360001	120.667000	80.366501	0.000338	142.343262
80.380005	125.833000	80.386505	0.000543	142.360962
80.400002	124.833000	80.406502	0.000886	142.378540
80.419998	126.833000	80.426498	0.001471	142.395996
80.440002	120.500000	80.446503	0.002485	142.413330
80.459999	122.667000	80.466499	0.004273	142.430542
80.480003	123.000000	80.486504	0.007482	142.447601
80.500000	125.833000	80.506500	0.013344	142.464569
80.519997	124.333000	80.526497	0.024257	142.481384
80.540001	125.333000	80.546501	0.044952	142.498077
80.559998	124.667000	80.566498	0.084815	142.514679
80.580002	128.000000	80.586502	0.162598	142.531097
80.599998	131.667007	80.606499	0.314869	142.547424
80.619995	124.167000	80.626495	0.610327	142.563599
80.639999	129.667007	80.646500	1.167866	142.579651
80.659996	132.667007	80.666496	2.163545	142.595581
80.680000	140.500000	80.686501	3.793447	142.611359
80.699997	142.500000	80.706497	6.132133	142.626984
80.719994	141.167007	80.726494	8.906193	142.642517
80.739998	155.333008	80.746498	11.359488	142.657898
80.759995	151.500000	80.766495	12.524454	142.673126
80.779999	158.833008	80.786499	11.887743	142.688263
80.800003	148.500000	80.806503	9.805507	142.703217
80.820000	150.167007	80.826500	7.184333	142.718018
80.840004	146.000000	80.846512	4.808623	142.732758
80.860001	138.333008	80.866508	3.025079	142.747284
80.880005	140.167007	80.886513	1.831398	142.761688

80.900002	142.333008	80.906509	1.096957	142.775970
80.919998	145.167007	80.926506	0.686671	142.790070
80.940002	144.500000	80.946510	0.508704	142.804047
80.959999	141.000000	80.966507	0.526632	142.817902
80.980003	141.833008	80.986511	0.769655	142.831573
81.000000	132.667007	81.006508	1.353472	142.845123
81.019997	138.000000	81.026505	2.510280	142.858551
81.040001	143.833008	81.046509	4.598736	142.871796
81.059998	147.667007	81.066505	8.011703	142.884888
81.080002	159.000000	81.086510	12.890441	142.897858
81.099998	165.000000	81.106506	18.639767	142.910675
81.119995	177.500000	81.126503	23.681822	142.923309
81.139999	178.333008	81.146507	26.034943	142.935822
81.159996	175.833008	81.166504	24.667553	142.948212
81.180000	170.500000	81.186508	20.335339	142.960419
81.199997	167.833008	81.206505	14.905128	142.972443
81.219994	145.167007	81.226501	9.985556	142.984344
81.239998	145.167007	81.246506	6.274570	142.996124
81.259995	140.000000	81.266502	3.770605	143.007721
81.279999	135.833008	81.286507	2.192225	143.019135
81.300003	135.000000	81.306511	1.243592	143.030426
81.320000	137.500000	81.326515	0.694266	143.041565
81.340004	131.833008	81.346519	0.386626	143.052551
81.360001	136.000000	81.366516	0.220829	143.063385
81.380005	133.333008	81.386520	0.137671	143.074005
81.400002	132.000000	81.406517	0.105491	143.084534
81.419998	123.667000	81.426514	0.111185	143.094879
81.440002	120.833000	81.446518	0.156686	143.105042
81.459999	127.667000	81.466515	0.260690	143.115082
81.480003	134.167007	81.486519	0.468345	143.124939
81.500000	130.000000	81.506516	0.873187	143.134644
81.519997	131.167007	81.526512	1.662887	143.144165
81.540001	141.333008	81.546516	3.203563	143.153534
81.559998	154.333008	81.566513	6.172364	143.162781
81.580002	166.333008	81.586517	11.726373	143.171814
81.599998	167.833008	81.606514	21.542814	143.180695
81.619995	192.000000	81.626511	37.420471	143.189423
81.639999	211.833008	81.646515	59.961563	143.197968

81.659996	242.500000	81.666512	86.471786	143.206360
81.680000	248.500000	81.686516	110.038925	143.214600
81.699997	244.500000	81.706512	122.234802	143.222626
81.719994	258.000000	81.726509	119.055176	143.230530
81.739998	250.833008	81.746513	104.021271	143.238251
81.759995	242.167007	81.766510	84.659119	143.245819
81.779999	239.667007	81.786514	66.463867	143.253204
81.800003	208.667007	81.806526	50.914322	143.260406
81.820000	202.667007	81.826523	37.575397	143.267487
81.840004	186.000000	81.846527	26.254599	143.274353
81.860001	182.000000	81.866524	17.288191	143.281036
81.880005	168.000000	81.886528	10.806846	143.287567
81.900002	152.333008	81.906525	6.493759	143.293976
81.919998	145.667007	81.926521	3.789562	143.300140
81.940002	144.833008	81.946526	2.162867	143.306183
81.959999	137.833008	81.966522	1.214802	143.312012
81.980003	130.500000	81.986526	0.675470	143.317719
82.000000	129.167007	82.006523	0.375429	143.323181
82.019997	129.333008	82.026520	0.212041	143.328522
82.040001	129.667007	82.046524	0.126266	143.333679
82.059998	130.833008	82.066521	0.086144	143.338654
82.080002	128.667007	82.086525	0.076637	143.343475
82.099998	141.333008	82.106522	0.094911	143.348083
82.119995	133.833008	82.126518	0.149970	143.352539
82.139999	129.167007	82.146523	0.267649	143.356812
82.159996	126.167000	82.166519	0.502723	143.360931
82.180000	136.500000	82.186523	0.962461	143.364838
82.199997	134.667007	82.206520	1.840620	143.368591
82.219994	129.167007	82.226517	3.452545	143.372162
82.239998	141.667007	82.246521	6.221117	143.375580
82.259995	160.833008	82.266518	10.510324	143.378754
82.279999	162.833008	82.286530	16.243114	143.381836
82.300003	161.667007	82.306534	22.403349	143.384674
82.320000	168.833008	82.326530	27.045885	143.387360
82.340004	166.667007	82.346535	28.266197	143.389832
82.360001	174.667007	82.366531	25.591417	143.392151
82.380005	158.167007	82.386536	20.344021	143.394287
82.400002	158.167007	82.406532	14.542336	143.396271

82.419998	141.500000	82.426529	9.605042	143.398041
82.440002	143.167007	82.446533	6.002886	143.399628
82.459999	139.333008	82.466530	3.615773	143.401062
82.480003	138.333008	82.486534	2.133478	143.402283
82.500000	134.667007	82.506531	1.270694	143.403351
82.519997	134.333008	82.526527	0.822301	143.404205
82.540001	128.667007	82.546532	0.672012	143.404938
82.559998	130.833008	82.566528	0.791080	143.405426
82.580002	135.500000	82.586533	1.248383	143.405731
82.599998	136.333008	82.606529	2.234577	143.405884
82.619995	152.333008	82.626526	4.085072	143.405853
82.639999	142.000000	82.646530	7.241208	143.405640
82.659996	152.833008	82.666527	12.034689	143.405243
82.680000	161.667007	82.686531	18.249535	143.404633
82.699997	170.833008	82.706528	24.641006	143.403900
82.719994	155.167007	82.726524	29.087696	143.402924
82.739998	163.000000	82.746536	29.737726	143.401794
82.759995	164.667007	82.766533	26.398626	143.400482
82.779999	161.167007	82.786537	20.658466	143.398956
82.800003	152.833008	82.806541	14.603196	143.397278
82.820000	149.000000	82.826538	9.579648	143.395416
82.840004	139.500000	82.846542	5.963124	143.393341
82.860001	136.000000	82.866539	3.581105	143.391113
82.880005	143.500000	82.886543	2.103360	143.388702
82.900002	140.167007	82.906540	1.237327	143.386078
82.919998	132.833008	82.926537	0.772017	143.383270
82.940002	138.833008	82.946541	0.580597	143.380310
82.959999	136.667007	82.966537	0.613468	143.377136
82.980003	145.000000	82.986542	0.901235	143.373810
83.000000	143.500000	83.006538	1.575157	143.370270
83.019997	143.833008	83.026535	2.905251	143.366577
83.040001	148.000000	83.046539	5.332804	143.362671
83.059998	160.333008	83.066536	9.422328	143.358612
83.080002	165.667007	83.086540	15.635867	143.354340
83.099998	183.667007	83.106537	23.850647	143.349884
83.119995	192.833008	83.126534	32.927059	143.345245
83.139999	203.167007	83.146538	40.846760	143.340424
83.159996	212.500000	83.166534	45.653130	143.335419

83.180000	225.833008	83.186539	46.464058	143.330231
83.199997	216.000000	83.206535	43.665291	143.324860
83.219994	207.500000	83.226540	38.660976	143.319275
83.239998	208.500000	83.246544	33.742382	143.313568
83.259995	217.500000	83.266541	31.812651	143.307648
83.279999	225.833008	83.286545	35.796844	143.301514
83.300003	246.500000	83.306549	47.936237	143.295227
83.320000	295.500000	83.326546	68.645851	143.288727
83.340004	347.166992	83.346550	94.772812	143.282074
83.360001	360.832977	83.366547	118.591141	143.275238
83.380005	370.832977	83.386551	130.489792	143.268219
83.400002	373.332977	83.406548	124.983452	143.260986
83.419998	348.166992	83.426544	104.833260	143.253601
83.440002	298.832977	83.446548	78.439651	143.246002
83.459999	260.332977	83.466545	53.715454	143.238251
83.480003	225.167007	83.486549	34.498581	143.230316
83.500000	199.833008	83.506546	21.172955	143.222198
83.519997	178.500000	83.526543	12.556459	143.213898
83.540001	168.167007	83.546547	7.243901	143.205414
83.559998	167.667007	83.566544	4.090142	143.196716
83.580002	150.000000	83.586548	2.272828	143.187836
83.599998	156.500000	83.606544	1.251539	143.178802
83.619995	147.333008	83.626541	0.687180	143.169617
83.639999	143.000000	83.646545	0.378372	143.160217
83.659996	134.833008	83.666542	0.210084	143.150635
83.680000	132.500000	83.686554	0.118047	143.140869
83.699997	136.833008	83.706551	0.067453	143.130920
83.719994	135.333008	83.726547	0.039378	143.120819
83.739998	132.167007	83.746552	0.023702	143.110535
83.759995	125.667000	83.766548	0.015018	143.100037
83.779999	133.333008	83.786552	0.010433	143.089386
83.800003	128.667007	83.806557	0.008482	143.078552
83.820000	128.667007	83.826553	0.008542	143.067535
83.840004	128.333008	83.846558	0.010605	143.056335
83.860001	127.333000	83.866554	0.015262	143.044952
83.880005	133.333008	83.886559	0.023924	143.033417
83.900002	128.667007	83.906555	0.039322	143.021698
83.919998	129.667007	83.926552	0.066582	143.009796

83.940002	128.833008	83.946556	0.115311	142.997711
83.959999	136.500000	83.966553	0.203571	142.985443
83.980003	133.000000	83.986557	0.366061	142.973022
84.000000	134.667007	84.006554	0.669511	142.960419
84.019997	141.833008	84.026550	1.243610	142.947662
84.040001	135.833008	84.046555	2.338748	142.934692
84.059998	147.167007	84.066551	4.425094	142.921570
84.080002	157.500000	84.086555	8.353844	142.908234
84.099998	169.500000	84.106552	15.541812	142.894775
84.119995	201.833008	84.126549	28.087078	142.881134
84.139999	235.833008	84.146553	48.485287	142.867310
84.159996	277.000000	84.166557	78.441727	142.853302
84.180000	328.832977	84.186562	116.641808	142.839142
84.199997	391.332977	84.206558	156.614426	142.824799
84.219994	435.666992	84.226555	187.761307	142.810333
84.239998	428.832977	84.246559	200.976898	142.795654
84.259995	427.332977	84.266556	194.705887	142.780792
84.279999	415.500000	84.286560	174.589783	142.765778
84.300003	354.500000	84.306564	147.509521	142.750610
84.320000	320.332977	84.326561	117.812874	142.735229
84.340004	288.666992	84.346565	88.335548	142.719727
84.360001	262.832977	84.366562	61.966049	142.704041
84.380005	224.667007	84.386566	40.890869	142.688202
84.400002	211.000000	84.406563	25.697321	142.672180
84.419998	197.667007	84.426559	15.562789	142.656006
84.440002	175.000000	84.446564	9.160509	142.639679
84.459999	169.500000	84.466560	5.273590	142.623169
84.480003	159.833008	84.486565	2.982571	142.606506
84.500000	151.500000	84.506561	1.666541	142.589661
84.519997	152.167007	84.526558	0.925081	142.572662
84.540001	139.000000	84.546562	0.513079	142.555511
84.559998	139.167007	84.566559	0.286184	142.538177
84.580002	133.333008	84.586563	0.161537	142.520721
84.599998	129.333008	84.606560	0.093253	142.503082
84.619995	130.833008	84.626564	0.056267	142.485260
84.639999	130.667007	84.646568	0.037451	142.467316
84.659996	132.000000	84.666565	0.030538	142.449188
84.680000	123.000000	84.686569	0.033837	142.430939

84.699997	130.167007	84.706566	0.049756	142.412537
84.719994	127.833000	84.726562	0.085859	142.393921
84.739998	131.000000	84.746567	0.157303	142.375214
84.759995	126.000000	84.766563	0.289449	142.356323
84.779999	139.833008	84.786568	0.517654	142.337250
84.800003	135.667007	84.806572	0.875904	142.318085
84.820000	133.167007	84.826569	1.367441	142.298737
84.840004	138.000000	84.846573	1.924963	142.279236
84.860001	136.167007	84.866570	2.395694	142.259613
84.880005	136.000000	84.886574	2.603678	142.239807
84.900002	132.833008	84.906570	2.465081	142.219879
84.919998	132.500000	84.926567	2.052418	142.199768
84.940002	133.500000	84.946571	1.532661	142.179535
84.959999	131.833008	84.966568	1.053194	142.159149
84.980003	125.333000	84.986572	0.682338	142.138611
85.000000	129.500000	85.006569	0.425427	142.117950
85.019997	130.167007	85.026566	0.260261	142.097137
85.040001	125.500000	85.046570	0.161495	142.076172
85.059998	127.500000	85.066566	0.109602	142.055084
85.080002	128.000000	85.086578	0.092869	142.033783
85.099998	124.667000	85.106575	0.108440	142.012421
85.119995	128.833008	85.126572	0.164281	141.990936
85.139999	133.333008	85.146576	0.284663	141.969238
85.159996	121.167000	85.166573	0.521072	141.947449
85.180000	131.333008	85.186577	0.972437	141.925507
85.199997	136.333008	85.206573	1.812914	141.903412
85.219994	142.000000	85.226570	3.320439	141.881226
85.239998	145.333008	85.246574	5.871955	141.858887
85.259995	143.833008	85.266571	9.848798	141.836426
85.279999	153.833008	85.286575	15.455371	141.813812
85.300003	158.833008	85.306580	22.565903	141.791046
85.320000	174.333008	85.326576	30.932266	141.768219
85.340004	186.833008	85.346581	40.792519	141.745209
85.360001	201.000000	85.366577	53.079292	141.722076
85.380005	221.333008	85.386581	68.309761	141.698792
85.400002	227.833008	85.406578	84.465523	141.675415
85.419998	233.833008	85.426575	96.398125	141.651917
85.440002	243.667007	85.446579	98.517616	141.628265

85.459999	240.333008	85.466576	89.087570	141.604523
85.480003	228.000000	85.486580	71.669342	141.580658
85.500000	191.833008	85.506577	52.237892	141.556671
85.519997	186.833008	85.526573	35.310364	141.532562
85.540001	170.500000	85.546585	22.602446	141.508301
85.559998	159.167007	85.566582	13.918642	141.483948
85.580002	155.833008	85.586586	8.326330	141.459442
85.599998	146.500000	85.606583	4.896056	141.434875
85.619995	144.500000	85.626579	2.899747	141.410187
85.639999	133.833008	85.646584	1.844030	141.385345
85.659996	142.000000	85.666580	1.441970	141.360443
85.680000	137.167007	85.686584	1.579442	141.335388
85.699997	126.333000	85.706581	2.284525	141.310242
85.719994	138.167007	85.726578	3.672068	141.284973
85.739998	140.833008	85.746582	5.824116	141.259613
85.759995	147.000000	85.766579	8.583747	141.234131
85.779999	146.333008	85.786583	11.379609	141.208527
85.800003	140.333008	85.806587	13.301951	141.182831
85.820000	139.000000	85.826584	13.585773	141.157043
85.840004	140.667007	85.846588	12.152658	141.131134
85.860001	136.167007	85.866585	9.654032	141.105164
85.880005	136.500000	85.886589	6.962502	141.079041
85.900002	143.833008	85.906586	4.673021	141.052826
85.919998	144.333008	85.926582	2.978714	141.026581
85.940002	132.833008	85.946587	1.827105	141.000153
85.959999	134.000000	85.966583	1.087103	140.973663
85.980003	135.333008	85.986588	0.630404	140.947083
86.000000	133.167007	86.006592	0.358183	140.920380
86.019997	122.500000	86.026588	0.200611	140.893616
86.040001	130.000000	86.046593	0.111406	140.866730
86.059998	121.667000	86.066589	0.061751	140.839783
86.080002	127.333000	86.086594	0.034338	140.812744
86.099998	132.500000	86.106590	0.019252	140.785614
86.119995	129.000000	86.126587	0.010917	140.758453
86.139999	132.333008	86.146591	0.006274	140.731110
86.159996	129.667007	86.166588	0.003662	140.703735
86.180000	132.167007	86.186592	0.002170	140.676300
86.199997	140.000000	86.206589	0.001307	140.648712

86.219994	126.833000	86.226585	0.000800	140.621124
86.239998	133.333008	86.246590	0.000497	140.593414
86.259995	131.167007	86.266586	0.000313	140.565613
86.279999	128.333008	86.286591	0.000200	140.537750
86.300003	125.667000	86.306595	0.000130	140.509796
86.320000	138.000000	86.326591	0.000086	140.481812
86.340004	138.167007	86.346596	0.000057	140.453735
86.360001	142.000000	86.366592	0.000039	140.425568
86.380005	141.500000	86.386597	0.000027	140.397369
86.400002	144.000000	86.406593	0.000019	140.369110
86.419998	137.667007	86.426590	0.000014	140.340729
86.440002	141.000000	86.446594	0.000011	140.312317
86.459999	140.667007	86.466599	0.000009	140.283813
86.480003	147.167007	86.486603	0.000008	140.255249
86.500000	145.667007	86.506599	0.000008	140.226685
86.519997	144.833008	86.526596	0.000009	140.197968
86.540001	151.333008	86.546600	0.000011	140.169281
86.559998	154.000000	86.566597	0.000013	140.140503
86.580002	150.500000	86.586601	0.000017	140.111664
86.599998	147.667007	86.606598	0.000022	140.082764
86.619995	154.500000	86.626595	0.000030	140.053833
86.639999	138.000000	86.646599	0.000040	140.024811
86.659996	145.500000	86.666595	0.000055	139.995758
86.680000	141.833008	86.686600	0.000077	139.966644
86.699997	146.500000	86.706596	0.000108	139.937531
86.719994	141.167007	86.726593	0.000153	139.908356
86.739998	144.667007	86.746597	0.000219	139.879120
86.759995	147.333008	86.766594	0.000316	139.849823
86.779999	139.667007	86.786598	0.000463	139.820496
86.800003	146.000000	86.806602	0.000684	139.791138
86.820000	141.833008	86.826599	0.001023	139.761749
86.840004	146.333008	86.846603	0.001549	139.732330
86.860001	136.167007	86.866600	0.002377	139.702850
86.880005	128.000000	86.886604	0.003696	139.673340
86.900002	140.333008	86.906601	0.005826	139.643799
86.919998	132.333008	86.926605	0.009320	139.614197
86.940002	140.667007	86.946609	0.015133	139.584595
86.959999	137.667007	86.966606	0.024946	139.554962

86.980003	129.000000	86.986610	0.041780	139.525330
87.000000	133.167007	87.006607	0.071083	139.495636
87.019997	137.667007	87.026604	0.122909	139.465942
87.040001	134.667007	87.046608	0.216051	139.436218
87.059998	136.833008	87.066605	0.385819	139.406464
87.080002	135.333008	87.086609	0.699583	139.376678
87.099998	146.000000	87.106606	1.284526	139.346893
87.119995	147.833008	87.126602	2.378798	139.317108
87.139999	157.833008	87.146606	4.414458	139.287262
87.159996	156.167007	87.166603	8.129301	139.257477
87.180000	177.500000	87.186607	14.690909	139.227600
87.199997	199.667007	87.206604	25.699293	139.197754
87.219994	234.333008	87.226601	42.920029	139.167908
87.239998	276.500000	87.246605	67.504112	139.138031
87.259995	327.500000	87.266602	98.795219	139.108124
87.279999	381.666992	87.286606	133.749298	139.078247
87.300003	424.832977	87.306610	167.828384	139.048370
87.320000	449.832977	87.326607	197.084000	139.018494
87.340004	488.000000	87.346611	218.687119	138.988586
87.360001	487.166992	87.366615	229.184616	138.958710
87.380005	467.666992	87.386620	224.543518	138.928833
87.400002	451.666992	87.406616	203.902252	138.898926
87.419998	407.500000	87.426613	172.217102	138.869110
87.440002	376.000000	87.446617	137.368301	138.839233
87.459999	319.000000	87.466614	105.174141	138.809387
87.480003	295.332977	87.486618	77.701515	138.779510
87.500000	259.832977	87.506615	55.142300	138.749725
87.519997	232.167007	87.526611	37.393074	138.719910
87.540001	206.167007	87.546616	24.259527	138.690094
87.559998	192.833008	87.566612	15.170078	138.660339
87.580002	175.167007	87.586617	9.217445	138.630585
87.599998	159.000000	87.606613	5.483150	138.600830
87.619995	152.333008	87.626610	3.215744	138.571136
87.639999	156.333008	87.646614	1.882215	138.541443
87.659996	150.500000	87.666611	1.133066	138.511780
87.680000	140.000000	87.686615	0.753014	138.482117
87.699997	140.667007	87.706612	0.627982	138.452545
87.719994	138.667007	87.726608	0.724552	138.422943

87.739998	144.167007	87.746613	1.092040	138.393402
87.759995	142.000000	87.766609	1.890138	138.363861
87.779999	140.667007	87.786613	3.456950	138.334412
87.800003	146.000000	87.806618	6.419903	138.304962
87.820000	152.500000	87.826622	11.834933	138.275543
87.840004	164.500000	87.846626	21.243721	138.246185
87.860001	198.667007	87.866623	36.381035	138.216858
87.880005	228.667007	87.886627	58.207165	138.187561
87.900002	257.000000	87.906624	85.130424	138.158356
87.919998	280.500000	87.926620	111.662720	138.129150
87.940002	301.000000	87.946625	129.470062	138.100037
87.959999	307.500000	87.966621	131.783890	138.070953
87.980003	294.666992	87.986626	118.127586	138.041901
88.000000	261.500000	88.006622	94.517616	138.012909
88.019997	232.833008	88.026619	68.946632	137.984009
88.040001	214.667007	88.046623	46.922478	137.955139
88.059998	192.500000	88.066620	30.385118	137.926331
88.080002	168.333008	88.086624	18.953526	137.897552
88.099998	163.333008	88.106621	11.475948	137.868896
88.119995	157.333008	88.126617	6.782057	137.840240
88.139999	153.000000	88.146622	3.943821	137.811676
88.159996	147.333008	88.166618	2.295877	137.783203
88.180000	151.167007	88.186623	1.390755	137.754761
88.199997	142.667007	88.206619	0.956717	137.726410
88.219994	151.000000	88.226616	0.854011	137.698120
88.239998	151.833008	88.246620	1.053702	137.669922
88.259995	148.833008	88.266624	1.640722	137.641754
88.279999	152.333008	88.286629	2.854815	137.613678
88.300003	152.667007	88.306633	5.185082	137.585724
88.320000	174.833008	88.326630	9.535044	137.557800
88.340004	191.667007	88.346634	17.473625	137.529968
88.360001	222.500000	88.366631	31.467028	137.502228
88.380005	270.666992	88.386635	54.882103	137.474579
88.400002	326.500000	88.406631	91.030937	137.446991
88.419998	388.166992	88.426628	140.736115	137.419525
88.440002	443.666992	88.446632	198.617584	137.392120
88.459999	498.000000	88.466629	251.049973	137.364807
88.480003	513.333008	88.486633	280.503967	137.337616

88.500000	511.832977	88.506630	275.690796	137.310486
88.519997	485.000000	88.526627	239.674255	137.283478
88.540001	421.000000	88.546631	187.146561	137.256531
88.559998	379.500000	88.566628	134.178680	137.229706
88.580002	324.332977	88.586632	90.310905	137.203003
88.599998	279.166992	88.606628	58.081215	137.176361
88.619995	240.833008	88.626625	36.065712	137.149841
88.639999	218.167007	88.646629	21.743210	137.123444
88.659996	205.333008	88.666626	12.784241	137.097137
88.680000	182.500000	88.686630	7.361031	137.070923
88.699997	185.333008	88.706627	4.175134	137.044891
88.719994	175.667007	88.726631	2.345884	137.018890
88.739998	154.167007	88.746635	1.313866	136.993011
88.759995	154.333008	88.766632	0.737670	136.967255
88.779999	149.167007	88.786636	0.416877	136.941650
88.800003	138.000000	88.806641	0.238000	136.916107
88.820000	132.500000	88.826637	0.137622	136.890747
88.840004	125.667000	88.846642	0.080685	136.865479
88.860001	129.167007	88.866638	0.048017	136.840363
88.880005	120.833000	88.886642	0.029002	136.815308
88.900002	126.167000	88.906639	0.017787	136.790466
88.919998	128.000000	88.926636	0.011075	136.765686
88.940002	137.833008	88.946640	0.007002	136.741028
88.959999	129.333008	88.966637	0.004504	136.716553
88.980003	129.333008	88.986641	0.002957	136.692200
89.000000	124.500000	89.006638	0.001999	136.667938
89.019997	124.667000	89.026634	0.001417	136.643860
89.040001	131.167007	89.046638	0.001086	136.619934
89.059998	132.000000	89.066635	0.000945	136.596100
89.080002	124.000000	89.086639	0.000969	136.572388
89.099998	132.833008	89.106636	0.001174	136.548889
89.119995	131.000000	89.126633	0.001622	136.525513
89.139999	128.500000	89.146637	0.002437	136.502258
89.159996	123.500000	89.166641	0.003851	136.479156
89.180000	127.333000	89.186646	0.006280	136.456207
89.199997	132.000000	89.206642	0.010469	136.433441
89.219994	135.667007	89.226639	0.017767	136.410797
89.239998	130.833008	89.246643	0.030652	136.388275

89.259995	137.000000	89.266640	0.053691	136.365967
89.279999	130.000000	89.286644	0.095502	136.343811
89.300003	137.333008	89.306648	0.172394	136.321777
89.320000	137.167007	89.326645	0.315343	136.299896
89.340004	132.333008	89.346649	0.583063	136.278198
89.360001	138.500000	89.366646	1.083128	136.256714
89.380005	128.500000	89.386650	2.004103	136.235352
89.400002	127.167000	89.406647	3.644285	136.214142
89.419998	127.500000	89.426643	6.407714	136.193115
89.440002	141.000000	89.446648	10.689583	136.172241
89.459999	143.333008	89.466644	16.570513	136.151550
89.480003	145.000000	89.486649	23.404394	136.131042
89.500000	144.667007	89.506645	29.596071	136.110687
89.519997	148.167007	89.526642	33.114948	136.090515
89.540001	142.500000	89.546646	32.651360	136.070526
89.559998	143.333008	89.566643	28.531107	136.050720
89.580002	139.500000	89.586647	22.427708	136.031097
89.599998	136.833008	89.606651	16.202696	136.011627
89.619995	136.167007	89.626648	10.998471	135.992371
89.639999	124.167000	89.646652	7.134206	135.973267
89.659996	126.667000	89.666649	4.469909	135.954376
89.680000	127.000000	89.686653	2.719455	135.935669
89.699997	134.000000	89.706650	1.613920	135.917145
89.719994	121.167000	89.726646	0.939299	135.898834
89.739998	129.833008	89.746651	0.541006	135.880676
89.759995	123.167000	89.766647	0.314389	135.862732
89.779999	129.000000	89.786652	0.192693	135.845001
89.800003	125.000000	89.806656	0.137756	135.827423
89.820000	124.833000	89.826653	0.132191	135.810059
89.840004	119.500000	89.846657	0.175399	135.792938
89.860001	126.167000	89.866653	0.282119	135.775970
89.880005	135.667007	89.886658	0.481166	135.759186
89.900002	127.000000	89.906654	0.807850	135.742645
89.919998	124.500000	89.926651	1.283604	135.726288
89.940002	129.500000	89.946655	1.879942	135.710144
89.959999	127.000000	89.966652	2.485854	135.694214
89.980003	127.333000	89.986656	2.924505	135.678497
90.000000	125.667000	90.006653	3.037345	135.662994

90.019997	127.500000	90.026649	2.789121	135.647675
90.040001	124.500000	90.046661	2.289554	135.632599
90.059998	128.000000	90.066658	1.712999	135.617706
90.080002	128.833008	90.086662	1.193997	135.603058
90.099998	127.500000	90.106659	0.790763	135.588593
90.119995	125.833000	90.126656	0.504059	135.574371
90.139999	119.500000	90.146660	0.311370	135.560333
90.159996	119.833000	90.166656	0.187211	135.546570
90.180000	119.500000	90.186661	0.109928	135.532990
90.199997	123.167000	90.206657	0.063361	135.519653
90.219994	120.000000	90.226654	0.036047	135.506561
90.239998	118.833000	90.246658	0.020362	135.493683
90.259995	116.333000	90.266655	0.011498	135.481018
90.279999	124.333000	90.286659	0.006529	135.468597
90.300003	124.833000	90.306664	0.003756	135.456390
90.320000	116.167000	90.326660	0.002213	135.444427
90.340004	113.167000	90.346664	0.001361	135.432678
90.360001	120.000000	90.366661	0.000907	135.421173
90.380005	121.000000	90.386665	0.000692	135.409882
90.400002	128.333008	90.406662	0.000639	135.398895
90.419998	122.667000	90.426659	0.000719	135.388062
90.440002	123.667000	90.446663	0.000941	135.377533
90.459999	119.333000	90.466660	0.001347	135.367188
90.480003	122.333000	90.486671	0.002028	135.357117
90.500000	118.833000	90.506668	0.003144	135.347260
90.519997	118.000000	90.526665	0.004973	135.337677
90.540001	118.667000	90.546669	0.007996	135.328339
90.559998	117.167000	90.566666	0.013048	135.319244
90.580002	119.333000	90.586670	0.021601	135.310394
90.599998	117.167000	90.606667	0.036258	135.301788
90.619995	118.167000	90.626663	0.061723	135.293427
90.639999	122.833000	90.646667	0.106588	135.285370
90.659996	117.833000	90.666664	0.186666	135.277496
90.680000	127.000000	90.686668	0.331675	135.269897
90.699997	129.667007	90.706665	0.597404	135.262543
90.719994	128.500000	90.726662	1.089343	135.255463
90.739998	130.500000	90.746666	2.004464	135.248627
90.759995	138.667007	90.766663	3.696749	135.242035

90.779999	142.333008	90.786667	6.769434	135.235718
90.800003	161.667007	90.806671	12.140113	135.229645
90.820000	183.667007	90.826668	20.964499	135.223877
90.840004	192.667007	90.846672	34.221970	135.218323
90.860001	206.667007	90.866669	51.756126	135.213043
90.880005	216.833008	90.886673	71.194374	135.208038
90.900002	235.500000	90.906670	87.673035	135.203278
90.919998	226.333008	90.926674	95.703461	135.198822
90.940002	236.500000	90.946678	92.397781	135.194580
90.959999	223.333008	90.966675	79.475166	135.190643
90.980003	206.500000	90.986679	61.871841	135.186951
91.000000	193.667007	91.006676	44.535110	135.183533
91.019997	189.000000	91.026672	30.258263	135.180389
91.040001	180.667007	91.046677	19.705488	135.177521
91.059998	160.667007	91.066673	12.415705	135.174927
91.080002	154.667007	91.086678	7.600548	135.172607
91.099998	150.833008	91.106674	4.537713	135.170532
91.119995	149.833008	91.126671	2.652616	135.168762
91.139999	146.833008	91.146675	1.526565	135.167267
91.159996	134.833008	91.166672	0.871805	135.166046
91.180000	134.333008	91.186676	0.499407	135.165100
91.199997	142.667007	91.206673	0.293163	135.164429
91.219994	131.667007	91.226669	0.184729	135.164062
91.239998	138.833008	91.246674	0.137337	135.163910
91.259995	126.333000	91.266670	0.135289	135.164093
91.279999	120.333000	91.286674	0.179263	135.164581
91.300003	122.667000	91.306679	0.286878	135.165283
91.320000	138.167007	91.326675	0.497801	135.166290
91.340004	131.167007	91.346680	0.883026	135.167603
91.360001	137.333008	91.366684	1.553615	135.169159
91.380005	136.000000	91.386688	2.663194	135.171082
91.400002	140.000000	91.406685	4.394782	135.173218
91.419998	144.333008	91.426682	6.937861	135.175659
91.440002	154.667007	91.446686	10.466969	135.178375
91.459999	169.167007	91.466682	15.110505	135.181427
91.480003	180.667007	91.486687	20.847963	135.184723
91.500000	187.500000	91.506683	27.206474	135.188324
91.519997	184.500000	91.526680	32.980148	135.192200

91.540001	190.333008	91.546684	36.417377	135.196411
91.559998	192.833008	91.566681	36.142887	135.200867
91.580002	191.667007	91.586685	32.155479	135.205627
91.599998	181.667007	91.606682	25.870327	135.210663
91.619995	177.667007	91.626678	19.145590	135.216034
91.639999	163.500000	91.646683	13.295382	135.221680
91.659996	153.833008	91.666679	8.815942	135.227631
91.680000	141.500000	91.686684	5.641939	135.233856
91.699997	145.167007	91.706680	3.505851	135.240387
91.719994	145.000000	91.726677	2.121824	135.247192
91.739998	141.000000	91.746681	1.254700	135.254333
91.759995	140.667007	91.766678	0.728420	135.261749
91.779999	138.333008	91.786682	0.417257	135.269470
91.800003	131.667007	91.806694	0.237207	135.277496
91.820000	131.667007	91.826691	0.134673	135.285828
91.840004	131.333008	91.846695	0.076697	135.294434
91.860001	122.833000	91.866692	0.044020	135.303375
91.880005	135.833008	91.886696	0.025544	135.312592
91.900002	130.000000	91.906693	0.015045	135.322113
91.919998	120.167000	91.926689	0.009039	135.331879
91.940002	121.000000	91.946693	0.005594	135.342010
91.959999	124.333000	91.966690	0.003648	135.352448
91.980003	121.500000	91.986694	0.002620	135.363190
92.000000	121.667000	92.006691	0.002221	135.374207
92.019997	127.333000	92.026688	0.002344	135.385529
92.040001	126.167000	92.046692	0.003040	135.397156
92.059998	121.167000	92.066689	0.004531	135.409119
92.080002	120.333000	92.086693	0.007296	135.421356
92.099998	128.500000	92.106689	0.012248	135.433899
92.119995	128.667007	92.126686	0.021090	135.446747
92.139999	123.333000	92.146690	0.036990	135.459900
92.159996	120.500000	92.166687	0.065818	135.473389
92.180000	124.833000	92.186691	0.118495	135.487152
92.199997	131.000000	92.206688	0.214825	135.501221
92.219994	124.333000	92.226685	0.389790	135.515594
92.239998	124.667000	92.246696	0.701989	135.530304
92.259995	134.333008	92.266693	1.240276	135.545288
92.279999	141.667007	92.286697	2.125746	135.560577

92.300003	137.667007	92.306702	3.491165	135.576202
92.320000	145.667007	92.326698	5.429734	135.592102
92.340004	140.167007	92.346703	7.910054	135.608337
92.360001	150.500000	92.366699	10.666519	135.624847
92.380005	158.667007	92.386703	13.157609	135.641693
92.400002	150.667007	92.406700	14.680073	135.658844
92.419998	152.667007	92.426697	14.707278	135.676300
92.440002	151.833008	92.446701	13.226121	135.694061
92.459999	143.000000	92.466698	10.767756	135.712097
92.480003	134.000000	92.486702	8.062649	135.730499
92.500000	142.833008	92.506699	5.661770	135.749176
92.519997	140.333008	92.526695	3.792570	135.768158
92.540001	137.500000	92.546700	2.450784	135.787445
92.559998	131.167007	92.566696	1.537438	135.807068
92.580002	129.333008	92.586700	0.938947	135.826996
92.599998	129.667007	92.606697	0.560155	135.847198
92.619995	128.333008	92.626694	0.327703	135.867737
92.639999	132.833008	92.646698	0.188926	135.888550
92.659996	119.833000	92.666702	0.107966	135.909698
92.680000	130.667007	92.686707	0.061505	135.931183
92.699997	128.500000	92.706703	0.035111	135.952911
92.719994	118.000000	92.726700	0.020167	135.974976
92.739998	116.667000	92.746704	0.011690	135.997345
92.759995	123.167000	92.766701	0.006857	136.020020
92.779999	129.500000	92.786705	0.004075	136.042999
92.800003	132.333008	92.806709	0.002457	136.066284
92.820000	124.500000	92.826706	0.001505	136.089905
92.840004	118.167000	92.846710	0.000938	136.113800
92.860001	116.333000	92.866707	0.000599	136.137970
92.880005	124.833000	92.886711	0.000395	136.162537
92.900002	126.000000	92.906708	0.000276	136.187347
92.919998	127.167000	92.926704	0.000212	136.212463
92.940002	128.667007	92.946709	0.000188	136.237915
92.959999	127.000000	92.966705	0.000198	136.263641
92.980003	123.000000	92.986710	0.000246	136.289673
93.000000	117.833000	93.006706	0.000342	136.316010
93.019997	122.000000	93.026703	0.000511	136.342682
93.040001	127.500000	93.046707	0.000795	136.369629

93.059998	132.167007	93.066704	0.001271	136.396851
93.080002	129.333008	93.086708	0.002069	136.424408
93.099998	126.167000	93.106712	0.003423	136.452271
93.119995	142.000000	93.126709	0.005742	136.480469
93.139999	120.500000	93.146713	0.009766	136.508911
93.159996	131.000000	93.166710	0.016831	136.537659
93.180000	130.000000	93.186714	0.029403	136.566742
93.199997	132.333008	93.206711	0.052039	136.596100
93.219994	132.667007	93.226707	0.093246	136.625732
93.239998	132.833008	93.246712	0.168805	136.655701
93.259995	129.333008	93.266708	0.307189	136.686005
93.279999	128.667007	93.286713	0.557916	136.716522
93.300003	135.667007	93.306717	0.999945	136.747375
93.320000	147.167007	93.326714	1.743234	136.778503
93.340004	144.167007	93.346718	2.908402	136.809967
93.360001	143.833008	93.366714	4.559457	136.841705
93.380005	156.000000	93.386719	6.600436	136.873749
93.400002	169.000000	93.406715	8.683267	136.906036
93.419998	164.167007	93.426712	10.263817	136.938721
93.440002	158.000000	93.446716	10.846311	136.971619
93.459999	152.000000	93.466713	10.281966	137.004791
93.480003	159.333008	93.486717	8.849916	137.038300
93.500000	152.500000	93.506714	7.043194	137.072113
93.519997	151.500000	93.526718	5.278130	137.106171
93.540001	133.000000	93.546722	3.774587	137.140564
93.559998	134.833008	93.566719	2.594394	137.175201
93.580002	137.667007	93.586723	1.719759	137.210144
93.599998	131.667007	93.606720	1.103846	137.245361
93.619995	132.500000	93.626717	0.689110	137.280884
93.639999	133.000000	93.646721	0.420362	137.316711
93.659996	134.500000	93.666718	0.251815	137.352783
93.680000	126.167000	93.686722	0.148819	137.389160
93.699997	129.000000	93.706718	0.087421	137.425781
93.719994	129.500000	93.726715	0.051720	137.462738
93.739998	138.667007	93.746719	0.031724	137.499969
93.759995	132.000000	93.766716	0.021505	137.537445
93.779999	125.000000	93.786720	0.017885	137.575226
93.800003	127.000000	93.806725	0.019745	137.613281

93.820000	123.000000	93.826721	0.027799	137.651642
93.840004	126.000000	93.846725	0.045005	137.690247
93.860001	124.333000	93.866722	0.077769	137.729095
93.880005	124.833000	93.886726	0.138538	137.768280
93.900002	123.667000	93.906723	0.250189	137.807709
93.919998	124.167000	93.926720	0.453154	137.847382
93.940002	129.333008	93.946724	0.814109	137.887360
93.959999	126.500000	93.966728	1.430788	137.927643
93.980003	132.333008	93.986732	2.420959	137.968170
94.000000	140.333008	94.006729	3.874423	138.008942
94.019997	134.667007	94.026726	5.761183	138.049957
94.040001	147.000000	94.046730	7.827228	138.091278
94.059998	147.333008	94.066727	9.579195	138.132904
94.080002	152.167007	94.086731	10.467466	138.174744
94.099998	151.167007	94.106728	10.191749	138.216858
94.119995	148.833008	94.126724	8.898118	138.259247
94.139999	142.167007	94.146729	7.064904	138.301880
94.159996	137.667007	94.166725	5.201711	138.344727
94.180000	139.333008	94.186729	3.620957	138.387848
94.199997	133.500000	94.206726	2.419867	138.431305
94.219994	131.167007	94.226723	1.565880	138.474945
94.239998	128.333008	94.246727	0.984859	138.518890
94.259995	120.833000	94.266724	0.603686	138.563049
94.279999	131.667007	94.286728	0.361553	138.607483
94.300003	124.667000	94.306732	0.212499	138.652130
94.320000	127.667000	94.326729	0.123238	138.697052
94.340004	127.000000	94.346733	0.070908	138.742218
94.360001	128.167007	94.366730	0.040747	138.787628
94.380005	128.333008	94.386742	0.023538	138.833282
94.400002	137.167007	94.406738	0.013830	138.879242
94.419998	128.167007	94.426735	0.008456	138.925323
94.440002	126.000000	94.446739	0.005678	138.971710
94.459999	132.333008	94.466736	0.004631	139.018341
94.480003	122.833000	94.486740	0.005020	139.065216
94.500000	117.167000	94.506737	0.006975	139.112305
94.519997	122.000000	94.526733	0.010951	139.159668
94.540001	124.333000	94.546738	0.017550	139.207245
94.559998	124.167000	94.566734	0.027109	139.255005

94.580002	128.500000	94.586739	0.039084	139.303040
94.599998	130.167007	94.606735	0.051472	139.351318
94.619995	130.000000	94.626732	0.061053	139.399780
94.639999	124.833000	94.646736	0.064736	139.448517
94.659996	127.500000	94.666733	0.061408	139.497467
94.680000	127.500000	94.686737	0.052659	139.546631
94.699997	125.167000	94.706734	0.041701	139.595978
94.719994	127.333000	94.726730	0.031541	139.645599
94.739998	126.167000	94.746735	0.024006	139.695435
94.759995	127.500000	94.766731	0.019971	139.745453
94.779999	130.667007	94.786736	0.020127	139.795715
94.800003	126.833000	94.806747	0.025841	139.846252
94.820000	116.333000	94.826744	0.040006	139.896912
94.840004	126.667000	94.846748	0.068230	139.947784
94.860001	122.000000	94.866745	0.120301	139.998901
94.880005	130.000000	94.886749	0.211732	140.050201
94.900002	126.833000	94.906746	0.363337	140.101715
94.919998	130.500000	94.926743	0.596364	140.153442
94.940002	125.500000	94.946747	0.919298	140.205353
94.959999	124.667000	94.966743	1.307408	140.257446
94.980003	132.333008	94.986748	1.689723	140.309753
95.000000	131.500000	95.006744	1.961430	140.362274
95.019997	130.333008	95.026741	2.033447	140.414978
95.040001	134.833008	95.046745	1.885877	140.467865
95.059998	130.667007	95.066742	1.580249	140.520966
95.080002	139.167007	95.086746	1.216550	140.574249
95.099998	123.667000	95.106743	0.878328	140.627686
95.119995	132.333008	95.126740	0.606350	140.681366
95.139999	128.500000	95.146744	0.406904	140.735199
95.159996	127.667000	95.166740	0.270449	140.789185
95.180000	131.500000	95.186745	0.184217	140.843414
95.199997	126.833000	95.206741	0.138405	140.897797
95.219994	126.167000	95.226746	0.128210	140.952362
95.239998	120.667000	95.246750	0.156062	141.007080
95.259995	132.333008	95.266747	0.235105	141.061951
95.279999	125.667000	95.286751	0.396026	141.117035
95.300003	128.333008	95.306755	0.698481	141.172272
95.320000	134.333008	95.326752	1.248692	141.227692

95.340004	132.167007	95.346756	2.221728	141.283295
95.360001	145.500000	95.366753	3.871232	141.338959
95.380005	149.667007	95.386757	6.502438	141.394867
95.400002	149.333008	95.406754	10.345991	141.450958
95.419998	160.167007	95.426750	15.330332	141.507141
95.440002	164.333008	95.446754	20.822506	141.563507
95.459999	172.667007	95.466751	25.587690	141.620026
95.480003	190.667007	95.486755	28.240831	141.676697
95.500000	186.667007	95.506752	28.015989	141.733490
95.519997	192.833008	95.526749	25.303560	141.790436
95.540001	176.167007	95.546753	21.420256	141.847565
95.559998	177.000000	95.566750	17.849340	141.904785
95.580002	181.667007	95.586754	15.565506	141.962158
95.599998	182.500000	95.606750	14.818263	142.019714
95.619995	172.333008	95.626747	15.207543	142.077332
95.639999	174.167007	95.646759	15.907052	142.135162
95.659996	177.667007	95.666756	16.011713	142.193085
95.680000	167.667007	95.686760	14.971415	142.251129
95.699997	174.667007	95.706757	12.842061	142.309357
95.719994	165.000000	95.726753	10.146558	142.367584
95.739998	156.333008	95.746758	7.486754	142.426056
95.759995	147.667007	95.766754	5.248127	142.484589
95.779999	149.000000	95.786758	3.544804	142.543304
95.800003	140.000000	95.806763	2.330901	142.602081
95.820000	135.167007	95.826759	1.506594	142.660950
95.840004	131.833008	95.846764	0.974697	142.719971
95.860001	141.833008	95.866760	0.660763	142.779083
95.880005	140.500000	95.886765	0.515770	142.838318
95.900002	137.667007	95.906761	0.520765	142.897644
95.919998	140.833008	95.926758	0.693950	142.957092
95.940002	144.333008	95.946762	1.106408	143.016632
95.959999	138.500000	95.966759	1.907620	143.076233
95.980003	146.500000	95.986763	3.364022	143.135956
96.000000	151.333008	96.006760	5.889580	143.195831
96.019997	164.667007	96.026756	10.035246	143.255676
96.040001	171.500000	96.046761	16.347824	143.315704
96.059998	181.833008	96.066765	25.012661	143.375824
96.080002	195.333008	96.086769	35.346775	143.436005

96.099998	202.667007	96.106766	45.457619	143.496246
96.119995	209.500000	96.126762	52.628819	143.556580
96.139999	210.000000	96.146767	54.556149	143.617004
96.159996	207.000000	96.166763	50.725945	143.677460
96.180000	199.667007	96.186768	42.717289	143.738037
96.199997	189.333008	96.206764	33.141449	143.798645
96.219994	175.167007	96.226761	24.202314	143.859375
96.239998	183.167007	96.246765	17.033998	143.920135
96.259995	167.667007	96.266762	11.896444	143.980927
96.279999	164.833008	96.286766	8.649403	144.041840
96.300003	168.667007	96.306770	7.136194	144.102783
96.320000	182.000000	96.326767	7.344775	144.163788
96.340004	183.000000	96.346771	9.444020	144.224854
96.360001	198.167007	96.366768	13.695102	144.285919
96.380005	216.333008	96.386772	20.225502	144.347076
96.400002	214.167007	96.406769	28.679153	144.408295
96.419998	234.000000	96.426765	38.032871	144.469513
96.440002	258.000000	96.446770	46.906971	144.530823
96.459999	268.166992	96.466766	54.486374	144.592133
96.480003	289.000000	96.486778	61.473248	144.653503
96.500000	308.832977	96.506775	69.940483	144.714905
96.519997	318.832977	96.526772	81.867012	144.776306
96.540001	343.166992	96.546776	97.201035	144.837799
96.559998	361.000000	96.566772	113.073593	144.899261
96.580002	366.666992	96.586777	125.126991	144.960785
96.599998	380.500000	96.606773	130.069199	145.022308
96.619995	394.500000	96.626770	127.453728	145.083832
96.639999	372.000000	96.646774	119.021561	145.145386
96.659996	359.666992	96.666771	106.725021	145.206940
96.680000	335.166992	96.686775	91.690338	145.268524
96.699997	306.666992	96.706772	74.873528	145.330109
96.719994	285.000000	96.726768	57.782063	145.391724
96.739998	257.166992	96.746773	42.211781	145.453308
96.759995	236.667007	96.766769	29.439358	145.514923
96.779999	222.333008	96.786774	19.796118	145.576569
96.800003	204.833008	96.806778	12.940053	145.638153
96.820000	194.833008	96.826775	8.262913	145.699707
96.840004	179.667007	96.846779	5.169562	145.761292

96.860001	174.500000	96.866776	3.187468	145.822906
96.880005	166.167007	96.886780	1.963200	145.884430
96.900002	165.000000	96.906784	1.251134	145.945984
96.919998	155.833008	96.926781	0.891695	146.007477
96.940002	151.167007	96.946785	0.796358	146.068970
96.959999	151.333008	96.966782	0.935364	146.130463
96.980003	150.667007	96.986786	1.325792	146.191925
97.000000	150.000000	97.006783	2.019870	146.253326
97.019997	154.167007	97.026779	3.095940	146.314697
97.040001	159.333008	97.046783	4.652416	146.376068
97.059998	166.167007	97.066780	6.794193	146.437347
97.080002	171.000000	97.086784	9.587600	146.498596
97.099998	163.333008	97.106781	12.927464	146.559845
97.119995	162.667007	97.126778	16.381315	146.621002
97.139999	178.833008	97.146782	19.159046	146.682159
97.159996	181.000000	97.166779	20.390453	146.743256
97.180000	178.000000	97.186783	19.623287	146.804291
97.199997	178.667007	97.206779	17.125364	146.865265
97.219994	169.500000	97.226776	13.707166	146.926178
97.239998	164.500000	97.246780	10.234065	146.987061
97.259995	159.833008	97.266777	7.261043	147.047791
97.279999	148.667007	97.286781	4.970658	147.108521
97.300003	149.000000	97.306786	3.323580	147.169189
97.320000	139.833008	97.326790	2.201991	147.229797
97.340004	146.000000	97.346794	1.490390	147.290344
97.360001	138.333008	97.366791	1.104320	147.350800
97.380005	134.667007	97.386795	1.001521	147.411163
97.400002	147.000000	97.406792	1.196315	147.471405
97.419998	140.167007	97.426788	1.782975	147.531616
97.440002	146.167007	97.446793	2.975479	147.591705
97.459999	150.667007	97.466789	5.157927	147.651703
97.480003	168.000000	97.486794	8.934235	147.711609
97.500000	167.000000	97.506790	15.096855	147.771484
97.519997	184.000000	97.526787	24.430479	147.831177
97.540001	208.500000	97.546791	37.217793	147.890839
97.559998	215.833008	97.566788	52.501896	147.950378
97.580002	231.167007	97.586792	67.633003	148.009827
97.599998	243.333008	97.606789	78.697403	148.069122

97.619995	250.167007	97.626785	82.261993	148.128326
97.639999	238.667007	97.646790	77.322655	148.187408
97.659996	224.667007	97.666786	65.911057	148.246368
97.680000	214.167007	97.686790	51.712223	148.305267
97.699997	198.833008	97.706787	38.045254	148.364014
97.719994	181.333008	97.726784	26.703838	148.422607
97.739998	164.333008	97.746796	18.098295	148.481171
97.759995	165.833008	97.766792	11.928905	148.539490
97.779999	153.333008	97.786797	7.667933	148.597748
97.800003	151.167007	97.806801	4.827127	148.655853
97.820000	152.167007	97.826797	3.006530	148.713837
97.840004	149.333008	97.846802	1.900086	148.771667
97.860001	157.333008	97.866798	1.295099	148.829346
97.880005	148.833008	97.886803	1.063987	148.886902
97.900002	145.500000	97.906799	1.165412	148.944305
97.919998	149.667007	97.926796	1.649973	149.001526
97.940002	155.667007	97.946800	2.677405	149.058655
97.959999	154.500000	97.966797	4.531897	149.115631
97.980003	158.833008	97.986801	7.614547	149.172424
98.000000	172.500000	98.006798	12.340987	149.229004
98.019997	173.667007	98.026794	18.907129	149.285522
98.040001	182.167007	98.046799	26.918770	149.341827
98.059998	188.167007	98.066795	35.087658	149.397949
98.080002	189.333008	98.086800	41.412174	149.453949
98.099998	188.333008	98.106796	43.970406	149.509766
98.119995	188.500000	98.126793	42.004208	149.565338
98.139999	186.000000	98.146797	36.362469	149.620819
98.159996	173.000000	98.166801	28.930265	149.676117
98.180000	173.500000	98.186806	21.546354	149.731232
98.199997	157.167007	98.206802	15.306931	149.786133
98.219994	158.000000	98.226799	10.537222	149.840851
98.239998	151.833008	98.246803	7.129977	149.895416
98.259995	149.333008	98.266800	4.850915	149.949738
98.279999	155.333008	98.286804	3.487333	150.003937
98.300003	153.333008	98.306808	2.917290	150.057922
98.320000	169.500000	98.326805	3.143456	150.111664
98.340004	165.167007	98.346809	4.342930	150.165253
98.360001	166.667007	98.366806	6.926728	150.218597

98.380005	184.667007	98.386810	11.590583	150.271759
98.400002	198.500000	98.406807	19.260654	150.324677
98.419998	219.000000	98.426804	30.834566	150.377441
98.440002	244.167007	98.446808	46.570812	150.429993
98.459999	251.000000	98.466805	65.213860	150.482269
98.480003	265.166992	98.486809	83.501511	150.534393
98.500000	278.832977	98.506805	96.735359	150.586273
98.519997	265.500000	98.526802	100.874001	150.637909
98.540001	274.832977	98.546806	94.801773	150.689362
98.559998	258.166992	98.566811	80.989906	150.740570
98.580002	235.333008	98.586815	63.875397	150.791565
98.599998	217.500000	98.606812	47.456615	150.842346
98.619995	205.833008	98.626808	33.945148	150.892853
98.639999	206.167007	98.646812	23.918129	150.943146
98.659996	195.667007	98.666809	17.076462	150.993195
98.680000	182.333008	98.686813	12.802827	151.043030
98.699997	177.500000	98.706810	10.413510	151.092560
98.719994	179.333008	98.726807	9.193767	151.141907
98.739998	177.500000	98.746811	8.463064	151.191010
98.759995	173.500000	98.766808	7.701854	151.239777
98.779999	166.333008	98.786812	6.671401	151.288361
98.800003	159.500000	98.806816	5.416604	151.336700
98.820000	155.000000	98.826813	4.130420	151.384796
98.840004	149.833008	98.846817	2.990527	151.432617
98.860001	141.833008	98.866814	2.082681	151.480164
98.880005	148.000000	98.886818	1.407651	151.527466
98.900002	141.667007	98.906815	0.927623	151.574493
98.919998	131.833008	98.926811	0.596678	151.621246
98.940002	136.500000	98.946815	0.374866	151.667755
98.959999	137.500000	98.966812	0.230572	151.714020
98.980003	134.667007	98.986824	0.139271	151.759979
99.000000	135.000000	99.006821	0.083089	151.805695
99.019997	139.833008	99.026817	0.049248	151.851074
99.040001	137.333008	99.046822	0.029219	151.896210
99.059998	136.667007	99.066818	0.017555	151.941071
99.080002	127.833000	99.086823	0.010884	151.985626
99.099998	134.000000	99.106819	0.007222	152.029907
99.119995	129.500000	99.126816	0.005442	152.073914

99.139999	135.500000	99.146820	0.004974	152.117645
99.159996	133.167007	99.166817	0.005618	152.161072
99.180000	137.333008	99.186821	0.007481	152.204193
99.199997	135.000000	99.206818	0.010990	152.247040
99.219994	128.667007	99.226814	0.017012	152.289581
99.239998	124.500000	99.246819	0.027110	152.331818
99.259995	136.167007	99.266815	0.043995	152.373810
99.279999	135.833008	99.286819	0.072412	152.415436
99.300003	137.500000	99.306824	0.120645	152.456818
99.320000	136.833008	99.326820	0.203302	152.497833
99.340004	133.833008	99.346825	0.346563	152.538574
99.360001	139.167007	99.366821	0.597181	152.579041
99.380005	141.667007	99.386833	1.039813	152.619171
99.400002	152.833008	99.406830	1.823721	152.658966
99.419998	148.500000	99.426826	3.209501	152.698456
99.440002	155.667007	99.446831	5.630731	152.737640
99.459999	168.833008	99.466827	9.752398	152.776520
99.480003	189.833008	99.486832	16.489367	152.815094
99.500000	201.000000	99.506828	26.843626	152.853271
99.519997	226.500000	99.526825	41.479164	152.891205
99.540001	258.666992	99.546829	59.960011	152.928802
99.559998	280.500000	99.566826	79.951805	152.966064
99.580002	300.666992	99.586830	97.227043	153.002991
99.599998	314.666992	99.606827	106.970222	153.039612
99.619995	314.332977	99.626823	106.219894	153.075867
99.639999	304.000000	99.646828	95.576309	153.111816
99.659996	272.166992	99.666824	78.782753	153.147430
99.680000	256.500000	99.686829	60.442650	153.182709
99.699997	231.500000	99.706825	43.936977	153.217651
99.719994	213.667007	99.726822	30.710155	153.252289
99.739998	188.833008	99.746826	20.825878	153.286530
99.759995	174.333008	99.766823	13.758885	153.320465
99.779999	163.000000	99.786827	8.862719	153.354065
99.800003	160.333008	99.806839	5.571949	153.387360
99.820000	162.333008	99.826836	3.429512	153.420197
99.840004	158.333008	99.846840	2.074627	153.452789
99.860001	159.000000	99.866837	1.242412	153.485016
99.880005	152.500000	99.886841	0.744443	153.516876

99.900002	154.333008	99.906837	0.455879	153.548340
99.919998	144.167007	99.926834	0.297848	153.579529
99.940002	138.833008	99.946838	0.224865	153.610352
99.959999	148.333008	99.966835	0.215312	153.640778
99.980003	139.667007	99.986839	0.266564	153.670929
100.000000	131.833008	100.006836	0.395349	153.700684
100.019997	139.833008	100.026833	0.644356	153.730042
100.040001	141.333008	100.046837	1.097730	153.759094
100.059998	141.667007	100.066833	1.908469	153.787781
100.080002	147.167007	100.086838	3.345209	153.816071
100.099998	153.667007	100.106834	5.850072	153.844025
100.119995	156.333008	100.126831	10.098378	153.871643
100.139999	163.667007	100.146835	16.993027	153.898895
100.159996	193.500000	100.166832	27.470970	153.925751
100.180000	220.667007	100.186836	42.044193	153.952240
100.199997	245.167007	100.206841	60.013817	153.978394
100.219994	272.166992	100.226837	78.817337	154.004181
100.239998	285.832977	100.246841	94.235840	154.029572
100.259995	285.500000	100.266838	101.877487	154.054596
100.279999	279.500000	100.286842	99.484009	154.079254
100.300003	273.000000	100.306847	88.224976	154.103546
100.320000	263.000000	100.326843	71.915482	154.127502
100.340004	239.500000	100.346848	54.773281	154.151031
100.360001	214.500000	100.366844	39.672390	154.174225
100.380005	205.167007	100.386848	27.708406	154.197021
100.400002	184.500000	100.406853	18.827097	154.219452
100.419998	167.500000	100.426849	12.508206	154.241516
100.440002	163.000000	100.446854	8.170444	154.263214
100.459999	161.667007	100.466850	5.327720	154.284515
100.480003	158.167007	100.486855	3.603391	154.305450
100.500000	168.500000	100.506851	2.746495	154.326019
100.519997	155.000000	100.526848	2.638846	154.346161
100.540001	157.167007	100.546852	3.321148	154.365967
100.559998	160.333008	100.566849	5.021843	154.385376
100.580002	167.167007	100.586853	8.185725	154.404388
100.599998	173.833008	100.606857	13.449020	154.423065
100.619995	181.167007	100.626854	21.492651	154.441315
100.639999	195.000000	100.646858	32.711269	154.459198

100.659996	206.833008	100.666855	46.682865	154.476746
100.680000	209.667007	100.686859	61.812450	154.493866
100.699997	235.667007	100.706856	75.483986	154.510590
100.719994	231.167007	100.726852	85.083862	154.526978
100.739998	241.667007	100.746857	89.208229	154.542969
100.759995	237.500000	100.766853	88.107491	154.558533
100.779999	231.667007	100.786858	82.968750	154.573761
100.800003	224.167007	100.806862	74.886871	154.588593
100.820000	209.500000	100.826859	64.605598	154.603027
100.840004	199.500000	100.846863	52.949875	154.617096
100.860001	187.167007	100.866859	41.131054	154.630737
100.880005	177.333008	100.886864	30.396181	154.644043
100.900002	167.333008	100.906860	21.566862	154.656952
100.919998	163.667007	100.926857	14.841949	154.669464
100.940002	165.333008	100.946861	9.997829	154.681610
100.959999	156.833008	100.966858	6.662620	154.693329
100.980003	152.000000	100.986862	4.480329	154.704742
101.000000	153.667007	101.006859	3.189586	154.715698
101.019997	155.167007	101.026863	2.633648	154.726288
101.040001	149.500000	101.046867	2.772502	154.736481
101.059998	149.667007	101.066864	3.687521	154.746307
101.080002	170.333008	101.086868	5.575968	154.755768
101.099998	158.000000	101.106865	8.691164	154.764801
101.119995	161.000000	101.126862	13.201669	154.773499
101.139999	167.167007	101.146866	18.951820	154.781769
101.159996	169.667007	101.166862	25.214989	154.789673
101.180000	179.667007	101.186867	30.691748	154.797180
101.199997	175.000000	101.206863	33.893925	154.804291
101.219994	173.000000	101.226860	33.872883	154.811066
101.239998	170.333008	101.246864	30.746307	154.817444
101.259995	164.667007	101.266861	25.609121	154.823425
101.279999	163.500000	101.286865	19.872095	154.829010
101.300003	150.500000	101.306870	14.615971	154.834229
101.320000	149.000000	101.326866	10.342763	154.839050
101.340004	155.333008	101.346870	7.106070	154.843536
101.360001	147.667007	101.366867	4.760240	154.847626
101.380005	151.167007	101.386871	3.110718	154.851288
101.400002	143.833008	101.406868	1.984533	154.854584

101.419998	136.667007	101.426872	1.237596	154.857544
101.440002	134.167007	101.446877	0.756926	154.860107
101.459999	146.500000	101.466873	0.456059	154.862274
101.480003	134.667007	101.486877	0.271904	154.864105
101.500000	130.833008	101.506874	0.161262	154.865509
101.519997	139.333008	101.526871	0.095555	154.866577
101.540001	142.333008	101.546875	0.056779	154.867249
101.559998	136.500000	101.566872	0.033952	154.867554
101.580002	139.833008	101.586876	0.020471	154.867523
101.599998	145.000000	101.606873	0.012470	154.867035
101.619995	139.667007	101.626869	0.007680	154.866241
101.639999	140.000000	101.646873	0.004784	154.865082
101.659996	133.167007	101.666870	0.003015	154.863495
101.680000	137.333008	101.686874	0.001922	154.861603
101.699997	143.333008	101.706871	0.001239	154.859283
101.719994	136.333008	101.726868	0.000808	154.856628
101.739998	142.667007	101.746872	0.000533	154.853607
101.759995	135.167007	101.766869	0.000355	154.850220
101.779999	145.333008	101.786873	0.000239	154.846466
101.800003	139.167007	101.806877	0.000163	154.842377
101.820000	138.167007	101.826881	0.000113	154.837860
101.840004	139.833008	101.846886	0.000079	154.833038
101.860001	139.833008	101.866882	0.000057	154.827850
101.880005	141.500000	101.886887	0.000042	154.822296
101.900002	134.167007	101.906883	0.000033	154.816406
101.919998	134.167007	101.926880	0.000028	154.810120
101.940002	126.833000	101.946884	0.000025	154.803497
101.959999	136.500000	101.966881	0.000026	154.796509
101.980003	140.833008	101.986885	0.000029	154.789215
102.000000	144.333008	102.006882	0.000035	154.781525
102.019997	132.000000	102.026878	0.000045	154.773499
102.040001	133.167007	102.046883	0.000060	154.765167
102.059998	133.667007	102.066879	0.000083	154.756409
102.080002	138.833008	102.086884	0.000117	154.747345
102.099998	137.667007	102.106880	0.000168	154.737946
102.119995	132.833008	102.126877	0.000242	154.728210
102.139999	135.500000	102.146881	0.000355	154.718140
102.159996	132.333008	102.166878	0.000524	154.707703

102.180000	138.833008	102.186882	0.000782	154.696960
102.199997	131.667007	102.206879	0.001180	154.685883
102.219994	132.333008	102.226883	0.001799	154.674438
102.239998	141.500000	102.246887	0.002772	154.662659
102.259995	139.333008	102.266884	0.004317	154.650574
102.279999	132.333008	102.286888	0.006794	154.638153
102.300003	128.500000	102.306892	0.010804	154.625427
102.320000	136.667007	102.326889	0.017354	154.612366
102.340004	136.333008	102.346893	0.028168	154.598969
102.360001	146.833008	102.366890	0.046181	154.585297
102.380005	134.333008	102.386894	0.076519	154.571228
102.400002	144.333008	102.406891	0.128107	154.556885
102.419998	137.667007	102.426888	0.216756	154.542267
102.440002	135.167007	102.446892	0.370521	154.527252
102.459999	135.667007	102.466888	0.638525	154.511993
102.480003	133.667007	102.486893	1.105884	154.496399
102.500000	134.667007	102.506889	1.912777	154.480499
102.519997	150.167007	102.526886	3.276837	154.464325
102.540001	150.500000	102.546890	5.501241	154.447845
102.559998	147.333008	102.566887	8.933656	154.431030
102.580002	159.000000	102.586891	13.848954	154.413940
102.599998	167.333008	102.606888	20.210476	154.396515
102.619995	163.333008	102.626892	27.429440	154.378845
102.639999	173.500000	102.646896	34.274033	154.360870
102.659996	183.167007	102.666893	39.217579	154.342590
102.680000	175.500000	102.686897	41.188618	154.324066
102.699997	195.167007	102.706894	40.289806	154.305206
102.719994	196.167007	102.726891	37.977726	154.286102
102.739998	205.833008	102.746895	36.564747	154.266693
102.759995	225.500000	102.766891	38.490349	154.247040
102.779999	236.333008	102.786896	45.773548	154.227081
102.800003	266.500000	102.806900	59.599590	154.206879
102.820000	294.166992	102.826897	79.718956	154.186371
102.840004	300.666992	102.846901	103.719200	154.165619
102.860001	319.332977	102.866898	126.708450	154.144623
102.880005	330.166992	102.886902	142.518417	154.123322
102.900002	325.166992	102.906898	146.232422	154.101776
102.919998	321.000000	102.926895	136.782028	154.079987

102.940002	320.332977	102.946899	117.404823	154.057953
102.959999	289.332977	102.966896	93.667534	154.035675
102.980003	274.832977	102.986900	70.575958	154.013123
103.000000	248.833008	103.006897	51.015301	153.990326
103.019997	220.833008	103.026901	35.763832	153.967285
103.040001	203.667007	103.046906	24.451363	153.944000
103.059998	193.500000	103.066902	16.327112	153.920502
103.080002	181.667007	103.086906	10.643558	153.896759
103.099998	183.833008	103.106903	6.780176	153.872772
103.119995	178.833008	103.126900	4.228861	153.848541
103.139999	170.500000	103.146904	2.591065	153.824158
103.159996	159.167007	103.166901	1.567042	153.799500
103.180000	163.167007	103.186905	0.939566	153.774597
103.199997	167.500000	103.206902	0.561315	153.749542
103.219994	158.500000	103.226898	0.335479	153.724243
103.239998	157.333008	103.246902	0.201275	153.698730
103.259995	149.167007	103.266899	0.121630	153.673004
103.279999	145.167007	103.286903	0.074186	153.647095
103.300003	139.000000	103.306908	0.045787	153.620972
103.320000	140.833008	103.326904	0.028690	153.594635
103.340004	138.000000	103.346909	0.018345	153.568115
103.360001	144.333008	103.366905	0.012110	153.541412
103.380005	142.833008	103.386909	0.008435	153.514496
103.400002	142.833008	103.406906	0.006443	153.487427
103.419998	136.333008	103.426910	0.005667	153.460175
103.440002	137.000000	103.446915	0.005930	153.432709
103.459999	141.333008	103.466911	0.007285	153.405121
103.480003	141.333008	103.486916	0.010036	153.377319
103.500000	137.667007	103.506912	0.014812	153.349365
103.519997	142.000000	103.526909	0.022738	153.321259
103.540001	145.667007	103.546913	0.035747	153.292938
103.559998	148.667007	103.566910	0.057108	153.264496
103.580002	142.000000	103.586914	0.092426	153.235901
103.599998	149.833008	103.606911	0.151258	153.207184
103.619995	145.833008	103.626907	0.250146	153.178253
103.639999	150.667007	103.646912	0.417797	153.149200
103.659996	142.333008	103.666908	0.703564	153.120056
103.680000	144.833008	103.686913	1.192380	153.090729

103.699997	164.333008	103.706909	2.025895	153.061279
103.719994	171.167007	103.726906	3.435109	153.031708
103.739998	184.167007	103.746910	5.780913	153.001984
103.759995	175.000000	103.766907	9.593341	152.972137
103.779999	208.500000	103.786911	15.613066	152.942230
103.800003	228.667007	103.806915	24.783497	152.912109
103.820000	250.833008	103.826920	38.174679	152.881927
103.840004	273.332977	103.846924	56.721230	152.851654
103.860001	316.166992	103.866920	80.640862	152.821259
103.880005	337.832977	103.886925	108.589912	152.790802
103.900002	355.000000	103.906921	136.825043	152.760162
103.919998	375.166992	103.926918	159.515610	152.729523
103.940002	376.166992	103.946922	170.632690	152.698730
103.959999	359.666992	103.966919	166.891922	152.667877
103.980003	353.666992	103.986923	149.661743	152.636932
104.000000	325.832977	104.006920	124.178795	152.605927
104.019997	320.666992	104.026917	96.664894	152.574829
104.040001	287.666992	104.046921	71.701271	152.543640
104.059998	258.832977	104.066917	51.378647	152.512421
104.080002	234.833008	104.086922	35.859882	152.481140
104.099998	211.500000	104.106918	24.466133	152.449799
104.119995	193.167007	104.126915	16.320005	152.418396
104.139999	187.500000	104.146919	10.639181	152.386902
104.159996	183.500000	104.166916	6.786363	152.355408
104.180000	178.500000	104.186920	4.244036	152.323853
104.199997	171.667007	104.206917	2.612562	152.292297
104.219994	173.000000	104.226921	1.589512	152.260651
104.239998	165.333008	104.246925	0.960487	152.229004
104.259995	162.500000	104.266922	0.579188	152.197327
104.279999	162.000000	104.286926	0.349914	152.165619
104.300003	156.000000	104.306931	0.212793	152.133881
104.320000	148.667007	104.326927	0.131067	152.102173
104.340004	145.667007	104.346931	0.082600	152.070435
104.360001	138.667007	104.366928	0.054414	152.038666
104.380005	151.833008	104.386932	0.038996	152.006927
104.400002	153.833008	104.406929	0.032304	151.975189
104.419998	143.333008	104.426926	0.032563	151.943420
104.440002	142.667007	104.446930	0.039782	151.911682

104.459999	149.667007	104.466927	0.055724	151.879974
104.480003	143.667007	104.486931	0.084428	151.848267
104.500000	144.833008	104.506927	0.133316	151.816620
104.519997	135.500000	104.526924	0.215482	151.784943
104.540001	143.500000	104.546928	0.353772	151.753326
104.559998	147.333008	104.566925	0.587836	151.721741
104.580002	145.833008	104.586929	0.987555	151.690186
104.599998	152.333008	104.606934	1.675030	151.658691
104.619995	148.667007	104.626930	2.861734	151.627258
104.639999	162.833008	104.646935	4.907945	151.595856
104.659996	166.167007	104.666931	8.395079	151.564514
104.680000	178.333008	104.686935	14.207442	151.533203
104.699997	193.833008	104.706932	23.529415	151.502014
104.719994	222.667007	104.726929	37.682022	151.470856
104.739998	245.167007	104.746933	57.614204	151.439789
104.759995	262.500000	104.766930	82.989624	151.408783
104.779999	285.000000	104.786934	111.293549	151.377869
104.800003	320.332977	104.806938	137.504684	151.347076
104.820000	326.832977	104.826935	155.353973	151.316284
104.840004	339.832977	104.846939	159.973740	151.285645
104.860001	342.332977	104.866936	150.390732	151.255127
104.880005	311.166992	104.886940	130.020844	151.224701
104.900002	294.666992	104.906937	104.692177	151.194366
104.919998	258.832977	104.926933	79.742737	151.164154
104.940002	235.667007	104.946938	58.327255	151.134064
104.959999	210.333008	104.966934	41.436573	151.104065
104.980003	199.000000	104.986938	28.741774	151.074219
105.000000	187.833008	105.006943	19.490654	151.044495
105.019997	168.833008	105.026939	12.917260	151.014923
105.040001	164.000000	105.046944	8.363976	150.985474
105.059998	162.167007	105.066940	5.300884	150.956146
105.080002	159.500000	105.086945	3.297018	150.927032
105.099998	148.000000	105.106941	2.021299	150.897980
105.119995	160.333008	105.126938	1.226762	150.869141
105.139999	158.000000	105.146942	0.740310	150.840454
105.159996	162.833008	105.166939	0.446322	150.811981
105.180000	157.667007	105.186943	0.269808	150.783569
105.199997	142.500000	105.206940	0.164266	150.755402

105.219994	142.500000	105.226936	0.101216	150.727386
105.239998	140.833008	105.246941	0.063679	150.699585
105.259995	139.833008	105.266937	0.041736	150.671967
105.279999	135.333008	105.286942	0.029731	150.644501
105.300003	135.333008	105.306946	0.024771	150.617218
105.320000	136.000000	105.326942	0.025838	150.590240
105.340004	141.000000	105.346947	0.033392	150.563385
105.360001	137.000000	105.366943	0.049125	150.536743
105.380005	139.167007	105.386948	0.075606	150.510315
105.400002	137.500000	105.406952	0.115338	150.484100
105.419998	133.500000	105.426949	0.169027	150.458160
105.440002	136.000000	105.446953	0.233385	150.432404
105.459999	137.000000	105.466949	0.299396	150.406860
105.480003	143.000000	105.486961	0.353593	150.381561
105.500000	139.667007	105.506958	0.382559	150.356537
105.519997	142.000000	105.526955	0.379757	150.331696
105.540001	136.833008	105.546959	0.349256	150.307159
105.559998	135.333008	105.566956	0.304110	150.282867
105.580002	138.167007	105.586960	0.260520	150.258789
105.599998	134.000000	105.606956	0.233083	150.235016
105.619995	129.333008	105.626953	0.234142	150.211517
105.639999	138.333008	105.646957	0.277638	150.188232
105.659996	145.167007	105.666954	0.385411	150.165283
105.680000	144.833008	105.686958	0.595789	150.142548
105.699997	137.833008	105.706955	0.974247	150.120148
105.719994	140.333008	105.726952	1.627646	150.097992
105.739998	149.833008	105.746956	2.717619	150.076172
105.759995	141.667007	105.766953	4.460599	150.054596
105.779999	155.667007	105.786964	7.103169	150.033356
105.800003	159.000000	105.806969	10.829828	150.012390
105.820000	168.000000	105.826965	15.627123	149.991760
105.840004	173.500000	105.846970	21.137060	149.971405
105.860001	189.333008	105.866966	26.600790	149.951385
105.880005	194.833008	105.886971	31.048077	149.931732
105.900002	207.333008	105.906967	33.647491	149.912323
105.919998	197.500000	105.926964	34.054188	149.893311
105.940002	203.333008	105.946968	32.461483	149.874573
105.959999	209.500000	105.966965	29.383526	149.856201

105.980003	197.833008	105.986969	25.383070	149.838135
106.000000	191.333008	106.006966	20.966976	149.820465
106.019997	181.000000	106.026962	16.580986	149.803131
106.040001	171.167007	106.046967	12.602334	149.786133
106.059998	164.667007	106.066963	9.287581	149.769501
106.080002	163.500000	106.086967	6.730263	149.753235
106.099998	155.667007	106.106964	4.901974	149.737335
106.119995	152.833008	106.126961	3.707594	149.721771
106.139999	156.833008	106.146965	3.035446	149.706573
106.159996	143.167007	106.166962	2.769207	149.691803
106.180000	145.000000	106.186974	2.777833	149.677368
106.199997	149.000000	106.206970	2.911351	149.663330
106.219994	148.000000	106.226967	3.018185	149.649689
106.239998	148.500000	106.246971	2.984851	149.636444
106.259995	143.333008	106.266968	2.774065	149.623566
106.279999	141.667007	106.286972	2.430974	149.611115
106.300003	144.333008	106.306976	2.052034	149.599030
106.320000	157.000000	106.326973	1.741073	149.587372
106.340004	144.500000	106.346977	1.585619	149.576141
106.360001	150.000000	106.366974	1.666020	149.565308
106.380005	144.500000	106.386978	2.083782	149.554901
106.400002	145.667007	106.406975	2.992311	149.544891
106.419998	145.167007	106.426971	4.615942	149.535339
106.440002	172.333008	106.446976	7.237362	149.526215
106.459999	171.667007	106.466972	11.121037	149.517487
106.480003	185.667007	106.486977	16.367258	149.509216
106.500000	195.667007	106.506973	22.685926	149.501373
106.519997	205.333008	106.526970	29.246346	149.493988
106.540001	205.500000	106.546974	34.741554	149.487030
106.559998	209.500000	106.566978	37.799484	149.480530
106.580002	212.500000	106.586983	37.610256	149.474487
106.599998	206.667007	106.606979	34.345596	149.468903
106.619995	209.667007	106.626976	29.042820	149.463776
106.639999	197.833008	106.646980	23.043705	149.459076
106.659996	189.500000	106.666977	17.423962	149.454895
106.680000	182.167007	106.686981	12.726440	149.451172
106.699997	165.167007	106.706978	9.063434	149.447906
106.719994	162.000000	106.726974	6.316488	149.445099

106.739998	164.167007	106.746979	4.307727	149.442810
106.759995	149.333008	106.766975	2.873389	149.440979
106.779999	156.500000	106.786980	1.874615	149.439636
106.800003	154.667007	106.806984	1.199307	149.438782
106.820000	159.667007	106.826981	0.756640	149.438446
106.840004	152.333008	106.846985	0.475601	149.438538
106.860001	147.333008	106.866982	0.304561	149.439178
106.880005	145.333008	106.886986	0.208110	149.440308
106.900002	141.000000	106.906982	0.165055	149.441925
106.919998	147.167007	106.926979	0.165925	149.444061
106.940002	145.167007	106.946983	0.211949	149.446686
106.959999	143.500000	106.966988	0.314602	149.449860
106.980003	139.333008	106.986992	0.495026	149.453522
107.000000	143.167007	107.006989	0.780828	149.457703
107.019997	145.000000	107.026985	1.197569	149.462433
107.040001	144.667007	107.046989	1.752049	149.467621
107.059998	134.000000	107.066986	2.409639	149.473389
107.080002	135.833008	107.086990	3.080974	149.479675
107.099998	143.500000	107.106987	3.630470	149.486481
107.119995	140.500000	107.126984	3.923087	149.493805
107.139999	137.833008	107.146988	3.885328	149.501678
107.159996	135.500000	107.166985	3.544230	149.510101
107.180000	138.333008	107.186989	3.011242	149.519043
107.199997	140.833008	107.206985	2.424562	149.528534
107.219994	138.667007	107.226982	1.889947	149.538544
107.239998	139.333008	107.246986	1.457385	149.549133
107.259995	131.167007	107.266983	1.131046	149.560272
107.279999	141.833008	107.286987	0.891066	149.571930
107.300003	141.000000	107.306992	0.714138	149.584198
107.320000	132.167007	107.326988	0.584338	149.596985
107.340004	128.833008	107.347000	0.497171	149.610321
107.360001	139.167007	107.366997	0.459326	149.624237
107.380005	136.833008	107.387001	0.485310	149.638672
107.400002	138.500000	107.406998	0.594383	149.653687
107.419998	135.667007	107.426994	0.806295	149.669281
107.440002	132.833008	107.446999	1.133471	149.685425
107.459999	137.333008	107.466995	1.567045	149.702148
107.480003	138.833008	107.487000	2.062852	149.719452

107.500000	137.500000	107.506996	2.535175	149.737305
107.519997	142.167007	107.526993	2.875827	149.755768
107.540001	144.500000	107.546997	2.994424	149.774750
107.559998	137.167007	107.566994	2.861607	149.794342
107.580002	135.000000	107.586998	2.523520	149.814484
107.599998	131.000000	107.606995	2.075627	149.835236
107.619995	145.500000	107.626991	1.615044	149.856537
107.639999	127.500000	107.646996	1.206372	149.878448
107.659996	147.167007	107.666992	0.875543	149.900940
107.680000	137.833008	107.686996	0.621388	149.924011
107.699997	140.667007	107.706993	0.432192	149.947662
107.719994	139.000000	107.726997	0.294379	149.971893
107.739998	141.833008	107.747002	0.196382	149.996735
107.759995	138.167007	107.766998	0.128668	150.022125
107.779999	145.500000	107.787003	0.083400	150.048187
107.800003	134.500000	107.807007	0.054406	150.074768
107.820000	136.833008	107.827003	0.037020	150.101929
107.840004	134.167007	107.847008	0.028023	150.129700
107.860001	138.333008	107.867004	0.025600	150.158081
107.880005	132.333008	107.887009	0.029263	150.187073
107.900002	137.833008	107.907005	0.039969	150.216583
107.919998	131.833008	107.927002	0.060544	150.246735
107.940002	138.333008	107.947006	0.096606	150.277496
107.959999	141.667007	107.967003	0.158247	150.308838
107.980003	136.500000	107.987007	0.263190	150.340759
108.000000	136.500000	108.007004	0.441491	150.373322
108.019997	129.333008	108.027000	0.743023	150.406433
108.040001	146.500000	108.047005	1.246598	150.440155
108.059998	142.333008	108.067001	2.066811	150.474487
108.080002	154.167007	108.087006	3.353928	150.509430
108.099998	162.000000	108.107002	5.265954	150.544891
108.119995	164.333008	108.127007	7.909141	150.581024
108.139999	178.167007	108.147011	11.230236	150.617706
108.159996	177.333008	108.167007	14.911655	150.655060
108.180000	171.667007	108.187012	18.350048	150.692932
108.199997	183.000000	108.207008	20.783855	150.731445
108.219994	181.167007	108.227005	21.596643	150.770538
108.239998	182.667007	108.247009	20.607933	150.810242

108.259995	180.667007	108.267006	18.166376	150.850525
108.279999	175.167007	108.287010	14.953100	150.891418
108.300003	170.000000	108.307014	11.657990	150.932892
108.320000	165.500000	108.327011	8.739069	150.974976
108.340004	165.833008	108.347015	6.375183	151.017639
108.360001	157.167007	108.367012	4.563151	151.060883
108.380005	151.167007	108.387016	3.220570	151.104736
108.400002	152.500000	108.407013	2.259326	151.149170
108.419998	146.500000	108.427010	1.606489	151.194214
108.440002	150.833008	108.447014	1.211296	151.239838
108.459999	142.667007	108.467010	1.047548	151.286072
108.480003	151.833008	108.487015	1.119703	151.332855
108.500000	151.500000	108.507019	1.476099	151.380249
108.519997	151.000000	108.527016	2.225768	151.428192
108.540001	149.833008	108.547020	3.559059	151.476746
108.559998	151.833008	108.567017	5.755643	151.525879
108.580002	162.167007	108.587021	9.168521	151.575623
108.599998	166.833008	108.607018	14.130779	151.625885
108.619995	174.667007	108.627014	20.784601	151.676788
108.639999	187.000000	108.647018	28.829483	151.728241
108.659996	192.667007	108.667015	37.307167	151.780273
108.680000	195.667007	108.687019	44.670296	151.832886
108.699997	193.167007	108.707016	49.202915	151.886047
108.719994	206.500000	108.727013	49.759182	151.939758
108.739998	194.333008	108.747017	46.316551	151.994049
108.759995	183.833008	108.767014	39.981655	152.048950
108.779999	180.333008	108.787018	32.394726	152.104401
108.800003	172.167007	108.807022	25.014961	152.160400
108.820000	177.500000	108.827019	18.702442	152.216949
108.840004	178.833008	108.847023	13.729783	152.274078
108.860001	159.000000	108.867020	10.043422	152.331757
108.880005	163.000000	108.887032	7.483603	152.389984
108.900002	158.333008	108.907028	5.929624	152.448761
108.919998	167.500000	108.927025	5.330926	152.508087
108.940002	160.500000	108.947029	5.721282	152.567963
108.959999	172.333008	108.967026	7.201588	152.628387
108.980003	175.000000	108.987030	9.901367	152.689331
109.000000	193.333008	109.007027	13.890109	152.750793

109.019997	203.000000	109.027023	19.053099	152.812836
109.040001	210.833008	109.047028	24.947388	152.875397
109.059998	210.333008	109.067024	30.722622	152.938446
109.080002	204.000000	109.087029	35.249329	153.002045
109.099998	215.333008	109.107025	37.448257	153.066223
109.119995	209.667007	109.127022	36.757439	153.130829
109.139999	223.333008	109.147026	33.405739	153.195984
109.159996	202.000000	109.167023	28.310459	153.261719
109.180000	204.000000	109.187027	22.624834	153.327881
109.199997	189.833008	109.207024	17.286871	153.394531
109.219994	182.667007	109.227020	12.788694	153.461700
109.239998	167.833008	109.247025	9.240394	153.529449
109.259995	161.333008	109.267029	6.546758	153.597626
109.279999	154.000000	109.287033	4.548827	153.666321
109.300003	156.333008	109.307045	3.095935	153.735504
109.320000	153.333008	109.327042	2.064889	153.805206
109.340004	149.500000	109.347046	1.352540	153.875336
109.360001	153.667007	109.367043	0.876486	153.945923
109.380005	157.000000	109.387047	0.570536	154.016998
109.400002	147.500000	109.407043	0.385793	154.088593
109.419998	147.500000	109.427040	0.288938	154.160553
109.440002	145.000000	109.447044	0.261864	154.233002
109.459999	151.167007	109.467041	0.301318	154.306000
109.480003	162.833008	109.487045	0.420214	154.379395
109.500000	162.000000	109.507042	0.650739	154.453186
109.519997	163.500000	109.527039	1.049044	154.527466
109.540001	158.333008	109.547043	1.698083	154.602203
109.559998	159.500000	109.567039	2.700979	154.677277
109.580002	164.333008	109.587044	4.159499	154.752838
109.599998	186.000000	109.607040	6.120871	154.828888
109.619995	170.333008	109.627037	8.509768	154.905212
109.639999	172.167007	109.647041	11.065514	154.982086
109.659996	167.667007	109.667046	13.343760	155.059296
109.680000	176.667007	109.687050	14.835490	155.136963
109.699997	173.833008	109.707047	15.169889	155.214996
109.719994	173.833008	109.727043	14.292400	155.293427
109.739998	163.000000	109.747047	12.486794	155.372223
109.759995	169.500000	109.767044	10.228681	155.451477

109.779999	164.667007	109.787048	7.965298	155.531036
109.800003	165.833008	109.807053	5.980642	155.611023
109.820000	165.500000	109.827049	4.378040	155.691315
109.840004	166.833008	109.847054	3.142725	155.772003
109.860001	152.833008	109.867050	2.216802	155.853058
109.880005	165.667007	109.887054	1.536933	155.934418
109.900002	156.667007	109.907051	1.050917	156.016083
109.919998	149.667007	109.927048	0.716282	156.098175
109.940002	153.333008	109.947052	0.499714	156.180573
109.959999	145.667007	109.967049	0.376839	156.263275
109.980003	159.667007	109.987053	0.332954	156.346375
110.000000	151.833008	110.007050	0.365725	156.429718
110.019997	150.167007	110.027046	0.488744	156.513336
110.040001	155.333008	110.047058	0.738197	156.597412
110.059998	163.500000	110.067055	1.182172	156.681641
110.080002	170.500000	110.087059	1.936485	156.766205
110.099998	173.167007	110.107056	3.179042	156.851044
110.119995	162.833008	110.127052	5.161228	156.936127
110.139999	167.667007	110.147057	8.194068	157.021515
110.159996	193.167007	110.167053	12.580589	157.107208
110.180000	205.167007	110.187057	18.496239	157.193115
110.199997	208.167007	110.207054	25.800318	157.279236
110.219994	232.333008	110.227051	33.917740	157.365631
110.239998	256.000000	110.247055	41.880882	157.452301
110.259995	262.666992	110.267052	48.629776	157.539154
110.279999	257.500000	110.287056	53.493103	157.626251
110.300003	274.666992	110.307060	56.469715	157.713593
110.320000	275.832977	110.327057	58.117439	157.801117
110.340004	275.166992	110.347061	59.098621	157.888855
110.360001	276.500000	110.367058	59.819084	157.976715
110.380005	275.166992	110.387062	60.520519	158.064850
110.400002	291.000000	110.407059	61.703392	158.153168
110.419998	296.832977	110.427063	64.403801	158.241638
110.440002	305.332977	110.447067	69.903473	158.330292
110.459999	304.832977	110.467064	78.922012	158.419006
110.480003	317.832977	110.487068	90.779068	158.507965
110.500000	318.166992	110.507065	103.056427	158.597076
110.519997	320.666992	110.527061	112.226700	158.686218

110.540001	328.832977	110.547066	115.035477	158.775604
110.559998	337.332977	110.567062	110.014580	158.865082
110.580002	304.166992	110.587067	98.164825	158.954681
110.599998	311.332977	110.607063	82.362267	159.044342
110.619995	291.500000	110.627060	65.908722	159.134125
110.639999	276.166992	110.647064	51.288364	159.224030
110.659996	267.332977	110.667061	39.730515	159.313934
110.680000	241.167007	110.687065	31.414078	159.403992
110.699997	218.500000	110.707062	25.957266	159.494110
110.719994	217.667007	110.727058	22.707973	159.584167
110.739998	215.833008	110.747063	20.893444	159.674408
110.759995	200.000000	110.767059	19.707573	159.764679
110.779999	200.000000	110.787064	18.450294	159.855011
110.800003	187.000000	110.807076	16.702377	159.945282
110.820000	193.833008	110.827072	14.413726	160.035645
110.840004	189.833008	110.847076	11.820971	160.125977
110.860001	173.000000	110.867073	9.266837	160.216278
110.880005	171.833008	110.887077	7.015937	160.306610
110.900002	175.833008	110.907074	5.189620	160.396942
110.919998	172.333008	110.927071	3.786260	160.487213
110.940002	160.333008	110.947075	2.748317	160.577484
110.959999	161.333008	110.967072	2.013147	160.667694
110.980003	159.833008	110.987076	1.533999	160.757874
111.000000	158.000000	111.007072	1.289242	160.847900
111.019997	157.167007	111.027069	1.283913	160.937958
111.040001	154.500000	111.047073	1.555700	161.027924
111.059998	161.667007	111.067070	2.179498	161.117767
111.080002	157.667007	111.087074	3.266897	161.207581
111.099998	162.500000	111.107071	4.944032	161.297272
111.119995	168.500000	111.127068	7.303372	161.386749
111.139999	169.333008	111.147072	10.319907	161.476227
111.159996	166.333008	111.167068	13.754272	161.565582
111.180000	167.667007	111.187080	17.120800	161.654755
111.199997	169.667007	111.207077	19.753077	161.743805
111.219994	170.167007	111.227074	21.040354	161.832611
111.239998	173.667007	111.247078	20.682045	161.921356
111.259995	158.833008	111.267075	18.836622	162.009918
111.279999	163.167007	111.287079	16.035423	162.098267

111.300003	162.833008	111.307083	12.930399	162.186432
111.320000	154.167007	111.327080	10.041697	162.274353
111.340004	160.500000	111.347084	7.643631	162.362122
111.360001	161.167007	111.367081	5.805848	162.449677
111.380005	152.833008	111.387085	4.477950	162.536987
111.400002	156.000000	111.407082	3.568376	162.623993
111.419998	152.333008	111.427078	2.972792	162.710815
111.440002	168.000000	111.447083	2.584899	162.797424
111.459999	155.833008	111.467079	2.304060	162.883636
111.480003	139.833008	111.487083	2.049553	162.969696
111.500000	157.667007	111.507080	1.776965	163.055450
111.519997	155.000000	111.527077	1.480848	163.140839
111.540001	154.167007	111.547089	1.182177	163.226074
111.559998	154.000000	111.567085	0.908793	163.310822
111.580002	151.333008	111.587090	0.678540	163.395355
111.599998	146.333008	111.607086	0.496316	163.479553
111.619995	140.667007	111.627083	0.357345	163.563324
111.639999	150.167007	111.647087	0.253422	163.646820
111.659996	146.333008	111.667084	0.176764	163.729950
111.680000	142.333008	111.687088	0.120988	163.812775
111.699997	142.500000	111.707085	0.081227	163.895111
111.719994	138.667007	111.727081	0.053548	163.977142
111.739998	145.833008	111.747086	0.034778	164.058746
111.759995	143.000000	111.767082	0.022397	164.139923
111.779999	143.667007	111.787086	0.014450	164.220734
111.800003	147.000000	111.807091	0.009523	164.301117
111.820000	143.667007	111.827087	0.006638	164.381042
111.840004	146.333008	111.847092	0.005168	164.460541
111.860001	141.667007	111.867088	0.004766	164.539490
111.880005	153.167007	111.887093	0.005303	164.618103
111.900002	147.833008	111.907089	0.006857	164.696228
111.919998	145.833008	111.927094	0.009728	164.773834
111.940002	141.667007	111.947098	0.014507	164.850983
111.959999	137.833008	111.967094	0.022214	164.927612
111.980003	143.167007	111.987099	0.034565	165.003754
112.000000	144.167007	112.007095	0.054388	165.079376
112.019997	147.500000	112.027092	0.086405	165.154449
112.040001	151.333008	112.047096	0.138541	165.229004

112.059998	142.333008	112.067093	0.224021	165.303009
112.080002	140.833008	112.087097	0.365116	165.376495
112.099998	147.000000	112.107094	0.598366	165.449310
112.119995	156.333008	112.127090	0.982706	165.521637
112.139999	156.000000	112.147095	1.608999	165.593384
112.159996	164.333008	112.167091	2.606933	165.664520
112.180000	162.667007	112.187096	4.146136	165.735077
112.199997	162.500000	112.207092	6.411137	165.805054
112.219994	174.500000	112.227089	9.550421	165.874359
112.239998	181.167007	112.247093	13.588974	165.943054
112.259995	190.500000	112.267090	18.332916	166.011139
112.279999	191.500000	112.287094	23.356394	166.078552
112.300003	199.667007	112.307106	28.103292	166.145325
112.320000	200.333008	112.327103	32.135624	166.211456
112.340004	212.667007	112.347107	35.407887	166.276917
112.360001	214.667007	112.367104	38.307667	166.341644
112.380005	232.000000	112.387108	41.403847	166.405731
112.400002	233.667007	112.407104	44.963692	166.469147
112.419998	229.500000	112.427101	48.591999	166.531738
112.440002	237.500000	112.447105	51.255402	166.593719
112.459999	240.667007	112.467102	51.750675	166.654999
112.480003	237.167007	112.487114	49.354946	166.715515
112.500000	244.000000	112.507111	44.236755	166.775269
112.519997	225.333008	112.527107	37.353424	166.834229
112.540001	207.167007	112.547112	29.964485	166.892517
112.559998	180.167007	112.567108	23.117319	166.950043
112.580002	187.667007	112.587112	17.368107	167.006775
112.599998	186.000000	112.607109	12.846175	167.062714
112.619995	170.833008	112.627106	9.427506	167.117859
112.639999	177.333008	112.647110	6.912805	167.172272
112.659996	167.667007	112.667107	5.112652	167.225800
112.680000	167.667007	112.687119	3.858921	167.278595
112.699997	162.167007	112.707115	3.005990	167.330475
112.719994	169.000000	112.727112	2.421489	167.381622
112.739998	168.000000	112.747116	1.998049	167.431885
112.759995	157.500000	112.767113	1.663847	167.481293
112.779999	163.833008	112.787117	1.385905	167.529846
112.800003	163.167007	112.807121	1.164352	167.577606

112.820000	164.500000	112.827118	1.019044	167.624481
112.840004	158.500000	112.847122	0.979882	167.670441
112.860001	156.833008	112.867119	1.087023	167.715515
112.880005	159.333008	112.887123	1.399888	167.759735
112.900002	155.500000	112.907120	2.009830	167.803009
112.919998	163.667007	112.927116	3.050701	167.845459
112.940002	159.333008	112.947121	4.696485	167.886932
112.959999	169.500000	112.967117	7.129862	167.927582
112.980003	152.333008	112.987122	10.478948	167.967255
113.000000	167.667007	113.007118	14.701134	168.005951
113.019997	163.500000	113.027115	19.475281	168.043762
113.040001	168.333008	113.047119	24.150146	168.080658
113.059998	170.500000	113.067123	27.844358	168.116608
113.080002	170.333008	113.087128	29.731838	168.151550
113.099998	164.333008	113.107124	29.383188	168.185516
113.119995	167.333008	113.127121	26.964773	168.218597
113.139999	171.333008	113.147125	23.148407	168.250702
113.159996	166.167007	113.167122	18.801638	168.281738
113.180000	162.167007	113.187126	14.640854	168.311859
113.199997	161.000000	113.207123	11.073575	168.340973
113.219994	154.333008	113.227119	8.211905	168.369080
113.239998	156.833008	113.247124	6.000159	168.396210
113.259995	150.833008	113.267120	4.329779	168.422333
113.279999	158.500000	113.287125	3.094757	168.447418
113.300003	140.333008	113.307129	2.214095	168.471527
113.320000	156.167007	113.327126	1.628500	168.494598
113.340004	152.667007	113.347130	1.298465	168.516632
113.360001	162.333008	113.367126	1.208264	168.537628
113.380005	154.500000	113.387131	1.371694	168.557648
113.400002	159.667007	113.407127	1.841059	168.576569
113.419998	169.333008	113.427132	2.715755	168.594421
113.440002	175.500000	113.447136	4.144359	168.611298
113.459999	185.667007	113.467133	6.316305	168.627075
113.480003	178.000000	113.487137	9.440981	168.641815
113.500000	185.333008	113.507133	13.700219	168.655487
113.519997	206.000000	113.527130	19.208216	168.668121
113.540001	202.167007	113.547134	25.969177	168.679626
113.559998	217.667007	113.567131	33.833393	168.690094

113.580002	226.000000	113.587135	42.463272	168.699493
113.599998	234.167007	113.607132	51.216385	168.707825
113.619995	242.167007	113.627129	59.083431	168.715027
113.639999	241.667007	113.647133	64.746742	168.721222
113.659996	238.667007	113.667130	66.926033	168.726288
113.680000	237.500000	113.687134	64.934807	168.730255
113.699997	230.833008	113.707130	59.078667	168.733124
113.719994	213.667007	113.727127	50.592064	168.734955
113.739998	219.833008	113.747131	41.115467	168.735626
113.759995	202.167007	113.767128	32.080437	168.735260
113.779999	202.500000	113.787132	24.318449	168.733734
113.800003	198.167007	113.807144	18.088770	168.731110
113.820000	196.500000	113.827141	13.297497	168.727417
113.840004	185.000000	113.847145	9.712964	168.722626
113.860001	172.000000	113.867142	7.125067	168.716705
113.880005	178.500000	113.887146	5.379097	168.709656
113.900002	180.000000	113.907143	4.388870	168.701538
113.919998	181.167007	113.927139	4.127666	168.692291
113.940002	166.667007	113.947144	4.627908	168.681946
113.959999	177.500000	113.967140	5.965846	168.670502
113.980003	175.500000	113.987144	8.224039	168.657928
114.000000	193.000000	114.007141	11.410288	168.644257
114.019997	185.333008	114.027138	15.358442	168.629486
114.040001	191.333008	114.047142	19.639164	168.613586
114.059998	192.500000	114.067139	23.556368	168.596619
114.080002	200.333008	114.087143	26.310244	168.578461
114.099998	188.667007	114.107140	27.264841	168.559296
114.119995	186.833008	114.127136	26.224836	168.538940
114.139999	182.500000	114.147141	23.516092	168.517517
114.159996	183.500000	114.167137	19.830530	168.495026
114.180000	180.833008	114.187149	15.912868	168.471344
114.199997	169.833008	114.207146	12.321645	168.446625
114.219994	174.500000	114.227142	9.321960	168.420807
114.239998	170.000000	114.247147	6.957066	168.393860
114.259995	168.000000	114.267143	5.161493	168.365875
114.279999	169.333008	114.287148	3.843687	168.336761
114.300003	170.167007	114.307152	2.934060	168.306549
114.320000	169.833008	114.327148	2.394880	168.275238

114.340004	171.833008	114.347153	2.225290	168.242889
114.360001	164.833008	114.367149	2.473299	168.209503
114.380005	186.833008	114.387154	3.257258	168.174957
114.400002	184.667007	114.407150	4.797816	168.139313
114.419998	188.167007	114.427147	7.458918	168.102722
114.440002	201.833008	114.447151	11.781352	168.064972
114.459999	209.167007	114.467148	18.470858	168.026215
114.480003	226.833008	114.487152	28.318859	167.986359
114.500000	256.332977	114.507149	41.941154	167.945465
114.519997	279.332977	114.527145	59.395805	167.903534
114.540001	299.166992	114.547157	79.706970	167.860565
114.559998	333.500000	114.567154	100.507530	167.816559
114.580002	331.000000	114.587158	118.376610	167.771515
114.599998	342.166992	114.607155	129.703705	167.725494
114.619995	348.832977	114.627151	132.135132	167.678406
114.639999	333.666992	114.647156	125.625908	167.630310
114.659996	340.166992	114.667152	112.519615	167.581268
114.680000	333.666992	114.687157	96.472206	167.531158
114.699997	303.832977	114.707153	80.982735	167.480072
114.719994	270.332977	114.727150	68.257927	167.428009
114.739998	265.000000	114.747154	58.945633	167.374969
114.759995	251.667007	114.767151	52.487991	167.320953
114.779999	226.000000	114.787155	47.696461	167.265961
114.800003	224.333008	114.807159	43.346493	167.210022
114.820000	222.500000	114.827156	38.595928	167.153168
114.840004	204.667007	114.847160	33.183609	167.095367
114.860001	199.833008	114.867157	27.380367	167.036621
114.880005	199.500000	114.887161	21.707516	166.976929
114.900002	192.667007	114.907158	16.651375	166.916412
114.919998	189.000000	114.927162	12.467443	166.854919
114.940002	193.167007	114.947166	9.183405	166.792511
114.959999	187.000000	114.967163	6.682954	166.729309
114.980003	187.667007	114.987167	4.804388	166.665131
115.000000	175.333008	115.007164	3.405275	166.600189
115.019997	179.333008	115.027161	2.373228	166.534332
115.040001	173.333008	115.047165	1.623629	166.467682
115.059998	165.000000	115.067162	1.090855	166.400177
115.080002	177.500000	115.087166	0.720697	166.331818

115.099998	166.167007	115.107162	0.469682	166.262726
115.119995	167.833008	115.127159	0.303035	166.192780
115.139999	154.167007	115.147163	0.194462	166.122040
115.159996	152.167007	115.167160	0.124975	166.050568
115.180000	147.833008	115.187164	0.081244	165.978271
115.199997	150.167007	115.207161	0.054431	165.905365
115.219994	158.667007	115.227158	0.038832	165.831573
115.239998	158.500000	115.247162	0.031036	165.757080
115.259995	155.333008	115.267159	0.029280	165.681946
115.279999	160.000000	115.287170	0.033090	165.606018
115.300003	147.000000	115.307182	0.043193	165.529449
115.320000	149.167007	115.327179	0.061675	165.452209
115.340004	151.167007	115.347183	0.092539	165.374298
115.360001	159.500000	115.367180	0.142639	165.295837
115.380005	153.167007	115.387184	0.223565	165.216675
115.400002	158.833008	115.407181	0.354476	165.136841
115.419998	153.333008	115.427177	0.567166	165.056580
115.440002	157.833008	115.447182	0.913670	164.975616
115.459999	163.167007	115.467178	1.476448	164.894073
115.480003	163.000000	115.487183	2.382830	164.811981
115.500000	178.833008	115.507179	3.815314	164.729401
115.519997	183.833008	115.527176	6.015953	164.646271
115.540001	176.000000	115.547180	9.263306	164.562622
115.559998	178.667007	115.567177	13.798676	164.478546
115.580002	187.667007	115.587181	19.710573	164.393951
115.599998	195.000000	115.607178	26.758524	164.308868
115.619995	189.833008	115.627174	34.261078	164.223480
115.639999	197.000000	115.647179	41.111794	164.137543
115.659996	211.167007	115.667183	46.030800	164.051239
115.680000	207.000000	115.687187	48.027695	163.964508
115.699997	204.000000	115.707184	46.838852	163.877441
115.719994	200.167007	115.727180	43.084553	163.790070
115.739998	204.667007	115.747185	38.031361	163.702271
115.759995	205.833008	115.767181	33.122856	163.614227
115.779999	199.833008	115.787186	29.522928	163.525848
115.800003	197.833008	115.807190	27.904583	163.437164
115.820000	201.167007	115.827187	28.417332	163.348358
115.840004	185.000000	115.847191	30.741865	163.259216

115.860001	203.667007	115.867188	34.140694	163.169830
115.880005	197.167007	115.887192	37.562180	163.080292
115.900002	195.500000	115.907188	39.857376	162.990601
115.919998	193.500000	115.927185	40.137287	162.900696
115.940002	197.000000	115.947189	38.090008	162.810669
115.959999	188.000000	115.967186	34.087673	162.720551
115.980003	192.167007	115.987190	28.976532	162.630310
116.000000	195.333008	116.007187	23.703236	162.539978
116.019997	188.833008	116.027191	18.963949	162.449554
116.040001	176.333008	116.047195	15.083512	162.359100
116.059998	176.167007	116.067192	12.069344	162.268768
116.080002	174.833008	116.087196	9.759307	162.178284
116.099998	176.667007	116.107193	7.966912	162.087860
116.119995	168.333008	116.127190	6.556703	161.997589
116.139999	176.500000	116.147194	5.476048	161.907288
116.159996	167.333008	116.167191	4.748080	161.817108
116.180000	160.833008	116.187195	4.444761	161.726990
116.199997	177.833008	116.207191	4.661951	161.637115
116.219994	158.167007	116.227188	5.493049	161.547272
116.239998	169.667007	116.247192	7.000031	161.457672
116.259995	165.833008	116.267189	9.165261	161.368317
116.279999	176.667007	116.287193	11.838894	161.279144
116.300003	165.333008	116.307198	14.692292	161.190155
116.320000	181.167007	116.327194	17.240047	161.101501
116.340004	171.500000	116.347198	18.954548	161.013153
116.360001	166.500000	116.367195	19.439402	160.925140
116.380005	160.500000	116.387207	18.593416	160.837341
116.400002	159.833008	116.407204	16.652054	160.750031
116.419998	167.500000	116.427200	14.074047	160.663086
116.440002	163.333008	116.447205	11.349437	160.576508
116.459999	163.500000	116.467201	8.843427	160.490448
116.480003	157.167007	116.487206	6.731955	160.404785
116.500000	154.333008	116.507202	5.044740	160.319550
116.519997	148.500000	116.527199	3.730944	160.235016
116.540001	168.667007	116.547203	2.720214	160.150818
116.559998	145.167007	116.567200	1.951182	160.067230
116.580002	152.667007	116.587204	1.374563	159.984222
116.599998	147.167007	116.607201	0.952259	159.901825

116.619995	147.667007	116.627197	0.651942	159.819977
116.639999	150.167007	116.647202	0.446429	159.738831
116.659996	156.667007	116.667198	0.313831	159.658417
116.680000	153.333008	116.687202	0.237751	159.578644
116.699997	158.000000	116.707199	0.208391	159.499542
116.719994	155.333008	116.727196	0.222972	159.421326
116.739998	152.000000	116.747200	0.286844	159.343781
116.759995	148.000000	116.767204	0.414720	159.266998
116.779999	162.833008	116.787209	0.632031	159.191010
116.800003	158.167007	116.807213	0.975080	159.115906
116.820000	164.667007	116.827209	1.487470	159.041626
116.840004	155.167007	116.847214	2.211119	158.968292
116.860001	165.167007	116.867210	3.166238	158.895905
116.880005	170.500000	116.887215	4.328455	158.824310
116.900002	175.833008	116.907211	5.603571	158.753754
116.919998	174.167007	116.927208	6.827319	158.684235
116.940002	182.500000	116.947212	7.794899	158.615631
116.959999	179.000000	116.967209	8.324022	158.548065
116.980003	186.333008	116.987213	8.327834	158.481598
117.000000	177.500000	117.007210	7.850756	158.416199
117.019997	178.833008	117.027206	7.048601	158.351898
117.040001	176.333008	117.047211	6.123897	158.288635
117.059998	191.500000	117.067207	5.261140	158.226593
117.080002	171.833008	117.087212	4.590315	158.165710
117.099998	178.500000	117.107208	4.190909	158.105988
117.119995	172.333008	117.127213	4.105389	158.047455
117.139999	171.833008	117.147217	4.346431	157.990173
117.159996	175.833008	117.167213	4.884880	157.934204
117.180000	174.500000	117.187218	5.632635	157.879364
117.199997	174.167007	117.207214	6.434538	157.825958
117.219994	171.667007	117.227211	7.092604	157.773834
117.239998	176.167007	117.247215	7.419087	157.723022
117.259995	172.333008	117.267212	7.302459	157.673553
117.279999	180.833008	117.287216	6.750361	157.625519
117.300003	171.833008	117.307220	5.880970	157.578796
117.320000	181.167007	117.327217	4.867887	157.533600
117.340004	163.833008	117.347221	3.870177	157.489746
117.360001	164.333008	117.367218	2.990922	157.447388

117.380005	163.833008	117.387222	2.268216	157.406494
117.400002	155.667007	117.407219	1.697562	157.367157
117.419998	164.833008	117.427216	1.254861	157.329254
117.440002	156.833008	117.447220	0.914285	157.292877
117.459999	160.167007	117.467216	0.654980	157.258087
117.480003	159.667007	117.487228	0.460412	157.224792
117.500000	158.167007	117.507225	0.317828	157.193146
117.519997	147.833008	117.527222	0.215993	157.163116
117.540001	151.333008	117.547226	0.145373	157.134613
117.559998	149.000000	117.567223	0.098099	157.107819
117.580002	148.000000	117.587227	0.067905	157.082581
117.599998	151.833008	117.607224	0.050308	157.059082
117.619995	149.333008	117.627220	0.042427	157.037231
117.639999	155.000000	117.647224	0.042969	157.016998
117.659996	164.000000	117.667221	0.052254	156.998535
117.680000	139.500000	117.687225	0.072504	156.981781
117.699997	149.333008	117.707222	0.108444	156.966705
117.719994	155.333008	117.727219	0.168461	156.953339
117.739998	148.667007	117.747223	0.266303	156.941742
117.759995	153.333008	117.767220	0.423086	156.931915
117.779999	162.667007	117.787224	0.669745	156.923798
117.800003	155.667007	117.807228	1.047483	156.917511
117.820000	173.833008	117.827225	1.604824	156.912964
117.840004	165.167007	117.847237	2.389082	156.910187
117.860001	161.000000	117.867241	3.424788	156.909210
117.880005	166.833008	117.887245	4.689430	156.910034
117.900002	174.167007	117.907242	6.086171	156.912628
117.919998	181.333008	117.927238	7.438596	156.917084
117.940002	179.333008	117.947243	8.517780	156.923309
117.959999	180.167007	117.967239	9.107269	156.931335
117.980003	174.333008	117.987244	9.085697	156.941193
118.000000	179.833008	118.007240	8.476454	156.952820
118.019997	167.667007	118.027237	7.437438	156.966309
118.040001	173.500000	118.047241	6.192666	156.981537
118.059998	169.333008	118.067238	4.950438	156.998627
118.080002	167.167007	118.087242	3.844881	157.017548
118.099998	160.500000	118.107239	2.930848	157.038239
118.119995	157.500000	118.127235	2.205024	157.060699

118.139999	155.333008	118.147240	1.639011	157.084991
118.159996	152.667007	118.167236	1.201559	157.111053
118.180000	157.167007	118.187241	0.866076	157.138885
118.199997	156.167007	118.207237	0.612668	157.168488
118.219994	160.333008	118.227242	0.425039	157.199860
118.239998	148.667007	118.247246	0.289583	157.232971
118.259995	150.833008	118.267242	0.194368	157.267822
118.279999	152.000000	118.287247	0.129137	157.304413
118.300003	158.833008	118.307251	0.085657	157.342651
118.320000	150.000000	118.327248	0.057586	157.382599
118.340004	149.167007	118.347252	0.040319	157.424255
118.360001	154.000000	118.367249	0.030799	157.467529
118.380005	144.167007	118.387253	0.027208	157.512421
118.400002	151.500000	118.407249	0.028824	157.558960
118.419998	154.333008	118.427246	0.035962	157.607056
118.440002	146.500000	118.447250	0.050118	157.656738
118.459999	154.333008	118.467247	0.074349	157.707977
118.480003	147.333008	118.487251	0.114073	157.760712
118.500000	152.833008	118.507248	0.178272	157.814911
118.519997	152.167007	118.527245	0.281549	157.870636
118.540001	156.833008	118.547249	0.446911	157.927704
118.559998	157.667007	118.567245	0.708837	157.986176
118.580002	157.500000	118.587257	1.117007	158.046112
118.599998	157.333008	118.607254	1.735231	158.107239
118.619995	156.667007	118.627251	2.637594	158.169678
118.639999	173.500000	118.647255	3.891461	158.233429
118.659996	167.000000	118.667252	5.525528	158.298309
118.680000	177.667007	118.687256	7.493292	158.364410
118.699997	177.333008	118.707253	9.632854	158.431610
118.719994	174.500000	118.727249	11.666953	158.499817
118.739998	187.167007	118.747253	13.249272	158.569092
118.759995	174.333008	118.767250	14.064849	158.639313
118.779999	184.500000	118.787254	13.950195	158.710480
118.800003	171.500000	118.807259	12.959043	158.782501
118.820000	186.000000	118.827255	11.340914	158.855316
118.840004	178.333008	118.847260	9.433829	158.928925
118.860001	178.833008	118.867256	7.545960	159.003143
118.880005	171.500000	118.887260	5.872444	159.078033

118.900002	162.667007	118.907257	4.490997	159.153473
118.919998	164.000000	118.927254	3.394215	159.229370
118.940002	154.500000	118.947266	2.538462	159.305817
118.959999	160.500000	118.967262	1.877861	159.382477
118.980003	159.000000	118.987267	1.373367	159.459534
119.000000	160.833008	119.007263	0.997142	159.536743
119.019997	156.833008	119.027260	0.727522	159.613983
119.040001	160.833008	119.047264	0.547001	159.691345
119.059998	156.333008	119.067261	0.440756	159.768707
119.080002	153.833008	119.087265	0.395262	159.845886
119.099998	160.833008	119.107262	0.397443	159.922821
119.119995	161.333008	119.127258	0.432999	159.999512
119.139999	153.667007	119.147263	0.485498	160.075806
119.159996	151.833008	119.167259	0.536817	160.151520
119.180000	157.500000	119.187263	0.569983	160.226715
119.199997	161.333008	119.207260	0.573144	160.301239
119.219994	159.500000	119.227257	0.543032	160.374847
119.239998	153.667007	119.247261	0.485302	160.447601
119.259995	147.000000	119.267258	0.411596	160.519379
119.279999	145.667007	119.287262	0.334477	160.589996
119.300003	156.000000	119.307274	0.263485	160.659332
119.320000	158.833008	119.327271	0.203549	160.727356
119.340004	155.833008	119.347275	0.155505	160.793854
119.360001	152.000000	119.367271	0.118146	160.858673
119.380005	154.167007	119.387276	0.089624	160.921783
119.400002	150.000000	119.407272	0.068417	160.983032
119.419998	146.833008	119.427269	0.053488	161.042236
119.440002	154.333008	119.447273	0.044303	161.099274
119.459999	153.667007	119.467270	0.040804	161.154022
119.480003	144.833008	119.487274	0.043484	161.206390
119.500000	144.333008	119.507271	0.053667	161.256073
119.519997	152.167007	119.527267	0.073974	161.302948
119.540001	160.667007	119.547272	0.109098	161.347076
119.559998	159.500000	119.567268	0.166920	161.388000
119.580002	156.167007	119.587273	0.260382	161.425720
119.599998	159.500000	119.607269	0.409702	161.460114
119.619995	152.000000	119.627266	0.645520	161.490845
119.639999	160.833008	119.647270	1.011621	161.517883

```
        119.659996      163.000000      119.667274        1.565272      161.540985
        119.680000      157.000000      119.687279        2.373018      161.559906
        119.699997      173.667007      119.707275        3.496573      161.574615
        119.719994      177.167007      119.727272        4.968658      161.584747
        119.739998      177.833008      119.747276        6.757923      161.590271
        119.759995      177.833008      119.767273        8.733524      161.590820
        119.779999      179.667007      119.787277       10.660264      161.586273
        119.800003      178.000000      119.807281       12.228301      161.576447
        119.820000      182.833008      119.827278       13.139578      161.561127
        119.840004      179.000000      119.847282       13.212828      161.540009
        119.860001      187.000000      119.867279       12.454929      161.512939
        119.880005      182.667007      119.887283       11.058356      161.479645
        119.900002      177.667007      119.907280        9.323314      161.440033
        119.919998      188.833008      119.927277        7.543815      161.393707
        119.940002      170.333008      119.947281        5.926422      161.340363
        119.959999      181.833008      119.967278        4.568253      161.279938
        119.980003      173.667007      119.987282        3.477653      161.212128
        120.000000      170.333008      120.007278        2.620664      161.136566

_reflns_number_total      288
_reflns_limit_h_min       0
_reflns_limit_h_max       10
_reflns_limit_k_min       -8
_reflns_limit_k_max       0
_reflns_limit_l_min       0
_reflns_limit_l_max       7
_reflns_d_resolution_high 8.157
_reflns_d_resolution_low  .890

loop_
      _refln_index_h
      _refln_index_k
      _refln_index_l
      _refln_F_squared_meas
      _refln_d_spacing
      _refln_intensity_meas
   1   0   0       42.269      8.15744    25310.180
   1   0   1       39.423      5.26160    19333.629
```

2	-1	0	65.508	4.70970	12784.610
2	0	0	206.213	4.07872	29850.154
2	-1	1	108.800	3.88729	28483.900
2	0	1	50.766	3.50922	10712.862
0	0	2	3537.756	3.44266	119476.109
1	0	2	203.690	3.17177	34656.508
3	-2	0	368.708	3.08322	29517.223
3	-1	0	368.585	3.08322	29507.340
3	-2	1	1399.155	2.81397	183836.531
3	-1	1	1398.832	2.81397	183794.156
2	-1	2	1352.113	2.77930	172920.281
3	0	0	3328.732	2.71915	202916.969
2	0	2	786.776	2.63080	89217.047
3	0	1	189.062	2.52907	19652.068
4	-2	0	55.174	2.35485	2446.155
3	-2	2	150.311	2.29677	12601.267
3	-1	2	150.293	2.29677	12599.729
4	-3	0	1194.871	2.26247	48416.344
4	-1	0	1194.206	2.26247	48389.363
4	-2	1	166.128	2.22814	13005.979
1	0	3	119.407	2.20933	9170.566
4	-3	1	226.494	2.14940	16341.876
4	-1	1	226.468	2.14940	16340.031
3	0	2	162.468	2.13383	11529.804
2	-1	3	462.380	2.06317	30382.352
4	0	0	414.611	2.03936	13263.789
2	0	3	388.126	2.00019	23749.287
4	0	1	789.192	1.95539	45830.445
4	-2	2	2544.304	1.94365	145709.406
4	-3	2	646.843	1.89072	34746.242
4	-1	2	646.678	1.89072	34737.363
5	-3	0	448.370	1.87145	11758.925
5	-2	0	448.297	1.87145	11757.012
3	-2	3	1745.527	1.84103	88127.469
3	-1	3	1745.184	1.84103	88110.156
5	-3	1	919.767	1.80593	44395.691
5	-2	1	919.538	1.80593	44384.641
5	-4	0	1523.108	1.78010	35541.234

5	-1	0	1522.938	1.78010	35537.242
4	0	2	1616.134	1.75461	72918.602
3	0	3	1617.276	1.75387	72897.906
5	-4	1	1060.421	1.72343	45875.762
5	-1	1	1060.665	1.72343	45886.258
0	0	4	12554.347	1.72133	90261.453
1	0	4	198.501	1.68424	8136.071
5	-3	2	466.190	1.64421	18058.498
5	-2	2	466.138	1.64421	18056.488
4	-2	3	937.457	1.64360	36282.051
5	0	0	270.186	1.63149	5138.425
2	-1	4	633.272	1.61673	23578.967
4	-3	3	327.426	1.61122	12093.941
4	-1	3	327.353	1.61122	12091.275
5	0	1	489.419	1.58753	17460.234
2	0	4	533.436	1.58588	18984.365
5	-4	2	159.180	1.58123	5626.137
5	-1	2	159.173	1.58123	5625.883
6	-3	0	401.407	1.56990	6975.360
6	-4	0	1048.180	1.54161	17456.602
6	-2	0	1047.797	1.54161	17450.227
6	-3	1	888.918	1.53062	29118.406
4	0	3	263.696	1.52448	8557.434
6	-4	1	807.124	1.50437	25396.430
6	-2	1	807.618	1.50437	25411.973
3	-2	4	810.991	1.50297	25463.010
3	-1	4	810.945	1.50297	25461.572
5	0	2	2207.358	1.47431	66287.531
6	-5	0	547.981	1.46512	8110.343
6	-1	0	547.799	1.46512	8107.647
3	0	4	3185.034	1.45440	92703.578
5	-2	3	1273.920	1.45039	36844.566
5	-3	3	1273.920	1.45039	36844.566
6	-5	1	1095.535	1.43304	30826.150
6	-1	1	1094.964	1.43304	30810.088
6	-3	2	1191.710	1.42839	33285.715
6	-4	2	462.694	1.40699	12489.335
6	-2	2	462.127	1.40699	12474.012

5	-4	3	455.955	1.40660	12299.903
5	-1	3	455.787	1.40660	12295.366
4	-2	4	171.497	1.38965	4502.164
4	-3	4	95.585	1.36992	2430.814
4	-1	4	95.585	1.36992	2430.813
6	0	0	386.630	1.35957	4834.849
1	0	5	180.639	1.35785	4505.268
6	-5	2	370.218	1.34811	9089.510
6	-1	2	370.073	1.34811	9085.945
7	-4	0	197.007	1.34108	2391.108
7	-3	0	196.949	1.34108	2390.401
6	0	1	120.588	1.33382	2893.113
5	0	3	112.660	1.32975	2685.226
2	-1	5	764.473	1.32172	17986.680
7	-4	1	802.370	1.31634	18715.305
7	-3	1	802.178	1.31634	18710.812
4	0	4	1584.157	1.31540	36894.711
7	-5	0	1132.174	1.30624	12991.223
7	-2	0	1131.897	1.30624	12988.043
2	0	5	1034.023	1.30471	23671.828
6	-3	3	302.617	1.29576	6829.166
7	-5	1	877.143	1.28335	19405.922
7	-2	1	877.208	1.28335	19407.336
6	-4	3	802.434	1.27972	17650.961
6	-2	3	802.312	1.27972	17648.273
5	-3	4	473.778	1.26691	10212.377
5	-2	4	473.813	1.26691	10213.125
6	0	2	924.183	1.26454	19846.379
3	-1	5	1205.635	1.25735	25599.965
3	-2	5	1205.635	1.25735	25599.969
7	-4	2	603.101	1.24961	12652.074
7	-3	2	602.981	1.24961	12649.559
7	-6	0	1201.571	1.24400	12494.152
7	-1	0	1200.931	1.24400	12487.487
5	-4	4	2083.709	1.23742	42895.684
5	-1	4	2083.219	1.23742	42885.562
6	-5	3	1715.051	1.23494	35172.383
6	-1	3	1714.941	1.23494	35170.102

3	0	5	473.732	1.22851	9620.185
7	-6	1	1346.176	1.22418	27157.996
7	-1	1	1346.416	1.22418	27162.826
7	-5	2	1525.911	1.22128	30649.426
7	-2	2	1525.488	1.22128	30640.922
4	-2	5	457.583	1.18873	8761.197
5	0	4	508.376	1.18412	9670.182
8	-4	0	2740.588	1.17743	25821.400
4	-3	5	630.805	1.17631	11868.205
4	-1	5	630.805	1.17631	11868.202
7	-6	2	243.161	1.16996	4535.081
7	-1	2	243.007	1.16996	4532.205
6	0	3	502.920	1.16974	9376.916
8	-5	0	335.684	1.16535	3110.807
8	-3	0	335.650	1.16535	3110.490
7	0	0	335.650	1.16535	3110.489
8	-4	1	2262.019	1.16058	41657.418
6	-3	4	2571.981	1.15994	47325.188
7	-4	3	1153.324	1.15790	21164.252
7	-3	3	1153.109	1.15790	21160.314
8	-5	1	944.784	1.14901	17138.131
8	-3	1	956.162	1.14901	17344.518
7	0	1	961.955	1.14901	17449.600
6	-4	4	1464.720	1.14838	26548.393
6	-2	4	1464.582	1.14838	26545.881
0	0	6	15927.398	1.14755	48063.625
4	0	5	192.499	1.14125	3457.810
1	0	6	1191.660	1.13636	21276.971
7	-5	3	590.825	1.13525	10534.807
7	-2	3	590.825	1.13525	10534.808
8	-6	0	321.450	1.13123	2852.013
8	-2	0	321.374	1.13123	2851.342
8	-6	1	1944.758	1.11627	33919.504
8	-2	1	1945.730	1.11627	33936.453
6	-5	4	2015.332	1.11570	35128.180
6	-1	4	2014.056	1.11570	35105.918
2	-1	6	3797.826	1.11493	66142.344
8	-4	2	3655.217	1.11407	63598.441

5	-2	5	876.216	1.10915	15164.962
5	-3	5	876.216	1.10915	15164.962
2	0	6	3921.351	1.10466	67550.258
8	-5	2	1308.507	1.10382	22521.230
8	-3	2	1308.005	1.10382	22512.590
7	0	2	1307.838	1.10382	22509.711
7	-6	3	177.425	1.09368	3023.224
7	-1	3	177.366	1.09367	3022.216
5	-4	5	77.701	1.08920	1318.447
5	-1	5	77.695	1.08920	1318.348
8	-7	0	1365.186	1.08048	11493.817
8	-1	0	1363.880	1.08048	11482.822
3	-2	6	484.663	1.07548	8127.442
3	-1	6	484.369	1.07548	8122.512
8	-6	2	480.075	1.07470	8045.517
8	-2	2	479.840	1.07470	8041.575
8	-7	1	253.252	1.06742	4220.770
8	-1	1	253.119	1.06742	4218.549
6	0	4	499.523	1.06692	8322.180
7	-4	4	304.663	1.05791	5044.893
7	-3	4	304.621	1.05791	5044.209
3	0	6	547.359	1.05726	9059.996
5	0	5	423.223	1.05232	6984.627
8	-4	3	190.263	1.04761	3131.931
9	-5	0	238.596	1.04445	1960.671
9	-4	0	238.596	1.04445	1960.671
7	-5	4	545.402	1.04055	8947.680
7	-2	4	545.115	1.04055	8942.980
8	-5	3	322.433	1.03908	5286.396
8	-3	3	322.409	1.03908	5286.015
7	0	3	322.363	1.03908	5285.253
6	-3	5	1824.729	1.03523	29871.793
9	-5	1	1898.502	1.03264	31051.023
9	-4	1	1898.763	1.03264	31055.289
4	-2	6	3675.201	1.03158	60088.922
8	-7	2	1720.932	1.03090	28130.816
8	-1	2	1720.352	1.03090	28121.324
9	-3	0	872.938	1.02774	7128.034

9	-6	0	872.938	1.02774	7128.034
6	-4	5	418.680	1.02700	6836.163
6	-2	5	418.648	1.02700	6835.628
4	-3	6	702.171	1.02343	11455.249
4	-1	6	702.023	1.02343	11452.849
8	0	0	2074.195	1.01968	16907.416
9	-6	1	988.555	1.01648	16109.012
9	-3	1	988.350	1.01648	16105.664
8	-6	3	416.685	1.01468	6788.875
8	-2	3	416.646	1.01468	6788.237
8	0	1	1996.195	1.00868	32515.316
7	-6	4	1001.018	1.00826	16305.302
7	-1	4	1000.593	1.00826	16298.376
6	-5	5	903.740	1.00342	14724.459
6	-1	5	903.684	1.00342	14723.537
4	0	6	1401.217	1.00009	22838.793
9	-5	2	688.296	0.99947	11219.782
9	-4	2	688.216	0.99947	11218.479
9	-7	0	571.422	0.99659	4659.760
9	-2	0	571.321	0.99659	4658.938
9	-7	1	1132.800	0.98631	18532.281
9	-2	1	1133.094	0.98631	18537.094
9	-6	2	1290.637	0.98480	21127.582
9	-3	2	1290.516	0.98479	21125.604
5	-2	6	1570.145	0.97828	25784.973
5	-3	6	1570.145	0.97828	25784.973
8	0	2	3117.935	0.97770	51219.570
8	-7	3	1545.288	0.97757	25386.918
8	-1	3	1544.580	0.97757	25375.281
1	0	7	1299.054	0.97654	21354.299
8	-4	4	2364.558	0.97182	38986.758
6	0	5	384.221	0.96749	6355.273
8	-5	4	345.376	0.96500	5724.318
8	-3	4	345.040	0.96500	5718.743
7	0	4	345.040	0.96500	5718.744
5	-4	6	493.567	0.96451	8183.871
5	-1	6	493.511	0.96451	8182.940
2	-1	7	933.015	0.96284	15492.809

7	-3	5	580.530	0.96075	9658.198
7	-4	5	580.530	0.96075	9658.198
9	-7	2	143.938	0.95729	2402.835
9	-2	2	143.921	0.95729	2402.549
2	0	7	262.934	0.95620	4394.229
9	-8	0	277.242	0.95476	2320.255
9	-1	0	277.278	0.95476	2320.554
9	-5	3	414.470	0.95064	6969.959
9	-4	3	414.470	0.95064	6969.962
7	-5	5	613.909	0.94770	10361.285
7	-2	5	613.930	0.94770	10361.641
9	-8	1	619.579	0.94571	10483.957
9	-1	1	620.320	0.94571	10496.506
8	-6	4	585.895	0.94536	9918.562
8	-2	4	585.528	0.94536	9912.363
10	-5	0	1526.937	0.94194	12985.722
5	0	6	3340.587	0.93862	57097.988
9	-6	3	1742.720	0.93799	29815.572
9	-3	3	1742.720	0.93799	29815.572
3	-2	7	1293.425	0.93709	22159.832
3	-1	7	1293.425	0.93709	22159.832
10	-6	0	1397.437	0.93572	11996.809
10	-4	0	1397.352	0.93572	11996.079
10	-5	1	536.827	0.93325	9254.706
8	0	3	276.006	0.93185	4769.569
10	-6	1	668.124	0.92720	11642.417
10	-4	1	668.172	0.92720	11643.255
6	-3	6	1170.314	0.92643	20422.746
3	0	7	448.599	0.92496	7850.525
7	-6	5	182.825	0.92311	3211.173
7	-1	5	182.848	0.92311	3211.578
6	-4	6	652.961	0.92052	11529.730
6	-2	6	652.795	0.92052	11526.790
9	-8	2	650.837	0.92003	11503.973
9	-1	2	650.603	0.92003	11499.830
10	-7	0	729.681	0.91778	6480.003
10	-3	0	729.559	0.91778	6478.921
8	-7	4	1284.375	0.91513	22947.691

8	-1	4	1283.853	0.91513	22938.371
9	-7	3	787.159	0.91413	14096.517
9	-2	3	787.032	0.91413	14094.262
10	-7	1	558.495	0.90974	10107.710
10	-3	1	558.406	0.90974	10106.105
10	-5	2	924.257	0.90855	16777.406
4	-2	7	563.486	0.90762	10252.765
9	0	0	850.397	0.90638	7761.489
6	-5	6	385.250	0.90342	7088.256
6	-1	6	384.991	0.90342	7083.493
10	-6	2	378.233	0.90296	6967.951
10	-4	2	378.129	0.90296	6966.051
4	-3	7	248.561	0.90206	4590.664
4	-1	7	248.520	0.90206	4589.912
9	0	1	504.942	0.89863	9417.910
8	-4	5	404.476	0.89490	7629.684
9	-5	4	0.416	0.89293	7.904
9	-4	4	0.416	0.89293	7.904
10	-8	0	136.241	0.89005	1305.244
10	-2	0	136.345	0.89005	1306.247
